# Potent Competitive Inhibitors of Ecto-5′-nucleotidase (CD73) based on 6‑(Het)aryl-7-deazapurine Ribonucleoside 5′‑*O*‑Bisphosphonates

**DOI:** 10.1021/acsptsci.5c00707

**Published:** 2025-12-22

**Authors:** Ugnė Šinkevičiu̅tė, Magdalena Šímová, Radek Staník, Lenka Poštová Slavětínská, Kristýna Blažková, Pavel Šácha, Martin Lepšík, Jan Řezáč, Jan Konvalinka, Tereza Ormsby, Michal Tichý, Michal Hocek

**Affiliations:** a Institute of Organic Chemistry and Biochemistry, Czech Academy of Sciences, Flemingovo nam. 2, Prague 6 CZ-16610, Czech Republic; b Department of Organic Chemistry, Faculty of Science, Charles University in Prague, Hlavova 8, Prague 2 CZ-12843, Czech Republic

**Keywords:** nucleotides, nucleoside bisphosphonates, pyrrolopyrimidines, CD73 inhibitors, cancer immunotherapy

## Abstract

CD73 generates immunosuppressive adenosine in the tumor microenvironment and is a promising target for cancer immunotherapy. We have designed and systematically studied diverse 2-substituted 7-deazapurine ribonucleoside 5′-*O*-bisphosphonates bearing a variety of (het)­aryl groups at position 6 and discovered their highly potent and selective CD73 inhibition activity. The most active compounds (with single-digit picomolar *K*_i_) contained bicyclic (het)­aryl groups at position 6 in combination with chlorine at position 2. Further optimization of pharmacokinetic properties identified inhibitors with low clearance, long half-life, high solubility, and excellent selectivity over CD39 and NTPDase3. They effectively suppressed adenosine formation in MDA-MB-231 cells, rescued CD8^+^ T cell activation, and were nontoxic to human fibroblasts. Overall, their profile compares favorably with **AB680**, a CD73 inhibitor currently in phase I/II clinical trials.

## Graphical abstract

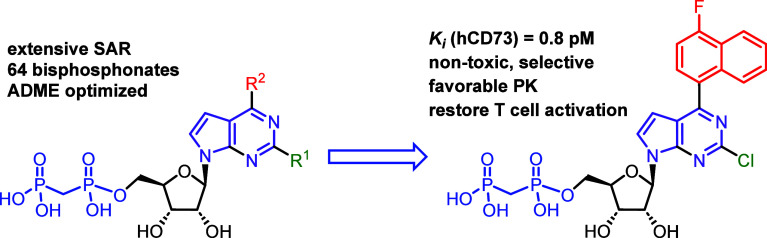

## Introduction

Tumor cells deploy sophisticated mechanisms to evade recognition and clearance by the immune system. The identification of these immune escape pathways led to the development of immune checkpoint inhibitors (ICIs) that aim to reactivate effector T cells and restore durable antitumor immunity. Yet only 20–50% of patients experience meaningful responses to ICIs,^1,2^ underscoring the need for additional therapeutic strategies. In the tumor microenvironment (TME), extracellular adenosine (Ado) has emerged as a metabolite-based checkpoint, potently suppressing antitumor immune responses. Ado originates from the sequential degradation of ATP, released from stressed or dying tumor cells. The catabolic cascade is initiated by CD39, which hydrolyzes ATP to AMP. Subsequently, CD73, a rate-limiting enzyme in this pathway, dephosphorylates AMP to yield Ado. The resulting Ado accumulates in the TME and engages A2A/B receptors on T, NK, and myeloid cells. Stimulation of A2A/B receptors elevates intracellular cAMP, which dampens cytotoxic effector functions, favors differentiation toward regulatory phenotypes, and thereby promotes tumor progression.^3^

CD73 is a GPI-anchored homodimer whose two flexible domains form the active site.^4^ In addition, a soluble form generated by proteolytic shedding exists and retains full catalytic activity.^5^ CD73 expression is upregulated by hypoxia,^6^ interferons,^7^ TGF-β,^8^ during tumor progression, and ICI treatment, where high levels typically correlate with poor prognosis.^9^ These observations have prompted clinical evaluation of CD73 blockade, most often in combination with PD-(L)­1 antibodies. During past years, many small-molecule inhibitors of CD73 have been developed, starting with the pioneering works by C. Müller and co-workers, who developed α,ß-methylene-ADP (**AOPCP**) derivatives represented by the nanomolar inhibitor **PSB-12379**.^10^ Crystallographic studies of this compound^11^ allowed rational design and extensive structure–activity relationship (SAR) studies of inhibitors based on pyrimidine, purine or pyrazolopyridine nucleoside 5′-bisphosphonates^12–14^ and monophosphonates,^15^ and led to the development of highly potent drug candidates with good metabolic stability, i.e., **PSB-12489** ^11^ and especially **AB680** (quemliclustat).^16^ Most of the SAR has been performed with purine and pyrazolopyridine nucleotides and only very few examples of 7-deazapurine nucleotides bearing alkylamino groups at position 6 were reported.^16,17^ Pyrazolopyridine nucleotide **AB680** is the most advanced small molecule in phase I/II trials. However, no CD73-targeted therapy has been approved so far. Thus, developing novel inhibitor classes capable of durable CD73 suppression still remains a valuable goal with a promising therapeutic potential.

To streamline the discovery of new inhibitors, we recently developed a novel DNA-linked inhibitor antibody assay^18^ that allowed high-throughput screening (HTS) of the internal IOCB compound library (5280 compounds) and identified two hits (nucleotide analogues **VMP145** and **VMP151,** ^19^ Figure 1) as submicromolar inhibitors of CD73. Both hits were based on 6-thiophen-2-yl-7-deazapurine ribonucleoside bearing a methylphosphonate moiety at the 5′ position, which prompted us to explore the effect of (het)­aryl group in position 6 on nucleobase in a small SAR study.^18^ Although we managed to improve the inhibitory activity by an order of magnitude and learnt about the tolerated size and orientation of (het)­aryl group, the most potent inhibitors from this nucleoside monophosphonate class represented by compound **USI506** (Figure 1) showed *K*_*i*_ only in the double-digit nanomolar range,^18^ which is still ca. 4 orders of magnitude less potent than the best nucleoside 5′-*O*-bisphosphonates, i.e. **AB680**.^16^ Considering the profound positive effect of bulky (het)­aryl groups in the nucleoside monophosphonate series^18^ and the fact that no 6-(het)­aryl derivatives of purine or deazapurine nucleoside bisphosphonates have been previously reported, it prompted us to design and explore the 6-(het)­aryl-7-deazapurine ribonucleoside motif in combination with a privileged bisphosphonate moiety in the 5′ position on ribose. Since in the previous studies on purine and pyrazolopyrimidine nucleoside bisphosphonates, a significant effect of substituent in the position 2 was observed^11–13,16^ and the most potent compounds contain a chlorine at this position, we also envisaged to systematically study the effect of substituents at position 2.

**Figure 1:**
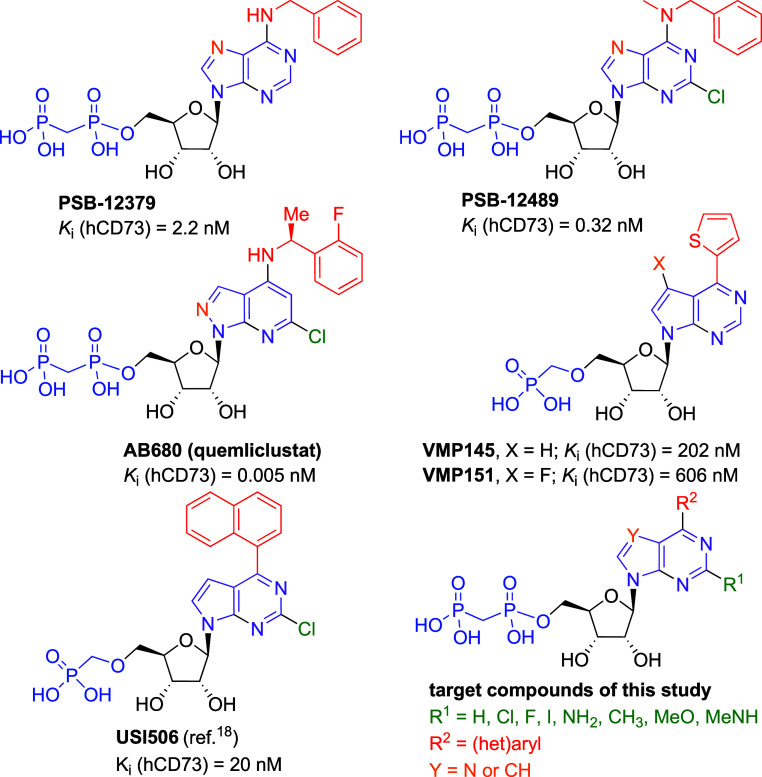
Structures of known CD73 inhibitors and target compounds.

## Results and Discussion

### Chemistry

The general synthetic strategy to the target 2-substituted 6-hetaryl-7-deazapurine ribonucleoside 5′-*O*-bisphosphonates involved the key steps of glycosylations of halogenated 7-deazapurine bases, followed by the Suzuki-Miyaura cross-coupling for introduction of the (het)­aryl substituents into position 6, and attachment of the bisphosphonate (Figure 2). We have introduced quite a wide diversity of aryl and hetaryl substituents in each series typically in several iterative rounds of synthesis and activity testing (see the SAR discussion). Additional reactions, such as diazotation or nucleophilic substitution, were used for functional group transformations and installing substituents at position 2. In some cases, these steps have been performed in a different order, depending on the reactivity of each substrate.

**Figure 2:**
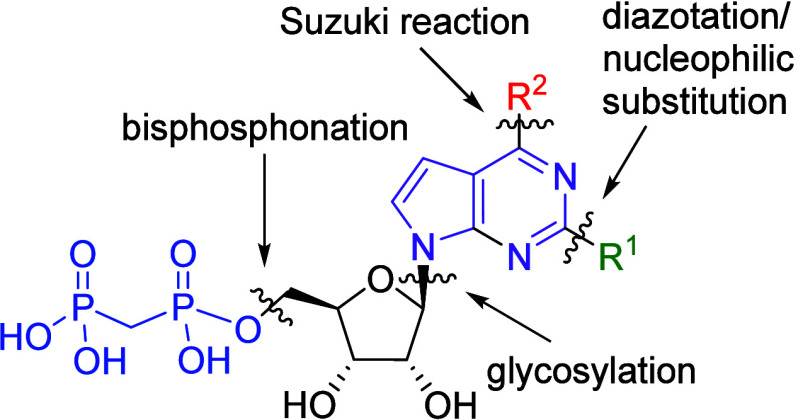
General synthetic strategy to the target 6-substituted 7-deazapurine ribonucleoside bisphosphonates.

The synthesis of parent (2-unsubstituted) 7-deazapurine derivatives started from a known protected 6-chloro-7-deazapurine ribonucleoside **1**.^20^ The silyl group was first removed using tetrabutylammonium fluoride (TBAF), followed by bisphosphonate-monoester formation using methylene bis­(phosphonic dichloride) in trimethyl phosphate. The isopropylidene group was hydrolyzed during aqueous workup, giving the desired key intermediate 6-chloro-7-deazapurine nucleoside bisphosphonate **3** in acceptable 54% yield. The aryl groups were introduced into position 6 by the Suzuki-Miyaura coupling with the corresponding (het)­arylboronic acids in the presence of Pd­(OAc)_2_ in combination with water-soluble triphenylphosphine-3,3′,3″-trisulfonic acid (TPPTS) ligand under aqueous conditions. Using an extended set of diverse mono-, di and tricyclic aryl and hetarylboronic acids, we synthesized a series of 22 examples of target 6-(het)­aryl-7-deazapurine ribonucleoside bisphosphonates **4A.1–4A.22** in acceptable yields (Scheme 1).

**Scheme 1:**
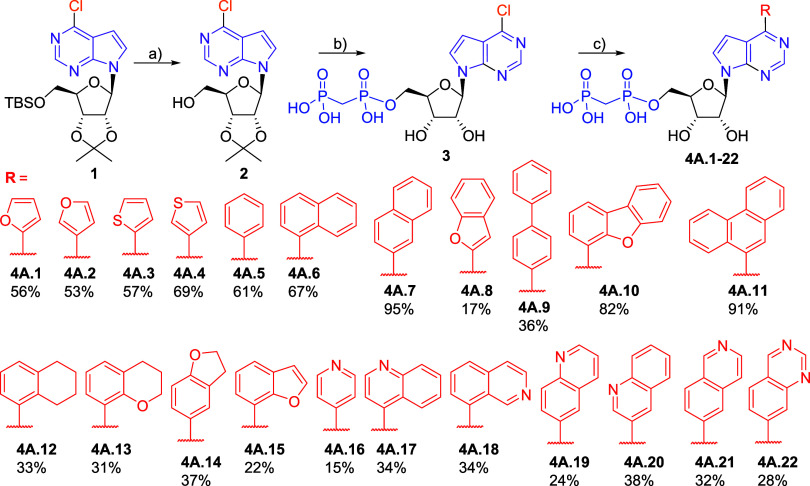
Synthesis of 6-(Het)­aryl 7-Deazapurine Ribonucleoside Bisphosphonates[Fn sch1-fn1]

a
*Reaction conditions:* a) TBAF 1 M solution in THF, 22 °C, 30 min; b) 1: methylene bis­(phosphonic dichloride), PO­(OMe)_3_, 0 °C, 3 h; 2: H_2_O, 22 °C, 1 h; c) R-B­(OH)_2_, Na_2_CO_3_, Pd­(OAc)_2_, TPPTS, H_2_O/MeCN, 80–100 °C, 1 h.

This approach could not be used for the synthesis of 2-chloro analogs because the bisphosphonylation reaction did not work on 2,6-dichloro-7-deazapurine nucleoside **5**.^19,21^ This forced us to switch the order of steps and first introduce the (het)­aryl group into position 6 by aqueous Suzuki-Miyaura cross-coupling, followed by the bisphosphonation of each derivative. Although this approach was more laborious, the 2-chloro-6-(het)­aryl nucleoside intermediates **6B.1, 6B.6–8, 6B.12, 6B.23–35** were obtained in good to excellent yields (46–98%) and were converted to the desired final bisphosphonates **7B.1, 7B.6–8, 7B.12, 7B.23–35** in acceptable yields (Scheme 2).

**Scheme 2:**
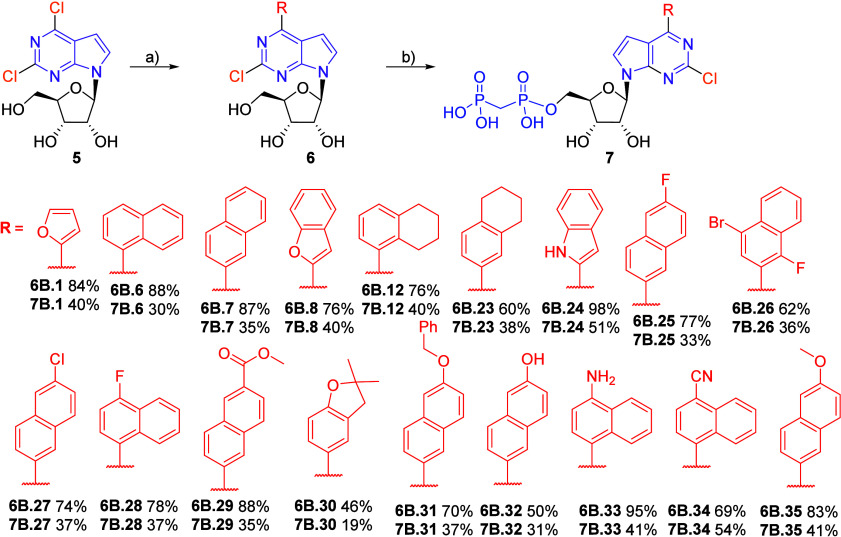
Synthesis of 2-Chloro-6-(het)­aryl 7-Deazapurine Ribonucleoside Bisphosphonates[Fn sch2-fn1]

a
*Reaction conditions:* a) R-B­(OH)_2_, Na_2_CO_3_, Pd­(OAc)_2_, TPPTS, H_2_O/MeCN, 80–100 °C, 1 h. b) 1: methylene bis­(phosphonic dichloride), PO­(OMe)_3_, 0 °C, 3 h; 2: H_2_O, 22 °C, 1 h.

For the synthesis of 2-amino derivatives, we first tested the phosphonation reaction on 2-amino-6-chloro nucleoside **8**,^19^ which gave the intermediate 2-amino-6-chloro-7-deazapurine bisphosphonate **9** in moderate 40% yield. However, when the Suzuki coupling was performed using 2-furylboronic acid, the desired 2-amino-6-(2-furyl)-7-deazapurine bisphosphonate **11C.1** was obtained in only 10% yield, due to incomplete conversion and laborious purification. To avoid these problems, the other target 2-amino-6-(het)­aryl-7-dezapurine bisphosphonates were synthesized using the alternative strategy, starting with the Suzuki-Miyaura cross-coupling reactions to produce the 2-amino-6-(het)­aryl-7-deazapurine nucleosides **10C.6–8,12** in excellent yields. These intermediates then underwent the final bisphosphonylation step, which furnished the target bisphosphonates **11C.1,6–8,12** in moderate yields (Scheme 3).

**Scheme 3:**
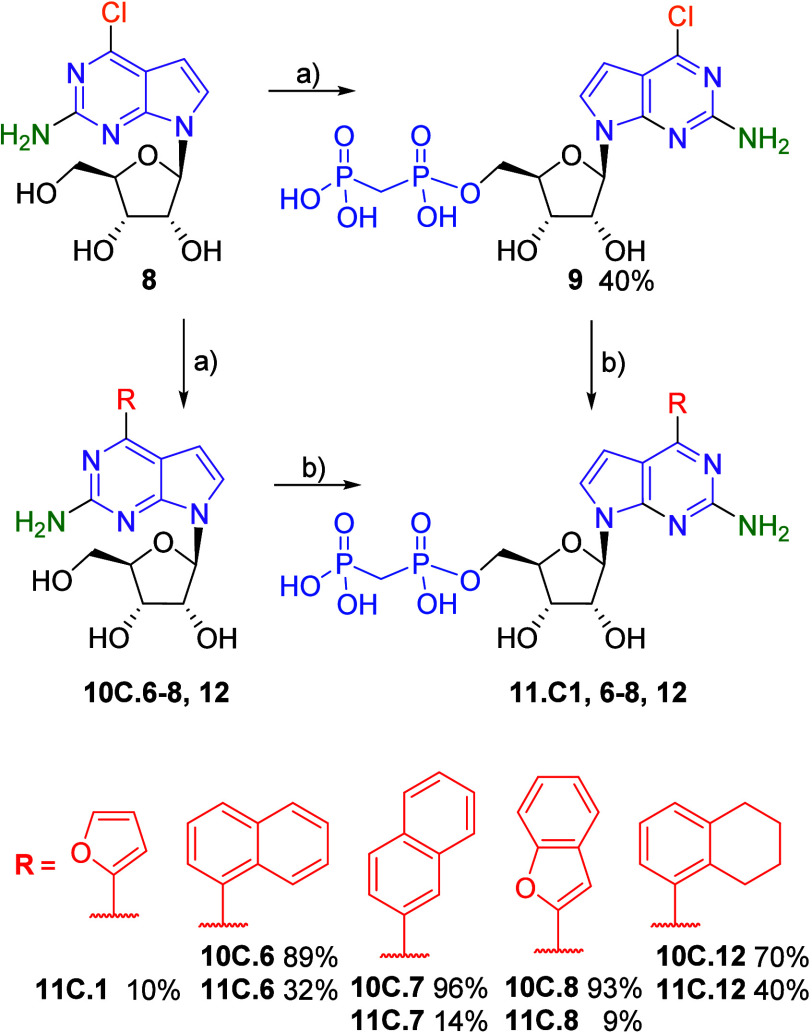
Synthesis of 2-Amino-6-(het)­aryl 7-Deazapurine Ribonucleoside Bisphosphonates[Fn sch3-fn1]

a
*Reaction conditions:* a) R-B­(OH)_2_, Na_2_CO_3_, Pd­(OAc)_2_, TPPTS, H_2_O/MeCN, 80–100 °C, 1 h. b) 1: methylene bis­(phosphonic dichloride), PO­(OMe)_3_, 0 °C, 3 h; 2: H_2_O, 22 °C, 1 h.

Synthesis of 2-fluoro derivatives started from 2-amino-6-chloro-7-deazapurine nucleoside **8**,^19^ which was subjected to diazotative fluorodeamination^22^ reaction using *tert*-butyl nitrite (TBN) and HF in pyridine, giving the corresponding 6-chloro-2-fluoro-7-deazapurine nucleoside **12** in good 61% yield. Then we performed a series of the Suzuki-Miyaura cross-coupling reactions first to afford a series of 6-het­(aryl)-2-fluoronucleosides **13D.1,6–8,12** in good to excellent yields. The bisphosphonylation reaction produced the target bisphosphonates **14D.1,6–8,12** in moderate yields (Scheme 4).

**Scheme 4:**
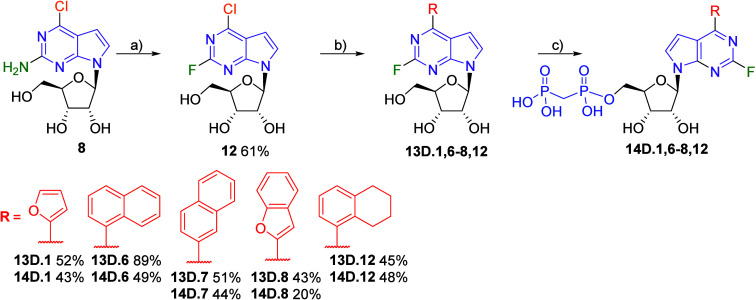
Synthesis of 2-Fluoro-6-(het)­aryl 7-Deazapurine Ribonucleoside Bisphosphonates[Fn sch4-fn1]

a
*Reaction conditions:* a) TBN, 70% HF·Py, – 30 °C, 30 min b) R-B­(OH)_2_, Na_2_CO_3_, Pd­(OAc)_2_, TPPTS, H_2_O/MeCN, 80–100 °C, 1 h. c) 1: methylene bis­(phosphonic dichloride), PO­(OMe)_3_, 0 °C, 3 h; 2: H_2_O, 22 °C, 1 h.

For the synthesis of 2-methyl-7-deazapurine derivatives, we investigated the reactivity of 6-chloro-2-methyl-7-deazapurine nucleoside **15** ^19^ in the phosphonation reaction and obtained the key intermediate 6-chloro-2-methyl bisphosphonate **16** (in 40% yield), which was then used for the synthesis of target bisphosphonates **18E.7** and **18E.12** through the Suzuki-Miyaura cross-coupling in good yields (79% and 83%, respectively). We also tested the other approach for comparison and prepared 6-aryl-2-methyl-7-deazapurine nucleosides **17E.1,6,8** in good yields. However, their bisphosphonylation gave the target bisphosphonates **18E.1,6,8** in only moderate yields (Scheme 5), which makes the first approach superior for the synthesis of 2-methyl derivatives.

**Scheme 5:**
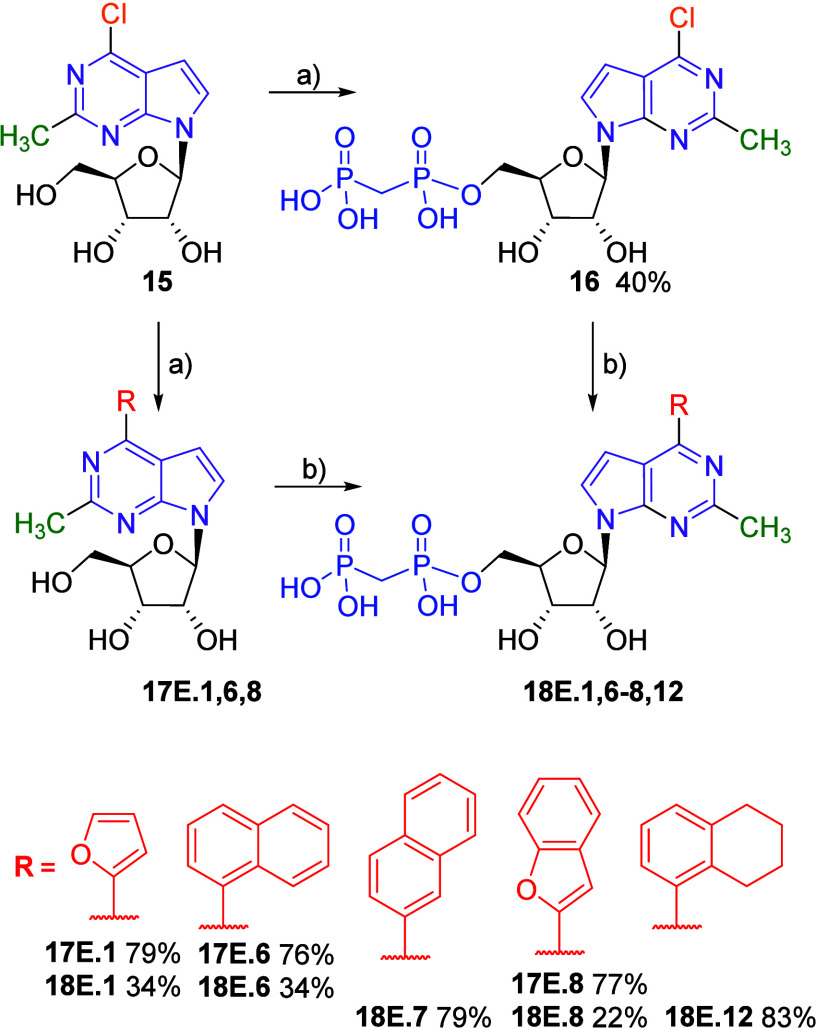
Synthesis of 2-Methyl-6-(het)­aryl 7-Deazapurine Ribonucleoside Bisphosphonates[Fn sch5-fn1]

a
*Reaction conditions:* a) R-B­(OH)_2_, Na_2_CO_3_, Pd­(OAc)_2_, TPPTS, H_2_O/MeCN, 80–100 °C, 1 h. b) 1: methylene bis­(phosphonic dichloride), PO­(OMe)_3_, 0 °C, 3 h; 2: H_2_O, 22 °C, 1 h.

To explore somewhat bulkier substituents at position 2, we synthesized methoxy and methylamino derivatives **21F.7** and **22G.7** starting from 2-chloro-6-(naphthalen-2-yl)-7-deazapurine nucleoside **7B.7**. In the first step, methoxy or methylamino substituents were introduced into position 2 by nucleophilic aromatic substitution using sodium methoxide or methylamine at elevated temperature giving 2-substituted nucleosides **19F.7** and **20G.7** in good yields (87 and 81%, respectively). Final bisphosphonylation yielded both target compounds, **21F.7** and **22G.7**, in moderate but sufficient yields (Scheme 6).

**Scheme 6:**
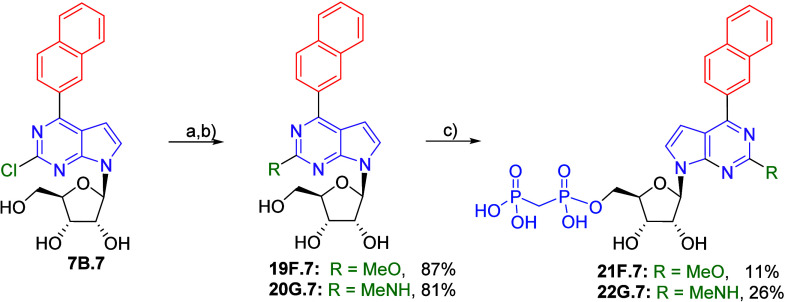
Synthesis of 2-Methoxy and 2-*N*-Methyl-6-(naphthalen-2-yl) 7-Deazapurine Ribonucleoside Bisphosphonates[Fn sch6-fn1]

a
*Reaction conditions:* a) 0.5 M NaOMe, 60 °C, 16 h; b) MeNH_2_ (33% in EtOH), TEA, 80 °C, 16 h; c) 1: methylene bis­(phosphonic dichloride), PO­(OMe)_3_, 0 °C, 3 h; 2: H_2_O, 22 °C, 1 h.

We also synthesized one example of a 2-iodo-6-aryl-7-deazapurine derivative bearing a 5,6,7,8-tetrahydronaphthalen-2-yl substituent at position 6. We started from a fully protected 2-amino-6-chloro nucleoside **23** ^23^ and first introduced the aryl substituent into position 6 by the Suzuki-Miyaura reaction. Diazotative iododeamination using isopentylnitrite, copper iodide, and iodine, followed by acidic deprotection, gave the desired nucleoside **26H.23**. Its bisphosphonylation provided the target bisphosphonate **27H.23** in good 50% yield (Scheme 7).

**Scheme 7:**
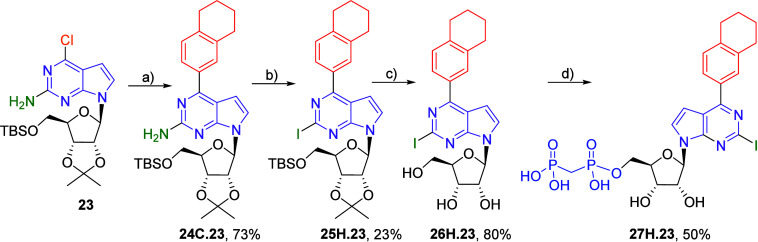
Synthesis of 2-Iodo-6-(5,6,7,8-tetrahydronaphthalen-2-yl) 7-Deazapurine Ribonucleoside Bisphosphonate **27H.23** [Fn sch7-fn1]

a
*Reaction conditions:* a) R-B­(OH)_2_, Na_2_CO_3_, Pd­(OAc)_2_, TPPTS, H_2_O/MeCN, 100 °C, 1 h; b) isopentylnitrite, CuI, I_2_, CH_2_I_2_, THF, 80 °C, 45 min; c) 75% TFA, 22 °C, 1 h; d) 1: methylene bis­(phosphonic dichloride), PO­(OMe)_3_, 0 °C, 3 h; 2: H_2_O, 22 °C, 1 h.

Most of the known CD73 inhibitors are based on a purine scaffold; thus, we decided to evaluate the effect of nucleobase and synthesized a small series of purine analogs bearing the privileged naphthyl or tetrahydronaphthyl substituents. We tested both synthetic approaches, and while the Suzuki-Miyaura coupling on nucleoside worked well and furnished the coupled products **30A.6,12** in high yields, the yield of bisphosphonate **31A.7** obtained by Suzuki coupling with naphthalen-2-boronic acid on 6-chlorobisphosphonate **29** was low (only 22%), probably due to difficult separation. Similarly, low yields of target compounds **31A.6,12** (18% and 19%, respectively) were obtained by phosphonylation of both 6-arylpurine nucleosides **30A.6,12** (Scheme 8).

**Scheme 8:**
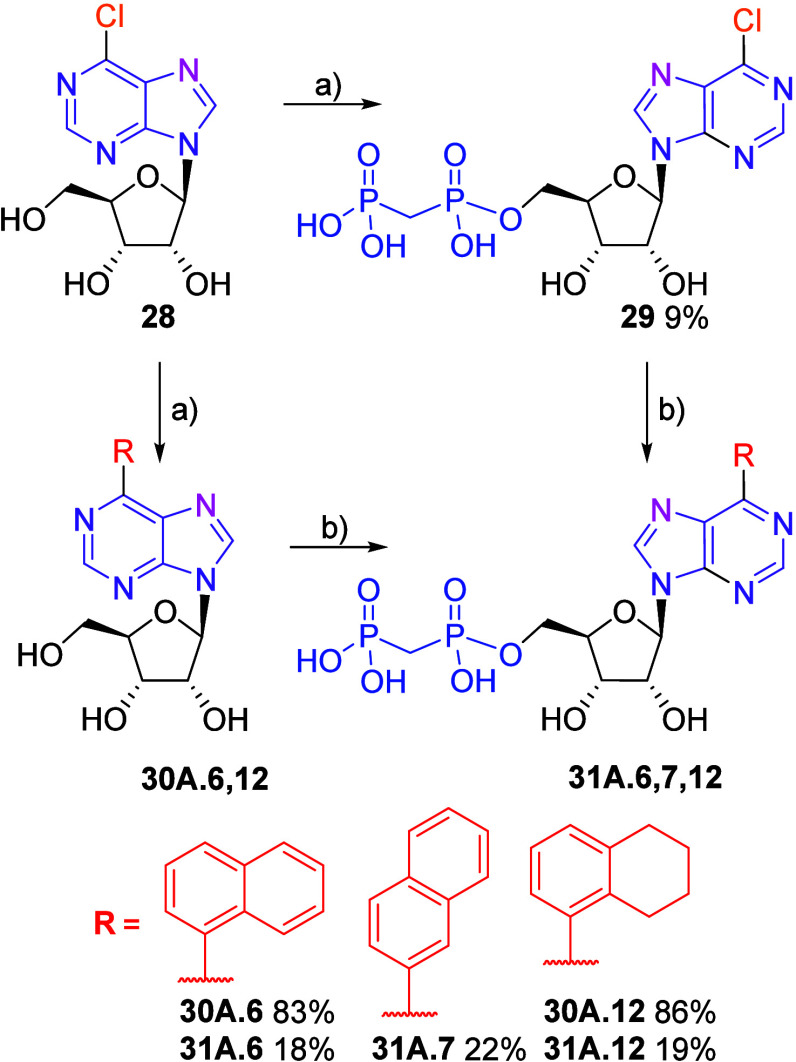
Synthesis of 6-(Het)­aryl) Purine Ribonucleoside Bisphosphonates[Fn sch8-fn1]

a
*Reaction conditions:* a) 0.5 M NaOMe, 60 °C, 16 h; b) MeNH_2_ (33% in EtOH), TEA, 80 °C, 16 h; c) 1: methylene bis­(phosphonic dichloride), PO­(OMe)_3_, 0 °C, 3 h; 2: H_2_O, 22 °C, 1 h.

Finally, we also decided to prepare a small series of sugar-modified analogs, specifically 2′-fluoroarabino nucleoside bisphosphonates in order to modulate the pharmacokinetic (PK) properties. Their synthesis started from known 2′-fluoroarabino nucleoside **32**,^24^ which was subjected to the Suzuki-Miyaura coupling reaction catalyzed by Pd­(PPh_3_)_4_ to introduce the aryl substituents into position 6, followed by deprotection of benzoyl groups by potassium carbonate. All 6-(het)­aryl nucleosides were obtained in good yields after two steps (Scheme 9). Bisphosphonylation reaction furnished the desired bisphosphonates **34A.5**–**7,12,17,23,28** in moderate yields (14–29%) after laborious purification, but all compounds were obtained in sufficient quantity and purity for biological profiling.

**Scheme 9:**
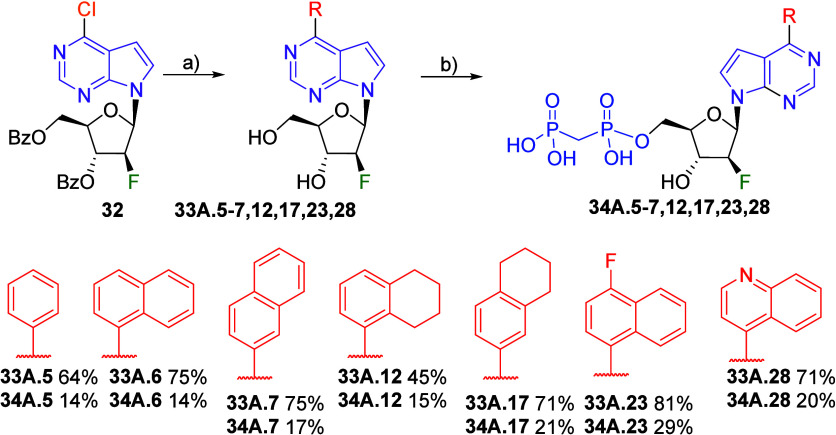
Synthesis of 6-(Het)­aryl) 7-Deazapurine 2′-Fluoroarabinonucleoside Bisphosphonates[Fn sch9-fn1]

a
*Reaction conditions*: a) 1) boronic acid (1.4 equiv), K_2_CO_3_ (2.0 equiv), THF/H_2_O 4:1, 63 °C; 2) K_2_CO_3_ (3.0 equiv), DCM/MeOH 3:7, rt; b) methylenebis­(phosphonic dichloride) (5 equiv), PO­(OMe)_3_, 0 °C.

### Biological Profiling

All the final nucleoside 5′-*O*-bisphosphonates were tested for their inhibitory potency on recombinant human CD73 (hCD73), recombinant mouse CD73 (mCD73), and the MDA-MB-231 cell line using the activity assay as described previously.^18^ The results are summarized in Tables 1–6. All the target compounds were found to be potent inhibitors of hCD73 in the enzymatic assay, they also decreased adenosine production in the human breast cancer cell line MDA-MB-231 and were all active (although ca. 1–2 orders of magnitude less potent) against murine CD73. The discussion of the SAR follows below.

**Table 1:**
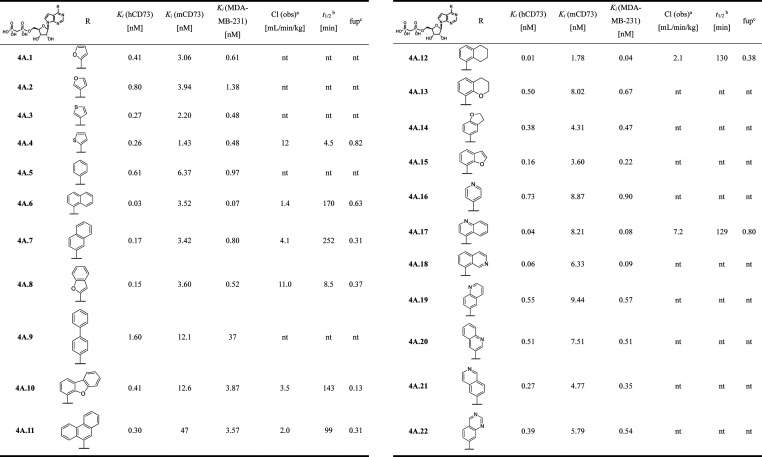
CD73 Inhibition and PK Properties of 2-Unsubstituted 7-deazapurine Bisphosphonates (**4A1–22**)

a Cl: observed clearance.

b *t*_*1/2*_ stability in plasma

c fup: fraction unbound in human plasma. nt: not tested.

**Table 2:**
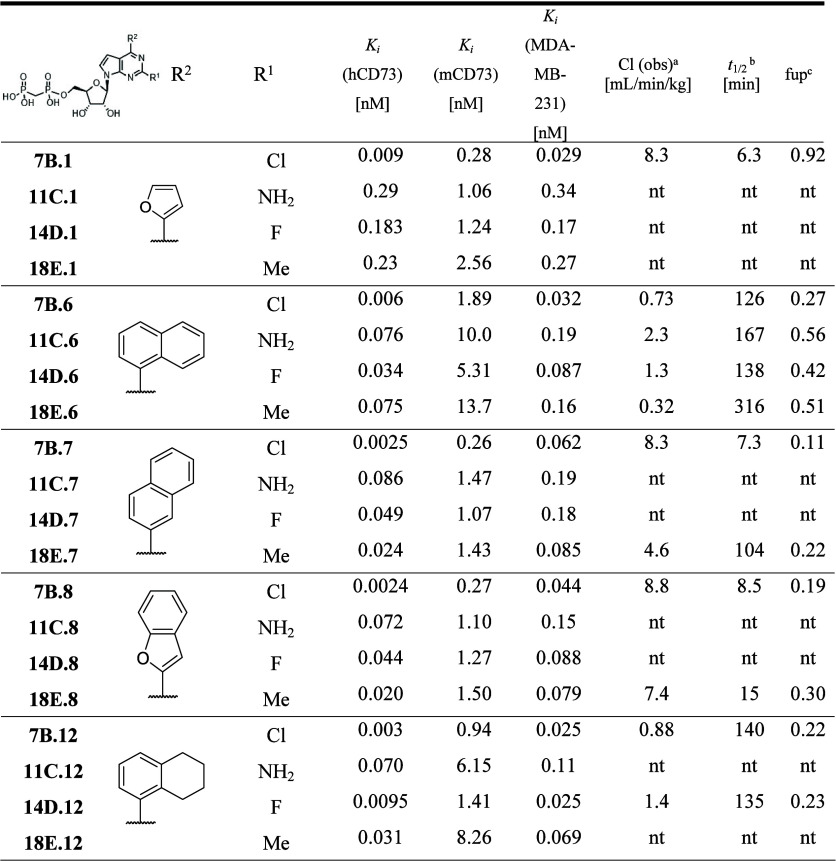
CD73 Inhibition and PK Properties of 2-Substituted 7-Deazapurine Bisphosphonates **7B, 11C, 14D, 18E**

a Cl: observed clearance.

b *t*_*1/2*_ stability in plasma.

c fup: fraction unbound in mouse plasma. nt: not tested.

**Table 3:**
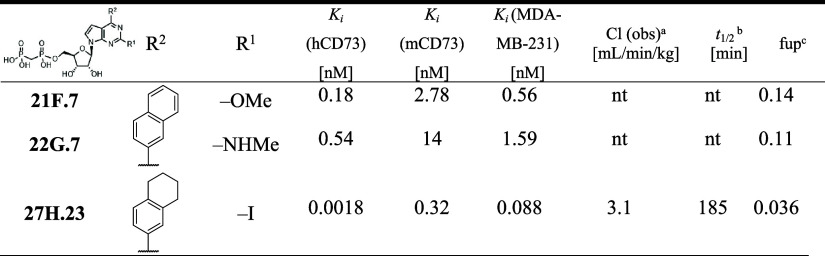
CD73 Inhibition and PK Properties of 2-Substituted 6-(Het)­aryl 7-Deazapurine Bisphosphonates (**21F.7, 22G.7, 27H.23**)

a Cl: observed clearance.

b *t*_*1/2*_ stability in plasma.

c fup: fraction unbound in human plasma. nt: not tested.

**Table 4:**
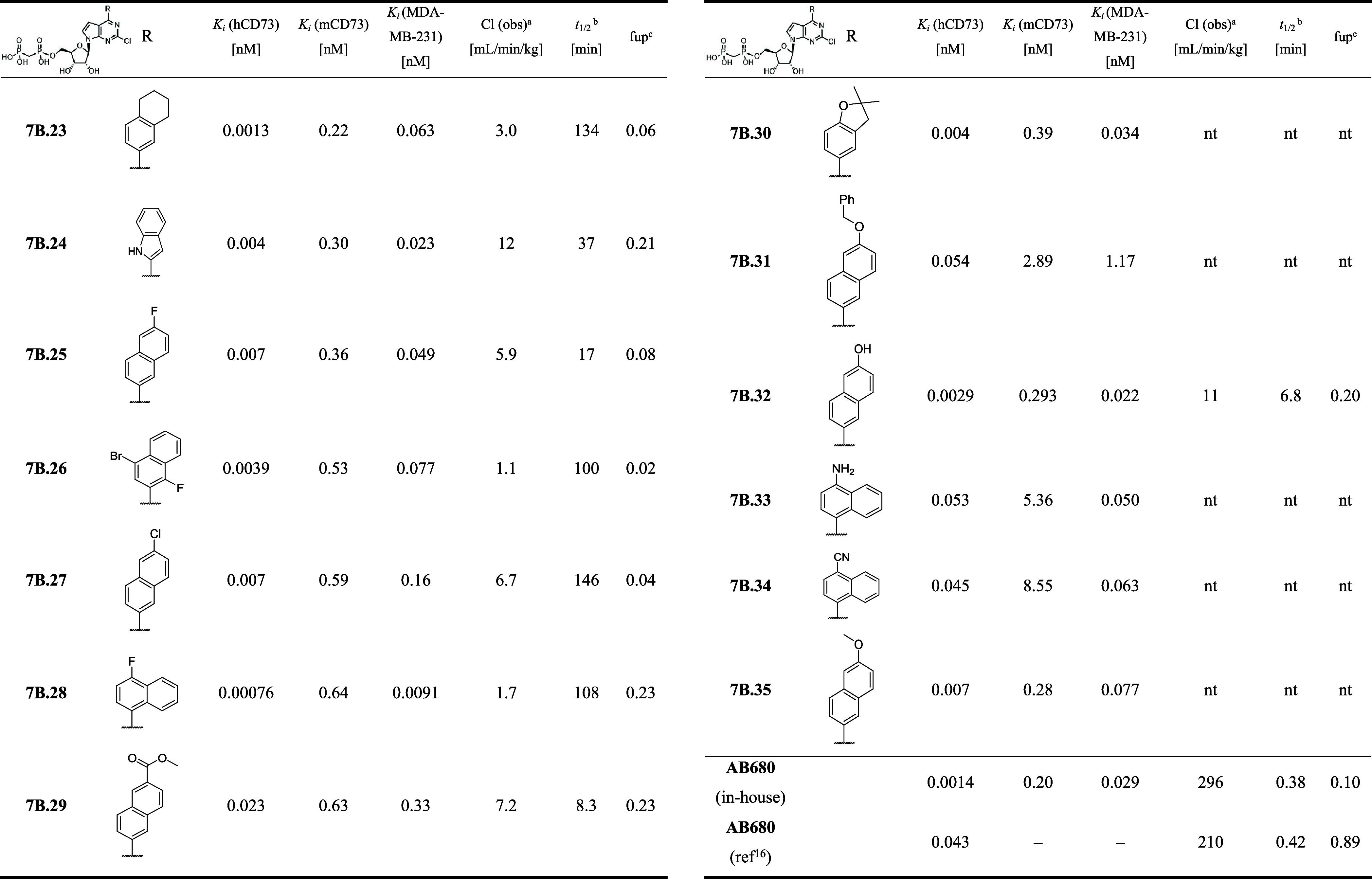
CD73 Inhibition and PK Properties of 2-Chloro Substituted 6-(Het)­aryl 7-Deazapurine Bisphosphonates (**7B.23–35**)

a Cl: observed clearance.

b *t*_*1/2*_ stability in plasma.

c fup: fraction unbound in mouse plasma. nt: not tested.

**Table 5:**
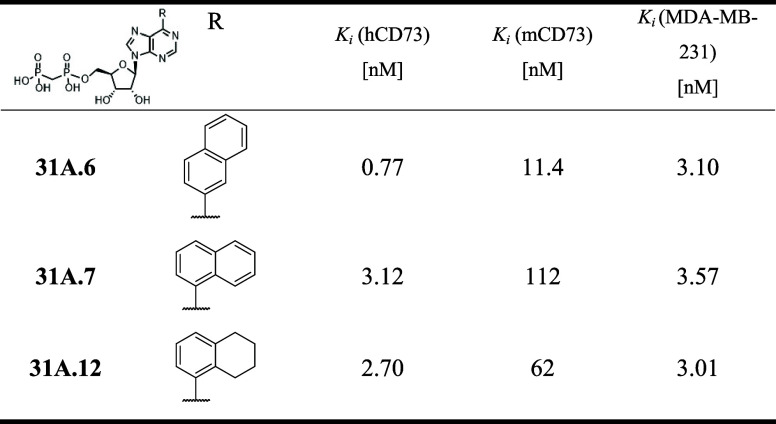
CD73 Inhibition of C-2 Unsubstituted Purine Bisphosphonates (**31I.6,7,12**)

**Table 6:**
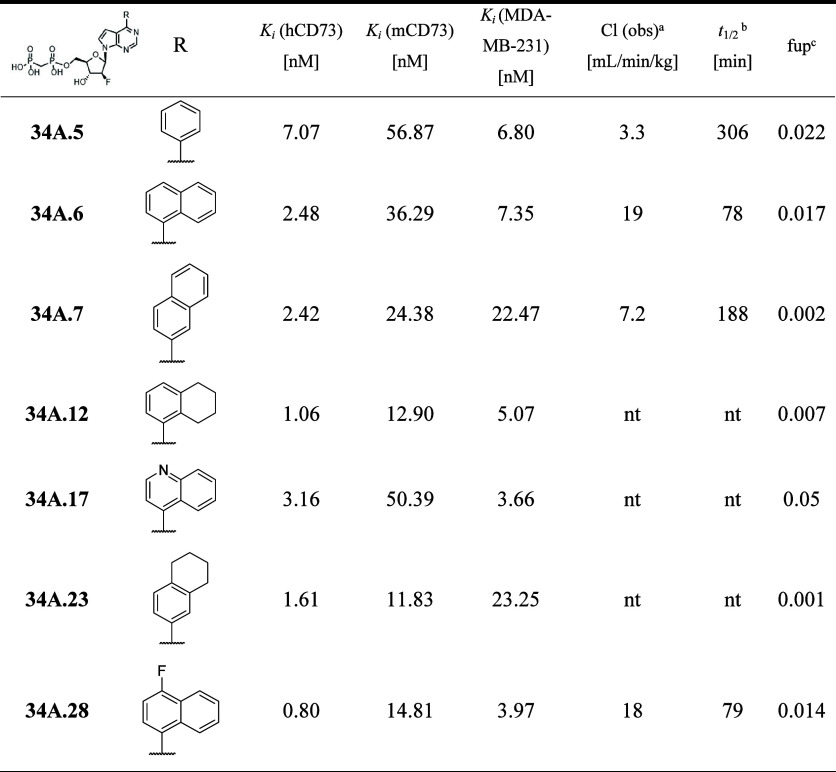
CD73 Inhibition and PK Properties of 6-(Het)­aryl 7-Deazapurine 2′-Fluoroarabino Nucleoside 5′-*O*-Bisphosphonates (**35A.5-7,12,17,23,28**)

a Cl: observed clearance.

b *t*_*1/2*_ stability in plasma.

c fup: fraction unbound in human plasma. nt: not tested.

In addition, the solubility in water as well as the stability in mouse and human plasma and microsomes were studied for all new compounds. All the data are in Tables S7–S10 in SI. All the compounds showed excellent solubility, very high plasma and microsomal stability (both mouse and human), and high to very high human plasma protein binding (>95% for most of the compounds). The binding to mouse plasma proteins was generally lower, which is common for many types of compounds.^25^ Selected compounds (**4A.7**, **4A.18**, **7B.12**, **7B.23**, **7B.28**, **18E.6**, **27H.23**) were also tested for their potential to cause drug–drug interactions and showed no inhibition against a panel of CYP isoforms up to 50 μM and for cardiotoxicity (showing no inhibition of hERG at 100 μM). Selected compounds with favorable *in vitro* data were subjected to pharmacokinetic studies in male CD-1 mice to find a candidate for further *in vivo* studies. Also these data are summarized in Tables 1–6.

All the final compounds were also evaluated for their selectivity against the ecto-nucleotidase CD39 and selected compounds also for the inhibition of NTPDase 3 – in all cases the compounds showed high selectivity toward CD73 with IC_50_ values >10 μM with selectivity index ranging from 1000 for the weakest inhibitors (fluoroarabino derivatives **34A** with *K*_*i*_ in single-digit nanomolar range) to more than 1000 000 for the most potent compounds (**7B** with single-digit picomolar *K*_*i*_ values) for both CD39 and NTPDase 3 (Table S11 in SI). All the compounds were also tested for their *in vitro* cytotoxic activity on a small panel of four cancer cell lines (CCRF-CEM, HepG2, HeLa S3, HL-60) and nonmalignant human dermal fibroblasts (NHDF). Most of the compounds showed no cytotoxicity in these cell lines, with a few exceptions that exerted moderate cytotoxicity for HL-60 at μM concentrations (Table S12 in SI).

### Structure Activity Relationship Study

Our initial investigation focused on bisphosphonate analogs of our previously discovered monophosphonate-based CD73 inhibitors.^18^ The available cocrystal structure of CD73 with **PSB12489** (PDB 6S7H) showed that the terminal phosphonate group interacts with the zinc cation in the active site, whereas the α phosphonate group is heavily involved in hydrogen bonding interactions with Asn245, Arg354, and Arg395. The nucleobase is sandwiched between Phe417 and Phe500. Modeling using software Moloc and the all-atom MAB force field^26^ showed that there is a space even for bulky hydrophobic modifications like phenanthrenyl or dibenzofuranyl at position 6 of the nucleobase. The initial series of parent 2-unsubstituted 6-(het)­aryl-7-deazapurine nucleoside bisphosphonates (**4A.1–4A.11**) showed significant improvement (by 3 orders of magnitude) of potency to hCD73, with naphth-1-yl derivative **4A.6** being the most potent inhibitor with *K*_*i*_ = 30 pM. This initiated our further design and development to deliver highly potent, stable, and selective CD73 inhibitors suitable for intravenous (i.v.) administration.

As the (het)­aryl substituent at position 6 is not involved in stacking, we first extended the series by several bicyclic and partially saturated substituents (**4A.12**–**4A.14**) to better fill the pocket, followed by several quinolines and isoquinolines to block the potential metabolic sites. This led to the identification of tetrahydronaphth-1-yl derivative **4A.12**, with 3-fold better potency (*K*_*i*_ = 10 pM, Table 1). Having several potent inhibitors in hand with high *in vitro* stability in mouse and human plasma and microsomes, we then focused on *in vivo* pharmacokinetic (PK) properties in mice, where we found significant differences between the tested compounds. Thiophen-2-yl **4A.4** and benzofuran-2-yl **4A.8** derivatives showed poor PK properties with high clearance and very short half-lives (5 and 9 min, respectively, Table 1). Inhibitors bearing bulky hetaryls like dibenzofuranyl **4A.10** and phenanthrenyl **4A.11** displayed moderate clearance. Both naphthalenyl derivatives **4A.6** and **4A.7,** showed lower clearance (1.4 and 4.1, respectively) and longer half-lives (170 and 252 min, respectively), but napht-1-yl analog **4A.6** is 6 times more potent than naphthalen-2-yl derivative **4A.7**. Introduction of a nitrogen atom leading to quinolin-4-yl **4A.17** and isoquinolin-8-yl **4A.18** derivatives gave compounds with a higher clearance rate than naphthalenyl analogs, thus naphth-1-yl derivative **4A.6** had the lowest clearance from all the 2-unsubstituted derivatives. Its partially saturated tetrahydronaphthyl derivative **4A.12** is 3 times more potent (*K*_*i*_ = 30 pM vs 10 pM), but with higher clearance (1.4 vs 2.1) and shorter half-life (170 min vs 130 min).

Previous works show^16^ that bisphosphonates based on purine scaffold bind into the open form of CD73, which closes upon inhibitor binding. The nucleobase is stacked between Phe-417 and Phe-500, and the hydrogen in position 2 is pointing to the hydrophobic C-2 pocket, which is filled with several water molecules. Our modeling confirmed a similar binding mode of our inhibitors, so we tried to fill the C-2 pocket with several small substituents, including amino, methyl, fluoro, and chloro. We prepared and tested a small library of 20 compounds (5 series of compounds differing in the 6-(het)­aryl group, each with Cl, Me, F, and NH_2_ group at position 2) to evaluate the effect of the C-2 substituent on both inhibiting CD73 and PK properties (Table 2). The 2-chloro derivatives **7B.1,6–8,12** were always the most active compounds in each series. However, the effect of other substituents varied. In the case of benzofuran-2-yl **7B.8, 11C.8, 14D.8, 18E.8** and naphthalen-2-yl **7B.7, 11C.7, 14D.7, 18E.7** derivatives, all the C-2 substituents improved the binding. The potency generally followed the trend Cl > Me > *F* > NH_2_ > H, with the 2-chloro derivatives **7B.7** and **7B.8** being the most potent inhibitors with *K*_*i*_ = 3 pM. On the other hand, in the tetrahydronaphth-1-yl series **7B.12, 11C.12, 14D.12, 18E.12**, the NH_2_ and Me derivatives **11C.12** and **18E.12**, respectively, showed reduced potency, whereas both halo derivatives **7B.12** and **14D.12** were more potent than the parent compound. Similarly, in the naphth-1-yl series **7B.6, 11C.6, 14D.6, 18E.6**, both the NH_2_ and Me derivatives **11C.6** and **18E.6** were less potent than the 2-unsubstituted analog. The fluoro derivative **14D.6** showed similar potency to the 2-unsubstituted derivative (**4A.6**), and only the introduction of a chlorine atom in **7B.6** improved the potency (Table 2 and Figure 3).

**Figure 3:**
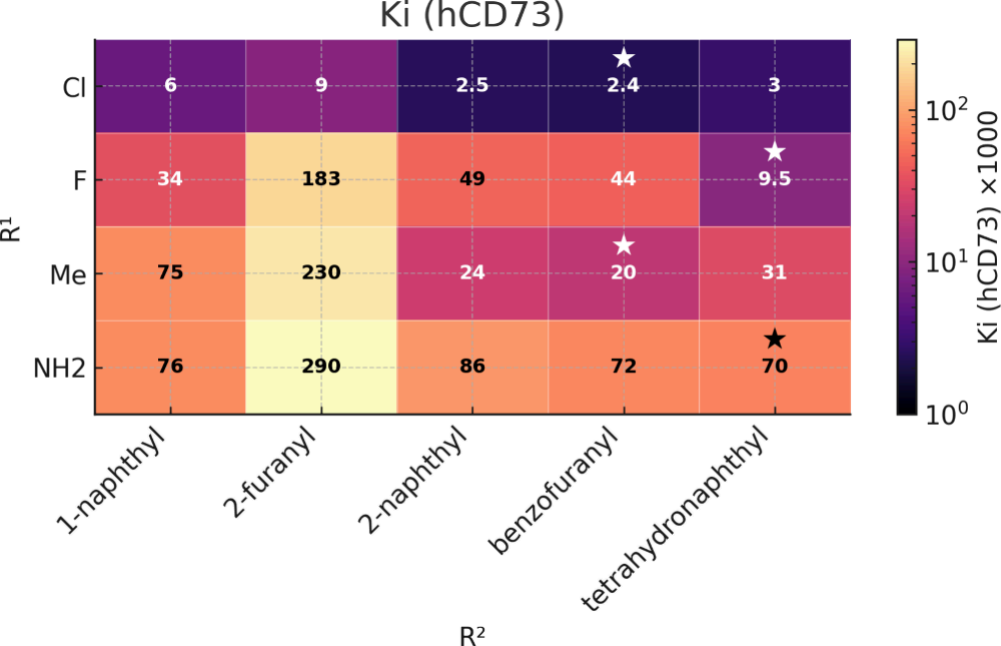
SAR in position 2. *K*_*i*_ values given in pM. ★/**☆** – the most potent inhibitor in each series.

We did not focus only on improving potency, but also on PK properties. The data in Table 2 show that replacing chlorine with both fluorine or amino group in position 2 leads to higher clearance, on the other hand, methyl substituent decreases the clearance and leads to higher half-life, which is best demonstrated in a series of 1-naphthyl analogs **7B.6, 11C.6, 14D.6, 18E.6**. At the end, the methyl derivative **18E.6** showed the most favorable PK profile from the whole study. Although our CD73 inhibitors suffer from very high plasma protein binding (see fraction unbound in plasma in Table 2 or Tables S7–S10 in SI), it is possible to reach sufficient unbound plasma concentration even with a low dose (1 mg/kg IV) due to the very high potency (see Figure S1 in SI). Together with the fact that all the target compounds showed high stability in plasma and microsomes, no CYP and hERG inhibition, excellent selectivity against CD39 and NTPDase 3, the main selection criterion was correlation of unbound exposure in plasma and potency against CD73. This makes the methyl derivative **18E.6** our lead compound for further *in vivo* efficacy studies.

In the series of inhibitors **7B.1, 11C.1, 14D.1, 18E.1** bearing only a small furan-2-yl substituent in position 6, all C-2 substituted analogues were more potent than the parent 2-unsubstituted compound **4A.1**, with the following trend: Cl > *F* > Me > NH_2_ > H. To extend our SAR, we synthesized 2-methoxy and 2-*N*-methylamino analogs of **4A.7**. These modifications led to improved potency in a previously reported *N*-6 benzyl purine bisphosphonates,^11,12^ but in the case of 6-naphthyl-7-deazapurine nucleoside bisphosphonates the inhibitory activity was only moderate (Table 3). As 2-chloro derivatives showed better potency and PK properties than fluorinated analogs, we also synthesized 2-iodo analog **27H.23** with a tetrahydronaphthalen-1-yl substituent in position 6, which has excellent potency (*K*_*i*_ = 1.8 pM) and clearance (3.1) comparable to the 2-chloro derivative (Tables 3 and 4).

Evaluation of *in vivo* PK properties showed that the effect of the aryl group in position 6 is more important than the effect of a small substituent in position 2. In the series of naphth-1-yl derivatives **7B.6, 11C.6, 14D.6, 18E.6**, the introduction of an amino group worsened the PK properties, while fluorine in **14D.6** showed very little effect (Table 2). On the other hand, chlorine significantly reduced the clearance in compound **7B.6**, and methyl derivative **18E.6** was even better; it showed superior PK properties to **AB680** – clearance only 0.32 and half-life 316 min. Lipophilic methyl substituent improved PK properties also in benzofuranyl **7B.8, 11C.8, 14D.8, 18E.8** and 2-naphthyl **7B.7, 11C.7, 14D.7, 18E.7** series, suggesting minimal metabolism of nucleoside bisphosphonates and renal excretion. On the other hand, methyl derivatives **18E.1,6–8,12** are less potent than their chloro analogs **7B.1,6–8,12** (Table 2).

These data clearly showed that chlorine is a privileged substituent for position 2 and prompted us to extend the series of 2-chloro derivatives to further improve the potency and PK properties of our inhibitors. We focused on various further substituted naphthalenyl groups and other extended aromatics and synthesized another 13 compounds **7B.23–35**. Tetrahydronaphtalen-1-yl derivative **7B.12** bearing a chlorine substituent in position 2 was one of the most potent inhibitors identified in the original series. Isomeric tetrahydronaphthalen-1-yl derivative **7B.23** is even more potent with *K*_*i*_ = 1.3 pM (Table 4). Its modeled binding mode is shown in Figure 4A. From all the derivatives bearing substituted naphthalenyl groups, the most potent compounds always contain an additional substituent, i.e. halogen atom (**7B.25–28**) or hydroxy and methoxy group (**7B.32, 7B.35**). On the other hand, introduction of cyano, amino, or methylcarboxylate groups led to the decrease in potency (**7B.34**, **7B.33**, **7B.29**, Table 4). Compound **7B.28** bearing 4-fluoronaphth-1-yl was identified as the most potent compound from this study with *K*_*i*_ = 0.8 pM and good PK properties (half-life of 108 min and clearance 1.7 mL/min/kg).

**Figure 4:**
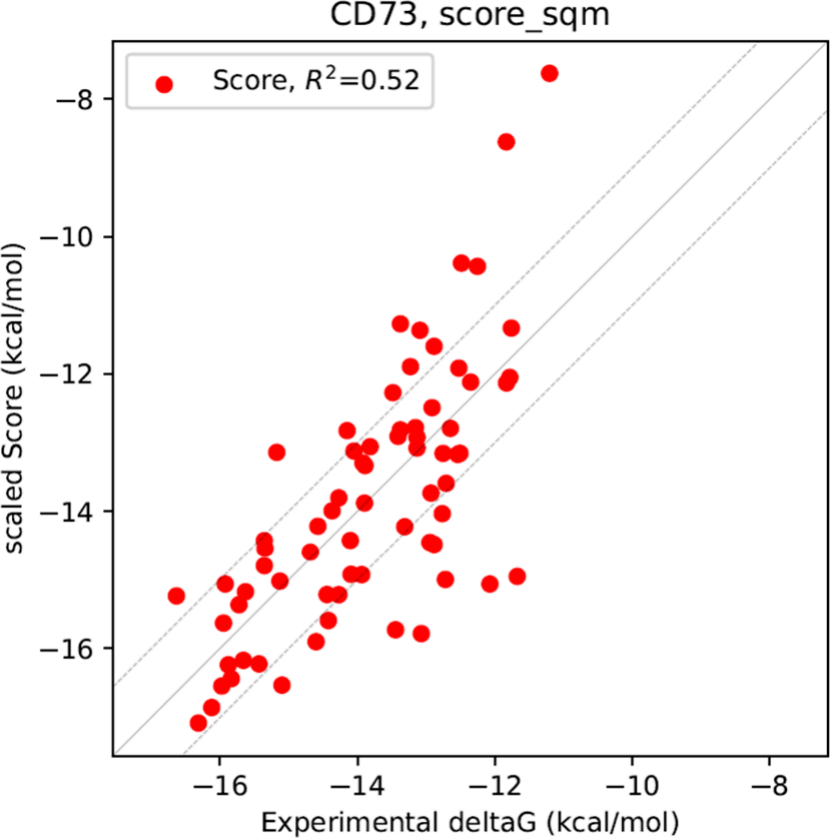
Calculated SQM2.20 scores were scaled by regression factor obtained from the series and shifted so that their average value matches the average experimental binding free energy (Δ*G*_exp_). The individual Δ*G*_exp_ values were obtained from the equation Δ*G*_exp_ = RT ln *K*_i_, where R is the universal gas constant, T is the temperature and *K*_i_ was measured in this work. The solid line represents the least-squares linear fit of all the points, and its coefficient of determination (R^2^) is shown. The two dashed lines are separated by 1 kcal/mol on either side.

For the comparison with previous works,^10–14,27^ we also tried to replace the 7-deazapurine nucleobase with purine, and synthesized three examples of 6-naphthyl- or 6-(tetrahydronaphthyl)­purine nucleoside bisphosphonates **31A.6,7,12**. Data in Table 5 show that the purine derivatives show inhibition of hCD73 at single-digit nanomolar concentrations, which is a significant (3 orders of magnitude) decrease in potency compared to 7-deazapurines. This shows that, indeed, the 7-deazapurine moiety is a superior scaffold for our CD73 inhibitors.

Although we identified several single-digit picomolar inhibitors of CD73 with reasonable PK properties, we wanted to expand our SAR and hopefully improve the PK properties by preparing more lipophilic derivatives fluorinated on ribose. It is known that the (*R*)-2′–OH group can be replaced by (*S*)-fluorine without a significant drop in potency.^16^ This is possible due to a unique puckered conformation of 2′-fluoroarabinose, in which the 3′–OH can compensate for the loss of a hydrogen bond with 2′–OH.^16^ To test this in combination with several 6-(het)­aryl substituents, we synthesized a small series of 2′-fluoroarabino analogs bearing simple phenyl, quinolin-4-yl, both isomers of very potent naphthalenyl and tetrahydronaphthalenyl derivatives, and an analog of the most active inhibitor **7B.28** in the ribonucleoside series. However, all 2-fluoroarabino derivatives **34A.5–7,12,17,23,28** inhibited CD73 only at single-digit nanomolar concentrations, and their PK properties were also significantly worse compared to ribose derivatives (Table 6).

### Molecular Modeling

Sixty-seven nucleoside bisphosphonate inhibitors were modeled into the active site of CD73 based on the crystal structures in refs 11 and 16 (codes 6S7H, 6Z9B) in more than 550 conformations in total. The SQM2.20 quantum-mechanical optimization defined the most probable conformation of each ligand in the CD73 binding site (comparison by TotalSQM energy) and the SQM2.20 scoring^28^ provided an affinity estimate. The SQM2.20-optimized structures are made available in the public repository.^29^ The scores and their terms are listed in Table 7 for a few representative compounds and Tab. S14 for all the modeled inhibitors. The coefficient of determination between the SQM2.20 scores and the experimental binding free energies R^2^ is 0.52, indicating a reasonable model predictivity (Figure 4).

**Table 7:** Experimental Affinities as Gibbs Free Energies (dG_exp) and SQM2.20 Scores and Their Terms (kcal/mol) for the 2-Substituted 6-(2-Furyl) 7-Deazapurine CD73 Inhibitors

code	dG_exp	score_shift_scale	score_sqm	Int_e	Int_e_vac	Solv_e_int
**4A.1**	–12.9	–11.6	–83.7	–88.3	–599.9	511.6
**7B.1**	–15.2	–13.1	–87.0	–91.8	–600.1	508.3
**18E.1**	–13.2	–11.9	–84.3	–89.9	–606.2	516.3
**14D.1**	–13.4	–11.3	–83.0	–87.2	–595.4	508.2
**11C.1**	–13.1	–11.4	–83.2	–87.1	–608.2	521.1

Due to the open nature of the CD73 binding site and the hydrophobicity of the moieties present in our target compounds, there are no specific interactions (hydrogen bonds) between the protein and the 2- and 6-substituents of our inhibitors. Instead, the inhibitor binding potency is defined by the effectiveness of filling the protein pocket by the 6-substituents and perturbing water molecule network and electronic effects induced by the 2-substituents. Figure 5A shows that the binding mode of the most potent inhibitor **7B.28** to CD73 closely aligns with that of one of the original ligand from the reported crystal structures.^11,16^ Figure 5B visualizes the water molecule network filling the pocket around 2-substituents which will be differently affected by the substituents (this effect is not captured by our modeling which uses implicit solvent model). Other effects, such as influence of the 2-substituent on the hydrogen bond strength with Asn390 and stacking of the 7-deazapurine core with Phe417 and Phe500, play roles and their quantitative description is included in the SQM2.20 score. We note that the bisphosphonate moiety binds to two zinc ions as well as Arg354 and Arg395 (Figure 5C) – these interactions are common to all the studied inhibitors.

**Figure 5:**
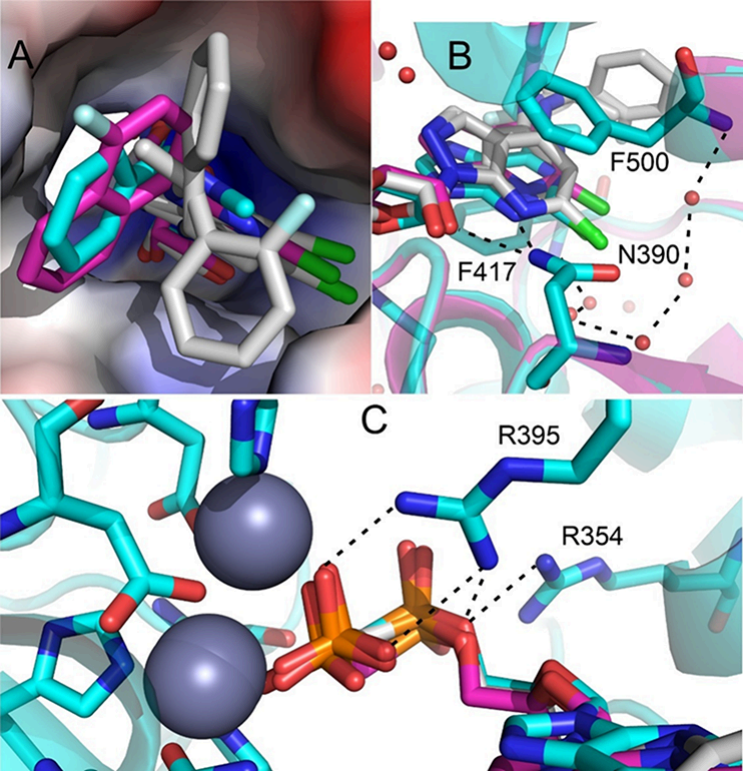
Overlay of the crystal ligands **PSB12489** (PDB code 6S7H, ref 11; carbon in cyan) and **AB680** (PDB code 6Z9D, ref 16, carbon in gray) with the SQM2.20 optimized **7B.28** (carbon in magenta) in the binding site of CD73. **A**. The surface of CD73 is colored by the electrostatic potential from the most negative in red via neutral in gray to the most positive in blue. **B.** Dashed lines show atoms involved in hydrogen bonding. **C.** Two zinc ions are shown as gray spheres. Color coding: nitrogen in blue, chlorine in green, phosphorus in orange.

QM scoring can give useful insights into the fine structural and energetic reasons for the features observed in SAR. A comparison of the three purine-based compounds with their 7-deazapurine counterparts (**31A.7** vs **4A.7, 31A.6** vs **4A.6** and **31A.12** vs **4A.12**) reveals that the best poses of each ligand selected by the TotalSQM energies differ in the orientation of the 6-substituent. The SQM score (score_sqm) differences are within the error bounds of the method for the two former pairs and only in the latter clearly show the better potency of the 7-deazapurine scaffold in agreement with the experiment (Tab. S14).

Regarding the substituents in the C-2 position in the 7-deazapurine series, we compared 2H (**4A.1**), 2Cl (**7B.1**), 2Me (**18E.1**), 2F (**14D.1**), and 2NH_2_ (**11C.1**) combined with 6-(2-furyl) substituents. While there are no structural differences in the best poses of each ligand, the SQM scores (score_sqm) and interaction energies in solvent (int_e) clearly show the superiority of the 2Cl substituent (Table 7). It is interesting to note that this is not caused simply by its best gas-phase interaction energy term (2Me and 2NH_2_ have better int_e_vac terms) but by its favorable balance with the desolvation penalty (solv_e_int). Such compensation has been observed before.^30^ We note that the C-2 position substituents may also perturb the water network in the cavity (Figure 5B; the effect we may not fully describe with the implicit solvent model) and thus modulate the activity of the compounds as has been shown before.^12^

In search for the reasons of the highest affinity of **7B.28** bearing 4-fluoronaphth-1-yl in position 6, we compared it with the less potent **7B.33** (4-aminonaphth-1-yl substituent). The poses selected by TotalSQM energy differed by the orientation of the 4-substituted naphth-1-yl group. Despite the lack of direct protein-inhibitor noncovalent interactions, both the SQM score and the interaction energy correctly identify **7B.28** as more potent than **7B.33.** However, the gas-phase interaction energy of **7B.28** was lower with respect to that of **7B.33**, whereas its desolvation penalty was lower (Tab. S14).

### Functional Assay

Selected highly potent CD73 inhibitors (**4A.7, 4A.18, 7B.6, 7B.12, 7B.23, 7B.28, 18E.6, 18E.7, 27H.23)** were tested for their *in vitro* cytotoxicity against the MDA-MB-231 breast cancer cell line (Figure S2 in SI) and all the tested compounds were found to be nontoxic in this assay. Based on these findings, we conclude that the nucleoside bisphosphonates are not intrinsically cytotoxic. Therefore, we hypothesize that the primary mechanism of action of our compounds is the mitigation of adenosine-mediated immunosuppression of immune cells in the TME, similar to what has been demonstrated for **AB680**.^31^ To test this hypothesis, selected compounds were evaluated in a functional assay, that monitors CD8+ T cell activation^31^ using upregulation of the activation marker CD25 and cytokine production. CD8+ T cells were isolated from a healthy human donor, characterized for CD73 and CD25 expression (Figure S3A in SI), and stimulated with CD3/CD28 beads to mimic T cell receptor (TCR) activation. Under these standard conditions, CD25 expression increases, accompanied by elevated secretion of IFN-γ and granzyme B (Figure 6 and Figure S3B). However, when AMP is added to the culture medium during stimulation, it is hydrolyzed by CD73 to produce immunosuppressive adenosine, which dampens T cell activation (Figure 6 and Figure S3B). To ensure reliable detection of adenosine, the adenosine deaminase inhibitor EHNA was included in the assay. Co-administration of AMP with a CD73 inhibitor restored T cell activation (Figure 6). All selected inhibitors were able to partially rescue CD8+ T cell activation. Minor differences were observed in cytokine rescue efficacy, with compounds **18E.6** and **18E.7** showing the weakest effects; however, these results are difficult to interpret due to substantial donor-to-donor variability in activation responses. In separate CD8^+^ T-cell activation assays performed under analogous stimulation conditions, **AB680** at 500 nM elicited a median 41% increase in CD25 expression, which is comparable to the activation levels observed with the CD73 inhibitors described here, although donor-to-donor variability precludes robust cross-experiment EC_5__0_ comparisons. In separate CD8^+^ T-cell activation assays performed under analogous stimulation conditions, AB680 at 500 nM elicited a median 41% increase in CD25 expression, which is comparable to the activation levels observed with the CD73 inhibitors described here, although donor-to-donor variability precludes robust cross-experiment EC_5__0_ comparisons.

Checkpoint blockade in combination with CD73 inhibition has been shown to synergistically enhance antitumor immunity and reduce tumor growth in multiple preclinical models.^32–34^ Mechanistically, inhibition of CD73 decreases adenosine-mediated suppression of effector lymphocytes, while PD-1/PD-L1 blockade restores T-cell receptor signaling and cytotoxic activity. Recent work further demonstrates that CD73 inhibition not only limits adenosine-dependent impairment of CD8+ T cells but also reshapes the immunosuppressive compartment of the tumor microenvironment by reducing tumor-infiltrating regulatory T cells (Treg) and dampening Treg-associated chemokine programs such as CCL5, thereby diminishing their recruitment and persistence.^34^ Because our inhibitors effectively rescue CD8^+^ T-cell activation *in vitro*, they are expected to act cooperatively with PD-1/PD-L1 inhibitors to reprogram the tumor microenvironment toward a more immunostimulatory state conducive to durable antitumor responses.

Collectively, the biochemical, mechanistic, cellular, and mouse PK data provide a coherent foundation to support prospective *in vivo* efficacy testing of a selected preclinical candidate from this class of competitive CD73 inhibitors in the future.

**Figure 6:**
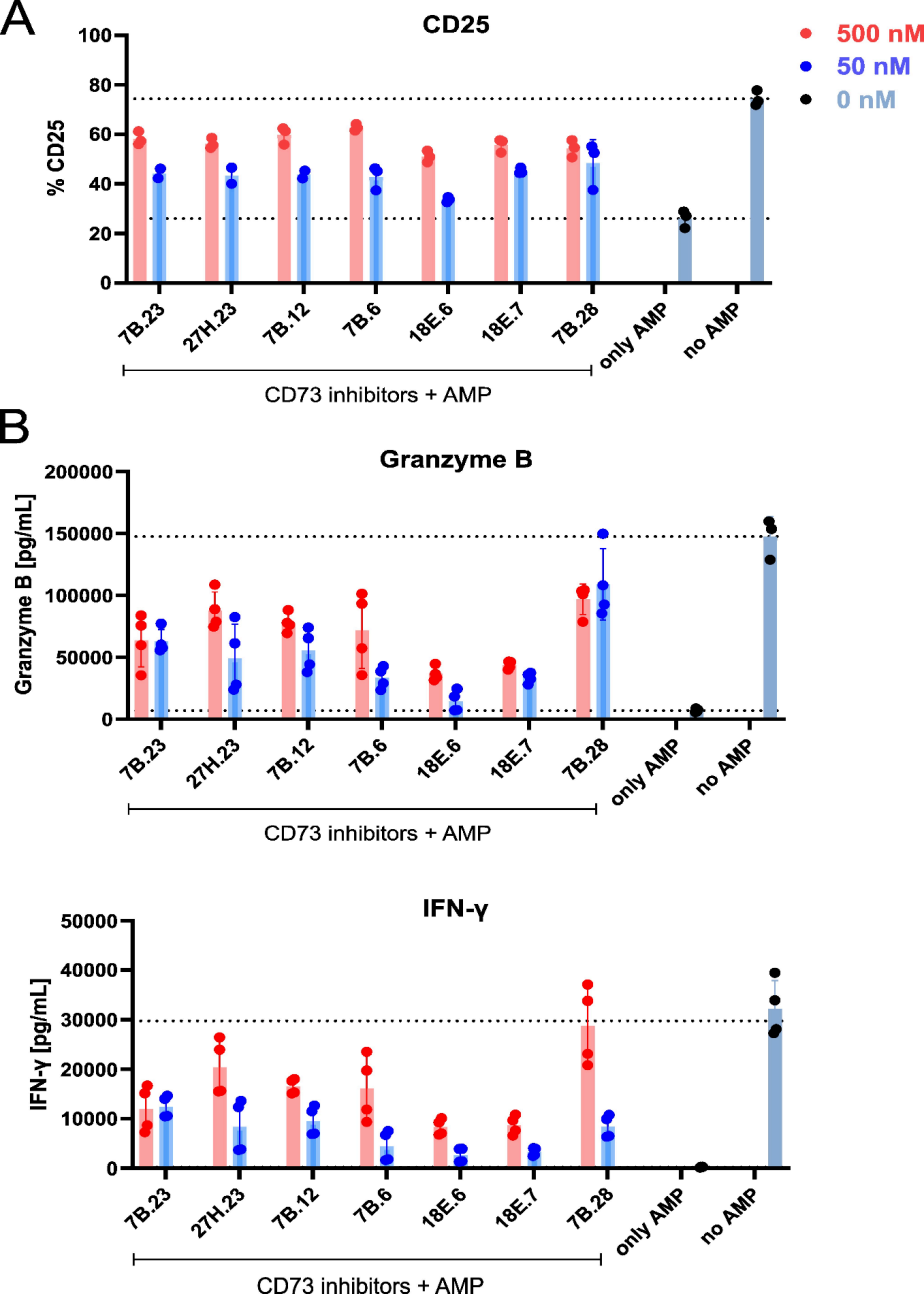
**CD73 inhibition partially reverses the immunosuppressive effect of adenosine generated from AMP in CD8** ^**+**^ **T cells.** CD8^+^ T cells were activated using CD3/CD28 activation beads in the presence of 2.5 μM EHNA, with or without 1 mM AMP and CD73 inhibitors (500 nM or 50 nM). **A. CD25 expression.** The percentage of CD8^+^ T cells expressing the activation marker CD25 was measured by flow cytometry 60 h after activation. Data represent technical triplicates. **B. Cytokine secretion.** Granzyme B and IFN-γ levels in the culture supernatant were quantified by ELISA 60 h after activation. The data represent technical duplicates from two independently treated wells. The figure shows representative data out of six replicates.

## Conclusions

We have prepared a large library of 63 potent inhibitors of human CD73 that are based on novel and previously unexplored 7-deazapurine ribonucleoside 5-*O*′-bisphosphonates bearing (het)­aryl group directly attached via C–C bond to position 6. The key steps in their synthetic strategy are Suzuki-Miyaura coupling and bisphosphonate attachment to the 5′–OH group on the sugar moiety, which allowed synthesis of several series of compounds with various (het)­aryl groups in position 6 and small substituents like amino, methyl, fluoro, chloro, and iodo in position 2. Screening of their potential to inhibit human CD73 showed that while amino, methyl, and fluoro substituents in position 2 have mixed effects in each series, introduction of chlorine into position 2 led to significant improvement of inhibition activity in all cases. We showed that in this series, 7-deazapurine nucleobase gives significantly more potent inhibitors than purine (by 3 orders of magnitude). In general, compounds bearing bicyclic (het)­aryl substituents are the most potent, with *K*_*i*_ values in the picomolar range. Introduction of another halogen atom into the naphthyl substituent gave the most active compounds in this work (compound **7B.28** with *K*_*i*_ = 0.8 pM). Interestingly, the compounds also inhibited the murine CD73 but with significantly (1–2 orders of magnitude) higher *K*_*i*_ values, mostly in the nanomolar range. This is an important finding for future considerations of *in vivo* testing in mice.

Screening of biological activities and ADME properties showed that our compounds are highly specific for CD73 (not inhibiting other ecto-nucleotidases CD39 and NTPDase 3), they decrease adenosine production in human breast cancer cell line, are not toxic to both cancer and nonmalignant cells, and are able to restore T cell activation. Moreover, all the compounds are nicely soluble, very stable in plasma and microsomes, and selected derivatives (like **18E.6**) have favorable pharmacokinetic properties *in vivo*, at least comparable with clinical candidate **AB680**. Taken together, this study identified several candidates based on a novel 6-(het)­aryl-7-deazapurine scaffold that are highly potent human CD73 inhibitors with favorable ADME properties, suitable for preclinical development as potential cancer immunotherapeutics, especially in combination therapies with other/already approved drugs. These findings establish a strong foundation for ongoing and future follow-up *in vivo* efficacy and combination studies, which will further explore the therapeutic potential of this novel inhibitor class.

## Experimental Section

### General Remarks - Synthesis

All solvents and reagents were purchased from commercial suppliers and used as received. All the starting 7-deazapurine nucleosides (**1,** ^20^ **5,** ^21^ **8,** ^21^ **12,** ^21^ **15,** ^19^ **23** ^23^) were synthesized according to literature procedures. Starting 6-chloropurine ribonucleoside **28** was purchased from a commercial supplier. Reactions were monitored by thin layer chromatography (TLC) on *Merck silica gel 60 F-254 aluminum sheets* and detected by UV (254 nm) and by *Advion Expression* Compact Mass Spectrometer connected with *Plate Express* TLC Plate Reader using electrospray ionization (ESI). NMR spectra were measured on *Bruker Avance III* 400 MHz spectrometer (400.1 MHz for ^1^H and 100.6 MHz for ^13^C) or *Bruker Avance III* 500 MHz spectrometer (500.0 MHz for ^1^H, 125.7 MHz for ^13^C and ^31^P at 202.4 MHz) or *Bruker Avance III* 600 MHz spectrometer (600.1 MHz for ^1^H and 150.9 MHz for ^13^C) in DMSO-*d*_*6*_ (referenced to the residual solvent signal, [δ (^1^H) = 2.50 ppm, δ (^13^C) = 39.52 ppm]), in D_2_O (*t*BuOH used as internal standard, [δ (^1^H) = 1.25 ppm, δ (^13^C) = 31.60 ppm]) or in CDCl_3_ (referenced to the residual solvent signal, [δ (^1^H) = 7.26 ppm, δ (^13^C) = 77.16 ppm]). Chemical shifts are given in ppm (δ-scale), coupling constants (*J*) in Hz. Complete assignments of all NMR signals were performed using a combination of H,H–COSY, H,H-ROESY, H,C-HSQC and H,C-HMBC experiments. Low-resolution mass spectra were measured on *LCQ Fleet* (Thermo Fisher Scientific) using electrospray ionization (ESI). High-resolution mass spectra were measured on *LTQ Orbitrap XL* (Thermo Fisher Scientific). All mass spectra were acquired by the MS service at IOCB. High-performance flash chromatography (HPFC) was performed with ISCO *Combi Flash R*_*f*_ system on *Redi Sep R*_*f*_ *Gold* Silica Gel Disposable columns. If needed, purification of final free phosphonates was performed using HPLC (Waters modular HPLC system) on a column packed with 5 μm C18 reversed phase (*Kinetex EVO*, *C18 100 Å*). Purity of all final compounds (>95%) was determined by analytical UPLC-MS and by clean NMR spectra. UPLC-MS analysis was performed on an *Agilent 1260 Infinity II LC* system with an *Agilent 1260 Photodiode Array Detector*, Columns: *Acquity Premier CSH C18 1.7* μ*m VanGuard FIT* (2.1 × 150 mm), *Kinetex EVO C18 100 Å 1.7* μ*m* (2.1 × 150 mm), Flow: 0.2–0.25 mL/min.

### Synthesis of the Target Bisphosphonates

#### General Procedure A (GP A): Suzuki Cross-Coupling Reaction on Ribonucleoside Derivatives

H_2_O/MeCN (2:1, 3 mL/100 mg) was added through a septum to an argon purged vial containing protected or unprotected nucleoside intermediate (1 equiv), corresponding boronic acid (1.1–5 equiv), Na_2_CO_3_ (3 equiv), TPPTS (0.12 equiv) and Pd­(OAc)_2_ (0.05 equiv). The mixture was heated at 80–140 °C from 10 min to 18 h. Solvent was evaporated, and the crude mixture was purified by HPFC.

#### General Procedure B (GP B): Bisphosphonation

Nucleoside was dissolved in dry trimethylphosphate (0.5 mL/50–150 mg of nucleoside) and cooled to 0 °C. A cold solution of methylene bis­(phosphonic dichloride) (3–5 equiv) in trimethylphosphate (0.5–1 mL) was added dropwise and the mixture was stirred at 0 °C for 3 h. The mixture was treated with water or 2 M TEAB solution and purified by RP-HPFC, HPLC or ion exchange chromatography. The final products were lyophilized from H_2_O/*t*-BuOH.

#### General Procedure C (GP C): Suzuki Cross-Coupling Reaction on 2′-Fluoroarabinonucleosides Followed by Deprotection

Degassed H_2_O/THF (1:4, 1.3 mL/100 mg) was added through a septum to an argon purged vial containing protected nucleoside intermediate (1 equiv), corresponding boronic acid (1.4 equiv), K_2_CO_3_ (2 equiv) and Pd­(PPh_3_)_4_ (0.05 equiv). The mixture was heated at 63 °C for 24 h. After completion of the reaction, the mixture was partitioned between DCM/water and extracted 3 times. Combined organic layers were dried over Na_2_SO_4_, filtered, evaporated, and purified by HPFC (SiO_2_, cHex/EtOAc 1:0 → 4:1). Obtained nucleoside was dissolved in a mixture of MeOH/DCM (4:1, 2 mL/100 mg) and treated with K_2_CO_3_ (3 equiv). The mixture was stirred at rt for 1 h. After completion of the reaction, the solvent was evaporated, and the mixture was purified by HPFC. The final product was lyophilized from an acetonitrile/water mixture.

#### 4-Chloro-7-(2,3-*O*-isopropylidene-β-d-ribofuranosyl)-7*H*-pyrrolo­[2,3-*d*]­pyrimidine (2)

Nucleoside **1** ^20^ (1.04 g, 2.36 mmol) was treated with TBAF (3.8 mL, 1 M in THF, 3.8 mmol) and stirred at rt for 30 min. HPFC (SiO_2_, cHex/EtOAc 5.7:1 → 1:1) gave compound **2** (653.6 mg, 85%) as a white amorphous solid. ^1^H NMR (500 MHz, CDCl_3_): 1.37 and 1.64 (2 × s, 2 × 3H, (CH_3_)_2_C); 3.81 (dd, 1H, *J*_*gem*_ = 12.6 Hz, *J*_*5′a,4′*_ = 2.1 Hz, H-5′a); 3.96 (dd, 1H, *J*_*gem*_ = 12.6 Hz, *J*_*5′b,4′*_ = 1.9 Hz, H-5′b); 4.49 (q, 1H, *J*_*4′,5′a*_ = *J*_*4′,5′b*_ = *J*_*4′,3′*_ = 1.9 Hz, H-4′); 5.11 (dd, 1H, *J*_*3′,2′*_ = 6.1 Hz, *J*_*3′,4′*_ = 1.8 Hz, H-3′); 5.23 (dd, 1H, *J*_*2′,3′*_ = 6.1 Hz, *J*_*2′,1′*_ = 4.8 Hz, H-2′); 5.87 (d, 1H, *J*_*1′,2′*_ = 4.8 Hz, H-1′); 6.63 (d, 1H, *J*_*5,6*_ = 3.7 Hz, H-5); 7.33 (d, 1H, *J*_*6,5*_ = 3.7 Hz, H-6); 8.63 (s, 1H, H-2); ^13^C NMR (125.7 MHz, CDCl_3_): 25.4 and 27.7 ((**C**H_3_)_2_C); 63.44 (CH_2_–5′); 81.5 (CH-3′); 83.15 (CH-2′); 85.7 (CH-4′); 95.8 (CH-1′); 100.3 (C-5); 114.4 ((CH_3_)_2_ **C**); 120.1 (C-4a); 129.8 (CH-6); 149.7 (C-7a); 150.49 (CH-2); 153.4 (C-4). HR-ESI-MS: *found*: 326.0905 ([M + H]^+^, calcd for C_14_H_17_O_4_N_3_Cl^+^: 326.0902); HR-ESI-MS: *found*: 348.0724 ([M + Na]^+^, calcd for C_14_H_16_O_4_N_3_ClNa^+^: 348.0722).

#### [(5-{[4-Chloro-7*H*-pyrrolo­[2,3-*d*]­pyrimidin-7-yl]-β-d-ribofuranosyl}­oxy)­phosphonomethyl]­phosphonic Acid (3)

GP B using nucleoside **2** (643 mg, 1.97 mmol). RP-HPFC (C-18, H_2_O/MeOH 0 → 100%) gave product **3** (476 mg, 54%) as a white powder. ^1^H NMR (500 MHz, DMSO-d_6_): 2.25 (t, 2H, *J*_*CH2,P*_ = 20.4 Hz, PCH_2_P); 4.07–4.15 (m, 3H, H-5′,4′); 4.20 (dd, 1H, *J*_*3′,2′*_ = 5.1 Hz, *J*_*3′,4′*_ = 2.9 Hz, H-3′); 4.46 (dd, 1H, *J*_*2′,1′*_ = 6.2 Hz, *J*_*2′,3′*_ = 5.2 Hz, H-2′); 6.24 (d, 1H, *J*_*1′,2′*_ = 6.2 Hz, H-1′); 6.74 (d, 1H, *J*_*5,6*_ = 3.8 Hz, H-5); 8.00 (d, 1H, *J*_*6,5*_ = 3.8 Hz, H-6); 8.68 (s, 1H, H-2); ^13^C NMR (125.7 MHz, DMSO-d_6_): 27.5 (t, *J*_*C,P*_ = 128.8 Hz, PCH_2_P); 64.7 (d, *J*_*C,P*_ = 5.3 Hz, CH_2_–5′); 70.3 (CH-3′); 73.9 (CH-2′); 83.1 (d, *J*_*C,P*_ = 7.4 Hz, CH-4′); 86.9 (CH-1′); 100.0 (CH-5); 117.3 (C-4a); 128.6 (CH-6); 150.7 (CH-2); 150.8 (C-4); 151.4 (C-7a); ^31^P NMR (202.4 MHz, DMSO-d_6_): 15.76 and 19.83 (2 × d, 2 × 1P, *J*_*P,P*_ = 6.7 Hz, PCH_2_P). HR-ESI-MS: *found*: 441.9973 ([M–H]^−^, calcd for C_12_H_15_O_9_N_3_ClP_2_^–^: 441.9978).

#### [(5-{[4-(Furan-2-yl)-7*H*-pyrrolo­[2,3-*d*]­pyrimidin-7-yl]-β-d-ribofuranosyl}­oxy)­phosphonomethyl]­phosphonic Acid (4A.1)

Compound **3** (51.6 mg, 0.12 mmol) was reacted with furan-2-ylboronic acid (19.5 mg, 0.17 mmol) for 5 min at 80 °C and 1 h at 65 °C according to the GP A. RP-HPFC (C-18, H_2_O/MeOH 0 → 100%), HPLC (C-18, H_2_O + 0.05% TFA/MeCN 0 → 80%) gave product **4A.1** (30.8 mg, 56%) as a pale-yellow powder. ^1^H NMR (500 MHz, DMSO-d_6_): 2.26 (t, 2H, *J*_*CH2,P*_ = 19.9 Hz, PCH_2_P); 4.06–4.17 (m, 3H, H-4′,5′); 4.21 (m, 1H, H-3′); 4.47 (t, 1H, *J*_*2′,1′*_ = *J*_*2′,3′*_ = 5.6 Hz, H-2′); 6.29 (d, 1H, *J*_*1′,2′*_ = 6.2 Hz, H-1′); 6.79 (dd, 1H, *J*_*4,3*_ = 3.5 Hz, *J*_*4,5*_ = 1.7 Hz, H-4-furyl); 7.07 (d, 1H, *J*_*5,6*_ = 3.7 Hz, H-5); 7.48 (dd, 1H, *J*_*3,4*_ = 3.5 Hz, *J*_*3,5*_ = 0.7 Hz, H-3-furyl); 7.94 (d, 1H, *J*_*6,5*_ = 3.7 Hz, H-6); 8.07 (d, 1H, *J*_*5,4*_ = 1.7 Hz, *J*_*5,3*_ = 0.7 Hz, H-5-furyl); 8.78 (s, 1H, H-2); ^13^C NMR (125.7 MHz, DMSO-d_6_): 27.5 (bt, *J*_*C,P*_ = 129.4 Hz, PCH_2_P); 64.7 (bs, CH_2_–5′); 70.4 (CH-3′); 73.7 (CH-2′); 82.9 (d, *J*_*C,P*_ = 5.8 Hz, CH-4′); 86.3 (CH-1′); 101.5 (CH-5); 112.5 (C-4a); 112.7 (CH-4-furyl); 113.3 (CH-3-furyl); 127.9 (CH-6); 146.31 (C-4); 146.4 (CH-5-furyl); 151.1 (CH-2); 152.30 and 152.3 (C-7a, C-2-furyl); ^31^P NMR (202.4 MHz, DMSO-d_6_): 15.83 and 19.68 (2 × bs, 2 × 1P, PCH_2_P). HR-ESI-MS: *found*: 474.0467 ([M–H]^−^, calcd for C_16_H_18_O_10_N_3_P_2_^–^: 474.0462); HR-ESI-MS: *found*: 496.0285 ([M–2H + Na]^−^, calcd for C_16_H_17_O_10_N_3_NaP_2_^–^: 496.0281).

#### 2-Chloro-4-(furan-2-yl)-7-(β-d-ribofuranosyl)-7*H*-pyrrolo­[2,3-*d*]­pyrimidine (6B.1)

Nucleoside **5** ^21^ (134 mg, 0.42 mmol) was reacted with furan-2-ylboronic acid (51.5 mg, 0.46 mmol) for 10 min at 100 °C according to the GP A. HPFC (SiO_2_, DCM/MeOH 1:0 → 9:1) gave **6B.1** (123 mg, 84%) as a white solid. ^1^H NMR spectrum was in agreement with the literature.^21^

#### [(5-{[2-Chloro-4-(furan-2-yl)-7*H*-pyrrolo­[2,3-*d*]­pyrimidin-7-yl]-β-d-ribofuranosyl}­oxy)­phosphonomethyl]­phosphonic Acid (7B.1)

GP B using compound **6B.1** (42.3 mg, 0.12 mmol). HPLC (C-18, H_2_O + 0.05% TFA/MeCN 0 → 80%) gave product **7B.1** (24.6 mg, 40%) as a brownish powder. ^1^H NMR (500 MHz, DMSO-d_6_): 2.26 (t, 2H, *J*_*CH2,P*_ = 20.4 Hz, PCH_2_P); 4.08–4.14 (m, 3H, H-4′,5′); 4.19 (dd, 1H, *J*_*3′,2′*_ = 5.1 Hz, *J*_*3′,4′*_ = 2.6 Hz, H-3′); 4.44 (dd, 1H, *J*_*2′,1′*_ = 6.4 Hz, *J*_*2′,3′*_ = 5.1 Hz, H-2′); 6.17 (d, 1H, *J*_*1′,2′*_ = 6.4 Hz, H-1′); 6.82 (dd, 1H, *J*_*4,3*_ = 3.6 Hz, *J*_*4,5*_ = 1.8 Hz, H-4-furyl); 7.10 (d, 1H, *J*_*5,6*_ = 3.7 Hz, H-5); 7.54 (dd, 1H, *J*_*3,4*_ = 3.6 Hz, *J*_*3,5*_ = 0.8 Hz, H-3-furyl); 7.97 (d, 1H, *J*_*6,5*_ = 3.8 Hz, H-6); 8.11 (d, 1H, *J*_*5,4*_ = 1.8 Hz, *J*_*5,3*_ = 0.8 Hz, H-5-furyl); ^13^C NMR (125.7 MHz, DMSO-d_6_): 27.5 (t, *J*_*C,P*_ = 128.7 Hz, PCH_2_P); 64.7 (d, *J*_*C,P*_ = 5.4 Hz, CH_2_–5′); 70.4 (CH-3′); 73.8 (CH-2′); 83.2 (d, *J*_*C,P*_ = 7.4 Hz, CH-4′); 86.2 (CH-1′); 102.0 (CH-5); 111.6 (C-4a); 113.1 (CH-4-furyl); 115.0 (CH-3-furyl); 128.6 (CH-6); 147.3 (CH-5-furyl); 148.0 (C-4a); 151.1 (C-2-furyl); 152.4 (C-2); 153.7 (C-7a); ^31^P NMR (202.4 MHz, DMSO-d_6_): 14.63 and 1.60 (2 × bd, 2 × 1P, *J*_*P,P*_ = 5.7 Hz, PCH_2_P). HR-ESI-MS: *found*: 508.0081 ([M–H]^−^, calcd for C_16_H_17_O_10_N_3_ClP_2_^–^: 508.0083).

#### [(5-{[2-Amino-4-chloro-7*H*-pyrrolo­[2,3-*d*]­pyrimidin-7-yl]-β-d-ribofuranosyl}­oxy)­phosphonomethyl]­phosphonic Acid (9)

GP B using compound **8** ^21^ (335.3 mg, 1.12 mmol). RP-HPFC (C-18, H_2_O/MeOH 0 → 100%) gave product **9** (204.3 mg, 40%) as a white powder. ^1^H NMR (500 MHz, DMSO-d_6_): 2.21 (t, 2H, *J*_*CH2,P*_ = 20.5 Hz, PCH_2_P); 4.01 (m, 1H, H-4′); 4.06 (ddd, 1H, *J*_*gem*_ = 15.5 Hz, *J*_*5′a,P*_ = 6.7 Hz, *J*_*5′a,4′*_ = 4.4 Hz, H-5′a); 4.12 (ddd, 1H, *J*_*gem*_ = 15.5 Hz, *J*_*5′b,P*_ = 6.3 Hz, *J*_*5′b,4′*_ = 4.2 Hz, H-5′b); 4.14 (dd, 1H, *J*_*3′,2′*_ = 5.1 Hz, *J*_*3′,4′*_ = 3.1 Hz, H-3′); 4.38 (dd, 1H, *J*_*2′,1′*_ = 6.5 Hz, *J*_*2′,3′*_ = 5.1 Hz, H-2′); 4.40–5.60 (m, 5H, OH-2′,3′,5′, NH_2_); 5.99 (d, 1H, *J*_*1′,2′*_ = 6.5 Hz, H-1′); 6.36 (d, 1H, *J*_*5,6*_ = 3.8 Hz, H-5); 7.37 (d, 1H, *J*_*6,5*_ = 3.9 Hz, H-6); ^13^C NMR (125.7 MHz, DMSO-d_6_): 27.6 (t, *J*_*C,P*_ = 129.0 Hz, PCH_2_P); 64.8 (d, *J*_*C,P*_ = 5.5 Hz, CH_2_–5′); 70.4 (CH-3′); 73.0 (CH-2′); 82.6 (d, *J*_*C,P*_ = 7.5 Hz, CH-4′); 86.1 (CH-1′); 99.9 (CH-5); 108.9 (C-4a); 123.5 (CH-6); 151.2 (C-4); 154.5 (C-7a); 159.4 (C-2); ^31^P NMR (202.4 MHz, DMSO-d_6_): 15.71 and 19.78 (2 × d, 2 × 1P, *J*_*P,P*_ = 8.1 Hz, PCH_2_P). HR-ESI-MS: *found*: 457.0083 ([M–H]^−,^ calcd for C_12_H_16_O_9_N_4_P_2_^–^: 457.0087).

#### 2-Amino-4-(naphth-1-yl)-7-(β-d-ribofuranosyl)-7*H*-pyrrolo­[2,3-*d*]­pyrimidine (10C.6)

Nucleoside **8** (81.9 mg, 0.27 mmol) was reacted with naphthalene-1-boronic acid (70.3 mg, 0.41 mmol) for 1 h at 100 °C according to the GP A. HPFC (SiO_2_, DCM/MeOH 1:0 → 9:1) gave **10C.6** (94.7 mg, 89%) as a white powder. ^1^H NMR (500 MHz, DMSO-d_6_): 3.53 (ddd, 1H, *J*_*gem*_ = 11.8 Hz, *J*_*5′a,OH*_ = 5.5 Hz, *J*_*5′a,4′*_ = 4.1 Hz, H-5′a); 3.60 (ddd, 1H, *J*_*gem*_ = 11.8 Hz, *J*_*5′b,OH*_ = 5.4 Hz, *J*_*5′b,4′*_ = 4.2 Hz, H-5′b); 3.88 (td, 1H, *J*_*4′,5′a*_ = *J*_*4′,5′b*_ = 4.1 Hz, *J*_*4′,3′*_ = 3.1 Hz, H-4′); 4.08 (td, 1H, *J*_*3′,2′*_ = *J*_*3′,OH*_ = 4.8 Hz, *J*_*3′,4′*_ = 3.1 Hz, H-3′); 4.39 (td, 1H, *J*_*2′,1′*_ = *J*_*2′,OH*_ = 6.4 Hz, *J*_*2′,3′*_ = 5.1 Hz, H-2′); 5.00 (t, 1H, *J*_*OH,5′a*_ = *J*_*OH,5′b*_ = 5.5 Hz, OH-5′); 5.11 (d, 1H, *J*_*OH,3′*_ = 4.6 Hz, OH-3′); 5.31 (d, 1H, *J*_*OH,2′*_ = 6.3 Hz, OH-2′); 6.04 (d, 1H, *J*_*5,6*_ = 3.8 Hz, H-5); 6.13 (d, 1H, *J*_*1′,2′*_ = 6.5 Hz, H-1′); 6.41 (s, 2H, NH_2_); 7.32 (d, 1H, *J*_*6,5*_ = 3.8 Hz, H-6); 7.49 (ddd, 1H, *J*_*7,8*_ = 8.3 Hz, *J*_*7,6*_ = 6.8 Hz, *J*_*7,5*_ = 1.5 Hz, H-7-naphthyl); 7.56 (ddd, 1H, *J*_*6,5*_ = 8.1 Hz, *J*_*6,7*_ = 6.8 Hz, *J*_*6,8*_ = 1.3 Hz, H-6-naphthyl); 7.62–7.68 (m, 2H, H-2,3-naphthyl); 8.02 (dm, 1H, *J*_*8,7*_ = 8.1 Hz, H-5-naphthyl); 8.04 (bd, 1H, *J*_*4,5*_ = 8.3 Hz, H-8-naphthyl); 8.05 (bd, 1H, *J*_*4,3*_ = 7.4 Hz, H-4-naphthyl); ^13^C NMR (125.7 MHz, DMSO-d_6_): 61.8 (CH_2_–5′); 70.7 (CH-3′); 73.4 (CH-2′); 84.8 (CH-4′); 85.7 (CH-1′); 100.9 (CH-5); 110.6 (C-4a); 122.9 (CH-6); 125.3 (CH-3-naphthyl); 125.7 (CH-8-naphthyl); 126.1 (CH-6-naphthyl); 126.3 (CH-7-naphthyl); 127.2 (CH-2-naphthyl); 128.3 (CH-5-naphthyl); 129.1 (CH-4-naphthyl); 130.3 (C-8a-naphthyl); 133.4 (C-4a-naphthyl); 135.4 (C-1-naphthyl); 154.4 (C-7a); 159.1 (C-4); 159.9 (C-2). HR-ESI-MS: *found*: 393.1556 ([M + H]^+^, calcd for C_21_H_21_O_4_N_4_^+^: 393.1557); HR-ESI-MS: *found*: 415.1375 ([M + Na]^+^, calcd for C_21_H_20_O_4_N_4_Na^+^: 415.1377).

#### [(5-{[2-Amino-4-(furan-2-yl)-7*H*-pyrrolo­[2,3-*d*]­pyrimidin-7-yl]-β-d-ribofuranosyl}­oxy)­phosphonomethyl]­phosphonic Acid (11C.1)

Compound **9** (53.6 mg, 0.12 mmol) was reacted with furan-2-yl-boronic acid (26.2 mg, 0.23 mmol) for 1 h at 65 °C according to the GP A. HPLC purification (C-18, H_2_O + 0.05% TFA/MeCN 0 → 80%) gave product **11C.1** (5.7 mg, 10%) as a yellow powder. ^1^H NMR (500 MHz, DMSO-d_6_): 2.23 (t, 2H, *J*_*CH2,P*_ = 20.3 Hz, PCH_2_P); 4.01 (bq, 1H, *J*_*4′,3′*_ = *J*_*4′,5′a*_ = *J*_*4′,5′b*_ = 3.9 Hz, H-4′); 4.05 (dm, 1H, *J*_*gem*_ = 11.1 Hz, H-5′a); 4.12 (dm, 1H, *J*_*gem*_ = 11.1 Hz, H-5′b); 4.16 (dd, 1H, *J*_*3′,2′*_ = 5.1 Hz, *J*_*3′,4′*_ = 3.0 Hz, H-3′); 4.41 (dd, 1H, *J*_*2′,1′*_ = 6.4 Hz, *J*_*2′,3′*_ = 5.1 Hz, H-2′); 6.07 (d, 1H, *J*_*1′,2′*_ = 6.4 Hz, H-1′); 6.76 (dd, 1H, *J*_*4,3*_ = 3.6 Hz, *J*_*4,5*_ = 1.7 Hz, H-4-furyl); 6.81 (d, 1H, *J*_*5,6*_ = 3.8 Hz, H-5); 7.35 (m, 1H, H-3-furyl); 7.44 (d, 1H, *J*_*6,5*_ = 3.8 Hz, H-6); 8.02 (bd, 1H, *J*_*5,4*_ = 1.7 Hz, H-5-furyl); ^13^C NMR (125.7 MHz, DMSO-d_6_): 27.5 (t, *J*_*C,P*_ = 127.8 Hz, PCH_2_P); 64.7 (d, *J*_*C,P*_ = 5.3 Hz, CH_2_–5′); 70.5 (CH-3′); 73.0 (CH-2′); 82.5 (d, *J*_*C,P*_ = 7.2 Hz, CH-4′); 85.7 (CH-1′); 101.8 (CH-5); 105.7 (C-4a); 112.6 (CH-4-furyl); 113.2 (CH-3-furyl); 124.3 (CH-6); 145.7 (CH-5-furyl); 151.1 (C-2-furyl); 155.3 (C-7a). *Carbon signals C-2,4 were not detected.* ^31^P NMR (202.4 MHz, DMSO-d_6_): 15.96 and 19.40 (2 × bs, 2 × 1P, PCH_2_P). HR-ESI-MS: *found*: 489.0579 ([M–H]^−^, calcd for C_16_H_19_O_10_N_4_P_2_^–^: 489.0582).

#### 2-Fluoro-4-(furan-2-yl)-7-(β-d-ribofuranosyl)-7*H*-pyrrolo­[2,3-*d*]­pyrimidine (13D.1)

Compound **12** ^21^ (87.7 mg, 0.29 mmol) was reacted with furan-2-ylboronic acid (48.5 mg, 0.43 mmol) for 1 h at 100 °C according to the GP A. HPFC purification (SiO_2_, DCM/MeOH 1:0 → 9:1) gave **13D.1** (50.8 mg, 52%) as a pale-yellow powder. ^1^H NMR spectrum was in agreement with the literature.^21^

#### [(5-{[2-Fluoro-4-(furan-2-yl)-7*H*-pyrrolo­[2,3-*d*]­pyrimidin-7-yl]-β-d-ribofuranosyl}­oxy)­phosphonomethyl]­phosphonic Acid (14D.1)

GP B using compound **13D.1** (43.4 mg, 0.13 mmol). HPLC purification (C-18, H_2_O + 0.05% TFA/MeCN 0 → 80%) gave **14D.1** (27.2 mg, 43%) as a yellow powder. ^1^H NMR (500 MHz, DMSO-d_6_): 2.25 (t, 2H, *J*_*CH2,P*_ = 20.4 Hz, PCH_2_P); 4.07–4.14 (m, 3H, H-4′,5′); 4.19 (dd, 1H, *J*_*3′,2′*_ = 5.1 Hz, *J*_*3′,4′*_ = 2.7 Hz, H-3′); 4.43 (dd, 1H, *J*_*2′,1′*_ = 6.3 Hz, *J*_*2′,3′*_ = 5.1 Hz, H-2′); 6.13 (d, 1H, *J*_*1′,2′*_ = 6.3 Hz, H-1′); 6.83 (dd, 1H, *J*_*4,3*_ = 3.6 Hz, *J*_*4,5*_ = 1.7 Hz, H-4-furyl); 7.10 (d, 1H, *J*_*5,6*_ = 3.8 Hz, H-5); 7.55 (bd, 1H, *J*_*3,4*_ = 3.6 Hz, H-3-furyl); 7.92 (d, 1H, *J*_*6,5*_ = 3.8 Hz, H-6); 8.12 (bd, 1H, *J*_*5,4*_ = 1.7 Hz, H-5-furyl); ^13^C NMR (125.7 MHz, DMSO-d_6_): 27.5 (t, *J*_*C,P*_ = 128.0 Hz, PCH_2_P); 64.6 (d, *J*_*C,P*_ = 5.4 Hz, CH_2_–5′); 70.4 (CH-3′); 73.74 (CH-2′); 83.1 (d, *J*_*C,P*_ = 7.3 Hz, CH-4′); 86.4 (CH-1′); 102.2 (CH-5); 111.3 (d, *J*_*C,F*_ = 3.4 Hz, C-4a); 113.0 (CH-4-furyl); 115.1 (CH-3-furyl); 128.3 (d, *J*_*C,F*_ = 3.5 Hz, CH-6); 147.3 (CH-5-furyl); 148.3 (d, *J*_*C,F*_ = 15.9 Hz, C-4); 151.2 (C-2-furyl); 154.4 (d, *J*_*C,F*_ = 16.3 Hz, C-7a); 158.4 (d, *J*_*C,F*_ = 205.5 Hz, C-2); ^31^P NMR (202.4 MHz, DMSO-d_6_): 15.92 and 19.49 (2 × bd, 2 × 1P, *J*_*P,P*_ = 5.1 Hz, PCH_2_P); ^19^F NMR (470.4 MHz, DMSO-d_6_): – 53.70 (s, 1F, F-2). HR-ESI-MS: *found*: 492.0377 ([M–H]^−^, calcd for C_16_H_17_O_10_N_3_FP_2_^–^: 492.0379).

#### [(5-{[4-Chloro-2-methyl-7*H*-pyrrolo­[2,3-*d*]­pyrimidin-7-yl]-β-d-ribofuranosyl}­oxy)­phosphonomethyl]­phosphonic Acid (16)

GP B using compound **15** ^21^ (331.4 mg, 1.11 mmol). RP-HPFC (C-18, H_2_O/MeOH 0 → 100%) gave product **16** (148.6 mg, 29%) as a white powder. ^1^H NMR (500 MHz, DMSO-d_6_): 2.25 (t, 2H, *J*_*CH2,P*_ = 20.5 Hz, PCH_2_P); 2.66 (s, 3H, CH_3_-2); 4.06–4.16 (m, 3H, H-4′,5′); 4.19 (dd, 1H, *J*_*3′,2′*_ = 5.1 Hz, *J*_*3′,4′*_ = 2.8 Hz, H-3′); 4.46 (dd, 1H, *J*_*2′,1′*_ = 6.5 Hz, *J*_*2′,3′*_ = 5.1 Hz, H-2′); 6.21 (d, 1H, *J*_*1′,2′*_ = 6.5 Hz, H-1′); 6.66 (d, 1H, *J*_*5,6*_ = 3.8 Hz, H-5); 7.87 (d, 1H, *J*_*6,5*_ = 3.8 Hz, H-6); ^13^C NMR (125.7 MHz, DMSO-d_6_): 25.2 (CH_3_-2); 27.5 (t, *J*_*C,P*_ = 129.0 Hz, PCH_2_P); 64.7 (d, *J*_*C,P*_ = 5.4 Hz, CH_2_–5′); 70.4 (CH-3′); 73.6 (CH-2′); 83.1 (d, *J*_*C,P*_ = 7.5 Hz, CH-4′); 86.5 (CH-1′); 99.8 (CH-5); 114.8 (C-4a); 127.7 (CH-6); 150.5 (C-4); 152.3 (C-7a); 160.2 (C-2); ^31^P NMR (202.4 MHz, DMSO-d_6_): 15.69 and 19.83 (2 × d, 2 × 1P, *J*_*P,P*_ = 8.2 Hz, PCH_2_P). HR-ESI-MS: *found*: 456.0130 ([M–H]^−^, calcd for C_13_H_17_O_9_N_3_ClP_2_^–^: 456.0134).

#### 4-(Furan-2-yl)-2-methyl-7-(β-d-ribofuranosyl)-7*H*-pyrrolo­[2,3-*d*]­pyrimidine (17E.1)

Compound **15** (89.2 mg, 0.30 mmol) was reacted with furan-2-ylboronic acid (50.0 mg, 0.45 mmol) for 1 h at 100 °C according to the GP A. HPFC (SiO_2_, DCM/MeOH 1:0 → 9:1) gave **17E.1** (77.7 mg, 79%) as a pale-yellow powder. ^1^H NMR spectrum was in agreement with the literature.^21^

#### [(5-{[4-(Furan-2-yl)-2-methyl)-7*H*-pyrrolo­[2,3-*d*]­pyrimidin-7-yl]-β-d-ribofuranosyl}­oxy)­phosphonomethyl]­phosphonic Acid (18E.1)

GP B using compound **17E.1** (69.4 mg, 0.21 mmol). Two HPLC purifications (C-18, H_2_O + 0.05% TFA/MeCN 0 → 80%) gave **18E.1** (34.6 mg, 34%) as a pale-yellow powder. ^1^H NMR (500 MHz, DMSO-d_6_): 2.26 (t, 2H, *J*_*CH2,P*_ = 20.5 Hz, PCH_2_P); 2.69 (s, 3H, CH_3_-2); 4.05–4.17 (m, 3H, H-4′,5′); 4.20 (dd, 1H, *J*_*3′,2′*_ = 5.1 Hz, *J*_*3′,4′*_ = 2.9 Hz, H-3′); 4.47 (dd, 1H, *J*_*2′,1′*_ = 6.4 Hz, *J*_*2′,3′*_ = 5.1 Hz, H-2′); 6.26 (d, 1H, *J*_*1′,2′*_ = 6.4 Hz, H-1′); 6.79 (dd, 1H, *J*_*4,3*_ = 3.5 Hz, *J*_*4,5*_ = 1.7 Hz, H-4-furyl); 7.03 (d, 1H, *J*_*5,6*_ = 3.8 Hz, H-5); 7.49 (d, 1H, *J*_*3,4*_ = 3.5 Hz, H-3-furyl); 7.85 (d, 1H, *J*_*6,5*_ = 3.8 Hz, H-6); 8.07 (bd, 1H, *J*_*5,4*_ = 1.3 Hz, H-5-furyl); ^13^C NMR (125.7 MHz, DMSO-d_6_): 25.4 (CH_3_-2); 27.6 (t, *J*_*C,P*_ = 128.9 Hz, PCH_2_P); 64.8 (d, *J*_*C,P*_ = 5.6 Hz, CH_2_–5′); 70.5 (CH-3′); 73.5 (CH-2′); 82.9 (d, *J*_*C,P*_ = 7.5 Hz, CH-4′); 85.9 (CH-1′); 101.6 (CH-5); 110.3 (C-4a); 112.8 (CH-4-furyl); 113.7 (CH-3-furyl); 127.4 (CH-6); 145.7 (C-4); 146.4 (CH-5-furyl); 151.8 (C-2-furyl); 153.2 (C-7a); 159.5 (C-2); ^31^P NMR (202.4 MHz, DMSO-d_6_): 15.70 and 19.83 (2 × d, 2 × 1P, *J*_*P,P*_ = 8.1 Hz, PCH_2_P). HR-ESI-MS: *found*: 488.0628 ([M–H]^−^, calcd for C_17_H_20_O_10_N_3_P_2_^–^: 488.0629).

#### 2-Methoxy-4-(naphthalen-2-yl)-7-(β-d-ribofuranosyl)-7*H*-pyrrolo­[2,3-*d*]­pyrimidine (19F.7)

Compound **6B.7** (40.4 mg, 0.098 mmol) was treated with 3 mL 0.5 M NaOMe (in methanol) and stirred overnight at 60 °C. HPFC (SiO_2_, DCM/MeOH 1:0 → 5.7:1) and lyophilization from H_2_O/*t*-BuOH gave **19F.7** (34.9 mg, 87%) as a white powder. ^1^H NMR (500 MHz, DMSO-d_6_): 3.57 (bdt, 1H, *J*_*gem*_ = 11.8 Hz, *J*_*5′a,OH*_ = *J*_*5′a,4′*_ = 4.5 Hz, H-5′a); 3.65 (bdt, 1H, *J*_*gem*_ = 11.8 Hz, *J*_*5′b,OH*_ = *J*_*5′b,4′*_ = 4.5 Hz, H-5′b); 3.93 (q, 1H, *J*_*4′,5′a*_ = *J*_*4′,5′b*_ = *J*_*4′,3′*_ = 3.9 Hz, H-4′); 4.05 (s, 3H, CH_3_O); 4.15 (bt, 1H, *J*_*3′,2′*_ = *J*_*3′,4′*_ = 4.3 Hz, H-3′); 4.48 (bt, 1H, *J*_*2′,1′*_ = *J*_*2′,3′*_ = 5.7 Hz, H-2′); 5.07 (bt, 1H, *J*_*OH,5′a*_ = *J*_*OH,5′b*_ = 5.5 Hz, OH-5′); 5.31 (bs, 1H, OH-3′); 5.48 (bs, 1H, OH-2′); 6.18 (d, 1H, *J*_*1′,2′*_ = 6.1 Hz, H-1′); 7.08 (d, 1H, *J*_*5,6*_ = 3.9 Hz, H-5); 7.58–7.66 (m, 2H, H-6,7-naphthyl); 7.77 (d, 1H, *J*_*6,5*_ = 3.9 Hz, H-6); 8.02 (m, 1H, H-5-naphthyl); 8.11 (d, 1H, *J*_*4,3*_ = 8.6 Hz, H-4-naphthyl); 8.18 (m, 1H, H-8-naphthyl); 8.30 (dd, 1H, *J*_*3,4*_ = 8.6 Hz, *J*_*3,1*_ = 1.8 Hz, H-3-naphthyl); 8.74 (bs, 1H, H-1-naphthyl); ^13^C NMR (125.7 MHz, DMSO-d_6_): 54.4 (CH_3_O); 61.6 (CH_2_–5′); 70.6 (CH-3′); 73.8 (CH-2′); 85.1 (CH-4′); 86.7 (CH-1′); 101.4 (CH-5); 111.4 (C-4a); 125.5 (CH-3-naphthyl); 126.5 (CH-6); 126.7 (CH-7-naphthyl); 127.5 (CH-5-naphthyl); 127.6 (CH-6-naphthyl); 128.4 (CH-4-naphthyl); 128.7 (CH-1-naphthyl); 129.1 (CH-8-naphthyl); 132.79 (C-8a-naphthyl); 133.8 (C-4a-naphthyl); 134.8 (C-2-naphthyl); 154.6 (C-7a); 157.4 (C-4); 161.4 (C-2). HR-ESI-MS: *found*: 408.1552 ([M + H]^+^, calcd for C_22_H_22_O_5_N_3_^+^: 408.1554); HR-ESI-MS: *found*: 430.1370 ([M + Na]^+^, calcd for C_22_H_21_O_5_N_3_Na^+^: 430.1373).

#### 2-(*N*-Methylamino)-4-(naphthalen-2-yl)-7-(β-d-ribofuranosyl)-7*H*-pyrrolo­[2,3-*d*]­pyrimidine (20G.7)

Compound **6B.7** (34.1 mg, 0.083 mmol) was treated with 2 mL MeNH_2_ (33% in EtOH), 0.02 mL TEA and stirred overnight at 80 °C. HPFC (SiO_2_, DCM/MeOH 1:0 → 5.7:1) and lyophilization from H_2_O/*t*-BuOH gave **20G.7** (27.4 mg, 81%) as a pale-yellow powder. HR-ESI-MS: *found*: 407.1711 ([M + H]^+^, calcd for C_22_H_23_O_4_N_4_^+^: 407.1714); ^1^H NMR (500 MHz, DMSO-d_6_): 2.93 (d, 3H, *J*_*CH3,NH*_ = 4.8 Hz, CH_3_NH); 3.54 (dt, 1H, *J*_*gem*_ = 11.7 Hz, *J*_*5′a,OH*_ = *J*_*5′a,4′*_ = 4.8 Hz, H-5′a); 3.66 (bdt, 1H, *J*_*gem*_ = 11.7 Hz, *J*_*5′b,OH*_ = *J*_*5′b,4′*_ = 4.8 Hz, H-5′b); 3.88 (td, 1H, *J*_*4′,5′a*_ = *J*_*4′,5′b*_ = 4.1 Hz, *J*_*4′,3′*_ = 3.7 Hz, H-4′); 4.12 (bq, 1H, *J*_*3′,2′*_ = *J*_*3′,OH*_ = *J*_*3′,4′*_ = 4.1 Hz, H-3′); 4.45 (q, 1H, *J*_*2′,1′*_ = *J*_*2′,OH*_ = *J*_*2′,3′*_ = 5.7 Hz, H-2′); 5.00 (t, 1H, *J*_*OH,5′a*_ = *J*_*OH,5′b*_ = 5.3 Hz, OH-5′); 5.16 (bs, 1H, OH-3′); 5.33 (d, 1H, *J*_*OH,2′*_ = 6.3 Hz, OH-2′); 6.13 (d, 1H, *J*_*1′,2′*_ = 6.1 Hz, H-1′); 6.82 (d, 1H, *J*_*5,6*_ = 3.9 Hz, H-5); 6.83 (m, 1H, CH_3_N**H**); 7.42 (d, 1H, *J*_*6,5*_ = 3.9 Hz, H-6); 7.56–7.63 (m; 2H, H-6,7-naphthyl); 7.99 (m, 1H, H-5-naphthyl); 8.07 (d, 1H, *J*_*4,3*_ = 8.6 Hz, H-4-naphthyl); 8.13 (m, 1H, H-8-naphthyl); 8.21 (bd, 1H, *J*_*3,4*_ = 8.6 Hz, H-3-naphthyl); 8.62 (s, 1H, H-1-naphthyl); ^13^C NMR (125.7 MHz, DMSO-d_6_): 28.5 (CH_3_NH); 61.9 (CH_2_–5′); 70.8 (CH-3′); 73.6 (CH-2′); 84.7 (CH-4′); 86.2 (CH-1′); 101.4 (CH-5); 108.1 (C-4a); 123.4 (CH-6); 125.7 (CH-3-naphthyl); 126.6 (CH-7-naphthyl); 127.2 (CH-6-naphthyl); 127.7 (CH-5-naphthyl); 128.18 and 128.20 (CH-1,4-naphthyl); 129.0 (CH-8-naphthyl); 132.8 (C-8a-naphthyl); 133.6 (C-4a-naphthyl); 135.7 (C-2-naphthyl); 155.0 (C-2); 156.9 (C-4); 159.8 (C-2). HR-ESI-MS: *found*: 429.1530 ([M + Na]^+^, calcd for C_22_H_22_O_4_N_4_Na^+^: 429.1533).

#### [(5-{[2-Methoxy-4-(naphthalen-2-yl)-7*H*-pyrrolo­[2,3-*d*]­pyrimidin-7-yl]-β-d-ribofuranosyl}­oxy)­phosphonomethyl]­phosphonic Acid (21F.7)

GP B using compound **19F.7** (56.8 mg, 0.14 mmol). HPLC (C-18, H_2_O + 0.05% TFA/MeCN 0 → 80%) gave **21F.7** (8.6 mg, 11%) as a pale-yellow powder. ^1^H NMR (500 MHz, DMSO-d_6_): 2.26 (t, 2H, d, *J*_*CH2,P*_ = 20.4 Hz, PCH_2_P); 4.05 (s, 3H, CH_3_O); 4.07–4.18 (m, 3H, H-4′,5′); 4.23 (dd, 1H, *J*_*3′,2′*_ = 5.2 Hz, *J*_*3′,4′*_ = 2.9 Hz, H-3′); 4.51 (dd, 1H, *J*_*2′,1′*_ = *J*_*2′,3′*_ = 5.7 Hz, H-2′); 6.22 (d, 1H, *J*_*2′,1′*_ = 6.2 Hz, H-1′); 7.08 (d, 1H, *J*_*5,6*_ = 3.9 Hz, H-5); 7.58–7.66 (m, 2H, H-6,7-naphthyl); 7.76 (d, 1H, *J*_*6,5*_ = 3.9 Hz, H-6); 8.02 (m, 1H, H-5-naphthyl); 8.11 (d, 1H, *J*_*4,3*_ = 8.6 Hz, H-4-naphthyl); 8.19 (m, 1H, H-8-naphthyl); 8.30 (dd, 1H, *J*_*3,4*_ = 8.6 Hz, *J*_*3,1*_ = 1.8 Hz, H-3-naphthyl); 8.75 (bd, 1H, *J*_*1,3*_ = 1.7 Hz, H-1-naphthyl); ^13^C NMR (125.7 MHz, DMSO-d_6_): 27.5 (t, *J*_*C,P*_ = 128.6 Hz, PCH_2_P); 54.5 (CH_3_O); 64.9 (d, *J*_*C,P*_ = 5.4 Hz, CH_2_–5′); 70.5 (CH-3′); 73.5 (CH-2′); 82.8 (d, *J*_*C,P*_ = 7.4 Hz, CH-4′); 86.5 (CH-1′); 101.7 (CH-5); 111.3 (C-4a); 125.5 (CH-3-naphthyl); 126.3 (CH-6); 126.7 (CH-7-naphthyl); 127.5 (CH-6-naphthyl); 127.6 (CH-5-naphthyl); 128.4 (CH-4-naphthyl); 128.8 (CH-1-naphthyl); 129.1 (CH-8-naphthyl); 132.8 (C-8a-naphthyl); 133.8 (C-4a-naphthyl); 134.8 (C-2-naphthyl); 154.7 (C-7a); 157.5 (C-4); 161.4 (C-2); ^31^P NMR (202.4 MHz, DMSO-d_6_): 15.80 and 19.69 (2 × bd, 2 × 1P, *J*_*P,P*_ = 7.5 Hz, PCH_2_P). HR-ESI-MS: *found*: 564.0938 ([M–H]^−^, calcd for C_23_H_24_O_10_N_3_P_2_^–^: 564.0942).

#### [(5-{[2-(*N*-Methylamino)-4-(naphthalen-2-yl)-7*H*-pyrrolo­[2,3-*d*]­pyrimidin-7-yl]-β-d-ribofuranosyl}­oxy)­phosphonomethyl]­phosphonic Acid (22G.7)

GP B using compound **20G.7** (51.4 mg, 0.13 mmol). HPLC purification (C-18, H_2_O + 0.05% TFA/MeCN 0 → 80%) gave **22G.7** (18.7 mg, 26%) as a yellow powder. ^1^H NMR (500 MHz, DMSO-d_6_): 2.24 (t, 2H, d, *J*_*CH2,P*_ = 20.4 Hz, PCH_2_P); 2.95 (s, 3H, C**H**_**3**_NH); 4.01–4.17 (m, 3H, H-4′,5′); 4.21 (dd, 1H, *J*_*3′,2′*_ = 5.2 Hz, *J*_*3′,4′*_ = 3.0 Hz, H-3′); 4.41 (t, 1H, *J*_*2′,1′*_ = *J*_*2′,3′*_ = 5.7 Hz, H-2′); 6.16 (d, 1H, *J*_*2′,1′*_ = 6.3 Hz, H-1′); 6.83 (d, 1H, *J*_*5,6*_ = 3.9 Hz, H-5); 7.46 (d, 1H, *J*_*6,5*_ = 3.9 Hz, H-6); 7.56–7.64 (m, 2H, H-6,7-naphthyl); 8.00 (m, 1H, H-5-naphthyl); 8.08 (d, 1H, *J*_*4,3*_ = 8.6 Hz, H-4-naphthyl); 8.14 (m, 1H, H-8-naphthyl); 8.21 (bd, 1H, *J*_*3,4*_ = 8.6 Hz, H-3-naphthyl); 8.62 (s 1H, H-1-naphthyl); ^13^C NMR (125.7 MHz, DMSO-d_6_): 25.5 (t, *J*_*C,P*_ = 128.4 Hz, PCH_2_P); 28.4 (CH_3_NH); 64.9 (d, *J*_*C,P*_ = 4.9 Hz, CH_2_–5′); 70.6 (CH-3′); 73.2 (CH-2′); 82.5 (d, *J*_*C,P*_ = 7.2 Hz, CH-4′); 86.1 (CH-1′); 101.7 (CH-5); 108.1 (C-4a); 123.7 (CH-6); 125.6 (CH-3-naphthyl); 126.6 (CH-7-naphthyl); 127.3 and 127.6 (CH-5,6-naphthyl); 128.2 (CH-4-naphthyl); 128.3 (CH-1-naphthyl); 128.9 (CH-8-naphthyl); 132.8 (C-8a-naphthyl); 133.6 (C-4a-naphthyl); 135.0 (C-2-naphthyl); 155.1 (C-7a); 156.4 (C-4); 159.3 (C-2); ^31^P NMR (202.4 MHz, DMSO-d_6_): 15.91 and 19.49 (2 × bd, 2 × 1P, *J*_*P,P*_ = 5.5 Hz, PCH_2_P). HR-ESI-MS: *found*: 563.1098 ([M–H]^−^, calcd for C_23_H_25_O_9_N_4_P_2_^–^: 563.1102).

#### 2-Amino-4-(5,6,7,8-tetrahydronaphthalen-2-yl)-7-(2,3-*O*-*iso*propylidene-5-*O*-*tert*-butyldimethylsilyl-β-d-ribofuranosyl)-7*H*-pyrrolo­[2,3-*d*]­pyrimidine (24C.23)

Compound **23** ^23^ (203.0 mg, 0.45 mmol) was reacted with 5,6,7,8-tetrahydronaphthalen-2-ylboronic acid (117.8 mg, 0.67 mmol) for 1 h at 100 °C according to the GP A. HPFC (SiO_2_, cH/EA 1:0 → 8:2) gave **24C.23** (180.2 mg, 73%) as a yellow oil. ^1^H NMR (500 MHz, DMSO-d_6_): – 0.02 and – 0.03 (2 × s, 2 × 3H, (CH_3_)_2_Si); 0.83 (s, 9H, (CH_3_)_3_C); 1.33 and 1.54 (2 × s, 2 × 3H, (CH_3_)_2_C); 1.73–1.81 (m, 4H, H-2,3-C_10_H_11_); 2.79 (m, 2H, H-4-C_10_H_11_); 2.82 (m, 2H, H-1-C_10_H_11_); 3.71 (dd, 1H, *J*_*gem*_ = 11.2 Hz, *J*_*5′a,4′*_ = 5.6 Hz, H-5′a); 3.74 (dd, 1H, *J*_*gem*_ = 11.2 Hz, *J*_*5′b,4′*_ = 4.7 Hz, H-5′b); 4.08 (btd, 1H, *J*_*4′,5′a*_ = *J*_*4′,5′b*_ = 5.1 Hz, *J*_*4′,3′*_ = 3.5 Hz, H-4′); 4.97 (dd, 1H, *J*_*3′,2′*_ = 6.4 Hz, *J*_*3′,4′*_ = 3.5 Hz, H-3′); 5.18 (dd, 1H, *J*_*2′,3′*_ = 6.4 Hz, *J*_*2′,1′*_ = 2.8 Hz, H-2′); 6.21 (d, 1H, *J*_*1′,2′*_ = 2.8 Hz, H-1′); 6.38 (s, 2H, NH_2_); 6.66 (d, 1H, *J*_*5,6*_ = 3.8 Hz, H-5); 7.20 (d, 1H, *J*_*8,7*_ = 8.0 Hz, H-8-C_10_H_11_); 7.29 (d, 1H, *J*_*6,5*_ = 3.8 Hz, H-6); 7.72–7.76 (m, 2H, H-5,7-C_10_H_11_); ^13^C NMR (125.7 MHz, DMSO-d_6_): – 5.43 and – 5.40 ((CH_3_)_2_Si); 18.0 ((CH_3_)_3_ **C**); 22.65 and 22.73 (CH_2_–2,3-C_10_H_11_); 25.3 ((**C**H_3_)_2_C); 25.8 ((**C**H_3_)_2_C); 27.1 ((**C**H_3_)_2_C); 28.8 and 28.9 (CH_2_–1,4-C_10_H_11_); 63.5 (CH_2_–5′); 80.8 (CH-3′); 83.5 (CH-2′); 85.6 (CH-4′); 88.0 (CH-1′); 101.7 (C-5); 107.7 (C-4a); 113.2 ((CH_3_)_2_ **C**); 123.2 (CH-6); 125.5 (CH-7-C_10_H_11_); 128.8 (CH-5-C_10_H_11_); 129.2 (CH-8-C_10_H_11_); 135.2 (C-6-C_10_H_11_); 136.9 (C-4a-C_10_H_11_); 138.6 (C-8a-C_10_H_11_); 154.2 (C-7a); 157.7 (C-4); 159.9 (C-2). HR-ESI-MS: *found*: 551.3050 ([M + H]^+^, calcd for C_30_H_43_O_4_N_4_Si^+^: 551.3048); HR-ESI-MS: *found*: 573.2870 ([M + Na]^+^, calcd for C_30_H_42_O_4_N_4_NaSi^+^: 573.2868).

#### 2-Iodo-4-(5,6,7,8-tetrahydronaphthalen-2-yl)-7-(2,3-*O*-*iso*propylidene-5-*O*-*tert*-butyldimethylsilyl-β-d-ribofuranosyl)-7*H*-pyrrolo­[2,3-*d*]­pyrimidine (25H.23)

Isopentyl nitrite (0.07 mL, 0.50 mmol) was added to a stirred mixture of **24C.23** (92.5 mg, 0.17 mmol), CuI (35.2 mg, 0.19 mmol), I_2_ (46.9 mg, 0.19 mmol) and CH_2_I_2_ (0.14 mL, 1.70 mmol) in 3 mL THF. After the completion of the reaction, volatiles were evaporated. HPFC (SiO_2_, cH/EA 1:0 → 9:1) gave **25H.23** (25.6 mg, 23%) as a yellow oil. ^1^H NMR (500 MHz, DMSO-d_6_): – 0.03 (s, 6H, (CH_3_)_2_Si); 0.81 (s, 9H, (CH_3_)_3_C); 1.34 and 1.56 (2 × s, 2 × 3H, (CH_3_)_2_C); 1.75–1.82 (m, 4H, H-2,3-C_10_H_11_); 2.81 (m, 2H, H-4-C_10_H_11_); 2.86 (m, 2H, H-1-C_10_H_11_); 3.72 (dd, 1H, *J*_*gem*_ = 11.2 Hz, *J*_*5′a,4′*_ = 5.8 Hz, H-5′a); 3.77 (dd, 1H, *J*_*gem*_ = 11.2 Hz, *J*_*5′b,4′*_ = 4.9 Hz, H-5′b); 4.17 (btd, 1H, *J*_*4′,5′a*_ = *J*_*4′,5′b*_ = 5.4 Hz, *J*_*4′,3′*_ = 3.4 Hz, H-4′); 4.94 (dd, 1H, *J*_*3′,2′*_ = 6.3 Hz, *J*_*3′,4′*_ = 3.4 Hz, H-3′); 5.27 (dd, 1H, *J*_*2′,3′*_ = 6.3 Hz, *J*_*2′,1′*_ = 2.5 Hz, H-2′); 6.30 (d, 1H, *J*_*1′,2′*_ = 2.5 Hz, H-1′); 7.02 (d, 1H, *J*_*5,6*_ = 3.8 Hz, H-5); 7.26 (d, 1H, *J*_*8,7*_ = 8.0 Hz, H-8-C_10_H_11_); 7.775 (d, 1H, *J*_*6,5*_ = 3.8 Hz, H-6); 7.779 (bd, 1H, *J*_*5,7*_ = 2.0 Hz, H-5-C_10_H_11_); 7.81 (dd, 1H, *J*_*7,8*_ = 7.9 Hz, *J*_*7,5*_ = 2.0 Hz, H-7-C_10_H_11_); ^13^C NMR (125.7 MHz, DMSO-d_6_): – 5.4 ((CH_3_)_2_Si); 18.0 ((CH_3_)_3_ **C**); 22.5 and 22.6 (CH_2_–2,3-C_10_H_11_); 25.3 ((**C**H_3_)_2_C); 25.8 ((**C**H_3_)_2_C); 27.1 ((**C**H_3_)_2_C); 28.8 and 28.9 (CH_2_–1,4-C_10_H_11_); 63.3 (CH_2_–5′); 80.9 (CH-3′); 83.8 (CH-2′); 86.3 (CH-4′); 89.0 (CH-1′); 101.7 (C-5); 113.4 ((CH_3_)_2_ **C**); 115.0 (C-4a); 119.9 (C-2); 125.9 (CH-7-C_10_H_11_); 128.6 (CH-6); 129.1 (CH-5-C_10_H_11_); 129.6 (CH-8-C_10_H_11_); 133.3 (C-6-C_10_H_11_); 137.5 (C-4a-C_10_H_11_); 140.3 (C-8a-C_10_H_11_); 151.8 (C-7a); 157.9 (C-4). HR-ESI-MS: *found*: 662.1903 ([M + H]^+^, calcd for C_30_H_41_O_4_N_3_ISi^+^: 662.1906); HR-ESI-MS: *found*: 684.1722 ([M + Na]^+^, calcd for C_30_H_40_O_4_N_3_INaSi^+^: 684.1725).

#### 2-Iodo-4-(5,6,7,8-tetrahydronaphthalen-2-yl)-7-(β-d-ribofuranosyl)-7*H*-pyrrolo­[2,3-*d*]­pyrimidine (26H.23)

Protected nucleoside **25H.23** (50.8 mg, 0.077 mmol) was dissolved in TFA (4 mL,75%) and stirred for 1 h at rt. Solvents were coevaporated 3 times with MeOH. HPFC (SiO_2_, DCM/MeOH 1:0 → 9:1) and lyophilization from H_2_O/*t*-BuOH gave compound **26H.3** (31.3 mg, 80%) as a white powder. ^1^H NMR (500 MHz, DMSO-d_6_): 1.75–1.82 (m, 4H, H-2,3-C_10_H_11_); 2.81 and 2.86 (2 × m, 2 × 2H, H-1,4-C_10_H_11_); 3.55 (ddd, 1H, *J*_*gem*_ = 11.8 Hz, *J*_*5′a,OH*_ = 5.5 Hz, *J*_*5′a,4′*_ = 3.9 Hz, H-5′a); 3.63 (ddd, 1H, *J*_*gem*_ = 11.8 Hz, *J*_*5′b,OH*_ = 5.5 Hz, *J*_*5′b,4′*_ = 4.3 Hz, H-5′a); 3.94 (td, 1H, *J*_*4′,5′a*_ = *J*_*4′,5′b*_ = 4.1 Hz, *J*_*4′,3′*_ = 2.9 Hz, H-4′); 4.11 (td, 1H, *J*_*3′,2′*_ = *J*_*3′,OH*_ = 5.0 Hz, *J*_*3′,4′*_ = 2.9 Hz, H-3′); 4.44 (td, 1H, *J*_*2′,1′*_ = *J*_*2′,OH*_ = 6.5 Hz, *J*_*2′,3′*_ = 5.0 Hz, H-2′); 5.03 (t, 1H, *J*_*OH,5′a*_ = *J*_*OH,5′b*_ = 5.5 Hz, OH-5′); 5.25 (d, 1H, *J*_*OH,3′*_ = 4.9 Hz, OH-3′); 5.42 (d, 1H, *J*_*OH,2′*_ = 6.5 Hz, OH-2′); 6.15 (d, 1H, *J*_*1′,2′*_ = 6.4 Hz, H-1′); 7.01 (d, 1H, *J*_*5,6*_ = 3.8 Hz, H-5); 7.27 (d, 1H, *J*_*8,7*_ = 8.0 Hz, H-8-C_10_H_11_); 7.78 (d, 1H, *J*_*5,7*_ = 1.9 Hz, H-5-C_10_H_11_); 7.81 (dd, 1H, *J*_*7,8*_ = 7.9 Hz, *J*_*7,5*_ = 1.9 Hz, H-7-C_10_H_11_); 7.87 (d, 1H, *J*_*6,5*_ = 3.8 Hz, H-6); ^13^C NMR (125.7 MHz, DMSO-d_6_): 22.55 and 22.63 (CH_2_–2,3-C_10_H_11_); 28.8 and 28.9 (CH_2_–1,4-C_10_H_11_); 61.6 (CH_2_–5′); 70.7 (CH-3′); 74.0 (CH-2′); 85.5 (CH-4′); 86.3 (CH-1′); 101.6 (CH-5); 114.9 (C-4a); 119.9 (C-2); 125.9 (CH-7-C_10_H_11_); 127.8 (CH-6); 129.1 (CH-5-C_10_H_11_); 129.6 (CH-8-C_10_H_11_); 133.5 (C-6-C_10_H_11_); 137.5 (C-4a-C_10_H_11_); 140.2 (C-8a-C_10_H_11_); 152.7 (C-7a); 157.8 (C-4). HR-ESI-MS: *found*: 508.0729 ([M + H]^+^, calcd for C_21_H_23_O_4_N_3_I^+^: 508.0728); HR-ESI-MS: *found*: 530.0549 ([M + Na]^+^, calcd for C_21_H_22_O_4_N_3_INa^+^: 530.0547).

#### [(5-{[2-Iodo-4-(5,6,7,8-tetrahydronaphthalen-2-yl)-7*H*-pyrrolo­[2,3-*d*]­pyrimidin-7-yl]-β-d-ribofuranosyl}­oxy)­phosphonomethyl]­phosphonic Acid (27H.23)

GP B using compound **26H.23** (27.5 mg, 0.054 mmol). HPLC (C-18, H_2_O + 0.05% TFA/MeCN 0 → 80%) gave **27H.23** (18.2 mg, 50%) as a white powder. ^1^H NMR (500 MHz, DMSO-d_6_): 1.74–1.83 (m, 4H, H-2,3-C_10_H_11_); 2.25 (t, 2H, *J*_*CH2,P*_ = 20.4 Hz, PCH_2_P); 2.81 (m, 2H, H-4-C_10_H_11_); 2.86 (m, 2H, H-1-C_10_H_11_); 4.07–4.13 (m, 3H, H-4′,5′); 4.19 (dm, 1H, *J*_*3′,2′*_ = 5.1 Hz, H-3′); 4.46 (dd, 1H, *J*_*2′,1′*_ = 6.6 Hz, *J*_*2′,3′*_ = 5.1 Hz, H-2′); 6.20 (d, 1H, *J*_*1′,2′*_ = 6.6 Hz, H-1′); 7.00 (d, 1H, *J*_*5,6*_ = 3.8 Hz, H-5); 7.27 (d, 1H, *J*_*8,7*_ = 8.0 Hz, H-8-C_10_H_11_); 7.78 (d, 1H, *J*_*5,7*_ = 1.9 Hz, H-5-C_10_H_11_); 7.81 (dd, 1H, *J*_*7,8*_ = 7.9 Hz, *J*_*7,5*_ = 1.9 Hz, H-7-C_10_H_11_); 7.88 (d, 1H, *J*_*6,5*_ = 3.8 Hz, H-6); ^13^C NMR (125.7 MHz, DMSO-d_6_): 22.64 and 22.62 (CH_2_–2,3-C_10_H_11_); 27.5 (t, *J*_*C,P*_ = 128.4 Hz, PCH_2_P); 28.8 and 28.9 (CH_2_–1,4-C_10_H_11_); 64.7 (d, *J*_*C,P*_ = 4.8 Hz, CH_2_–5′); 70.5 (CH-3′); 73.7 (CH-2′); 83.3 (d, *J*_*C,P*_ = 7.6 Hz, CH-4′); 86.0 (CH-1′); 101.9 (CH-5); 114.8 (C-4a); 120.0 (C-2); 125.8 (CH-7-C_10_H_11_); 127.7 (CH-6); 129.1 (CH-5-C_10_H_11_); 129.6 (CH-8-C_10_H_11_); 133.4 (C-6-C_10_H_11_); 137.5 (C-4a-C_10_H_11_); 140.2 (C-8a-C_10_H_11_); 152.8 (C-7a); 157.8 (C-4); ^31^P NMR (202.4 MHz, DMSO-d_6_): 15.83 and 19.61 (2 × s, 2 × 1P, PCH_2_P). HR-ESI-MS: *found*: 664.0118 ([M–H]^−^, calcd for C_22_H_25_O_9_N_3_IP_2_^–^: 664.0116).

#### [(5-{[6-Chloro-9*H*-purin-9-yl]-β-d-ribofuranosyl}­oxy)­phosphonomethyl]­phosphonic Acid (29)

GP B using 6-chloro-(β-d-ribofuranosyl)-9*H*-purine **28** (576.2 mg, 2.01 mmol). RP-HPFC (C-18, H_2_O/MeOH 0 → 100%) gave product **29** (84.6 mg, 9%) as a white powder. ^1^H NMR (500 MHz, DMSO-d_6_): 2.26 (t, 2H, *J*_*CH2,P*_ = 20.5 Hz, PCH_2_P); 4.10–4.22 (m, 3H, H-4′,5′); 4.26 (dd, 1H, *J*_*3′,2′*_ = 5.0 Hz, *J*_*3′,4′*_ = 3.4 Hz, H-3′); 4.64 (t, 1H, *J*_*2′,1′*_ = *J*_*2′,3′*_ = 5.2 Hz, H-2′); 6.07 (d, 1H, *J*_*1′,2′*_ = 5.5 Hz, H-1′); 8.82 (s, 1H, H-2); 8.94 (s, 1H, H-8); ^13^C NMR (125.7 MHz, DMSO-d_6_): 27.6 (t, *J*_*C,P*_ = 129.2 Hz, PCH_2_P); 64.5 (d, *J*_*C,P*_ = 5.6 Hz, CH_2_–5′); 70.1 (CH-3′); 73.7 (CH-2′); 83.5 (d, *J*_*C,P*_ = 7.4 Hz, CH-4′); 87.8 (CH-1′); 131.3 (CH-5); 145.7 (CH-8); 149.3 (C-6); 151.8 (C-4); 151.9 (CH-2); ^31^P NMR (202.4 MHz, DMSO-d_6_): 15.62 and 19.99 (2 × d, 2 × 1P, *J*_*P,P*_ = 8.1 Hz, PCH_2_P). HR-ESI-MS: *found*: 442.9930 ([M–H]^−^, calcd for C_11_H_14_O_9_N_4_ClP_2_^–^: 442.9930).

#### 6-(Naphth-1-yl)-7-(β-d-ribofuranosyl)-9*H*-purin-9-yl (30A.6)

6-Chloro-(β-d-ribofuranosyl)-9*H*-purine **28** (104.0 mg, 0.36 mmol) was reacted with naphthalene-1-boronic acid (93.6 mg, 0.54 mmol) for 1 h at 100 °C according to the GP A. HPFC (SiO_2_, DCM/MeOH 1:0 → 9:1) gave **30A.6** (114.5 mg, 83%) as a white powder. ^1^H NMR (500 MHz, DMSO-d_6_): 3.61 (ddd, 1H, *J*_*gem*_ = 11.9 Hz, *J*_*5′a,OH*_ = 6.0 Hz, *J*_*5′a,4′*_ = 4.0 Hz, H-5′a); 3.72 (ddd, 1H, *J*_*gem*_ = 11.9 Hz, *J*_*5′b,OH*_ = 5.2 Hz, *J*_*5′b,4′*_ = 4.1 Hz, H-5′b); 4.02 (q, 1H, *J*_*4′,5′a*_ = *J*_*4′,5′b*_ = *J*_*4′,3′*_ = 4.0 Hz, H-4′); 4.23 (td, 1H, *J*_*3′,2′*_ = *J*_*3′,OH*_ = 5.0 Hz, *J*_*3′,4′*_ = 3.6 Hz, H-3′); 4.71 (td, 1H, *J*_*2′,1′*_ = *J*_*2′,OH*_ = 5.9 Hz, *J*_*2′,3′*_ = 5.0 Hz, H-2′); 5.13 (t, 1H, *J*_*OH,5′a*_ = *J*_*OH,5′b*_ = 5.5 Hz, OH-5′); 5.29 (d, 1H, *J*_*OH,3′*_ = 5.0 Hz, OH-3′); 5.59 (d, 1H, *J*_*OH,2′*_ = 6.1 Hz, OH-2′); 6.13 (d, 1H, *J*_*1′,2′*_ = 5.7 Hz, H-1′); 7.51 (ddd, 1H, *J*_*7,8*_ = 8.6 Hz, *J*_*7,6*_ = 6.8 Hz, *J*_*7,5*_ = 1.4 Hz, H-7-naphthyl); 7.58 (ddd, 1H, *J*_*6,5*_ = 8.3 Hz, *J*_*6,7*_ = 6.8 Hz, *J*_*6,8*_ = 1.3 Hz, H-6-naphthyl); 7.70 (dd, 1H, *J*_*3,4*_ = 8.2 Hz, *J*_*3,2*_ = 7.1 Hz, H-3-naphthyl); 7.97 (dd, 1H, *J*_*2,3*_ = 7.1 Hz, *J*_*2,4*_ = 1.3 Hz, H-2-naphthyl); 8.06 (bd, 1H, *J*_*5,6*_ = 8.3 Hz, H-5-naphthyl); 8.14 (dm, 1H, *J*_*4,3*_ = 8.4 Hz, H-4-naphthyl). 8.15 (dm, 1H, *J*_*8,7*_ = 8.6 Hz, H-8-naphthyl); 8.86 (s, 1H, H-8); 9.12 (s, 1H, H-2); ^13^C NMR (125.7 MHz, DMSO-d_6_): 61.3 (CH_2_–5′); 70.4 (CH-3′); 73.7 (CH-2′); 85.8 (CH-4′); 87.7 (CH-1′); 125.2 (CH-3-naphthyl); 125.8 (CH-8-naphthyl); 126.2 (CH-6-naphthyl); 126.7 (CH-7-naphthyl); 128.3 (CH-5-naphthyl); 129.7 (CH-2-naphthyl); 130.2 (CH-4-naphthyl); 130.5 (C-8a-naphthyl); 132.4 (C-1-naphthyl); 132.7 (C-5); 133.4 (C-4a-naphthyl); 145.2 (CH-8); 151.8 (CH-2); 151.8 (C-4); 156.7 (C-6). HR-ESI-MS: *found*: 379.1398 ([M + H]^+^, calcd for C_20_H_19_O_4_N_4_^+^: 379.1401); HR-ESI-MS: *found*: 401.1217 ([M + Na]^+^, calcd for C_20_H_18_O_4_N_4_Na^+^: 401.1220).

#### [(5-{[6-(Naphth-1-yl)-9*H*-purin-9-yl]-β-d-ribofuranosyl}­oxy)­phosphonomethyl]­phosphonic Acid (31A.6)

GP B using compound **30A.6** (53.4 mg, 0.14 mmol). HPLC (C-18, H_2_O + 0.05% TFA/MeCN 0 → 80%) gave **31A.6** (13.5 mg, 18%) as a pale-yellow powder. ^1^H NMR (500 MHz, DMSO-d_6_): 2.26 (t, 2H, d, *J*_*CH2,P*_ = 20.5 Hz, PCH_2_P); 4.12–4.23 (m, 3H, H-4′,5′); 4.30 (m, 1H, H-3′); 4.74 (t, 1H, *J*_*2′,1′*_ = *J*_*2′,3′*_ = 5.4 Hz, H-2′); 6.17 (d, 1H, *J*_*2′,1′*_ = 5.8 Hz, H-1′); 7.52 (ddd, 1H, *J*_*7,8*_ = 8.5 Hz, *J*_*7,6*_ = 6.8 Hz, *J*_*7,5*_ = 1.3 Hz, H-7-naphthyl); 7.59 (ddd, 1H, *J*_*6,5*_ = 8.2 Hz, *J*_*6,7*_ = 6.8 Hz, *J*_*6,8*_ = 1.3 Hz, H-6-naphthyl); 7.70 (dd, 1H, *J*_*3,4*_ = 8.2 Hz, *J*_*3,2*_ = 7.2 Hz, H-3-naphthyl); 7.98 (dd, 1H, *J*_*2,3*_ = 7.8 Hz, *J*_*2,4*_ = 1.3 Hz, H-2-naphthyl); 8.06 (bd, 1H, *J*_*5,6*_ = 8.2 Hz, H-5-naphthyl); 8.14 (dm, 1H, *J*_*4,3*_ = 8.2 Hz, H-4-naphthyl); 8.17 (dm, 1H, *J*_*8,7*_ = 8.5 Hz, H-8-naphthyl); 8.87 (s, 1H, H-8); 9.13 (s, 1H, H-2); ^13^C NMR (125.7 MHz, DMSO-d_6_): 27.6 (t, *J*_*C,P*_ = 129.0 Hz, PCH_2_P); 64.6 (d, *J*_*C,P*_ = 5.5 Hz, CH_2_–5′); 70.3 (CH-3′); 73.5 (CH-2′); 83.4 (d, *J*_*C,P*_ = 7.3 Hz, CH-4′); 87.2 (CH-1′); 125.2 (CH-3-naphthyl); 125.8 (CH-8-naphthyl); 126.2 (CH-6-naphthyl); 126.7 (CH-7-naphthyl); 128.3 (CH-5-naphthyl); 129.7 (CH-2-naphthyl); 130.2 (CH-4-naphthyl); 130.5 (C-8a-naphthyl); 132.4 and 132.6 (C-5,C-1-naphthyl); 133.4 (C-4a-naphthyl); 145.0 (CH-8); 151.9 (CH-2); 152.0 (C-4); 156.6 (C-6); ^31^P NMR (202.4 MHz, DMSO-d_6_): 15.67 and 19.91 (2 × d, 2 × 1P, *J*_*P,P*_ = 7.8 Hz, PCH_2_P). HR-ESI-MS: *found*: 535.0785 ([M–H]^−^, calcd for C_21_H_21_O_9_N_4_P_2_^–^: 535.0789).

#### 4-(Phenyl)-7-(2-deoxy-2-fluoro-β-d-arabinosyl)-7*H*-pyrrolo­[2,3-*d*]­pyrimidine (33A.5)

GP C using nucleoside **32** ^24^ (500 mg, 1.01 mmol). HPFC (SiO_2_, cHex/DCM/MeOH 1:1:0 → 1:1:0.23) gave **33A.5** (213.0 mg, 64%) as a white powder. ^1^H NMR (500 MHz, DMSO-d_6_): 3.66 (bdt, 1H, *J*_*gem*_ = 12.0 Hz, *J*_*5′a,OH*_ = *J*_*5′a,4′*_ = 5.4 Hz, H-5′a); 3.72 (dddd, 1H, *J*_*gem*_ = 12.0 Hz, *J*_*5′b,OH*_ = 5.4 Hz, *J*_*5′b,4′*_ = 4.5 Hz, *J*_*5′b,F*_ = 1.4 Hz, H-5′b); 3.88 (bq, 1H, *J*_*4′,5′a*_ = *J*_*4′,5′b*_ = *J*_*4′,3′*_ = 4.9 Hz, H-4′); 4.44 (bdq, 1H, *J*_*3′,F*_ = 19.1 Hz, *J*_*3′,2′*_ = *J*_*3′,4′*_ = *J*_*3′,OH*_ = 4.6 Hz, H-3′); 5.13 (t, 1H, *J*_*OH,5′a*_ = *J*_*OH,5′b*_ = 5.6 Hz, OH-5′); 5.24 (ddd, 1H, *J*_*2′,F*_ = 52.7 Hz, *J*_*2′,1′*_ = 4.6 Hz, *J*_*2′,3′*_ = 3.8 Hz, H-2′); 5.98 (d, 1H, *J*_*OH,3′*_ = 4.9 Hz, OH-3′); 6.79 (dd, 1H, *J*_*1′,F*_ = 15.1 Hz, *J*_*1′,2′*_ = 4.6 Hz, H-1′); 7.02 (d, 1H, *J*_*5,6*_ = 3.8 Hz, H-5); 7.58 (m, 1H, H-*p*-Ph); 7.60 (m; 2H, H-*m*-Ph); 7.85 (dd, 1H, *J*_*6,5*_ = 3.8 Hz, *J*_*6,F*_ = 2.2 Hz, H-6); 8.17 (m, 2H, H-*o*-Ph); 8.92 (s, 1H, H-2); ^13^C NMR (125.7 MHz, DMSO-d_6_): 60.5 (CH_2_–5′); 72.8 (d, *J*_*C,F*_ = 23.2 Hz, CH-3′); 81.2 (d, *J*_*C,F*_ = 16.8 Hz, CH-1′); 83.2 (d, *J*_*C,F*_ = 5.2 Hz, CH-4′); 95.9 (d, *J*_*C,F*_ = 191.6 Hz, CH-2′); 101.0 (CH-5); 115.0 (C-4a); 128.7 (CH-*o*-Ph); 129.0 (CH-*m*-Ph); 129.0 (bd, *J*_*C,F*_ = 3.6 Hz, CH-6); 130.4 (CH-*p*-Ph); 137.4 (C-*i*-Ph); 151.2 (CH-2); 151.6 (C-7a); 156.2 (C-4); ^19^F NMR (470.4 MHz, DMSO-d_6_): – 194.68 (dt, 1F, *J*_*F,2′*_ = 52.7 Hz, *J*_*F,1′*_ = *J*_*F,3′*_ = 17.0 Hz, F-2′). HR-ESI-MS: *found*: 330.12470 ([M + H]^+^, calcd for C_17_H_17_O_3_N_3_F^+^: 330.12485); HR-ESI-MS: *found*: 352.10662 ([M + Na]^+^, calcd for C_17_H_16_O_3_N_3_FNa^+^: 352.10679).

#### [(5-{[4-Phenyl-7*H*-pyrrolo­[2,3-*d*]­pyrimidin-7-yl]-2-deoxy-2-fluoro-β-d-arabinosyl}­oxy)­phosphonomethyl]­phosphonic Acid (34A.5)

GP B using compound **33A.5** (202 mg, 0.6 mmol). HPFC (DEAE Sepharose fast flow, H_2_O/TEAB 400 mM 0 → 100%), HPLC (H_2_O 100 M TEAB/MeCN 80% 1 M TEAB 0 → 40%) and lyophilization from H_2_O gave **34A.5** (55 mg, 14%) as a white powder. ^1^H NMR (600.1 MHz, D_2_O): 2.23 (t, 2H, *J*_*CH2,P*_ = 19.9 Hz, PCH_2_P); 4.20–4.29 (m, 3H, H-4′,5′); 4.77 (bdt, 1H, *J*_*3′,F*_ = 19.4 Hz, *J*_*3′,2′*_ = *J*_*3′,4′*_ = 4.4 Hz, H-3′); 5.36 (ddd, 1H, *J*_*2′,F*_ = 51.9 Hz, *J*_*2′,1′*_ = 4.7 Hz, *J*_*2′,3′*_ = 3.9 Hz, H-2′); 6.84 (dd, 1H, *J*_*1′,F*_ = 14.5 Hz, *J*_*1′,2′*_ = 4.7 Hz, H-1′); 6.98 (d, 1H, *J*_*5,6*_ = 3.9 Hz, H-5); 7.61–7.68 (m; 3H, H-*m*,*p*-Ph); 7.91 (dd, 1H, *J*_*6,5*_ = 3.9 Hz, *J*_*6,F*_ = 2.4 Hz, H-6); 7.95 (m, 2H, H-*o*-Ph); 8.82 (s, 1H, H-2); ^13^C NMR (150.9 MHz, D_2_O): 29.6 (t, *J*_*C,P*_ = 124.8 Hz, PCH_2_P); 64.8 (d, *J*_*C,P*_ = 5.1 Hz, CH_2_–5′); 75.1 (d, *J*_*C,F*_ = 24.9 Hz, CH-3′); 83.3 (dd, *J*_*C,P*_ = 7.8 Hz, *J*_*C,F*_ = 5.4 Hz, CH-4′); 83.8 (d, *J*_*C,F*_ = 17.0 Hz, CH-1′); 97.0 (d, *J*_*C,F*_ = 192.1 Hz, CH-2′); 104.6 (CH-5); 118.33 (C-4a); 130.9 (CH-*o*-Ph); 131.17 (CH-*m*-Ph); 131.9 (d, *J*_*C,F*_ = 4.1 Hz, CH-6); 133.2 (CH-*p*-Ph); 137.1 (C-*i*-Ph); 151.57 (CH-2); 153.1 (C-7a); 158.8 (C-4); ^19^F NMR (470.4 MHz, D_2_O): – 195.59 (dt, 1F, *J*_*F,2′*_ = 51.9 Hz, *J*_*F,1′*_ = *J*_*F,3′*_ = 16.96 Hz, F-2′); ^31^P NMR (202.4 MHz, D_2_O): 15.55 and 19.14 (2 × d, 2 × 1P, *J*_*P,P*_ = 8.8 Hz, PCH_2_P). HR-ESI-MS: *found*: 486.06332 ([M – H]^−^, calcd for C_18_H_19_O_8_N_3_FP_2_^–^: 486.06369).

### In Vitro ADME Procedures

#### Kinetic Solubility

Test compounds were prepared as 10 mM stock solutions in DMSO and diluted in phosphate-buffered saline (PBS, pH 7.4) to a 100 μM solution, which was then aliquoted in duplicate. The duplicate samples were incubated in Multiscreen PCF Filter Plates (Merck Millipore) with sealed bottom at room temperature on a shaker for 2 h to allow equilibration. Following incubation, the samples were centrifuged into nonsterile, flat-bottom 96-well plates at 2,200 RCF for 12 min. Supernatants were then diluted 1:99 in a solution of 50% acetonitrile containing 0.1% DMSO. The diluted samples were transferred to 384-well Echo plates, which contained prespotted calibration standards prepared by Echo transfer of 10 mM and 1 mM DMSO stock solutions, resulting in calibration points ranging from 0.05 to 4 μM. Samples and standards were subsequently analyzed by Echo-MS.

#### Chemical Stability Assay

Chemical stability was evaluated in three buffer systems: (a) pH 7.4 PBS, (b) pH 10.0 CHES, (c) pH 1.6 buffer – diluted FASSGF; aqueous HCl and NaCl, representing a simplified acidic gastric environment without additional physiological components. Compounds (10 mM in DMSO) were dispensed into 384-well plates (15 nL/well) using an ECHO acoustic dispenser (Labcyte). All samples were prepared in triplicate. After buffer addition (15 μL), plates were incubated at room temperature with shaking (500 rpm) for 0 and 4 h. Then, 60 μL of ice-cold 100% methanol was added, and plates were shaken for 5 min. A 10 μL aliquot was diluted with 40 μL of 30% methanol (v/v in water) and analyzed immediately by ECHO-MS.

#### Plasma Stability Assay

The plasma stability of the compounds was evaluated by incubating 10 μM solutions with human or mouse pooled plasma (human - Biowest, mouse - Innovative Research) at 37 °C for 10, 60, and 120 min. Four volumes of ice-cold methanol were added to terminate the reactions. The samples were then thoroughly mixed, stored at – 20 °C for 30 min and left overnight at 8 °C. Before analysis, the samples were centrifuged at 2,000RCF at 8 °C for 20 min. The supernatants were diluted with four volumes of 30% methanol in MQ and analyzed using the Echo MS system (SCIEX). Zero time points were prepared by rapid stopping with methanol immediately after plasma addition.

#### Microsomal Stability Assay

The stability of the compounds in human or mouse liver microsomes was evaluated using pooled microsomes (Thermo Fisher Scientific) at a concentration of 0.5 mg/mL. Compounds were tested at a concentration of 10 μM in 90 mM Tris-HCl buffer (pH 7.4) supplemented with 2 mM NADPH and 2 mM MgCl_2_. Incubations were performed at 37 °C for 10, 30, and 45 min. To stop the reactions, four volumes of ice-cold methanol were added, followed by vigorous mixing. The samples were then stored at – 20 °C for 30 min and subsequently left overnight at 8 °C. After centrifugation at 2,000 RCF and 8 °C for 20 min, the supernatants were diluted with four volumes of 30% methanol in water and analyzed using the Echo-MS system (SCIEX). Zero-time samples were generated by adding methanol immediately after the microsomes were introduced.

Plasma and microsomal half-lives (t_1_/_2_) were obtained by fitting compound depletion data to a one-phase exponential decay model in CDD Vault. The rate constant (*k*) from the fit was used to calculate t_1_/_2_ as t_1_/_2_ = 0.693/*k*. Intrinsic clearance (CLint) was calculated using CLint = *V* × ln2/*t_1_/*_*2*_, where *V* is the incubation volume per milligram of microsomal protein (μL/mg).

#### Plasma Protein Binding Assay

Plasma protein binding of the investigated compounds was determined using equilibrium dialysis in single-use Rapid Equilibrium Dialysis devices with an 8 kDa molecular weight cutoff (Thermo Fisher Scientific), following the manufacturer’s protocol. In brief, 50 μL of undiluted human plasma (Biowest), spiked with the test compound at a final concentration of 100 μM, was added to the sample chamber, while 300 μL of phosphate-buffered saline (PBS) was placed in the buffer chamber. Devices were incubated for 4 h at 37 °C with gentle shaking.

After dialysis, 7.5 μL aliquots from both the plasma and buffer chambers were transferred directly into wells of a 384-well plate. To equalize the matrices for downstream processing, 7.5 μL of PBS was added to each plasma aliquot, and 7.5 μL of plasma was added to each buffer aliquot. Proteins were precipitated by adding four volumes of ice-cold methanol to each well, followed by thorough mixing and incubation at – 20 °C for 30 min. Plates were then stored overnight at 8 °C. Prior to analysis, the plate was centrifuged at 2,000 × g for 20 min at 8 °C. The resulting supernatants were diluted with four volumes of 30% methanol in Milli-Q water and analyzed using an Echo MS system (SCIEX). The fraction of unbound compound was calculated using the following equations:

%Free=c(bufferchamber)/c(plasmachamber)*100%

%Bound=100%−%Free

#### In Vitro CYP450 Inhibition Assay

The study was performed by Bienta (Enamine Biology Services). The potential for CYP450 inhibition (isoforms 1A2, 2C9, 2C19, 2D6, and 3A4) was assessed by performing *in vitro* inhibition studies using fluorogenic CYP450 substrates with the corresponding CYP450 enzymes and NADPH regeneration system (Vivid CYP450 Screening Kits) with some minor changes to the manufacturer’s protocols. The fluorescent signal produced from the reaction is directly proportional to the cytochrome P450 activity. In the cases when tested compounds interfere with the CYP450 enzyme–substrate reaction, the fluorescent signal decreases.

Briefly, the tested compounds were first dissolved in DMSO at 100× concentration (1 mM) and diluted in buffer to 2.5× concentration (25 μM). Then the 2.5× compound solutions were mixed with the Master Premix consisting of Human CYP450+Oxidoreductase and NADP+ Regeneration System (glucose-6-phosphate and glucose-6-phosphate dehydrogenase). After 10 min of preincubation, the enzymatic reaction was initiated by the addition of a mix of NADPH and the appropriate CYP450 substrates. The plate was incubated for the desired reaction time (25 min for CYP1A2, CYP2C9, CYP2D6, and CYP3A4; 60 min for CYP2C19) after which Stop Reagent was added and fluorescence measured using SpectraMax Paradigm Multi-Mode Microplate Reader. All test points were performed in quadruplicates at a concentration of 10 μM (1% DMSO).

#### hERG Inhibition Assay

The study was performed by Bienta (Enamine Biology Services). HEK293-hERG cells (stably expressing human potassium channel hERG – human Ether-a-go-go Related Gene) were cultured in DMEM/F-12 medium supplemented with 10% FBS, 100 U/mL penicillin, 100 μg/mL streptomycin, and 500 μg/mL G418. The assay was performed according to the manufacturer’s instructions. Cells were seeded into a 384-well poly-d-lysine, clear-bottom microtiter plate (8000 cells/well) in DMEM/F-12 medium supplemented with 5% FBS, 100 U/mL penicillin, 100 μg/mL streptomycin 24 h prior to assay. After 24 h of cell growth, the conditioned medium was substituted with the Dye Loading Solution comprising thallium-sensitive dye, followed by incubation for 1 h in a CO_2_ incubator. After the incubation, the Dye Loading Solution was substituted with a 15 μL/well mixture of the Assay Buffer and the TRS. Then, 5 μL of the test compound diluted in the assay buffer (0.5% DMSO final concentration) was added to each well of the plate and incubated at room temperature for 30 min hERG inhibition by test compounds was assessed at 3 concentrations of 10–50–100 μM. All test points were performed in quadruplicates. Then, the stimulation buffer containing thallium and potassium was added to each well, and the intracellular fluorescence was measured in a kinetic assay at 470–495 nm excitation and 515–575 emission filter set (every 3 s for 3 min). DMSO was used as a negative control, and Haloperidol was used as a reference compound to verify the assay validity. Haloperidol was dissolved in 100% DMSO at a concentration of 20 mM and stored at – 20 °C (for no more than a month). Haloperidol was assessed at the final concentrations ranging from 0.023 μM to 50.0 μM (8 points, 3-fold serial dilutions). The test compounds were dissolved in 100% DMSO at a concentration of 20 mM.

### Cell Culture

All cell lines were grown and maintained at 37 °C, 5% CO_2_, > 95% humidity, and ambient oxygen levels in a cell culture incubator. All media were supplemented with 10% fetal bovine serum (FBS, Gibco); additionally, RPMI 1640 was supplemented with 2 mM glutamine (complete RPMI). Cell lines were obtained and cultured as follows: MDA-MB-231 (kindly provided by Dr. Cyril Bařinka) were grown in RPMI 1640 (Biosera, Biotech), HEK293T (kindly provided by Dr. Kvido Stříšovský) were grown in DMEM (Sigma-Aldrich), and CD39-transfected HEK293T cells (clonal line 293T-CD39–27), which have been described previously,^18^ were grown in DMEM (Sigma-Aldrich). Isolated CD8+ T cells were cultivated in complete RPMI.

### Isolation of Human CD8 T Cells

Peripheral blood was obtained from healthy donors at the Military University Hospital Prague, following informed consent. CD8+ T cells were enriched using the RosetteSep Human CD8+ T Cell Enrichment Cocktail (STEMCELL Technologies) according to the manufacturer’s instructions and subsequently isolated by density gradient centrifugation using SepMate tubes (STEMCELL Technologies). Residual red blood cells were lysed using a red blood cell lysis buffer (155 mM NH_4_Cl, 12 mM NaHCO_3_, 0.1 mM EDTA, pH = 7.1–7.4). The isolated CD8+ T cells were resuspended in complete RPMI and counted using an automated cell counter.

### T Cell Activation Assay

CD8+ T cells were seeded at 5 × 10^4^ cells/well in 50 μL of complete RPMI in a flat-bottom 96-well plate. Next, 20 μL of 12.5 μM EHNA (erythro-9-(2-hydroxy-3-nonyl)­adenine; STEMCELL Technologies) was added to each well. This was followed by the addition of 10 μL of a CD73 inhibitor at either 5 μM or 500 nM (experimental wells) or 10 μL of complete RPMI (control wells). Subsequently, 20 μL of 5 mM AMP was added to all wells except those serving as controls for maximal T cell activation. After a 1-h incubation at 37 °C, 2.5 × 10^4^ activation beads (Dynabeads Human T-Activator CD3/CD28; Gibco) were washed and resuspended in 50 μL of complete RPMI per well. Cells were incubated for 60 h at 37 °C in a humidified CO_2_ incubator.

Following incubation, supernatants were collected for quantification of IFN-γ and granzyme B using ELISA. Cells were washed with PBS and stained with Zombie viability dye (diluted 1:200 in PBS; Zombie NIR Fixable Viability Kit, BioLegend) for 20 min at room temperature in the dark. After washing with FACS buffer (2 mM EDTA, 0.5% BSA in PBS), cells were stained with anti-CD8 (diluted 1:50, APC-conjugated, clone RPA-T8, BD Pharmingen), anti-CD25 (diluted 1:100, PE-CF594-conjugated, clone M-A251, BD Horizon), and anti-CD73 (diluted 1:50, AF488-conjugated, clone AD-2, Exbio) antibodies, all diluted in FACS buffer for 30 min at 4 °C in the dark. Finally, stained cells were washed and analyzed using a BD LSRFortessa flow cytometer. Data were processed and analyzed using FlowJo software (version 10.8.1) and GraphPad Prism 10.

### Quantification of IFN-γ and Granzyme B Secretion by ELISA

Secretion of IFN-γ and granzyme B was quantified using Human IFN-γ DuoSet ELISA and Human Granzyme B DuoSet ELISA (R&D Systems) according to the manufacturer’s instructions. To ensure that sample signals fell within the range of the calibration curve, samples were diluted as follows: 1–10× for IFN-γ, and 2–100× for granzyme B.

### Testing of CD73 Activity

The inhibitory potency of the compounds was evaluated as previously described,^18^ using recombinant human CD73 (rhCD73), recombinant mouse CD73 (rmCD73), and the MDA-MB-231 cell line.

### Selectivity Assays against CD39 and NTPDase3

The cellular assay for assessing the selectivity of CD73 inhibitors toward CD39 has been described previously.^18^

To evaluate the inhibitory activity of CD73 inhibitors against NTPDase3, HEK293T cells were transiently transfected with plasmid DNA encoding ENTPDase3_GFP (CD39L3/ENTPD3 cDNA ORF Clone, C-GFPSpark tag; Sino Biological). Two days prior to the experiment, HEK293T cells were seeded into a 24-well plate to reach 50–60% confluency on the day of transfection. The transfection mixture, consisting of 25 μL of Opti-MEM (Gibco), 0.5 μg of plasmid DNA encoding ENTPDase3_GFP, and 1.5 μL of FuGENE (Promega), was added to each well. Cells were then incubated for 48 h to allow for protein expression.

Following transfection, cells were harvested using 0.25% trypsin, 0.1% EDTA, and ENTPDase3_GFP expression was confirmed by flow cytometry (BD LSRFortessa). Cells were subsequently washed twice with assay buffer (20 mM HEPES, 137 mM NaCl, 5.4 mM KCl, 1.3 mM CaCl_2_, 4.2 mM NaHCO_3_, 0.1% glucose) and counted using an automated cell counter.

For the enzyme activity assay, 500 cells/well were resuspended in 70 μL of assay buffer and seeded into U-bottom 96-well plates. Then, 10 μL/well of each inhibitor (final concentration: 10 μM) was added. After a 1-h incubation at 37 °C, the reaction was initiated by adding 20 μL of ATP (final concentration: 50 μM), followed by a 30 min incubation at room temperature. The plate was centrifuged at 300 × g for 5 min, and 80 μL of supernatant was transferred to a flat-bottom 96-well plate containing 20 μL of PiColorLock Gold mix (Novus Biologicals). After 5 min, 5 μL of stabilizer was added, and absorbance was measured at 635 nm using an Infinite M1000 plate reader (Tecan). Phosphate concentrations were quantified using a standard curve, and conversion rates were calculated for each well. Data were further analyzed using GraphPad Prism 10.

### CellTox Green Cytotoxicity Assay

MDA-MB-231 cells were harvested and counted using an automated cell counter. A total of 2,500 cells per well were seeded in 20 μL of phenol red-free complete RPMI medium into black 384-well plates. Five μL of CD73 inhibitors (final concentration: 50 μM) or 5 μL PBS (negative control) were added to each well, except for wells designed as maximal signal (dead cell) controls. Plates were incubated for 24 h at 37 °C in a humidified CO_2_ incubator. To generate the maximum signal control, 5 μL of lysis buffer (CellTox Green Cytotoxicity Assay, Promega) was added to the dead cell controls, followed by a 30 min incubation at room temperature. Subsequently, 25 μL/well of CellTox Green Dye (diluted 1:500 in the assay buffer; Promega) was added. Plates were incubated in the dark on an orbital shaker at 500–700 rpm for 15 min. Fluorescence was measured using an Infinite M1000 plate reader (Tecan) with excitation at 485–500 nm and emission at 520–530 nm. Data were analyzed in GraphPad Prism 10.

### Molecular Modeling

#### Protein Preparation

The available crystal structures of human CD73 in the closed form with inhibitors were used as templates for structure-based modeling (Protein Data Bank accession codes 6S7H^11^ and 6Z9B^16^). Protein preparation of the 6S7H structure compatible with SQM2.20 scoring was carried out.^28^ Special care was taken to correctly describe the protonation of histidine residues, especially those coordinating the two Zn^2+^ ions (residues 13 and 195 were protonated on the delta nitrogen and residue 218 on the epsilon nitrogen) and bridging the inhibitor beta-phosphate with Asp121 (doubly protonated His 93). Four disulfide bridges were formed between residues 26–32, 328–333, 340–355, and 444–447.

#### Ligand Preparation

To construct starting 3D models of the ligands, the in-house LigandBuilder tool was used. The 3D coordinates of the bisphosphonate-2’-fluoro-arabinose-deazapurine core were taken from the 6Z9B ligand, whereas cores for all the others were taken from the 6S7H ligand. The SMILES codes for the 2- and 6-substituents were then used as inputs to generate their plausible conformations using RDKit.^35^

#### Scoring

A modified SQM2.20 procedure^28^ was applied to all the ligands and their conformations. Specifically, AM1-BCC charges^36^ were used instead of the original PM6 charges due to the presence of the bisphosphonate moiety. The inhibitor conformation for affinity ranking was selected by the total energy of the complex after SQM2.20 optimization.

### *In* *Vivo* Pharmacokinetic Study

The pharmacokinetic study was performed by Bienta (Enamine Biology Services) using male CD-1 mice (3 animals, 9 weeks old, body weight ranging from 29.2 to 33.7 g) following intravenous administration. Levels of the test compound were determined by LC-MS/MS in blood plasma over time (seven sampling time points: 5, 15, 30, 60, 120, 240, and 480 min) after a single dose.

## Supplementary Material



## Data Availability

Data set containing raw data (raw NMR, UPLC and activity assay data) is available at https://doi.org/10.48700/datst.vqbzk-pet80.

## Notes

In vitro ADME data, cytotoxic activities, preparation and characterization of all compounds, copies of ^1^H and ^13^C NMR spectra, flow cytometry gating, selectivity measurements (PDF)

## Abbreviations

Ado: adenosine
CD39: ectonucleoside triphosphate diphosphohydrolase-1
CD73: ecto-5′-nucleotidase
ICI: immune checkpoint inhibitor
TBN: *tert*-butyl nitrite
TMP: trimethyphosphate
TPPTS: triphenylphosphine-3,3′,3″-trisulfonic acid trisodium salt

## References

[1] Noringriis I. M., Donia M., Madsen K., Schmidt H., Haslund C. A., Bastholt L., Svane I. M., Ellebaek E. (2025). Long-Term Clinical
Outcome of Patients with Metastatic Melanoma and Initial Stable Disease
during Anti-PD-1 Checkpoint Inhibitor Immunotherapy with Pembrolizumab. Br. J. Cancer.

[2] Larkin J., Chiarion-Sileni V., Gonzalez R., Grob J.-J., Rutkowski P., Lao C. D., Cowey C. L., Schadendorf D., Wagstaff J., Dummer R., Ferrucci P. F., Smylie M., Hogg D., Hill A., Márquez-Rodas I., Haanen J., Guidoboni M., Maio M., Schöffski P., Carlino M. S., Lebbé C., McArthur G., Ascierto P. A., Daniels G. A., Long G. V., Bastholt L., Rizzo J. I., Balogh A., Moshyk A., Hodi F. S., Wolchok J. D. (2019). Five-Year
Survival with Combined Nivolumab and Ipilimumab in Advanced Melanoma. N. Engl. J. Med..

[3] Boison D., Yegutkin G. G. (2019). Adenosine Metabolism: Emerging Concepts for Cancer
Therapy. Cancer Cell.

[4] Knapp K., Zebisch M., Pippel J., El-Tayeb A., Müller C. E., Sträter N. (2012). Crystal Structure
of the Human Ecto-5′-Nucleotidase
(CD73): Insights into the Regulation of Purinergic Signaling. Structure.

[5] Klemens M. R., Sherman W. R., Holmberg N. J., Ruedi J. M., Low M. G., Thompson L. F. (1990). Characterization of Soluble vs Membrane-Bound
Human
Placental 5′-Nucleotidase. Biochem. Biophys.
Res. Commun..

[6] Synnestvedt K., Furuta G. T., Comerford K. M., Louis N., Karhausen J., Eltzschig H. K., Hansen K. R., Thompson L. F., Colgan S. P. (2002). Ecto-5′-Nucleotidase
(CD73) Regulation by Hypoxia-Inducible Factor-1 Mediates Permeability
Changes in Intestinal Epithelia. J. Clin. Invest..

[7] Niemelä J., Ifergan I., Yegutkin G. G., Jalkanen S., Prat A., Airas L. (2008). IFN-β Regulates
CD73 and Adenosine Expression at the Blood–Brain
Barrier. Eur. J. Immunol..

[8] Xing Y., Ren Z., Jin R., Liu L., Pei J., Yu K. (2022). Therapeutic
Efficacy and Mechanism of CD73-TGFβ Dual-Blockade in a Mouse
Model of Triple-Negative Breast Cancer. Acta
Pharmacol. Sin..

[9] Xia C., Yin S., To K. K. W., Fu L. (2023). CD39/CD73/A2AR Pathway
and Cancer
Immunotherapy. Mol. Cancer.

[10] Bhattarai S., Freundlieb M., Pippel J., Meyer A., Abdelrahman A., Fiene A., Lee S.-Y., Zimmermann H., Yegutkin G. G., Sträter N., El-Tayeb A., Müller C. E. (2015). α,β-Methylene-ADP
(AOPCP) Derivatives and Analogues: Development of Potent and Selective *Ecto* −5′-Nucleotidase (CD73) Inhibitors. J. Med. Chem..

[11] Bhattarai S., Pippel J., Meyer A., Freundlieb M., Schmies C., Abdelrahman A., Fiene A., Lee S., Zimmermann H., El-Tayeb A., Yegutkin G. G., Sträter N., Müller C. E. (2019). X-Ray Co-Crystal Structure Guides the Way to Subnanomolar
Competitive Ecto-5′-Nucleotidase (CD73) Inhibitors for Cancer
Immunotherapy. Adv. Ther..

[12] Bhattarai S., Pippel J., Scaletti E., Idris R., Freundlieb M., Rolshoven G., Renn C., Lee S.-Y., Abdelrahman A., Zimmermann H., El-Tayeb A., Müller C. E., Sträter N. (2020). 2-Substituted α,β-Methylene-ADP Derivatives:
Potent Competitive Ecto-5′-Nucleotidase (CD73) Inhibitors with
Variable Binding Modes. J. Med. Chem..

[13] Junker A., Renn C., Dobelmann C., Namasivayam V., Jain S., Losenkova K., Irjala H., Duca S., Balasubramanian R., Chakraborty S., Börgel F., Zimmermann H., Yegutkin G. G., Müller C. E., Jacobson K. A. (2019). Structure-Activity
Relationship of Purine and Pyrimidine
Nucleotides as Ecto-5′-Nucleotidase (CD73) Inhibitors. J. Med. Chem..

[14] Ge G.-H., Wang Q.-Y., Zhang Z.-H., Zhang X., Guo S., Zhang T.-J., Meng F.-H. (2024). Small Molecular CD73 Inhibitors:
Recent Progress and Future Perspectives. Eur.
J. Med. Chem..

[15] Sharif E. U., Kalisiak J., Lawson K. V., Miles D. H., Newcomb E., Lindsey E. A., Rosen B. R., Debien L. P. P., Chen A., Zhao X., Young S. W., Walker N. P., Sträter N., Scaletti E. R., Jin L., Xu G., Leleti M. R., Powers J. P. (2021). Discovery of Potent and Selective
Methylenephosphonic
Acid CD73 Inhibitors. J. Med. Chem..

[16] Lawson K. V., Kalisiak J., Lindsey E. A., Newcomb E. T., Leleti M. R., Debien L., Rosen B. R., Miles D. H., Sharif E. U., Jeffrey J. L., Tan J. B. L., Chen A., Zhao S., Xu G., Fu L., Jin L., Park T. W., Berry W., Moschütz S., Scaletti E., Sträter N., Walker N. P., Young S. W., Walters M. J., Schindler U., Powers J. P. (2020). Discovery of AB680:
A Potent and Selective Inhibitor
of CD73. J. Med. Chem..

[17] Debien, L. P. P. ; Jaen, J. C. ; Kalisiak, J. ; Lawson, K. V. ; Leleti, M. R. ; Lindsey, E. A. ; Miles, D. H. ; Newcomb, E. ; Powers, J. P. ; Rosen, B. R. ; Sharif, E. U. Modulators of 5 ’ -Nucleotidase, ecto and the use of thereof. U.S. Patent US10981944B2, 2021.

[18] Šímová M., Ormsby T., Šinkevičiu̅tė U., Sirotová Veselovská L., Čermáková K., Hadzima M., Bartoň L., Staňurová J., Kramná A., Šácha P., Tichý M., Hocek M., Konvalinka J., Blazkova K. (2025). Identification of 6-Aryl-7-Deazapurine
Ribonucleoside Phosphonates as Inhibitors of Ecto-5′-Nucleotidase
(CD73). ACS Pharmacol. Transl. Sci..

[19] Malnuit V., Slavětínská L. P., Nauš P., Džubák P., Hajdúch M., Stolaříková J., Snášel J., Pichová I., Hocek M. (2015). 2-Substituted 6-(Het)­Aryl-7-deazapurine
Ribonucleosides: Synthesis, Inhibition of Adenosine Kinases, and Antimycobacterial
Activity. ChemMedChem..

[20] Kim Y. A., Sharon A., Chu C. K., Rais R. H., Al Safarjalani O. N., Naguib F. N. M., El
Kouni M. H. (2008). Structure–Activity Relationships
of 7-Deaza-6-Benzylthioinosine Analogues as Ligands of *Toxoplasma
Gondii* Adenosine Kinase. J. Med. Chem..

[21] Serafinowski P., Dorland E., Balzarini J., De Clercq E. (1995). The Synthesis
and Antiviral Activity of Some New S-Adenosyl-L-Homocysteine Derivatives
and Their Nucleoside Precursors. Nucleosides,
Nucleotides Nucleic Acids.

[22] Eldrup A. B., Prhavc M., Brooks J., Bhat B., Prakash T. P., Song Q., Bera S., Bhat N., Dande P., Cook P. D., Bennett C. F., Carroll S. S., Ball R. G., Bosserman M., Burlein C., Colwell L. F., Fay J. F., Flores O. A., Getty K., LaFemina R. L., Leone J., MacCoss M., McMasters D. R., Tomassini J. E., Von Langen D., Wolanski B., Olsen D. B. (2004). Structure–Activity
Relationship of Heterobase-Modified 2‘- *C* -Methyl
Ribonucleosides as Inhibitors of Hepatitis C Virus RNA Replication. J. Med. Chem..

[23] Seela F., Soulimane T., Mersmann K., Jürgens T. (1990). 2,4-Disubstituted
Pyrrolo­[2,3-*d*]­Pyrimidine α-d- and
ß-d-Ribofuranosides Related to 7-Deazaguanosine. Helv. Chim. Acta.

[24] Nauš P., Perlíková P., Pohl R., Hocek M. (2011). Sugar-Modified
Derivatives of Cytostatic 6-(Het)­Aryl-7-Deazapurine Nucleosides: 2′-*C*-Methylribonucleosides, Arabinonucleosides and 2′-Deoxy-2′-Fluoroarabinonucleosides. Collect. Czech. Chem. Commun..

[25] Colclough N., Ruston L., Wood J. M., MacFaul P. A. (2014). Species Differences
in Drug Plasma Protein Binding. Med. Chem. Commun..

[26] Gerber P.
R., Müller K. (1995). MAB, a Generally
Applicable Molecular Force Field for
Structure Modelling in Medicinal Chemistry. J. Comput.-Aided Mol. Des..

[27] Nocentini A., Capasso C., Supuran C. T. (2021). Small-Molecule CD73 Inhibitors for
the Immunotherapy of Cancer: A Patent and Literature Review (2017–Present). Expert Opin. Ther. Pat..

[28] Pecina A., Fanfrlík J., Lepšík M., Řezáč J. (2024). SQM2.20: Semiempirical
Quantum-Mechanical Scoring Function Yields DFT-Quality Protein–Ligand
Binding Affinity Predictions in Minutes. Nat.
Commun..

[29] Lepsik, M. CD73_inhibitors_SQM; Mendeley Data: V2, 2025. 10.17632/j4rbk4gnr4.2.

[30] Fanfrlík J., Ruiz F. X., Kadlčíková A., Řezáč J., Cousido-Siah A., Mitschler A., Haldar S., Lepšík M., Kolář M. H., Majer P., Podjarny A. D., Hobza P. (2015). The Effect of Halogen-to-Hydrogen Bond Substitution on Human Aldose
Reductase Inhibition. ACS Chem. Biol..

[31] Piovesan D., Tan J. B. L., Becker A., Banuelos J., Narasappa N., DiRenzo D., Zhang K., Chen A., Ginn E., Udyavar A. R., Yin F., Paprcka S. L., Purandare B., Park T. W., Kimura N., Kalisiak J., Young S. W., Powers J. P., Schindler U., Sivick K. E., Walters M. J. (2022). Targeting
CD73 with AB680 (Quemliclustat), a Novel and Potent Small-Molecule
CD73 Inhibitor, Restores Immune Functionality and Facilitates Antitumor
Immunity. Mol. Cancer Ther..

[32] Allard B., Pommey S., Smyth M. J., Stagg J. (2013). Targeting CD73 Enhances
the Antitumor Activity of Anti-PD-1 and Anti-CTLA-4 mAbs. Clin. Cancer Res..

[33] Chen Q., Yin H., He J., Xie Y., Wang W., Xu H., Zhang L., Shi C., Yu J., Wu W., Liu L., Pu N., Lou W. (2023). Tumor Microenvironment
Responsive
CD8+ T Cells and Myeloid-Derived Suppressor Cells to Trigger CD73
Inhibitor AB680-Based Synergistic Therapy for Pancreatic Cancer. Adv. Sci..

[34] Tang T., Huang X., Lu M., Zhang G., Han X., Liang T. (2023). Transcriptional Control of Pancreatic Cancer Immunosuppression by
Metabolic Enzyme CD73 in a Tumor-Autonomous and -Autocrine Manner. Nat. Commun..

[35] RDKit: Open-source cheminformatics. https://www.rdkit.org.

[36] Jakalian A., Jack D. B., Bayly C. I. (2002). Fast, Efficient
Generation of High-quality
Atomic Charges. AM1-BCC Model: II. Parameterization and Validation. J. Comput. Chem..

