# Supplementary material for: Potent Competitive Inhibitors of Ecto-5′-nucleotidase (CD73) based on 6‑(Het)aryl-7-deazapurine Ribonucleoside 5′‑O‑Bisphosphonates
Source: ACS Pharmacol Transl Sci. 2025 Dec 22;9(1):191–213. doi: 10.1021/acsptsci.5c00707 (PMC12797170; doi:10.1021/acsptsci.5c00707)
Supplement: Supplementary file 1 [file pt5c00707_si_001.pdf]

Potent Competitive Inhibitors of Ecto-5´-Nucleotidase (CD73) based on 6-(Het)aryl-7-Deazapurine Ribonucleoside 5'-*O*-Bisphosphonates

Ugnė Šinkevičiūtė#[a,b], Magdalena Šímová#[a], Radek Staník# [a,b], Lenka Poštová Slavětínská[a], Kristýna Blažková[a], Pavel Šácha[a], Martin Lepšík[a], Jan Řezáč[a], Jan Konvalinka[a], Tereza Ormsby*[a], Michal Tichý*[a], and Michal Hocek*[a,b]

[a] Institute of Organic Chemistry and Biochemistry, Czech Academy of Sciences, Flemingovo nam. 2, CZ-16610 Prague 6, Czech Republic.

[b] Department of Organic Chemistry, Faculty of Science, Charles University in Prague, Hlavova 8, CZ-12843 Prague 2, Czech Republic.

# these authors contributed equally

* authors for correspondence: tereza.ormsby@uochb.cas.cz; michal.tichy@uochb.cas.cz; hocek@uochb.cas.cz

Contents

S1. INHIBITION OF HUMAN AND MOUSE CD73 AND CD73 INHIBITION IN MDA-MB-231 CELLS – COMPLETE DATA 3

S2. *IN VITRO* ADME DATA OF FINAL BISPHOSPHONATES 9

S3. INHIBITION OF CYTOCHROME P450 AND HERG 13

S4. SELECTIVITY AGAINST CD39 AND NTPDASE 3 13

S5. *IN VITRO* CYTOTOXIC ACTIVITIES OF FINAL BISPHOSPHONATES 15

S6 GATING FOR FUNCTIONAL ASSAY 18

S7. SQM2.20 SCORES, THEIR TERMS AND EXPERIMENTAL AFFINITIES OF HCD73 LIGANDS 19

S8 ANALYSIS OF UNBOUND PK PROFILES OF COMPOUNDS 7B.28, 18E.6 AND AB680 20

S9 ADDITIONAL SYNTHETIC PROCEDURES AND FULL COMPOUND CHARACTERIZATION 21

S10 HPLC PURITY OF FINAL COMPOUNDS 91

S11 COPIES OF NMR SPECTRA 93

REFERENCES 254

S1. Inhibition of human and mouse CD73 and CD73 inhibition in MDA-MB-231 cells – complete data

Table S1. CD73 inhibition by 2-unsubstituted 7-deazapurine bisphosphonates (**4A1–22**)

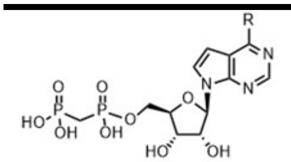	R	K_i (hCD73) [nM]	K_i (mCD73) [nM]	K_i (MDA-MB-231) [nM]
**4A.1**	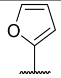	0.41 ± 0.04	3.06 ± 0.09	0.61 ± 0.02
**4A.2**	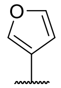	0.80 ± 0.04	3.94 ± 0.33	1.38 ± 0.13
**4A.3**	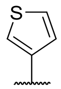	0.27 ± 0.02	2.20 ± 0.06	0.48 ± 0.04
**4A.4**	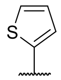	0.26 ± 0.04	1.43 ± 0.28	0.48 ± 0.05
**4A.5**	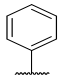	0.61 ± 0.07	6.37 ± 0.34	0.97 ± 0.05
**4A.6**	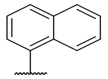	0.03 ± 0.01	3.52 ± 0.39	0.07 ± 0.02
**4A.7**	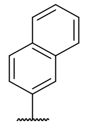	0.17 ± 0.004	3.42 ± 0.20	0.80 ± 0.12
**4A.8**	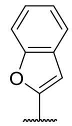	0.15 ± 0.01	3.60 ± 0.10	0.52 ± 0.20
**4A.9**	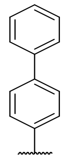	1.60 ± 0.24	12.1 ± 0.8	37 ± 7
**4A.10**	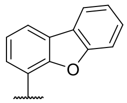	0.41 ± 0.04	12.6 ± 1.9	3.87 ± 0.68
**4A.11**	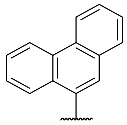	0.30 ± 0.06	47 ± 3	3.57 ± 0.99
**4A.12**	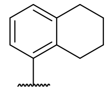	0.010 ± 0.002	1.78 ± 0.12	0.04 ± 0.01
**4A.13**	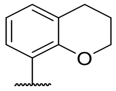	0.50 ± 0.04	8.02 ± 1.21	0.67 ± 0.11

**4A.14**	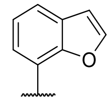	0.16 ± 0.01	3.60 ± 0.20	0.22 ± 0.07
**4A.15**	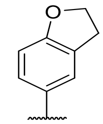	0.38 ± 0.01	4.31 ± 0.11	0.47 ± 0.06
**4A.16**	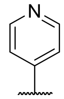	0.73 ± 0.01	8.87 ± 0.49	0.90 ± 0.25
**4A.17**	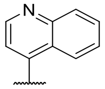	0.04 ± 0.02	8.21 ± 0.48	0.08 ± 0.01
**4A.18**	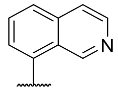	0.06 ± 0.02	6.33 ± 0.40	0.09 ± 0.02
**4A.19**	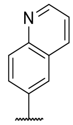	0.55 ± 0.12	9.44 ± 0.51	0.57 ± 0.18
**4A.20**	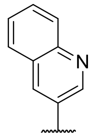	0.51 ± 0.12	7.51 ± 0.05	0.51 ± 0.11
**4A.21**	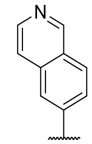	0.27 ± 0.04	4.77 ± 0.18	0.35 ± 0.13
**4A.22**	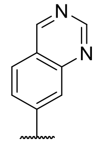	0.39 ± 0.08	5.79 ± 0.68	0.54 ± 0.16

Table S2. CD73 inhibition by 2-substituted 7-deazapurine bisphosphonates **7B, 11C, 14D, 18E**

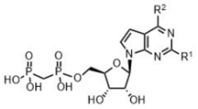	R^2	R^1	K_i (hCD73) [nM]	K_i (mCD73) [nM]	K_i (MDA-MB-231) [nM]
**7B.1**	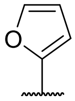	Cl	0.009 ± 0.002	0.28 ± 0.02	0.029 ± 0.01
**11C.1**		NH_2	0.183 ± 0.004	1.24 ± 0.07	0.17 ± 0.04
**14D.1**		F	0.29 ± 0.02	1.06 ± 0.08	0.34 ± 0.09
**18E.1**		Me	0.23 ± 0.02	2.56 ± 0.23	0.27 ± 0.04
**7B.6**	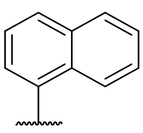	Cl	0.006 ± 0.001	1.89 ± 0.35	0.032 ± 0.001
**11C.6**		NH_2	0.034 ± 0.007	5.31 ± 0.61	0.087 ± 0.013
**14D.6**		F	0.076 ± 0.004	10.0 ± 0.4	0.19 ± 0.05
**18E.6**		Me	0.075 ± 0.013	13.7 ± 0.8	0.16 ± 0.04
**7B.7**	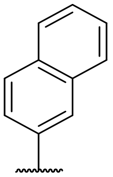	Cl	0.0025 ± 0.0005	0.26 ± 0.02	0.062 ± 0.006
**11C.7**		NH_2	0.049 ± 0.006	1.07 ± 0.10	0.18 ± 0.01
**14D.7**		F	0.086 ± 0.006	1.47 ± 0.18	0.19 ± 0.03
**18E.7**		Me	0.024 ± 0.005	1.43 ± 0.26	0.085 ± 0.004
**7B.8**	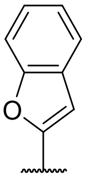	Cl	0.0024 ± 0.001	0.27 ± 0.03	0.044 ± 0.006
**11C.8**		NH_2	0.044 ± 0.006	1.27 ± 0.08	0.088 ± 0.012
**14D.8**		F	0.072 ± 0.012	1.10 ± 0.20	0.15 ± 0.02
**18E.8**		Me	0.020 ± 0.002	1.50 ± 0.26	0.079 ± 0.004
**7B.12**	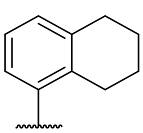	Cl	0.003 ± 0.001	0.94 ± 0.17	0.025 ± 0.001
**11C.12**		NH_2	0.010 ± 0.002	1.41 ± 0.11	0.025 ± 0.006
**14D.12**		F	0.07 ± 0.03	6.15 ± 0.81	0.11 ± 0.02
**18E.12**		Me	0.031 ± 0.006	8.26 ± 0.39	0.069 ± 0.013

Table S3. CD73 inhibition by 2-substituted 6-(het)aryl 7-deazapurine bisphosphonates (**21F.7, 22G.7, 27H.23**)

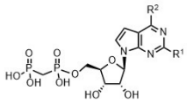	R^2	R^1	K_i (hCD73) [nM]	K_i (mCD73) [nM]	K_i (MDA-MB-231) [nM]
**21F.7**	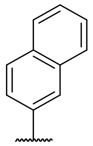	–OMe	0.18 ± 0.01	2.78 ± 0.22	0.56 ± 0.14
**22G.7**		–NHMe	0.54 ± 0.02	14 ± 2	1.59 ± 0.43
**27H.23**	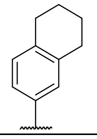	–I	0.0018 ± 0.0006	0.32 ± 0.05	0.088 ± 0.04

Table S4. CD73 inhibition by 2-chloro substituted 6-(het)aryl 7-deazapurine bisphosphonates (**7B.23–35**)

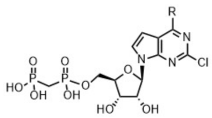	R	K_i (hCD73) [nM]	K_i (mCD73) [nM]	K_i (MDA-MB-231) [nM]
**7B.23**	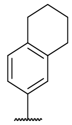	0.0013 ± 0.0001	0.22 ± 0.02	0.063 ± 0.01
**7B.24**	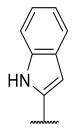	0.004 ± 0.001	0.30 ± 0.02	0.023 ± 0.006
**7B.25**	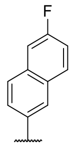	0.007 ± 0.004	0.36 ± 0.04	0.049 ± 0.01
**7B.26**	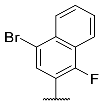	0.0039 ± 0.003	0.53 ± 0.04	0.077 ± 0.010
**7B.27**	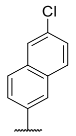	0.007 ± 0.001	0.59 ± 0.06	0.16 ± 0.05
**7B.28**	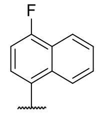	0.00076 ± 0.00038	0.64 ± 0.07	0.0091 ± 0.0016
**7B.29**	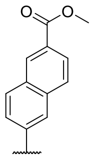	0.023 ± 0.003	0.63 ± 0.02	0.33 ± 0.07
**7B.30**	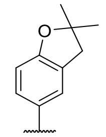	0.004 ± 0.002	0.39 ± 0.05	0.034 ± 0.010
**7B.31**	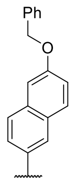	0.054 ± 0.020	2.89 ± 0.60	1.17 ± 0.17
**7B.32**	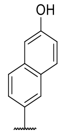	0.0029 ± 0.0002	0.293 ± 0.002	0.022 ± 0.002

**7B.33**	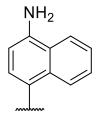	0.053 ± 0.019	5.36 ± 0.67	0.050 ± 0.004
**7B.34**	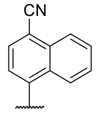	0.045 ± 0.014	8.55 ± 1.16	0.063 ± 0.011
**7B.35**	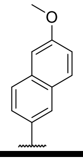	0.007 ± 0.01	0.28 ± 0.08	0.077 ± 0.009

Table S5. CD73 inhibition of C-2 unsubstituted purine bisphosphonates (**31I.6,7,12**)

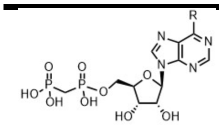	R	K_i (hCD73) [nM]	K_i (mCD73) [nM]	K_i (MDA-MB-231) [nM]
**31A.6**	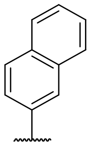	0.77 ± 0.06	11.4 ± 0.8	3.10 ± 0.61
**31A.7**	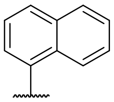	3.12 ± 0.26	112 ± 6	3.57 ± 1.15
**31A.12**	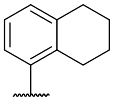	2.70 ± 0.10	62 ± 2	3.01 ± 0.70

Table S6. CD73 inhibition by 6-(het)aryl 7-deazapurine 2´-fluoroarabino nucleoside 5´-*O*-bisphosphonates (**35A.5-7,12,17,23,28**)

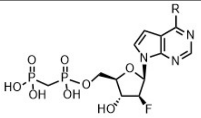	R	K_i (hCD73) [nM]	K_i (mCD73) [nM]	K_i (MDA-MB-231) [nM]
**34A.5**	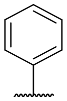	7.07	56.87	6.80
**34A.6**	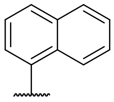	2.48	36.29	7.35

**34A.7**	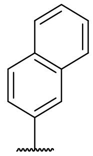	2.42	24.38	22.47
**34A.12**	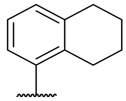	1.06	12.90	5.07
**34A.17**	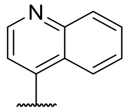	3.16	50.39	3.66
**34A.23**	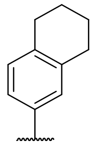	1.61	11.83	23.25
**34A.28**	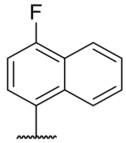	0.80	14.81	3.97

S2. *In vitro* ADME data of final bisphosphonates

Table S7. *In vitro* ADME data of final bisphosphonates **4A.1–22**

Comp	Solubility in PBS [µM]	Plasma stab. (hu) $t_{1/2}$ [min]	Plasma stab. (m) $t_{1/2}$ [min]	Mics (hu) comp remaining 60 min [%]	Mics (hu) CL_{int} [µL/min/mg]	Mics (m) comp remaining 60 min [%]	Mics (m) CL_{int} [µL/min/mg]	PPB (hu) [%]	PPB (m) [%]
**4A.1**	25	>120	>120	nd	nd	nd	nd	94.7	30.1
**4A.2**	97	>120	>120	nd	nd	nd	nd	83.8	12.8
**4A.3**	76	>120	>120	nd	nd	nd	nd	91.9	46.5
**4A.4**	90	>120	>120	nd	nd	nd	nd	87.5	27.4
**4A.5**	80	>120	>120	nd	nd	nd	nd	90.5	21.2
**4A.6**	62	>120	>120	nd	nd	nd	nd	91.4	37.4
**4A.7**	>100	>120	>120	nd	nd	nd	nd	98.9	68.5
**4A.8**	98	>120	>120	nd	nd	nd	nd	98.1	62.9
**4A.9**	64	>120	>120	nd	nd	nd	nd	98.9	85.6
**4A.10**	>100	>120	>120	nd	nd	nd	nd	99.6	86.5
**4A.11**	68	>120	>120	nd	nd	nd	nd	98.7	77.8
**4A.12**	94	>120	>120	96	1	100	<1	92.7	61.9
**4A.13**	81	>120	>120	96	1	94	1	89.2	40.6
**4A.14**	>100	>120	>120	112	<1	94	3	93.8	45.3
**4A.15**	76	>120	>120	118	<1	102	<1	96.4	45.8
**4A.16**	79	>120	>120	94	3	80	9	69.6	16.7
**4A.17**	>100	>120	>120	104	<1	79	9	74.3	20.0
**4A.18**	56	>120	>120	105	<1	102	<1	80.5	27.3
**4A.19**	85	>120	>120	94	<1	105	<1	95.2	31.5
**4A.20**	>100	>120	>120	88	2	110	<1	94.6	46.4
**4A.21**	>100	>120	>120	95	3	79	8	94.6	26.3
**4A.22**	66	>120	>120	92	4	101	<1	88.7	5.2

Mics – microsomal stability, CL_{int} – microsomal intrinsic clearance, hu – human, m – mouse, PPB – plasma protein binding; nd – not determined, microsomal stability was measured at 3 time points (10, 30, 45 min) and measured half-life is >>45 min.

Table S8. *In vitro* ADME data of final bisphosphonates **7B.1,6–8,12; 11C.1,6–8,12; 14D.1,6–8,12; 18E.1,6–8,12**

Comp	Solubility in PBS [µM]	Plasma stab. (hu) $t_{1/2}$ [min]	Plasma stab. (m) $t_{1/2}$ [min]	Mics (hu) comp remaining 60 min [%]	Mics (hu) CL_{int} [µL/min/mg]	Mics (m) comp remaining 60 min [%]	Mics (m) CL_{int} [µL/min/mg]	PPB (hu) [%]	PPB (m) [%]
**7B.1**	>100	>120	>120	98	2	106	<1	90.2	18.2
**11C.1**	>100	>120	>120	107	<1	103	<1	77.6	37.8
**14D.1**	80	>120	>120	103	<1	96	1	95.5	33.6
**18E.1**	89	>120	>120	100	<1	96	1	93.1	18.4
**7B.6**	92	>120	>120	96	1	94	2	96.6	72.6
**11C.6**	>100	>120	>120	92	3	102	<1	93.3	43.6
**14D.6**	89	>120	>120	100	<1	111	<1	96.7	58.0
**18E.6**	89	>120	>120	98	1	95	1	95.5	48.5
**7B.7**	84	>120	>120	111	<1	106	<1	96.7	88.8
**11C.7**	>100	>120	>120	100	<1	97	2	96.3	69.5
**14D.7**	85	>120	>120	112	<1	105	<1	97.6	81.7
**18E.7**	78	>120	>120	86	5	97	1	99.6	77.9
**7B.8**	87	>120	>120	98	1	109	<1	96.7	81.3
**11C.8**	87	>120	>120	100	<1	97	2	94.2	60.9
**14D.8**	76	>120	>120	107	<1	104	<1	99.2	71.4
**18E.8**	86	>120	>120	95	2	103	<1	97.4	69.8
**7B.12**	85	>120	>120	101	<1	117	<1	99.5	78.4
**11C.12**	88	>120	>120	101	<1	100	<1	84.3	55.5
**14D.12**	84	>120	>120	99	<1	116	<1	97.7	76.5
**18E.12**	80	>120	>120	108	<1	106	<1	97.8	67.7

Mics – microsomal stability, CL_{int} – microsomal intrinsic clearance, hu – human, m – mouse, PPB – plasma protein binding

Table S9. *In vitro* ADME data of final bisphosphonates **7B.23–35**

Comp	Solubility in PBS [µM]	Plasma stab. (hu) $t_{1/2}$ [min]	Plasma stab. (m) $t_{1/2}$ [min]	Mics (hu) comp remaining 60 min [%]	Mics (hu) CL_{int} [µL/min/mg]	Mics (m) comp remaining 60 min [%]	Mics (m) CL_{int} [µL/min/mg]	PPB (hu) [%]	PPB (m) [%]
**7B.23**	84	>120	>120	124	<1	123	<1	≥99	94.1
**7B.24**	>100	>120	>120	111	<1	72	12	98.8	79.9
**7B.25**	>100	>120	>120	108	<2	109	<2	≥99	92.1
**7B.26**	71	>120	>120	117	<2	113	<2	≥99	98.4
**7B.27**	89	>120	>120	148	<2	140	<2	≥99	96.4
**7B.28**	>100	>120	>120	95	2	101	<2	≥99	76.8
**7B.29**	85	>120	>120	132	<2	112	<2	≥99	76.6
**7B.30**	86	>120	>120	102	<1	88	6	≥99	77.1
**7B.31**	>100	>120	>120	147	<2	134	<2	≥99	≥99
**7B.32**	>100	>120	>120	104	<2	111	<2	91.6	80.2
**7B.33**	>100	>120	>120	109	<2	103	<2	≥99	62.6
**7B.34**	>100	>120	>120	97	<2	103	<2	≥99	66.1
**7B.35**	84	>120	>120	106	<2	103	<2	≥99	91.2
**AB680**	76	>120	>120	118	<2	117	<2	≥99	90.2

Mics – microsomal stability, CL_{int} – microsomal intrinsic clearance, hu – human, m – mouse, PPB – plasma protein binding

Table S10. *In vitro* ADME data of final bisphosphonates **21F.7; 21G.7; 27H.23; 31A.6,7,12; 34A.5–7,12,17,23,28**

Comp	Solubility in PBS [μM]	Plasma stab. (hu) $t_{1/2}$ [min]	Plasma stab. (m) $t_{1/2}$ [min]	Mics (hu) comp remaining 60 min [%]	Mics (hu) CL_{int} [μL/min/mg]	Mics (m) comp remaining 60 min [%]	Mics (m) CL_{int} [μL/min/mg]	PPB (hu) [%]	PPB (m) [%]
**21F.7**	79	>120	>120	104	<1	nd	<1	≥99	86.4
**22F.7**	84	>120	>120	106	<1	nd	3	≥99	88.8
**27H.23**	>100	>120	>120	104	<1	nd	2	≥99	96.4
**31A.6**	87	>120	>120	121	<1	108	<1	90.7	37.2
**31A.7**	86	>120	>120	106	<1	87	6	≥99	77.6
**31A.12**	80	>120	>120	102	<1	88	6	91.6	49.1
**34A.5**	245	>120	>120	93	nd	97	nd	97.8	80
**34A.6**	167	>120	>120	97	nd	94	nd	98.3	93.5
**34A.7**	82	>120	>120	94	nd	90	nd	99.8	97.8
**34A.12**	75	>120	>120	96	nd	115	nd	99.3	94.5
**34A.17**	73	>120	>120	90	nd	89	nd	95.0	74.7
**34A.23**	100	>120	>120	98	nd	116	nd	99.9	98.3
**34A.28**	100	>120	>120	102	nd	101	nd	98.6	94.2

Mics – microsomal stability, CL_{int} – microsomal intrinsic clearance, hu – human, m – mouse, PPB – plasma protein bindingy nd – not determined, microsomal stability was measured at 3 time points (10, 30, 45 min) and measured half-life is >>45 min.

S3. Inhibition of Cytochrome P450 and hERG

The tested compounds did not show any significant inhibition of CYP450 activity at a concentration of 10 μM. Similarly, they did not show any inhibition of the hERG potassium channel even at 100 μM.

Table S11. Inhibition of CYP450 and hERG

	CYP isoforms, % inhibition at 10 μM					hERG
Compound	1A2	2D6	2C9	2C19	3A4	% inhibition at 100 μM
**4A.7**	7	–17	23	10	–7	1
**4A.18**	7	–10	11	3	–3	6
**7B.12**	0	–13	15	10	–8	3
**7B.23**	–17	6	21	3	–23	5
**7B.28**	9	–6	24	22	9	6
**18E.6**	8	–18	12	–20	0	2
**27H.23**	–4	–12	11	–7	–23	7

S4. Selectivity against CD39 and NTPDase 3

HEK cells expressing CD39 (293T-CD39-27) or transiently transfected with NTPDase3 were incubated with a dilution series of the tested compounds. Enzymatic activity of CD39 or NTPDase3 was measured using the PiColorLock Gold assay. Data were analyzed by non-linear regression in GraphPad Prism and represent the mean of technical duplicates. CD39 inhibition was assessed for all developed compounds with the same results; NTPDase3 inhibition was evaluated on the selected compounds listed in Table S11.

Table S12. Inhibition of NTPDase3 by selected compounds.

Compound	**NTPDase3-HEK IC_{50} [μM]**
**7B.6**	>10
**7B.12**	>10
**7B.23**	>10
**7B.28**	>10
**18E.6**	>10
**18E.7**	>10
**27H.23**	>10

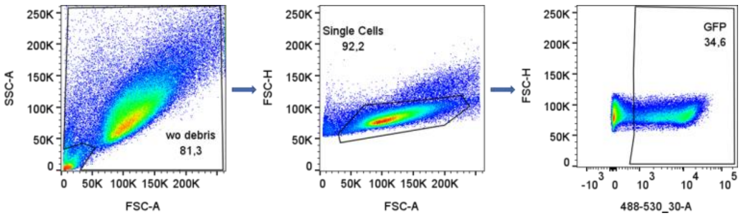

Figure S1: Expression of NTPDase 3 in transiently transfected HEK293T. Debris was excluded using FSC-A/SSC-A, followed by doublet discrimination using FSC-A/FSC-H parameters. NTPDase3-GFP expression was analyzed using a BD LSRFortessa™ flow cytometer and FlowJo software.

S5. *In vitro* Cytotoxic Activities of Final Bisphosphonates

Table S13. Cytotoxic Activities of Final Bisphosphonates

	CCRF-CEM IC_{50} [μM]	HepG2 IC_{50} [μM]	Hela S3 IC_{50} [μM]	HL-60 IC_{50} [μM]	NHDF IC_{50} [μM]
**4A.1**	>10	>10	>10	3.7	>10
**4A.2**	>10	>10	>10	2.8	>10
**4A.3**	9.9	>10	>10	4.2	>10
**4A.4**	9.7	>10	>10	3.4	>10
**4A.5**	>10	>10	>10	3.7	>10
**4A.6**	>10	>10	>10	>10	>10
**4A.7**	>10	>10	>10	>10	>10
**4A.8**	>10	>10	>10	>10	>10
**4A.9**	>10	>10	>10	>10	>10
**4A.10**	>10	>10	>10	9.5	>10
**4A.11**	>10	>10	>10	>10	>10
**4A.12**	>10	>10	>10	>10	>10
**4A.13**	>10	>10	>10	>10	>10
**4A.14**	>10	6.3	>10	6.6	5.6
**4A.15**	>10	8.7	>10	8.6	5.7
**4A.16**	>10	>10	>10	>10	>10
**4A.17**	>10	>10	>10	>10	>10
**4A.18**	>10	>10	>10	>10	>10
**4A.19**	>10	>10	>10	3.1	>10
**4A.20**	>10	>10	>10	>10	>10

Continuation of Table S13

	CCRF-CEM IC_{50} [μM]	HepG2 IC_{50} [μM]	Hela S3 IC_{50} [μM]	HL-60 IC_{50} [μM]	NHDF IC_{50} [μM]
**4A.21**	>10	>10	>10	5.9	>10
**4A.22**	9.9	>10	>10	4.8	>10
**7B.1**	8.8	>10	>10	5.6	>10
**7B.6**	>10	>10	>10	2.9	>10
**7B.7**	>10	>10	>10	>10	>10
**7B.8**	>10	>10	>10	6.0	>10
**7B.12**	>10	>10	>10	>10	>10
**7B.23**	>10	9.3	>10	5.7	>10
**7B.24**	>10	>10	>10	>10	>10
**7B.25**	>10	>10	>10	>10	>10
**7B.26**	>10	>10	>10	>10	>10
**7B.27**	>10	>10	>10	>10	>10
**7B.28**	>10	>10	>10	>10	>10
**7B.29**	>10	>10	>10	>10	>10
**7B.30**	>10	>10	>10	>10	>10
**7B.31**	>10	>10	>10	>10	>10
**7B.32**	>10	>10	>10	>10	>10
**7B.33**	9.6	7.9	8.7	9.1	8.8
**7B.34**	>10	>10	>10	>10	>10
**7B.35**	>10	>10	>10	>10	>10
**11C.1**	>10	>10	>10	>10	>10
**11C.6**	>10	>10	>10	>10	>10
**11C.7**	>10	>10	>10	>10	>10
**11C.8**	>10	>10	>10	>10	>10
**11C.12**	>10	>10	>10	>10	>10
**14D.1**	>10	>10	>10	>10	>10
**14D.6**	>10	>10	>10	>10	>10
**14D.7**	>10	>10	>10	>10	>10
**14D.8**	>10	>10	>10	>10	>10
**14D.12**	>10	>10	>10	>10	>10
**18E.1**	>10	>10	>10	>10	>10
**18E.6**	>10	>10	>10	>10	>10
**18E.7**	>10	>10	>10	>10	>10
**18E.8**	>10	>10	>10	>10	>10
**18E.12**	>10	>10	>10	>10	>10
**21F.7**	>10	>10	>10	>10	>10
**22G.7**	>10	>10	>10	8.1	>10
**27H.23**	>10	>10	>10	>10	>10
**31A.6**	>10	>10	>10	>10	>10
**31A.7**	>10	4.8	>10	7.6	3.7
**31A.12**	>10	>10	>10	>10	>10

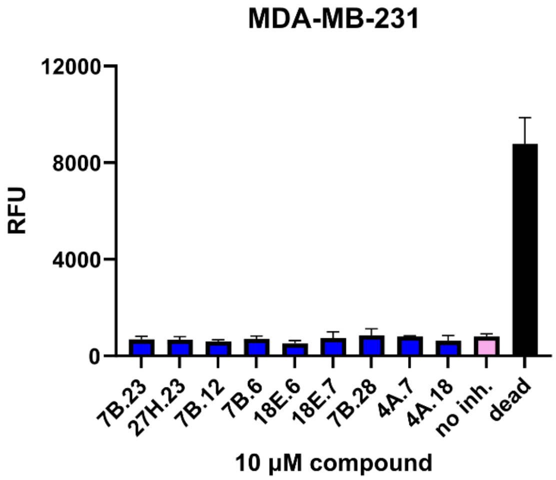

Figure S2: Effect of CD73 inhibition on the viability of CD73-expressing breast cancer cell line. MDA-MB-231 cells were incubated with 10 µM CD73 inhibitors for 24 hours. Viability was assessed using the CellTox™ Green Cytotoxicity Assay. Displayed data represent the mean ± s.d. of technical triplicates (s.d., standard deviation).

S6 Gating for Functional Assay

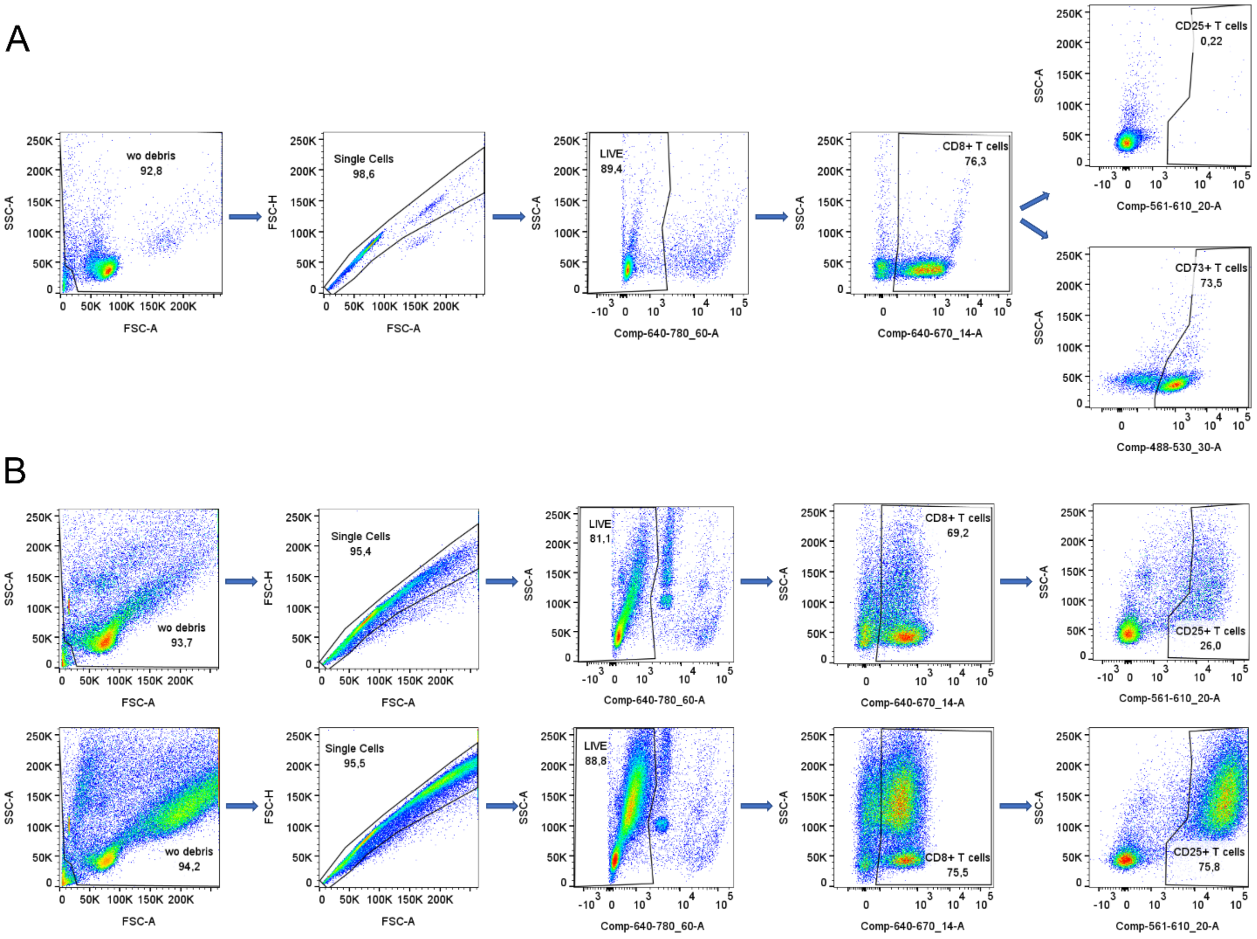

Figure S3: Immunophenotyping and gating strategy for the T cell activation assay. For all gating strategies, debris was excluded using FSC-A/SSC-A, followed by doublet discrimination using FSC-A/FSC-H parameters. **A.** Immunophenotyping of isolated, non-activated primary human CD8+ T cells. Live CD8+ T cells were stained with anti-CD25 and anti-CD73 antibodies. **B.** Gating strategy for the T cell activation assay. CD8+ T cells were stimulated with CD3/CD28 activation beads, in the presence of EHNA, and in the presence (upper panel) or absence (lower panel) of AMP. Live CD8+ T cells were stained using an anti-CD25 antibody to assess activation status. All samples were analyzed using a BD LSRFortessa™ flow cytometer and FlowJo software.

S7. SQM2.20 scores, their terms and experimental affinities of hCD73 ligands

Tab. S14. Experimental affinities as Gibbs free energies (dG_exp) and SQM2.20 scores and their terms (kcal/mol) of sixty-seven CD73 ligands modeled.

Code	dG_exp	score_shift_scale	score_sqm	Int_e	Int_e_vac	Solv_e_int
**4A.8**	–13.5	–12.3	–85.1	–90.3	–598.5	508.2
**34A.6**	–11.8	–12.1	–84.6	–84.0	–576.5	492.4
**34A.7**	–11.8	–8.6	–77.3	–77.0	–570.3	493.2
**34A.17**	–11.8	–12.1	–84.8	–82.9	–568.6	485.7
**34A.28**	–12.5	–11.9	–84.4	–83.4	–574.8	491.4
**34A.23**	–12.3	–10.4	–81.2	–80.2	–579.7	499.4
**34A.5**	–11.2	–7.6	–75.2	–74.0	–568.6	494.6
**34A.12**	–12.4	–12.1	–84.8	–83.3	–573.1	489.8
**4A.3**	–13.1	–12.9	–86.5	–90.3	–597.6	507.3
**4A.7**	–13.4	–12.9	–86.5	–91.9	–600.4	508.6
**4A.10**	–12.9	–14.5	–89.8	–95.3	–605.9	510.6
**4A.4**	–13.2	–12.8	–86.2	–91.2	–598.7	507.5
**4A.1**	–12.9	–11.6	–83.7	–88.3	–599.9	511.6
**4A.5**	–12.6	–12.8	–86.2	–90.3	–598.9	508.5
**4A.9**	–12.1	–15.1	–91.1	–94.8	–603.7	508.8
**4A.2**	–12.5	–10.4	–81.1	–86.3	–597.4	511.1
**4A.6**	–14.4	–15.2	–91.4	–95.5	–602.1	506.7
**4A.11**	–13.1	–15.8	–92.6	–96.8	–607.8	510.9
**4A.17**	–14.3	–15.2	–91.4	–94.4	–601.9	507.6
**4A.18**	–14.1	–13.1	–86.9	–90.9	–608.2	517.3
**4A.19**	–12.7	–13.6	–87.9	–91.7	–599.6	507.9
**4A.20**	–12.8	–13.2	–87.0	–92.0	–598.9	506.9
**4A.22**	–12.9	–12.5	–85.6	–89.3	–599.8	510.5
**4A.21**	–13.1	–13.1	–86.8	–91.8	–602.4	510.5
**7B.1**	–15.2	–13.1	–87.0	–91.8	–600.1	508.3
**4A.12**	–15.1	–16.5	–94.2	–97.5	–605.8	508.2
**7B.6**	–15.4	–16.2	–93.6	–95.8	–602.0	506.1
**7B.8**	–16.0	–15.6	–92.3	–96.4	–603.1	506.7
**7B.7**	–15.9	–15.1	–91.1	–95.2	–600.8	505.6
**11C.6**	–13.9	–13.3	–87.4	–90.8	–609.1	518.4
**11C.8**	–13.9	–13.3	–87.3	–92.6	–612.1	519.4
**11C.7**	–13.8	–13.1	–86.8	–91.2	–610.4	519.2
**14D.6**	–14.4	–14.0	–88.8	–93.6	–599.5	505.9
**14D.7**	–14.2	–12.8	–86.3	–91.4	–597.0	505.7
**18E.6**	–13.9	–13.9	–88.6	–94.9	–611.4	516.5
**4A.13**	–12.8	–14.0	–88.9	–91.7	–605.1	513.4
**18E.8**	–14.7	–14.6	–90.1	–94.4	–608.4	514.0
**11C.12**	–13.9	–14.9	–90.8	–93.4	–611.9	518.5
**18E.1**	–13.2	–11.9	–84.3	–89.9	–606.2	516.3
**14D.1**	–13.4	–11.3	–83.0	–87.2	–595.4	508.2

**14D.8**	–14.3	–13.8	–88.4	–92.8	–599.0	506.3
**18E.12**	–14.4	–15.6	–92.2	–96.4	–609.9	513.5
**18E.7**	–14.6	–14.2	–89.3	–93.7	–608.7	515.0
**7B.12**	–15.9	–16.2	–93.6	–97.7	–605.4	507.7
**11C.1**	–13.1	–11.4	–83.2	–87.1	–608.2	521.1
**14D.12**	–15.1	–15.0	–91.0	–95.7	–602.5	506.8
**21F.7**	–13.4	–12.8	–86.3	–92.1	–603.8	511.6
**22G.7**	–12.7	–15.0	–90.9	–92.5	–609.9	517.4
**4A.16**	–12.5	–13.2	–87.0	–90.1	–594.3	504.2
**4A.14**	–12.9	–13.7	–88.2	–92.4	–604.9	512.5
**7B.23**	–16.3	–17.1	–95.4	–98.8	–607.4	508.6
**4A.15**	–13.4	–15.7	–92.5	–93.8	–607.3	513.5
**31A.7**	–12.5	–13.2	–87.0	–93.5	–601.3	507.9
**31A.6**	–11.7	–15.0	–90.8	–94.5	–602.8	508.2
**31A.12**	–11.8	–11.3	–83.1	–95.9	–606.3	510.4
**7B.30**	–15.6	–15.2	–91.3	–95.6	–604.8	509.2
**7B.24**	–15.7	–15.4	–91.7	–94.8	–599.7	505.0
**27H.23**	–16.1	–16.9	–94.9	–98.3	–608.8	510.5
**7B.25**	–15.4	–14.4	–89.7	–94.1	–599.1	505.0
**7B.26**	–15.7	–16.2	–93.5	–98.7	–604.8	506.1
**7B.27**	–15.3	–14.5	–90.0	–94.6	–602.3	507.6
**7B.28**	–16.6	–15.2	–91.5	–96.0	–599.5	503.5
**7B.29**	–14.6	–15.9	–92.9	–98.9	–609.4	510.5
**7B.31**	–14.1	–14.9	–90.8	–94.7	–602.7	507.9
**7B.32**	–15.8	–16.4	–94.0	–97.1	–611.0	513.9
**7B.33**	–14.1	–14.4	–89.7	–94.4	–610.4	516.0
**7B.35**	–15.4	–14.8	–90.5	–95.0	–602.5	507.4

S8 Analysis of unbound PK profiles of compounds 7B.28, 18E.6 and AB680

We used mouse *in vivo* PK data to calculate the unbound fraction using mouse PPB and plotted that against mouse CD73 K_i values. The graph clearly shows that exposure remains well above Ki at all time points for all three compounds (Fig S1).

Figure S1. Unbound PK profiles of compounds **7B.28**, **18E.6** and **AB680**

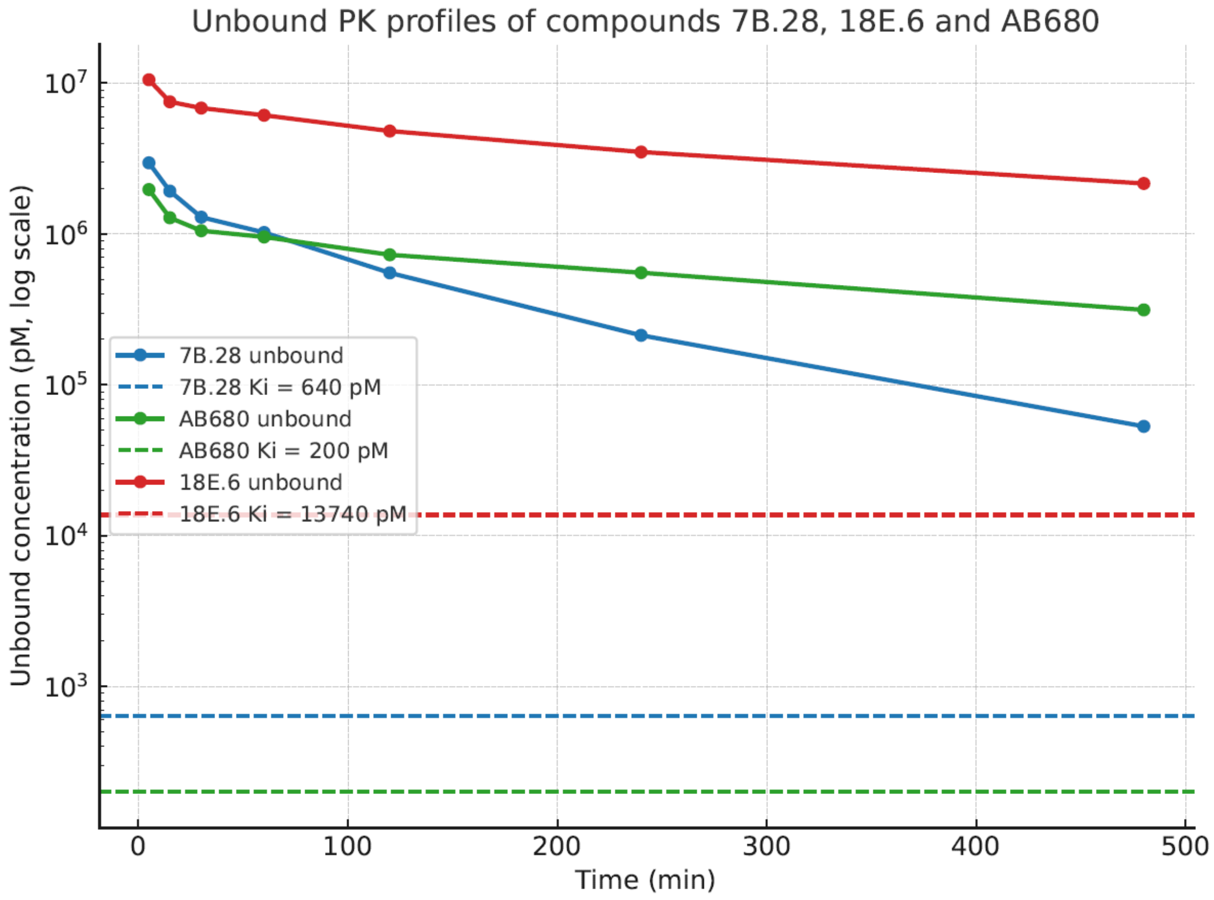

S9 Additional Synthetic Procedures and Full Compound Characterization

[(5-{[4-(Furan-3-yl)-7*H*-pyrrolo[2,3-*d*]pyrimidin-7-yl]-*β*-D-ribofuranosyl}oxy)phosphonomethyl]phosphonic acid (4A.2)

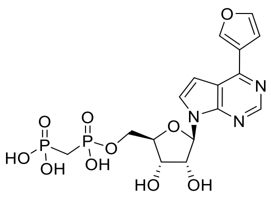

Compound **3** (68.9 mg, 0.16 mmol) was reacted with furan-3-ylboronic acid (26.1 mg, 0.23 mmol) for 5 min at 80 °C and 1 h at 65 °C according to the GP A. 2 HPLC purifications (C-18, H_2O + 0.05 % TFA/MeCN 0 → 80 %) gave product **4A.2** (38.9 mg, 53 %) as a white powder. ^{1}H NMR (500 MHz, DMSO-d$_6$): 2.26 (t, 2H, $J_{CH2,P}$ = 20.4 Hz, PCH$_2$P); 4.06–4.17 (m, 3H, H-4´,5´); 4.21 (dd, 1H, $J_{3´,2´}$ = 5.1 Hz, $J_{3´,4´}$ = 3.0 Hz, H-3´); 4.47 (dd, 1H, $J_{2´,1´}$ = 6.2 Hz, $J_{2´,3´}$ = 5.1 Hz, H-2´); 6.29 (d, 1H, $J_{1´,2´}$ = 6.2 Hz, H-1´); 7.10 (d, 1H, $J_{5,6}$ = 3.9 Hz, H-5); 7.27 (dd, 1H, $J_{4,5}$ =

1.9 Hz, $J_{4,2}$ = 0.6 Hz, H-4-furyl); 7.90 (t, 1H, $J_{5,4}$ = $J_{5,2}$ = 1.7 Hz, H-5-furyl); 7.92 (d, 1H, $J_{6,5}$ = 3.9 Hz, H-6); 8.74 (bs, 1H, H-2-furyl); 8.80 (s, 1H, H-2); ^{13}C NMR (125.7 MHz, DMSO-d_6): 27.5 (bt, $J_{C,P}$ = 128.5 Hz, PCH_2P); 64.8 (d, $J_{C,P}$ = 5.3 Hz, CH_2-5´); 70.4 (CH-3´); 73.7 (CH-2´); 82.9 (d, $J_{C,P}$ = 7.4 Hz, CH-4´); 86.3 (CH-1´); 101.1 (CH-5); 109.4 (CH-4-furyl); 114.3 (C-4a); 124.9 (C-3-furyl); 127.4 (CH-6); 144.6 and 144.9 (CH-2,5-furyl); 149.9 (C-4); 151.0 (CH-2); 151.8 (C-7a); ^{31}P NMR (202.4 MHz, DMSO-d_6): 15.80 and 19.67 (2×d, 2×1P, $J_{P,P}$ = 7.1 Hz, PCH_2P). HR-ESI-MS: *found*: 474.0470 ([M–H]$^-$, calcd for $C_{16}H_{18}O_{10}N_3P_2^-$: 474.0473).

[(5-{[4-(Thiophen-2-yl)-7*H*-pyrrolo[2,3-*d*]pyrimidin-7-yl]-*β*-D-ribofuranosyl}oxy)phosphonomethyl]phosphonic acid (4A.3)

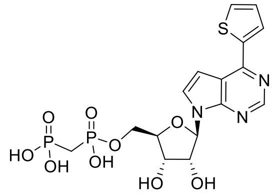

Compound **3** (52.2 mg, 0.12 mmol) was reacted with thiophen-2-ylboronic acid (22.6 mg, 0.18 mmol) for 5 min at 80 °C and 1 h at 65 °C according to the GP A. RP-HPFC (C-18, H_2O/MeOH 0 → 100 %), HPLC (C-18, H_2O + 0.05 % TFA/MeCN 0 → 80 %) gave product **4A.3** (33.2 mg, 57 %) as a pale-yellow powder. ^{1}H NMR (500 MHz, DMSO-d_6): 2.27 (t, 2H, $J_{CH2,P}$ = 20.1 Hz, PCH_2P); 4.05–4.17 (m, 3H, H-4´,5´); 4.21 (m, 1H, H-3´); 4.47 (t, 1H, $J_{2',1'}$ = $J_{2',3'}$ = 5.6 Hz, H-2´); 6.30 (d, 1H, $J_{1',2'}$ = 6.2 Hz, H-1´); 7.17 (d, 1H, $J_{5,6}$ = 3.8 Hz, H-5); 7.31 (dd, 1H, $J_{4,5}$ = 5.0 Hz, $J_{4,3}$ = 3.8 Hz, H-4-thienyl); 7.86 (dd, 1H, $J_{5,4}$ = 5.0 Hz, $J_{5,3}$ = 1.0 Hz, H-5-thienyl); 7.96 (d, 1H, $J_{6,5}$ = 3.8 Hz, H-6); 8.17 (dd, 1H, $J_{3,4}$ = 3.8 Hz, $J_{3,5}$ = 1.0 Hz, H-3-thienyl); 8.76 (s, 1H, H-2); ^{13}C NMR (125.7 MHz, DMSO-d_6): 64.8 (bd, $J_{C,P}$ = 3.4 Hz, CH_2-5´); 70.3 (CH-3´); 73.7 (CH-2´); 82.9 (d, $J_{C,P}$ = 5.6 Hz, CH-4´); 86.3 (CH-1´); 101.1 (CH-5); 112.9 (C-4a); 128.1 (CH-6); 129.1 (CH-4-thienyl); 129.6 (CH-3-thienyl); 130.8 (CH-5-thienyl); 142.4 (C-2-thienyl); 150.1 (C-4); 150.9 (CH-2); 152.3 (C-7a). *Carbon of PCH_2P was not detected*; ^{31}P NMR (202.4 MHz, DMSO-d_6): 18.15 and 21.91 (2×bs, 2×1P, PCH_2P). HR-ESI-MS: *found*: 490.0238 ([M–H]$^-$, calcd for $C_1H_{18}O_9N_3P_2S^-$: 490.0234).

[(5-{[4-(Thiophen-3-yl)-7*H*-pyrrolo[2,3-*d*]pyrimidin-7-yl]-*β*-D-ribofuranosyl}oxy)phosphonomethyl]phosphonic acid (4A.4)

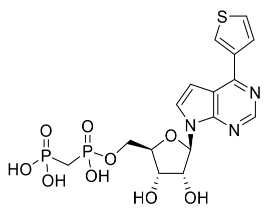

Compound **3** (52.2 mg, 0.12 mmol) was reacted with thiophen-3-ylboronic acid (22.6 mg, 0.18 mmol) for 5 min at 80 °C and 1 h at 65 °C according to the GP A. RP-HPFC (C-18, H_2O/MeOH 0 → 100 %) gave product **4A.4** (39.9 mg, 69 %) as a white powder. ^{1}H NMR (600.1 MHz, DMSO-d$_6$): 2.26 (t, 2H, $J_{CH2,P}$ = 20.3 Hz, PCH$_2$P); 4.06–4.17 (m, 3H, H-5′,4′); 4.22 (dd, 1H, $J_{3',2'}$ = 5.2 Hz, $J_{3',4'}$ = 3.1 Hz, H-3′); 4.48 (bt, 1H, $J_{2',3'}$ = $J_{2',1'}$ = 5.7 Hz, H-2′); 6.31 (d, 1H, $J_{1',2'}$ = 6.2 Hz, H-1′); 7.14 (d, 1H, $J_{5,6}$ = 3.8 Hz, H-5); 7.76 (dd, 1H, $J_{5,4}$ = 5.0 Hz, $J_{5,2}$ = 2.9 Hz, H-5-thienyl); 7.95 (d, 1H, $J_{6,5}$ = 3.8 Hz, H-6); 7.96 (dd, 1H, $J_{4,5}$ = 5.0 Hz, $J_{4,2}$ = 1.3 Hz, H-4-thienyl); 8.55 (dd, 1H, $J_{2,5}$ = 2.9 Hz, $J_{2,4}$ = 1.3 Hz, H-2-thienyl); 8.83 (s, 1H, H-2); ^{13}C NMR (150.9 MHz, DMSO-d$_6$): 28.0 (t, $J_{C,P}$ = 129.5 Hz, PCH$_2$P); 65.2 (d, $J_{C,P}$ = 4.9 Hz, CH$_2$-5′); 70.9 (CH-3′); 74.2 (CH-2′); 83.3 (d, $J_{C,P}$ = 7.0 Hz, CH-4′); 86.8 (CH-1′); 101.8 (C-5); 114.9 (C-4a); 127.6 (CH-5-thienyl); 127.9 (CH-4-thienyl); 128.2 (CH-6); 129.1 (CH-2-thienyl); 140.2 (C-3-thienyl); 151.4 (CH-2); 151.8 (C-4); 152.7 (C-7a); ^{31}P NMR (202.4 MHz, DMSO-d$_6$): 15.83 and 19.66 (2×s, 2×1P, PCH$_2$P). HR-ESI-MS: *found*: 490.0240 ([M–H]$^-$, calcd for $C_{16}H_{18}O_9N_3P_2S^-$: 490.0245); HR-ESI-MS: *found*: 512.0058 ([M–2H + Na]$^-$, calcd for $C_{16}H_{17}O_9N_3NaP_2S^-$: 512.0064).

[(5-{[4-Phenyl-7*H*-pyrrolo[2,3-*d*]pyrimidin-7-yl]-*β*-D-ribofuranosyl}oxy)phosphonomethyl]phosphonic acid (4A.5)

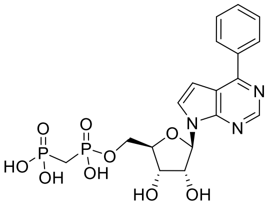

Compound **3** (51.5 mg, 0.12 mmol) was reacted with phenylboronic acid (21.2 mg, 0.17 mmol) for 5 min at 80 °C and 1 h at 65 °C according to the GP A. RP-HPFC (C-18, H_2O/MeOH 0 → 100 %), HPLC (C-18, H_2O + 0.05 % TFA/MeCN 0 → 80 %) gave product **4A.5** (34.1 mg, 61 %) as a white powder. ^{1}H NMR (500 MHz, DMSO-d$_6$): 2.26 (t, 2H, $J_{CH2,P}$ = 20.2 Hz, PCH$_2$P); 4.07–4.17 (m, 3H, H-4′,5′); 4.22 (dd, 1H, $J_{3',2'}$ = 5.3 Hz, $J_{3',4'}$ = 2.9 Hz, H-3′); 4.49 (bdd, 1H, $J_{2',1'}$ = 6.2 Hz, $J_{2',3'}$ = 5.2 Hz, H-2′); 6.33 (d, 1H, $J_{1',2'}$ = 6.2 Hz, H-1′); 7.01 (d, 1H, $J_{5,6}$ = 3.8 Hz, H-5); 7.55–7.63 (m, 3H, H-*m*,*p*-Ph); 7.97 (d, 1H, $J_{6,5}$ = 3.8 Hz, H-6); 8.17 (m, 2H, H-*o*-Ph);

8.91 (s, 1H, H-2); ^{13}C NMR (125.7 MHz, DMSO-d$_6$): 27.53 (t, $J_{C,P}$ = 129.4 Hz, PCH_2P); 64.8 (bd, $J_{C,P}$ = 3.5 Hz, CH_2-5′); 70.4 (CH-3′); 73.7 (CH-2′); 82.9 (d, $J_{C,P}$ = 6.8 Hz, CH-4′); 86.4 (CH-1′); 101.3 (CH-5); 115.4 (C-4a); 128.0 (CH-6); 128.7 (CH-*o*-Ph); 129.0 (CH-*m*-Ph); 130.3 (CH-*p*-Ph); 137.4 (C-*i*-Ph); 151.1 (CH-2); 152.2 (C-7a); 156.0 (C-4); ^{31}P NMR (202.4 MHz, DMSO-d$_6$): 15.82 and 19.71 (2×bs, 2×1P, PCH_2P). HR-ESI-MS: *found*: 484.0674 ([M–H]$^-$, calcd for $C_{18}H_{20}O_9N_3P_2^-$: 484.0669); HR-ESI-MS: *found*: 506.0490 ([M–2H + Na]$^-$, calcd for $C_{18}H_{19}O_9N_3NaP_2^-$: 506.0489).

[(5-{[4-(Naphth-1-yl)-7*H*-pyrrolo[2,3-*d*]pyrimidin-7-yl]-*β*-D-ribofuranosyl}oxy)phosphonomethyl]phosphonic acid (4A.6)

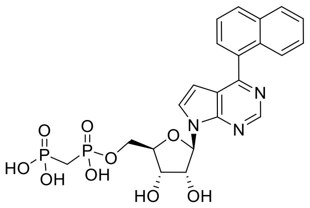

Compound **3** (68.4 mg, 0.15 mmol) was reacted with naphth-1-ylboronic acid (39.8 mg, 0.23 mmol) for 5 min at 80 °C and 1 h at 65 °C according to the GP A. HPLC (C-18, H_2O + 0.05 % TFA/MeCN 0 → 80 %) gave product **4A.6** (55.6 mg, 67 %) as a white powder. ^{1}H NMR (500 MHz, DMSO-d$_6$): 2.25 (t, 2H, $J_{CH2,P}$ = 20.5 Hz, PCH_2P); 4.09–4.18 (m, 3H, H-4′,5′); 4.23 (dd, 1H, $J_{3',2'}$ = 5.1 Hz, $J_{3',4'}$ = 2.5 Hz, H-3′); 4.52 (bt, 1H, $J_{2',1'}$ = $J_{2',3'}$ = 5.7 Hz, H-2′); 6.37 (d, 1H, $J_{1',2'}$ = 6.3 Hz, H-1′); 7.46 (d, 1H, $J_{5,6}$ = 3.8 Hz, H-5); 7.52 (ddd, 1H, $J_{7,8}$ = 8.5 Hz, $J_{7,6}$ = 6.8 Hz, $J_{7,5}$ = 1.4 Hz, H-7-naphthyl); 7.60 (ddd, 1H, $J_{6,5}$ = 8.2 Hz, $J_{6,7}$ = 6.8 Hz, $J_{6,8}$ = 1.3 Hz, H-6-naphthyl); 7.70 (dd, 1H, $J_{3,4}$ = 8.21 Hz, $J_{3,2}$ = 7.1 Hz, H-3-naphthyl); 7.79 (dd, 1H, $J_{2,3}$ = 7.1 Hz, $J_{2,4}$ = 1.3 Hz, H-2-naphthyl); 7.94 (d, 1H, $J_{6,5}$ = 3.8 Hz, H-6); 8.03 (bd, 1H, $J_{8,7}$ = 8.5 Hz, H-8-naphthyl); 8.07 (bd, 1H, $J_{4,5}$ = 8.3 Hz, H-4-naphthyl); 8.14 (bd, 1H, $J_{4,3}$ = 8.3 Hz, H-4-naphthyl); 9.03 (s, 1H, H-2); ^{13}C NMR (125.7 MHz, DMSO-d$_6$): 27.5 (t, $J_{C,P}$ = 129.0 Hz, PCH_2P); 64.8 (d, $J_{C,P}$ = 5.1 Hz, CH_2-5′); 70.4 (CH-3′); 73.7 (CH-2′); 83.0 (d, $J_{C,P}$ = 7.2 Hz, CH-4′); 86.5 (CH-1′); 101.3 (CH-5); 118.1 (C-4a); 125.43 and 125.45 (CH-3,8-naphthyl); 126.4 (CH-6-naphthyl); 126.8 (CH-7-naphthyl); 128.1 (CH-6); 128.2 (CH-2-naphthyl); 128.4 (CH-5-naphthyl); 130.0 (CH-4-naphthyl); 130.3 (C-8a-naphthyl); 133.5 (C-4a-naphthyl); 134.0 (C-1-naphthyl); 150.6 (CH-2); 151.8 (C-7a); 157.4 (C-4); ^{31}P NMR (202.4 MHz, DMSO-d$_6$): 15.71 and 19.83 (2×d, 2×1P, $J_{P,P}$ = 6.8 Hz, PCH_2P). HR-ESI-MS: *found*: 534.0835 ([M–H]$^-$, calcd for $C_{22}H_{22}O_9N_3P_2^-$: 534.0837).

[(5-{[4-(Naphthalen-2-yl)-7*H*-pyrrolo[2,3-*d*]pyrimidin-7-yl]-*β*-D-ribofuranosyl}oxy)phosphonomethyl]phosphonic acid (4A.7)

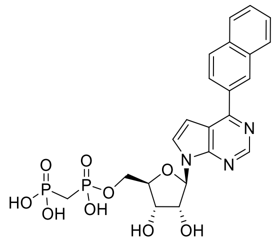

Compound **3** (55.2 mg, 0.12 mmol) was reacted with naphthalen-2-ylboronic acid (32.1 mg, 0.19 mmol) for 5 min at 80 °C and 1 h at 65 °C according to the GP A. RP-HPFC (C-18, H_2O/MeOH 0 → 100 %) gave product **4A.7** (62.8 mg, 95 %) as a white powder. ^{1}H NMR (600.1 MHz, D_2O): 2.15 (t, 2H, $J_{CH2,P}$ = 19.7 Hz, PCH_2P); 4.16 (ddd, 1H, J_{gem} = 11.5 Hz, $J_{5'a,P}$ = 5.0 Hz, $J_{5'a,4'}$ = 3.8 Hz, H-5′a); 4.22 (ddd, 1H, J_{gem} = 11.5 Hz, $J_{5'b,P}$ = 5.9 Hz, $J_{5'b,4'}$ = 3.7 Hz, H-5′b); 4.28 (bq, 1H, $J_{4',5'}$ = $J_{4',3'}$ = 3.7 Hz, H-4′); 4.60 (dd, 1H, $J_{3',2'}$ = 5.2 Hz, $J_{3',4'}$ = 3.9 Hz, H-3′); 4.75 (t, 1H, $J_{2',3'}$ = $J_{2',1'}$ = 5.6 Hz, H-2′); 6.37 (d, 1H, $J_{1',2'}$ = 5.9 Hz, H-1′); 6.92 (d, 1H, $J_{5,6}$ = 3.8 Hz, H-5); 7.51 (ddd, 1H, $J_{7,8}$ = 8.0 Hz, $J_{7,6}$ = 6.8 Hz, $J_{7,5}$ = 1.3 Hz, H-7-naphthyl); 7.55 (ddd, 1H, $J_{6,5}$ = 8.0 Hz, $J_{6,7}$ = 6.8 Hz, $J_{6,8}$ = 1.3 Hz, H-6-naphthyl); 7.83 (d, 1H, $J_{5,6}$ = 8.0 Hz, H-5-naphthyl); 7.85 (bd, 1H, $J_{6,5}$ = 3.7 Hz, H-6); 7.86 (d, 1H, $J_{8.7}$ = 8.0 Hz, H-8-naphthyl); 7.88 (bs, 2H, H-3,4-naphthyl); 8.22 (s, 1H, H-2-naphthyl); 8.60 (s, 1H, H-2); ^{13}C NMR (150.9 MHz, D_2O): 30.2 (bt, $J_{C,P}$ = 123.5 Hz, PCH_2P); 65.6 (d, $J_{C,P}$ = 5.2 Hz, CH_2-5′); 72.6 (CH-3′); 75.9 (CH-2′); 85.4 (d, $J_{C,P}$ = 8.0 Hz, CH-4′); 88.3 (CH-1′); 105.0 (C-5); 118.6 (C-4a); 127.3 (CH-3-naphthyl); 128.7 (CH-7-naphthyl); 129.0 (CH-6); 129.5 (CH-5-naphthyl); 129.6 (CH-6-naphthyl); 130.47 and 130.51 (CH-4,8-naphthyl); 131.2 (CH-1-naphthyl); 134.4 (C-8a-naphthyl); 135.5 and 135.6 (C-2,4a-naphthyl); 152.2 (CH-2); 153.3 (C-7a); 159.2 (C-4); ^{31}P NMR (202.4 MHz, D_2O): 13.52 and 21.42 (2×d, 2×1P, $J_{P,P}$ = 8.8 Hz, PCH_2P). HR-ESI-MS: *found*: 534.0832 ([M–H]$^-$, calcd for $C_{22}H_{22}O_9N_3P_2^-$: 534.0837); HR-ESI-MS: *found*: 556.0652 ([M–2H + Na]$^-$, calcd for $C_{22}H_{21}O_9N_3NaP_2^-$: 556.0656).

[(5-{[4-(Benzofuran-2-yl)-7*H*-pyrrolo[2,3-*d*]pyrimidin-7-yl]-*β*-d-ribofuranosyl}oxy)phosphonomethyl]phosphonic acid (4A.8)

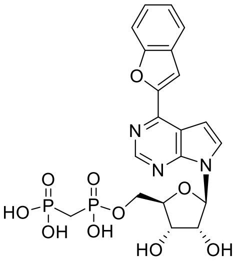

Phosphonic acid **17** was prepared according to the literature procedure.[1] Nucleoside **8d** (240 mg, 0.59 mmol) was dissolved in trimethylphosphate (4 mL) and cooled to 0 °C. A cold solution of methylene bis(phosphonic dichloride) (736 mg, 2.9 mmol) in trimethylphosphate (2 mL) was added dropwise and the mixture was strirred at 0 °C for 3 h. The mixture was treated with water (2 mL) and evaporated. HPLC (C-18, H_2O + 0.05% TFA/MeCN 0 →80 %) and lyophilization from H_2O gave product **4A.8** (51 mg, 17%) as a white powder.

^{1}H NMR (500.0 MHz, DMSO-d_6): 2.29 (t, 2H, $J_{H,P}$ = 20.0, CH_2P); 4.09–4.18 (bm, 3H, H-4′,5′); 4.23 (dd, 1H, $J_{3',2'}$ = 5.1, $J_{3',4'}$ = 3.3, H-3′); 4.50 (dd, 1H, $J_{2',1'}$ = 6.2, $J_{2',3'}$ = 5.1, H-2′); 6.33 (d, 1H, $J_{1',2'}$ = 6.2, H-1′); 7.29 (d, 1H, $J_{5,6}$ = 3.8, H-5); 7.37 (ddd, 1H, $J_{5,4}$ = 7.8, $J_{5,6}$ = 7.2, $J_{5,7}$ = 1.1, H-5-benzofuryl); 7.48 (ddd, 1H, $J_{6,7}$ = 8.3, $J_{6,5}$ = 7.2, $J_{6,4}$ = 1.3, H-6-benzofuryl);. 7.81–7.84 (m, 2H, H-4,7-benzofuryl); 7.94 (d, 1H, $J_{3,7}$ = 0,8, H-3-benzofuryl);. 8.05 (d, 1H, $J_{6,5}$ = 3.8, H-6); 8.90 (s, 1H, H-2). ^{13}C NMR (125.7 MHz, DMSO-d_6): 28,26 (d, $J_{C,P}$ = 130.4, CH_2P); 64.95 (d, $J_{C,P}$ = 4.5, CH_2-5′); 70.57 (CH-3′); 73.96 (CH-2′); 83.10 (d, $J_{C,P}$ = 7.2, CH-4′); 86.54 (CH-1′); 101.97 (CH-5); 109.17 (CH-3-benzofuryl); 112.11 (CH-7-benzofuryl); 114.03 (C-4a); 122.62 (CH-4-benzofuryl); 123.99 (CH-5-benzofuryl); 126.70 (CH-6-benzofuryl); 127.94 (C-3a-benzofuryl); 128.77 (CH-6); 146.36 (C-4); 151.30 (CH-2); 152.75 (C-7a); 154.20 (C-2-benzofuryl); 155.50 (C-7a-benzofuryl). ^{31}P{^{1}H} NMR (202.4 MHz, DMSO-d_6): 15.81, 19.82. HR-ESI-MS: *found*: 526.07716 ($[M + H]^+$, calcd for $C_{20}H_{22}O_{10}N_3P_2^+$: 526.07749).

[(5-{[4-([1,1'-Biphenyl]-4-yl)-7*H*-pyrrolo[2,3-*d*]pyrimidin-7-yl]-*β*-D-ribofuranosyl}oxy)phosphonomethyl]phosphonic acid (4A.9)

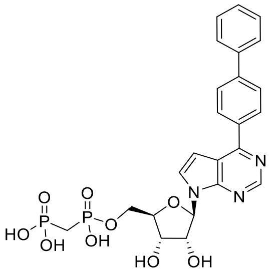

Compound **3** (53.6 mg, 0.12 mmol) was reacted with 4-biphenylboronic acid (35.9 mg, 0.18 mmol) for 5 min at 80 °C and 1 h at 65 °C according to the GP A. RP-HPFC (C-18, H_2O/MeOH 0 → 100 %), HPLC (C-18, H_2O + 0.05 % TFA/MeCN 0 → 80 %) gave product **4A.9** (24.4 mg, 36 %) as a pale-yellow powder. ^{1}H NMR (500 MHz, DMSO-d_6): 2.27 (t, 2H, $J_{CH2,P}$ = 20.3 Hz, PCH_2P); 4.07–4.19 (m, 3H, H-4′,5′); 4.23 (m, 1H, H-3′); 4.50 (t, 1H, $J_{2',1'} = J_{2',3'}$ = 5.7 Hz, H-2′); 6.35 (d, 1H, $J_{1',2'}$ = 6.2 Hz, H-1′); 7.08 (d, 1H, $J_{5,6}$ = 3.8 Hz, H-5); 7.43 (m, 1H, H-4′-biphe); 7.52 (m, 2H, H-3′-biphe); 7.79 (m, 2H, H-2′-biphe); 7.91 (m, 2H, H-3-biphe); 8.00 (d, 1H, $J_{6,5}$ = 3.8 Hz, H-6); 8.29 (m, 2H, H-2-biphe); 8.93 (s, 1H, H-2); ^{13}C NMR (125.7 MHz, DMSO-d_6): 27.6 (t, $J_{C,P}$ = 128.9 Hz, PCH_2P); 64.8 (bs, CH_2-5′); 70.4 (CH-3′); 73.7 (CH-2′); 82.9 (d, $J_{C,P}$ = 6.9 Hz, CH-4′); 86.4 (CH-1′); 101.3 (CH-5); 115.3 (C-4a); 126.8 (CH-2′-biphe); 127.2 (CH-3-biphe); 128.0 (CH-6, CH-4′-biphe); 129.1 (CH-3′-biphe); 129.3 (CH-2-biphe); 136.4 (C-1-biphe); 139.3 (C-1′-biphe); 141.9 (C-4-biphe); 151.1 (CH-2); 152.3 (C-7a); 155.5 (C-4); ^{31}P NMR (202.4 MHz, DMSO-d_6): 15.80 and 19.76 (2×bs, 2×1P, PCH_2P). HR-ESI-MS: *found*: 560.0988 ($[M–H]^-$, calcd for $C_{24}H_{24}O_9N_3P_2^-$: 560.0982); HR-ESI-MS: *found*: 582.0807 ($[M–2H + Na]^-$, calcd for $C_{24}H_{23}O_9N_3NaP_2^-$: 582.0802).

[(5-{[4-(Dibenzo[*b,d*]furan-4-yl)-7*H*-pyrrolo[2,3-*d*]pyrimidin-7-yl]-*β*-D-ribofuranosyl}oxy)phosphonomethyl]phosphonic acid (4A.10)

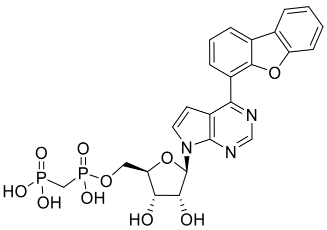

Compound **3** (59.3 mg, 0.13 mmol) was reacted with dibenzofuran-4-boronic acid (42.5 mg, 0.20 mmol) for 5 min at 80 °C and 1 h at 65 °C according to the GP A. RP-HPFC (C-18, H_2O/MeOH 0 → 100 %) gave product **4A.10** (63.5 mg, 82 %) as a white powder. ^{1}H NMR (500 MHz, D_2O): 2.18 (t, 2H, $J_{CH2,P}$ = 19.7 Hz, PCH_2P); 4.17 (ddd, 1H, J_{gem} = 11.4 Hz, $J_{5'a,P}$ = 5.0 Hz, $J_{5'a,4'}$ = 4.0 Hz, H-5′a); 4.22 (ddd, 1H, J_{gem} = 11.4 Hz, $J_{5'b,P}$ = 5.8 Hz, $J_{5'b,4'}$ = 3.8 Hz, H-5′b); 4.41 (bq, 1H, $J_{4',5'} = J_{4',3'}$ = 3.8 Hz, H-4′); 4.58 (dd, 1H, $J_{3',2'}$ = 5.3 Hz, $J_{3',4'}$ = 3.9 Hz, H-3′); 4.75 (t, 1H, $J_{2',3'} = J_{2',1'}$ = 5.7 Hz, H-2′); 6.38 (d, 1H, $J_{1',2'}$ = 5.9 Hz, H-1′); 6.53 (d, 1H, $J_{5,6}$ = 3.7 Hz, H-5); 7.29 (t, 1H, $J_{2,1} = J_{2,3}$ = 7.8 Hz, H-2-dibenzofuryl); 7.42 (td, 1H, $J_{8,7} = J_{8,9}$ = 7.3 Hz, $J_{8,6}$ = 1.2 Hz, H-8-dibenzofuryl); 7.51 (bddd, 1H, $J_{7,6}$ = 8.2 Hz, $J_{7,8}$ = 7.0 Hz, $J_{7,9}$ = 1.2 Hz, H-7-dibenzofuryl); 7.56 (d, 1H, $J_{6,7}$ = 8.2 Hz, H-6-dibenzofuryl); 7.63 (bd, 1H, $J_{6,5}$ = 3.7 Hz, H-6); 7.65

(bd, 1H, $J_{3.2}$ = 7.8 Hz, H-3-dibenzofuryl); 7.91 (d, 1H, $J_{1.2}$ = 7.7 Hz, H-1-dibenzofuryl); 7.96 (d, 1H, $J_{9.8}$ = 7.6 Hz, H-9-dibenzofuryl); 8.41 (s, 1H, H-2); ^{13}C NMR (125.7 MHz, D_2O): 30.0 (dd, $J_{C,P}$ = 126.0 and 121.8 Hz, PCH_2P); 65.8 (d, $J_{C,P}$ = 5.1 Hz, CH_2-5′); 72.6 (CH-3′); 75.9 (CH-2′); 85.4 (d, $J_{C,P}$ = 8.0 Hz, CH-4′); 88.2 (CH-1′); 105.1 (C-5); 113.6 (CH-6-dibenzofuryl); 119.3 (C-4a); 122.5 (C-4-dibenzofuryl); 122.8 (CH-9-dibenzofuryl); 124.9 (CH-2-dibenzofuryl); 124.9 (C-9a-dibenzofuryl); 125.0 (CH-1-dibenzofuryl); 125.3 (CH-8-dibenzofuryl); 126.7 (C-9b-dibenzofuryl); 128.6 (CH-6); 129.7 (CH-7-dibenzofuryl); 130.2 (CH-3-dibenzofuryl); 151.9 (CH-2); 153.1 (C-7a); 154.4 (C-4a-dibenzofuryl); 156.0 (C-4); 157.3 (C-5a-dibenzofuryl); ^{31}P NMR (202.4 MHz, D_2O): 14.27 and 20.52 (bd, 1P, $J_{P,P}$ = 8.7 Hz, PCH_2P). HR-ESI-MS: *found*: 574.0782 ($[M–H]^-$, calcd for $C_{24}H_{22}O_{10}N_3P_2^-$: 574.0786); HR-ESI-MS: *found*: 596.0597 ($[M–2H + Na]^-$, calcd for $C_{24}H_{21}O_{10}N_3NaP_2^-$: 596.0605).

[(5-{[4-(Phenanthren-9-yl)-7*H*-pyrrolo[2,3-*d*]pyrimidin-7-yl]-*β*-D-ribofuranosyl}oxy)phosphonomethyl]phosphonic acid (4A.11)

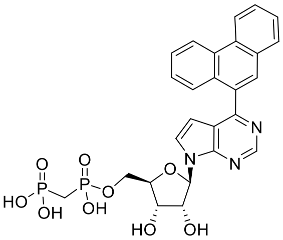

Compound **3** (61.5 mg, 0.14 mmol) was reacted with 9-phenanthrenylboronic acid (46.2 mg, 0.21 mmol) for 5 min at 80 °C and 1 h at 65 °C according to the GP A. HPLC (C-18, H_2O + 0.05 % TFA/MeCN 0 → 80 %) gave product **4A.11** (73.9 mg, 91 %) as a white powder. ^{1}H NMR (500 MHz, DMSO-d$_6$): 2.25 (t, 2H, $J_{CH2,P}$ = 20.4 Hz, PCH_2P); 4.10–4.19 (m, 3H, H-4′,5′); 4.24 (dd, 1H, $J_{3',2'}$ = 5.1 Hz, $J_{3',4'}$ = 2.5 Hz, H-3′); 4.54 (bt, 1H, $J_{2',1'}$ = $J_{2',3'}$ = 5.7 Hz, H-2′); 6.39 (d, 1H, $J_{1',2'}$ = 6.3 Hz, H-1′); 6.52 (d, 1H, $J_{5,6}$ = 3.8 Hz, H-5); 7.62 (ddd, 1H, $J_{7,8}$ = 8.3 Hz, $J_{7,6}$ = 6.9 Hz, $J_{7,5}$ = 1.2 Hz, H-7-phenantrenyl); 7.73 (ddd, 1H, $J_{2,1}$ = 7.9 Hz, $J_{2,3}$ = 7.1 Hz, $J_{2,4}$ = 1.1 Hz, H-2-phenantrenyl); 7.77 (ddd, 1H, $J_{6,5}$ = 8.4 Hz, $J_{6,7}$ = 7.0 Hz, $J_{6,8}$ = 1.4 Hz, H-6-phenantrenyl); 7.81 (ddd, 1H, $J_{3,4}$ = 8.4 Hz, $J_{3,2}$ = 7.0 Hz, $J_{3,1}$ = 1.4 Hz, H-3-phenantrenyl); 7.96 (bd, 1H, $J_{6,5}$ = 3.9 Hz, H-6); 8.03 (dd, 1H, $J_{8,7}$ = 8.3 Hz, $J_{8,6}$ = 1.3 Hz, H-8-phenantrenyl); 8.12 (dd, 1H, $J_{1,2}$ = 8.0 Hz, $J_{1,3}$ = 1.4 Hz, H-1-phenantrenyl); 8.14 (s, 1H, H-10-phenantrenyl); 8.95 (bd, 1H, $J_{4,3}$ = 8.5 Hz, H-4-phenantrenyl); 8.99 (bd, 1H, $J_{5,6}$ = 8.5 Hz, H-5-phenantrenyl); 9.06 (s, 1H, H-2); ^{13}C NMR (125.7 MHz, DMSO- d$_6$): 27.55 (t, $J_{C,P}$ = 129.0 Hz, PCH_2P); 64.8 (d, $J_{C,P}$ = 4.5 Hz, CH_2-5′); 70.5 (CH-

3′); 73.75 (CH-2′); 83.0 (d, $J_{C,P}$ = 7.1 Hz, CH-4′); 86.5 (CH-1′); 101.4 (CH-5); 118.3 (C-4a); 123.0 (CH-4-phenantrenyl); 123.4 (CH-5-phenantrenyl); 126.4 (CH-8-phenantrenyl); 127.0 (CH-7-phenantrenyl); 127.2 and 127.4 (CH-2,6-phenantrenyl); 128.1 (CH-3-phenantrenyl); 128.1 (CH-6); 129.1 (C-8a-phenantrenyl); 129.3 and 129.4 (CH-1,10-phenantrenyl); 130.2 and 130.3 (C-4a,4b-phenantrenyl); 130.6 (C-10a-phenantrenyl); 132.8 (C-9-phenantrenyl); 150.6 (CH-2); 151.8 (C-7a); 157.5 (C-4); ^{31}P NMR (202.4 MHz, DMSO-d$_6$): 15.69 and 19.84 (2×bs, 2×1P, PCH$_2$P). HR-ESI-MS: *found*: 584.0991 ([M–H]$^-$, calcd for $C_{26}H_{24}O_9N_3P_2^-$: 584.0993).

[(5-{[(4-(5,6,7,8-Tetrahydronaphth-1-yl)-7*H*-pyrrolo[2,3-*d*]pyrimidin-7-yl]-*β*-D-ribofuranosyl}oxy)phosphonomethyl]phosphonic acid (4A.12)

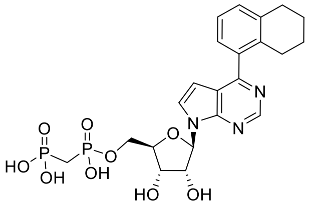

Compound **3** (88.3 mg, 0.20 mmol) was reacted with 5,6,7,8-tetrahydronaphth-1-ylboronic acid (52.5 mg, 0.30 mmol) for 1 h at 80 °C according to the GP A. HPLC (C-18, H_2O + 0.05 % TFA/MeCN 0 → 80 %) gave product **4A.12** (35.5 mg, 33 %) as a white powder. ^{1}H NMR (500 MHz, DMSO-d$_6$): 1.64 (m, 2H, H-3-$C_{10}H_{11}$); 1.75 (H-2-$C_{10}H_{11}$); 2.25 (t, 2H, $J_{CH2,P}$ = 20.5 Hz, PCH$_2$P); 2.63 (H-4-$C_{10}H_{11}$); 2.84 (H-1-$C_{10}H_{11}$); 4.07–4.16 (m, 3H, H-4′,5′); 4.21 (dd, 1H, $J_{3',2'}$ = 5.2 Hz, $J_{3',4'}$ = 2.7 Hz, H-3′); 4.50 (dd, 1H, $J_{2',1'}$ = 6.3 Hz, $J_{2',3'}$ = 5.2 Hz, H-2′); 6.32 (d, 1H, $J_{1',2'}$ = 6.3 Hz, H-1′); 6.47 (d, 1H, $J_{5,6}$ = 3.8 Hz, H-5); 7.21–7.29 (m, 3H, H-6,7,8-$C_{10}H_{11}$); 7.91 (d, 1H, $J_{6,5}$ = 3.8 Hz, H-6); 8.91 (s, 1H, H-2); ^{13}C NMR (125.7 MHz, DMSO-d$_6$): 22.3 (CH$_2$-2-$C_{10}H_{11}$); 22.5 (CH$_2$-3-$C_{10}H_{11}$); 26.8 (CH$_2$-4-$C_{10}H_{11}$); 27.5 (t, $J_{C,P}$ = 128.8 Hz, PCH$_2$P); 29.3 (CH$_2$-1-$C_{10}H_{11}$); 64.7 (d, $J_{C,P}$ = 5.5 Hz, CH$_2$-5′); 70.4 (CH-3′); 73.7 (CH-2′); 83.0 (d, $J_{C,P}$ = 7.4 Hz, CH-4′); 86.4 (CH-1′); 101.3 (CH-5); 117.5 (C-4a); 125.3 and 126.9 (CH-6,7-$C_{10}H_{11}$); 128.0 (CH-6); 130.2 (CH-8-$C_{10}H_{11}$); 135.0 (C-4a/8a-$C_{10}H_{11}$); 136.1 (C-5-$C_{10}H_{11}$); 137.7 (C-8a/4a-$C_{10}H_{11}$); 150.1 (CH-2); 151.5 (C-7a); 158.7 (C-4); ^{31}P NMR (202.4 MHz, DMSO-d$_6$): 15.72 and 19.79 (2×d, 2×1P, $J_{P,P}$ = 8.1 Hz, PCH$_2$P). HR-ESI-MS: *found*: 538.1147 ([M–H]$^-$, calcd for $C_{22}H_{26}O_9N_3P_2^-$: 538.1150).

[(5-{[(4-(Chroman-8-yl)-7*H*-pyrrolo[2,3-*d*]pyrimidin-7-yl]-*β*-D-ribofuranosyl}oxy)phosphonomethyl]phosphonic acid (4A.13)

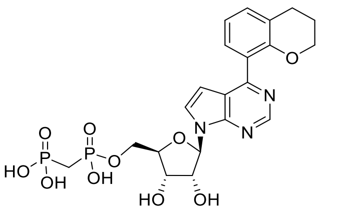

Compound **3** (91.1 mg, 0.21 mmol) was reacted with chroman-8-ylboronic acid (54.8 mg, 0.31 mmol) for 1 h at 80 °C according to the GP A. HPLC (C-18, H_2O + 0.05 % TFA/MeCN 0 → 80 %) gave product **4A.13** (34.2 mg, 31 %) as a white powder. ^1H NMR (500 MHz, DMSO-d$_6$): 1.94–2.03 (m, 2H, H-3-chromanyl); 2.26 (t, 2H, d, $J_{CH2,P}$ = 20.5 Hz, PCH_2P); 2.87 (t, 2H, $J_{4,3}$ = 6.4 Hz, H-4-chromanyl); 4.09–4.14 (m, 3H, H-4′,5′); 4.16 (m, 2H, H-2-chromanyl); 4.22 (dd, 1H, $J_{3',2'}$ = 5.1 Hz, $J_{3',4'}$ = 2.5 Hz, H-3′); 4.49 (dd, 1H, $J_{2',1'}$ = 6.4 Hz, $J_{2',3'}$ = 5.1 Hz, H-3′); 6.31 (d, 1H, $J_{2',1'}$ = 6.4 Hz, H-1′); 6.62 (d, 1H, $J_{5,6}$ = 3.8 Hz, H-5); 7.01 (t, 1H, $J_{6,5} = J_{6,7}$ = 7.5 Hz, H-7-chromanyl); 7.28 (dd, 1H, $J_{5,6}$ = 7.5 Hz, $J_{5,7}$ = 1.7 Hz, H-5-chromanyl); 7.37 (dd, 1H, $J_{7,6}$ = 7.6 Hz, $J_{7,5}$ = 1.7 Hz, H-7-naphthyl); 7.93 (d, 1H, $J_{6,5}$ = 3.8 Hz, H-6); 8.96 (s, 1H, H-2); ^{13}C NMR (125.7 MHz, DMSO-d$_6$): 21.4 (CH_2-3-chromanyl); 24.5 (CH_2-4-chromanyl); 27.5 (t, $J_{C,P}$ = 128.8 Hz, PCH_2P); 64.8 (d, $J_{C,P}$ = 5.4 Hz, CH_2-5′); 66.2 (CH_2-2-chromanyl); 70.4 (CH-3′); 73.7 (CH-2′); 83.1 (d, $J_{C,P}$ = 7.5 Hz, CH-4′); 86.4 (CH-1′); 103.0 (CH-5); 117.6 (C-4a); 119.9 (CH-6-chromanyl); 123.4 (C-4a-chromanyl); 127.9 (CH-6); 129.0 (CH-7-chromanyl); 132.1 (CH-5-chromanyl); 149.6 (CH-2); 151.2 (C-7a); 152.3 (C-8a-chromanyl); 152.8 (C-8-chromanyl); 154.6 (C-4); ^{31}P NMR (202.4 MHz, DMSO-d$_6$): 19.77 and 15.74 (2×d, 2×1P, $J_{P,P}$ = 7.7 Hz, PCH_2P). HR-ESI-MS: *found*: 540.0942 ([M–H]$^-$, calcd for $C_{21}H_{24}O_{10}N_3P_2^-$: 540.0942).

[(5-{[4-(2,3-dihydrobenzofuran-5-yl)-7*H*-pyrrolo[2,3-*d*]pyrimidin-7-yl]-*β*-D-ribofuranosyl}oxy)phosphonomethyl]phosphonic acid (4A.14)

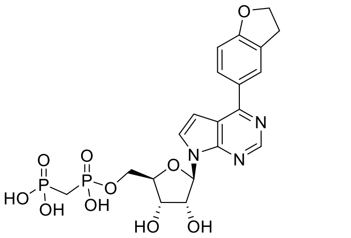

Compound **3** (73.1 mg, 0.17 mmol) was reacted with 2,3-dihydrobenzofuran-5-boronic acid (40.5 mg, 0.25 mmol) for 1 h at 80 °C according to the GP A. HPLC (C-18, H_2O + 0.05 % TFA/MeCN 0 → 80 %) gave product **4A.14** (32 mg, 37 %) as a white powder. ^1H NMR (500 MHz, DMSO-d$_6$): 2.26 (t, 2H, $J_{CH2,P}$ = 20.4 Hz, PCH_2P); 3.31 (t, 2H, $J_{3,2}$ = 8.8 Hz, H-3-C_8H_7O); 4.07–

4.17 (m, 3H, H-4′,5′); 4.21 (dd, 1H, $J_{3',2'}$ = 5.2 Hz, $J_{3',4'}$ = 3.0 Hz, H-3′); 4.48 (dd, 1H, $J_{2',1'}$ = 6.2 Hz, $J_{2',3'}$ = 5.2 Hz, H-2′); 4.65 (t, 2H, $J_{2,3}$ = 8.8 Hz, H-2-C_8H_7O); 6.31 (d, 1H, $J_{1',2'}$ = 6.2 Hz, H-1′); 6.97 (d, 1H, $J_{7,6}$ = 8.4 Hz, H-7-C_8H_7O); 7.03 (d, 1H, $J_{5,6}$ = 3.8 Hz, H-5); 7.94 (d, 1H, $J_{6,5}$ = 3.9 Hz, H-6); 7.97 (dd, 1H, $J_{6,7}$ = 8.4 Hz, $J_{6,4}$ = 1.9 Hz, H-6-C_8H_7O); 8.08 (bd, 1H, $J_{4,6}$ = 1.9 Hz, H-4-C_8H_7O); 8.85 (s, 1H, H-2); ^{13}C NMR (125.7 MHz, DMSO-d$_6$): 27.6 (t, $J_{C,P}$ = 128.6 Hz, PCH_2P); 28.9 (CH_2-3-C_8H_7O); 64.8 (d, $J_{C,P}$ = 5.3 Hz, CH_2-5′); 70.5 (CH-3′); 71.8 (CH_2-2-C_8H_7O); 73.8 (CH-2′); 82.9 (d, $J_{C,P}$ = 7.1 Hz, CH-4′); 86.4 (CH-1′); 101.8 (CH-5); 109.4 (CH-7-C_8H_7O); 114.6 (C-4a); 125.8 (CH-4-C_8H_7O); 127.7 (CH-6); 128.6 (C-3a-C_8H_7O); 129.2 (C-5-C_8H_7O); 129.5 (CH-6-C_8H_7O); 150.5 (CH-2); 152.1 (C-7a); 155.7 (C-4); 162.0 (C-7a-6-C_8H_7O); ^{31}P NMR (202.4 MHz, DMSO-d$_6$): 15.73 and 19.73 (2×s, 2×1P, PCH_2P). HR-ESI-MS: *found*: 526.0782 ([M–H]$^-$, calcd for $C_{20}H_{22}O_{10}N_3P_2^-$: 526.0786).

[(5-{[4-(Benzofuran-7-yl)-7*H*-pyrrolo[2,3-*d*]pyrimidin-7-yl]-*β*-D-ribofuranosyl}oxy)phosphonomethyl]phosphonic acid (4A.15)

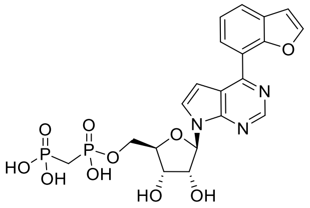

Compound **3** (63.3 mg, 0.14 mmol) was reacted with benzofuran-7-ylboronic acid (34.7 mg, 0.21 mmol) for 1 h at 80 °C according to the GP A. HPLC (C-18, H_2O + 0.05 % TFA/MeCN 0 → 80 %) and lyophilization from H_2O/*t*-BuOH gave product **4A.15** (16.2 mg, 22 %) as a pale-yellow powder. ^{1}H NMR (500 MHz, DMSO-d$_6$): 2.24 (bt, 2H, $J_{CH2,P}$ = 16.5 Hz, PCH_2P); 4.07–4.19 (m, 3H, H-4′,5′); 4.23 (m, 1H, H-3′); 4.51 (t, 1H, $J_{2',1'}$ = $J_{2',3'}$ = 5.5 Hz, H-2′); 6.36 (d, 1H, $J_{1',2'}$ = 6.2 Hz, H-1′); 6.68 (d, 1H, $J_{5,6}$ = 3.6 Hz, H-5); 7.12 (d, 1H, $J_{2,4}$ = 2.2 Hz, H-2-benzofuryl); 7.47 (t, 1H, $J_{5,6}$ = $J_{5,4}$ = 7.6 Hz, H-5-benzofuryl); 7.83 (dd, 1H, $J_{6,5}$ = 7.6 Hz, $J_{6,4}$ = 1.2 Hz, H-6-benzofuryl); 7.87 (dd, 1H, $J_{4,5}$ = 7.7 Hz, $J_{4,6}$ = 1.2 Hz, H-4-benzofuryl); 7.94 (bd, 1H, $J_{6,5}$ = 3.5 Hz, H-6); 8.10 (d, 1H, $J_{2,4}$ = 2.2 Hz, H-2-benzofuryl); 8.96 (s, 1H, H-2); ^{13}C NMR (125.7 MHz, DMSO-d$_6$): 64.8 (CH_2-5′); 70.5 (CH-3′); 73.8 (CH-2′); 83.0 (CH-4′); 86.3 (CH-1′); 101.9 (CH-5); 107.0 (CH-3-benzofuryl); 117.0 (C-4a); 122.1 (CH-7-benzofuryl); 123.25 and 123.3 (CH-4,5-benzofuryl); 125.5 (CH-6-benzofuryl); 127.4 (CH-6); 128.5 (C-3a-benzofuryl); 146.5 (CH-2-benzofuryl); 151.2 (CH-2); 151.6 (C-7a-benzofuryl); 151.9 (C-7a); 153.7 (C-4); *Signal of carbon PCH_2P was*

not detected. ^{31}P NMR (202.4 MHz, DMSO-d$_6$): 15.91 and 19.52 (2×s, 2×1P, PCH$_2$P). HR-ESI-MS: *found*: 524.0625 ([M–H]$^-$, calcd for $C_{20}H_{20}O_{10}N_3P_2^-$: 524.0629).

[(5-{[4-(Pyridin-4-yl)-7*H*-pyrrolo[2,3-*d*]pyrimidin-7-yl]-*β*-D-ribofuranosyl}oxy)phosphonomethyl]phosphonic acid (4A.16)

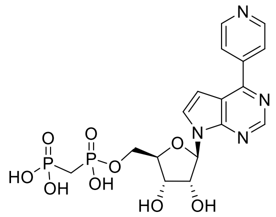

Compound **3** (72.1 mg, 0.16 mmol) was reacted with pyridine-4-boronic acid (30.0 mg, 0.24 mmol) for 1 h at 80 °C according to the GP A. HPLC (C-18, H_2O + 0.05 % TFA/MeCN 0 → 80 %) gave product **4A.16** (12.1 mg, 15 %) as a pale-yellow powder. ^{1}H NMR (500 MHz, DMSO-d$_6$): 2.27 (t, 2H, d, $J_{CH2,P}$ = 20.5 Hz, PCH$_2$P); 4.08–4.18 (m, 3H, H-4′,5′); 4.23 (dd, 1H, $J_{3',2'}$ = 5.1 Hz, $J_{3',4'}$ = 2.9 Hz, H-3′); 4.50 (dd, 1H, $J_{2',1'}$ = 6.2 Hz, $J_{2',3'}$ = 5.1 Hz, H-2′); 6.35 (d, 1H, $J_{2',1'}$ = 6.2 Hz, H-1′); 7.12 (d, 1H, $J_{5,6}$ = 3.9 Hz, H-5); 8.08 (d, 1H, $J_{6,5}$ = 3.9 Hz, H-6); 8.21 (m, 2H, H-3,5-py); 8.86 (m, 2H, H-2,6-py); 9.00 (s 1H, H-2); ^{13}C NMR (125.7 MHz, DMSO-d$_6$): 27.6 (t, $J_{C,P}$ = 128.7 Hz, PCH$_2$P); 64.7 (d, $J_{C,P}$ = 5.5 Hz, CH$_2$-5′); 70.4 (CH-3′); 73.8 (CH-2′); 83.0 (d, $J_{C,P}$ = 7.5 Hz, CH-4′); 86.4 (CH-1′); 100.9 (CH-5); 116.0 (C-4a); 123.2 (CH-3,5-py); 129.2 (CH-6); 145.7 (C-1-py); 149.4 (CH-2,6-py); 151.2 (CH-2); 152.5 (C-7a); 152.9 (C-4); ^{31}P NMR (202.4 MHz, DMSO-d$_6$): 15.78 and 19.77 (2×d, 2×1P, $J_{P,P}$ = 7.7 Hz, PCH$_2$P). HR-ESI-MS: *found*: 485.0628 ([M–H]$^-$, calcd for $C_{17}H_{19}O_9N_4P_2^-$: 485.0633).

[(5-{[4-(Quinolin-4-yl)-7*H*-pyrrolo[2,3-*d*]pyrimidin-7-yl]-*β*-D-ribofuranosyl}oxy)phosphonomethyl]phosphonic acid (4A.17)

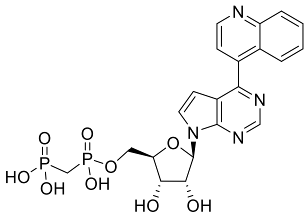

Compound **3** (69.7 mg, 0.16 mmol) was reacted with quinoline-4-boronic acid (40.8 mg, 0.24 mmol) for 5 min at 80 °C and 1 h at 65 °C according to the GP A. HPLC (C-18, H_2O + 0.05 % TFA/MeCN 0 → 80 %) gave product **4A.17** (28.4 mg, 34 %) as a yellow powder. ^{1}H NMR (500 MHz, D_2O): 2.31–2.50 (m, 2H, PCH$_2$P); 4.26 (ddd, 1H, J_{gem} = 11.7 Hz, $J_{5'a,P}$ = 5.5 Hz, $J_{5'a,4'}$ = 3.2 Hz, H-5′a); 4.30 (ddd, 1H, J_{gem} = 11.7 Hz, $J_{5'b,P}$ = 5.4 Hz, $J_{5'b,4'}$ = 2.7 Hz, H-5′b); 4.46 (m, 1H, H-

4′); 4.57 (dd, 1H, $J_{3',2'}$ = 5.0 Hz, $J_{3',4'}$ = 3.2 Hz, H-3′); 4.77 (bt, 1H, $J_{2',1'}$ = $J_{2',3'}$ = 5.6 Hz, H-2′); 6.49 (bs, 1H, H-5); 6.52 (d, 1H, $J_{1',2'}$ = 6.3 Hz, H-1′); 7.84 (dd, 1H, $J_{6,5}$ = 8.6 Hz, $J_{6,7}$ = 6.9 Hz, H-6-quinolinyl); 8.02 (bs, 1H, H-6); 8.10–8.15 (m, 2H, H-5,7-quinolinyl); 8.22 (d, 1H, $J_{3,2}$ = 5.0 Hz, H-3-quinolinyl); 8.33 (d, 1H, $J_{8,7}$ = 8.7 Hz, H-8-quinolinyl); 9.01 (s, 1H, H-2); 9.27 (bs, 1H, H-2-quinolinyl); ^{13}C NMR (125.7 MHz, D_2O): 28.4 (m, PCH_2P); 66.4 (d, $J_{C,P}$ = 4.7 Hz, CH_2-5′); 72.6 (CH-3′); 76.3 (CH-2′); 85.9 (d, $J_{C,P}$ = 7.7 Hz, CH-4′); 88.4 (CH-1′); 103.6 (CH-5); 121.0 (C-4a); 123.3 (CH-8-quinolinyl); 124.8 (CH-3-quinolinyl); 128.2 (C-4a-quinolinyl); 128.5 (CH-5/7-quinolinyl); 132.0 (CH-6); 132.6 (CH-6-quinolinyl); 137.2 (CH-5/7-quinolinyl); 140.4 (C-4-quinolinyl); 146.0 (CH-2-quinolinyl); 151.7 (CH-2); 152.3 (C-8a-quinolinyl); 153.3 (C-4); 154.0 (C-7a); ^{31}P NMR (202.4 MHz, D_2O): 16.35 and 19.36 (2×bs, 2×1P, PCH_2P). HR-ESI-MS: *found*: 535.0784 ($[M–H]^-$, calcd for $C_{21}H_{21}O_9N_4P_2^-$: 535.0789).

[(5-{[4-(Isoquinolin-8-yl)-7*H*-pyrrolo[2,3-*d*]pyrimidin-7-yl]-*β*-D-ribofuranosyl}oxy)phosphonomethyl]phosphonic acid (4A.18)

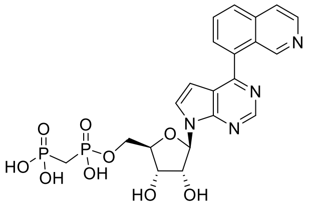

Compound **3** (80.9 mg, 0.18 mmol) was reacted with isoquinolin-8-ylboronic acid (47.3 mg, 0.27 mmol) for 5 min at 80 °C and 1 h at 65 °C according to the GP A. HPLC (C-18, H_2O + 0.05 % TFA/MeCN 0 → 80 %) gave product **4A.18** (33 mg, 34 %) as a yellow powder. ^{1}H NMR (500 MHz, DMSO-d$_6$): 2.13 (t, 2H, $J_{CH2,P}$ = 19.7 Hz, PCH_2P); 4.16 (ddd, 1H, J_{gem} = 11.5 Hz, $J_{5'a,P}$ = 5.0 Hz, $J_{5'a,4'}$ = 3.6 Hz, H-5′a); 4.23 (ddd, 1H, J_{gem} = 11.5 Hz, $J_{5'b,P}$ = 5.9 Hz, $J_{5'b,4'}$ = 3.5 Hz, H-5′b); 4.41 (bqd, 1H, $J_{4',5'a}$ = $J_{4',5'b}$ = $J_{4',3'}$ = 3.5 Hz, $J_{4',P}$ = 1.0 Hz, H-4′); 4.62 (dd, 1H, $J_{3',2'}$ = 5.3 Hz, $J_{3',4'}$ = 3.5 Hz, H-3′); 4.81 (dd, 1H, $J_{2',1'}$ = 6.2 Hz, $J_{2',3'}$ = 5.3 Hz, H-2′); 6.51 (d, 1H, $J_{1',2'}$ = 6.2 Hz, H-1′); 6.58 (d, 1H, $J_{5,6}$ = 3.8 Hz, H-5); 7.89 (d, 1H, $J_{6,5}$ = 3.9 Hz, H-6); 7.90–7.94 (m, 2H, H-6,7-isoquinolinyl); 7.94 (bd, 1H, $J_{4,3}$ = 5.8 Hz, H-4-isoquinolinyl); 8.12 (m, 1H, H-5-isoquinolinyl); 8.44 (d, 1H, $J_{3,4}$ = 5.8 Hz, H-3-isoquinolinyl); 8.87 (s, 1H, H-2); 9.21 (s, 1H, H-1-isoquinolinyl); ^{13}C NMR (125.7 MHz, DMSO-d$_6$): 30.3 (dd, $J_{C,P}$ = 125.9 and 120.2 Hz, PCH_2P); 65.7 (d, $J_{C,P}$ = 5.1 Hz, CH_2-5′); 72.6 (CH-3′); 76.0 (CH-2′); 85.8 (d, $J_{C,P}$ = 8.1 Hz, CH-4′); 88.22 (CH-1′); 104.4 (CH-5); 121.2 (C-4a); 123.7 (CH-4-isoquinolinyl); 127.3 (C-8a-isoquinolinyl); 129.7 (CH-6); 131.0 (CH-5-isoquinolinyl); 132.2 and 132.6 (CH-6,7-

isoquinolinyl); 136.0 (CH-8-isoquinolinyl); 138.5 (C-4a-isoquinolinyl); 143.5 (C-3-isoquinolinyl); 151.5 (CH-1-isoquinolinyl); 152.2 (CH-2); 153.6 (C-7a); 158.6 (C-4); ^{31}P NMR (202.4 MHz, DMSO-d$_6$): 21.68 and 13.21 (2×bd, 2×1P, $J_{P,P}$ = 8.9 Hz, PCH$_2$P). HR-ESI-MS: *found*: 535.0784 ([M–H]$^-$, calcd for $C_{21}H_{21}O_9N_4P_2^-$: 535.0789).

[(5-{[4-(Quinolin-6-yl)-7*H*-pyrrolo[2,3-*d*]pyrimidin-7-yl]-*β*-D-ribofuranosyl}oxy)phosphonomethyl]phosphonic acid (4A.19)

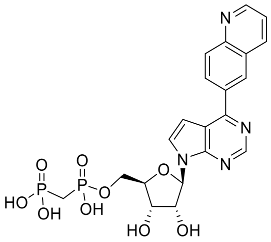

Compound **3** (88.5 mg, 0.20 mmol) was reacted with quinoline-6-boronic acid (51.8 mg, 0.30 mmol) for 30 min at 80 °C and 30 min at 70 °C according to the GP A. 2 HPLC purifications (C-18, H_2O + 0.05 % TFA/MeCN 0 → 80 %) gave product **4A.19** (25.9 mg, 24 %) as a yellow powder. ^{1}H NMR (500 MHz, DMSO-d$_6$): 2.27 (t, 2H, $J_{CH2,P}$ = 20.4 Hz, PCH$_2$P); 4.10–4.19 (m, 3H, H-5′,4′); 4.24 (dd, 1H, $J_{3',2'}$ = 5.1 Hz, $J_{3',4'}$ = 2.9 Hz, H-3′); 4.52 (t, 1H, $J_{2',1'}$ = $J_{2',3'}$ = 5.7 Hz, H-2′); 6.37 (d, 1H, $J_{1',2'}$ = 6.2 Hz, H-1′); 7.24 (d, 1H, $J_{5,6}$ = 3.8 Hz, H-5); 7.68 (dd, 1H, $J_{3,4}$ = 8.2 Hz, $J_{3,2}$ = 4.2 Hz, H-3-quinolinyl); 8.05 (d, 1H, $J_{6,5}$ = 3.8 Hz, H-6); 8.23 (d, 1H, $J_{8,7}$ = 8.8 Hz, H-8-quinolinyl); 8.60 (dd, 1H, $J_{7,8}$ = 8.8 Hz, $J_{7,5}$ = 2.0 Hz, H-7-quinolinyl); 8.71 (d, 1H, $J_{4,3}$ = 8.2 Hz, H-4-quinolinyl); 8.87 (d, 1H, $J_{5,7}$ = 2.0 Hz, H-5-quinolinyl); 8.98 (s, 1H, H-2); 9.03 (bs, 1H, H-2-quinolinyl); ^{13}C NMR (125.7 MHz, DMSO-d$_6$): 27.6 (t, $J_{C,P}$ = 128.4 Hz, PCH$_2$P); 64.8 (d, $J_{C,P}$ = 4.9 Hz, CH$_2$-5′); 70.4 (CH-3′); 73.8 (CH-2′); 82.9 (d, $J_{C,P}$ = 7.2 Hz, CH-4′); 86.7 (CH-1′); 101.5 (CH-5); 115.6 (C-4a); 122.2 (CH-3-quinolinyl); 128.0 (C-4a-quinolinyl); 128.3 (CH-6); 129.0 (CH-8-quinolinyl); 129.1 (CH-5-quinolinyl); 129.5 (CH-7-quinolinyl); 135.5 (C-6-quinolinyl); 138.0 (CH-4-quinolinyl); 147.6 (C-8a-quinolinyl); 151.1 (CH-2); 151.4 (CH-2-quinolinyl); 152.4 (C-7a); 155.0 (C-4); ^{31}P NMR (202.4 MHz, DMSO-d$_6$): 15.81 and 19.70 (2×bs, 2×1P, PCH$_2$P). HR-ESI-MS: *found*: 535.0784 ([M–H]$^-$, calcd for $C_{21}H_{21}O_9N_4P_2^-$: 535.0789).

[(5-{[4-(Quinolin-3-yl)-7*H*-pyrrolo[2,3-*d*]pyrimidin-7-yl]-*β*-D-ribofuranosyl}oxy)phosphonomethyl]phosphonic acid (4A.20)

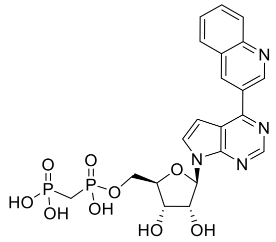

Compound **3** (88.8 mg, 0.20 mmol) was reacted with quinolin-3-ylboronic acid (51.9 mg, 0.30 mmol) for 30 min at 80 °C and 30 min at 70 °C according to the GP A. 2 HPLC purifications (C-18, H_2O + 0.05 % TFA/MeCN 0 → 80 %) gave product **4A.20** (41.3 mg, 38 %) as a yellow powder. ^{1}H NMR (500 MHz, DMSO-d$_6$): 2.28 (t, 2H, $J_{CH2,P}$ = 20.5 Hz, PCH_2P); 4.10–4.20 (m, 3H, H-5′,4′); 4.21 (dd, 1H, $J_{3',2'}$ = 5.1 Hz, $J_{3',4'}$ = 3.0 Hz, H-3′); 4.52 (dd, 1H, $J_{2',1'}$ = 6.2 Hz, $J_{2',3'}$ = 5.1 Hz, H-2′); 6.37 (d, 1H, $J_{1',2'}$ = 6.2 Hz, H-1′); 7.28 (d, 1H, $J_{5,6}$ = 3.8 Hz, H-5); 7.75 (ddd, 1H, $J_{6,5}$ = 8.2 Hz, $J_{6,7}$ = 6.9 Hz, $J_{6,8}$ = 1.2 Hz, H-6-quinolinyl); 7.91 (ddd, 1H, $J_{7,8}$ = 8.4 Hz, $J_{7,6}$ = 6.9 Hz, $J_{7,5}$ = 1.5 Hz, H-7-quinolinyl); 8.07 (d, 1H, $J_{6,5}$ = 3.8 Hz, H-); 8.14 (bd, 1H, $J_{8,7}$ = 8.4 Hz, H-8-quinolinyl); 8.30 (dd, 1H, $J_{5,6}$ = 8.2 Hz, $J_{5,7}$ = 1.5 Hz, H-5-quinolinyl); 9.01 (s, 1H, H-2); 9.21 (d, 1H, $J_{4,2}$ = 2.2 Hz, H-4-quinolinyl); 9.70 (d, 1H, $J_{2,4}$ = 2.2 Hz, H-2-quinolinyl); ^{13}C NMR (125.7 MHz, DMSO-d$_6$): 27.6 (t, $J_{C,P}$ = 128.7 Hz, PCH_2P); 64.8 (d, $J_{C,P}$ = 5.3 Hz, CH_2-5′); 70.4 (CH-3′); 73.8 (CH-2′); 82.9 (d, $J_{C,P}$ = 7.3 Hz, CH-4′); 86.5 (CH-1′); 101.3 (CH-5); 115.8 (C-4a); 127.3 (C-4a-quinolinyl); 127.5 (CH-6-quinolinyl); 128.3 (CH-8-quinolinyl); 128.6 (CH-6); 129.4 (CH-5-quinolinyl); 130.2 (C-3-quinolinyl); 131.2 (CH-7-quinolinyl); 136.8 (CH-4-quinolinyl); 147.5 (C-8a-quinolinyl); 149.6 (C-2-quinolinyl); 151.1 (CH-2); 152.3 (C-7a); 153.3 (C-4); ^{31}P NMR (202.4 MHz, DMSO-d$_6$): 15.73 and 19.82 (2×d, 2×1P, $J_{P,P}$ = 8.4 Hz, PCH_2P). HR-ESI-MS: *found*: 535.0786 ($[M–H]^-$, calcd for $C_{21}H_{21}O_9N_4P_2^-$: 535.0789).

[(5-{[4-(Isoquinolin-6-yl)-7*H*-pyrrolo[2,3-*d*]pyrimidin-7-yl]-*β*-D-ribofuranosyl}oxy)phosphonomethyl]phosphonic acid (4A.21)

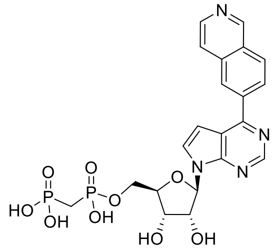

Compound **3** (83.1 mg, 0.19 mmol) was reacted with isoquinolin-6-ylboronic acid (48.6 mg, 0.28 mmol) for 1 h at 80 °C according to the GP A. 3 HPLC purifications (C-18, H_2O + 0.05 % TFA/MeCN 0 → 80 %) gave product **4A.21** (32.3 mg, 32 %) as a yellow powder. ^{1}H NMR (500

MHz, DMSO-d6): 2.26 (t, 2H, $J_{CH2,P}$ = 20.3 Hz, PCH_2P); 4.08–4.19 (m, 3H, H-5′,4′); 4.24 (dd, 1H, $J_{3',2'}$ = 5.1 Hz, $J_{3',4'}$ = 2.8 Hz, H-3′); 4.53 (dd, 1H, $J_{2',1'}$ = 6.2 Hz, $J_{2',3'}$ = 5.1 Hz, H-2′); 6.37 (d, 1H, $J_{1',2'}$ = 6.2 Hz, H-1′); 7.21 (d, 1H, $J_{5,6}$ = 3.8 Hz, H-5); 8.08 (d, 1H, $J_{6,5}$ = 3.8 Hz, H-6); 8.20 (d, 1H, $J_{4,3}$ = 5.8 Hz, H-4-isoquinolinyl); 8.37 (d, 1H, $J_{8,7}$ = 8.6 Hz, H-8-isoquinolinyl); 8.51 (dd, 1H, $J_{7,8}$ = 8.6 Hz, $J_{7,5}$ = 1.7 Hz, H-7-isoquinolinyl); 8.63 (d, 1H, $J_{3,4}$ = 5.8 Hz, H-3-isoquinolinyl); 8.84 (bd, 1H, $J_{5,7}$ = 1.6 Hz, H-5-isoquinolinyl); 9.00 (s, 1H, H-2); 9.52 (bs, 1H, H-1-isoquinolinyl); ^{13}C NMR (125.7 MHz, DMSO-d6): 27.5 (t, $J_{C,P}$ = 128.0 Hz, PCH_2P); 64.7 (d, $J_{C,P}$ = 5.1 Hz, CH_2-5′); 70.5 (CH-3′); 73.8 (CH-2′); 83.0 (d, $J_{C,P}$ = 7.3 Hz, CH-4′); 86.4 (CH-1′); 101.4 (CH-5); 115.9 (C-4a); 122.1 (CH-4-isoquinolinyl); 127.3 (CH-5-isoquinolinyl); 127.7 (CH-7-isoquinolinyl); 128.3 (C-8a-isoquinolinyl); 128.7 (CH-6); 128.8 (CH-8-isoquinolinyl); 135.8 (C-4a-isoquinolinyl); 139.9 (C-6-isoquinolinyl); 141.7 (CH-3-isoquinolinyl); 151.1 (CH-2); 151.5 (CH-1-isoquinolinyl); 152.5 (C-7a); 154.7 (C-4); ^{31}P NMR (202.4 MHz, DMSO-d6): 15.99 and 19.43 (2×bd, 2×1P, $J_{P,P}$ = 7.5 Hz, PCH_2P). HR-ESI-MS: *found*: 535.0786 ([M–H]$^-$, calcd for $C_{21}H_{21}O_9N_4P_2^-$: 535.0789).

[(5-{[4-(Quinazolin-7-yl)-7*H*-pyrrolo[2,3-*d*]pyrimidin-7-yl]-*β*-D-ribofuranosyl}oxy)phosphonomethyl]phosphonic acid (4A.22)

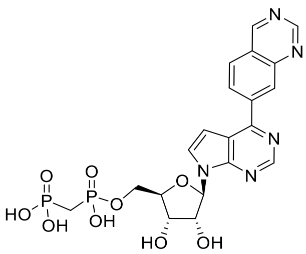

Compound **3** (82.1 mg, 0.19 mmol) was reacted with quinazolin-7-boronic acid (48.3 mg, 0.28 mmol) for 30 min at 80 °C and 30 min at 70 °C according to the GP A. 3 HPLC purifications (C-18, H_2O + 0.05 % TFA/MeCN 0 → 80 %) and lyophilization from H_2O/*t*-BuOH gave product **4A.22** (28 mg, 28 %) as a yellow powder. ^{1}H NMR (500 MHz, DMSO-d6): 2.28 (t, 2H, $J_{CH2,P}$ = 20.2 Hz, PCH_2P); 4.10–4.19 (m, 3H, H-5′,4′); 4.24 (dd, 1H, $J_{3',2'}$ = 5.2 Hz, $J_{3',4'}$ = 2.9 Hz, H-3′); 4.52 (t, 1H, $J_{2',1'}$ = $J_{2',3'}$ = 5.7 Hz, H-2′); 6.37 (d, 1H, $J_{1',2'}$ = 6.2 Hz, H-1′); 7.13 (d, 1H, $J_{5,6}$ = 3.8 Hz, H-5); 8.09 (d, 1H, $J_{6,5}$ = 3.8 Hz, H-6); 8.39 (d, 1H, $J_{5,6}$ = 8.5 Hz, H-5-quinazolinyl); 8.53 (dd, 1H, $J_{6,5}$ = 8.5 Hz, $J_{6,8}$ = 1.7 Hz, H-6-quinazolinyl); 8.70(bd, 1H, $J_{8,6}$ = 1.6 Hz, H-8-quinazolinyl); 9.03 (s, 1H, H-2); 9.42 (bs, 1H, H-2-quinazolinyl); 9.76 (bs, 1H, H-4-quinazolinyl); ^{13}C NMR (125.7 MHz, DMSO-d6): 27.5 (bt, $J_{C,P}$ = 127.4 Hz, PCH_2P); 64.8 (bd, $J_{C,P}$ = 3.4 Hz, CH_2-5′); 70.4

(CH-3′); 73.8 (CH-2′); 83.0 (d, $J_{C,P}$ = 6.5 Hz, CH-4′); 86.5 (CH-1′); 101.0 (CH-5); 116.1 (C-4a); 125.2 (CH-4-quinazolinyl); 127.8 (CH-8-quinazolinyl); 127.9 (CH-6-quinazolinyl); 128.7 (CH-5-quinazolinyl); 129.0 (CH-6); 142.9 (C-7-quinazolinyl); 149.3 (C-8a-quinazolinyl); 151.2 (CH-2); 152.5 (C-7a); 154.4 (C-4); 155.8 (CH-2-quinazolinyl); 160.9 (CH-4-quinazolinyl); ^{31}P NMR (202.4 MHz, DMSO-d_6): 15.72 and 19.88 (2×bs, 2×1P, PCH_2P). HR-ESI-MS: *found*: 536.0737 ($[M–H]^–$, calcd for $C_{20}H_{20}O_9N_5P_2^–$: 536.0742).

2-Chloro-4-(naphth-1-yl)-7-(*β*-D-ribofuranosyl)-7*H*-pyrrolo[2,3-*d*]pyrimidine (6B.6)

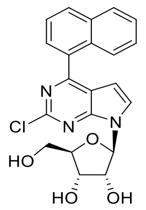

Compound **5** (81.8 mg, 0.26 mmol) was reacted with naphthalene-1-boronic acid (48.3 mg, 0.28 mmol) for 10 min at 100 °C according to the GP A. HPFC (SiO_2, DCM/MeOH 1:0 → 9:1) gave **20d** (92.8 mg, 88 %) as a white powder. ^{1}H NMR (500 MHz, DMSO-d_6): 3.58 (ddd, 1H, J_{gem} = 11.9 Hz, $J_{5'a,OH}$ = 5.4 Hz, $J_{5'a,4'}$ = 3.9 Hz, H-5′a); 3.65 (ddd, 1H, J_{gem} = 11.9 Hz, $J_{5'b,OH}$ = 5.4 Hz, $J_{5'b,4'}$ = 4.1 Hz, H-5′b); 3.97 (q, 1H, $J_{4',5'a} = J_{4',5'b} = J_{4',3'}$ = 3.9 Hz, H-4′); 4.13 (td, 1H, $J_{3',2'} = J_{3',OH}$ = 5.0 Hz, $J_{3',4'}$ = 2.9 Hz, H-3′); 4.47 (td, 1H, $J_{2',1'} = J_{2',OH}$ = 6.4 Hz, $J_{2',3'}$ = 5.0 Hz, H-2′); 5.05 (t, 1H, $J_{OH,5'a} = J_{OH,5'b}$ = 5.3 Hz, OH-5′); 5.27 (d, 1H, $J_{OH,3'}$ = 4.9 Hz, OH-3′); 5.47 (d, 1H, $J_{OH,2'}$ = 6.4 Hz, OH-2′); 6.23 (d, 1H, $J_{1',2'}$ = 6.4 Hz, H-1′); 6.49 (d, 1H, $J_{5,6}$ = 3.8 Hz, H-5); 7.55 (ddd, 1H, $J_{7,8}$ = 8.5 Hz, $J_{7,6}$ = 6.8 Hz, $J_{7,5}$ = 1.5 Hz, H-7-naphthyl); 7.61 (ddd, 1H, $J_{6,5}$ = 8.2 Hz, $J_{6,7}$ = 6.8 Hz, $J_{6,8}$ = 1.3 Hz, H-6-naphthyl); 7.70 (dd, 1H, $J_{3,4}$ = 8.2 Hz, $J_{3,2}$ = 7.1 Hz, H-3-naphthyl); 7.79 (dd, 1H, $J_{2,3}$ = 7.1 Hz, $J_{2,4}$ = 1.3 Hz, H-2-naphthyl); 7.95 (d, 1H, $J_{6,5}$ = 3.8 Hz, H-6); 8.01 (bd, 1H, $J_{8,7}$ = 8.5 Hz, H-8-naphthyl); 8.08 (bd, 1H, $J_{4,5}$ = 8.3 Hz, H-5-naphthyl); 8.16 (bd, 1H, $J_{4,3}$ = 8.2 Hz, H-4-naphthyl); ^{13}C NMR (125.7 MHz, DMSO-d_6): 61.5 (CH_2-5′); 70.7 (CH-3′); 74.1 (CH-2′); 85.6 (CH-4′); 86.6 (CH-1′); 101.4 (CH-5); 117.4 (C-4a); 125.2 (CH-8-naphthyl); 125.4 (CH-3-naphthyl); 126.4 (CH-6-naphthyl); 127.0 (CH-7-naphthyl); 128.4 (CH-2-naphthyl); 128.5 (CH-5-naphthyl); 128.7 (CH-6); 130.1 (C-8a-naphthyl); 130.4 (CH-4-naphthyl); 133.53 and 133.47 (C-1,4a-naphthyl); 152.3 (C-2); 152.9 (C-7a); 160.0 (C-4). HR-ESI-MS: *found*: 412.1059 ($[M + H]^+$, calcd for $C_{21}H_{19}O_4N_3Cl^+$: 412.1059); HR-ESI-MS: *found*: 434.0879 ($[M + Na]^+$, calcd for $C_{21}H_{18}O_4N_3ClNa^+$: 434.0878).

2-Chloro-4-(naphthalen-2-yl)-7-(*β*-D-ribofuranosyl)-7*H*-pyrrolo[2,3-*d*]pyrimidine (6B.7)

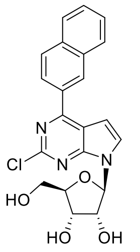

Nucleoside **5** (81.1 mg, 0.25 mmol) was reacted with naphthalene-2-boronic acid (47.9 mg, 0.28 mmol) for 10 min at 100 °C according to the GP A. HPFC (SiO_2, DCM/MeOH 1:0 → 9:1) gave **6B.7** (90.8 mg, 87 %) as a white powder. ^{1}H NMR (500 MHz, DMSO-d_6): 3.59 (ddd, 1H, J_{gem} = 11.8 Hz, $J_{5'a,OH}$ = 5.5 Hz, $J_{5'a,4'}$ = 3.9 Hz, H-5´a); 3.66 (ddd, 1H, J_{gem} = 11.8 Hz, $J_{5'b,OH}$ = 5.5 Hz, $J_{5'b,4'}$ = 4.1 Hz, H-5´b); 3.97 (q, 1H, $J_{4',5'a}$ = $J_{4',5'b}$ = $J_{4',3'}$ = 3.9 Hz, H-4´); 4.14 (td, 1H, $J_{3',2'}$ = $J_{3',OH}$ = 5.0 Hz, $J_{3',4'}$ = 3.1 Hz, H-3´); 4.46 (td, 1H, $J_{2',1'}$ = $J_{2',OH}$ = 6.3 Hz, $J_{2',3'}$ = 5.0 Hz, H-2´); 5.07 (t, 1H, $J_{OH,5'a}$ = $J_{OH,5'b}$ = 5.5 Hz, OH-5´); 5.25 (d, 1H, $J_{OH,3'}$ = 4.9 Hz, OH-3´); 5.46 (d, 1H, $J_{OH,2'}$ = 6.3 Hz, OH-2´); 6.22 (d, 1H, $J_{1',2'}$ = 6.2 Hz, H-1´); 7.27 (d, 1H, $J_{5,6}$ = 3.9 Hz, H-5); 7.63 (m; 1H, H-7-naphthyl); 7.66 (m, 1H, H-6-naphthyl); 8.04 (m, 1H, H-5-naphthyl); 8.06 (d, 1H, $J_{6,5}$ = 3.9 Hz, H-6); 8.14 (d, 1H, $J_{4,3}$ =8.6 Hz, H-4-naphthyl); 8.22 (m, 1H, H-8-naphthyl); 8.27 (dd, 1H, $J_{3,4}$ = 8.6 Hz, $J_{3,1}$ = 1.8 Hz, H-3-naphthyl); 8.77 (d, 1H, $J_{1,3}$ = 1.8 Hz, H-1-naphthyl); ^{13}C NMR (125.7 MHz, DMSO-d_6): 61.5 (CH_2-5´); 70.6 (CH-3´); 74.1 (CH-2´); 85.5 (CH-4´); 86.6 (CH-1´); 101.9 (CH-5); 114.8 (C-4a); 125.4 (CH-3-naphthyl); 126.8 (CH-7-naphthyl); 127.7 (CH-5-naphthyl); 127.9 (CH-6-naphthyl); 128.7 (CH-4-naphthyl); 128.9 (CH-6); 129.2 and 129.3 (CH-1,8-naphthyl); 132.3 (C-8a-naphthyl); 133.6 (C-2-naphthyl); 134.0 (C-4a-naphthyl); 152.5 (C-2); 153.5 (C-7a); 158.1 (C-4). HR-ESI-MS: *found*: 412.1057 ($[M + H]^+$, calcd for $C_{21}H_{19}O_4N_3Cl^+$: 412.1059); HR-ESI-MS: *found*: 434.0876 ($[M + Na]^+$, calcd for $C_{21}H_{18}O_4N_3ClNa^+$: 434.0878).

2-Chloro-4-(benzofuran-2-yl)-7-(*β*-D-ribofuranosyl)-7*H*-pyrrolo-[2,3-*d*]pyrimidine (6B.8)

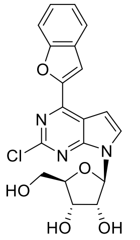

Nucleoside **5** (74.3 mg, 0.23 mmol) was reacted with benzofuran-2-ylboronic acid (41.3 mg, 0.26 mmol) for 20 min at 100 °C according to the GP A. HPFC (SiO_2, DCM/MeOH 1:0 → 9:1) gave **6B.8** (70.4 mg, 76 %) as a pale-yellow solid. ^{1}H NMR spectra is in agreement with the literature.[2]

2-Chloro-4-(5,6,7,8-tetrahydronaphth-1-yl)-7-(*β*-D-ribofuranosyl)-7*H*-pyrrolo[2,3-*d*]pyrimidine (6B.12)

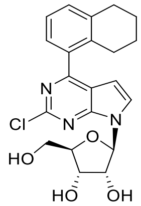

Nucleoside **5** (91.8 mg, 0.29 mmol) was reacted with 5,6,7,8-tetrahydronaphth-1-ylboronic acid (55.5 mg, 0.32 mmol) for 1 h at 100 °C according to the GP A. HPFC (SiO_2, DCM/MeOH 1:0 → 9:1) gave **6B.12** (90.2 mg, 76 %) as a white powder. ^1H NMR (500 MHz, DMSO-d_6): 1.65 (m, 2H, H-3-$C_{10}H_{11}$); 1.75 (m, 2H, H-2-$C_{10}H_{11}$); 2.64 (t, 2H, $J_{4,3}$ = 6.4 Hz, H-4-$C_{10}H_{11}$); 2.84 (t, 2H, $J_{1,2}$ = 6.4 Hz, H-1-$C_{10}H_{11}$); 3.56 (ddd, 1H, J_{gem} = 11.8 Hz, $J_{5'a,OH}$ = 5.4 Hz, $J_{5'a,4'}$ = 3.9 Hz, H-5′a); 3.63 (ddd, 1H, J_{gem} = 11.8 Hz, $J_{5'b,OH}$ = 5.4 Hz, $J_{5'b,4'}$ = 4.2 Hz, H-5′a); 3.95 (td, 1H, $J_{4',5'a} = J_{4',5'b}$ = 4.0 Hz, $J_{4',3'}$ = 2.8 Hz, H-4′); 4.12 (td, 1H, $J_{3',2'} = J_{3',OH}$ = 4.9 Hz, $J_{3',4'}$ = 2.8 Hz, H-3′); 4.45 (td, 1H, $J_{2',1'} = J_{2',OH}$ = 6.4 Hz, $J_{2',3'}$ = 5.0 Hz, H-2′); 5.04 (t, 1H, $J_{OH,5'a} = J_{OH,5'b}$ = 5.4 Hz, OH-5′); 5.25 (d, 1H, $J_{OH,3'}$ = 4.8 Hz, OH-3′); 5.44 (d, 1H, $J_{OH,2'}$ = 6.4 Hz, OH-2′); 6.17 (d, 1H, $J_{1',2'}$ = 6.4 Hz, H-1′); 6.50 (d, 1H, $J_{5,6}$ = 3.8 Hz, H-5); 7.21–7.30 (m, 3H, H-6,7,8-$C_{10}H_{11}$); 7.91 (d, 1H, $J_{6,5}$ = 3.8 Hz, H-6); ^{13}C NMR (125.7 MHz, DMSO-d_6): 22.2 (CH_2-2-$C_{10}H_{11}$); 22.5 (CH_2-3-$C_{10}H_{11}$); 26.8 (CH_2-4-$C_{10}H_{11}$); 29.3 (CH_2-1-$C_{10}H_{11}$); 61.5 (CH_2-5′); 70.7 (CH-3′); 74.1 (CH-2′); 85.6 (CH-4′); 86.6 (CH-1′); 101.4 (CH-5); 116.8 (C-4a); 125.4 (CH-7-$C_{10}H_{11}$); 126.9 (CH-6-$C_{10}H_{11}$); 128.5 (CH-6); 130.6 (CH-8-$C_{10}H_{11}$); 135.0 (C-4a-$C_{10}H_{11}$); 135.6 (C-5-$C_{10}H_{11}$); 137.8 (C-5-$C_{10}H_{11}$); 151.9 (C-2); 152.7 (C-7a); 161.5 (C-4). HR-ESI-MS: *found*: 416.1371 ($[M + H]^+$, calcd for $C_{21}H_{23}O_4N_3Cl^+$: 416.1372); HR-ESI-MS: *found*: 438.1190 ($[M + Na]^+$, calcd for $C_{21}H_{22}O_4N_3ClNa^+$: 438.1191).

2-Chloro-4-(5,6,7,8-tetrahydronaphthalen-2-yl)-7-(*β*-D-ribofuranosyl)-7*H*-pyrrolo[2,3-*d*]pyrimidine (6B.23)

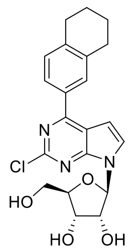

Nucleoside **5** (83.5 mg, 0.26 mmol) was reacted with 5,6,7,8-tetrahydronaphthalen-2-ylboronic acid (50.5 mg, 0.29 mmol) for 1 h at 100 °C according to the GP A. 2 HPFC purifications (SiO_2, DCM/MeOH 1:0 → 9:1) gave **6B.23** (65.0 mg, 60 %) as a white powder. ^{1}H NMR (500 MHz, DMSO-d_6): 1.74–1.82 (m, 4H, H-2,3-$C_{10}H_{11}$); 2.81 and 2.86 (2×m, 2×2H, H-1,4-$C_{10}H_{11}$); 3.57 (ddd, 1H, J_{gem} = 11.9 Hz, $J_{5'a,OH}$ = 5.5 Hz, $J_{5'a,4'}$ = 3.9 Hz, H-5′a); 3.64 (ddd, 1H, J_{gem} = 11.9 Hz, $J_{5'b,OH}$ = 5.4 Hz, $J_{5'b,4'}$ = 4.1 Hz, H-5′a); 3.95 (q, 1H, $J_{4',5'a}$ = $J_{4',5'b}$ = $J_{4',3'}$ = 3.9 Hz, H-4′); 4.12 (td, 1H, $J_{3',2'}$ = $J_{3',OH}$ = 5.0 Hz, $J_{3',4'}$ = 3.0 Hz, H-3′); 4.43 (td, 1H, $J_{2',1'}$ = $J_{2',OH}$ = 6.3 Hz, $J_{2',3'}$ = 5.0 Hz, H-2′); 5.06 (m, 1H, OH-5′); 5.24 (d, 1H, OH-3′); 5.43 (bd, 1H, $J_{OH,2'}$ = 6.4 Hz, OH-2′); 6.17 (d, 1H, $J_{1',2'}$ = 6.2 Hz, H-1′); 7.06 (d, 1H, $J_{5,6}$ = 3.9 Hz, H-5); 7.28 (d, 1H, $J_{8,7}$ = 8.0 Hz, H-8-$C_{10}H_{11}$); 7.83 (bs, 1H, H-5-$C_{10}H_{11}$); 7.85 (dd, 1H, $J_{7,8}$ = 8.0 Hz, $J_{7,5}$ = 2.0 Hz, H-7-$C_{10}H_{11}$); 7.97 (d, 1H, $J_{6,5}$ = 3.9 Hz, H-6); ^{13}C NMR (125.7 MHz, DMSO-d_6): 22.55 and 22.63 (CH_2-2,3-$C_{10}H_{11}$); 28.8 and 28.9 (CH_2-1,4-$C_{10}H_{11}$); 61.5 (CH_2-5′); 70.6 (CH-3′); 74.1 (CH-2′); 85.5 (CH-4′); 86.6 (CH-1′); 101.8 (CH-5); 114.3 (C-4a); 125.9 (CH-7-$C_{10}H_{11}$); 128.5 (CH-6); 129.3 (CH-5-$C_{10}H_{11}$); 129.7 (CH-8-$C_{10}H_{11}$); 133.4 (C-6-$C_{10}H_{11}$); 137.5 (C-4a-$C_{10}H_{11}$); 140.4 (C-8a-$C_{10}H_{11}$); 152.5 (C-2); 153.4 (C-7a); 158.5 (C-4). HR-ESI-MS: *found*: 416.1371 ([M + H]$^+$, calcd for $C_{21}H_{23}O_4N_3Cl^+$: 416.1372); HR-ESI-MS: *found*: 438.1189 ([M + Na]$^+$, calcd for $C_{21}H_{22}O_4N_3ClNa^+$: 438.1191).

2-Chloro-4-(1*H*-indol-2-yl)-7-(*β*-D-ribofuranosyl)-7*H*-pyrrolo[2,3-*d*]pyrimidine (6B.24)

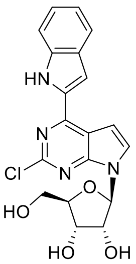

Nucleoside **5** (82.3 mg, 0.26 mmol) was reacted with 2-(4,4,5,5-tetramethyl-1,3,2-dioxaborolan-2-yl)-1*H*-indole (68.7 mg, 0.28 mmol) for 1 h at 100 °C according to the GP A. HPFC (SiO_2, DCM/MeOH 1:0 → 9:1) gave **6B.24** (101.5 mg, 98 %) as a pale-yellow powder. ^{1}H NMR (500 MHz, DMSO-d_6): 3.58 (ddd, 1H, (m, 3H, J_{gem} = 11.8 Hz, $J_{5'a,OH}$ = 5.4 Hz, $J_{5'a,4'}$ = 3.9 Hz, H-5′a); 3.66 (dd, 1H, J_{gem} = 11.8 Hz, $J_{5'b,OH}$ = 5.5 Hz, $J_{5'b,4'}$ = 4.1 Hz, H-5′b); 3.95 (q, 1H, $J_{4',5'a}$ = $J_{4',5'b}$ = $J_{4',3'}$ = 3.9 Hz, H-4′); 4.13 (td, 1H, $J_{3',2'}$ = $J_{3',OH}$ = 4.9 Hz, $J_{3',4'}$ = 3.0 Hz, H-3′); 4.44 (td, 1H, $J_{2',1'}$ = $J_{2',OH}$ = 6.3 Hz, $J_{2',3'}$ = 4.9 Hz, H-2′); 5.07 (t, 1H, $J_{OH,5'a}$ = $J_{OH,5'b}$ = 5.4 Hz, OH-5′); 5.24 (d, 1H, $J_{OH,3'}$ = 4.9 Hz, OH-3′); 5.44 (d, 1H, $J_{OH,2'}$ = 6.3 Hz, OH-2′); 6.17 (d, 1H, $J_{1',2'}$ = 6.3 Hz, H-1′); 7.08 (ddd, 1H, $J_{5,4}$ = 8.0 Hz, $J_{5,6}$ = 6.9 Hz, $J_{5,7}$ = 1.0 Hz, H-5-indolyl); 7.24 (ddd, 1H,

$J_{6,7}$ = 8.3 Hz, $J_{6,5}$ = 6.9 Hz, $J_{6,4}$ = 1.2 Hz, H-6-indolyl); 7.31 (d, 1H, $J_{5,6}$ = 3.8 Hz, H-5); 7.58 (bdq, 1H, $J_{7,6}$ = 8.3 Hz, $J_{7,5}$ = $J_{7,4}$ = $J_{7,3}$ = 1.0 Hz, H-7-indolyl); 7.65 (bd, 1H, $J_{3,NH}$ = 2.3 Hz, $J_{3,7}$ = 0.9 Hz, H-3-indolyl); 7.68 (bdq, 1H, $J_{4,5}$ = 8.0 Hz, $J_{4,6}$ = $J_{4,7}$ = $J_{4,3}$ = 1.0 Hz, H-4-indolyl); 8.00 (d, 1H, $J_{6,5}$ = 3.8 Hz, H-6); 11.86 (d, 1H, $J_{NH,3}$ = 2.0 Hz, NH); ^{13}C NMR (125.7 MHz, DMSO-d$_6$): 61.5 (CH_2-5′); 70.6 (CH-3′); 74.1 (CH-2′); 85.47 (CH-4′); 86.5 (CH-1′); 101.6 (CH-5); 107.1 (CH-3-indolyl); 112.7 (CH-7-indolyl); 112.7 (C-4a); 120.1 (CH-5-indolyl); 121.5 (CH-4-indolyl); 124.2 (CH-6-indolyl); 128.3 (C-3a-indolyl); 128.6 (CH-6); 133.2 (C-2-indolyl); 137.6 (C-7a-indolyl); 150.6 (C-4); 152.2 (C-2); 153.2 (C-7a). HR-ESI-MS: *found*: 401.1012 ([M + H]$^+$, calcd for $C_{19}H_{18}O_4N_4Cl^+$: 401.1011); HR-ESI-MS: *found*: 423.0832 ([M + Na]$^+$, calcd for $C_{19}H_{17}O_4N_4ClNa^+$: 423.0831).

2-Chloro-4-(6-fluoronaphthalen-2-yl)-7-(*β*-D-ribofuranosyl)-7*H*-pyrrolo[2,3-*d*]pyrimidine (6B.25)

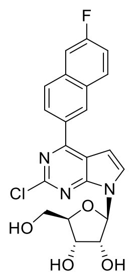

Nucleoside **5** (80.4 mg, 0.25 mmol) was reacted with 6-fluoronaphthalene-2-boronic acid (52.5 mg, 0.28 mmol) for 1 h at 100 °C according to the GP A. HPFC (SiO_2, DCM/MeOH 1:0 → 9:1) gave **6B.25** (82.7 mg, 77 %) as a white powder. ^{1}H NMR (500 MHz, DMSO-d$_6$): 3.58 (ddd, 1H, J_{gem} = 11.9 Hz, $J_{5'a,OH}$ = 5.3 Hz, $J_{5'a,4'}$ = 4.0 Hz, H-5′a); 3.67 (ddd, 1H, J_{gem} = 11.9 Hz, $J_{5'b,OH}$ = 5.4 Hz, $J_{5'b,4'}$ = 4.1 Hz, H-5′b); 3.97 (q, 1H, $J_{4',5'a}$ = $J_{4',5'b}$ = $J_{4',3'}$ = 3.8 Hz, H-4′); 4.14 (td, 1H, $J_{3',2'}$ = $J_{3',OH}$ = 4.9 Hz, $J_{3',4'}$ = 3.1 Hz, H-3′); 4.46 (td, 1H, $J_{2',1'}$ = $J_{2',OH}$ = 6.2 Hz, $J_{2',3'}$ = 4.9 Hz, H-2′); 5.07 (t, 1H, $J_{OH,5'a}$ = $J_{OH,5'b}$ = 5.4 Hz, OH-5′); 5.26 (d, 1H, $J_{OH,3'}$ = 4.9 Hz, OH-3′); 5.46 (d, 1H, $J_{OH,2'}$ = 6.3 Hz, OH-2′); 6.21 (d, 1H, $J_{1',2'}$ = 6.2 Hz, H-1′); 7.28 (d, 1H, $J_{5,6}$ = 3.9 Hz, H-5); 7.56 (td; 1H, $J_{7,8}$ = $J_{7,F}$ = 8.9 Hz, $J_{7,5}$ = 2.6 Hz, H-7-naphthyl); 7.85 (dd, 1H, $J_{5,F}$ = 10.2 Hz, $J_{5,7}$ = 2.6 Hz, H-5-naphthyl); 8.06 (d, 1H, $J_{6,5}$ = 3.9 Hz, H-6); 8.13 (d, 1H, $J_{4,3}$ =8.7 Hz, H-4-naphthyl); 8.32 (dd, 1H, $J_{3,4}$ = 8.7 Hz, $J_{3,1}$ = 1.8 Hz, H-3-naphthyl); 8.34 (dd, 1H, $J_{8,7}$ = 9.1 Hz, $J_{8,F}$ = 5.9 Hz, H-8-naphthyl); 8.82 (d, 1H, $J_{1,3}$ = 1.8 Hz, H-1-naphthyl); ^{13}C NMR (125.7 MHz, DMSO-d$_6$): 61.5 (CH_2-5′); 70.6 (CH-3′); 74.1 (CH-2′); 85.5 (CH-4′); 86.6 (CH-1′); 101.9 (CH-5); 110.9 (d, $J_{C,F}$ = 20.7 Hz, CH-5-naphthyl); 114.7 (C-4a); 117.072 (d, $J_{C,F}$ = 25.4 Hz, CH-7-naphthyl); 126.5 (CH-

3-naphthyl); 128.2 (d, $J_{C,F}$ = 5.3 Hz, CH-4-naphthyl); 129.0 (CH-6); 129.3 (CH-1-naphthyl); 130.0 (C-8a-naphthyl); 132.4 (d, $J_{C,F}$ = 9.3 Hz, CH-8-naphthyl); 133.1 (d, $J_{C,F}$ = 2.6 Hz, C-2-naphthyl); 135.0 (d, $J_{C,F}$ = 10.0 Hz, C-4a-naphthyl); 152.5 (C-2); 153.6 (C-7a); 157.8 (C-4); 161.1 (d, $J_{C,F}$ = 246.5 Hz, C-6-naphthyl); ^{19}F NMR (470.4 MHz, DMSO-d$_6$): -107.51 (td, 1F, $J_{F,5}$ = $J_{F,7}$ = 9.4 Hz, $J_{F,8}$ = 5.9 Hz, F-6). HR-ESI-MS: *found*: 430.0963 ([M + H]$^+$, calcd for $C_{21}H_{18}O_4N_3ClF^+$: 430.0964); HR-ESI-MS: *found*: 452.0781 ([M + Na]$^+$, calcd for $C_{21}H_{17}O_4N_3ClFNa^+$: 452.0784).

4-(4-Bromo-1-fluoronaphthalen-2-yl)-2-chloro-7-(*β*-D-ribofuranosyl)-7*H*-pyrrolo[2,3-*d*]pyrimidine (6B.26)

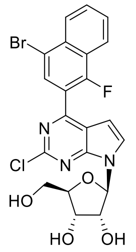

Nucleoside **5** (81.0 mg, 0.25 mmol) was reacted with 4-bromo-1-fluoronaphthalene-2-boronic acid (74.8 mg, 0.28 mmol) for 1 h at 100 °C according to the GP A. 2 HPFC purifications (SiO_2, DCM/MeOH 1:0 → 9:1) gave **6B.26** (79.9 mg, 62 %) as a white powder. ^{1}H NMR (500 MHz, DMSO-d$_6$): 3.58 (ddd, 1H, J_{gem} = 11.9 Hz, $J_{5'a,OH}$ = 5.5 Hz, $J_{5'a,4'}$ = 3.9 Hz, H-5′a); 3.65 (ddd, 1H, J_{gem} = 11.9 Hz, $J_{5'b,OH}$ = 5.4 Hz, $J_{5'b,4'}$ = 4.1 Hz, H-5′b); 3.97 (q, 1H, $J_{4',5'a}$ = $J_{4',5'b}$ = $J_{4',3'}$ = 3.9 Hz, H-4′); 4.13 (td, 1H, $J_{3',2'}$ = $J_{3',OH}$ = 4.9 Hz, $J_{3',4'}$ = 2.9 Hz, H-3′); 4.46 (td, 1H, $J_{2',1'}$ = $J_{2',OH}$ = 6.4 Hz, $J_{2',3'}$ = 4.9 Hz, H-2′); 5.04 (t, 1H, $J_{OH,5'a}$ = $J_{OH,5'b}$ = 5.4 Hz, OH-5′); 5.27 (d, 1H, $J_{OH,3'}$ = 4.9 Hz, OH-3′); 5.48 (d, 1H, $J_{OH,2'}$ = 6.4 Hz, OH-2′); 6.21 (d, 1H, $J_{1',2'}$ = 6.4 Hz, H-1′); 6.83 (bt, 1H, $J_{5,6}$ = $J_{5,F}$ = 4.3 Hz, H-5); 7.89 (ddd; 1H, $J_{7,8}$ = 8.3 Hz, $J_{7,6}$ = 6.9 Hz, $J_{7,5}$ = 1.0 Hz, H-7-naphthyl); 7.94 (ddd, 1H, $J_{6,5}$ = 8.5 Hz, $J_{6,7}$ = 6.9 Hz, $J_{6,8}$ = 1.3 Hz, H-6-naphthyl); 8.03 (d, 1H, $J_{6,5}$ = 3.9 Hz, H-6); 8.21 (d, 1H, $J_{3,F}$ = 6.4 Hz, H-3-naphthyl); 8.26 (bd, 1H, $J_{5,6}$ = 8.4 Hz, H-5-naphthyl); 8.31 (bd, 1H, $J_{8,7}$ = 8.1 Hz, H-8-naphthyl); ^{13}C NMR (125.7 MHz, DMSO-d$_6$): 61.5 (CH$_2$-5′); 70.7 (CH-3′); 74.1 (CH-2′); 85.6 (CH-4′); 86.6 (CH-1′); 102.1 (d, $J_{C,F}$ = 9.1 Hz, CH-5); 116.9 (d, $J_{C,F}$ = 3.6 Hz, C-4-naphthyl); 117.1 (C-4a); 119.3 (d, $J_{C,F}$ = 14.0 Hz, C-2-naphthyl); 122.0 (d, $J_{C,F}$ = 6.2 Hz, CH-8-naphthyl); 124.1 (d, $J_{C,F}$ = 17.4 Hz, C-8a-naphthyl); 126.8 (CH-5-naphthyl); 128.6 (CH-7-naphthyl); 129.1 (CH-6); 129.9 (d, $J_{C,F}$ = 3.3 Hz, CH-3-naphthyl); 130.6 (CH-6-naphthyl); 132.9 (d, $J_{C,F}$ = 4.9 Hz, C-4a-naphthyl); 152.3 (C-2); 153.1 and 153.2 (C-4,7a); 155.2 (d, $J_{C,F}$ = 260.0 Hz, C-1-naphthyl); ^{19}F NMR (470.4 MHz, DMSO-d$_6$): –116.99 (bt, 1F, $J_{F,5}$ = $J_{F,3}$ = 5.5 Hz,

F-1). HR-ESI-MS: *found*: 508.0071 ([M + H]$^+$, calcd for $C_{21}H_{17}O_4N_3BrClF^+$: 508.0070); HR-ESI-MS: *found*: 529.9891 ([M + Na]$^+$, calcd for $C_{21}H_{16}O_4N_3BrClFNa^+$: 529.9889).

2-Chloro-4-(6-chloronaphthalen-2-yl)-7-(*β*-D-ribofuranosyl)-7*H*-pyrrolo[2,3-*d*]pyrimidine (6B.27)

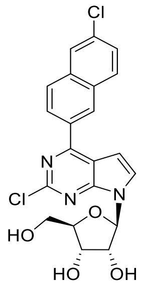

Nucleoside **5** (79.5 mg, 0.25 mmol) was reacted with 6-chloronaphthalene-2-boronic acid (56.4 mg, 0.27 mmol) for 1 h at 100 °C according to the GP A. HPFC (SiO_2, DCM/MeOH 1:0 → 9:1) gave **6B.27** (81.6 mg, 74 %) as a white powder. ^{1}H NMR (500 MHz, DMSO-d$_6$): 3.59 (ddd, 1H, J_{gem} = 11.8 Hz, $J_{5'a,OH}$ = 5.4 Hz, $J_{5'a,4'}$ = 4.0 Hz, H-5′a); 3.67 (ddd, 1H, J_{gem} = 11.8 Hz, $J_{5'b,OH}$ = 5.4 Hz, $J_{5'b,4'}$ = 4.1 Hz, H-5′b); 3.97 (q, 1H, $J_{4',5'a} = J_{4',5'b} = J_{4',3'}$ = 3.8 Hz, H-4′); 4.14 (td, 1H, $J_{3',2'} = J_{3',OH}$ = 5.0 Hz, $J_{3',4'}$ = 3.1 Hz, H-3′); 4.46 (td, 1H, $J_{2',1'} = J_{2',OH}$ = 6.3 Hz, $J_{2',3'}$ = 5.0 Hz, H-2′); 5.07 (t, 1H, $J_{OH,5'a} = J_{OH,5'b}$ = 5.4 Hz, OH-5′); 5.26 (d, 1H, $J_{OH,3'}$ = 4.9 Hz, OH-3′); 5.46 (d, 1H, $J_{OH,2'}$ = 6.3 Hz, OH-2′); 6.21 (d, 1H, $J_{1',2'}$ = 6.2 Hz, H-1′); 7.28 (d, 1H, $J_{5,6}$ = 3.9 Hz, H-5); 7.65 (dd; 1H, $J_{7,8}$ = 8.8 Hz, $J_{7,5}$ = 2.2 Hz, H-7-naphthyl); 8.07 (d, 1H, $J_{6,5}$ = 3.9 Hz, H-6); 8.13 (d, 1H, $J_{4,3}$ =8.7 Hz, H-4-naphthyl); 8.18 (d, 1H, $J_{5,7}$ = 2.2 Hz, H-5-naphthyl); 8.28 (d, 1H, $J_{8,7}$ = 8.9 Hz, H-8-naphthyl); 8.33 (dd, 1H, $J_{3,4}$ = 8.6 Hz, $J_{3,1}$ = 1.8 Hz, H-3-naphthyl); 8.81 (bd, 1H, $J_{1,3}$ = 1.8 Hz, H-1-naphthyl); ^{13}C NMR (125.7 MHz, DMSO-d$_6$): 61.5 (CH_2-5′); 70.6 (CH-3′); 74.1 (CH-2′); 85.5 (CH-4′); 86.6 (CH-1′); 101.91 (CH-5); 114.8 (C-4a); 126.4 (CH-5-naphthyl); 126.6 (CH-3-naphthyl); 127.4 (CH-7-naphthyl); 128.0 (CH-4-naphthyl); 129.1 (CH-6); 129.2 (CH-1-naphthyl); 131.2 (C-8a-naphthyl); 131.5 (CH-8-naphthyl); 132.4 (C-6-naphthyl); 134.0 (C-2-naphthyl); 134.7 (C-4a-naphthyl); 152.4 (C-2); 153.6 (C-7a); 157.6 (C-4). HR-ESI-MS: *found*: 446.0666 ([M + H]$^+$, calcd for $C_{21}H_{18}O_4N_3Cl_2^+$: 446.0669); HR-ESI-MS: *found*: 468.0488 ([M + Na]$^+$, calcd for $C_{21}H_{17}O_4N_3Cl_2Na^+$: 468.0488).

2-Chloro-4-(4-fluoronaphth-1-yl)-7-(*β*-D-ribofuranosyl)-7*H*-pyrrolo[2,3-*d*]pyrimidine (6B.28)

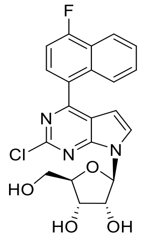

Nucleoside **5** (81.6 mg, 0.26 mmol) was reacted with 4-fluoronaphthalene-1-boronic acid (53.3 mg, 0.28 mmol) for 1 h at 100 °C according to the GP A. HPFC (SiO_2, DCM/MeOH 1:0 → 9:1) gave **6B.28** (85.1 mg, 78 %) as a white powder. ^{1}H NMR (500 MHz, DMSO-d_6): 3.57 (ddd, 1H, J_{gem} = 11.9 Hz, $J_{5'a,OH}$ = 5.3 Hz, $J_{5'a,4'}$ = 4.0 Hz, H-5′a); 3.65 (ddd, 1H, J_{gem} = 11.9 Hz, $J_{5'b,OH}$ = 5.3 Hz, $J_{5'b,4'}$ = 4.1 Hz, H-5′b); 3.97 (td, 1H, $J_{4',5'a}$ = $J_{4',5'b}$ = 4.0 Hz, $J_{4',3'}$ = 3.0 Hz, H-4′); 4.13 (td, 1H, $J_{3',2'}$ = $J_{3',OH}$ = 4.9 Hz, $J_{3',4'}$ = 3.0 Hz, H-3′); 4.46 (td, 1H, $J_{2',1'}$ = $J_{2',OH}$ = 6.3 Hz, $J_{2',3'}$ = 4.9 Hz, H-2′); 5.05 (t, 1H, $J_{OH,5'a}$ = $J_{OH,5'b}$ = 5.3 Hz, OH-5′); 5.28 (d, 1H, $J_{OH,3'}$ = 4.9 Hz, OH-3′); 5.47 (d, 1H, $J_{OH,2'}$ = 6.3 Hz, OH-2′); 6.22 (d, 1H, $J_{1',2'}$ = 6.3 Hz, H-1′); 6.52 (d, 1H, $J_{5,6}$ = 3.8 Hz, H-5); 7.54 (dd; 1H, $J_{3,F}$ = 10.4 Hz, $J_{3,2}$ = 8.0 Hz, H-3-naphthyl); 7.67 (ddd, 1H, $J_{7,8}$ = 8.6 Hz, $J_{7,6}$ = 6.8 Hz, $J_{7,5}$ = 1.4 Hz, H-7- naphthyl); 7.73 (ddd, 1H, $J_{6,5}$ = 8.4 Hz, $J_{6,7}$ = 6.8 Hz, $J_{6,8}$ = 1.2 Hz, H-6-naphthyl); 7.82 (dd, 1H, $J_{2,3}$ = 8.0 Hz, $J_{2,F}$ = 5.5 Hz, H-2-naphthyl); 7.96 (d, 1H, $J_{6,5}$ = 3.8 Hz, H-6); 8.11 (dm, 1H, $J_{8,7}$ = 8.6 Hz, H-8-naphthyl); 8.20 (bd, 1H, $J_{5,6}$ = 8.4 Hz, H-5-naphthyl); ^{13}C NMR (125.7 MHz, DMSO-d_6): 61.5 (CH_2-5′); 70.7 (CH-3′); 74.1 (CH-2′); 85.6 (CH-4′); 86.6 (CH-1′); 101.4 (CH-5); 109.5 (d, $J_{C,F}$ = 20.4 Hz, CH-3-naphthyl); 117.5 (C-4a); 120.4 (d, $J_{C,F}$ = 5.6 Hz, CH-5-naphthyl); 123.2 (d, $J_{C,F}$ = 16.3 Hz, C-4a-naphthyl); 125.6 (d, $J_{C,F}$ = 2.5 Hz, CH-8-naphthyl); 127.3 (CH-6-naphthyl); 128.2 (CH-7-naphthyl); 128.8 (CH-6); 129.0 (d, $J_{C,F}$ = 9.2 Hz, CH-2-naphthyl); 129.9 (d, $J_{C,F}$ = 4.2 Hz, C-1-naphthyl); 131.7 (d, $J_{C,F}$ = 5.0 Hz, C-8a-naphthyl); 152.2 (C-2); 153.0 (C-7a); 158.9 (d, $J_{C,F}$ = 254.0 Hz, C-4-naphthyl); 159.2 (C-4); ^{19}F NMR (470.4 MHz, DMSO-d_6): –115.7 (ddd, 1F, $J_{F,3}$ = 10.4 Hz, $J_{F,2}$ = 5.5 Hz, $J_{F,5}$ = 2.0 Hz, F-4). HR-ESI-MS: *found*: 430.0964 ($[M + H]^+$, calcd for $C_{21}H_{18}O_4N_3ClF^+$: 430.0964); HR-ESI-MS: *found*: 452.0783 ($[M + Na]^+$, calcd for $C_{21}H_{17}O_4N_3ClFNa^+$: 452.0784).

2-Chloro-4-(6-(methoxycarbonyl)naphthalen-2-yl)-7-(*β*-D-ribofuranosyl)-7*H*-pyrrolo[2,3-*d*]pyrimidine (6B.29)

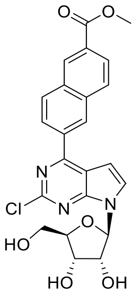

Nucleoside **5** (82.3 mg, 0.26 mmol) was reacted with methyl 6-(4,4,5,5-tetramethyl-1,3,2-dioxaborolan-2-yl)-2-naphthalenecarboxylate (88.3 mg, 0.28 mmol) for 1 h at 100 °C according to the GP A. HPFC (SiO_2, DCM/MeOH 1:0 → 9:1) gave **6B.29** (106.4 mg, 88 %) as a white powder. ^{1}H NMR (500 MHz, DMSO-d$_6$): 3.59 (ddd, 1H, J_{gem} = 11.9 Hz, $J_{5'a,OH}$ = 5.4 Hz, $J_{5'a,4'}$ = 4.0 Hz, H-5′a); 3.67 (ddd, 1H, J_{gem} = 11.9 Hz, $J_{5'b,OH}$ = 5.4 Hz, $J_{5'b,4'}$ = 4.1 Hz, H-5′b); 3.95 (s, 3H, CH_3O); 3.97 (m, 1H, H-4′); 4.14 (td, 1H, $J_{3',2'}$ = $J_{3',OH}$ = 4.9 Hz, $J_{3',4'}$ = 3.1 Hz, H-3′); 4.46 (td, 1H, $J_{2',1'}$ = $J_{2',OH}$ = 6.2 Hz, $J_{2',3'}$ = 5.0 Hz, H-2′); 5.08 (t, 1H, $J_{OH,5'a}$ = $J_{OH,5'b}$ = 5.4 Hz, OH-5′); 5.26 (d, 1H, $J_{OH,3'}$ = 4.9 Hz, OH-3′); 5.47 (d, 1H, $J_{OH,2'}$ = 6.3 Hz, OH-2′); 6.22 (d, 1H, $J_{1',2'}$ = 6.2 Hz, H-1′); 7.29 (d, 1H, $J_{5,6}$ = 3.8 Hz, H-5); 8.04–8.13 (m; 2H, H-6, H-7-naphthyl); 8.31–8.39 (m, 3H, H-3,4,8-naphthyl); 8.74 (m, 1H, H-5-naphthyl); 8.83 (m, 1H, H-1-naphthyl); ^{13}C NMR (125.7 MHz, DMSO-d$_6$): 52.4 (**C**H_3OCO); 61.5 (CH_2-5′); 70.6 (CH-3′); 74.2 (CH-2′); 85.5 (CH-4′); 86.6 (CH-1′); 101.9 (CH-5); 115.0 (C-4a); 125.3 (CH-7-naphthyl); 126.4 (CH-3-naphthyl); 128.4 (C-6-naphthyl); 128.9 (CH-1-naphthyl); 129.3 (CH-6); 129.9 (CH-8-naphthyl); 130.24 and 130.27 (CH-4,5-naphthyl); 133.1 (C-4a-naphthyl); 134.9 (C-8a-naphthyl); 135.9 (C-2-naphthyl); 152.5 (C-2); 153.6 (C-7a); 157.5 (C-4); 166.2 (CH_3O**C**O). HR-ESI-MS: *found*: 470.1111 ($[M + H]^+$, calcd for $C_{23}H_{21}O_6N_3Cl^+$: 470.1113); HR-ESI-MS: *found*: 492.0930 ($[M + Na]^+$, calcd for $C_{23}H_{20}O_6N_3ClNa^+$: 492.0933).

2-Chloro-4-(2,2-dimethyl-2,3-dihydrobenzofuran-5-yl)-7-(*β*-D-ribofuranosyl)-7*H*-pyrrolo[2,3-*d*]pyrimidine (6B.30)

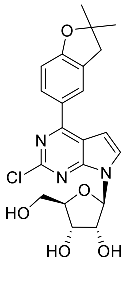

Nucleoside **5** (80.6 mg, 0.25 mmol) was reacted with 2-(2,2-dimethyl-2,3-dihydro-1-benzofuran-5-yl)-4,4,5,5-tetramethyl-1,3,2-dioxaborolane (75.9 mg, 0.28 mmol) for 1 h at 100 °C according to the GP A. HPFC (SiO_2, DCM/MeOH 1:0 → 9:1) gave **6B.30** (49.6 mg, 46 %) as a white powder. ^{1}H NMR (500 MHz, DMSO-d_6): 1.47 (s, 6H, CH_3-$C_{10}H_{11}O$); 3.14 (s, 2H, H-3-$C_{10}H_{11}O$); 3.56 (ddd, 1H, J_{gem} = 11.8 Hz, $J_{5'a,OH}$ = 5.5 Hz, $J_{5'a,4'}$ = 4.0 Hz, H-5′a); 3.64 (ddd, 1H, J_{gem} = 11.8 Hz, $J_{5'b,OH}$ = 5.4 Hz, $J_{5'b,4'}$ = 4.1 Hz, H-5′a); 3.94 (btd, 1H, $J_{4',5'a}$ = $J_{4',5'b}$ = 4.0 Hz, $J_{4',3'}$ = 3.1 Hz, H-4′); 4.11 (td, 1H, $J_{3',2'}$ = $J_{3',OH}$ = 5.0 Hz, $J_{3',4'}$ = 3.0 Hz, H-3′); 4.42 (td, 1H, $J_{2',1'}$ = $J_{2',OH}$ = 6.3 Hz, $J_{2',3'}$ = 5.0 Hz, H-2′); 5.04 (t, 1H, $J_{OH,5'a}$ = $J_{OH,5'b}$ = 5.4 Hz, OH-5′); 5.24 (d, 1H, $J_{OH,3'}$ = 4.8 Hz, OH-3′); 5.42 (d, 1H, $J_{OH,2'}$ = 6.3 Hz, OH-2′); 6.16 (d, 1H, $J_{1',2'}$ = 6.3 Hz, H-1′); 6.91 (d, 1H, $J_{7,6}$ = 8.4 Hz, H-7-$C_{10}H_{11}O$); 7.08 (d, 1H, $J_{5,6}$ = 3.8 Hz, H-5); 7.94 (d, 1H, $J_{6,5}$ = 3.9 Hz, H-6); 7.99 (dd, 1H, $J_{6,7}$ = 8.4 Hz, $J_{6,4}$ = 2.1 Hz, H-6-$C_{10}H_{11}O$); 8.04 (m, 1H, H-4-$C_{10}H_{11}O$); ^{13}C NMR (125.7 MHz, DMSO-d_6): 28.0 (CH_3-$C_{10}H_{11}O$); 41.7 (CH_2-3-$C_{10}H_{11}O$); 61.5 (CH_2-5′); 70.6 (CH-3′); 74.0 (CH-2′); 85.4 (CH-4′); 86.5 (CH-1′); 88.3 (C-2-$C_{10}H_{11}O$); 101.9 (CH-5); 109.5 (CH-7-$C_{10}H_{11}O$); 113.5 (C-4a); 126.1 (CH-4-$C_{10}H_{11}O$); 128.1 (CH-6); 128.3 (C-5-$C_{10}H_{11}O$); 128.6 (C-3a-$C_{10}H_{11}O$); 129.8 (CH-6-$C_{10}H_{11}O$); 152.4 (C-2); 153.3 (C-7a); 158.3 (C-4); 161.2 (C-7a-$C_{10}H_{11}O$). HR-ESI-MS: *found*: 432.1320 ([M + H]$^+$, calcd for $C_{21}H_{23}O_5N_3Cl^+$: 432.1321); HR-ESI-MS: *found*: 454.1139 ([M + Na]$^+$, calcd for $C_{21}H_{22}O_5N_3ClNa^+$: 454.1140).

4-(6-(Benzyloxy)naphthalen-2-yl)-2-chloro-7-(*β*-D-ribofuranosyl)-7*H*-pyrrolo[2,3-*d*]pyrimidine (6B.31)

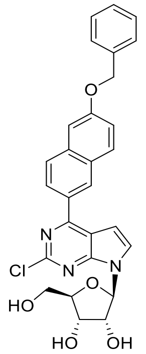

Nucleoside **5** (81.7 mg, 0.26 mmol) was reacted with 6-benzyloxy-2-naphthylboronic acid (78.4 mg, 0.28 mmol) for 20 min at 100 ° C in microwave according to the GP A. HPFC (SiO_2, DCM/MeOH 1:0 → 9:1) gave **6B.31** (91.9 mg, 70 %) as a white powder. ^{1}H NMR (500 MHz, DMSO-d_6): 3.59 (ddd, 1H, J_{gem} = 11.9 Hz, $J_{5'a,OH}$ = 5.4 Hz, $J_{5'a,4'}$ = 4.0 Hz, H-5′a); 3.67 (ddd, 1H, J_{gem} = 11.9 Hz, $J_{5'b,OH}$ = 5.4 Hz, $J_{5'b,4'}$ = 4.3 Hz, H-5′b); 3.96 (q, 1H, $J_{4',5'a}$ = $J_{4',5'b}$

= $J_{4',3'}$ = 3.8 Hz, H-4′); 4.14 (td, 1H, $J_{3',2'}$ = $J_{3',OH}$ = 5.0 Hz, $J_{3',4'}$ = 3.1 Hz, H-3′); 4.46 (td, 1H, $J_{2',1'}$ = $J_{2',OH}$ = 6.2 Hz, $J_{2',3'}$ = 5.0 Hz, H-2′); 5.07 (t, 1H, $J_{OH,5'a}$ = $J_{OH,5'b}$ = 5.4 Hz, OH-5′); 5.25 (d, 1H, $J_{OH,3'}$ = 4.9 Hz, OH-3′); 5.29 (s, 2H, CH_2-Ph); 5.46 (d, 1H, $J_{OH,2'}$ = 6.3 Hz, OH-2′); 6.21 (d, 1H, $J_{1',2'}$ = 6.2 Hz, H-1′); 7.26 (d, 1H, $J_{5,6}$ = 3.8 Hz, H-5); 7.35 (dd; 1H, $J_{7,8}$ = 9.0 Hz, $J_{7,5}$ = 2.6 Hz, H-7-naphthyl); 7.36 (m, 1H, H-*p*-Ph); 7.43 (m, 2H, H-*m*-Ph); 7.54 (m, 2H, H-o-Ph); 7.56 (d, 1H, $J_{5,7}$ = 2.6 Hz, H-5-naphthyl); 8.00 (d, 1H, $J_{4,3}$ =8.7 Hz, H-4-naphthyl); 8.04 (d, 1H, $J_{6,5}$ = 3.8 Hz, H-6); 8.15 (d, 1H, $J_{8,7}$ = 9.1 Hz, H-8-naphthyl); 8.25 (dd, 1H, $J_{3,4}$ = 8.6 Hz, $J_{3,1}$ = 1.9 Hz, H-3-naphthyl); 8.72 (d, 1H, $J_{1,3}$ = 1.9 Hz, H-1-naphthyl); ^{13}C NMR (125.7 MHz, DMSO-d_6): 61.5 (CH_2-5′); 69.5 (CH_2Ph); 70.6 (CH-3′); 74.1 (CH-2′); 85.5 (CH-4′); 86.6 (CH-1′); 102.0 (CH-5); 107.2 (CH-5-naphthyl); 114.4 (C-4a); 119.7 (CH-7-naphthyl); 125.9 (CH-3-naphthyl); 127.5 (CH-4-naphthyl); 128.0 (CH-*o*-Ph); 128.0 (CH-*p*-Ph); 128.3 (C-8a-naphthyl); 128.5 (CH-*m*-Ph); 128.7 (CH-6); 129.1 (CH-1-naphthyl); 131.0 (CH-8-naphthyl); 131.4 (C-2-naphthyl); 135.6 (C-4a-naphthyl); 136.7 (C-*i*-Ph); 152.5 (C-2); 153.5 (C-7a); 157.9 (C-6-naphthyl); 158.2 (C-4). HR-ESI-MS: *found*: 518.1473 ([M + H]$^+$, calcd for $C_{28}H_{25}O_5N_3Cl^+$: 518.1477); HR-ESI-MS: *found*: 540.1293 ([M + Na]$^+$, calcd for $C_{28}H_{24}O_5N_3ClNa^+$: 540.1297).

2-Chloro-4-(6-hydroxynaphthalen-2-yl)-7-(*β*-D-ribofuranosyl)-7*H*-pyrrolo[2,3-*d*]pyrimidine (6B.32)

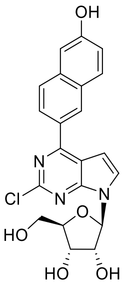

Nucleoside **5** (82.2 mg, 0.26 mmol) was reacted with 6-hydroxynaphthalene-2-boronic acid (53.1 mg, 0.28 mmol) for 1 h at 100 °C according to the GP A. HPFC (SiO_2, DCM/MeOH 1:0 → 9:1) gave **6B.32** (54.8 mg, 50 %) as a pale-yellow powder. ^{1}H NMR (500 MHz, DMSO-d_6): 3.58 (ddd, 1H, J_{gem} = 12.0 Hz, $J_{5'a,OH}$ = 5.4 Hz, $J_{5'a,4'}$ = 4.0 Hz, H-5′a); 3.66 (ddd, 1H, J_{gem} = 12.0 Hz, $J_{5'b,OH}$ = 5.4 Hz, $J_{5'b,4'}$ = 4.3 Hz, H-5′b); 3.96 (q, 1H, $J_{4',5'a}$ = $J_{4',5'b}$ = $J_{4',3'}$ = 3.8 Hz, H-4′); 4.14 (td, 1H, $J_{3',2'}$ = $J_{3',OH}$ = 5.0 Hz, $J_{3',4'}$ = 3.1 Hz, H-3′); 4.45 (td, 1H, $J_{2',1'}$ = $J_{2',OH}$ = 6.3 Hz, $J_{2',3'}$ = 5.0 Hz, H-2′); 5.07 (t, 1H, $J_{OH,5'a}$ = $J_{OH,5'b}$ = 5.4 Hz, OH-5′); 5.25 (d, 1H, $J_{OH,3'}$ = 4.9 Hz, OH-3′); 5.45 (d, 1H, $J_{OH,2'}$ = 6.4 Hz, OH-2′); 6.20 (d, 1H, $J_{1',2'}$ = 6.2 Hz, H-1′); 7.18 (dd; 1H, $J_{7,8}$ = 8.8 Hz, $J_{7,5}$ = 2.4 Hz, H-7-naphthyl); 7.22 (d, 1H, $J_{5,7}$ = 2.4 Hz, H-5-naphthyl); 7.24 (d, 1H, $J_{5,6}$ = 3.8 Hz,

H-5); 7.88 (d, 1H, $J_{4,3}$ =8.7 Hz, H-4-naphthyl); 8.02 (d, 1H, $J_{6,5}$ = 3.8 Hz, H-6); 8.09 (d, 1H, $J_{8,7}$ = 8.9 Hz, H-8-naphthyl); 8.17 (dd, 1H, $J_{3,4}$ = 8.7 Hz, $J_{3,1}$ = 1.8 Hz, H-3-naphthyl); 8.66 (d, 1H, $J_{1,3}$ = 1.8 Hz, H-1-naphthyl); 10.12 (s, 1H, OH-6-naphthyl); ^{13}C NMR (125.7 MHz, DMSO-d$_6$): 61.5 (CH$_2$-5′); 70.6 (CH-3′); 74.1 (CH-2′); 85.5 (CH-4′); 86.6 (CH-1′); 102.0 (CH-5); 108.7 (CH-5-naphthyl); 114.3 (C-4a); 119.5 (CH-7-naphthyl); 125.6 (CH-3-naphthyl); 126.8 (CH-4-naphthyl); 127.4 (C-8a-naphthyl); 128.5 (CH-6); 129.3 (CH-1-naphthyl); 130.4 (C-2-naphthyl); 131.2 (CH-8-naphthyl); 136.0 (C-4a-naphthyl); 152.5 (C-2); 153.5 (C-7a); 157.2 (C-6-naphthyl); 158.4 (C-4). HR-ESI-MS: *found*: 428.1005 ([M + H]$^+$, calcd for $C_{21}H_{19}O_5N_3Cl^+$: 428.1008); HR-ESI-MS: *found*: 450.0825 ([M + Na]$^+$, calcd for $C_{21}H_{18}O_5N_3ClNa^+$: 450.0827).

4-(4-Aminonaphth-1-yl)-2-chloro-7-(*β*-D-ribofuranosyl)-7*H*-pyrrolo[2,3-*d*]pyrimidine (6B.33)

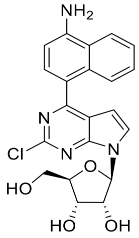

Nucleoside **5** (91.4 mg, 0.29 mmol) was reacted with 4-(4,4,5,5-tetramethyl-1,3,2-dioxaborolan-2-yl)naphthalen-1-amine (84.5 mg, 0.31 mmol) for 1 hour at 100 °C according to the GP A. HPFC purification (SiO_2, DCM/MeOH 1:0 → 9:1) gave **6B.33** (115.8 mg, 95 %) as a yellow powder. ^{1}H NMR (500 MHz, DMSO-d$_6$): 3.57 (ddd, 1H, J_{gem} = 11.8 Hz, $J_{5'a,OH}$ = 5.5 Hz, $J_{5'a,4'}$ = 4.0 Hz, H-5′a); 3.64 (ddd, 1H, J_{gem} = 11.8 Hz, $J_{5'b,OH}$ = 5.4 Hz, $J_{5'b,4'}$ = 4.2 Hz, H-5′b); 3.95 (q, 1H, $J_{4',3'}$ = $J_{4',5'a}$ = $J_{4',5'b}$ = 3.8 Hz, H-4′); 4.12 (td, 1H, $J_{3',2'}$ = $J_{3',OH}$ = 4.9 Hz, $J_{3',4'}$ = 2.9 Hz, H-3′); 4.45 (td, 1H, $J_{2',1'}$ = $J_{2',OH}$ = 6.4 Hz, $J_{2',3'}$ = 5.0 Hz, H-2′); 5.04 (t, 1H, $J_{OH,5'a}$ = $J_{OH,5'b}$ = 5.4 Hz, OH-5′); 5.24 (d, 1H, $J_{OH,3'}$ = 4.8 Hz, OH-3′); 5.44 (d, 1H, $J_{OH,2'}$ = 6.4 Hz, OH-2′); 6.18 (d, 1H, $J_{1',2'}$ = 6.4 Hz, H-1′); 6.42 (bs, 2H, NH$_2$); 6.54 (d, 1H, $J_{5,6}$ = 3.8 Hz, H-5); 6.81 (d; 1H, $J_{3,2}$ = 8.0 Hz, H-3-naphthyl); 7.42–7.50 (m, 2H, H-6,7-naphthyl); 7.63 (d, 1H, $J_{2,3}$ = 8.0 Hz, H-2-naphthyl); 7.86 (d, 1H, $J_{6,5}$ = 3.8 Hz, H-6); 8.20 (m, 1H, H-5-naphthyl); 8.25 (m, 1H, H-8-naphthyl); ^{13}C NMR (125.7 MHz, DMSO-d$_6$): 61.55 (CH$_2$-5′); 70.7 (CH-3′); 74.0 (CH-2′); 85.4 (CH-4′); 86.5 (CH-1′); 102.0 (CH-5); 106.5 (CH-3-naphthyl); 116.30 (C-4a); 120.0 (C-1-naphthyl); 122.3 (C-4a-naphthyl); 122.7 (CH-5-naphthyl); 124.1 (CH-6-naphthyl); 125.3 (CH-8-naphthyl); 126.7 (CH-7-naphthyl); 127.4 (CH-6); 131.2 (CH-2-naphthyl); 131.5 (C-8a-naphthyl); 147.7 (C-4-naphthyl); 152.1 (C-2);

152.8 (C-7a); 161.2 (C-4). HR-ESI-MS: *found*: 427.1170 ([M + H]$^+$, calcd for $C_{21}H_{20}O_4N_4Cl^+$: 427.1168); HR-ESI-MS: *found*: 449.0990 ([M + Na]$^+$, calcd for $C_{21}H_{19}O_4N_4ClNa^+$: 449.0987).

2-Chloro-4-(4-cyanonaphth-1-yl)-7-(*β*-D-ribofuranosyl)-7*H*-pyrrolo[2,3-*d*]pyrimidine (6B.34)

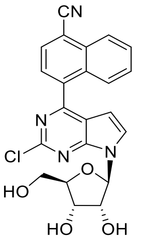

Nucleoside **5** (81.7 mg, 0.26 mmol) was reacted with 4-(tetramethyl-1,3,2-dioxaborolan-2-yl)naphthalene-1-carbonitrile (78.4 mg, 0.28 mmol) for 1 h at 100 °C according to the GP A. HPFC (SiO_2, DCM/MeOH 1:0 → 9:1) gave **6B.34** (76.9 mg, 69 %) as a white powder. ^{1}H NMR (500 MHz, DMSO-d$_6$): 3.58 (ddd, 1H, J_{gem} = 11.8 Hz, $J_{5'a,OH}$ = 5.3 Hz, $J_{5'a,4'}$ = 3.9 Hz, H-5′a); 3.65 (ddd, 1H, J_{gem} = 11.8 Hz, $J_{5'b,OH}$ = 5.4 Hz, $J_{5'b,4'}$ = 4.1 Hz, H-5′b); 3.98 (m, 1H, H-4′); 4.13 (td, 1H, $J_{3',2'}$ = $J_{3',OH}$ = 4.9 Hz, $J_{3',4'}$ = 3.0 Hz, H-3′); 4.47 (td, 1H, $J_{2',1'}$ = $J_{2',OH}$ = 6.3 Hz, $J_{2',3'}$ = 4.9 Hz, H-2′); 5.06 (t, 1H, $J_{OH,5'a}$ = $J_{OH,5'b}$ = 5.3 Hz, OH-5′); 5.29 (d, 1H, $J_{OH,3'}$ = 4.8 Hz, OH-3′); 5.48 (d, 1H, $J_{OH,2'}$ = 6.3 Hz, OH-2′); 6.23 (d, 1H, $J_{1',2'}$ = 6.3 Hz, H-1′); 6.50 (d, 1H, $J_{5,6}$ = 3.8 Hz, H-5); 7.74 (ddd, 1H, $J_{7,8}$ = 8.6 Hz, $J_{7,6}$ = 6.8 Hz, $J_{7,5}$ = 1.3 Hz, H-7- naphthyl); 7.90 (ddd, 1H, $J_{6,5}$ = 8.4 Hz, $J_{6,7}$ = 6.9 Hz, $J_{6,8}$ = 1.2 Hz, H-6-naphthyl); 7.94 (d, 1H, $J_{2,3}$ = 7.4 Hz, H-2-naphthyl); 8.00 (d, 1H, $J_{6,5}$ = 3.8 Hz, H-6); 8.10 (dm, 1H, $J_{8,7}$ = 8.6 Hz, H-8-naphthyl); 8.28 (dm, 1H, $J_{5,6}$ = 8.4 Hz, H-5-naphthyl); 8.36 (d; 1H, $J_{3,2}$ = 7.4 Hz, H-3-naphthyl); ^{13}C NMR (125.7 MHz, DMSO-d$_6$): 61.48 (CH_2-5′); 70.7 (CH-3′); 74.2 (CH-2′); 85.6 (CH-4′); 86.7 (CH-1′); 101.1 (CH-5); 110.7 (C-4-naphthyl); 117.4 (CN); 117.7 (C-4a); 124.9 (CH-5-naphthyl); 126.6 (CH-8-naphthyl); 127.6 (CH-2-naphthyl); 128.6 (CH-7-naphthyl); 129.4 (CH-6); 129.5 (CH-6-naphthyl); 129.8 (C-8a-naphthyl); 132.0 (C-4a-naphthyl); 132.8 (CH-3-naphthyl); 138.6 (C-1-naphthyl); 152.2 (C-2); 153.1 (C-7a); 158.1 (C-4). HR-ESI-MS: *found*: 437.1013 ([M + H]$^+$, calcd for $C_{22}H_{18}O_4N_4Cl^+$: 437.1011); HR-ESI-MS: *found*: 459.0834 ([M + Na]$^+$, calcd for $C_{22}H_{17}O_4N_4ClNa^+$: 459.0831).

2-(2-Chloro-4-(6-methoxynaphthalen-2-yl)-7-(*β*-D-ribofuranosyl)-7*H*-pyrrolo[2,3-*d*]pyrimidine (6B.35)

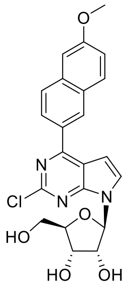

Nucleoside **5** (109.7 mg, 0.34 mmol) was reacted with 6-methoxy-2-naphthylboronic acid (76.1 mg, 0.38 mmol) for 10 min at 100 °C in microwave according to the GP A. HPFC (SiO_2, DCM/MeOH 1:0 → 9:1) gave **6B.35** (125.2 mg, 83 %) as a white powder. ^{1}H NMR (500 MHz, DMSO-d_6): 3.59 (ddd, 1H, J_{gem} = 11.8 Hz, $J_{5'a,OH}$ = 5.4 Hz, $J_{5'a,4'}$ = 4.0 Hz, H-5′a); 3.67 (ddd, 1H, J_{gem} = 11.8 Hz, $J_{5'b,OH}$ = 5.4 Hz, $J_{5'b,4'}$ = 4.1 Hz, H-5′b); 3.93 (s, 3H, CH_3O); 3.93 (q, 1H, $J_{4',5'a}$ = $J_{4',5'b}$ = $J_{4',3'}$ = 3.8 Hz, H-4′); 4.14 (td, 1H, $J_{3',2'}$ = $J_{3',OH}$ = 5.0 Hz, $J_{3',4'}$ = 3.1 Hz, H-3′); 4.46 (td, 1H, $J_{2',1'}$ = $J_{2',OH}$ = 6.3 Hz, $J_{2',3'}$ = 4.9 Hz, H-2′); 5.06 (t, 1H, $J_{OH,5'a}$ = $J_{OH,5'b}$ = 5.4 Hz, OH-5′); 5.24 (d, 1H, $J_{OH,3'}$ = 5.0 Hz, OH-3′); 5.45 (d, 1H, $J_{OH,2'}$ = 6.4 Hz, OH-2′); 6.21 (d, 1H, $J_{1',2'}$ = 6.2 Hz, H-1′); 7.26 (d, 1H, $J_{5,6}$ = 3.9 Hz, H-5); 7.27 (dd; 1H, $J_{7,8}$ = 9.1 Hz, $J_{7,5}$ = 2.5 Hz, H-7-naphthyl); 7.44 (d, 1H, $J_{5,7}$ = 2.5 Hz, H-5-naphthyl); 8.02 (d, 1H, $J_{4,3}$ =8.6 Hz, H-4-naphthyl); 8.03 (d, 1H, $J_{6,5}$ = 3.9 Hz, H-6); 8.13 (d, 1H, $J_{8,7}$ = 9.1 Hz, H-8-naphthyl); 8.25 (dd, 1H, $J_{3,4}$ = 8.6 Hz, $J_{3,1}$ = 1.9 Hz, H-3-naphthyl); 8.71 (d, 1H, $J_{1,3}$ = 1.9 Hz, H-1-naphthyl); ^{13}C NMR (125.7 MHz, DMSO-d_6): 55.4 (CH_3O); 61.5 (CH_2-5′); 70.6 (CH-3′); 74.1 (CH-2′); 85.5 (CH-4′); 86.6 (CH-1′); 102.0 (CH-5); 105.9 (CH-5-naphthyl); 114.4 (C-4a); 119.4 (CH-7-naphthyl); 125.9 (CH-3-naphthyl); 127.5 (CH-4-naphthyl); 128.2 (C-8a-naphthyl); 128.6 (CH-6); 129.1 (CH-1-naphthyl); 130.9 (CH-8-naphthyl); 131.3 (C-2-naphthyl); 135.7 (C-4a-naphthyl); 152.5 (C-2); 153.5 (C-7a); 158.2 (C-4); 158.8 (C-6-naphthyl). HR-ESI-MS: *found*: 442.1165 ([M + H]$^+$, calcd for $C_{22}H_{21}O_5N_3Cl^+$: 442.1164); HR-ESI-MS: *found*: 464.0986 ([M + Na]$^+$, calcd for $C_{22}H_{20}O_5N_3ClNa^+$: 464.0984).

[(5-{[2-Chloro-4-(naphth-1-yl)-7*H*-pyrrolo[2,3-*d*]pyrimidin-7-yl]-*β*-D-ribofuranosyl}oxy)phosphonomethyl]phosphonic acid (7B.6)

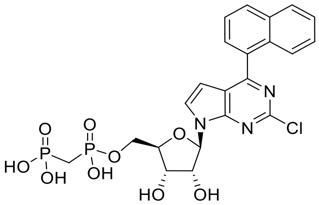

GP B using compound **6B.6** (83.3 mg, 0.20 mmol). HPLC (C-18, H_2O + 0.05 % TFA/MeCN 0 → 80 %) gave product **7B.6** (34 mg, 30 %) as a pale-yellow powder. ^{1}H NMR (500 MHz, DMSO-

d_6): 2.26 (t, 2H, d, $J_{CH2,P}$ = 20.5 Hz, PCH_2P); 4.09–4.17 (m, 3H, H-4′,5′); 4.21 (dd, 1H, $J_{3′,2′}$ = 5.1 Hz, $J_{3′,4′}$ = 2.5 Hz, H-3′); 4.49 (dd, 1H, $J_{2′,1′}$ = 6.5 Hz, $J_{2′,3′}$ = 5.1 Hz, H-2′); 6.26 (d, 1H, $J_{1′,2′}$ = 6.5 Hz, H-1′); 6.47 (d, 1H, $J_{5,6}$ = 3.8 Hz, H-5); 7.55 (ddd, 1H, $J_{7,8}$ = 8.4 Hz, $J_{7,6}$ = 6.8 Hz, $J_{7,5}$ = 1.5 Hz, H-7-naphthyl); 7.61 (ddd, 1H, $J_{6,5}$ = 8.1 Hz, $J_{6,7}$ = 6.8 Hz, $J_{6,8}$ = 1.3 Hz, H-6-naphthyl); 7.70 (dd, 1H, $J_{3,4}$ = 8.3 Hz, $J_{3,2}$ = 7.1 Hz, H-3-naphthyl); 7.80 (dd, 1H, $J_{2,3}$ = 7.1 Hz, $J_{2,4}$ = 1.3 Hz, H-2-naphthyl); 7.95 (d, 1H, $J_{6,5}$ = 3.8 Hz, H-6); 8.03 (dm, 1H, $J_{8,7}$ = 8.4 Hz, H-8-naphthyl); 8.08 (dm, 1H, $J_{5,6}$ = 8.2 Hz, H-5-naphthyl); 8.16 (bd, 1H, $J_{4,3}$ = 8.3 Hz, H-4-naphthyl); ^{13}C NMR (125.7 MHz, DMSO-d_6): 27.6 (t, $J_{C,P}$ = 129.0 Hz, PCH_2P); 64.7 (d, $J_{C,P}$ = 5.4 Hz, CH_2-5′); 70.4 (CH-3′); 73.7 (CH-2′); 83.3 (d, $J_{C,P}$ = 7.4 Hz, CH-4′); 86.3 (CH-1′); 101.7 (CH-5); 117.4 (C-4a); 125.2 (CH-8-naphthyl); 125.4 (CH-3-naphthyl); 126.5 (CH-6-naphthyl); 127.0 (CH-7-naphthyl); 128.47 and 128.49 (CH-2,5-naphthyl); 128.6 (CH-6); 130.1 (C-8a-naphthyl); 130.4 (CH-4-naphthyl); 133.3 and 133.5 (C-1,4a-naphthyl); 152.3 (C-2); 153.1 (C-7a); 160.1 (C-4); ^{31}P NMR (202.4 MHz, DMSO-d_6): 18.88 and 14.67 (2×d, 2×1P, $J_{P,P}$ = 8.2 Hz, PCH_2P). HR-ESI-MS: *found*: 568.0443 ($[M–H]^-$, calcd for $C_{22}H_{21}O_9N_3ClP_2^-$: 568.0447).

[(5-{[2-Chloro-4-(naphthalen-2-yl)-7*H*-pyrrolo[2,3-*d*]pyrimidin-7-yl]-*β*-D-ribofuranosyl}oxy)phosphonomethyl]phosphonic acid (7B.7)

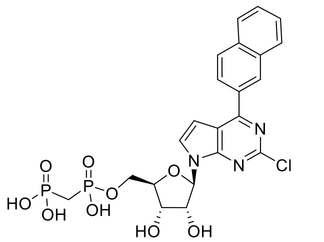

GP B using compound **6B.7** (81.9 mg, 0.2 mmol). HPLC (C-18, H_2O + 0.05 % TFA/MeCN 0 → 80 %) gave product **7B.7** (39.2 mg, 35 %) as a yellow powder. ^{1}H NMR (500 MHz, DMSO-d_6): 2.28 (t, 2H, d, $J_{CH2,P}$ = 20.5 Hz, PCH_2P); 4.11–4.17 (m, 3H, H-4′,5′); 4.22 (dd, 1H, $J_{3′,2′}$ = 5.1 Hz, $J_{3′,4′}$ = 2.6 Hz, H-3′); 4.49 (dd, 1H, $J_{2′,1′}$ = 6.4 Hz, $J_{2′,3′}$ = 5.1 Hz, H-2′); 6.25 (d, 1H, $J_{1′,2′}$ = 6.4 Hz, H-1′); 7.26 (d, 1H, $J_{5,6}$ = 3.9 Hz, H-5); 7.60–7.68 (m, 2H, H-6,7-naphthyl); 8.04 (m, 1H, H-5-naphthyl); 8.06 (d, 1H, $J_{6,5}$ = 3.9 Hz, H-6); 8.14 (d, 1H, $J_{4,3}$ = 8.3 Hz, H-4-naphthyl); 8.23 (m, 1H, H-8-naphthyl); 8.27 (dd, 1H, $J_{3,4}$ = 8.6 Hz, $J_{3,1}$ = 1.8 Hz, H-3-naphthyl); 8.77 (d, 1H, $J_{1,3}$ = 1.8 Hz, H-1-naphthyl); ^{13}C NMR (125.7 MHz, DMSO-d_6): 27.6 (d, $J_{C,P}$ = 128.7 Hz, PCH_2P); 64.7 (d, $J_{C,P}$ = 5.5 Hz, CH_2-5′); 70.4 (CH-3′); 73.8 (CH-2′); 83.2 (d, $J_{C,P}$ = 7.5 Hz, CH-4′); 86.3 (CH-1′); 102.2 (CH-5); 114.7 (C-4a); 125.4 (CH-3-naphthyl); 126.8 (CH-7-naphthyl); 127.7 (CH-5-naphthyl);

127.9 (CH-6-naphthyl); 128.7 (CH-4-naphthyl); 128.8 (CH-6); 129.3 (CH-1,8-naphthyl); 132.8 (C-8a-naphthyl); 133.6 (C-2-naphthyl); 134.0 (C-4a-naphthyl); 152.5 (C-2); 153.7 (C-7a); 158.1 (C-4); ^{31}P NMR (202.4 MHz, DMSO-d$_6$): 19.83 and 15.71 (2×d, 2×1P, $J_{P,P}$ = 8.2 Hz, PCH$_2$P). HR-ESI-MS: *found*: 568.0443 ([M–H]$^-$, calcd for $C_{22}H_{21}O_9N_3ClP_2^-$: 568.0447).

[(5-{[2-Chloro-4-(benzofuran-2-yl)-7*H*-pyrrolo[2,3-*d*]pyrimidin-7-yl]-*β*-D-ribofuranosyl}oxy)phosphonomethyl]phosphonic acid (7B.8)

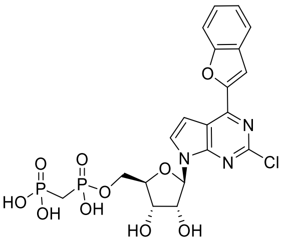

GP B using compound **6B.8** (65.8 mg, 0.16 mmol). HPLC (C-18, H_2O + 0.05 % TFA/MeCN 0 → 80 %) gave product **7B.8** (36.8 mg, 40 %) as a yellow powder. ^{1}H NMR (500 MHz, DMSO-d$_6$): 2.28 (t, 2H, $J_{CH2,P}$ = 20.5 Hz, PCH$_2$P); 4.10–4.17 (m, 3H, H-4′,5′); 4.21 (dd, 1H, $J_{3',2'}$ = 5.1 Hz, $J_{3',4'}$ = 2.7 Hz, H-3′); 4.47 (dd, 1H, $J_{2',1'}$ = 6.4 Hz, $J_{2',3'}$ = 5.1 Hz, H-2′); 6.21 (d, 1H, $J_{1',2'}$ = 6.4 Hz, H-1′); 7.32 (d, 1H, $J_{5,6}$ = 3.8 Hz, H-5); 7.38 (ddd, 1H, $J_{5,4}$ = 8.0 Hz, $J_{5,6}$ =7.2 Hz, $J_{5,7}$ = 0.9 Hz, H-5-benzofuryl); 7.51 (ddd, 1H, $J_{6,7}$ = 8.4 Hz, $J_{6,5}$ =7.2 Hz, $J_{6,4}$ = 1.3 Hz, H-6-benzofuryl); 7.83 (dm, 1H, $J_{4.5}$ = 8.0 Hz, H-4-benzofuryl); 7.84 (bdq, 1H, $J_{7,6}$ = 8.4 Hz, $J_{7,5}$ = $J_{7,4}$ = $J_{7,3}$ = 0.8 Hz, H-7-benzofuryl); 8.01 (d, 1H, $J_{3.7}$ = 1.0 Hz, H-3-benzofuryl); 8.07 (d, 1H, $J_{6,5}$ = 3.8 Hz, H-6); ^{13}C NMR (125.7 MHz, DMSO-d$_6$): 27.6 (t, $J_{C,P}$ = 128.6 Hz, PCH$_2$P); 64.7 (d, $J_{C,P}$ = 5.3 Hz, CH$_2$-5′); 70.4 (CH-3′); 73.8 (CH-2′); 83.2 (d, $J_{C,P}$ = 7.4 Hz, CH-4′); 86.3 (CH-1′); 102.52 (CH-5); 110.7 (CH-3-benzofuryl); 112.1 (CH-7-benzofuryl); 113.0 (C-4a); 122.7 (CH-4-benzofuryl); 124.0 (CH-5-benzofuryl); 127.1 (CH-6-benzofuryl); 127.6 (C-3a-benzofuryl); 129.3 (CH-6); 148.0 (C-4); 152.39 and 152.5 (C-2, C-2-benzofuryl); 154.0 (C-7a); 155.5 (C-7a-benzofuryl); ^{31}P NMR (202.4 MHz, DMSO-d$_6$): 14.56 and 18.74 (2×bd, 2×1P, $J_{P,P}$ = 7.4 Hz, PCH$_2$P). HR-ESI-MS: *found*: 558.0236 ([M–H]$^-$, calcd for $C_{20}H_{19}O_{10}N_3ClP_2^-$: 558.0240).

[(5-{[2-Chloro-4-(5,6,7,8-tetrahydronaphth-1-yl)-7*H*-pyrrolo[2,3-*d*]pyrimidin-7-yl]-*β*-D-ribofuranosyl}oxy)phosphonomethyl]phosphonic acid (7B.12)

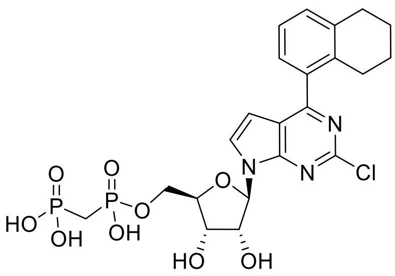

GP B using compound **6B.12** (59.3 mg, 0.14 mmol). HPLC (C-18, H_2O + 0.05 % TFA/MeCN 0 → 80 %) gave **7B.12** (32.5 mg, 40 %) as a white powder. ^{1}H NMR (500 MHz, DMSO-d$_6$): 1.66 (m, 2H, H-3-$C_{10}H_{11}$); 1.75 (m, 2H, H-2-$C_{10}H_{11}$); 2.25 (t, 2H, $J_{CH2,P}$ = 20.4 Hz, PCH_2P); 2.64 (t, 2H, $J_{4,3}$ = 6.4 Hz, H-4-$C_{10}H_{11}$); 2.84 (t, 2H, $J_{1,2}$ = 6.4 Hz, H-1-$C_{10}H_{11}$); 4.07–4.11 (m, 3H, H-4′,5′); 4.19 (bd, 1H, $J_{3',2'}$ = 5.1 Hz, H-3′); 4.47 (dd, 1H, $J_{2',1'}$ = 6.5 Hz, $J_{2',3'}$ = 5.1 Hz, H-2′); 6.21 (d, 1H, $J_{1',2'}$ = 6.5 Hz, H-1′); 6.49 (d, 1H, $J_{5,6}$ = 3.8 Hz, H-5); 7.21–7.29 (m, 3H, H-6,7,8-$C_{10}H_{11}$); 7.91 (d, 1H, $J_{6,5}$ = 3.8 Hz, H-6); ^{13}C NMR (125.7 MHz, DMSO-d$_6$): 22.2 (CH_2-2-$C_{10}H_{11}$); 22.5 (CH_2-3-$C_{10}H_{11}$); 26.8 (CH_2-4-$C_{10}H_{11}$); 27.5 (t, $J_{C,P}$ = 129.3 Hz, PCH_2P); 29.3 (CH_2-1-$C_{10}H_{11}$); 64.7 (d, $J_{C,P}$ = 5.7 Hz, CH_2-5′); 70.4 (CH-3′); 73.7 (CH-2′); 83.3 (d, $J_{C,P}$ = 7.4 Hz, CH-4′); 86.2 (CH-1′); 101.6 (CH-5); 116.8 (C-4a); 125.4 (CH-7-$C_{10}H_{11}$); 127.0 (CH-6-$C_{10}H_{11}$); 128.4 (CH-6); 130.6 (CH-8-$C_{10}H_{11}$); 135.0 (C-5-$C_{10}H_{11}$); 135.6 (C-4a-$C_{10}H_{11}$); 137.9 (C-8a-$C_{10}H_{11}$); 152.0 (C-2); 152.9 (C-7a); 161.5 (C-4); ^{31}P NMR (202.4 MHz, DMSO-d$_6$): 15.77 and 19.72 (2×bd, 2×1P, $J_{P,P}$ = 6.1 Hz, PCH_2P). HR-ESI-MS: *found*: 572.0758 ($[M{-}H]^-$, calcd for $C_{22}H_{25}O_9N_3ClP_2^-$: 572.0760).

[(5-{[2-Chloro-4-(5,6,7,8-tetrahydronaphthalen-2-yl)-7*H*-pyrrolo[2,3-*d*]pyrimidin-7-yl]-*β*-D-ribofuranosyl}oxy)phosphonomethyl]phosphonic acid (7B.23)

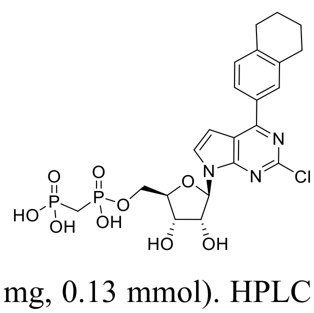

GP B using compound **6B.23** (53.9 mg, 0.13 mmol). HPLC (C-18, H_2O + 0.05 % TFA/MeCN 0 → 80 %) gave **7B.23** (28.2 mg, 38 %) as a white powder. ^{1}H NMR (500 MHz, DMSO-d$_6$): 1.75–1.82 (m, 4H, H-2,3-$C_{10}H_{11}$); 2.25 (t, 2H, $J_{CH2,P}$ = 19.7 Hz, PCH_2P); 2.81 (m, 2H, H-4-$C_{10}H_{11}$); 2.87 (m, 2H, H-1-$C_{10}H_{11}$); 4.07–4.15 (m, 3H, H-4′,5′); 4.20 (m, 1H, H-3′); 4.46 (t, 1H, $J_{2',1'}$ = $J_{2',3'}$ = 5.7 Hz, H-2′); 6.21 (d, 1H, $J_{1',2'}$ = 6.4 Hz, H-1′); 7.05 (d, 1H, $J_{5,6}$ = 3.8 Hz, H-5); 7.28 (d, 1H, $J_{8,7}$ = 8.0 Hz, H-8-$C_{10}H_{11}$); 7.83 (bd, 1H, $J_{5,7}$ = 1.9 Hz, H-5-$C_{10}H_{11}$); 7.86 (dd, 1H, $J_{7,8}$ = 7.9 Hz, $J_{7,5}$ =

2.0 Hz, H-7-$C_{10}H_{11}$); 7.97 (d, 1H, $J_{6,5}$ = 3.8 Hz, H-6); ^{13}C NMR (125.7 MHz, DMSO-d_6): 22.5 and 22.6 (CH_2-2,3-$C_{10}H_{11}$); 27.5 (t, $J_{C,P}$ = 129.0 Hz, PCH_2P); 28.8 and 28.9 (CH_2-1,4-$C_{10}H_{11}$); 64.7 (bd, $J_{C,P}$ = 3.9 Hz, CH_2-5′); 70.4 (CH-3′); 73.8 (CH-2′); 83.2 (d, $J_{C,P}$ = 6.8 Hz, CH-4′); 86.2 (CH-1′); 102.1 (CH-5); 114.2 (C-4a); 125.9 (CH-7-$C_{10}H_{11}$); 128.4 (CH-6); 129.2 (CH-5-$C_{10}H_{11}$); 129.7 (CH-8-$C_{10}H_{11}$); 133.4 (C-6-$C_{10}H_{11}$); 137.5 (C-4a-$C_{10}H_{11}$); 140.4 (C-8a-$C_{10}H_{11}$); 152.5 (C-2); 153.6 (C-7a); 158.5 (C-4); ^{31}P NMR (202.4 MHz, DMSO-d_6): 15.85 and 19.60 (2×s, 2×1P, PCH_2P). HR-ESI-MS: *found*: 572.0756 ([M–H]$^-$, calcd for $C_{22}H_{25}O_9N_3ClP_2^-$: 572.0760).

[(5-{[2-Chloro-4-(1*H*-indol-2-yl)-7*H*-pyrrolo[2,3-*d*]pyrimidin-7-yl]-*β*-D-ribofuranosyl}oxy)phosphonomethyl]phosphonic acid (7B.24)

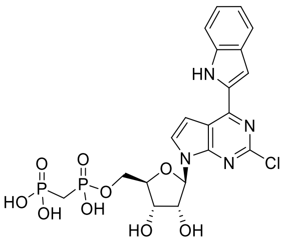

GP B using compound **6B.24** (38.2 mg, 0.095 mmol). HPLC (C-18, H_2O + 0.05 % TFA/MeCN 0 → 80 %) gave **7B.24** (27.1 mg, 51 %) as a yellow powder. ^{1}H NMR (500 MHz, DMSO-d_6): 2.28 (t, 2H, $J_{CH2,P}$ = 20.4 Hz, PCH_2P); 4.09–4.17 (m, 3H, H-4′,5′); 4.21 (dd, 1H, $J_{3',2'}$ = 5.1 Hz, $J_{3',4'}$ = 2.6 Hz, H-3′); 4.47 (dd, 1H, $J_{2',1'}$ = 6.4 Hz, $J_{2',3'}$ = 5.1 Hz, H-2′); 6.20 (d, 1H, $J_{1',2'}$ = 6.4 Hz, H-1′); 7.08 (ddd, 1H, $J_{5,4}$ = 8.0 Hz, $J_{5,6}$ = 6.9 Hz, $J_{5,7}$ = 1.0 Hz, H-5-indolyl); 7.24 (ddd, 1H, $J_{6,7}$ = 8.3 Hz, $J_{6,5}$ = 6.9 Hz, $J_{6,4}$ = 1.2 Hz, H-6-indolyl); 7.30 (d, 1H, $J_{5,6}$ = 3.8 Hz, H-5); 7.58 (bdq, 1H, $J_{7,6}$ = 8.3 Hz, $J_{7,5}$ = $J_{7,4}$ = $J_{7,3}$ = 1.0 Hz, H-7-indolyl); 7.65 (bd, 1H, $J_{3,NH}$ = 2.3 Hz, H-3-indolyl); 7.68 (dq, 1H, $J_{4,5}$ = 8.0 Hz, $J_{4,6}$ = $J_{4,7}$ = $J_{4,3}$ = 1.0 Hz, H-4-indolyl); 7.99 (d, 1H, $J_{6,5}$ = 3.8 Hz, H-6); 11.86 (d, 1H, $J_{NH,3}$ = 1.9 Hz, NH); ^{13}C NMR (125.7 MHz, DMSO-d_6): 27.6 (t, $J_{C,P}$ = 128.7 Hz, PCH_2P); 64.8 (d, $J_{C,P}$ = 5.4 Hz, CH_2-5′); 70.5 (CH-3′); 73.7 (CH-2′); 83.2 (d, $J_{C,P}$ = 7.4 Hz, CH-4′); 86.2 (CH-1′); 101.9 (CH-5); 107.1 (CH-3-indolyl); 112.7 (C-4a); 112.7 (CH-7-indolyl); 120.1 (CH-5-indolyl); 121.5 (CH-4-indolyl); 124.3 (CH-6-indolyl); 128.3 (C-3a-indolyl); 128.5 (CH-6); 133.2 (C-2-indolyl); 137.6 (C-7a-indolyl); 150.7 (C-4); 152.4 (C-2); 153.6 (C-7a); ^{31}P NMR (202.4 MHz, DMSO-d_6): 15.80 and 19.74 (2×bd, 2×1P, $J_{P,P}$ = 6.5 Hz, PCH_2P). HR-ESI-MS: *found*: 557.0399 ([M–H]$^-$, calcd for $C_{20}H_{20}O_9N_4ClP_2^-$: 557.0400).

[(5-{[2-Chloro-4-(6-fluoronaphthalen-2-yl)-7*H*-pyrrolo[2,3-*d*]pyrimidin-7-yl]-*β*-D-ribofuranosyl}oxy)phosphonomethyl]phosphonic acid (7B.25)

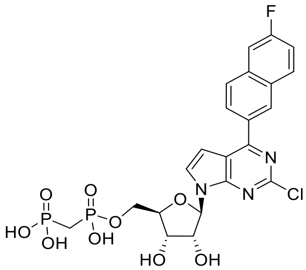

GP B using compound **6B.25** (51.3 mg, 0.12 mmol). HPLC (C-18, H_2O + 0.05 % TFA/MeCN 0 → 80 %) gave **7B.25** (23.5 mg, 33 %) as a pale-yellow powder. ^{1}H NMR (500 MHz, DMSO-d$_6$): 2.25 (bt, 2H, $J_{CH2,P}$ = 19.9 Hz, PCH$_2$P); 4.07–4.17 (m, 3H, H-4′,5′); 4.22 (m, 1H, H-3′); 4.49 (bt, 1H, $J_{2',1'}$ = $J_{2',3'}$ = 5.7 Hz, H-2′); 6.25 (d, 1H, $J_{1',2'}$ = 6.4 Hz, H-1′); 7.27 (d, 1H, $J_{5,6}$ = 3.8 Hz, H-5); 7.55 (td; 1H, $J_{7,8}$ = $J_{7,F}$ = 8.9 Hz, $J_{7,5}$ = 2.6 Hz, H-7-naphthyl); 7.84 (dd, 1H, $J_{5,F}$ = 10.2 Hz, $J_{5,7}$ = 2.6 Hz, H-5-naphthyl); 8.07 (d, 1H, $J_{6,5}$ = 3.8 Hz, H-6); 8.12 (d, 1H, $J_{4,3}$ =8.7 Hz, H-4-naphthyl); 8.32 (bdd, 1H, $J_{3,4}$ = 8.6 Hz, $J_{3,1}$ = 1.9 Hz, H-3-naphthyl); 8.34 (dd, 1H, $J_{8,7}$ = 9.1 Hz, $J_{8,F}$ = 5.8 Hz, H-8-naphthyl); 8.82 (bd, 1H, $J_{1,3}$ = 1.8 Hz, H-1-naphthyl); ^{13}C NMR (125.7 MHz, DMSO-d$_6$): 27.5 (t, $J_{C,P}$ = 129.0 Hz, PCH$_2$P); 64.7 (bs, CH$_2$-5′); 70.5 (CH-3′); 73.8 (CH-2′); 83.3 (d, $J_{C,P}$ = 6.5 Hz, CH-4′); 86.3 (CH-1′); 102.2 (CH-5); 110.9 (d, $J_{C,F}$ = 20.8 Hz, CH-5-naphthyl); 114.6 (C-4a); 117.1 (d, $J_{C,F}$ = 25.5 Hz, CH-7-naphthyl); 126.5 (CH-3-naphthyl); 128.2 (d, $J_{C,F}$ = 5.3 Hz, CH-4-naphthyl); 128.9 (CH-6); 129.3 (CH-1-naphthyl); 130.0 (C-8a-naphthyl); 132.4 (d, $J_{C,F}$ = 9.4 Hz, CH-8-naphthyl); 133.1 (d, $J_{C,F}$ = 2.6 Hz, C-2-naphthyl); 135.0 (d, $J_{C,F}$ = 9.9 Hz, C-4a-naphthyl); 152.5 (C-2); 153.7 (C-7a); 157.8 (C-4); 161.1 (d, $J_{C,F}$ = 246.3 Hz, C-6-naphthyl); ^{31}P NMR (202.4 MHz, DMSO-d$_6$): 16.06 and 19.25 (2×bs, 2×1P, PCH$_2$P); ^{19}F NMR (470.4 MHz, DMSO-d$_6$): –107.52 (td, 1F, $J_{F,5}$ = $J_{F,7}$ = 9.5 Hz, $J_{F,8}$ = 5.9 Hz, F-6). HR-ESI-MS: *found*: 586.0358 ([M–H]$^-$, calcd for $C_{22}H_{20}O_9N_3ClFP_2^-$: 586.0353).

[(5-{[2-Chloro-4-(4-bromo-1-fluoronaphthalen-2-yl)-7*H*-pyrrolo[2,3-*d*]pyrimidin-7-yl]-*β*-D-ribofuranosyl}oxy)phosphonomethyl]phosphonic acid (7B.26)

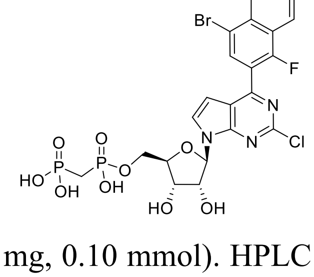

GP B using compound **6B.26** (50.0 mg, 0.10 mmol). HPLC (C-18, H_2O + 0.05 % TFA/MeCN 0 → 80 %) gave **7B.26** (23.9 mg, 36 %) as a white powder. ^{1}H NMR (500 MHz, DMSO-d$_6$): 2.15

(t, 2H, $J_{CH2,P}$ = 19.9 Hz, PCH_2P); 4.03–4.08 (m, 2H, H-5′); 4.10 (m, 1H, H-4′); 4.21 (bdd, 1H, $J_{3',2'}$ = 5.0 Hz, $J_{3',4'}$ = 2.7 Hz, H-3′); 4.49 (bdd, 1H, $J_{2',1'}$ = 6.5 Hz, $J_{2',3'}$ = 5.0 Hz, H-2′); 6.24 (d, 1H, $J_{1',2'}$ = 6.5 Hz, H-1′); 6.80 (bt, 1H, $J_{5,6}$ = $J_{5,F}$ = 4.4 Hz, H-5); 7.86 (bddd; 1H, $J_{7,8}$ = 8.3 Hz, $J_{7,6}$ = 7.0 Hz, $J_{7,5}$ = 1.1 Hz, H-7-naphthyl); 7.94 (ddd, 1H, $J_{6,5}$ = 8.5 Hz, $J_{6,7}$ = 7.0 Hz, $J_{6,8}$ = 1.2 Hz, H-6-naphthyl); 8.08 (d, 1H, $J_{6,5}$ = 3.8 Hz, H-6); 8.21 (d, 1H, $J_{3,F}$ = 6.3 Hz, H-3-naphthyl); 8.25 (bd, 1H, $J_{5,6}$ = 8.5 Hz, H-5-naphthyl); 8.31 (bd, 1H, $J_{8,7}$ = 8.3 Hz, H-8-naphthyl); ^{13}C NMR (125.7 MHz, DMSO-d_6): 27.4 (t, $J_{C,P}$ = 121.8 Hz, PCH_2P); 64.4 (CH_2-5′); 70.6 (CH-3′); 73.9 (CH-2′); 83.6 (d, $J_{C,P}$ = 7.3 Hz, CH-4′); 86.3 (CH-1′); 102.3 (d, $J_{C,F}$ = 9.0 Hz, CH-5); 116.8 (d, $J_{C,F}$ = 3.6 Hz, C-4-naphthyl); 117.0 (C-4a); 119.3 (d, $J_{C,F}$ = 13.9 Hz, C-2-naphthyl); 122.0 (d, $J_{C,F}$ = 6.3 Hz, CH-8-naphthyl); 124.1 (d, $J_{C,F}$ = 17.3 Hz, C-8a-naphthyl); 126.8 (CH-5-naphthyl); 128.6 (CH-7-naphthyl); 129.1 (CH-6); 129.9 (d, $J_{C,F}$ = 3.2 Hz, CH-3-naphthyl); 130.6 (CH-6-naphthyl); 132.9 (d, $J_{C,F}$ = 5.1 Hz, C-4a-naphthyl); 152.3 (C-2); 153.1 and 153.3 (C-4,7a); 155.2 (d, $J_{C,F}$ = 259.7 Hz, C-1-naphthyl); ^{31}P NMR (202.4 MHz, DMSO-d_6): 16.71 and 18.08 (2×bs, 2×1P, PCH_2P); ^{19}F NMR (470.4 MHz, DMSO-d_6): –116.80 (bm, 1F, F-1). HR-ESI-MS: *found*: 663.9467 ($[M–H]^-$, calcd for $C_{22}H_{19}O_9N_3BrClFP_2^-$: 663.9458).

[(5-{[2-Chloro-4-(6-chloronaphthalen-2-yl)-7*H*-pyrrolo[2,3-*d*]pyrimidin-7-yl]-*β*-D-ribofuranosyl}oxy)phosphonomethyl]phosphonic acid (7B.27)

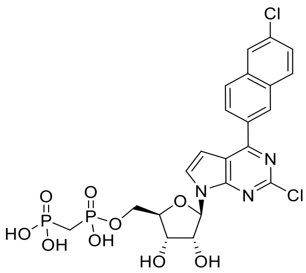

GP B using compound **6B.27** (50.7 mg, 0.11 mmol). HPLC (C-18, H_2O + 0.05 % TFA/MeCN 0 → 80 %) gave **7B.27** (25.6 mg, 37 %) as a white powder. ^1H NMR (500 MHz, DMSO-d_6): 2.24 (t, 2H, $J_{CH2,P}$ = 20.2 Hz, PCH_2P); 4.07–4.16 (m, 3H, H-4′,5′); 4.22 (dd, 1H, $J_{3',2'}$ = 5.1 Hz, $J_{3',4'}$ = 2.2 Hz, H-3′); 4.49 (dd, 1H, $J_{2',1'}$ = 6.4 Hz, $J_{2',3'}$ = 5.1 Hz, H-2′); 6.25 (d, 1H, $J_{1',2'}$ = 6.4 Hz, H-1′); 7.27 (d, 1H, $J_{5,6}$ = 3.9 Hz, H-5); 7.64 (dd; 1H, $J_{7,8}$ = 8.8 Hz, $J_{7,5}$ = 2.2 Hz, H-7-naphthyl); 8.08 (d, 1H, $J_{6,5}$ = 3.9 Hz, H-6); 8.12 (d, 1H, $J_{4,3}$ =8.8 Hz, H-4-naphthyl); 8.17 (d, 1H, $J_{5,7}$ = 2.2 Hz, H-5-naphthyl); 8.28 (d, 1H, $J_{8,7}$ = 8.9 Hz, H-8-naphthyl); 8.32 (dd, 1H, $J_{3,4}$ = 8.7 Hz, $J_{3,1}$ = 1.8 Hz, H-3-naphthyl); 8.80 (d, 1H, $J_{1,3}$ = 1.8 Hz, H-1-naphthyl); ^{13}C NMR (125.7 MHz, DMSO-d_6): 27.5 (t, $J_{C,P}$ = 127.2 Hz, PCH_2P); 64.7 (d, $J_{C,P}$ = 4.3 Hz, CH_2-5′); 70.5 (CH-3′); 73.9 (CH-2′); 83.4 (d,

$J_{C,P}$ = 7.3 Hz, CH-4′); 86.3 (CH-1′); 102.2 (CH-5); 114.7 (C-4a); 126.4 (CH-5-naphthyl); 126.6 (CH-3-naphthyl); 127.4 (CH-7-naphthyl); 128.0 (CH-4-naphthyl); 129.0 (CH-6); 129.2 (CH-1-naphthyl); 131.2 (C-8a-naphthyl); 131.5 (CH-8-naphthyl); 132.4 (C-6-naphthyl); 134.0 (C-2-naphthyl); 134.7 (C-4a-naphthyl); 152.5 (C-2); 153.8 (C-7a); 157.6 (C-4); ^{31}P NMR (202.4 MHz, DMSO-d$_6$): 16.10 and 19.18 (2×bs, 2×1P, PCH$_2$P). HR-ESI-MS: *found*: 602.0059 ([M–H]$^-$, calcd for $C_{22}H_{20}O_9N_3Cl_2P_2^-$: 602.0057).

[(5-{[2-Chloro-4-(4-fluoronaphth-1-yl)-7*H*-pyrrolo[2,3-*d*]pyrimidin-7-yl]-*β*-D-ribofuranosyl}oxy)phosphonomethyl]phosphonic acid (7B.28)

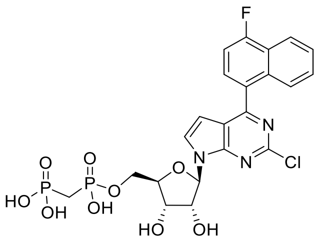

GP B using compound **6B.28** (50.6 mg, 0.12 mmol). HPLC (C-18, H_2O + 0.05 % TFA/MeCN 0 → 80 %) gave **7B.28** (25.6 mg, 37 %) as a white powder. ^{1}H NMR (500 MHz, DMSO-d$_6$): 2.24 (t, 2H, $J_{CH2,P}$ = 20.4 Hz, PCH$_2$P); 4.09–4.25 (m, 3H, H-4′,5′); 4.21 (dd, 1H, $J_{3',2'}$ = 5.1 Hz, $J_{3',4'}$ = 2.4 Hz, H-3′); 4.49 (dd, 1H, $J_{2',1'}$ = 6.4 Hz, $J_{2',3'}$ = 5.1 Hz, H-2′); 6.26 (d, 1H, $J_{1',2'}$ = 6.4 Hz, H-1′); 6.50 (d, 1H, $J_{5,6}$ = 3.8 Hz, H-5); 7.54 (dd; 1H, $J_{3,F}$ = 10.4 Hz, $J_{3,2}$ = 8.0 Hz, H-3-naphthyl); 7.67 (ddd, 1H, $J_{7,8}$ = 8.6 Hz, $J_{7,6}$ = 6.8 Hz, $J_{7,5}$ = 1.2 Hz, H-7-naphthyl); 7.73 (ddd, 1H, $J_{6,5}$ = 8.3 Hz, $J_{6,7}$ = 6.8 Hz, $J_{6,8}$ = 1.2 Hz, H-6-naphthyl); 7.83 (dd, 1H, $J_{2,3}$ = 8.0 Hz, $J_{2,F}$ = 5.5 Hz, H-2-naphthyl); 7.96 (d, 1H, $J_{6,5}$ = 3.8 Hz, H-6); 8.12 (dm, 1H, $J_{8,7}$ = 8.6 Hz, H-8-naphthyl); 8.20 (bd, 1H, $J_{5,6}$ = 8.3 Hz, H-5-naphthyl); ^{13}C NMR (125.7 MHz, DMSO-d$_6$): 27.5 (t, $J_{C,P}$ = 127.8 Hz, PCH$_2$P); 64.7 (d, $J_{C,P}$ = 5.6 Hz, CH$_2$-5′); 70.5 (CH-3′); 73.8 (CH-2′); 83.3 (d, $J_{C,P}$ = 7.4 Hz, CH-4′); 86.3 (CH-1′); 101.6 (CH-5); 109.5 (d, $J_{C,F}$ = 20.4 Hz, CH-3-naphthyl); 117.5 (C-4a); 120.4 (d, $J_{C,F}$ = 5.5 Hz, CH-5-naphthyl); 123.2 (d, $J_{C,F}$ = 16.3 Hz, C-4a-naphthyl); 125.6 (d, $J_{C,F}$ = 2.2 Hz, CH-8-naphthyl); 127.3 (CH-6-naphthyl); 128.2 (CH-7-naphthyl); 128.7 (CH-6); 129.1 (d, $J_{C,F}$ = 9.2 Hz, CH-2-naphthyl); 129.9 (d, $J_{C,F}$ = 4.2 Hz, C-1-naphthyl); 131.7 (d, $J_{C,F}$ = 5.1 Hz, C-8a-naphthyl); 152.2 (C-2); 153.2 (C-7a); 158.9 (d, $J_{C,F}$ = 254.0 Hz, C-4-naphthyl); 159.2 (C-4); ^{31}P NMR (202.4 MHz, DMSO-d$_6$): 15.84 and 19.59 (2×bs, 2×1P, PCH$_2$P); ^{19}F NMR (470.4 MHz, DMSO-d$_6$): –115.63 (bdd, 1F, $J_{F,3}$ = 10.4 Hz, $J_{F,2}$ = 5.5 Hz, F-4). HR-ESI-MS: *found*: 586.0353 ([M–H]$^-$, calcd for $C_{22}H_{19}O_9N_3BrClFP_2^-$: 586.0353).

[(5-{[2-Chloro-4-(6-(methoxycarbonyl)naphthalen-2-yl)-7*H*-pyrrolo[2,3-*d*]pyrimidin-7-yl]-*β*-D-ribofuranosyl}oxy)phosphonomethyl]phosphonic acid (7B.29)

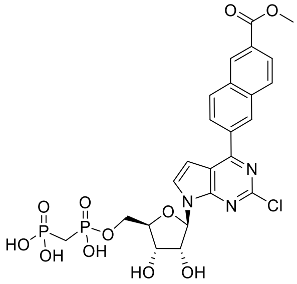

GP B using compound **6B.29** (50.2 mg, 0.11 mmol). HPLC (C-18, H_2O + 0.05 % TFA/MeCN 0 → 80 %) gave **7B.29** (23.2 mg, 35 %) as a pale-yellow powder. ^{1}H NMR (500 MHz, DMSO-d$_6$): 2.27 (t, 2H, $J_{CH2,P}$ = 19.9 Hz, PCH$_2$P); 3.95 (s, 3H, CH$_3$O); 4.09–4.19 (m, 3H, H-4′,5′); 4.22 (m, 1H, H-3′); 4.50 (bt, 1H, $J_{2',1'}$ = $J_{2',3'}$ = 5.7 Hz, H-2′); 6.26 (d, 1H, $J_{1',2'}$ = 6.4 Hz, H-1′); 7.28 (d, 1H, $J_{5,6}$ = 3.8 Hz, H-5); 8.06–8.11 (m; 2H, H-7-naphthyl, H-6); 8.32–8.38 (m, 3H, H-3,4,8-naphthyl); 8.74 (m, 1H, H-5-naphthyl); 8.84 (bs, 1H, H-1-naphthyl); ^{13}C NMR (125.7 MHz, DMSO-d$_6$): 27.5 (bm, PCH$_2$P); 52.4 (**C**H$_3$OCO); 64.7 (bs, CH$_2$-5′); 70.5 (CH-3′); 73.8 (CH-2′); 83.3 (bs, CH-4′); 86.3 (CH-1′); 102.2 (CH-5); 114.9 (C-4a); 125.5 (CH-7-naphthyl); 126.4 (CH-3-naphthyl); 128.4 (C-6-naphthyl); 128.9 (CH-1-naphthyl); 129.1 (CH-6); 129.9 (CH-8-naphthyl); 130.2 and 130.3 (CH-4,5-naphthyl); 133.1 (C-4a-naphthyl); 134.9 (C-8a-naphthyl); 135.9 (C-2-naphthyl); 152.5 (C-2); 153.8 (C-7a); 157.5 (C-4); 166.2 (CH$_3$O**C**O); ^{31}P NMR (202.4 MHz, DMSO-d$_6$): 15.83 and 19.69 (2×bs, 2×1P, PCH$_2$P). HR-ESI-MS: *found*: 626.0499 ([M–H]$^-$, calcd for $C_{24}H_{23}O_{11}N_3ClP_2^-$: 626.0502).

[(5-{[2-Chloro-4-(2,2-dimethyl-2,3-dihydrobenzofuran-5-yl)-7*H*-pyrrolo[2,3-*d*]pyrimidin-7-yl]-*β*-D-ribofuranosyl}oxy)phosphonomethyl]phosphonic acid (7B.30)

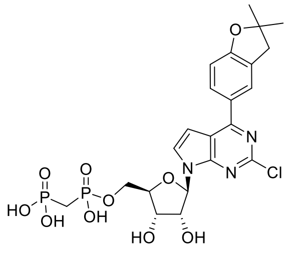

GP B using compound **6B.30** (23.6 mg, 0.055 mmol). HPLC (C-18, H_2O + 0.05 % TFA/MeCN 0 → 80 %) gave **7B.30** (13.5 mg, 19 %) as a pale-yellow powder. ^{1}H NMR (500 MHz, DMSO-d$_6$): 1.47 (s, 6H, CH$_3$-C$_{10}$H$_{11}$O); 2.21 (t, 2H, $J_{CH2,P}$ = 20.0 Hz, PCH$_2$P); 3.14 (s, 2H, H-3-C$_{10}$H$_{11}$O); 4.04–4.13 (m, 3H, H-4′,5′); 4.19 (bd, 1H, $J_{3',2'}$ = 5.0 Hz, H-3′); 4.46 (bt, 1H, $J_{2',1'}$ = $J_{2',3'}$ = 5.8 Hz,

H-2′); 6.20 (d, 1H, $J_{1',2'}$ = 6.5 Hz, H-1′); 6.90 (d, 1H, $J_{7,6}$ = 8.4 Hz, H-7-$C_{10}H_{11}O$); 7.06 (d, 1H, $J_{5,6}$ = 3.9 Hz, H-5); 7.96 (d, 1H, $J_{6,5}$ = 3.9 Hz, H-6); 7.98 (dd, 1H, $J_{6,7}$ = 8.4 Hz, $J_{6,4}$ = 2.0 Hz, H-6-$C_{10}H_{11}O$); 8.03 (m, 1H, H-4-$C_{10}H_{11}O$); ^{13}C NMR (125.7 MHz, DMSO-d$_6$): 27.5 (t, $J_{C,P}$ = 128.5 Hz, PCH_2P); 28.0 (CH_3-$C_{10}H_{11}O$); 41.7 (CH_2-3-$C_{10}H_{11}O$); 64.6 (m, CH_2-5′); 70.5 (CH-3′); 73.8 (CH-2′); 83.3 (d, $J_{C,P}$ = 7.5 Hz, CH-4′); 86.2 (CH-1′); 88.3 (C-2-$C_{10}H_{11}O$); 102.2 (CH-5); 109.5 (CH-7-$C_{10}H_{11}O$); 113.5 (C-4a); 126.1 (CH-4-$C_{10}H_{11}O$); 128.0 (CH-6); 128.2 (C-5-$C_{10}H_{11}O$); 128.6 (C-3a-$C_{10}H_{11}O$); 129.8 (CH-6-$C_{10}H_{11}O$); 152.4 (C-2); 153.5 (C-7a); 158.3 (C-4); 161.2 (C-7a-$C_{10}H_{11}O$); ^{31}P NMR (202.4 MHz, DMSO-d$_6$): 16.32 and 18.86 (2×s, 2×1P, PCH_2P). HR-ESI-MS: *found*: 588.0711 ([M–H]$^-$, calcd for $C_{22}H_{25}O_{10}N_3ClP_2^-$: 588.0709).

[(5-{[4-(6-(Benzyloxy)naphthalen-2-yl)-2-chloro-7*H*-pyrrolo[2,3-*d*]pyrimidin-7-yl]-*β*-D-ribofuranosyl}oxy)phosphonomethyl]phosphonic acid (7B.31)

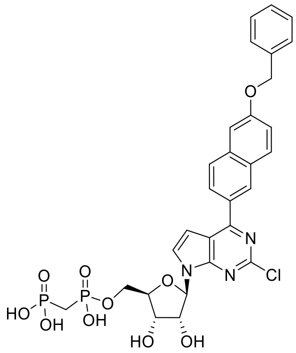

GP B using compound **6B.31** (48.2 mg, 0.093 mmol). HPLC (C-18, H_2O + 0.05 % TFA/MeCN 0 → 80 %) gave **7B.31** (23.4 mg, 37 %) as a yellow powder. ^{1}H NMR (500 MHz, DMSO-d$_6$): 2.12 (t, 2H, $J_{CH2,P}$ = 19.7 Hz, PCH_2P); 3.99–4.07 (m, 2H, H-5′); 4.10 (m, 1H, H-4′); 4.22 (m, 1H, H-3′); 4.50 (bt, 1H, $J_{2',1'} = J_{2',3'}$ = 5.8 Hz, H-2′); 5.28 (s, 2H, CH_2-Ph); 6.24 (d, 1H, $J_{1',2'}$ = 6.4 Hz, H-1′); 7.24 (d, 1H, $J_{5,6}$ = 3.9 Hz, H-5); 7.33 (dd; 1H, $J_{7,8}$ = 9.0 Hz, $J_{7,5}$ = 2.6 Hz, H-7-naphthyl); 7.36 (m, 1H, H-*p*-Ph); 7.43 (m, 2H, H-*m*-Ph); 7.52–7.56 (m, 3H, H-*o*-Ph, H-5-naphthyl); 7.99 (d, 1H, $J_{4,3}$ =8.7 Hz, H-4-naphthyl); 8.09 (d, 1H, $J_{6,5}$ = 3.9 Hz, H-6); 8.15 (d, 1H, $J_{8,7}$ = 9.1 Hz, H-8-naphthyl); 8.24 (dd, 1H, $J_{3,4}$ = 8.6 Hz, $J_{3,1}$ = 1.8 Hz, H-3-naphthyl); 8.71 (d, 1H, $J_{1,3}$ = 1.8 Hz, H-1-naphthyl); ^{13}C NMR (125.7 MHz, DMSO-d$_6$): 27.3 (t, $J_{C,P}$ = 120.0 Hz, PCH_2P); 64.3 (bs, CH_2-5′); 69.5 (CH_2-Ph); 70.7 (CH-3′); 74.0 (CH-2′); 83.6 (d, $J_{C,P}$ = 7.1 Hz, CH-4′); 86.2 (CH-1′); 102.2 (CH-5); 107.2 (CH-5-naphthyl); 114.3 (C-4a); 119.6 (CH-7-naphthyl); 125.9 (CH-3-naphthyl); 127.5 (CH-4-naphthyl); 127.96 (CH-*o*-Ph); 128.00 (CH-*p*-Ph); 128.3 (C-8a-naphthyl); 128.5 (CH-*m*-Ph); 128.7 (CH-6); 129.1 (CH-1-naphthyl); 131.0 (CH-8-naphthyl); 131.4 (C-2-

naphthyl); 135.6 (C-4a-naphthyl); 136.8 (C-*i*-Ph); 152.5 (C-2); 153.7 (C-7a); 157.8 (C-6-naphthyl); 158.1 (C-4); ^{31}P NMR (202.4 MHz, DMSO-d$_6$): 17.29 and 17.43 (2×bs, 2×1P, PCH_2P). HR-ESI-MS: *found*: 674.0871 ([M–H]$^-$, calcd for $C_{29}H_{27}O_{10}N_3ClP_2^-$: 674.0866).

[(5-{[2-Chloro-4-(6-hydroxynaphthalen-2-yl)-7*H*-pyrrolo[2,3-*d*]pyrimidin-7-yl]-*β*-D-ribofuranosyl}oxy)phosphonomethyl]phosphonic acid (7B.32)

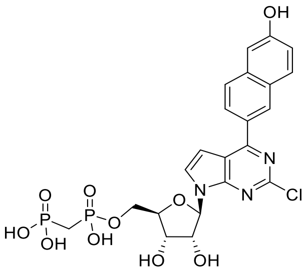

GP B using compound **6B.32** (51.4 mg, 0.12 mmol). HPLC (C-18, H_2O + 0.05 % TFA/MeCN 0 → 80 %) gave **7B.32** (21.7 mg, 31 %) as a yellow powder. ^{1}H NMR (500 MHz, DMSO-d$_6$): 2.27 (t, 2H, $J_{CH2,P}$ = 20.4 Hz, PCH_2P); 4.09–4.16 (m, 3H, H-4′,5′); 4.21 (dd, 1H, $J_{3',2'}$ = 5.1 Hz, $J_{3',4'}$ = 2.4 Hz, H-3′); 4.48 (dd, 1H, $J_{2',1'}$ = 6.4 Hz, $J_{2',3'}$ = 5.1 Hz, H-2′); 6.24 (d, 1H, $J_{1',2'}$ = 6.4 Hz, H-1′); 7.18 (dd; 1H, $J_{7,8}$ = 8.8 Hz, $J_{7,5}$ = 2.4 Hz, H-7-naphthyl); 7.21 (d, 1H, $J_{5,7}$ = 2.4 Hz, H-5-naphthyl); 7.23 (d, 1H, $J_{5,6}$ = 3.9 Hz, H-5); 7.88 (d, 1H, $J_{4,3}$ =8.7 Hz, H-4-naphthyl); 8.01 (d, 1H, $J_{6,5}$ = 3.8 Hz, H-6); 8.07 (d, 1H, $J_{8,7}$ = 8.9 Hz, H-8-naphthyl); 8.17 (dd, 1H, $J_{3,4}$ = 8.7 Hz, $J_{3,1}$ = 1.8 Hz, H-3-naphthyl); 8.66 (d, 1H, $J_{1,3}$ = 1.8 Hz, H-1-naphthyl); 10.11 (vbs, 1H, OH-6-naphthyl); ^{13}C NMR (125.7 MHz, DMSO-d$_6$): 27.6 (t, $J_{C,P}$ = 128.3 Hz, PCH_2P); 64.7 (d, $J_{C,P}$ = 5.4 Hz, CH_2-5′); 70.4 (CH-3′); 73.8 (CH-2′); 83.2 (d, $J_{C,P}$ = 7.5 Hz, CH-4′); 86.3 (CH-1′); 102.3 (CH-5); 108.7 (CH-5-naphthyl); 114.2 (C-4a); 119.5 (CH-7-naphthyl); 125.6 (CH-3-naphthyl); 126.8 (CH-4-naphthyl); 127.4 (C-8a-naphthyl); 128.4 (CH-6); 129.3 (CH-1-naphthyl); 130.4 (C-2-naphthyl); 131.2 (CH-8-naphthyl); 136.0 (C-4a-naphthyl); 152.5 (C-2); 153.6 (C-7a); 157.2 (C-6-naphthyl); 158.4 (C-4); ^{31}P NMR (202.4 MHz, DMSO-d$_6$): 15.80 and 19.71 (2×bd, 2×1P, $J_{P,P}$ = 7.6 Hz, PCH_2P). HR-ESI-MS: *found*: 584.0398 ([M–H]$^-$, calcd for $C_{22}H_{21}O_{10}N_3ClP_2^-$: 584.0396).

[(5-{[4-(4-Aminonaphth-1-yl)-2-chloro-7*H*-pyrrolo[2,3-*d*]pyrimidin-7-yl]-*β*-D-ribofuranosyl}oxy)phosphonomethyl]phosphonic acid (7B.33)

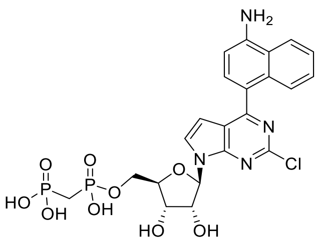

GP B using compound **6B.33** (48.5 mg, 0.11 mmol). HPLC (C-18, H_2O + 0.05 % TFA/MeCN 0 → 80 %) gave **7B.33** (27.5 mg, 41 %) as a yellow powder. ^{1}H NMR (500 MHz, DMSO-d_6): 2.25 (t, 2H, $J_{CH2,P}$ = 20.4 Hz, PCH_2P); 4.09–4.15 (m, 3H, H-4′,5′); 4.20 (dm, 1H, $J_{3',2'}$ = 5.1 Hz, H-3′); 4.46 (dd, 1H, $J_{2',1'}$ = 6.5 Hz, $J_{2',3'}$ = 5.1 Hz, H-2′); 6.22 (d, 1H, $J_{1',2'}$ = 6.5 Hz, H-1′); 6.53 (d, 1H, $J_{5,6}$ = 3.8 Hz, H-5); 6.82 (d, 1H, $J_{3,2}$ = 8.0 Hz, H-3-naphthyl); 7.45 (ddd; 1H, $J_{6,5}$ = 8.3 Hz, $J_{6,7}$ = 6.7 Hz, $J_{6,8}$ = 1.6 Hz, H-6-naphthyl); 7.48 45 (ddd; 1H, $J_{7,8}$ = 8.4 Hz, $J_{7,6}$ = 6.7 Hz, $J_{7,5}$ = 1.5 Hz, H-7-naphthyl); 7.64 (d, 1H, $J_{2,3}$ =8.0 Hz, H-2-naphthyl); 7.86 (d, 1H, $J_{6,5}$ = 3.8 Hz, H-6); 8.20 (m, 1H, H-5-naphthyl); 8.26 (m, 1H, H-8-naphthyl); ^{13}C NMR (125.7 MHz, DMSO-d_6): 27.5 (t, $J_{C,P}$ = 129.0 Hz, PCH_2P); 64.8 (d, $J_{C,P}$ = 5.5 Hz, CH_2-5′); 70.5 (CH-3′); 73.7 (CH-2′); 83.1 (d, $J_{C,P}$ = 7.6 Hz, CH-4′); 86.2 (CH-1′); 102.3 (CH-5); 106.7 (CH-3-naphthyl); 116.2 (C-4a); 120.1 (C-1-naphthyl); 122.3 (C-4a-naphthyl); 122.7 (CH-5-naphthyl); 124.2 (CH-6-naphthyl); 125.3 (CH-8-naphthyl); 126.7 (CH-7-naphthyl); 127.3 (CH-6); 131.2 (CH-2-naphthyl); 131.5 (C-8a-naphthyl); 147.6 (C-4-naphthyl); 152.5 (C-2); 153.0 (C-7a); 161.2 (C-4); ^{31}P NMR (202.4 MHz, DMSO-d_6): 15.71 and 19.80 (2×d, 2×1P, $J_{P,P}$ = 7.9 Hz, PCH_2P). HR-ESI-MS: *found*: 583.0551 ([M–H]$^-$, calcd for $C_{22}H_{22}O_9N_4ClP_2^-$: 583.0556).

[(5-{[2-Chloro-4-(4-cyanonaphth-1-yl)-7*H*-pyrrolo[2,3-*d*]pyrimidin-7-yl]-*β*-D-ribofuranosyl}oxy)phosphonomethyl]phosphonic acid (7B.34)

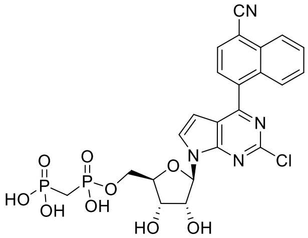

GP B using compound **6B.34** (48.6 mg, 0.11 mmol). HPLC (C-18, H_2O + 0.05 % TFA/MeCN 0 → 80 %) gave **7B.34** (36.0 mg, 54 %) as a white powder. ^{1}H NMR (500 MHz, DMSO-d_6): 2.25 (t, 2H, $J_{CH2,P}$ = 20.4 Hz, PCH_2P); 4.10–4.16 (m, 3H, H-4′,5′); 4.22 (dd, 1H, $J_{3',2'}$ = 5.1 Hz, $J_{3',4'}$ = 2.5 Hz, H-3′); 4.50 (dd, 1H, $J_{2',1'}$ = 6.4 Hz, $J_{2',3'}$ = 5.1 Hz, H-2′); 6.27 (d, 1H, $J_{1',2'}$ = 6.4 Hz, H-1′); 6.49 (d, 1H, $J_{5,6}$ = 3.8 Hz, H-5); 7.75 (ddd; 1H, $J_{7,8}$ = 8.6 Hz, $J_{7,6}$ = 6.9 Hz, $J_{7,5}$ = 1.3 Hz, H-7-

naphthyl); 7.90 (ddd; 1H, $J_{6,5}$ = 8.4 Hz, $J_{6,7}$ = 6.9 Hz, $J_{6,8}$ = 1.2 Hz, H-6-naphthyl); 7.93 (d, 1H, $J_{2,3}$ = 7.4 Hz, H-2-naphthyl); 8.00 (d, 1H, $J_{6,5}$ = 3.8 Hz, H-6); 8.12 (bdt, 1H, $J_{8,7}$ = 8.6 Hz, $J_{8,6}$ = $J_{8,5}$ = 1.0 Hz, H-8-naphthyl); 8.28 (bdt, 1H, $J_{5,6}$ = 8.4 Hz, $J_{5,7}$ = $J_{5,8}$ = 1.0 Hz, H-5-naphthyl); 8.36 (d, 1H, $J_{3,2}$ = 7.4 Hz, H-3-naphthyl); ^{13}C NMR (125.7 MHz, DMSO-d$_6$): 27.5 (t, $J_{C,P}$ = 128.6 Hz, PCH$_2$P); 64.7 (d, $J_{C,P}$ = 5.4 Hz, CH$_2$-5′); 70.4 (CH-3′); 73.8 (CH-2′); 83.3 (d, $J_{C,P}$ = 7.5 Hz, CH-4′); 86.4 (CH-1′); 101.4 (CH-5); 110.7 (C-4-naphthyl); 117.4 (CN); 117.6 (C-4a); 124.8 (CH-5-naphthyl); 126.6 (CH-8-naphthyl); 127.6 (CH-2-naphthyl); 128.6 (CH-7-naphthyl); 129.3 (CH-6); 129.5 (CH-6-naphthyl); 129.8 (C-8a-naphthyl); 132.0 (C-4a-naphthyl); 132.8 (CH-3-naphthyl); 138.6 (C-1-naphthyl); 152.2 (C-2); 153.2 (C-7a); 158.2 (C-4); ^{31}P NMR (202.4 MHz, DMSO-d$_6$): 15.78 and 19.69 (2×bs, 2×1P, PCH$_2$P). HR-ESI-MS: *found*: 593.0396 ([M–H]$^-$, calcd for $C_{23}H_{20}O_9N_4ClP_2^-$: 593.0400).

[(5-{[2-Chloro-4-(6-methoxynaphthalen-2-yl)-7*H*-pyrrolo[2,3-*d*]pyrimidin-7-yl]-*β*-D-ribofuranosyl}oxy)phosphonomethyl]phosphonic acid (7B.35)

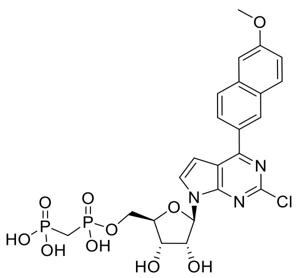

GP B using compound **6B.35** (52.7 mg, 0.12 mmol). HPLC (C-18, H$_2$O + 0.05 % TFA/MeCN 0 → 80 %) gave **7B.35** (29.4 mg, 41 %) as a yellow powder. ^{1}H NMR (500 MHz, DMSO-d$_6$): 2.27 (t, 2H, $J_{CH2,P}$ = 20.3 Hz, PCH$_2$P); 3.93 (s, 3H, CH$_3$O); 4.10–4.16 (m, 3H, H-4′,5′); 4.22 (dd, 1H, $J_{3',2'}$ = 5.4 Hz, $J_{3',4'}$ = 2.4 Hz, H-3′); 4.49 (bdd, 1H, $J_{2',1'}$ = 6.4 Hz, $J_{2',3'}$ = 5.3 Hz, H-2′); 6.24 (d, 1H, $J_{1',2'}$ = 6.4 Hz, H-1′); 7.26 (d, 1H, $J_{5,6}$ = 3.8 Hz, H-5); 7.27 (dd; 1H, $J_{7,8}$ = 9.0 Hz, $J_{7,5}$ = 2.5 Hz, H-7-naphthyl); 7.44 (d, 1H, $J_{5,7}$ = 2.5 Hz, H-5-naphthyl); 8.02 (d, 1H, $J_{4,3}$ =8.6 Hz, H-4-naphthyl); 8.03 (d, 1H, $J_{6,5}$ = 3.7 Hz, H-6); 8.14 (d, 1H, $J_{8,7}$ = 9.0 Hz, H-8-naphthyl); 8.25 (dd, 1H, $J_{3,4}$ = 8.6 Hz, $J_{3,1}$ = 1.9 Hz, H-3-naphthyl); 8.71 (d, 1H, $J_{1,3}$ = 1.9 Hz, H-1-naphthyl); ^{13}C NMR (125.7 MHz, DMSO-d$_6$): 27.6 (t, $J_{C,P}$ = 128.5 Hz, PCH$_2$P); 55.4 (CH$_3$O); 64.7 (d, $J_{C,P}$ = 4.6 Hz, CH$_2$-5′); 70.4 (CH-3′); 73.8 (CH-2′); 83.2 (d, $J_{C,P}$ = 7.5 Hz, CH-4′); 86.3 (CH-1′); 102.3 (CH-5); 105.9 (CH-5-naphthyl); 114.3 (C-4a); 119.4 (CH-7-naphthyl); 125.9 (CH-3-naphthyl); 127.5 (CH-4-naphthyl); 128.2 (C-8a-naphthyl); 128.5 (CH-6); 129.1 (CH-1-naphthyl); 130.9 (CH-8-naphthyl); 131.2 (C-2-naphthyl); 135.7 (C-4a-naphthyl); 152.5 (C-2); 153.7 (C-7a); 158.2 (C-6-

naphthyl); 158.8 (C-4); ^{31}P NMR (202.4 MHz, DMSO-d$_6$): 15.79 and 19.73 (2×bs, 2×1P, PCH$_2$P). HR-ESI-MS: *found*: 598.0546 ([M–H]$^-$, calcd for $C_{23}H_{23}O_{10}N_3ClP_2^-$: 598.0553).

2-Amino-4-(naphthalen-2-yl)-7-(*β*-D-ribofuranosyl)-7*H*-pyrrolo[2,3-*d*]pyrimidine (10C.7)

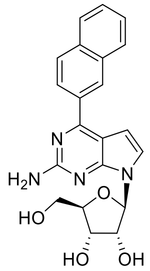

Nucleoside **8** (82.2 mg, 0.27 mmol) was reacted with naphthalene-2-boronic acid (70.5 mg, 0.41 mmol) for 1 h at 100 °C according to the GP A. HPFC (SiO_2, DCM/MeOH 1:0 → 9:1) gave **10C.7** (102.7 mg, 96 %) as a pale-yellow powder. ^{1}H NMR (500 MHz, DMSO-d$_6$): 3.54 (ddd, 1H, J_{gem} = 11.8 Hz, $J_{5'a,OH}$ = 5.6 Hz, $J_{5'a,4'}$ = 4.1 Hz, H-5′a); 3.62 (ddd, 1H, J_{gem} = 11.8 Hz, $J_{5'b,OH}$ = 5.5 Hz, $J_{5'b,4'}$ = 4.2 Hz, H-5′b); 3.87 (q, 1H, $J_{4',5'a} = J_{4',5'b} = J_{4',3'}$ = 4.0 Hz, H-4′); 4.10 (td, 1H, $J_{3',2'} = J_{3',OH}$ = 4.9 Hz, $J_{3',4'}$ = 3.2 Hz, H-3′); 4.38 (td, 1H, $J_{2',1'} = J_{2',OH}$ = 6.3 Hz, $J_{2',3'}$ = 5.1 Hz, H-2′); 5.02 (t, 1H, $J_{OH,5'a} = J_{OH,5'b}$ = 5.5 Hz, OH-5′); 5.11 (d, 1H, $J_{OH,3'}$ = 4.6 Hz, OH-3′); 5.30 (d, 1H, $J_{OH,2'}$ = 6.3 Hz, OH-2′); 6.13 (d, 1H, $J_{1',2'}$ = 6.4 Hz, H-1′); 6.39 (bs, 2H, NH$_2$); 6.82 (d, 1H, $J_{5,6}$ = 3.9 Hz, H-5); 7.45 (d, 1H, $J_{6,5}$ = 3.9 Hz, H-6); 7.57–7.63 (m; 2H, H-6,7-naphthyl); 8.00 (m, 1H, H-5-naphthyl); 8.07 (d, 1H, $J_{4,3}$ =8.6 Hz, H-4-naphthyl); 8.12 (m, 1H, H-8-naphthyl); 8.20 (dd, 1H, $J_{3,4}$ = 8.6 Hz, $J_{3,1}$ = 1.7 Hz, H-3-naphthyl); 8.61 (d, 1H, $J_{1,3}$ = 1.7 Hz, H-1-naphthyl); ^{13}C NMR (125.7 MHz, DMSO-d$_6$): 61.7 (CH$_2$-5′); 70.6 (CH-3′); 73.5 (CH-2′); 84.7 (CH-4′); 85.7 (CH-1′); 101.3 (CH-5); 108.3 (C-4a); 123.2 (CH-6); 125.7 (CH-3-naphthyl); 126.5 and 127.1 (CH-6,7-naphthyl); 127.6 (CH-5-naphthyl); 128.05 and 128.12 (CH-1,4-naphthyl); 128.9 (CH-8-naphthyl); 132.8 (C-8a-naphthyl); 133.5 (C-4a-naphthyl); 135.6 (C-2-naphthyl); 155.1 (C-7a); 157.1 (C-4); 159.9 (C-2). HR-ESI-MS: *found*: 393.1556 ([M + H]$^+$, calcd for $C_{21}H_{21}O_4N_4^+$: 393.1557); HR-ESI-MS: *found*: 415.1374 ([M + Na]$^+$, calcd for $C_{21}H_{20}O_4N_4Na^+$: 415.1377).

2-Amino-4-(benzofuran-2-yl)-7-(*β*-D-ribofuranosyl)-7*H*-pyrrolo[2,3-*d*]pyrimidine (10C.8)

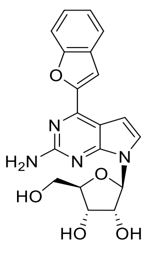

Nucleoside **8** (81.8 mg, 0.27 mmol) was reacted with benzofuran-2-ylboronic acid (66.1 mg, 0.41 mmol) for 1 h at 100 °C according to the GP A. HPFC (SiO_2, DCM/MeOH 1:0 → 9:1) gave **10C.8** (97.2 mg, 93 %) as a yellow powder. ^{1}H NMR (500 MHz, DMSO-d_6): 3.54 (btd, 1H, J_{gem} = 11.8 Hz, $J_{5'a,OH} = J_{5'a,4'}$ = 4.8 Hz, H-5′a); 3.61 (btd, 1H, J_{gem} = 11.8 Hz, $J_{5'b,OH} = J_{5'b,4'}$ = 4.7 Hz, H-5′b); 3.87 (td, 1H, $J_{4',5'a} = J_{4',5'b}$ = 4.1 Hz, $J_{4',3'}$ = 3.3 Hz, H-4′); 4.09 (m, 1H, H-3′); 4.37 (q, 1H, $J_{2',1'} = J_{2',OH} = J_{2',3'}$ = 5.6 Hz, H-2′); 5.02 (t, 1H, $J_{OH,5'a} = J_{OH,5'b}$ = 5.4 Hz, OH-5′); 5.12 (bs, 1H, OH-3′); 5.32 (bd, 1H, $J_{OH,2'}$ = 5.9 Hz, OH-2′); 6.10 (d, 1H, $J_{1',2'}$ = 6.4 Hz, H-1′); 6.43 (bs, 2H, NH_2); 6.96 (d, 1H, $J_{5,6}$ = 3.8 Hz, H-5); 7.34 (btd, 1H, $J_{5,6} = J_{5,4}$ = 7.5 Hz, $J_{5,7}$ = 1.0 Hz, H-5-benzofuryl); 7.44 (ddd, 1H, $J_{6,7}$ = 8.4 Hz, $J_{6,5}$ = 7.2 Hz, $J_{6,4}$ = 1.4 Hz, H-6-benzofuryl); 7.48 (d, 1H, $J_{6,5}$ = 3.8 Hz, H-6); 7.72 (d, 1H, $J_{3,7}$ = 1.0 Hz, H-3-benzofuryl); 7.76 (bdq, 1H, $J_{7,6}$ = 8.4 Hz, $J_{7,5} = J_{7,4} = J_{7,3}$ = 0.9 Hz, H-7-benzofuryl); 7.79 (dm, 1H, $J_{4,5}$ = 8.0 Hz, H-4-benzofuryl); ^{13}C NMR (125.7 MHz, DMSO-d_6): 61.7 (CH_2-5′); 70.6 (CH-3′); 73.5 (CH-2′); 84.7 (CH-4′); 85.6 (CH-1′); 101.5 (C-5); 107.1 (C-4a); 108.0 (CH-3-benzofuryl); 111.7 (CH-7-benzofuryl); 122.2 (CH-4-benzofuryl); 123.6 (CH-5-benzofuryl); 123.9 (CH-6); 126.1 (CH-6-benzofuryl); 127.8 (C-3a-benzofuryl); 147.1 (C-4); 154.4 (C-2-benzofuryl); 154.9 (C-7a-benzofuryl); 155.4 (C-7a); 159.8 (C-2). HR-ESI-MS: *found*: 383.1348 ($[M + H]^+$, calcd for $C_{19}H_{19}O_5N_4^+$: 383.1350); HR-ESI-MS: *found*: 405.1169 ($[M + Na]^+$, calcd for $C_{19}H_{18}O_5N_4Na^+$: 405.1169).

2-Amino-4-(5,6,7,8-tetrahydronaphth-1-yl)-7-(*β*-D-ribofuranosyl)-7*H*-pyrrolo[2,3-*d*]pyrimidine (10C.12)

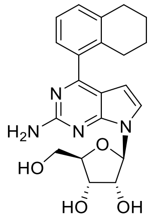

Nucleoside **8** (85.8 mg, 0.29 mmol) was reacted with 5,6,7,8-tetrahydronaphth-1-ylboronic acid (75.3 mg, 0.43 mmol) for 1 h at 100 °C according to the GP A. HPFC (SiO_2, DCM/MeOH 1:0 → 9:1) gave **10C.12** (79.1 mg, 70 %) as a white powder. ^{1}H NMR (500 MHz, DMSO-d_6): 1.65 (m, 2H, H-3-$C_{10}H_{11}$); 1.73 (m, 2H, H-2-$C_{10}H_{11}$); 2.64 (t, 2H, $J_{4,3}$ = 6.4 Hz, H-4-$C_{10}H_{11}$); 2.81 (t, 2H, $J_{1,2}$ = 6.4 Hz, H-1-$C_{10}H_{11}$); 3.51 (ddd, 1H, J_{gem} = 11.8 Hz, $J_{5'a,OH}$ = 5.6 Hz, $J_{5'a,4'}$ = 4.1 Hz, H-5′a); 3.58 (ddd, 1H, J_{gem} = 11.8 Hz, $J_{5'b,OH}$ = 5.4 Hz, $J_{5'b,4'}$ = 4.2 Hz, H-5′a); 3.86 (td, 1H, $J_{4',5'a} = J_{4',5'b}$ = 4.1 Hz, $J_{4',3'}$ = 3.0 Hz, H-4′); 4.07 (td, 1H, $J_{3',2'} = J_{3',OH}$ = 4.8 Hz, $J_{3',4'}$ = 3.0 Hz, H-3′); 4.37 (td, 1H, $J_{2',1'} = J_{2',OH}$ = 6.4 Hz, $J_{2',3'}$ = 5.1 Hz, H-2′); 5.00 (t, 1H, $J_{OH,5'a} = J_{OH,5'b}$ = 5.5 Hz, OH-5′);

5.08 (d, 1H, $J_{OH,3'}$ = 4.5 Hz, OH-3′); 5.28 (d, 1H, $J_{OH,2'}$ = 6.3 Hz, OH-2′); 6.066 (d, 1H, $J_{5,6}$ = 3.8 Hz, H-5); 6.070 (d, 1H, $J_{1',2'}$ = 6.5 Hz, H-1′); 6.27 (bs, 2H, NH_2); 7.11 (dd, 1H, $J_{6,7}$ = 7.2 Hz, $J_{6,8}$ = 1.8 Hz, H-6-$C_{10}H_{11}$); 7.15 (bdd, 1H, $J_{8,7}$ = 7.7 Hz, $J_{8,6}$ = 1.8 Hz, H-8-$C_{10}H_{11}$); 7.19 (t, 1H, $J_{7,8}$ = $J_{7,6}$ = 7.5 Hz, H-7-$C_{10}H_{11}$); 7.28 (d, 1H, $J_{6,5}$ = 3.8 Hz, H-6); ^{13}C NMR (125.7 MHz, DMSO-d_6): 22.4 (CH_2-2-$C_{10}H_{11}$); 22.6 (CH_2-3-$C_{10}H_{11}$); 26.7 (CH_2-4-$C_{10}H_{11}$); 29.4 (CH_2-1-$C_{10}H_{11}$); 61.8 (CH_2-5′); 70.7 (CH-3′); 73.4 (CH-2′); 84.7 (CH-4′); 85.7 (CH-1′); 100.8 (CH-5); 109.9 (C-4a); 122.6 (CH-6); 125.0 (CH-7-$C_{10}H_{11}$); 126.2 (CH-6-$C_{10}H_{11}$); 129.4 (CH-8-$C_{10}H_{11}$); 134.5 (C-4a-$C_{10}H_{11}$); 137.3 (C-8a-$C_{10}H_{11}$); 137.6 (C-5-$C_{10}H_{11}$); 154.1 (C-7a); 159.7 (C-2); 160.6 (C-4). HR-ESI-MS: *found*: 395.1728 ($[M + H]^+$, calcd for $C_{21}H_{23}O_4N_4^+$: 395.1725);

[(5-{[2-Amino-4-(naphth-1-yl)-7*H*-pyrrolo[2,3-*d*]pyrimidin-7-yl]-*β*-D-ribofuranosyl}oxy)phosphonomethyl]phosphonic acid (11C.6)

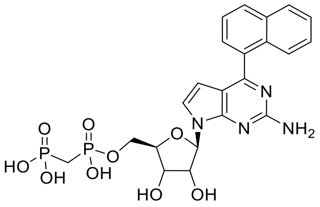

GP B using compound **10C.6** (75.0 mg, 0.19 mmol). HPLC (C-18, H_2O + 0.05 % TFA/MeCN 0 → 80 %) gave **11C.6** (33.8 mg, 32 %) as a pale-yellow powder. ^1H NMR (500 MHz, DMSO-d_6): 2.21 (t, 2H, d, $J_{CH2,P}$ = 20.3 Hz, PCH_2P); 4.02–4.14 (m, 3H, H-4′,5′); 4.18 (dd, 1H, $J_{3',2'}$ = 5.1 Hz, $J_{3',4'}$ = 2.6 Hz, H-3′); 4.46 (dd, 1H, $J_{2',1'}$ = 6.6 Hz, $J_{2',3'}$ = 5.1 Hz, H-3′); 6.12 (d, 1H, $J_{5,6}$ = 3.9 Hz, H-5); 6.13 (d, 1H, $J_{2',1'}$ = 6.6 Hz, H-1′); 7.47 (d, 1H, $J_{6,5}$ = 3.9 Hz, H-6); 7.54 (ddd, 1H, $J_{7,8}$ = 8.3 Hz, $J_{7,6}$ = 6.8 Hz, $J_{7,5}$ = 1.5 Hz, H-7-naphthyl); 7.60 (ddd, 1H, $J_{6,5}$ = 8.2 Hz, $J_{6,7}$ = 6.8 Hz, $J_{6,8}$ = 1.3 Hz, H-6-naphthyl); 7.68 (dd, 1H, $J_{3,4}$ = 8.2 Hz, $J_{3,2}$ = 7.1 Hz, H-3-naphthyl); 7.74 (dd, 1H, $J_{2,3}$ = 7.1 Hz, $J_{2,4}$ = 1.3 Hz, H-2-naphthyl); 8.01 (d, 1H, $J_{8,7}$ = 8.4 Hz, H-8-naphthyl); 8.06 (bd, 1H, $J_{5,6}$ = 8.2 Hz, H-5-naphthyl); 8.13 (d, 1H, $J_{4,3}$ = 8.2 Hz, H-4-naphthyl); ^{13}C NMR (125.7 MHz, DMSO-d_6): 27.5 (t, $J_{C,P}$ = 127.2 Hz, PCH_2P); 64.7 (d, $J_{C,P}$ = 5.4 Hz, CH_2-5′); 70.5 (CH-3′); 73.0 (CH-2′); 82.8 (d, $J_{C,P}$ = 7.4 Hz, CH-4′); 85.9 (CH-1′); 101.6 (CH-5); 111.0 (C-4a); 125.4 and 125.4 (CH-6, CH-3,8-naphthyl); 126.4 (CH-6-naphthyl); 126.8 (CH-7-naphthyl); 127.9 (CH-2-naphthyl); 128.4 (CH-5-naphthyl); 130.1 (CH-4-naphthyl); 130.1 (C-8a-naphthyl); 133.3 (C-4a-naphthyl); 154.9 (C-7a); 157.6 (C-4). *Carbons C-2 and C-1-naphthyl were not detected*; ^{31}P NMR (202.4 MHz, DMSO-d_6): 19.24 and 16.01 (2×s, 2×1P, PCH_2P). HR-ESI-MS: *found*: 549.0941 ($[M–H]^–$, calcd for $C_{22}H_{23}O_9N_4P_2^–$: 549.0946).

[(5-{[2-Amino-4-(naphthalen-2-yl)-7*H*-pyrrolo[2,3-*d*]pyrimidin-7-yl]-*β*-D-ribofuranosyl}oxy)phosphonomethyl]phosphonic acid (11C.7)

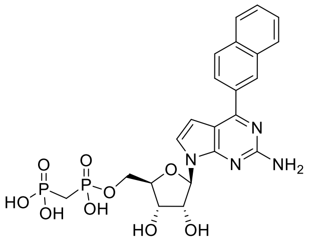

GP B using compound **10C.7** (76.6 mg, 0.20 mmol). HPLC (C-18, H_2O + 0.05 % TFA/MeCN 0 → 80 %) gave **11C.7** (15 mg, 14 %) as a yellow powder. ^{1}H NMR (500 MHz, DMSO-d$_6$): 2.23 (t, 2H, d, $J_{CH2,P}$ = 20.2 Hz, PCH_2P); 4.04 (m, 1H, H-4′); 4.03–4.16 (m, 2H, H-5′); 4.19 (bdd, 1H, $J_{3',2'}$ = 4.8 Hz, $J_{3',4'}$ = 2.8 Hz, H-3′); 4.46 (t, 1H, $J_{2',3'}$ = $J_{2',1'}$ = 5.8 Hz, H-3′); 6.14 (d, 1H, $J_{2',1'}$ = 6.5 Hz, H-1′); 6.84 (d, 1H, $J_{5,6}$ = 3.9 Hz, H-5); 7.51 (d, 1H, $J_{6,5}$ = 3.9 Hz, H-6); 7.58–7.64 (m, 2H, H-6,7-naphthyl); 8.01 (m, 1H, H-5-naphthyl); 8.09 (d, 1H, $J_{4,3}$ = 8.6 Hz, H-4-naphthyl); 8.11–8.20 (m, 1H, H-3,8-naphthyl); 8.61 (bs, 1H, H-1-naphthyl); ^{13}C NMR (125.7 MHz, DMSO-d$_6$): 27.5 (bt, $J_{C,P}$ = 128.0 Hz, PCH_2P); 64.7 (d, $J_{C,P}$ = 4.5 Hz, CH_2-5′); 70.5 (CH-3′); 73.1 (CH-2′); 82.6 (d, $J_{C,P}$ = 7.1 Hz, CH-4′); 85.8 (CH-1′); 101.8 (CH-5); 108.4 (C-4a); 124.2 (CH-6); 125.6 (CH-3-naphthyl); 126.7 (CH-7-naphthyl); 127.4 (CH-5-naphthyl); 126.6 (CH-6-naphthyl); 128.2 (CH-4-naphthyl); 128.4 (CH-1-naphthyl); 128.9 (CH-8-naphthyl); 132.7 (C-8a-naphthyl); 133.6 (C-4a-naphthyl); 134.5 (C-2-naphthyl); 155.2 (C-7a); 156.0 (C-4); 159.1 (C-2); ^{31}P NMR (202.4 MHz, DMSO-d$_6$): 19.14 and 16.11 (2×s, 2×1P, PCH_2P). HR-ESI-MS: *found*: 549.0940 ([M–H]$^-$, calcd for $C_{22}H_{23}O_9N_4P_2^-$: 549.0946).

[(5-{[2-Amino-4-(benzofuran-2-yl)-7*H*-pyrrolo[2,3-*d*]pyrimidin-7-yl]-*β*-D-ribofuranosyl}oxy)phosphonomethyl]phosphonic acid (11C.8)

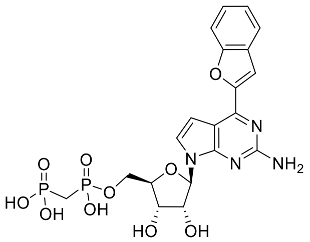

GP B using compound **10C.8** (78.3 mg, 0.21 mmol). HPLC (C-18, H_2O + 0.05 % TFA/MeCN 0 → 80 %) gave **11C.8** (9.8 mg, 9 %) as a yellow powder. ^{1}H NMR (500 MHz, DMSO-d$_6$): 2.25 (t, 2H, $J_{CH2,P}$ = 20.3 Hz, PCH_2P); 4.03 (m, 1H, H-4′); 4.03–4.18 (m, 2H, H-5′); 4.18 (dd, 1H, $J_{3',2'}$ = 4.9 Hz, $J_{3',4'}$ = 3.0 Hz, H-3′); 4.44 (dd, 1H, $J_{2',1'}$ = $J_{2',3'}$ = 5.8 Hz, H-2′); 6.11 (d, 1H, $J_{1',2'}$ = 6.5

Hz, H-1′); 6.98 (d, 1H, $J_{5,6}$ = 3.8 Hz, H-5); 7.35 (btd, 1H, $J_{5,4}$ = $J_{5,6}$ =7.5 Hz, $J_{5,7}$ = 0.9 Hz, H-5-benzofuryl); 7.45 (ddd, 1H, $J_{6,7}$ = 8.3 Hz, $J_{6,5}$ =7.2 Hz, $J_{6,4}$ = 1.3 Hz, H-6-benzofuryl); 7.51 (d, 1H, $J_{6,5}$ = 3.8 Hz, H-6). 7.76 (bs, 1H, H-3-benzofuryl); 7.77 (bd, 1H, $J_{7,6}$ = 8.3 Hz, H-7-benzofuryl); 7.80 (bd, 1H, $J_{4,5}$ = 7.8 Hz, H-4-benzofuryl); ^{13}C NMR (125.7 MHz, DMSO-d$_6$): 27.5 (t, $J_{C,P}$ = 127.0 Hz, PCH$_2$P); 64.8 (bd, $J_{C,P}$ = 5.1 Hz, CH$_2$-5′); 70.5 (CH-3′); 73.0 (CH-2′); 85.5 (d, $J_{C,P}$ = 7.1 Hz, CH-4′); 85.7 (CH-1′); 101.8 (CH-5); 107.1 (C-4a); 108.4 (CH-3-benzofuryl); 111.8 (CH-7-benzofuryl); 122.3 (CH-4-benzofuryl); 123.6 (CH-5-benzofuryl); 124.3 (CH-6); 126.2 (CH-6-benzofuryl); 127.8 (C-3a-benzofuryl); 154.0 (C-2-benzofuryl); 154.9 (C-7a-benzofuryl); 155.5 (C-7a); 159.4 (C-2). *Carbon C-4 was not detected*; ^{31}P NMR (202.4 MHz, DMSO-d$_6$): 15.89 and 19.53 (2×s, 2×1P, PCH$_2$P). HR-ESI-MS: *found*: 539.0736 ([M–H]$^-$, calcd for $C_{20}H_{21}O_{10}N_4P_2^-$: 539.0738).

[(5-{[2-Amino-4-(5,6,7,8-tetrahydronaphth-1-yl)-7*H*-pyrrolo[2,3-*d*]pyrimidin-7-yl]-*β*-D-ribofuranosyl}oxy)phosphonomethyl]phosphonic acid (11C.12)

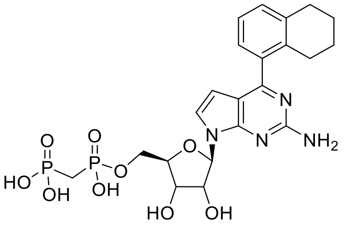

GP B using compound **10C.12** (68.3 mg, 0.17 mmol). HPLC (C-18, H_2O + 0.05 % TFA/MeCN 0 → 80 %) gave **11C.12** (38.3 mg, 40 %) as a white powder. ^{1}H NMR (500 MHz, DMSO-d$_6$): 1.66 (m, 2H, H-3-$C_{10}H_{11}$); 1.74 (m, 2H, H-2-$C_{10}H_{11}$); 2.19 (t, 2H, $J_{CH2,P}$ = 20.2 Hz, PCH$_2$P); 2.63 (t, 2H, $J_{4,3}$ = 6.3 Hz, H-4-$C_{10}H_{11}$); 2.83 (t, 2H, $J_{1,2}$ = 6.3 Hz, H-1-$C_{10}H_{11}$); 4.00–4.11 (m, 3H, H-4,5); 4.16 (dd, 1H, $J_{3',2'}$ = 5.1 Hz, $J_{3',4'}$ = 2.5 Hz, H-3′); 4.45 (dd, 1H, $J_{2',1'}$ = 6.6 Hz, $J_{2',3'}$ = 5.1 Hz, H-2′); 6.07 (d, 1H, $J_{1',2'}$ = 6.6 Hz, H-1′); 6.19 (d, 1H, $J_{5,6}$ = 3.8 Hz, H-5); 7.18–7.28 (m, 3H, H-6,7,8-$C_{10}H_{11}$); 7.48 (bd, 1H, $J_{6,5}$ = 3.6 Hz, H-6); ^{13}C NMR (125.7 MHz, DMSO-d$_6$): 22.3 (CH$_2$-2-$C_{10}H_{11}$); 22.5 (CH$_2$-3-$C_{10}H_{11}$); 26.7 (CH$_2$-4-$C_{10}H_{11}$); 27.5 (t, $J_{C,P}$ = 126.3 Hz, PCH$_2$P); 29.2 (CH$_2$-1-$C_{10}H_{11}$); 64.6 (d, $J_{C,P}$ = 5.1 Hz, CH$_2$-5′); 70.6 (CH-3′); 73.0 (CH-2′); 82.9 (d, $J_{C,P}$ = 7.3 Hz, CH-4′); 85.9 (CH-1′); 101.7 (CH-5); 110.4 (C-4a); 125.4 (CH-6/7-$C_{10}H_{11}$); 125.8 (CH-6); 126.7 (CH-6/7-$C_{10}H_{11}$); 130.6 (CH-8-$C_{10}H_{11}$); 134.9 (C-4a-$C_{10}H_{11}$); 137.7 (C-8a-$C_{10}H_{11}$); 154.7 (C-7a). *Carbon signals and C-2,4 and C-5-$C_{10}H_{11}$ were not detected*; ^{31}P NMR (202.4 MHz, DMSO-d$_6$): 16.19 and 18.88 (2×bs, 2×1P, PCH$_2$P). HR-ESI-MS: *found*: 553.1261 ([M–H]$^-$, calcd for $C_{22}H_{27}O_9N_4P_2^-$: 553.1259).

2-Fluoro-4-(naphth-1-yl)-7-(*β*-D-ribofuranosyl)-7*H*-pyrrolo[2,3-*d*]pyrimidine (13D.6)

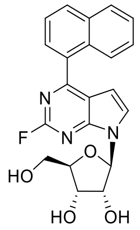

Compound **12**[2] (82.2 mg, 0.27 mmol) was reacted with naphthalene-1-boronic acid (69.8 mg, 0.41 mmol) for 1 h at 100 °C according to the GP A. HPFC (SiO_2, DCM/MeOH 1:0 → 9:1) gave **13D.6** (94.7 mg, 89 %) as a white powder. ^{1}H NMR (500 MHz, DMSO-d$_6$): 3.58 (ddd, 1H, J_{gem} = 11.9 Hz, $J_{5'a,OH}$ = 5.3 Hz, $J_{5'a,4'}$ = 3.9 Hz, H-5′a); 3.65 (ddd, 1H, J_{gem} = 11.9 Hz, $J_{5'b,OH}$ = 5.3 Hz, $J_{5'b,4'}$ = 4.1 Hz, H-5′b); 3.97 (btd, 1H, $J_{4',5'a}$ = $J_{4',5'b}$ = 4.0 Hz, $J_{4',3'}$ = 3.2 Hz, H-4′); 4.13 (td, 1H, $J_{3',2'}$ = $J_{3',OH}$ = 4.7 Hz, $J_{3',4'}$ = 3.1 Hz, H-3′); 4.46 (q, 1H, $J_{2',1'}$ = $J_{2',OH}$ = $J_{2',3'}$ = 6.1 Hz, H-2′); 5.05 (t, 1H, $J_{OH,5'a}$ = $J_{OH,5'b}$ = 5.3 Hz, OH-5′); 5.26 (d, 1H, $J_{OH,3'}$ = 4.8 Hz, OH-3′); 5.47 (d, 1H, $J_{OH,2'}$ = 6.4 Hz, OH-2′); 6.18 (d, 1H, $J_{1',2'}$ = 6.3 Hz, H-1′); 6.50 (d, 1H, $J_{5,6}$ = 3.8 Hz, H-5); 7.55 (ddd, 1H, $J_{7,8}$ = 8.5 Hz, $J_{7,6}$ = 6.8 Hz, $J_{7,5}$ = 1.4 Hz, H-7-naphthyl); 7.61 (ddd, 1H, $J_{6,5}$ = 8.0 Hz, $J_{6,7}$ = 6.8 Hz, $J_{6,8}$ = 1.3 Hz, H-6-naphthyl); 7.70 (dd, 1H, $J_{3,4}$ = 8.3 Hz, $J_{3,2}$ = 7.1 Hz, H-3-naphthyl); 7.81 (dd, 1H, $J_{2,3}$ = 7.1 Hz, $J_{2,4}$ = 1.3 Hz, H-2-naphthyl); 7.91 (d, 1H, $J_{6,5}$ = 3.8 Hz, H-6); 8.06–8.10 (m, 2H, H-5,8-naphthyl); 8.16 (bd, 1H, $J_{4,3}$ = 8.3 Hz, H-4-naphthyl); ^{13}C NMR (125.7 MHz, DMSO-d$_6$): 61.5 (CH$_2$-5′); 70.6 (CH-3′); 74.1 (CH-2′); 85.5 (CH-4′); 86.8 (CH-1′); 101.6 (CH-5); 117.0 (d, $J_{C,F}$ = 3.6 Hz, C-4a); 125.3 (CH-8-naphthyl); 125.4 (CH-3-naphthyl); 126.5 (CH-6-naphthyl); 127.0 (CH-7-naphthyl); 128.4 (d, $J_{C,F}$ = 3.6 Hz, CH-6); 128.4 and 128.5 (CH-2,5-naphthyl); 130.1 (C-8a-naphthyl); 130.4 (CH-4-naphthyl); 133.3 (C-1-naphthyl); 133.5 (C-4a-naphthyl); 153.6 (d, $J_{C,F}$ = 16.2 Hz, C-7a); 158.2 (d, $J_{C,F}$ = 206.8 Hz, C-2); 160.6 (d, $J_{C,F}$ = 15.0 Hz, C-4); ^{19}F NMR (470.4 MHz, DMSO-d$_6$): –53.61 (s, 1F, F-2). HR-ESI-MS: *found*: 396.1356 ($[M + H]^+$, calcd for $C_{21}H_{19}O_4N_3F^+$: 396.1354); HR-ESI-MS: *found*: 418.1172 ($[M + Na]^+$, calcd for $C_{21}H_{18}O_4N_3FNa^+$: 418.1174).

2-Fluoro-4-(naphthalen-2-yl)-7-(*β*-D-ribofuranosyl)-7*H*-pyrrolo[2,3-*d*]pyrimidine (13D.7)

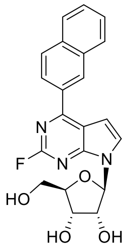

Compound **12**[2] (88.3 mg, 0.29 mmol) was reacted with naphthalene-2-boronic acid (75.0 mg, 0.44 mmol) for 1 h at 100 °C according to the GP A. HPFC (SiO_2, DCM/MeOH 1:0 → 9:1) gave **13D.7** (58.8 mg, 51 %) as a white powder. ^{1}H NMR (500 MHz, DMSO-d$_6$): 3.59 (ddd, 1H, J_{gem} = 11.8 Hz, $J_{5'a,OH}$ = 5.4 Hz, $J_{5'a,4'}$ = 3.9 Hz, H-5´a); 3.67 (ddd, 1H, J_{gem} = 11.8 Hz, $J_{5'b,OH}$ = 5.4 Hz, $J_{5'b,4'}$ = 4.1 Hz, H-5´b); 3.97 (q, 1H, $J_{4',5'a}$ = $J_{4',5'b}$ = $J_{4',3'}$ = 3.8 Hz, H-4´); 4.14 (td, 1H, $J_{3',2'}$ = $J_{3',OH}$ = 5.0 Hz, $J_{3',4'}$ = 3.2 Hz, H-3´); 4.45 (td, 1H, $J_{2',1'}$ = $J_{2',OH}$ = 6.3 Hz, $J_{2',3'}$ = 5.0 Hz, H-2´); 5.07 (t, 1H, $J_{OH,5'a}$ = $J_{OH,5'b}$ = 5.4 Hz, OH-5´); 5.24 (d, 1H, $J_{OH,3'}$ = 5.0 Hz, OH-3´); 5.45 (d, 1H, $J_{OH,2'}$ = 6.4 Hz, OH-2´); 6.17 (d, 1H, $J_{1',2'}$ = 6.1 Hz, H-1´); 7.28 (d, 1H, $J_{5,6}$ = 3.9 Hz, H-5); 7.63 and 7.66 (2×m, 2×1H, H-6,7-naphthyl); 8.02 (d, 1H, $J_{6,5}$ = 3.9 Hz, H-6); 8.04 (dm, 1H, $J_{5,6}$ = 7.7 Hz, H-5-naphthyl); 8.14 (d, 1H, $J_{4,3}$ = 8.7 Hz, H-4-naphthyl); 8.22 (dm, 1H, $J_{8,7}$ = 7.7 Hz, H-8-naphthyl); 8.29 (dd, 1H, $J_{3,4}$ = 8.7 Hz, $J_{3,1}$ = 1.8 Hz, H-3-naphthyl); 8.80 (d, 1H, $J_{1,3}$ = 1.8 Hz, H-1-naphthyl); ^{13}C NMR (125.7 MHz, DMSO-d$_6$): 61.4 (CH$_2$-5´); 70.5 (CH-3´); 74.1 (CH-2´); 85.4 (CH-4´); 86.8 (CH-1´); 102.1 (CH-5); 114.4 (d, $J_{C,F}$ = 3.6 Hz, C-4a); 125.3 (CH-3-naphthyl); 126.8, 127.7 and 127.9 (CH-5,6,7-naphthyl); 128.6 and 128.7 (CH-6, CH-4-naphthyl); 129.27 and 129.29 (CH-1,8-naphthyl); 132.8 and 133.6 (C-2,8a-naphthyl); 134.0 (C-4a-naphthyl); 154.2 (d, $J_{C,F}$ = 15.9 Hz, C-7a); 158.4 (d, $J_{C,F}$ = 205.9 Hz, C-2); 158.6 (d, $J_{C,F}$ = 14.6 Hz, C-4); ^{19}F NMR (470.4 MHz, DMSO-d$_6$): –53.64 (s, 1F, F-2). HR-ESI-MS: *found*: 396.1357 ([M + H]$^+$, calcd for $C_{21}H_{19}O_4N_3F^+$: 396.1354); HR-ESI-MS: *found*: 418.1175 ([M + Na]$^+$, calcd for $C_{21}H_{18}O_4N_3FNa^+$: 418.1174).

4-(Benzofuran-2-yl)-2-fluoro-7-(*β*-D-ribofuranosyl)-7*H*-pyrrolo[2,3-*d*]pyrimidine (13D.8)

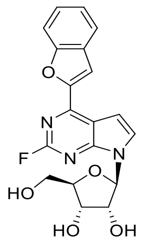

Compound **12** (83.2 mg, 0.27 mmol) was reacted with benzofuran-2-ylboronic acid (66.6 mg, 0.41 mmol) for 1 h at 100 °C according to the GP A. HPFC (SiO_2, DCM/MeOH 1:0 → 9:1) gave **13D.8** (45.6 mg, 43 %) as a white powder. ^{1}H NMR (500 MHz, DMSO-d$_6$): 3.59 (ddd, 1H, J_{gem} = 11.9 Hz, $J_{5'a,OH}$ = 5.3 Hz, $J_{5'a,4'}$ = 3.9 Hz, H-5´a); 3.67 (ddd, 1H, J_{gem} = 11.9 Hz, $J_{5'b,OH}$ = 5.4 Hz, $J_{5'b,4'}$ = 4.0 Hz, H-5´b); 3.96 (q, 1H, $J_{4',5'a}$ = $J_{4',5'b}$ = $J_{4',3'}$ = 3.8 Hz, H-4´); 4.13 (td, 1H, $J_{3',2'}$ = $J_{3',OH}$ = 4.9 Hz, $J_{3',4'}$ = 3.2 Hz, H-3´); 4.43 (q, 1H, $J_{2',1'}$ = $J_{2',OH}$ = $J_{2',3'}$ = 5.9 Hz, H-2´); 5.07 (t, 1H, $J_{OH,5'a}$ = $J_{OH,5'b}$ = 5.3 Hz, OH-5´); 5.24 (d, 1H, $J_{OH,3'}$ = 4.6 Hz, OH-3´); 5.46 (d, 1H, $J_{OH,2'}$ = 5.1 Hz, OH-

2′); 6.13 (d, 1H, $J_{1',2'}$ = 6.1 Hz, H-1′); 7.33 (d, 1H, $J_{5,6}$ = 3.8 Hz, H-5); 7.39 (ddd, 1H, $J_{5,4}$ = 7.9 Hz, $J_{5,6}$ = 7.2 Hz, $J_{5,7}$ = 0.9 Hz, H-5-benzofuryl); 7.52 (ddd, 1H, $J_{6,7}$ = 8.6 Hz, $J_{6,5}$ = 7.2 Hz, $J_{6,4}$ = 1.3 Hz, H-6-benzofuryl); 7.81–7.86 (m, 2H, H-4,7-benzofuryl); 8.01 (d, 1H, $J_{3,7}$ = 0.9 Hz, H-3-benzofuryl); 8.03 (d, 1H, $J_{6,5}$ = 3.8 Hz, H-6); ^{13}C NMR (125.7 MHz, DMSO-d$_6$): 61.4 (CH$_2$-5′); 70.5 (CH-3′); 74.1 (CH-2′); 85.4 (CH-4′); 86.8 (CH-1′); 102.2 (C-5); 110.7 (CH-3-benzofuryl); 112.0 (CH-7-benzofuryl); 112.8 (d, $J_{C,F}$ = 3.2 Hz, C-4a); 122.7 (CH-4-benzofuryl); 124.0 (CH-5-benzofuryl); 127.2 (CH-6-benzofuryl); 127.6 (C-3a-benzofuryl); 129.1 (d, $J_{C,F}$ = 3.3 Hz, CH-6); 148.1 (d, $J_{C,F}$ = 15.8 Hz, C-4); 152.6 (C-2-benzofuryl); 154.6 (d, $J_{C,F}$ = 16.1 Hz, C-7a); 155.5 (C-7a-benzofuryl); 158.3 (d, $J_{C,F}$ = 206.1, C-2); ^{19}F NMR (470.4 MHz, DMSO-d$_6$): –53.69 (s, 1F, F-2). HR-ESI-MS: *found*: 386.1149 ([M + H]$^+$, calcd for $C_{19}H_{17}O_5N_3F^+$: 386.1147). HR-ESI-MS: *found*: 408.0968 ([M + Na]$^+$, calcd for $C_{19}H_{16}O_5N_3FNa^+$: 408.0966).

2-Fluoro-4-(5,6,7,8-tetrahydronaphth-1-yl)-7-(*β*-D-ribofuranosyl)-7*H*-pyrrolo[2,3-*d*]pyrimidine (13D.12)

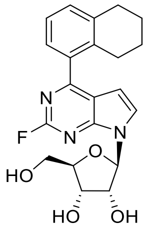

Compound **12** (102.4 mg, 0.34 mmol) was reacted with 5,6,7,8-tetrahydronaphth-1-ylboronic acid (89.0 mg, 0.51 mmol) for 1 h at 100 °C according to the GP A. HPFC (SiO$_2$, DCM/MeOH 1:0 → 9:1) gave **13D.12** (60.2 mg, 45 %) as a white powder. ^{1}H NMR (500 MHz, DMSO-d$_6$): 1.66 (m, 2H, H-3-$C_{10}H_{11}$); 1.75 (m, 2H, H-2-$C_{10}H_{11}$); 2.66 (t, 2H, $J_{4,3}$ = 6.4 Hz, H-4-$C_{10}H_{11}$); 2.84 (t, 2H, $J_{1,2}$ = 6.4 Hz, H-1-$C_{10}H_{11}$); 3.56 (ddd, 1H, J_{gem} = 11.8 Hz, $J_{5'a,OH}$ = 5.4 Hz, $J_{5'a,4'}$ = 3.9 Hz, H-5′a); 3.63 (ddd, 1H, J_{gem} = 11.8 Hz, $J_{5'b,OH}$ = 5.4 Hz, $J_{5'b,4'}$ = 4.1 Hz, H-5′a); 3.95 (q, 1H, $J_{4',5'a}$ = $J_{4',5'b}$ = $J_{4',3'}$ = 3.9 Hz, H-4′); 4.12 (td, 1H, $J_{3',2'}$ = $J_{3',OH}$ = 4.9 Hz, $J_{3',4'}$ = 3.0 Hz, H-3′); 4.44 (td, 1H, $J_{2',1'}$ = $J_{2',OH}$ = 6.3 Hz, $J_{2',3'}$ = 5.0 Hz, H-2′); 5.04 (t, 1H, $J_{OH,5'a}$ = $J_{OH,5'b}$ = 5.4 Hz, OH-5′); 5.23 (d, 1H, $J_{OH,3'}$ = 4.8 Hz, OH-3′); 5.44 (d, 1H, $J_{OH,2'}$ = 6.4 Hz, OH-2′); 6.13 (d, 1H, $J_{1',2'}$ = 6.3 Hz, H-1′); 6.50 (d, 1H, $J_{5,6}$ = 3.8 Hz, H-5); 7.22–7.30 (m, 3H, H-6,7,8-$C_{10}H_{11}$); 7.87 (d, 1H, $J_{6,5}$ = 3.8 Hz, H-6); ^{13}C NMR (125.7 MHz, DMSO-d$_6$): 22.2 (CH$_2$-2-$C_{10}H_{11}$); 22.5 (CH$_2$-3-$C_{10}H_{11}$); 26.8 (CH$_2$-4-$C_{10}H_{11}$); 29.3 (CH$_2$-1-$C_{10}H_{11}$); 61.5 (CH$_2$-5′); 70.6 (CH-3′); 74.0 (CH-2′); 85.5 (CH-4′); 86.7 (CH-1′); 101.6 (CH-5); 116.5 (d, $J_{C,F}$ = 3.6 Hz, C-4a); 125.4 (CH-7-$C_{10}H_{11}$); 127.0 (CH-6-$C_{10}H_{11}$); 128.2 (d, $J_{C,F}$ = 3.5 Hz, CH-6); 130.6 (CH-8-$C_{10}H_{11}$); 135.0 and 135.6 (C-4a,5-$C_{10}H_{11}$); 137.9 (C-

8a-$C_{10}H_{11}$); 153.3 (d, $J_{C,F}$ = 16.1 Hz, C-7a); 157.9 (d, $J_{C,F}$ = 206.9 Hz, C-2); 162.1 (d, $J_{C,F}$ = 14.6 Hz, C-4); ^{19F} NMR (470.4 MHz, DMSO-d_6): –53.79 (s, 1F, F-2). HR-ESI-MS: *found*: 400.1669 ($[M + H]^+$, calcd for $C_{21}H_{23}O_4N_3F^+$: 400.1667); HR-ESI-MS: *found*: 422.1489 ($[M + Na]^+$, calcd for $C_{21}H_{22}O_4N_3FNa^+$: 422.1487).

[(5-{[2-Fluoro-4-(naphth-1-yl)-7*H*-pyrrolo[2,3-*d*]pyrimidin-7-yl]-*β*-D-ribofuranosyl}oxy)phosphonomethyl]phosphonic acid (14D.6)

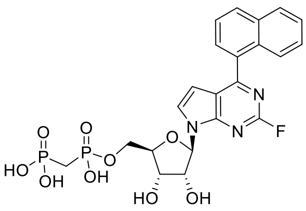

GP B using compound **13D.6** (46.4 mg, 0.12 mmol). HPLC (C-18, H_2O + 0.05 % TFA/MeCN 0 → 80 %) gave **14D.6** (31.9 mg, 49 %) as a yellow powder. ^1H NMR (500 MHz, DMSO-d_6): 2.25 (t, 2H, d, $J_{CH2,P}$ = 20.5 Hz, PCH_2P); 4.09–4.17 (m, 3H, H-4′,5′); 4.21 (dm, 1H, $J_{3',2'}$ = 5.1 Hz, H-3′); 4.48 (dd, 1H, $J_{2',1'}$ = 6.4 Hz, $J_{2',3'}$ = 5.1 Hz, H-2′); 6.21 (d, 1H, $J_{1',2'}$ = 6.4 Hz, H-1′); 6.49 (d, 1H, $J_{5,6}$ = 3.8 Hz, H-5); 7.55 (ddd, 1H, $J_{7,8}$ = 8.3 Hz, $J_{7,6}$ = 6.8 Hz, $J_{7,5}$ = 1.4 Hz, H-7-naphthyl); 7.61 (ddd, 1H, $J_{6,5}$ = 8.2 Hz, $J_{6,7}$ = 6.8 Hz, $J_{6,8}$ = 1.3 Hz, H-6-naphthyl); 7.70 (dd, 1H, $J_{3,4}$ = 8.2 Hz, $J_{3,2}$ = 7.1 Hz, H-3-naphthyl); 7.82 (dd, 1H, $J_{2,3}$ = 7.1 Hz, $J_{2,4}$ = 1.3 Hz, H-2-naphthyl); 7.90 (d, 1H, $J_{6,5}$ = 3.8 Hz, H-6); 8.06–8.11 (m, 2H, H-5,8-naphthyl); 8.16 (dt, 1H, $J_{4,3}$ = 8.3 Hz, $J_{4,2}$ = $J_{4,5}$ = 1.0 Hz, H-4-naphthyl); ^{13}C NMR (125.7 MHz, DMSO-d_6): 27.5 (d, $J_{C,P}$ = 129.0 Hz, PCH_2P); 64.7 (d, $J_{C,P}$ = 5.5 Hz, CH_2-5′); 70.4 (CH-3′); 73.7 (CH-2′); 83.2 (d, $J_{C,P}$ = 7.5 Hz, CH-4′); 86.5 (CH-1′); 101.9 (CH-5); 117.0 (d, $J_{C,F}$ = 3.8 Hz, C-4a); 125.3 and 125.4 (CH-3,8-naphthyl); 126.5 (CH-6-naphthyl); 127.0 (CH-7-naphthyl); 128.3 (d, $J_{C,F}$ = 3.0 Hz, CH-6); 128.48 and 128.49 (CH-2,5-naphthyl); 130.1 (C-8a-naphthyl); 130.5 (CH-4-naphthyl); 133.3 and 133.5 (C-1,4a-naphthyl); 153.8 (d, $J_{C,F}$ = 16.2 Hz, C-7a); 158.22 (d, $J_{C,F}$ = 206.9 Hz, C-2); 160.63 (d, $J_{C,F}$ = 14.9 Hz, C-4); ^{31}P NMR (202.4 MHz, DMSO-d_6): 19.85 and 15.69 (2×d, 2×1P, $J_{P,P}$ = 8.3 Hz, PCH_2P); ^{19}F NMR (470.4 MHz, DMSO-d_6): –53.51 (s, 1F, F-2). HR-ESI-MS: *found*: 552.0737 ($[M–H]^-$, calcd for $C_{22}H_{21}O_9N_3FP_2^-$: 552.0743).

[(5-{[2-Fluoro-4-(naphthalen-2-yl)-7*H*-pyrrolo[2,3-*d*]pyrimidin-7-yl]-*β*-D-ribofuranosyl}oxy)phosphonomethyl]phosphonic acid (14D.7)

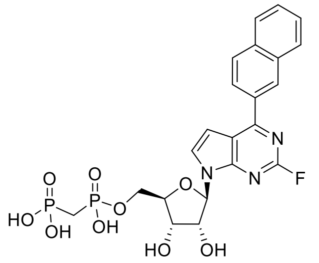

GP B using compound **13D.7** (48.5 mg, 0.12 mmol). HPLC (C-18, H_2O + 0.05 % TFA/MeCN 0 → 80 %) gave **14D.7** (29.8 mg, 44 %) as a pale-yellow powder. ^{1}H NMR (500 MHz, DMSO-d$_6$): 2.28 (t, 2H, d, $J_{CH2,P}$ = 20.5 Hz, PCH_2P); 4.11–4.18 (m, 3H, H-4′,5′); 4.23 (dd, 1H, $J_{3',2'}$ = 5.1 Hz, $J_{3',4'}$ = 2.8 Hz, H-3′); 4.49 (dd, 1H, $J_{2',1'}$ = 6.3 Hz, $J_{2',3'}$ = 5.1 Hz, H-2′); 6.21 (d, 1H, $J_{1',2'}$ = 6.3 Hz, H-1′); 7.28 (d, 1H, $J_{5,6}$ = 3.9 Hz, H-5); 7.60–7.68 (m, 2H, H-6,7-naphthyl); 8.02 (d, 1H, $J_{6,5}$ = 3.9 Hz, H-6); 8.04 (m, 1H, H-5-naphthyl); 8.14 (d, 1H, $J_{4,3}$ = 8.7 Hz, H-4-naphthyl); 8.23 (m, 1H, H-8-naphthyl); 8.30 (dd, 1H, $J_{3,4}$ = 8.6 Hz, $J_{3,1}$ = 1.8 Hz, H-3-naphthyl); 8.81 (d, 1H, $J_{1,3}$ = 1.8 Hz, H-1-naphthyl); ^{13}C NMR (125.7 MHz, DMSO-d$_6$): 27.6 (d, $J_{C,P}$ = 128.7 Hz, PCH_2P); 64.7 (d, $J_{C,P}$ = 5.5 Hz, CH_2-5′); 70.4 (CH-3′); 73.8 (CH-2′); 83.2 (d, $J_{C,P}$ = 7.5 Hz, CH-4′); 86.5 (CH-1′); 102.4 (CH-5); 114.3 (d, $J_{C,F}$ = 3.7 Hz, C-4a); 125.3 (CH-3-naphthyl); 126.8 (CH-7-naphthyl); 127.7 (CH-5-naphthyl); 127.9 (CH-6-naphthyl); 128.5 (d, $J_{C,F}$ = 3.2 Hz, CH-6); 128.7 (CH-4-naphthyl); 129.29 and 129.32 (CH-1,8-naphthyl); 132.8 (C-8a-naphthyl); 133.6 (C-2-naphthyl); 134.0 (C-4a-naphthyl); 154.4 (d, $J_{C,F}$ = 16.0 Hz, C-7a); 158.5 (d, $J_{C,F}$ = 205.8 Hz, C-2); 158.6 (d, $J_{C,F}$ = 14.5 Hz, C-4); ^{31}P NMR (202.4 MHz, DMSO-d$_6$): 19.76 and 15.76 (2×d, 2×1P, $J_{P,P}$ = 8.1 Hz, PCH_2P); ^{19}F NMR (470.4 MHz, DMSO-d$_6$): –53.56 (s, 1F, F-2). HR-ESI-MS: *found*: 552.0741 ([M–H]$^-$, calcd for $C_{22}H_{21}O_9N_3FP_2^-$: 552.0743).

[(5-{[4-(Benzofuran-2-yl)-2-fluoro)-7*H*-pyrrolo[2,3-*d*]pyrimidin-7-yl]-*β*-D-ribofuranosyl}oxy)phosphonomethyl]phosphonic acid (14D.8)

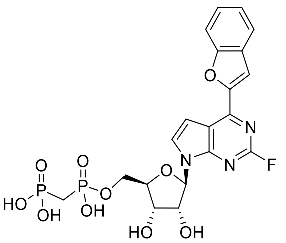

GP B using compound **13D.8** (46.5 mg, 0.12 mmol). HPLC (C-18, H_2O + 0.05 % TFA/MeCN 0 → 80 %) gave **14D.8** (13.3 mg, 20 %) as a pale-yellow powder. ^{1}H NMR (500 MHz, DMSO-d$_6$): 2.27 (t, 2H, $J_{CH2,P}$ = 20.3 Hz, PCH_2P); 4.09–4.17 (m, 3H, H-4′,5′); 4.21 (dd, 1H, $J_{3',2'}$ = 5.1 Hz,

$J_{3',4'}$ = 2.7 Hz, H-3´); 4.46 (dd, 1H, $J_{2',1'}$ = 6.2 Hz, $J_{2',3'}$ = 5.1 Hz, H-2´); 6.17 (d, 1H, $J_{1',2'}$ = 6.2 Hz, H-1´); 7.32 (d, 1H, $J_{5,6}$ = 3.8 Hz, H-5); 7.38 (btd, 1H, $J_{5,4}$ = $J_{5,6}$ = 7.6 Hz, $J_{5,7}$ = 0.9 Hz, H-5-benzofuryl); 7.52 (ddd, 1H, $J_{6,7}$ = 8.3 Hz, $J_{6,5}$ = 7.2 Hz, $J_{6,4}$ = 1.3 Hz, H-6-benzofuryl); 7.82–7.86 (m, 2H, H-4,7-benzofuryl); 8.00 (d, 1H, $J_{3,LR}$ = 0.9 Hz, H-7-benzofuryl); 8.03 (d, 1H, $J_{6,5}$ = 3.8 Hz, H-6); ^{13}C NMR (125.7 MHz, DMSO-d_6): 27.5 (t, $J_{C,P}$ = 128.3 Hz, PCH_2P); 64.7 (d, $J_{C,P}$ = 4.4 Hz, CH_2-5´); 70.4 (CH-3´); 73.8 (CH-2´); 83.2 (d, $J_{C,P}$ = 7.2 Hz, CH-4´); 86.5 (CH-1´); 102.5 (CH-5); 110.7 (CH-3-benzofuryl); 112.0 (CH-7-benzofuryl); 112.7 (d, $J_{C,F}$ = 3.5 Hz, C-4a); 122.7 (CH-4-benzofuryl); 124.0 (CH-5-benzofuryl); 1127.2 (CH-6-benzofuryl); 127.6 (C-3a-benzofuryl); 129.1 (d, $J_{C,F}$ = 3.6 Hz, CH-6); 148.1 (d, $J_{C,F}$ = 15.8 Hz, C-4); 152.6 (C-2-benzofuryl); 154.8 (d, $J_{C,F}$ = 16.2 Hz, C-7a); 155.5 (C-7a-benzofuryl); 158.3 (d, $J_{C,F}$ = 206.1 Hz, C-2); ^{31}P NMR (202.4 MHz, DMSO-d_6): 15.87 and 19.64 (2×bs, 2×1P, PCH_2P); ^{19}F NMR (470.4 MHz, DMSO-d_6): –53.63 (s, 1F, F-2). HR-ESI-MS: *found*: 542.0536 ([M–H]$^-$, calcd for $C_{20}H_{19}O_{10}N_3FP_2^-$: 542.0535).

[(5-{[2-Fluoro-4-(5,6,7,8-tetrahydronaphth-1-yl)-7*H*-pyrrolo[2,3-*d*]pyrimidin-7-yl]-*β*-D-ribofuranosyl}oxy)phosphonomethyl]phosphonic acid (14D.12)

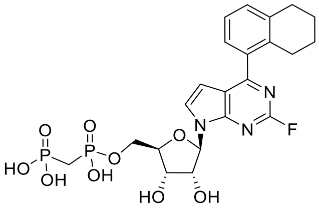

GP B using compound **13D.12** (53.9 mg, 0.14 mmol). HPLC (C-18, H_2O + 0.05 % TFA/MeCN 0 → 80 %) gave **14D.12** (35.9 mg, 48 %) as a white powder. ^{1}H NMR (500 MHz, DMSO-d_6): 1.66 (m, 2H, H-3-$C_{10}H_{11}$); 1.75 (m, 2H, H-2-$C_{10}H_{11}$); 2.24 (t, 2H, $J_{CH2,P}$ = 20.4 Hz, PCH_2P); 2.67 (t, 2H, $J_{4,3}$ = 6.4 Hz, H-4-$C_{10}H_{11}$); 2.84 (t, 2H, $J_{1,2}$ = 6.4 Hz, H-1-$C_{10}H_{11}$); 4.06–4.15 (m, 3H, H-4´,5´); 4.19 (bd, 1H, $J_{3',2'}$ = 5.1 Hz, H-3´); 4.46 (dd, 1H, $J_{2',1'}$ = 6.4 Hz, $J_{2',3'}$ = 5.1 Hz, H-2´); 6.16 (d, 1H, $J_{1',2'}$ = 6.4 Hz, H-1´); 6.49 (d, 1H, $J_{5,6}$ = 3.8 Hz, H-5); 7.22–7.31 (m, 3H, H-6,7,8-$C_{10}H_{11}$); 7.86 (d, 1H, $J_{6,5}$ = 3.8 Hz, H-6); ^{13}C NMR (125.7 MHz, DMSO-d_6): 22.2 (CH_2-2-$C_{10}H_{11}$); 22.5 (CH_2-3-$C_{10}H_{11}$); 26.8 (CH_2-4-$C_{10}H_{11}$); 27.5 (t, $J_{C,P}$ = 129.9 Hz, PCH_2P); 29.3 (CH_2-1-$C_{10}H_{11}$); 64.7 (d, $J_{C,P}$ = 4.2 Hz, CH_2-5´); 70.4 (CH-3´); 73.6 (CH-2´); 83.2 (d, $J_{C,P}$ = 7.0 Hz, CH-4´); 86.4 (CH-1´); 101.8 (CH-5); 116.4 (d, $J_{C,F}$ = 4.0 Hz, C-4a); 125.4 (CH-7-$C_{10}H_{11}$); 127.0 (CH-6-$C_{10}H_{11}$); 128.1 (d, $J_{C,F}$ = 3.3 Hz, CH-6); 130.6 (CH-8-$C_{10}H_{11}$); 135.1 (C-5-$C_{10}H_{11}$); 135.6 (C-4a-$C_{10}H_{11}$); 137.9 (C-8a-$C_{10}H_{11}$); 153.5 (d, $J_{C,F}$ = 16.0 Hz, C-7a); 158.0 (d, $J_{C,F}$ = 206.9 Hz, C-2); 162.2 (d, $J_{C,F}$ = 14.9 Hz, C-4); ^{31}P NMR (202.4 MHz, DMSO-d_6): 15.76 and 19.72 (2×s, 2×1P, PCH_2P); ^{19}F NMR (470.4

MHz, DMSO-d_6): –53.70 (s, 1F, F-2). HR-ESI-MS: *found*: 556.1053 ($[M–H]^-$, calcd for $C_{22}H_{25}O_9N_3FP_2^-$: 556.1056).

2-Methyl-4-(naphth-1-yl)-7-(*β*-D-ribofuranosyl)-7*H*-pyrrolo[2,3-*d*]pyrimidine (17E.6)

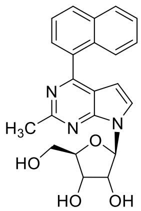

Compound **15** (95.8 mg, 0.32 mmol) was reacted with naphthalene-1-boronic acid (82.5 mg, 0.48 mmol) for 1 h at 100 °C according to the GP A. HPFC (SiO_2, DCM/MeOH 1:0 → 9:1) gave **17E.6** (94.6 mg, 76 %) as a white powder. ^{1}H NMR (500 MHz, DMSO-d_6): 2.76 (s, 3H, CH_3); 3.57 (ddd, 1H, J_{gem} = 11.9 Hz, $J_{5'a,OH}$ = 6.0 Hz, $J_{5'a,4'}$ = 3.9 Hz, H-5′a); 3.65 (ddd, 1H, J_{gem} = 11.9 Hz, $J_{5'b,OH}$ = 5.1 Hz, $J_{5'b,4'}$ = 4.1 Hz, H-5′b); 3.96 (td, 1H, $J_{4',5'a}$ = $J_{4',5'b}$ = 4.0 Hz, $J_{4',3'}$ = 2.8 Hz, H-4′); 4.14 (td, 1H, $J_{3',2'}$ = $J_{3',OH}$ = 4.9 Hz, $J_{3',4'}$ = 2.8 Hz, H-3′); 4.52 (td, 1H, $J_{2',1'}$ = $J_{2',OH}$ = 6.5 Hz, $J_{2',3'}$ = 5.0 Hz, H-2′); 5.31 (t, 1H, $J_{OH,5'a}$ = $J_{OH,5'b}$ = 5.5 Hz, OH-5′); 5.21 (d, 1H, $J_{OH,3'}$ = 4.7 Hz, OH-3′); 5.38 (d, 1H, $J_{OH,2'}$ = 6.5 Hz, OH-2′); 6.27 (d, 1H, $J_{1',2'}$ = 6.6 Hz, H-1′); 6.33 (d, 1H, $J_{5,6}$ = 3.7 Hz, H-5); 7.50 (ddd, 1H, $J_{7,8}$ = 8.5 Hz, $J_{7,6}$ = 6.8 Hz, $J_{7,5}$ = 1.4 Hz, H-7-naphthyl); 7.58 (ddd, 1H, $J_{6,5}$ = 8.2 Hz, $J_{6,7}$ = 6.8 Hz, $J_{6,8}$ = 1.3 Hz, H-6-naphthyl); 7.68 (dd, 1H, $J_{3,4}$ = 8.1 Hz, $J_{3,2}$ = 7.1 Hz, H-3-naphthyl); 7.73 (dd, 1H, $J_{2,3}$ = 7.1 Hz, $J_{2,4}$ = 1.4 Hz, H-2-naphthyl); 7.77 (d, 1H, $J_{6,5}$ = 3.7 Hz, H-6); 8.00 (dm, 1H, $J_{8,7}$ = 8.5 Hz, H-8-naphthyl); 8.05 (bd, 1H, $J_{5,6}$ = 8.3 Hz, H-5-naphthyl); 8.10 (bd, 1H, $J_{4,3}$ = 8.2 Hz, $J_{4,2}$ = $J_{4,5}$ = 1.1 Hz, H-4-naphthyl); ^{13}C NMR (125.7 MHz, DMSO-d_6): 25.7 (CH_3); 61.8 (CH_2-5′); 70.8 (CH-3′); 73.8 (CH-2′); 85.4 (CH-4′); 86.6 (CH-1′); 100.5 (CH-5); 115.9 (C-4a); 125.4 and 125.5 (CH-3,8-naphthyl); 126.2 (CH-6-naphthyl); 126.6 (CH-7-naphthyl); 127.2 (CH-6); 127.8 (CH-2-naphthyl); 128.4 (CH-5-naphthyl); 129.5 (CH-4-naphthyl); 130.3 (C-8a-naphthyl); 133.5 (C-4a-naphthyl); 134.9 (C-1-naphthyl); 152.4 (C-7a); 158.0 (C-4); 159.7 (C-2). HR-ESI-MS: *found*: 392.1607 ($[M + H]^+$, calcd for $C_{22}H_{22}O_4N_3^+$: 392.1605); HR-ESI-MS: *found*: 414.1427 ($[M + Na]^+$, calcd for $C_{22}H_{21}O_4N_3Na^+$: 414.1424).

2-Methyl-4-(benzofuran-2-yl)-7-(*β*-D-ribofuranosyl)-7*H*-pyrrolo[2,3-*d*]pyrimidine (17E.8)

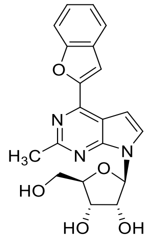

Compound **15** (87.5 mg, 0.29 mmol) was reacted with benzofuran-2-ylboronic acid (70.9 mg, 0.44 mmol) for 1 h at 100 °C according to the GP A. HPFC (SiO_2, DCM/MeOH 1:0 → 9:1) gave **17E.6** (96.2 mg, 77 %) as a white powder. ^{1}H NMR spectra is in agreement with the literature.[2]

[(5-{[2-Methyl-4-(naphth-1-yl)-7*H*-pyrrolo[2,3-*d*]pyrimidin-7-yl]-*β*-D-ribofuranosyl}oxy)phosphonomethyl]phosphonic acid (18E.6)

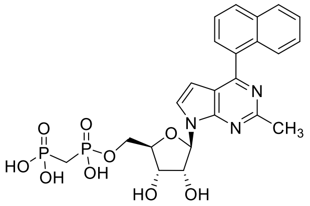

GP B using compound **17E.6** (76.4 mg, 0.20 mmol). HPLC (C-18, H_2O + 0.05 % TFA/MeCN 0 → 80 %) gave **18E.6** (36.4 mg, 34 %) as a white powder. ^{1}H NMR (500 MHz, DMSO-d$_6$): 2.24 (t, 2H, d, $J_{CH2,P}$ = 20.4 Hz, PCH$_2$P); 2.80 (s, 3H, CH$_3$); 4.07–4.17 (m, 3H, H-4′,5′); 4.22 (bdd, 1H, $J_{3',2'}$ = 5.1 Hz, $J_{3',4'}$ = 2.1 Hz, H-3′); 4.53 (dd, 1H, $J_{2',1'}$ = 6.5 Hz, $J_{2',3'}$ = 5.1 Hz, H-3′); 6.35 (d, 1H, $J_{2',1'}$ = 6.5 Hz, H-1′); 6.41 (d, 1H, $J_{5,6}$ = 3.7 Hz, H-5); 7.54 (ddd, 1H, $J_{7,8}$ = 8.6 Hz, $J_{7,6}$ = 6.8 Hz, $J_{7,5}$ = 1.4 Hz, H-7-naphthyl); 7.61 (ddd, 1H, $J_{6,5}$ = 8.2 Hz, $J_{6,7}$ = 6.8 Hz, $J_{6,8}$ = 1.3 Hz, H-6-naphthyl); 7.71 (dd, 1H, $J_{3,4}$ = 8.2 Hz, $J_{3,2}$ = 7.1 Hz, H-3-naphthyl); 7.78 (dd, 1H, $J_{2,3}$ = 7.1 Hz, $J_{2,4}$ = 1.3 Hz, H-2-naphthyl); 7.90 (m, 1H, H-6); 7.98 (bd, 1H, $J_{8,7}$ = 8.6 Hz, H-8-naphthyl); 8.08 (bd, 1H, $J_{5,6}$ = 8.2 Hz, H-5-naphthyl); 8.16 (d, 1H, $J_{4,3}$ = 8.2 Hz, H-4-naphthyl); ^{13}C NMR (125.7 MHz, DMSO-d$_6$): 24.8 (CH$_3$); 27.5 (t, $J_{C,P}$ = 128.8 Hz, PCH$_2$P); 64.7 (d, $J_{C,P}$ = 5.4 Hz, CH$_2$-5′); 70.5 (CH-3′); 73.6 (CH-2′); 83.1 (d, $J_{C,P}$ = 7.4 Hz, CH-4′); 86.2 (CH-1′); 101.5 (CH-5); 115.9 (C-4a); 125.3 and 125.4 (CH-6, CH-3,8-naphthyl); 126.5 (CH-6-naphthyl); 126.9 (CH-7-naphthyl); 128.3 (CH-2-naphthyl); 128.5 (CH-5-naphthyl); 130.1 (CH-4-naphthyl); 130.3 (C-8a-naphthyl); 133.4 (C-4a-naphthyl); 152.6 (C-7a); 156.2 (C-4); 158.8 (C-2). *Carbons CH-6 and C-1-naphthyl were not detected*; ^{31}P NMR (202.4 MHz, DMSO-d$_6$): 19.73 and 15.73 (2×d, 2×1P, $J_{P,P}$ = 7.8 Hz, PCH$_2$P). HR-ESI-MS: *found*: 548.0994 ([M–H]$^-$, calcd for $C_{23}H_{24}O_9N_3P_2^-$: 548.0993).

[(5-{[2-Methyl-4-(naphthalen-2-yl)-7*H*-pyrrolo[2,3-*d*]pyrimidin-7-yl]-*β*-D-ribofuranosyl}oxy)phosphonomethyl]phosphonic acid (18E.7)

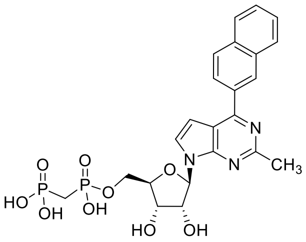

Compound **16** (50.7 mg, 0.11 mmol) was reacted with naphthalene-2-boronic acid (28.6 mg, 0.17 mmol) for 1 h at 90 °C according to the GP A. HPLC (C-18, H_2O + 0.05 % TFA/MeCN 0 → 80 %) gave product **18E.7** (47.8 mg, 79 %) as a pale-yellow powder. ^{1}H NMR (500 MHz, DMSO-d$_6$): 2.27 (t, 2H, d, $J_{CH2,P}$ = 20.4 Hz, PCH$_2$P); 2.79 (s, 3H, CH$_3$); 4.07–4.19 (m, 3H, H-4′,5′); 4.23 (dd, 1H, $J_{3',2'}$ = 5.1 Hz, $J_{3',4'}$ = 2.7 Hz, H-3′); 4.52 (dd, 1H, $J_{2',1'}$ = 6.5 Hz, $J_{2',3'}$ = 5.1 Hz, H-2′); 6.34 (d, 1H, $J_{2',1'}$ = 6.5 Hz, H-1′); 7.12 (d, 1H, $J_{5,6}$ = 3.8 Hz, H-5); 7.58–7.66 (m, 2H, H-6,7-naphthyl); 7.93 (d, 1H, $J_{6,5}$ = 3.8 Hz, H-6); 8.02 (m, 1H, H-5-naphthyl); 8.12 (d, 1H, $J_{4,3}$ = 8.7 Hz, H-4-naphthyl); 8.19 (m, 1H, H-8-naphthyl); 8.29 (dd, 1H, $J_{3,4}$ = 8.7 Hz, $J_{3,1}$ = 1.7 Hz, H-3-naphthyl); 8.72 (bd, 1H, $J_{1,3}$ = 1.7 Hz, H-1-naphthyl); ^{13}C NMR (125.7 MHz, DMSO-d$_6$): 25.5 (CH$_3$); 27.6 (bt, $J_{C,P}$ = 128.8 Hz, PCH$_2$P); 64.8 (d, $J_{C,P}$ = 5.6 Hz, CH$_2$-5′); 70.5 (CH-3′); 73.6 (CH-2′); 82.9 (d, $J_{C,P}$ = 7.5 Hz, CH-4′); 86.1 (CH-1′); 101.6 (CH-5); 113.3 (C-4a); 125.7 (CH-3-naphthyl); 126.7 (CH-6/7-naphthyl); 127.6 (CH-5,6/7-naphthyl); 128.4 (CH-4-naphthyl); 128.8 (CH-1-naphthyl); 129.1 (CH-8-naphthyl); 132.8 (C-8a-naphthyl); 133.7 (C-4a-naphthyl); 134.5 (C-2-naphthyl); 153.2 (C-7a); 155.5 (C-4); 159.6 (C-2); ^{31}P NMR (202.4 MHz, DMSO-d$_6$): 19.73 and 15.78 (2×d, 2×1P, $J_{P,P}$ = 7.9 Hz, PCH$_2$P). HR-ESI-MS: *found*: 548.0989 ([M–H]$^-$, calcd for $C_{23}H_{24}O_9N_3P_2^-$: 548.0993).

[(5-{[2-Methyl-4-(benzofuran-2-yl)-7*H*-pyrrolo[2,3-*d*]pyrimidin-7-yl]-*β*-D-ribofuranosyl}oxy)phosphonomethyl]phosphonic acid (18E.8)

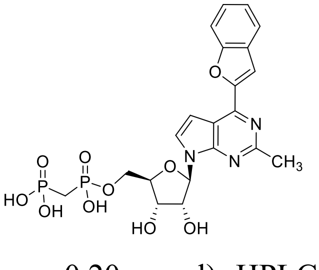

GP B using compound **17E.8** (77.5 mg, 0.20 mmol). HPLC (C-18, H_2O + 0.05 % TFA/MeCN 0 → 80 %) gave **18E.8** (23.9 mg, 22 %) as a yellow powder. ^{1}H NMR (500 MHz, DMSO-d$_6$): 2.27 (t, 2H, $J_{CH2,P}$ = 18.1 Hz, PCH$_2$P); 2.74 (s, 3H, CH$_3$); 4.05–4.18 (m, 3H, H-4′,5′); 4.22 (m, 1H, H-3′); 4.50 (dd, 1H, $J_{2',1'}$ = $J_{2',3'}$ = 5.7 Hz, H-2′); 6.30 (d, 1H, $J_{1',2'}$ = 6.4 Hz, H-1′); 7.21 (d, 1H, $J_{5,6}$

= 3.7 Hz, H-5); 7.36 (bt, 1H, $J_{5,4}$ = $J_{5,6}$ =7.6 Hz, H-5-benzofuryl); 7.47 (bt, 1H, $J_{6,7}$ = 7.8 Hz, H-6-benzofuryl); 7.75 - 7.85 (m, 2H, H-4,7-benzofuryl); 7.85–8.00 (m, 2H, H-6, H-3-benzofuryl); ^{13}C NMR (125.7 MHz, DMSO-d$_6$): 25.7 (CH_3); 27.6 (t, $J_{C,P}$ = 128.4 Hz, PCH_2P); 64.8 (bd, $J_{C,P}$ = 5.3 Hz, CH_2-5′); 70.5 (CH-3′); 73.5 (CH-2′); 82.9 (d, $J_{C,P}$ = 7.3 Hz, CH-4′); 85.9 (CH-1′); 101.6 (CH-5); 108.8 (CH-3-benzofuryl); 111.6 (C-4a); 111.9 (CH-7-benzofuryl); 122.4 (CH-4-benzofuryl); 123.7 (CH-5-benzofuryl); 126.4 (CH-6-benzofuryl); 127.7 (CH-6); 127.8 (C-3a-benzofuryl); 146.1 (C-4); 153.5 (C-7a); 154.1 (C-2-benzofuryl); 155.2 (C-7a-benzofuryl); 160.0 (C-2); ^{31}P NMR (202.4 MHz, DMSO-d$_6$): 15.78 and 19.73 (2×bd, 2×1P, $J_{P,P}$ = 7.8 Hz, PCH_2P). HR-ESI-MS: *found*: 538.0787 ([M–H]$^-$, calcd for $C_{21}H_{22}O_{10}N_3P_2^-$: 538.0786).

[(5-{[2-Methyl-4-(5,6,7,8-tetrahydronaphth-1-yl)-7*H*-pyrrolo[2,3-*d*]pyrimidin-7-yl]-*β*-D-ribofuranosyl}oxy)phosphonomethyl]phosphonic acid (18E.12)

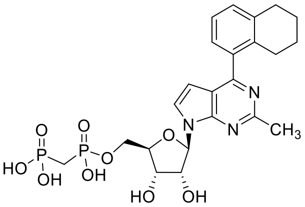

Compound **16** (51.3 mg, 0.11 mmol) was reacted with 5,6,7,8-tetrahydronaphth-1-ylboronic acid (29.6 mg, 0.17 mmol) for 1 h at 90 °C according to the GP A. HPLC (C-18, H_2O + 0.05 % TFA/MeCN 0 → 80 %) gave product **18E.12** (51.7 mg, 83 %) as a white powder. ^{1}H NMR (500 MHz, DMSO-d$_6$): 1.65 (m, 2H, H-3-$C_{10}H_{11}$); 1.76 (m, 2H, H-2-$C_{10}H_{11}$); 2.24 (t, 2H, $J_{CH2,P}$ = 20.4 Hz, PCH_2P); 2.59 (t, 2H, $J_{4,3}$ = 6.4 Hz, H-4-$C_{10}H_{11}$); 2.85 (t, 2H, $J_{1,2}$ = 6.4 Hz, H-1-$C_{10}H_{11}$); 4.05–4.16 (m, 3H, H-4′,5′); 4.21 (bd, 1H, $J_{3',2'}$ = 5.1 Hz, H-3′); 4.51 (dd, 1H, $J_{2',1'}$ = 6.5 Hz, $J_{2',3'}$ = 5.1 Hz, H-2′); 6.30 (d, 1H, $J_{1',2'}$ = 6.5 Hz, H-1′); 6.53 (bs, 1H, H-5); 7.21–7.33 (m, 3H, H-6,7,8-$C_{10}H_{11}$); 7.95 (bs, 1H, H-6); ^{13}C NMR (125.7 MHz, DMSO-d$_6$): 22.2 (CH_2-2-$C_{10}H_{11}$); 22.4 (CH_2-3-$C_{10}H_{11}$); 24.1 (CH_3); 26.7 (CH_2-4-$C_{10}H_{11}$); 27.5 (t, $J_{C,P}$ = 128.9 Hz, PCH_2P); 29.2 (CH_2-1-$C_{10}H_{11}$); 64.7 (d, $J_{C,P}$ = 5.5 Hz, CH_2-5′); 70.5 (CH-3′); 73.6 (CH-2′); 83.3 (d, $J_{C,P}$ = 7.3 Hz, CH-4′); 86.3 (CH-1′); 101.9 (CH-5); 115.4 (C-4a); 125.5 (CH-7-$C_{10}H_{11}$); 127.1 (CH-6-$C_{10}H_{11}$); 129.1 (CH-6); 130.9 (CH-8-$C_{10}H_{11}$); 135.2 (C-4a-$C_{10}H_{11}$); 137.9 (C-8a-$C_{10}H_{11}$); 152.2 (C-7a); 158.1 (C-2). *Carbon signals and C-4, C-5-$C_{10}H_{11}$ were not detected*; ^{31}P NMR (202.4 MHz, DMSO-d$_6$): 15.74 and 19.71 (2×d, 2×1P, $J_{P,P}$ = 7.6 Hz, PCH_2P). HR-ESI-MS: *found*: 552.1304 ([M–H]$^-$, calcd for $C_{23}H_{28}O_9N_3P_2^-$: 552.1306).

6-(5,6,7,8-Tetrahydronaphth-1-yl)-7-(*β*-D-ribofuranosyl)-9*H*-purin-9-yl (30A.12)

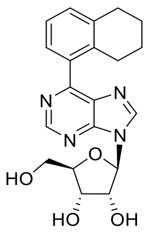

6-Chloro-(*β*-D-ribofuranosyl)-9*H*-purine **28** (111.5 mg, 0.39 mmol) was reacted with 5,6,7,8-tetrahydronaphth-1-ylboronic acid (102.7 mg, 0.58 mmol) for 1 h at 100 °C according to the GP A. HPFC (SiO_2, DCM/MeOH 1:0 → 9:1) gave **30A.6** (127.8 mg, 86 %) as a white powder. ^{1}H NMR (500 MHz, DMSO-d_6): 1.64 (m, 2H, H-3-$C_{10}H_{11}$); 1.75 (m, 2H, H-2-$C_{10}H_{11}$); 2.68 (t, 2H, $J_{4,3}$ = 6.4 Hz, H-4-$C_{10}H_{11}$); 2.84 (t, 2H, $J_{1,2}$ = 6.4 Hz, H-1-$C_{10}H_{11}$); 3.59 (ddd, 1H, J_{gem} = 11.9 Hz, $J_{5'a,OH}$ = 6.0 Hz, $J_{5'a,4'}$ = 4.0 Hz, H-5′a); 3.70 (ddd, 1H, J_{gem} = 11.9 Hz, $J_{5'b,OH}$ = 5.2 Hz, $J_{5'b,4'}$ = 4.1 Hz, H-5′a); 4.00 (q, 1H, $J_{4',5'a} = J_{4',5'b} = J_{4',3'}$ = 3.9 Hz, H-4′); 4.21 (td, 1H, $J_{3',2'} = J_{3',OH}$ = 4.9 Hz, $J_{3',4'}$ = 3.5 Hz, H-3′); 4.69 (q, 1H, $J_{2',1'} = J_{2',OH} = J_{2',3'}$ = 5.8 Hz, H-2′); 5.12 (t, 1H, $J_{OH,5'a}$ = $J_{OH,5'b}$ = 5.5 Hz, OH-5′); 5.27 (d, 1H, $J_{OH,3'}$ = 5.0 Hz, OH-3′); 5.57 (d, 1H, $J_{OH,2'}$ = 6.4 Hz, OH-2′); 6.08 (d, 1H, $J_{1',2'}$ = 5.8 Hz, H-1′); 7.21 -7.26 (m, 2H, H-7,8-$C_{10}H_{11}$); 7.32 (m, 1H, H-6-$C_{10}H_{11}$); 8.82 (s, 1H, H-8); 8.99 (s, 2H, H-2); ^{13}C NMR (125.7 MHz, DMSO-d_6): 22.3 (CH_2-2-$C_{10}H_{11}$); 22.6 (CH_2-3-$C_{10}H_{11}$); 27.0 (CH_2-4-$C_{10}H_{11}$); 29.3 (CH_2-1-$C_{10}H_{11}$); 61.3 (CH_2-5′); 70.4 (CH-3′); 73.6 (CH-2′); 85.8 (CH-4′); 87.6 (CH-1′); 125.1 (CH-7-$C_{10}H_{11}$); 128.0 (CH-6-$C_{10}H_{11}$); 130.3 (CH-8-$C_{10}H_{11}$); 132.3 (C-5); 135.3 and 135.4 (C-4a,5-$C_{10}H_{11}$); 137.3 (C-8a-$C_{10}H_{11}$); 144.9 (CH-8-$C_{10}H_{11}$); 151.4 (C-4); 151.7 (CH-2); 158.1 (C-6). HR-ESI-MS: *found*: 383.1711 ($[M + H]^+$, calcd for $C_{20}H_{23}O_4N_4^+$: 383.1714); HR-ESI-MS: *found*: 405.1529 ($[M + Na]^+$, calcd for $C_{20}H_{22}O_4N_4Na^+$: 405.1533).

[(5-{[6-(Naphthalen-2-yl)-9*H*-purin-9-yl]-*β*-D-ribofuranosyl}oxy)phosphonomethyl]phosphonic acid (31A.7)

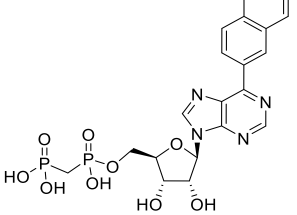

Compound **29** (75.3 mg, 0.17 mmol) was reacted with naphthalene-2-boronic acid (43.7 mg, 0.25 mmol) for 1 h at 80 °C according to the GP A. HPLC (C-18, H_2O + 0.05 % TFA/MeCN 0 → 80 %) gave product **31A.7** (16.2 mg, 22 %) as a yellow powder. ^{1}H NMR (500

MHz, DMSO-d$_6$): 2.28 (t, 2H, d, $J_{CH2,P}$ = 20.3 Hz, PCH$_2$P); 4.13–4.23 (m, 3H, H-4′,5′); 4.31 (dd, 1H, $J_{3',2'}$ = 5.1 Hz, $J_{3',4'}$ = 2.9 Hz, H-3′); 4.71 (t, 1H, $J_{2',1'}$ = $J_{2',3'}$ = 5.4 Hz, H-2′); 6.16 (d, 1H, $J_{2',1'}$ = 5.7 Hz, H-1′); 7.61 (ddd, 1H, $J_{7,8}$ = 7.8 Hz, $J_{7,6}$ = 6.9 Hz, $J_{7,5}$ = 1.5 Hz, H-7-naphthyl); 7.65 (ddd, 1H, $J_{6,5}$ = 8.0 Hz, $J_{6,7}$ = 6.9 Hz, $J_{6,8}$ = 1.5 Hz, H-6-naphthyl); 8.02 (d, 1H, $J_{5,6}$ = 8.0 Hz, H-5-naphthyl); 8.13 (d, 1H, $J_{4,3}$ = 8.8 Hz, H-4-naphthyl); 8.15 (bd, 1H, $J_{8,7}$ = 7.9 Hz, H-8-naphthyl); 8.92 (dd, 1H, $J_{3,4}$ = 8.7 Hz, $J_{3,1}$ = 1.7 Hz, H-3-naphthyl); 8.99 (s, 1H, H-8); 9.08 (s, 1H, H-2); 9.50 (s, 1H, H-1-naphthyl); ^{13}C NMR (125.7 MHz, DMSO-d$_6$): 27.5 (t, $J_{C,P}$ = 128.0 Hz, PCH$_2$P); 64.6 (d, $J_{C,P}$ = 4.9 Hz, CH$_2$-5′); 70.3 (CH-3′); 73.7 (CH-2′); 83.5 (d, $J_{C,P}$ = 6.9 Hz, CH-4′); 87.7 (CH-1′); 125.7 (CH-3-naphthyl); 126.8 (CH-7-naphthyl); 127.1 (CH-5-naphthyl); 127.9 (CH-6-naphthyl); 128.2 (CH-4-naphthyl); 129.3 (CH-8-naphthyl); 130.3 (CH-1-naphthyl); 130.9 (C-5); 132.7 and 132.8 (C-2,8a-naphthyl); 134.2 (C-4a-naphthyl); 144.9 (CH-8); 152.1 (CH-2); 152.5 (C-4); 152.8 (C-6); ^{31}P NMR (202.4 MHz, DMSO-d$_6$): 15.83 and 19.70 (2×bs, 2×1P, PCH$_2$P). HR-ESI-MS: *found*: 535.0786 ([M–H]$^-$, calcd for $C_{21}H_{21}O_9N_4P_2^-$: 535.0789).

[(5-{[6-(5,6,7,8-Tetrahydronaphth-1-yl)-9*H*-purin-9-yl]-*β*-D-ribofuranosyl}oxy)phosphonomethyl]phosphonic acid (31A.12)

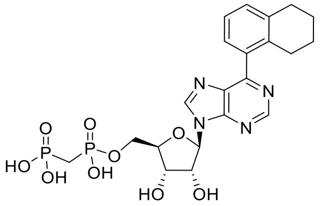

GP B using compound **30A.12** (50.7 mg, 0.13 mmol). HPLC (C-18, H$_2$O + 0.05 % TFA/MeCN 0 → 80 %) gave **31A.12** (13.5 mg, 19 %) as a white powder. ^{1}H NMR (500 MHz, DMSO-d$_6$): 1.65 (m, 2H, H-3-$C_{10}H_{11}$); 1.75 (m, 2H, H-2-$C_{10}H_{11}$); 2.26 (t, 2H, $J_{CH2,P}$ = 20.5 Hz, PCH$_2$P); 2.70 (t, 2H, $J_{4,3}$ = 6.4 Hz, H-4-$C_{10}H_{11}$); 2.84 (t, 2H, $J_{1,2}$ = 6.4 Hz, H-1-$C_{10}H_{11}$); 4.10–4.22 (m, 3H, H-4′,5′); 4.28 (dd, 1H, $J_{3',2'}$ = 5.2 Hz, $J_{3',4'}$ = 2.6 Hz, H-3′); 4.71 (t, 1H, $J_{2',1'}$ = $J_{2',3'}$ = 5.5 Hz, H-2′); 6.11 (d, 1H, $J_{1',2'}$ = 5.9 Hz, H-1′); 7.22–7.27 (m, 2H, H-7,8-$C_{10}H_{11}$); 7.33 (s, 1H, H-6-$C_{10}H_{11}$); 8.83 (s, 1H, H-8); 9.01 (s, 1H, H-2); ^{13}C NMR (125.7 MHz, DMSO-d$_6$): 22.3 (CH$_2$-2-$C_{10}H_{11}$); 22.6 (CH$_2$-3-$C_{10}H_{11}$); 27.0 (CH$_2$-4-$C_{10}H_{11}$); 27.6 (t, $J_{C,P}$ = 128.9 Hz, PCH$_2$P); 29.3 (CH$_2$-1-$C_{10}H_{11}$); 64.6 (d, $J_{C,P}$ = 5.6 Hz, CH$_2$-5′); 70.3 (CH-3′); 73.4 (CH-2′); 83.4 (d, $J_{C,P}$ = 7.4 Hz, CH-4′); 87.1 (CH-1′); 125.0 (CH-7-$C_{10}H_{11}$); 128.0 (CH-6-$C_{10}H_{11}$); 130.4 (CH-8-$C_{10}H_{11}$); 132.1 (C-5); 135.2 and 135.5 (C-4a,5-$C_{10}H_{11}$); 137.3 (C-8a-$C_{10}H_{11}$); 144.7 (CH-8); 151.6 (C-4); 151.8 (CH-2); 158.0 (C-6); ^{31}P

NMR (202.4 MHz, DMSO-d_6): 15.64 and 19.94 (2×d, 2×1P, $J_{P,P}$ = 8.1 Hz, PCH_2P). HR-ESI-MS: *found*: 539.1099 ([M–H]$^-$, calcd for $C_{21}H_{25}O_9N_4P_2^-$: 539.1102).

4-(Phenyl)-7-(*β*-D-2-fluoroarabinosyl)-7*H*-pyrrolo[2,3-*d*]pyrimidine (33A.5)

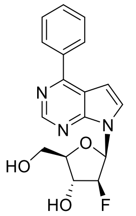

GP C using compound **32**[3] (500 mg, 1.01 mmol). HPFC (SiO_2, cHex/DCM/MeOH 1:1:0 → 1:1:0.23) gave 33A.5 (213.0 mg, 64 %) as a white powder. ^{1}H NMR (500 MHz, DMSO-d_6): 3.66 (bdt, 1H, J_{gem} = 12.0 Hz, $J_{5'a,OH} = J_{5'a,4'}$ = 5.4 Hz, H-5′a); 3.72 (dddd, 1H, J_{gem} = 12.0 Hz, $J_{5'b,OH}$ = 5.4 Hz, $J_{5'b,4'}$ = 4.5 Hz, $J_{5'b,F}$ = 1.4 Hz, H-5′b); 3.88 (bq, 1H, $J_{4',5'a} = J_{4',5'b} = J_{4',3'}$ = 4.9 Hz, H-4′); 4.44 (bdq, 1H, $J_{3',F}$ = 19.1 Hz, $J_{3',2'} = J_{3',4'} = J_{3',OH}$ = 4.6 Hz, H-3′); 5.13 (t, 1H, $J_{OH,5'a} = J_{OH,5'b}$ = 5.6 Hz, OH-5′); 5.24 (ddd, 1H, $J_{2',F}$ = 52.7 Hz, $J_{2',1'}$ = 4.6 Hz, $J_{2',3'}$ = 3.8 Hz, H-2′); 5.98 (d, 1H, $J_{OH,3'}$ = 4.9 Hz, OH-3′); 6.79 (dd, 1H, $J_{1',F}$ = 15.1 Hz, $J_{1',2'}$ = 4.6 Hz, H-1′); 7.02 (d, 1H, $J_{5,6}$ = 3.8 Hz, H-5); 7.58 (m, 1H, H-*p*-Ph); 7.60 (m; 2H, H-*m*-Ph); 7.85 (dd, 1H, $J_{6,5}$ = 3.8 Hz, $J_{6,F}$ = 2.2 Hz, H-6); 8.17 (m, 2H, H-*o*-Ph); 8.92 (s, 1H, H-2); ^{13}C NMR (125.7 MHz, DMSO-d_6): 60.5 (CH_2-5′); 72.8 (d, $J_{C,F}$ = 23.2 Hz, CH-3′); 81.2 (d, $J_{C,F}$ = 16.8 Hz, CH-1′); 83.2 (d, $J_{C,F}$ = 5.2 Hz, CH-4′); 95.9 (d, $J_{C,F}$ = 191.6 Hz, CH-2′); 101.0 (CH-5); 115.0 (C-4a); 128.7 (CH-*o*-Ph); 129.0 (CH-*m*-Ph); 129.0 (bd, $J_{C,F}$ = 3.6 Hz, CH-6); 130.4 (CH-*p*-Ph); 137.4 (C-*i*-Ph); 151.2 (CH-2); 151.6 (C-7a); 156.2 (C-4); ^{19}F NMR (470.4 MHz, DMSO-d_6): -194.68 (dt, 1F, $J_{F,2'}$ = 52.7 Hz, $J_{F,1'} = J_{F,3'}$ = 17.0 Hz, F-2′). HR-ESI-MS: *found*: 330.12470 ([M + H]$^+$, calcd for $C_{17}H_{17}O_3N_3F^+$: 330.12485); HR-ESI-MS: *found*: 352.10662 ([M + Na]$^+$, calcd for $C_{17}H_{16}O_3N_3FNa^+$: 352.10679)

4-(Naphth-1-yl)-7-(2-deoxy-2-fluoro-*β*-D-arabinosyl)-7*H*-pyrrolo[2,3-*d*]pyrimidine (33A.6)

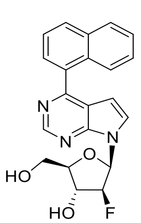

GP C using compound **32** (403 mg, 0.81 mmol). HPFC (SiO_2, cHex/DCM/MeOH 1:1:0 → 1:1:0.23) gave **33A.6** (232.0 mg, 75 %) as a white powder. ^{1}H NMR (500 MHz, DMSO-d_6): 3.65 (bdt, 1H, J_{gem} = 11.9 Hz, $J_{5'a,OH} = J_{5'a,4'}$ = 5.5 Hz, H-5′a); 3.71 (dddd, 1H, J_{gem} = 11.9 Hz, $J_{5'b,OH}$ = 5.8 Hz, $J_{5'b,4'}$ = 4.4 Hz, $J_{5'b,F}$ = 1.6 Hz, H-5′b); 3.89 (bq, 1H, $J_{4',5'a} = J_{4',5'b} = J_{4',3'}$ = 4.9 Hz, H-4′);

4.45 (dtd, 1H, $J_{3',F}$ = 19.0 Hz, $J_{3',OH} = J_{3',4'}$ = 5.2 Hz, $J_{3',2'}$ = 3.8 Hz, H-3´); 5.10 (t, 1H, $J_{OH,5'a} = J_{OH,5'b}$ = 5.6 Hz, OH-5´); 5.27 (ddd, 1H, $J_{2',F}$ = 52.7 Hz, $J_{2',1'}$ = 4.5 Hz, $J_{2',3'}$ = 3.8 Hz, H-2´); 5.99 (d, 1H, $J_{OH,3'}$ = 5.0 Hz, OH-3´); 6.46 (d, 1H, $J_{5,6}$ = 3.8 Hz, H-5); 6.83 (dd, 1H, $J_{1',F}$ = 15.3 Hz, $J_{1',2'}$ = 4.5 Hz, H-1´); 7.51 (ddd; 1H, $J_{7,8}$ = 8.5 Hz, $J_{7,6}$ = 6.8 Hz, $J_{7,5}$ = 1.4 Hz, H-7-naphthyl); 7.59 (ddd; 1H, $J_{6,5}$ = 8.2 Hz, $J_{6,7}$ = 6.8 Hz, $J_{6,8}$ = 1.3 Hz, H-6-naphthyl); 7.69 (dd; 1H, $J_{3,4}$ = 8.2 Hz, $J_{3,2}$ = 7.1 Hz, H-3-naphthyl); 7.77–7.80 (m, 2H, H-6, H-2-naphthyl); 8.04 (bd, 1H, $J_{8,7}$ = 8.5 Hz, H-8-naphthyl); 8.06 (bd, 1H, $J_{5,6}$ = 8.3 Hz, H-5-naphthyl); 8.12 (bd, 1H, $J_{4,3}$ = 8.2 Hz, H-4-naphthyl); 9.01 (s, 1H, H-2); ^{13}C NMR (125.7 MHz, DMSO-d$_6$): 60.5 (CH$_2$-5´); 72.9 (d, $J_{C,F}$ = 23.3 Hz, CH-3´); 81.1 (d, $J_{C,F}$ = 16.8 Hz, CH-1´); 83.2 (d, $J_{C,F}$ = 5.0 Hz, CH-4´); 95.9 (d, $J_{C,F}$ = 191.6 Hz, CH-2´); 100.8 (CH-5); 117.7 (C-4a); 125.4 and 125.6 (CH-3,8-naphthyl); 126.3 (CH-6-naphthyl); 126.7 (CH-7-naphthyl); 128.1 (CH-2-naphthyl); 128.4 (CH-5-naphthyl); 128.9 (bd, $J_{C,F}$ = 3.7 Hz, CH-6); 129.8 (CH-4-naphthyl); 130.3 (CH-8a-naphthyl); 133.6 (C-4a-naphthyl); 134.5 (C-1-naphthyl); 151.0 (CH-2); 151.2 (C-7a); 158.0 (C-4); ^{19}F NMR (470.4 MHz, DMSO-d$_6$): –194.53 (dt, 1F, $J_{F,2'}$ = 52.7 Hz, $J_{F,1'} = J_{F,3'}$ = 17.2 Hz, F-2´). HR-ESI-MS: *found*: 380.14037 ([M + H]$^+$, calcd for $C_{21}H_{19}O_3N_3F^+$: 380.14050); HR-ESI-MS: *found*: 402.12238 ([M + Na]$^+$, calcd for $C_{21}H_{18}O_3N_3FNa^+$: 402.12244).

4-(Naphthalen-2-yl)-7-(2-deoxy-2-fluoro-*β*-D-arabinosyl)-7*H*-pyrrolo[2,3-*d*]pyrimidine (33A.7)

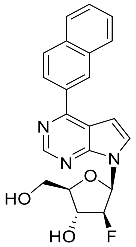

GP C using compound **32** (404 mg, 0.82 mmol). HPFC (SiO$_2$, cHex/DCM/EtOAc 1:1:0 → 1:1:0.23) gave **33A.7** (231.0 mg, 75 %) as a white powder. ^{1}H NMR (500 MHz, DMSO-d$_6$): 3.67 (dt, 1H, J_{gem} = 11.5 Hz, $J_{5'a,OH} = J_{5'a,4'}$ = 5.4 Hz, H-5´a); 3.73 (dddd, 1H, J_{gem} = 11.5 Hz, $J_{5'b,OH}$ = 5.7 Hz, $J_{5'b,4'}$ = 4.2 Hz, $J_{5'b,F}$ = 1.5 Hz, H-5´b); 3.89 (bq, 1H, $J_{4',5'a} = J_{4',5'b} = J_{4',3'}$ = 4.9 Hz, H-4´); 4.46 (dtd, 1H, $J_{3',F}$ = 19.1 Hz, $J_{3',OH} = J_{3',4'}$ = 5.2 Hz, $J_{3',2'}$ = 3.8 Hz, H-3´); 5.13 (t, 1H, $J_{OH,5'a} = J_{OH,5'b}$ = 5.6 Hz, OH-5´); 5.26 (ddd, 1H, $J_{2',F}$ = 52.7 Hz, $J_{2',1'}$ = 4.6 Hz, $J_{2',3'}$ = 3.8 Hz, H-2´); 5.98 (d, 1H, $J_{OH,3'}$ = 5.0 Hz, OH-3´); 6.82 (dd, 1H, $J_{1',F}$ = 14.9 Hz, $J_{1',2'}$ = 4.6 Hz, H-1´); 7.21 (d, 1H, $J_{5,6}$ = 3.8 Hz, H-5); 7.61 (m; 1H, H-7-naphthyl); 7.63 (m; 1H, H-6-naphthyl); 7.90 (dd; 1H, $J_{6,5}$ =

3.8 Hz, $J_{6,F}$ = 2.2 Hz, H-6); 8.02 (m, 1H, H-5-naphthyl); 8.12 (d, 1H, $J_{4,3}$ = 8.7 Hz, H-4-naphthyl); 8.19 (m, 1H, H-8-naphthyl); 8.33 (dd, 1H, $J_{3,4}$ = 8.6 Hz, $J_{3,1}$ = 1.8 Hz, H-3-naphthyl); 8.78 (d, 1H, $J_{1,3}$ = 1.8 Hz, H-1-naphthyl); 8.97 (s, 1H, H-2); ^{13}C NMR (125.7 MHz, DMSO-d$_6$): 60.4 (CH$_2$-5′); 72.8 (d, $J_{C,F}$ = 23.2 Hz, CH-3′); 81.2 (d, $J_{C,F}$ = 16.8 Hz, CH-1′); 83.2 (d, $J_{C,F}$ = 5.4 Hz, CH-4′); 95.9 (d, $J_{C,F}$ = 191.7 Hz, CH-2′); 101.2 (CH-5); 115.2 (C-4a); 125.6 (CH-3-naphthyl); 126.7 (CH-7-naphthyl); 127.5 (CH-6-naphthyl); 127.6 (CH-5-naphthyl); 128.5 (CH-4-naphthyl); 128.8 (CH-1-naphthyl); 129.0 (bd, $J_{C,F}$ = 4.0 Hz, CH-6); 129.1 (CH-1-naphthyl); 132.9 (C-8a-naphthyl); 133.7 (C-4a-naphthyl); 134.8 (C-2-naphthyl); 151.2 (CH-2); 151.6 (C-7a); 156 (C-4); ^{19}F NMR (470.4 MHz, DMSO-d$_6$): –194.73 (dt, 1F, $J_{F,2'}$ = 52.7 Hz, $J_{F,1'}$ = $J_{F,3'}$ = 17.0 Hz, F-2′). HR-ESI-MS: *found*: 380.14073 ([M + H]$^+$, calcd for $C_{21}H_{19}O_3N_3F^+$: 380.14050); HR-ESI-MS: *found*: 402.12255 ([M + Na]$^+$, calcd for $C_{21}H_{18}O_3N_3FNa^+$: 402.12244).

4-(5,6,7,8-Tetrahydronaphth-1-yl)-7-(2-deoxy-2-fluoro-*β*-D-arabinosyl)-7*H*-pyrrolo[2,3-*d*]pyrimidine (33A.12)

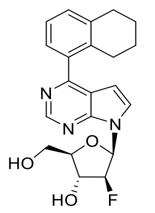

GP C using compound **32** (900 mg, 1.82 mmol). HPFC (SiO$_2$, cHex/DCM/MeOH 1:1:0 → 1:1:0.23) gave **33A.12** (314.0 mg, 45 %) as a white powder. ^{1}H NMR (500 MHz, DMSO-d$_6$): 1.64 (m, 2H, H-7-$C_{10}H_{11}$); 1.75 (m, 2H, H-6-$C_{10}H_{11}$); 2.62 (t, 2H, $J_{8,7}$ = 6.4 Hz, H-8-$C_{10}H_{11}$); 2.84 (t, 2H, $J_{5,6}$ = 6.4 Hz, H-5-$C_{10}H_{11}$); 3.63 (bdt, 1H, J_{gem} = 11.9 Hz, $J_{5'a,4'}$ = $J_{5'a,OH}$ = 5.5 Hz, H-5′a); 3.69 (dddd, 1H, J_{gem} = 11.9 Hz, $J_{5'b,OH}$ = 5.6 Hz, $J_{5'b,4'}$ = 4.5 Hz, $J_{5'b,F}$ = 1.5 Hz, H-5′b); 3.86 (q, 1H, $J_{4',5'a}$ = $J_{4',5'b}$ = $J_{4',3'}$ = 4.9 Hz, H-4′); 4.43 (dtd, 1H, $J_{3',F}$ = 19.0 Hz, $J_{3',4'}$ = $J_{3',OH}$ = 5.1 Hz, $J_{3',2'}$ = 3.8 Hz, H-3′); 5.08 (t, 1H, $J_{OH,5'a}$ = $J_{OH,5'b}$ = 5.6 Hz, OH-5′); 5.24 (ddd, 1H, $J_{2',F}$ = 52.7 Hz, $J_{2',1'}$ = 5.4 Hz, $J_{2',3'}$ = 3.8 Hz, H-2′); 5.96 (d, 1H, $J_{OH,3'}$ = 5.1 Hz, OH-3′); 6.44 (d, 1H, $J_{5,6}$ = 3.8 Hz, H-5); 6.77 (dd, 1H, $J_{1',F}$ = 15.3 Hz, $J_{1',2'}$ = 4.5 Hz, H-1′); 7.19–7.27 (m; 3H, H-2,3,4-$C_{10}H_{11}$); 7.73 (dd, 1H, $J_{6,5}$ = 3.8 Hz, $J_{6,F}$ = 2.3 Hz, H-6); 8.88 (s, 1H, H-2); ^{13}C NMR (125.7 MHz, DMSO-d$_6$): 22.3 (CH$_2$-6-$C_{10}H_{11}$); 22.6 (CH$_2$-7-$C_{10}H_{11}$); 26.8 (CH$_2$-8-$C_{10}H_{11}$); 29.3 (CH$_2$-5-$C_{10}H_{11}$); 60.5 (CH$_2$-5′); 72.9 (d, $J_{C,F}$ = 23.1 Hz, CH-3′); 81.1 (d, $J_{C,F}$ = 16.7 Hz, CH-1′); 83.2 (d, $J_{C,F}$ = 5.0 Hz, CH-4′); 95.9 (d, $J_{C,F}$ = 191.4 Hz, CH-2′); 100.7 (CH-5); 117.1 (C-4a); 125.3 (CH-3-$C_{10}H_{11}$); 126.6 (CH-2-$C_{10}H_{11}$); 128.6 (d, $J_{C,F}$ = 3.8 Hz, CH-6); 130.0 (CH-4-$C_{10}H_{11}$); 134.9 (C-8a-$C_{10}H_{11}$); 136.8

(C-1-$C_{10}H_{11}$); 137.7 (C-4a-$C_{10}H_{11}$); 150.8 (CH-2); 150.9 (C-7a); 159.5 (C-4); ^{19}F NMR (470.4 MHz, DMSO-d_6): –194.55 (dt, 1F, $J_{F,2'}$ = 52.6 Hz, $J_{F,1'}$ = $J_{F,3'}$ = 17.1 Hz, F-2′). HR-ESI-MS: *found*: 384.17166 ([M + H]$^+$, calcd for $C_{21}H_{23}O_3N_3F^+$: 384.17180); HR-ESI-MS: *found*: 406.15356 ([M + Na]$^+$, calcd for $C_{21}H_{22}O_3N_3FNa^+$: 406.15374).

[(5-{[4-(5,6,7,8-Tetrahydronaphth-2-yl)-7*H*-pyrrolo[2,3-*d*]pyrimidin-7-yl]-β-D-2-fluoroarabinosyl-}oxy)phosphonomethyl]phosphonic acid (33A.17)

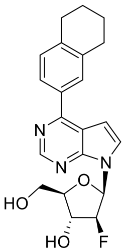

GP C using compound **32** (900 mg, 1.82 mmol). HPFC (SiO_2, cHex/DCM/MeOH 1:1:0 → 1:1:0.23) gave **33A.12** (314.0 mg, 45 %) as a white powder. HPFC (SiO_2, cHex/DCM/MeOH 1:1:0 → 1:1:0.23) **33A.17** (252.0 mg, 81 %) as a white powder. ^{1}H NMR (500 MHz, DMSO-d_6): 2.77–2.83 (m, 4H, H-6,7-$C_{10}H_{11}$); 2.83–2.89 (m, 4H, H-5,8-$C_{10}H_{11}$); 3.65 (bdt, 1H, J_{gem} = 11.9 Hz, $J_{5'a,OH}$ = $J_{5'a,4'}$ = 5.4 Hz, H-5′a); 3.71 (dddd, 1H, J_{gem} = 11.9 Hz, $J_{5'b,OH}$ = 5.5 Hz, $J_{5'b,4'}$ = 4.4 Hz, $J_{5'b,F}$ = 1.5 Hz, H-5′b); 3.86 (bq, 1H, $J_{4',5'a}$ = $J_{4',5'b}$ = $J_{4',3'}$ = 4.8 Hz, H-4′); 4.43 (dtd, 1H, $J_{3',F}$ = 19.1 Hz, $J_{3',OH}$ = $J_{3',4'}$ = 5.2 Hz, $J_{3',2'}$ = 3.8 Hz, H-3′); 5.11(t, 1H, $J_{OH,5'a}$ = $J_{OH,5'b}$ = 5.6 Hz, OH-5′); 5.22 (bddd, 1H, $J_{2',F}$ = 52.7 Hz, $J_{2',1'}$ = 4.6 Hz, $J_{2',3'}$ = 3.8 Hz, H-2′); 5.96 (d, 1H, $J_{OH,3'}$ = 5.0 Hz, OH-3′); 6.77 (dd, 1H, $J_{1',F}$ = 15.1 Hz, $J_{1',2'}$ = 4.6 Hz, H-1′); 7.00 (d, 1H, $J_{5,6}$ = 3.9 Hz, H-5); 7.26 (bd; 1H, $J_{4,3}$ = 7.8 Hz, H-4-$C_{10}H_{11}$); 7.81 (dd, 1H, $J_{6,5}$ = 3.9 Hz, $J_{6,F}$ = 2.2 Hz, H-6); 7.87 (bs, 1H, H-1-$C_{10}H_{11}$); 7.87 (bdd; 1H, $J_{3,4}$ = 7.8 Hz, $J_{3,1}$ = 2.0 Hz, H-3-$C_{10}H_{11}$); 8.87 (s, 1H, H-2); ^{13}C NMR (125.7 MHz, DMSO-d_6): 22.6 and 22.7 (CH_2-6,7-$C_{10}H_{11}$); 28.8 and 28.9 (CH_2-5,8-$C_{10}H_{11}$); 60.4 (CH_2-5′); 72.8 (d, $J_{C,F}$ = 23.2 Hz, CH-3′); 81.1 (d, $J_{C,F}$ = 16.8 Hz, CH-1′); 83.1 (d, $J_{C,F}$ = 5.2 Hz, CH-4′); 95.9 (d, $J_{C,F}$ = 191.5 Hz, CH-2′); 101.1 (CH-5); 114.7 (C-4a); 127.7 (CH-3-$C_{10}H_{11}$); 128.6 (d, $J_{C,F}$ = 3.7 Hz, CH-6);129.2 (CH-1-$C_{10}H_{11}$); 129.5 (CH-4-$C_{10}H_{11}$); 131.7 (C-2-$C_{10}H_{11}$); 137.3 (C-8a-$C_{10}H_{11}$); 139.4 (C-4a-$C_{10}H_{11}$); 151.1 (CH-2); 151.5 (C-7a); 156.3 (C-4); ^{19}F NMR (470.4 MHz, DMSO-d_6): -194.71 (dt, 1F, $J_{F,2'}$ = 52.7 Hz, $J_{F,1'}$ = $J_{F,3'}$ = 17.1 Hz, F-2′). HR-ESI-MS: *found*: 384.17166 ([M + H]$^+$, calcd for $C_{21}H_{23}O_3N_3F^+$: 384.17180); HR-ESI-MS: *found*: 406.15356 ([M + Na]$^+$, calcd for $C_{21}H_{22}O_3N_3FNa^+$: 406.15374)

4-(4-Fluoronaphth-1-yl)-7-(*β*-D-2-fluoroarabinosyl)-7*H*-pyrrolo[2,3-*d*]pyrimidine (33A.23)

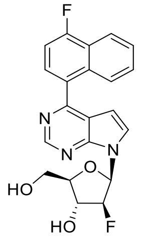

GP C using compound **32** ((402 mg, 0.81 mmol). HPFC (SiO_2, cHex/DCM/MeOH 1:1:0 → 1:1:0.23) gave **33A.23** (245.0 mg, 76 %) as a white powder.

^{1}H NMR (500 MHz, DMSO-d$_6$): 3.65 (bdt, 1H, J_{gem} = 12.0 Hz, $J_{5'a,4'} = J_{5'a,OH}$ = 5.4 Hz, H-5′a); 3.71 (dddd, 1H, J_{gem} = 12.0 Hz, $J_{5'b,OH}$ = 5.5 Hz, $J_{5'b,4'}$ = 4.5 Hz, $J_{5'b,F}$ = 1.5 Hz, H-5′b); 3.88 (bq, 1H, $J_{4',5'a} = J_{4',5'b} = J_{4',3'}$ = 4.9 Hz, H-4′); 4.44 (dtd, 1H, $J_{3',F}$ = 19.0 Hz, $J_{3',4'} = J_{3',OH}$ = 5.2 Hz, $J_{3',2'}$ = 3.8 Hz, H-3′); 5.10 (t, 1H, $J_{OH,5'a} = J_{OH,5'b}$ = 5.6 Hz, OH-5′); 5.27 (dt, 1H, $J_{2',F}$ = 52.7 Hz, $J_{2',1'} = J_{2',3'}$ = 4.2 Hz, H-2′); 5.99 (d, 1H, $J_{OH,3'}$ = 5.0 Hz, OH-3′); 6.49 (d, 1H, $J_{5,6}$ = 3.8 Hz, H-5); 6.83 (dd, 1H, $J_{1',F}$ = 15.2 Hz, $J_{1',2'}$ = 4.6 Hz, H-1′); 7.53 (dd; 1H, $J_{3,F}$ = 10.5 Hz, $J_{3,2}$ = 8.0 Hz, H-3-naphthyl); 7.63 (ddd, 1H, $J_{7,8}$ = 8.6Hz, $J_{7,6}$ = 6.8 Hz, $J_{7,5}$ = 1.4 Hz, H-7-naphthyl); 7.71 (ddd, 1H, $J_{6,5}$ = 8.4 Hz, $J_{6,7}$ = 6.8 Hz, $J_{6,8}$ = 1.2 Hz, H-6-naphthyl); 7.79 (dd, 1H, $J_{6,5}$ = 3.8 Hz, $J_{6,F}$ = 2.3 Hz, H-6); 7.81 (dd, 1H, $J_{2,3}$ = 8.0 Hz, $J_{2,F}$ = 5.6 Hz, H-2-naphthyl); 8.13 (dm, 1H, $J_{8,7}$ = 8.6 Hz, H-8-naphthyl); 8.20 (bd, 1H, $J_{5,6}$ = 8.4 Hz, H-5-naphthyl); 9.01 (s, 1H, H-2); ^{13}C NMR (125.7 MHz, DMSO-d$_6$): 60.5 (CH$_2$-5′); 72.9 (d, $J_{C,F}$ = 23.1 Hz, CH-3′); 81.1 (d, $J_{C,F}$ = 16.8 Hz, CH-1′); 83.2 (d, $J_{C,F}$ = 5.2 Hz, CH-4′); 95.9 (d, $J_{C,F}$ = 191.6 Hz, CH-2′); 100.7 (CH-5); 109.5 (d, $J_{C,F}$ = 20.2 Hz, CH-3-naphthyl); 117.7 (C-4a); 120.3 (d, $J_{C,F}$ = 5.5 Hz, CH-5-naphthyl); 123.2 (d, $J_{C,F}$ = 16.2 Hz, C-4a-naphthyl); 125.8 (d, $J_{C,F}$ = 2.5 Hz, CH-8-naphthyl); 127.1 (d, $J_{C,F}$ = 1.8 Hz, CH-6-naphthyl); 127.9 (CH-7-naphthyl); 128.6 (d, $J_{C,F}$ = 9.0 Hz, CH-2-naphthyl); 129.0 (d, $J_{C,F}$ = 3.9 Hz, CH-6); 131.1 (d, $J_{C,F}$ = 4.2 Hz, C-1-naphthyl); 131.9 (d, $J_{C,F}$ = 4.7 Hz, C-8a-naphthyl); 151.0 (CH-2); 151.2 (C-7a); 157.2 (C-4); 158.6 (d, $J_{C,F}$ = 252.9 Hz, C-4-naphthyl) ; ^{19}F NMR (470.4 MHz, DMSO-d$_6$): -194.56 (dt, 1F, $J_{F,2'}$ = 52.7 Hz, $J_{F,1'} = J_{F,3'}$ = 17.1 Hz, F-2′); -116.95 (dd, 1F, $J_{F,3}$ = 10.5 Hz, $J_{F,2}$ = 5.6 Hz, F-4-naphthyl). HR-ESI-MS: *found*: 398.13098 ([M + H]$^+$, calcd for $C_{21}H_{18}O_3N_3F_2^+$: 398.13107); HR-ESI-MS: *found*: 420.11283 ([M + Na]$^+$, calcd for $C_{21}H_{17}O_3N_3F_2Na^+$: 420.11302)

4-(Quinolin-4-yl)-7-(2-deoxy-2-fluoro-*β*-D-arabinosyl)-7*H*-pyrrolo[2,3-*d*]pyrimidine (33A.28)

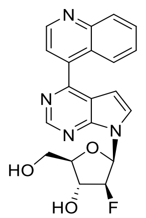

GP C using compound **32** (402 mg, 0.81 mmol). HPFC (SiO_2, cHex/DCM/EtOAc 1:1:0 → 1:1:0.23) gave **33A.28** (219.0 mg, 71 %) as a white powder. ^{1}H NMR (500 MHz, DMSO-d_6): 3.65 (bdt, 1H, J_{gem} = 12.0 Hz, $J_{5'a,4'}$ = $J_{5'a,OH}$ = 5.5 Hz, H-5′a); 3.71 (dddd, 1H, J_{gem} = 12.0 Hz, $J_{5'b,OH}$ = 5.5 Hz, $J_{5'b,4'}$ = 4.5 Hz, $J_{5'b,F}$ = 1.5 Hz, H-5′b); 3.89 (bq, 1H, $J_{4',5'a}$ = $J_{4',5'b}$ = $J_{4',3'}$ = 4.9 Hz, H-4′); 4.45 (dtd, 1H, $J_{3',F}$ = 19.0 Hz, $J_{3',4'}$ = $J_{3',OH}$ = 5.1 Hz, $J_{3',2'}$ = 3.8 Hz, H-3′); 5.11 (t, 1H, $J_{OH,5'a}$ = $J_{OH,5'b}$ = 5.6 Hz, OH-5′); 5.28 (dt, 1H, $J_{2',F}$ = 52.7 Hz, $J_{2',1'}$ = $J_{2',3'}$ = 4.2 Hz, H-2′); 6.00 (d, 1H, $J_{OH,3'}$ = 5.0 Hz, OH-3′); 6.55 (d, 1H, $J_{5,6}$ = 3.8 Hz, H-5); 6.84 (dd, 1H, $J_{1',F}$ = 14.8 Hz, $J_{1',2'}$ = 4.6 Hz, H-1′); 7.61 (ddd; 1H, $J_{6,5}$ = 8.5 Hz, $J_{6,7}$ = 6.8 Hz, $J_{6,8}$ = 1.3 Hz, H-6-quinolinyl); 7.78 (d, 1H, $J_{3,2}$ = 4.4 Hz, H-3-quinolinyl); 7.84 (ddd, 1H, $J_{7,8}$ = 8.5 Hz, $J_{7,6}$ = 6.8 Hz, $J_{7,5}$ = 1.4 Hz, H-7-quinolinyl); 7.86 (m, 1H, H-6); 8.07 (dd, 1H, $J_{5,6}$ = 8.5 Hz, $J_{5,7}$ = 1.3 Hz, H-5-quinolinyl); 8.17 (bd, 1H, $J_{8,7}$ = 8.5 Hz, H-8-quinolinyl); 9.06 (s, 1H, H-2); 9.09 (bd, 1H, $J_{2,3}$ = 4.4 Hz, H-2-quinolinyl); ^{13}C NMR (125.7 MHz, DMSO-d_6): 60.4 (CH_2-5′); 72.8 (d, $J_{C,F}$ = 23.1 Hz, CH-3′); 81.2 (d, $J_{C,F}$ = 16.8 Hz, CH-1′); 83.3 (d, $J_{C,F}$ = 5.3 Hz, CH-4′); 95.9 (d, $J_{C,F}$ = 191.7 Hz, CH-2′); 100.5 (CH-5); 117.6 (C-4a); 122.0 (CH-3-quinolinyl); 125.0 (C-4a-quinolinyl); 125.7 (CH-5-quinolinyl); 127.2 (CH-6-quinolinyl); 129.5 (CH-8-quinolinyl); 129.6 (d, $J_{C,F}$ = 3.7 Hz, CH-6); 129.8 (CH-7-quinolinyl); 142.1 (C-4-quinolinyl); 148.4 (C-8a-quinolinyl); 150.3 (CH-2-quinolinyl); 151.0 (CH-2); 151.2 (C-7a); 155.4 (C-4); ^{19}F NMR (470.4 MHz, DMSO-d_6): -194.62 (dt, 1F, $J_{F,2'}$ = 52.7 Hz, $J_{F,1'}$ = $J_{F,3'}$ = 16.9 Hz, F-2′). HR-ESI-MS: *found*: 381.13567 ($[M + H]^+$, calcd for $C_{20}H_{18}O_3N_4F^+$: 381.13575); HR-ESI-MS: *found*: 403.11746 ($[M + Na]^+$, calcd for $C_{20}H_{17}O_3N_4FNa^+$: 403.11769)

[(5-{[4-Phenyl-7*H*-pyrrolo[2,3-*d*]pyrimidin-7-yl]-*β*-D-2-fluoroarabinosyl}oxy)phosphonomethyl]phosphonic acid (34A.5)

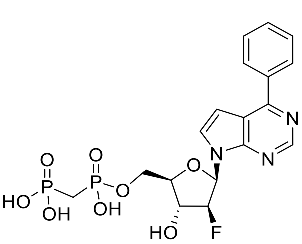

GP B using compound **33A.5** (202 mg, 0.6 mmol). HPFC purification (DEAE Sepharose fast flow, H_2O/TEAB 400 mM 0 → 100 %), HPLC (H_2O 100M TEAB /MeCN 80 % 1M TEAB 0 → 40 %) gave **34A.5** (55 mg, 14 %) as a white powder. ^1H NMR (600.1 MHz, D_2O): 2.23 (t, 2H, $J_{CH2,P}$ = 19.9 Hz, PCH_2P); 4.20–4.29 (m, 3H, H-4′,5′); 4.77 (bdt, 1H, $J_{3',F}$ = 19.4 Hz, $J_{3',2'}$ = $J_{3',4'}$ = 4.4 Hz, H-3′); 5.36 (ddd, 1H, $J_{2',F}$ = 51.9 Hz, $J_{2',1'}$ = 4.7 Hz, $J_{2',3'}$ = 3.9 Hz, H-2′); 6.84 (dd, 1H, $J_{1',F}$ = 14.5 Hz, $J_{1',2'}$ = 4.7 Hz, H-1′); 6.98 (d, 1H, $J_{5,6}$ = 3.9 Hz, H-5); 7.61–7.68 (m; 3H, H-*m*,*p*-Ph); 7.91 (dd, 1H, $J_{6,5}$ = 3.9 Hz, $J_{6,F}$ = 2.4 Hz, H-6); 7.95 (m, 2H, H-*o*-Ph); 8.82 (s, 1H, H-2); ^{13}C NMR (150.9 MHz, D_2O): 29.6 (t, $J_{C,P}$ = 124.8 Hz, PCH_2P); 64.8 (d, $J_{C,P}$ = 5.1 Hz, CH_2-5′); 75.1 (d, $J_{C,F}$ = 24.9 Hz, CH-3′); 83.3 (dd, $J_{C,P}$ = 7.8 Hz, $J_{C,F}$ = 5.4 Hz, CH-4′); 83.8 (d, $J_{C,F}$ = 17.0 Hz, CH-1′); 97.0 (d, $J_{C,F}$ = 192.1 Hz, CH-2′); 104.6 (CH-5); 118.33 (C-4a); 130.9 (CH-*o*-Ph); 131.17 (CH-*m*-Ph); 131.9 (d, $J_{C,F}$ = 4.1 Hz, CH-6); 133.2 (CH-*p*-Ph); 137.1 (C-*i*-Ph); 151.57 (CH-2); 153.1 (C-7a); 158.8 (C-4); ^{19}F NMR (470.4 MHz, D_2O): -195.59 (dt, 1F, $J_{F,2'}$ = 51.9 Hz, $J_{F,1'}$ = $J_{F,3'}$ = 16.96 Hz, F-2′); ^{31}P NMR (202.4 MHz, D_2O): 15.55 and 19.14 (2×d, 2×1P, $J_{P,P}$ = 8.8 Hz, PCH_2P). HR-ESI-MS: *found*: 486.06332 ([M - H]$^-$, calcd for $C_{18}H_{19}O_8N_3FP_2^-$: 486.06369).

[(5-{[4-(Naphtalen-1-yl)-7*H*-pyrrolo[2,3-*d*]pyrimidin-7-yl]-2-deoxy-2-fluoro-*β*-D-arabinosyl-}oxy)phosphonomethyl]phosphonic acid (34A.6)

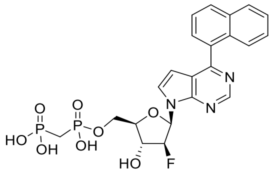

GP B using compound **33A.6** (200 mg, 0.53 mmol). HPFC (DEAE Sepharose fast flow, H_2O/TEAB 400 mM 0 → 100 %), HPLC (H_2O + 100 mM TEAB/80% MeCN + 1M TEAB 0 → 40 %) gave **34A.6** (54 mg, 14 %) as a white powder. ^1H NMR (500 MHz, D_2O): 2.21 (t, 2H, $J_{CH2,P}$ = 19.8 Hz, PCH_2P); 4.21–4.31 (m, 3H, H-4′,5′); 4.81 (m, 1H, H-3′); 5.42 (dt, 1H, $J_{2',F}$ = 51.9 Hz, $J_{2',1'}$ = $J_{2',3'}$ = 4.3 Hz, H-2′); 6.51 (d, 1H, $J_{5,6}$ = 3.8 Hz, H-5); 6.94 (dd, 1H, $J_{1',F}$ = 14.4 Hz, $J_{1',2'}$ = 4.7 Hz, H-1′); 7.51 (ddd; 1H, $J_{7,8}$ = 8.6 Hz, $J_{7,6}$ = 6.8 Hz, $J_{7,5}$ = 1.1 Hz, H-7-naphthyl); 7.64 (btd; 1H, $J_{6,5}$ = $J_{6,7}$ = 7.5 Hz, $J_{6,8}$ = 0.8 Hz, H-6-naphthyl); 7.71 (bdd, 1H, $J_{3,4}$ = 8.1 Hz, $J_{3,2}$ = 7.3 Hz,

H-3-naphthyl); 7.77 (bdd, 1H, $J_{2,3}$ = 7.3 Hz, $J_{2,4}$ = 1.0 Hz, H-2-naphthyl); 7.82 (d, 1H, $J_{8,7}$ = 8.5 Hz, H-8-naphthyl); 7.85 (dd, 1H, $J_{6,5}$ = 3.8 Hz, $J_{6,F}$ = 2.4 Hz, H-6); 8.10 (d, 1H, $J_{5,6}$ = 8.1 Hz, H-5-naphthyl); 8.18 (d, 1H, $J_{4,3}$ = 8.2 Hz, H-4-naphthyl); 8.96 (s, 1H, H-2); ^{13}C NMR (125.7 MHz, D_2O): 29.5 (t, $J_{C,P}$ = 124.5 Hz, PCH_2P); 64.8 (d, $J_{C,P}$ = 4.1 Hz, CH_2-5′); 75.2 (d, $J_{C,F}$ = 24.8 Hz, CH-3′); 83.3 (bt, $J_{C,P}$ = $J_{C,P}$ = 6.4 Hz, CH-4′); 83.8 (d, $J_{C,F}$ = 16.8 Hz, CH-1′); 97.1 (d, $J_{C,F}$ = 192.3 Hz, CH-2′); 105.0 (CH-5); 121.0 (C-4a); 126.9 (CH-8-naphthyl); 127.5 (CH-3-naphthyl); 128.7 (CH-6-naphthyl); 129.2 (CH-7-naphthyl); 130.6 (CH-2,5-naphthyl); 131.9 (d, $J_{C,F}$ = 4.1 Hz, CH-6); 132.1 (C-8a-naphthyl); 132.8 (CH-4-naphthyl); 134.2 (C-1-naphthyl); 134.5 (C-4a-naphthyl); 151.3 (CH-2); 153.0 (C-7a); 159.3 (C-4); ^{19}F NMR (470.4 MHz, D_2O): –195.58 (bdt, 1F, $J_{F,2'}$ = 51.9 Hz, $J_{F,1'}$ = $J_{F,3'}$ = 17.0 Hz, F-2′); ^{31}P NMR (202.4 MHz, D_2O): 15.63 and 19.19 (2×bs, 2×1P, PCH_2P). HR-ESI-MS: *found*: 536.07944 ([M–H]$^-$, calcd for $C_{22}H_{21}O_8N_3FP_2^-$: 536.07934).

[(5-{[4-(Naphtalen-2-yl)-7*H*-pyrrolo[2,3-*d*]pyrimidin-7-yl]-2-deoxy-2-fluoro-*β*-D-arabinosyl-}oxy)phosphonomethyl]phosphonic acid (34A.7)

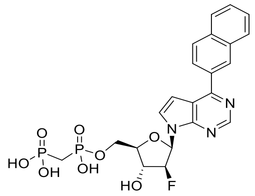

GP B using compound **33A.7** (200 mg, 0.52 mmol). HPFC (DEAE Sepharose fast flow, H_2O/400mM TEAB 0 → 100 %), HPLC (H_2O +100mM TEAB/80% MeCN + 1M TEAB 0 → 40 %) gave **34A.7** (57 mg, 17 %) as a white powder. ^{1}H NMR (600.1 MHz, D_2O): 2.25 (t, 2H, $J_{CH2,P}$ = 19.8 Hz, PCH_2P); 4.23–4.28 (m, 3H, H-4′,5′); 4.75 (bdt, 1H, $J_{3',F}$ = 18.9 Hz, $J_{3',2'}$ = $J_{3',4'}$ = 4.0 Hz, H-3′); 5.29 (ddd, 1H, $J_{2',F}$ = 51.6 Hz, $J_{2',1'}$ = 4.4 Hz, $J_{2',3'}$ = 3.4 Hz, H-2′); 6.58 (dd, 1H, $J_{1',F}$ = 16.2 Hz, $J_{1',2'}$ = 4.4 Hz, H-1′); 6.72 (d, 1H, $J_{5,6}$ = 3.8 Hz, H-5); 7.46 (bddd; 1H, $J_{7,8}$ = 8.0 Hz, $J_{7,6}$ = 6.9 Hz, $J_{7,5}$ = 1.1 Hz, H-7-naphthyl); 7.52 (ddd; 1H, $J_{6,5}$ = 8.1 Hz, $J_{6,7}$ = 6.9 Hz, $J_{6,8}$ = 1.2 Hz, H-6-naphthyl); 7.69 (dd, 1H, $J_{6,5}$ = 3.8 Hz, $J_{6,F}$ = 2.5 Hz, H-6); 7.70–7.76 (m, 4H, H-3,4,5,8-naphthyl); 8.01 (s, 1H, H-1-naphthyl); 8.45 (s, 1H, H-2); ^{13}C NMR (150.9 MHz, D_2O): 29.6 (t, $J_{C,P}$ = 124.6 Hz, PCH_2P); 65.0 (d, $J_{C,P}$ = 4.9 Hz, CH_2-5′); 75.5 (d, $J_{C,F}$ = 25.2 Hz, CH-3′); 83.4 (dd, $J_{C,P}$ = 7.6 Hz, $J_{C,F}$ = 4.7 Hz, CH-4′); 83.8 (d, $J_{C,F}$ = 16.9 Hz, CH-1′); 97.0 (d, $J_{C,F}$ = 191.9 Hz, CH-2′); 104.4 (CH-5); 117.9 (C-4a); 127.1 (CH-3-naphthyl); 128.6 (CH-7-naphthyl); 129.4, 129.6 and 130.4 (CH-4,5,6-naphthyl); 130.4 (CH-8-naphthyl); 130.8 (d, $J_{C,F}$ = 4.4 Hz, CH-6); 131.0 (CH-1-naphthyl); 134.2 (C-2-naphthyl); 135.1 (C-8a-naphthyl); 135.5 (C-4a-naphthyl); 151.9

(CH-2); 152.5 (C-7a); 158.6 (C-4); ^{19}F NMR (470.4 MHz, D_2O): –194.91 (dt, 1F, $J_{F,2'}$ = 51.6 Hz, $J_{F,1'}$ = $J_{F,3'}$ = 17.6 Hz, F-2′); ^{31}P NMR (202.4 MHz, D_2O): 15.57 and 19.24 (2×bs, 2×1P, PCH_2P). HR-ESI-MS: *found*: 536.07899 ($[M–H]^–$, calcd for $C_{22}H_{21}O_8N_3FP_2^–$: 536.07934).

[(5-{[4-(5,6,7,8-Tetrahydronaphth-1-yl)-7*H*-pyrrolo[2,3-*d*]pyrimidin-7-yl]2-deoxy-2-fluoro-*β*-D-arabinosyl-}oxy)phosphonomethyl]phosphonic acid (34A.12)

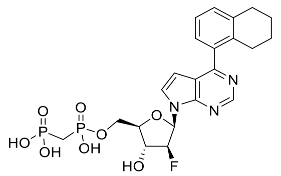

GP B using compound **33A.12** (200 mg, 0.51 mmol). HPFC (DEAE Sepharose fast flow, H_2O/400mM TEAB 0 → 100 %), HPLC (H_2O +100mM TEAB/80% MeCN + 1M TEAB 0 → 40 %) gave **34A.12** (51 mg, 15 %) as a white powder. ^{1}H NMR (600.1 MHz, D_2O): 1.64 (m, 2H, H-7-$C_{10}H_{11}$); 1.78 (m, 2H, H-6-$C_{10}H_{11}$); 2.21 (t, 2H, $J_{CH2,P}$ = 19.9 Hz, PCH_2P); 2.50 (t, 2H, $J_{8,7}$ = 6.4 Hz, H-8-$C_{10}H_{11}$); 2.88 (t, 2H, $J_{5,6}$ = 6.5 Hz, H-5-$C_{10}H_{11}$); 4.19–4.29 (m, 3H, H-4′,5′); 4.77 (bdt, 1H, $J_{3',F}$ = 19.4 Hz, $J_{3',2'}$ = $J_{3',4'}$ = 4.6 Hz, H-3′); 5.38 (dt, 1H, $J_{2',F}$ = 52.0 Hz, $J_{2',1'}$ = $J_{2',3'}$ = 4.3 Hz, H-2′); 6.54 (d, 1H, $J_{5,6}$ = 3.9 Hz, H-5); 6.88 (dd, 1H, $J_{1',F}$ = 14.6 Hz, $J_{1',2'}$ = 4.7 Hz, H-1′); 7.24 (dd; 1H, $J_{2,3}$ = 7.2 Hz, $J_{2,4}$ = 1.7 Hz, H-2-$C_{10}H_{11}$); 7.32 (t, 1H, $J_{3,2}$ = $J_{3,4}$ = 7.4 Hz, H-3-$C_{10}H_{11}$); 7.36 (dd; 1H, $J_{4,3}$ = 7.7 Hz, $J_{4,2}$ = 1.7 Hz, H-4-$C_{10}H_{11}$); 7.83 (dd, 1H, $J_{6,5}$ = 3.9 Hz, $J_{6,F}$ = 2.4 Hz, H-6); 8.84 (s, 1H, H-2); ^{13}C NMR (150.9 MHz, D_2O): 24.3 (CH_2-6-$C_{10}H_{11}$); 24.5 (CH_2-7-$C_{10}H_{11}$); 29.5 (t, $J_{C,P}$ = 124.8 Hz, PCH_2P); 28.7 (CH_2-8-$C_{10}H_{11}$); 31.2 (CH_2-5-$C_{10}H_{11}$); 64.8 (d, $J_{C,P}$ = 5.2 Hz, CH_2-5′); 75.2 (d, $J_{C,F}$ = 24.9 Hz, CH-3′); 83.2 (dd, $J_{C,P}$ = 7.7 Hz, $J_{C,F}$ = 5.6 Hz, CH-4′); 83.7 (d, $J_{C,F}$ = 17.0 Hz, CH-1′); 97.1 (d, $J_{C,F}$ = 192.2 Hz, CH-2′); 104.2 (CH-5); 120.5 (C-4a); 127.6 (CH-3-$C_{10}H_{11}$); 128.6 (CH-2-$C_{10}H_{11}$); 131.2 (d, $J_{C,F}$ = 4.3 Hz, CH-6); 132.7 (CH-4-$C_{10}H_{11}$); 137.6 and 137.7 (C-1,8a-$C_{10}H_{11}$); 140.8 (C-4a-$C_{10}H_{11}$); 151.9 (CH-2); 152.7 (C-7a); 161.7 (C-4); ^{19}F NMR (470.4 MHz, D_2O): –195.61 (dt, 1F, $J_{F,2'}$ = 52.0 Hz, $J_{F,1'}$ = $J_{F,3'}$ = 17.0 Hz, F-2′); ^{31}P NMR (202.4 MHz, D_2O): 15.51 and 19.16 (2×d, 2×1P, $J_{P,P}$ = 9.5 Hz, PCH_2P). HR-ESI-MS: *found*: 540.11016 ($[M–H]^–$, calcd for $C_{22}H_{25}O_8N_3FP_2^–$: 540.11064).

[(5-{[4-(5,6,7,8-Tetrahydronaphth-2-yl)-7*H*-pyrrolo[2,3-*d*]pyrimidin-7-yl]-β-D-2-fluoroarabinosyl-}oxy)phosphonomethyl]phosphonic acid (34A.17)

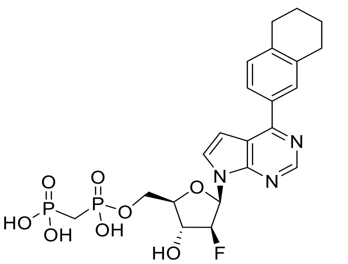

GP B using compound **33A.17** (200 mg, 0.51 mmol). HPFC purification (DEAE Sepharose fast flow, H_2O/TEAB 400 mM 0 → 100 %), HPLC (H_2O 100M TEAB /MeCN 80 % 1M TEAB 0 → 40 %) gave **34A.17** (75 mg, 21 %) as a white powder. ^1H NMR (600.1 MHz, D_2O): 1.70–1.80 (m, 4H, H-6,7-$C_{10}H_{11}$); 2.24 (t, 2H, $J_{CH2,P}$ = 19.8 Hz, PCH_2P); 2.64–2.79 (m, 4H, H-5,8-$C_{10}H_{11}$); 4.20–4.32 (m, 3H, H-4′,5′); 4.77 (bdt, 1H, $J_{3',F}$ = 19.2 Hz, $J_{3',2'}$ = $J_{3',4'}$ = 4.2 Hz, H-3′); 5.33 (dt, 1H, $J_{2',F}$ = 51.9 Hz, $J_{2',1'}$ = $J_{2',3'}$ = 4.2 Hz, H-2′); 6.77 (dd, 1H, $J_{1',F}$ = 14.8 Hz, $J_{1',2'}$ = 4.6 Hz, H-1′); 6.82 (d, 1H, $J_{5,6}$ = 3.8 Hz, H-5); 7.15 (bd; 1H, $J_{4,3}$ = 8.0 Hz, H-4-$C_{10}H_{11}$); 7.39 (bs, 1H, H-1-$C_{10}H_{11}$); 7.45 (bd; 1H, $J_{3,4}$ = 8.0 Hz, H-3-$C_{10}H_{11}$); 7.83 (dd, 1H, $J_{6,5}$ = 3.8 Hz, $J_{6,F}$ = 2.5 Hz, H-6); 8.64 (s, 1H, H-2); ^{13}C NMR (150.9 MHz, D_2O): 24.4 and 24.5 (CH_2-6,7-$C_{10}H_{11}$); 29.6 (t, $J_{C,P}$ = 124.6 Hz, PCH_2P); 30.8 and 30.9 (CH_2-5,8-$C_{10}H_{11}$); 64.9 (d, $J_{C,P}$ = 4.3 Hz, CH_2-5′); 75.2 (d, $J_{C,F}$ = 24.9 Hz, CH-3′); 83.4 (dd, $J_{C,P}$ = 7.8 Hz, $J_{C,F}$ = 5.1 Hz, CH-4′); 83.8 (d, $J_{C,F}$ = 17.1 Hz, CH-1′); 97.0 (d, $J_{C,F}$ = 192.5 Hz, CH-2′); 104.9 (CH-5); 117.6 (C-4a); 127.9 (CH-3-$C_{10}H_{11}$); 131.4 (CH-1-$C_{10}H_{11}$); 131.7 (CH-6,CH-4-$C_{10}H_{11}$); 133.4 (C-2-$C_{10}H_{11}$); 140.2 (C-8a-$C_{10}H_{11}$); 143.5 (C-4a-$C_{10}H_{11}$); 150.9 (CH-2); 152.6 (C-7a); 158.0 (C-4); ^{19}F NMR (470.4 MHz, D_2O): -195.32 (dt, 1F, $J_{F,2'}$ = 51.9 Hz, $J_{F,1'}$ = $J_{F,3'}$ = 17.0 Hz, F-2′); ^{31}P NMR (202.4 MHz, D_2O): 15.56 and 19.13 (2×d, 2×1P, $J_{P,P}$ = 9.4 Hz, PCH_2P). HR-ESI-MS: *found*: 540.11030 ([M - H]$^-$, calcd for $C_{22}H_{25}O_8N_3FP_2^-$: 540.11064)

[(5-{[4-(4-Fluoronaphth-1-yl)-7*H*-pyrrolo[2,3-*d*]pyrimidin-7-yl]-β-D-2-fluoroarabinosyl-}oxy)phosphonomethyl]phosphonic acid (34A.23)

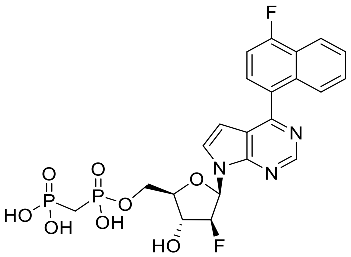

GP B using compound **33A.23** (200 mg, 0.48 mmol). HPFC purification (DEAE Sepharose fast flow, H_2O/TEAB 400 mM 0 → 100 %), HPLC (H_2O 100M TEAB /MeCN 80 % 1M TEAB 0 →

40 %) gave **34A.23** (113 mg, 29 %) as a white powder. ^{1}H NMR (500 MHz, D_2O): 2.22 (t, 2H, $J_{CH2,P}$ = 19.9 Hz, PCH_2P); 4.20–4.31 (m, 3H, H-4′,5′); 4.76 (ddd, 1H, $J_{3',F}$ = 19.2 Hz, $J_{3',4'}$ = 5.2 Hz, $J_{3',2'}$ = 3.8 Hz, H-3′); 5.39 (dt, 1H, $J_{2',F}$ = 51.9 Hz, $J_{2',1'}$ = $J_{2',3'}$ = 4.1 Hz, H-2′); 6.35 (d, 1H, $J_{5,6}$ = 3.9 Hz, H-5); 6.85 (dd, 1H, $J_{1',F}$ = 15.2 Hz, $J_{1',2'}$ = 4.5 Hz, H-1′); 7.31 (dd; 1H, $J_{3,F}$ = 10.4 Hz, $J_{3,2}$ = 8.0 Hz, H-3-naphthyl); 7.46 (ddd; 1H, $J_{7,8}$ = 8.7 Hz, $J_{7,6}$ = 6.9 Hz, $J_{7,5}$ = 1.3 Hz, H-7-naphthyl); 7.60 (dd; 1H, $J_{2,3}$ = 8.0 Hz, $J_{2,F}$ = 5.3 Hz, H-2-naphthyl); 7.64 (bddd; 1H, $J_{6,5}$ = 8.4 Hz, $J_{6,7}$ = 6.9 Hz, $J_{6,8}$ = 0.9 Hz, H-6-naphthyl); 7.70 (dd, 1H, $J_{6,5}$ = 3.9 Hz, $J_{6,F}$ = 2.4 Hz, H-6); 7.79 (d; 1H, $J_{8,7}$ = 8.7 Hz, H-8-naphthyl); 8.21 (d, $J_{5,6}$ = 8.4 Hz, H-5-naphthyl); 8.78 (s, 1H, H-2) ; ^{13}C NMR (125.7 MHz, D_2O): 29.5 (t, $J_{C,P}$ = 124.6 Hz, PCH_2P); 64.9 (d, $J_{C,P}$ = 5.5 Hz, CH_2-5′); 75.3 (d, $J_{C,F}$ = 24.9 Hz, CH-3′); 83.3 (dd, $J_{C,P}$ = 7.9 Hz, $J_{C,F}$ = 5.2 Hz, CH-4′); 83.7 (d, $J_{C,F}$ = 16.9 Hz, CH-1′); 97.1 (d, $J_{C,F}$ = 192.1 Hz, CH-2′); 104.2 (CH-5); 111.1 (d, $J_{C,F}$ = 20.8 Hz, CH-3-naphthyl); 120.7 (C-4a); 122.6 (d, $J_{C,F}$ = 5.7 Hz, CH-5-naphthyl); 129.5 (d, $J_{C,F}$ = 16.6 Hz, C-4a-nphthyl); 127.0 (d, $J_{C,F}$ = 2.4 Hz, CH-8-naphthyl); 128.9 (CH-6-naphthyl); 129.8 (CH-7-naphthyl); 130.8 (d, $J_{C,F}$ = 9.3 Hz, CH-2-naphthyl); 131.0 (d, $J_{C,F}$ = 4.4 Hz, CH-6); 131.5 (d, $J_{C,F}$ = 4.3 Hz, C-1-naphthyl); 133.1 (d, $J_{C,F}$ = 5.0 Hz, C-8a-naphthyl); 152.1 (CH-2); 152.8 (C-7a); 159.7 (C-4); 161.5 (d, $J_{C,F}$ = 253.8 Hz, C-2-naphthyl); ^{19}F NMR (470.4 MHz, D_2O): -194.41 (dt, 1F, $J_{F,2'}$ = 51.9 Hz, $J_{F,1'}$ = $J_{F,3'}$ = 17.2 Hz, F-2′); -116.30 (m, 1F, F-4-naphthyl); ^{31}P NMR (202.4 MHz, D_2O): 15.50 and 19.16 (2×d, 2×1P, $J_{P,P}$ = 9.5 Hz, PCH_2P). HR-ESI-MS: *found*: 554.07019 ([M - H]$^-$, calcd for $C_{20}H_{20}O_8N_3F_2P_2^-$: 554.06992).

[(5-{[4-(Quinolin-4-yl)-7*H*-pyrrolo[2,3-*d*]pyrimidin-7-yl]-2-deoxy-2-fluoro-*β*-D-arabinosyl-}oxy)phosphonomethyl]phosphonic acid (34A.28)

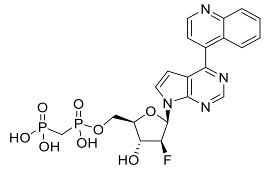

GP B using compound **33A.28** (200 mg, 0.48 mmol). HPFC (DEAE Sepharose fast flow, H_2O/400mM TEAB 0 → 100 %), HPLC (H_2O +100mM TEAB/80% MeCN + 1M TEAB 0 → 40 %) gave **34A.28** (70 mg, 20 %) as a green powder. ^{1}H NMR (600.1 MHz, D_2O): 2.24 (t, 2H, $J_{CH2,P}$ = 19.9 Hz, PCH_2P); 4.23–4.32 (m, 3H, H-4′,5′); 4.76 (md, 1H, H-3′); 5.41 (dt, 1H, $J_{2',F}$ = 51.9 Hz, $J_{2',1'}$ = $J_{2',3'}$ = 4.3 Hz, H-2′); 6.33 (m, 1H, H-5); 6.88 (dd, 1H, $J_{1',F}$ = 14.3 Hz, $J_{1',2'}$ = 4.6 Hz, H-1′); 7.51 (m; 1H, H-6-quinolinyl); 7.82 (m, 1H, H-3-quinolinyl); 7.76 (m, 1H, H-5-quinolinyl); 7.79 (m, 1H, H-6); 7.88 (bddd, 1H, $J_{7,8}$ = 8.6 Hz, $J_{7,6}$ = 6.9 Hz, $J_{7,5}$ = 1.3 Hz, H-7-quinolinyl); 8.17

(d, 1H, $J_{8,7}$ = 8.6 Hz, H-8-quinolinyl); 8.85 (s, 1H, H-2); 9.00 (bd, 1H, $J_{2,3}$ = 4.6 Hz, H-2-quinolinyl); ^{13}C NMR (150.9 MHz, D_2O): 29.6 (t, $J_{C,P}$ = 124.5 Hz, PCH_2P); 64.9 (d, $J_{C,P}$ = 5.2 Hz, CH_2-5′); 75.1 (d, $J_{C,F}$ = 24.8 Hz, CH-3′); 83.2 (t, $J_{C,F}$ = $J_{C,P}$ = 6.6 Hz, CH-4′); 83.7 (d, $J_{C,F}$ = 17.1 Hz, CH-1′); 97.1 (d, $J_{C,F}$ = 192.3 Hz, CH-2′); 103.34 (CH-5); 120.6 (C-4a); 124.2 (CH-3-quinolinyl); 127.2 (C-4a-quinolinyl); 127.4 (CH-5-quinolinyl); 128.7 (CH-8-quinolinyl); 130.2 (CH-6-quinolinyl); 132.1 (d, $J_{C,F}$ = 4.3 Hz, CH-6); 133.5 (CH-7-quinolinyl); 146.2 (C-4-quinolinyl); 147.3 (C-8a-quinolinyl); 150.4 (CH-2-quinolinyl); 152.2 (CH-2); 153.0 (C-7a); 156.4 (C-4); ^{19}F NMR (470.4 MHz, D_2O): –195.53 (dm, 1F, $J_{F,2'}$ = 51.9 Hz, F-2′); ^{31}P NMR (202.4 MHz, D_2O): 15.53 and 19.17 (2×d, 2×1P, $J_{P,P}$ = 9.8 Hz, PCH_2P). HR-ESI-MS: *found*: 537.07432 ([M–H]$^-$, calcd for $C_{21}H_{19}O_4N_3F^-$: 537.07459)

S10 HPLC purity of final compounds

Table S15. UPLC purity of compounds **4A.1–22; 7B.1,6–8,12,23–35; 11C.1,6–8,12, 14D.1,6–8,12; 18E.1,6–8,12; 21F.7; 21G.7; 27H.23; 31A.6,7,12; 34A.5–7,12,17,23,28**

Comp	Method	t_r [min]	Purity [%]	Comp	Method	t_r [min]	Purity [%]
**4A.1**	A	5.523	99.72	**7B.27**	D	8.136	96.24
**4A.2**	A	5.512	100	**7B.28**	D	7.308	99.02
**4A.3**	A	5.976	99.73	**7B.29**	D	7.595	95.53
**4A.4**	A	5.989	99.04	**7B.30**	D	7.184	98.57
**4A.5**	A	5.990	99.60	**7B.31**	D	8.701	97.94
**4A.6**	A	6.620	99.63	**7B.32**	D	6.527	96.92
**4A.7**	A	6.961	99.24	**7B.33**	D	6.332	95.35
**4A.8**	A	6.752	98.86	**7B.34**	D	6.870	98.80
**4A.9**	A	7.376	99.58	**7B.35**	D	8.724	96.62
**4A.10**	A	7.085	99.27	**11C.1**	A	5.403	96.87
**4A.11**	A	7.302	99.35	**11C.6**	A	6.601	97.95
**4A.12**	A	6.927	99.23	**11C.7**	A	6.896	95.95
**4A.13**	A	6.176	99.81	**11C.8**	A	6.631	96.02
**4A.14**	A	6.137	100	**11C.12**	A	6.876	99.34
**4A.15**	A	6.209	99.65	**14D.1**	A	3.143*;5.882	27.01*;72.99
**4A.16**	A	5.284	99.89	**14D.6**	A	6.037*;7.070	1.78*;96.24
**4A.17**	A	5.871	97.35	**14D.7**	A	7.350	98.67
**4A.18**	A	5.880	98.32	**14D.8**	A	6.087*;7.053	5.56*;93.73
**4A.19**	A	5.943	97.61	**14D.12**	A	6.334*;7.334	25.42*;73.83
**4A.20**	A	6.233	97.86	**18E.1**	A	5.782	95.88
**4A.21**	A	5.940	99.49	**18E.6**	A	6.136	99.75
**4A.22**	A	5.669	97.03	**18E.7**	A	7.108	97.63

Continuation of Table S14

Comp	Method	t_r [min]	Purity [%]	Comp	Method	t_r [min]	Purity [%]
**7B.1**	A	6.189	98.87	**18E.8**	A	6.891	97.89
**7B.6**	A	7.238	99.18	**18E.12**	A	7.012	100
**7B.7**	A	7.532	98.55	**21F.7**	A	7.339	97.77
**7B.8**	A	7.260	99.32	**22G.7**	A	7.311	95.95
**7B.12**	A	7.455	98.64	**27H.23**	D	8.045	98.99
**7B.23**	A	7.726	99.31	**31A.6**	A	6.327	95.07
**7B.24**	D	6.954	96.86	**31A.7**	A	6.837	99.35
**7B.25**	B	8.365	98.73	**31.12**	A	6.641	96.05
**7B.26**	C	6.172	98.90				

Mobile phases: H_2O + 10 mM CH_3COONH_4 and ACN, Method: A – 1 %-100 % in 10 min; Mobile phases: H_2O + 12 mM TEA and 80 % ACN + 12 mM TEA, Methods: B – 20 %-100 % in 13 min; C 30 %-100 % in 13 min; B – 1 %-100 % in 13 min
* Hydrolyzed product (C–2 Fluoro substituted compounds are hydrolyzed during the analysis)

S11 Copies of NMR spectra

NMR spectra of compound **2**

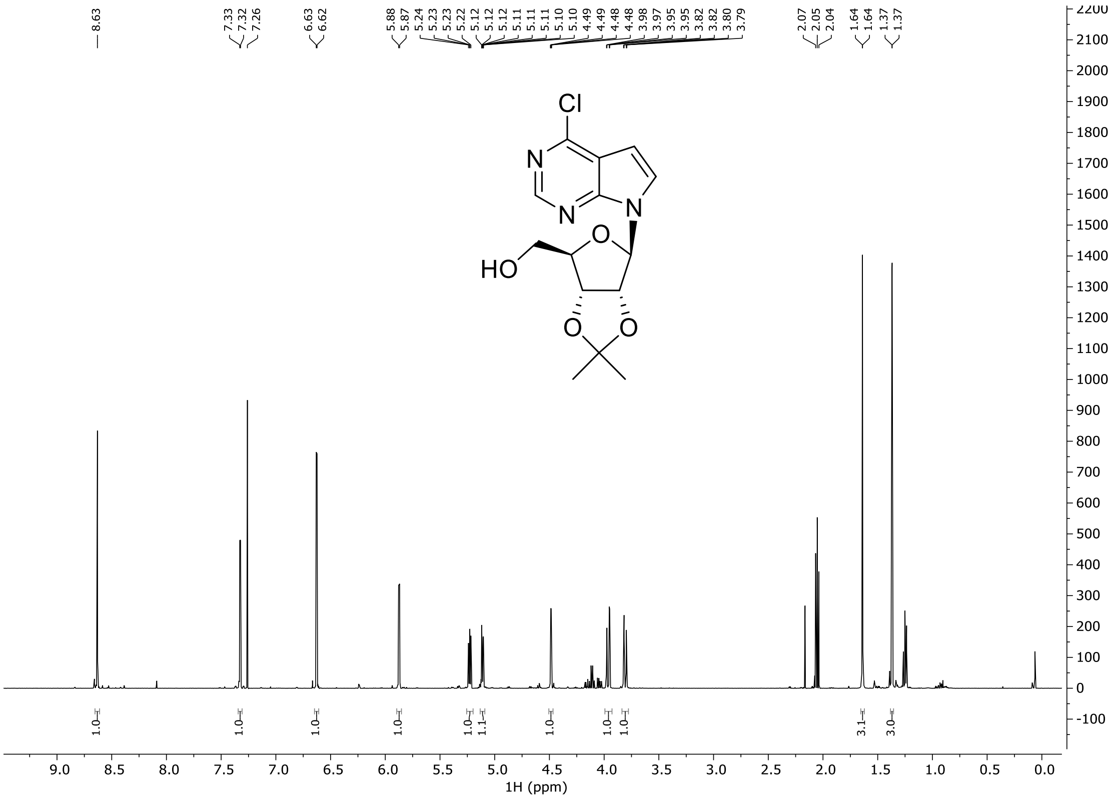

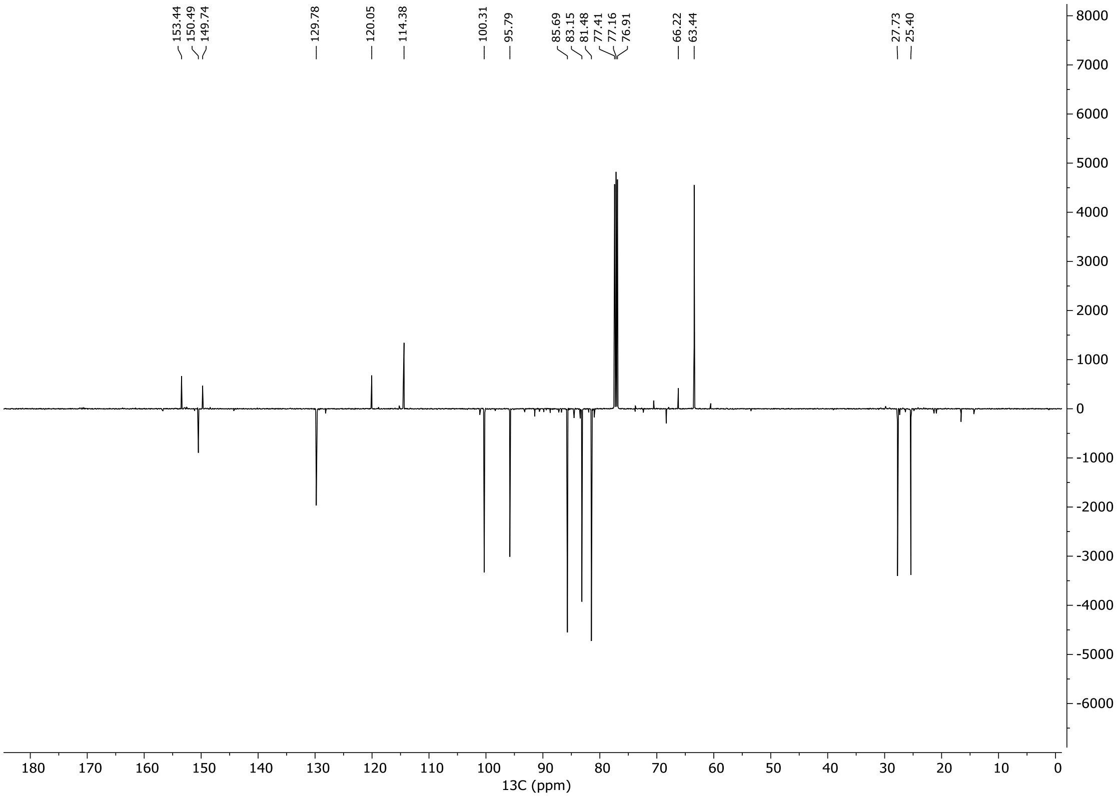

NMR spectra of compound **3**

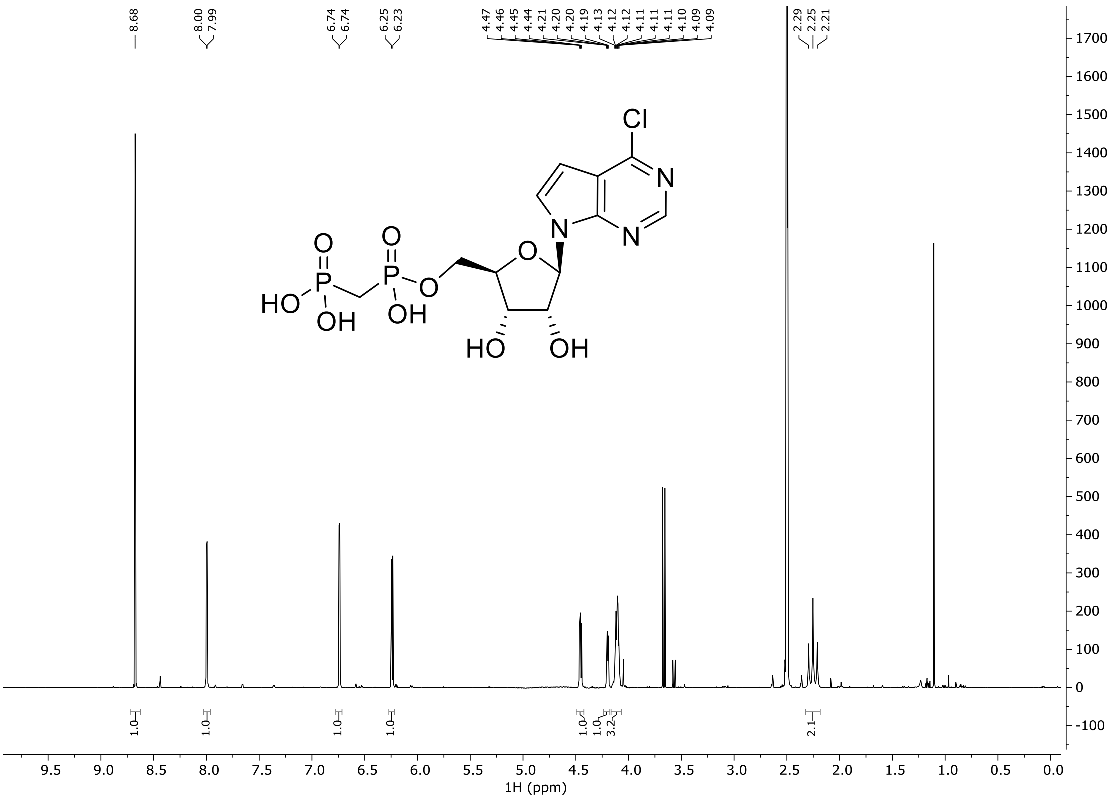

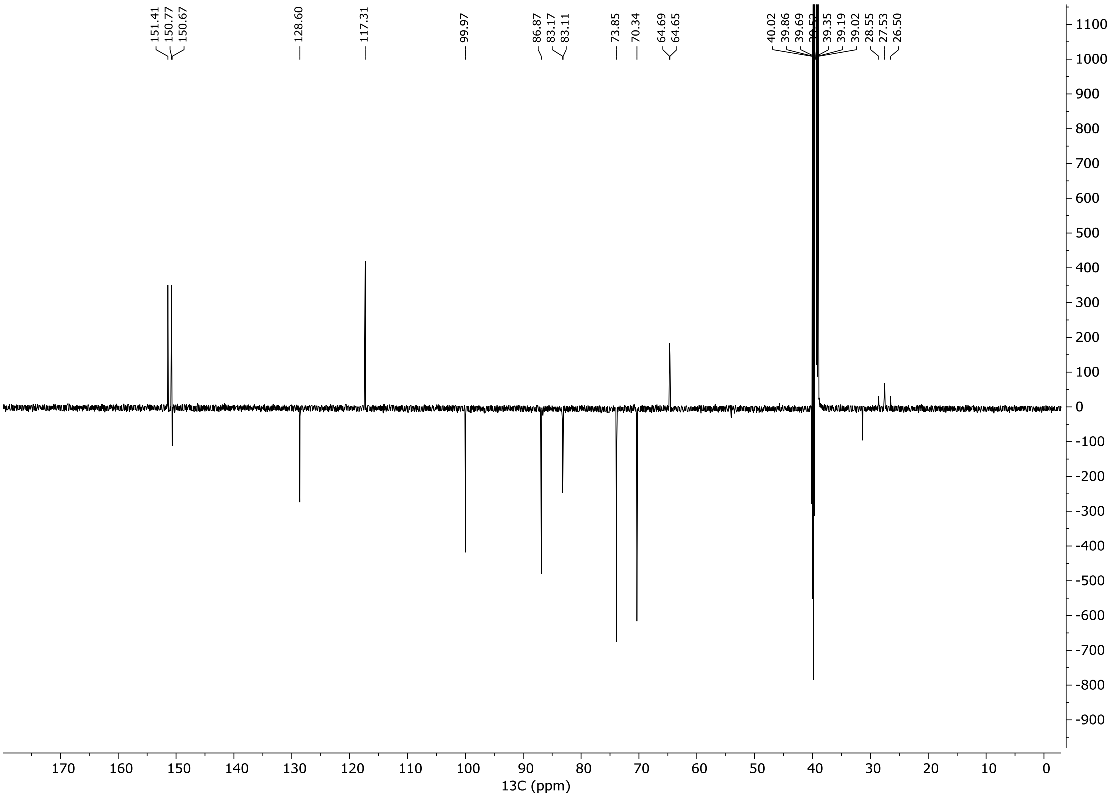

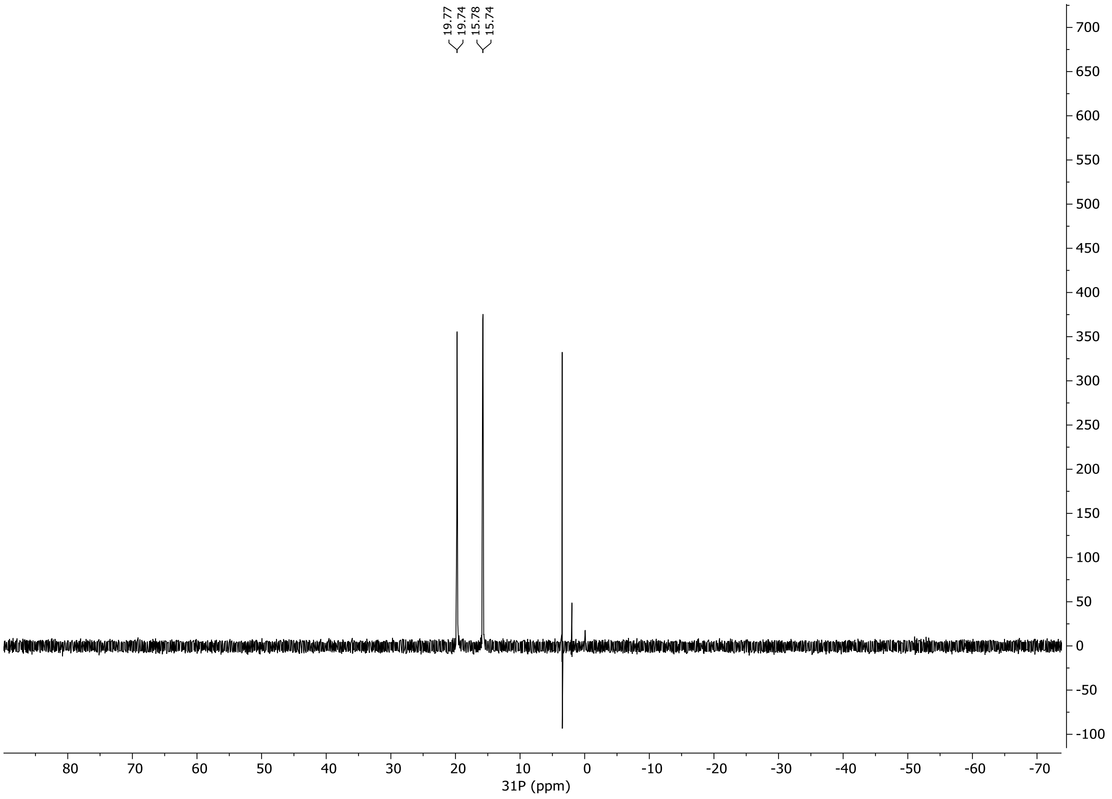

NMR spectra of compound **4A.1**

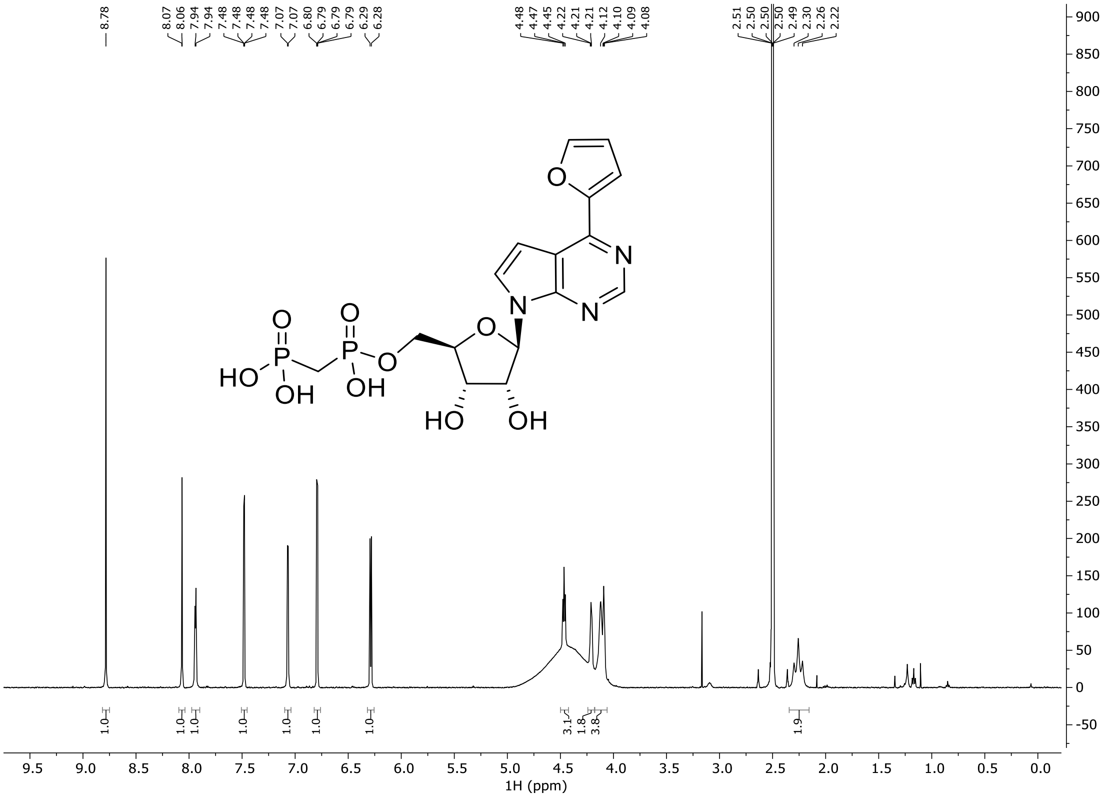

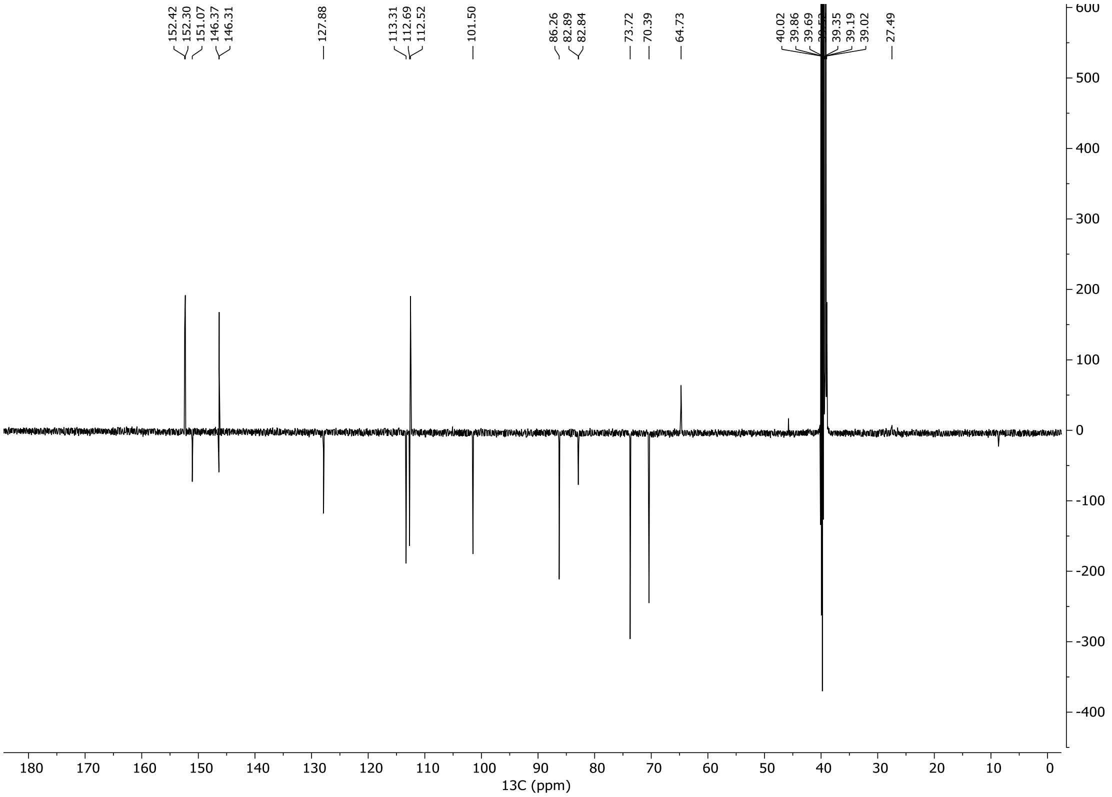
152.42
152.30
151.07
146.37
146.31
127.88
113.31
112.69
112.52
101.50
86.26
82.89
82.84
73.72
70.39
64.73
40.02
39.86
39.69
39.35
39.19
39.02
27.49
13C (ppm)

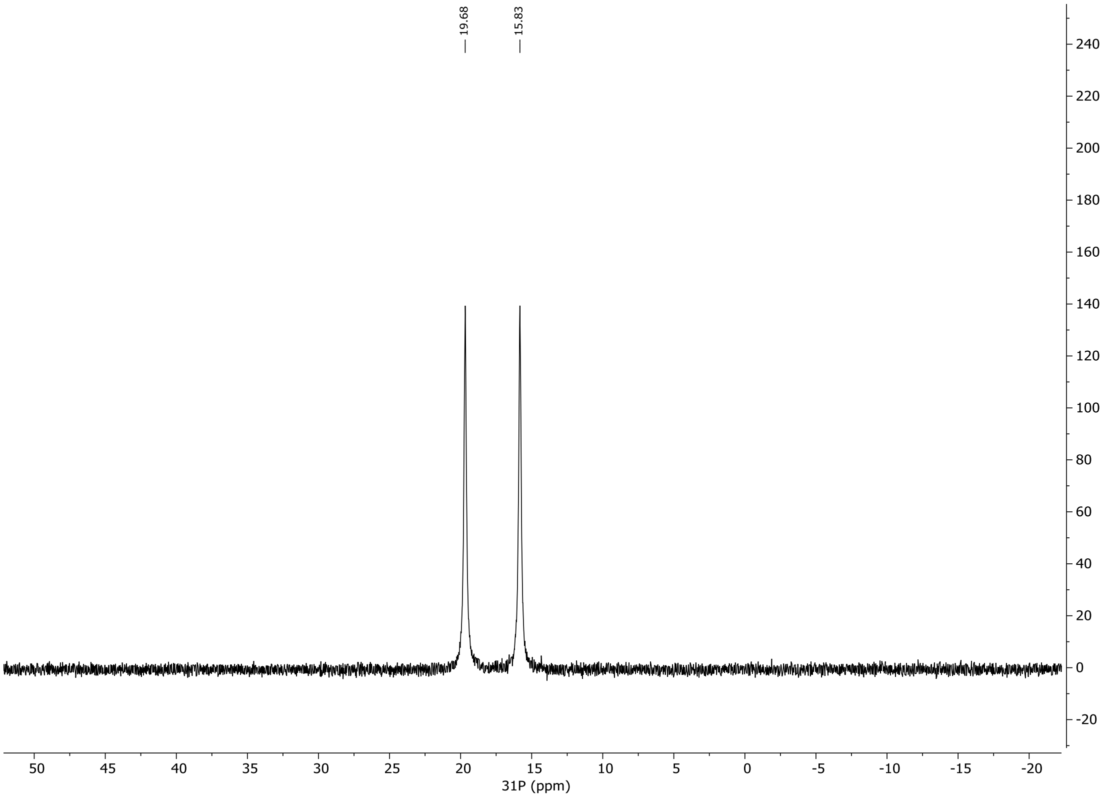
19.68
15.83
31P (ppm)

NMR spectra of compound **4A.2**

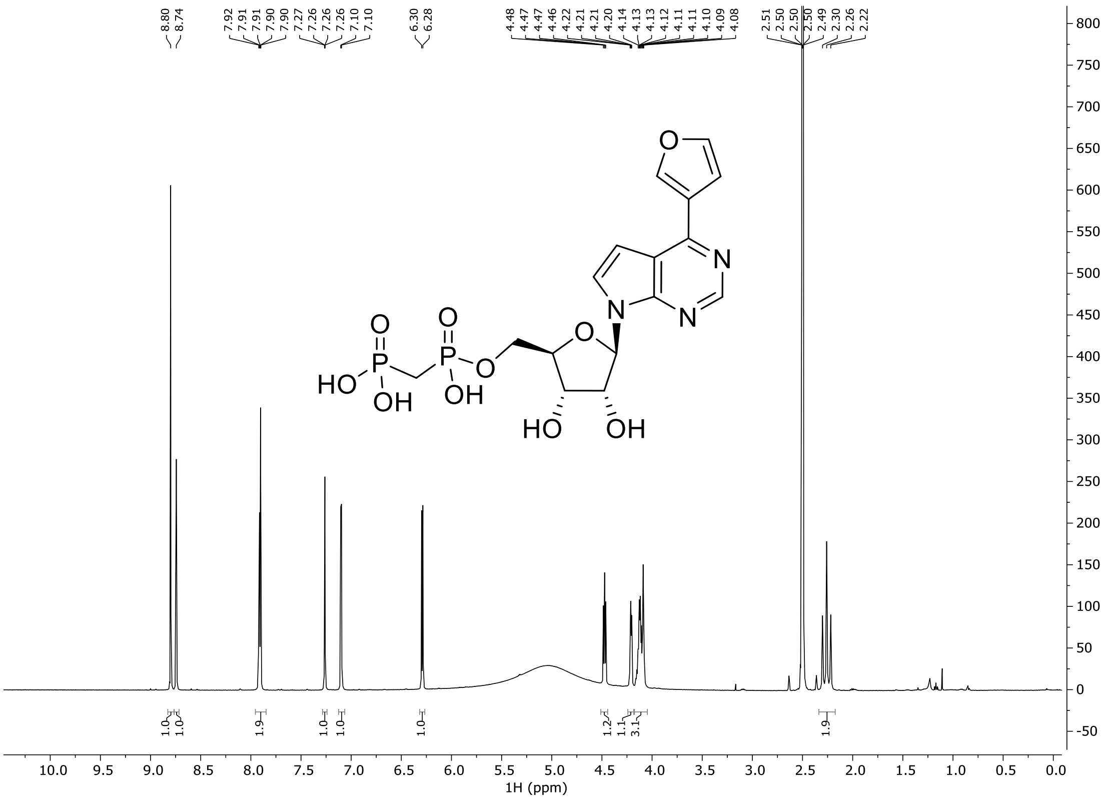

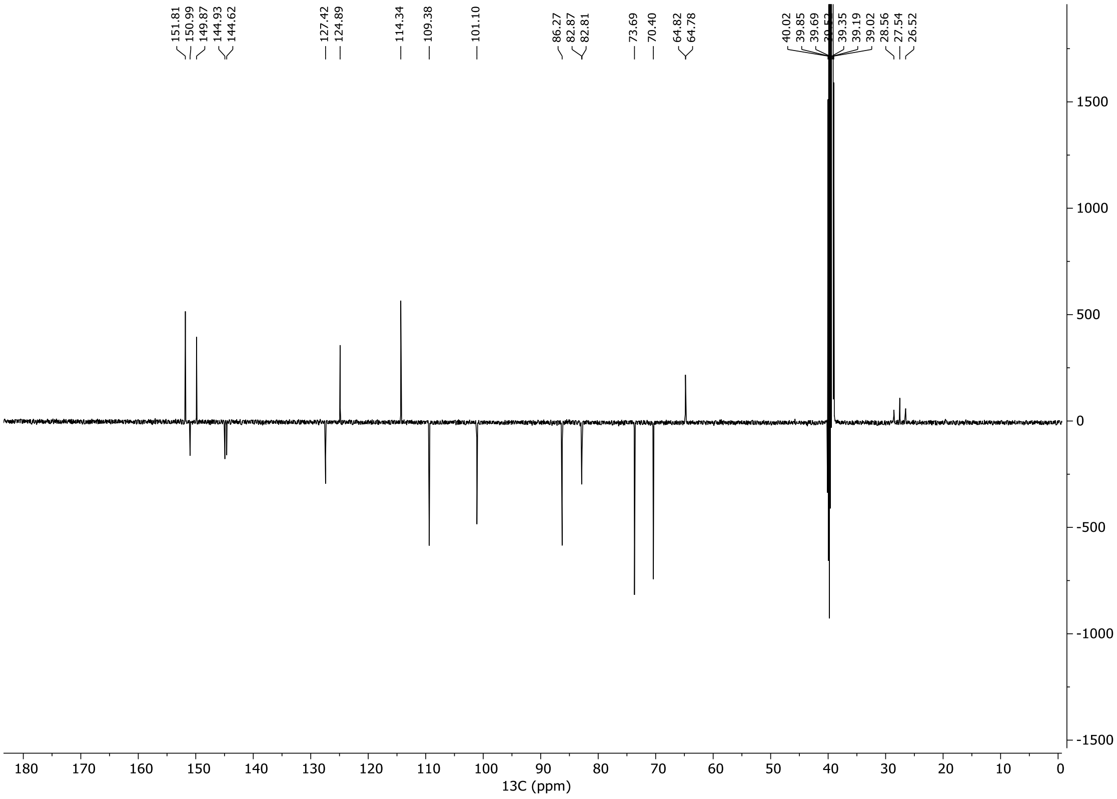

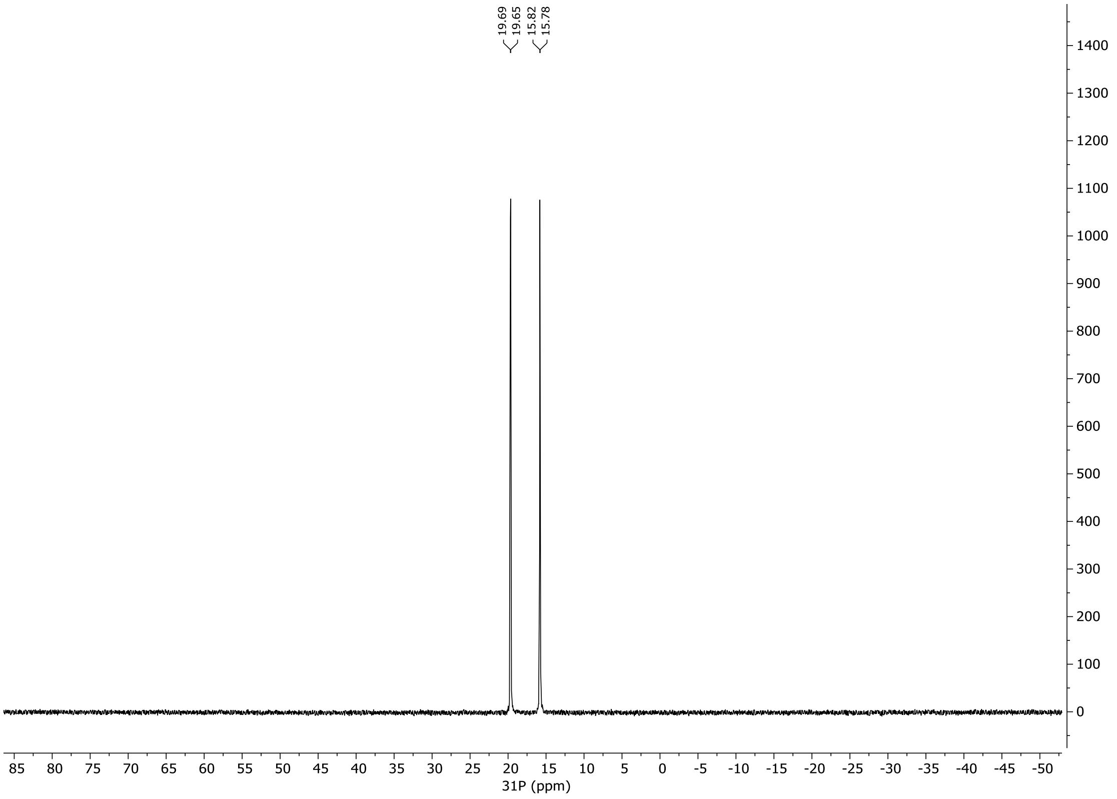

NMR spectra of compound **4A.3**

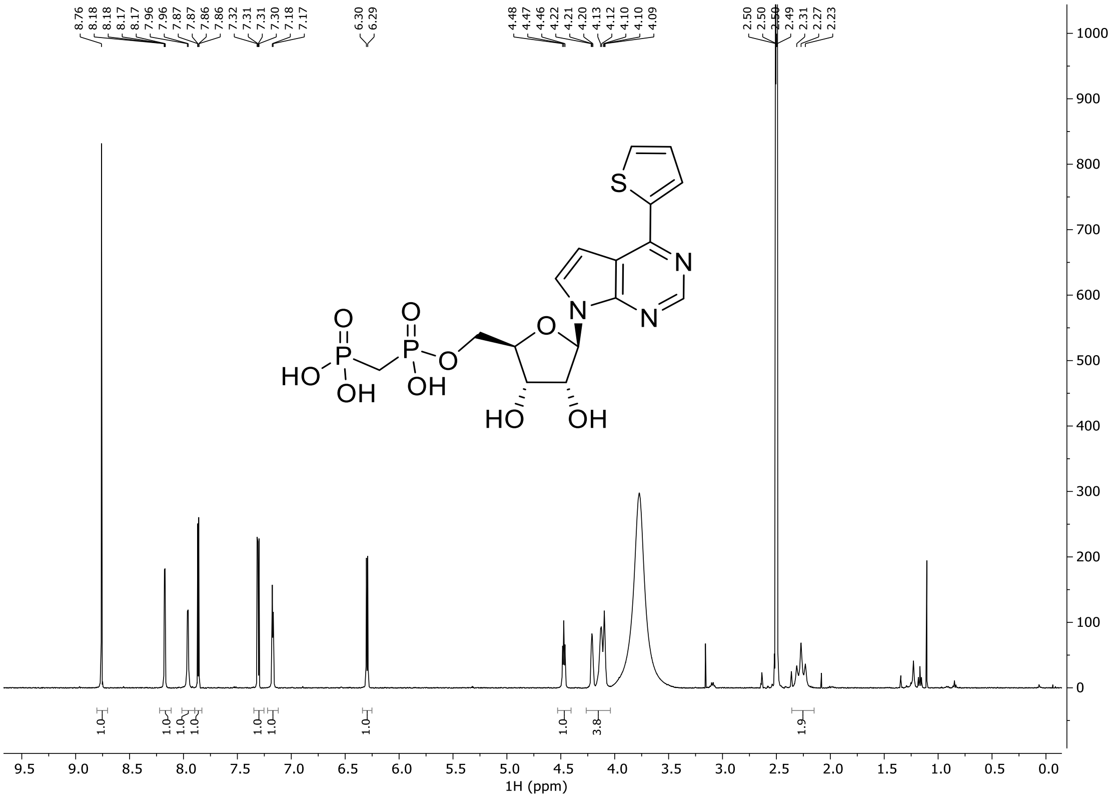

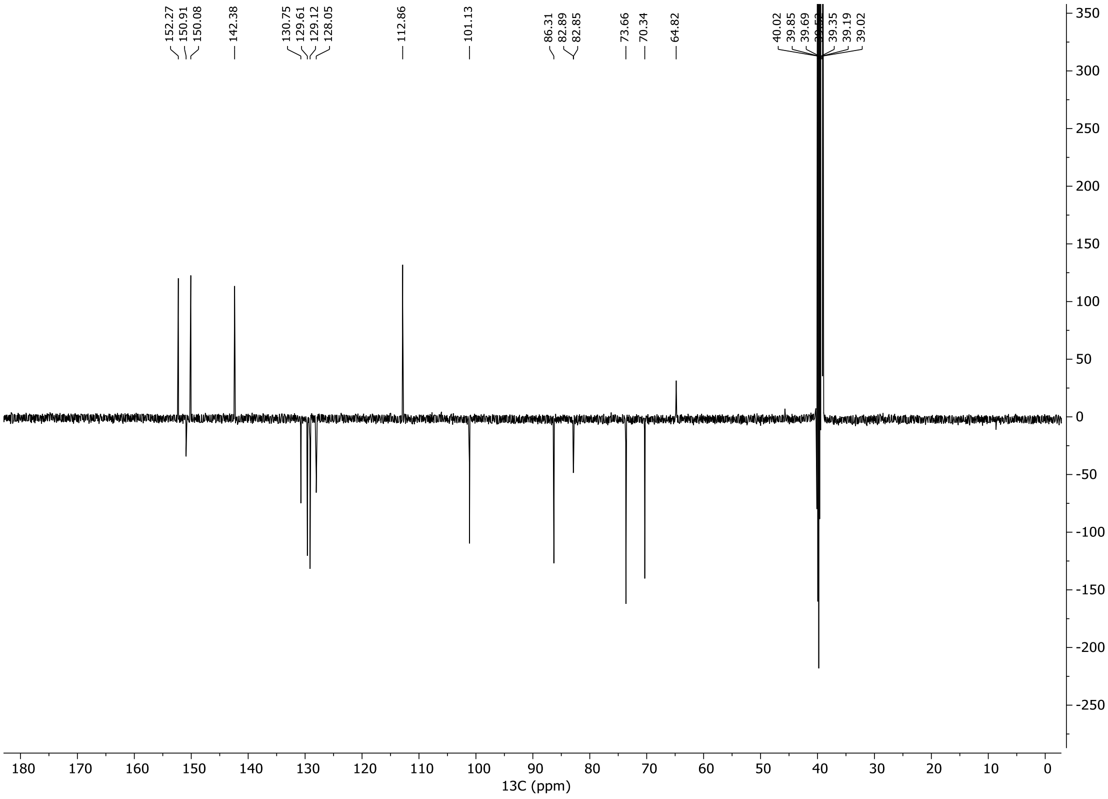
152.27
150.91
150.08
142.38
130.75
129.61
129.12
128.05
112.86
101.13
86.31
82.89
82.85
73.66
70.34
64.82
40.02
39.85
39.69
39.35
39.19
39.02
13C (ppm)

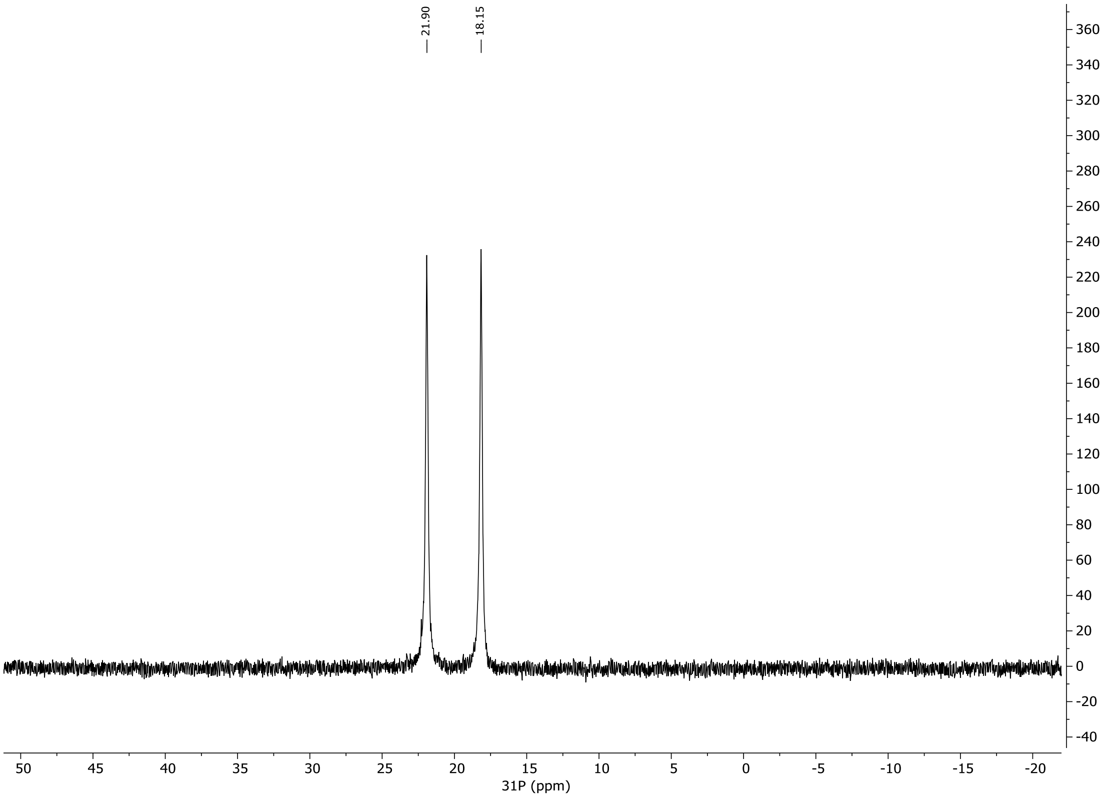
21.90
18.15
31P (ppm)

NMR spectra of compound **4A.4**

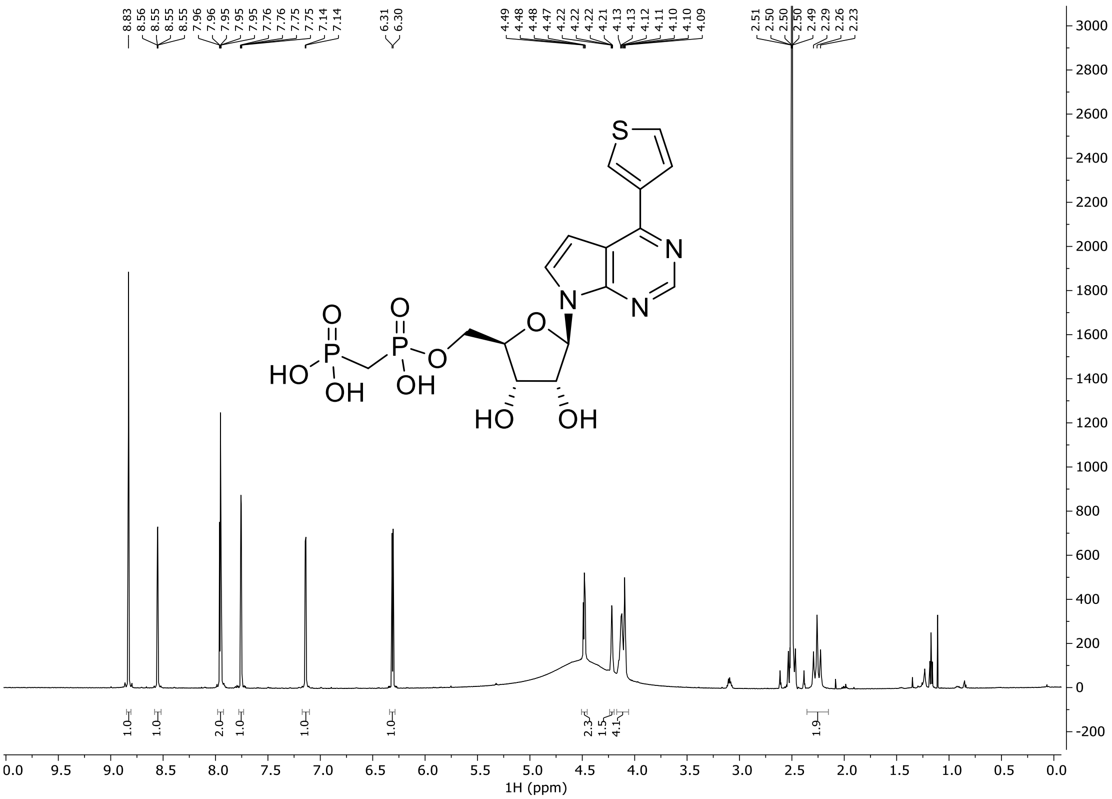

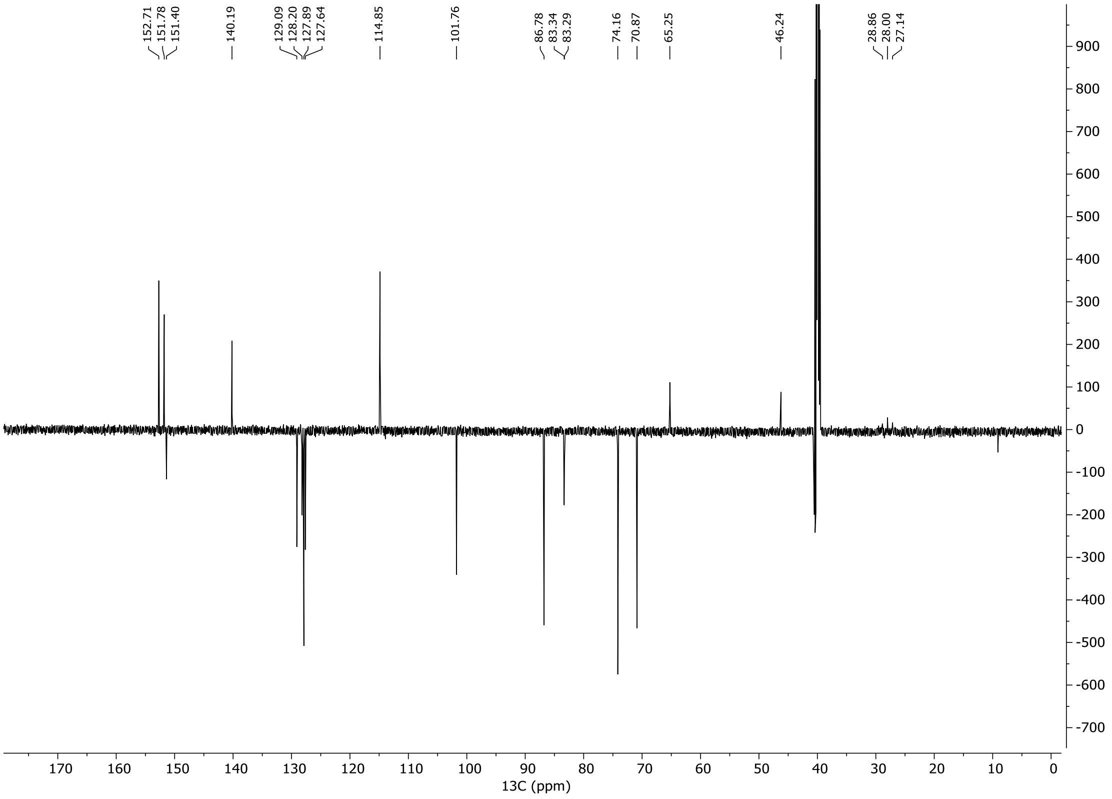

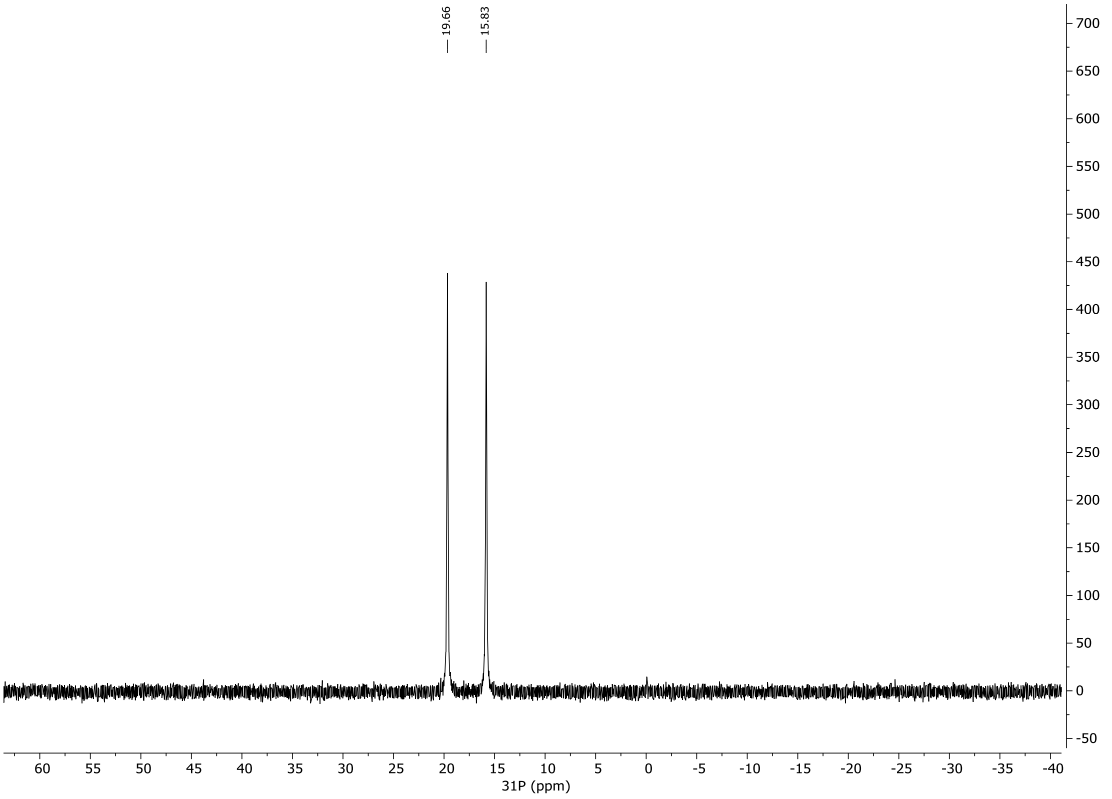

NMR spectra of compound **4A.5**

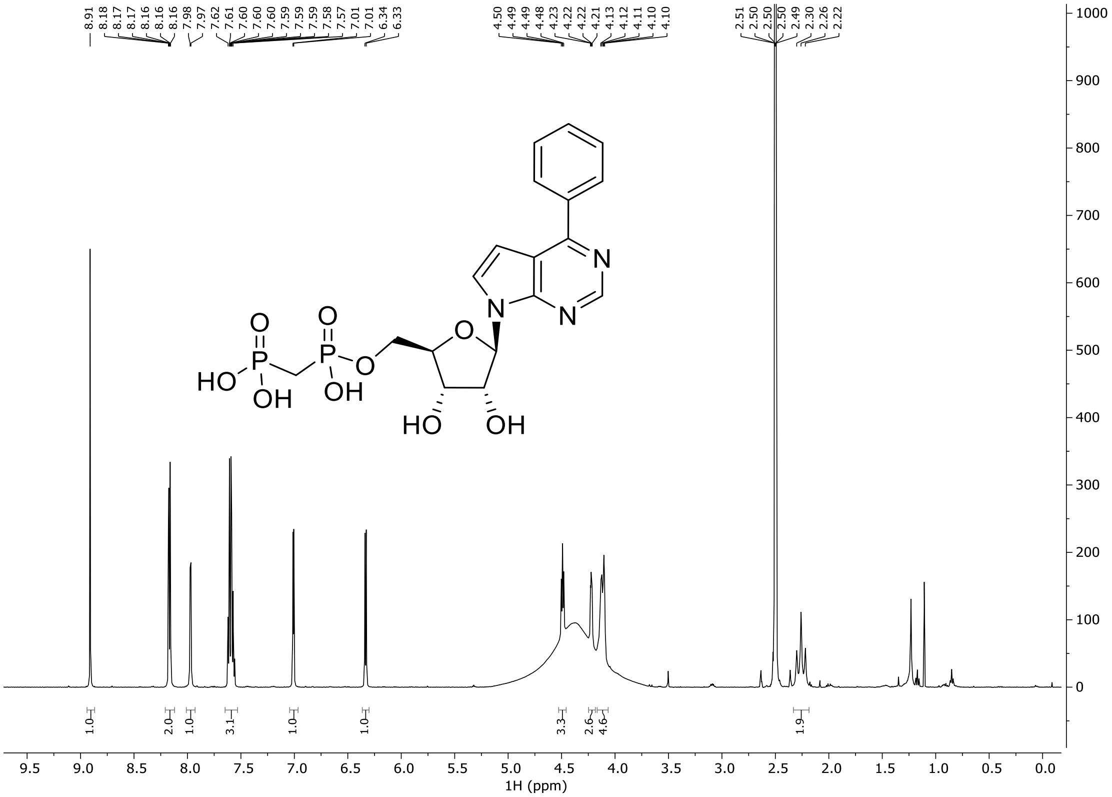

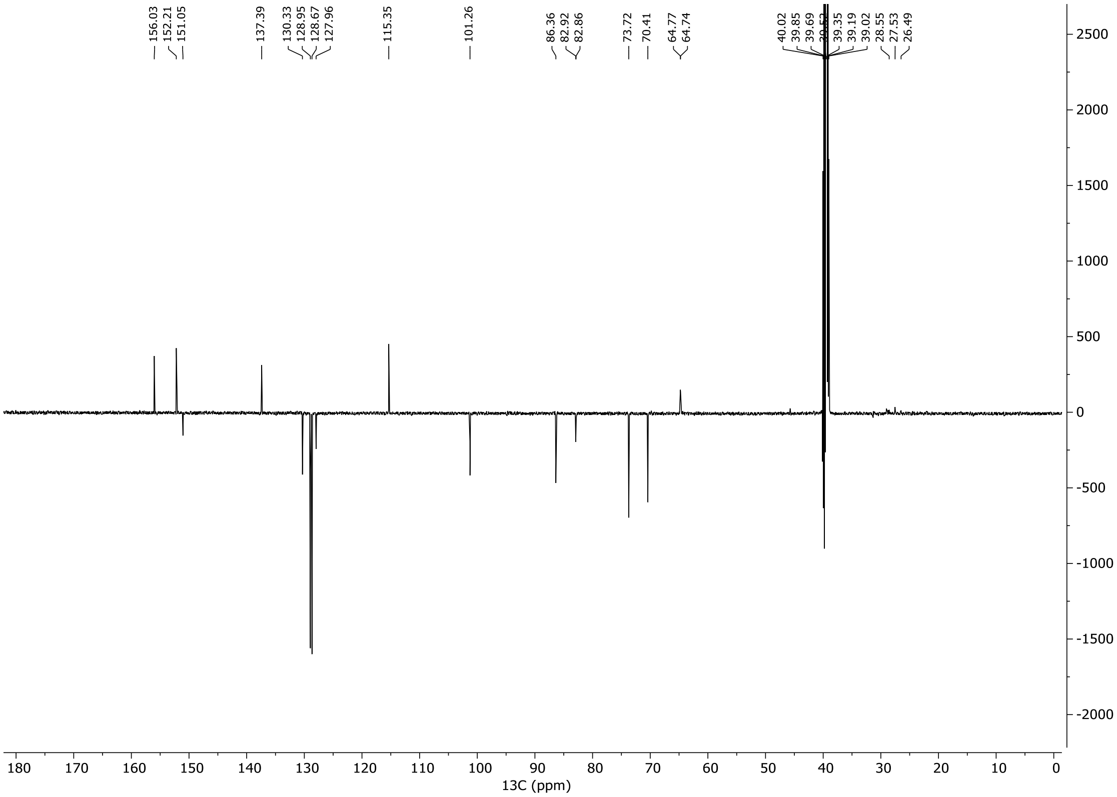
156.03
152.21
151.05
137.39
130.33
128.95
128.67
127.96
115.35
101.26
86.36
82.92
82.86
73.72
70.41
64.77
64.74
40.02
39.85
39.69
39.35
39.19
39.02
28.55
27.53
26.49
13C (ppm)

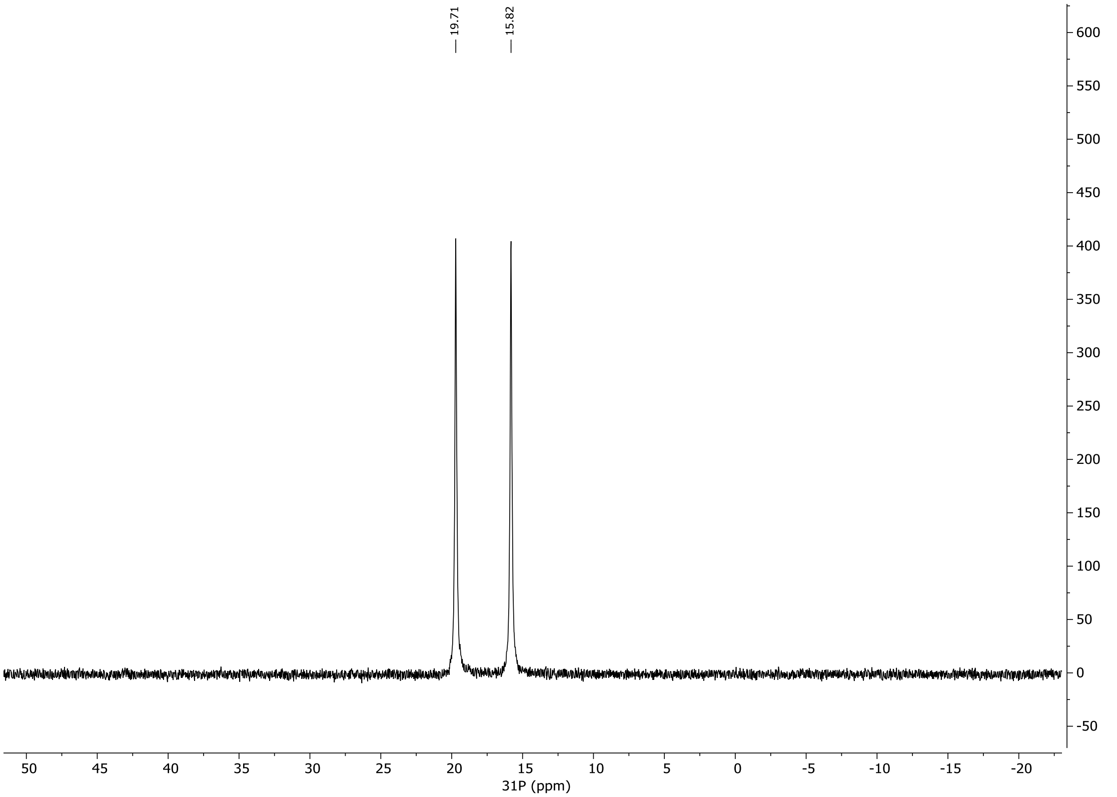
19.71
15.82
31P (ppm)

NMR spectra of compound **4A.6**

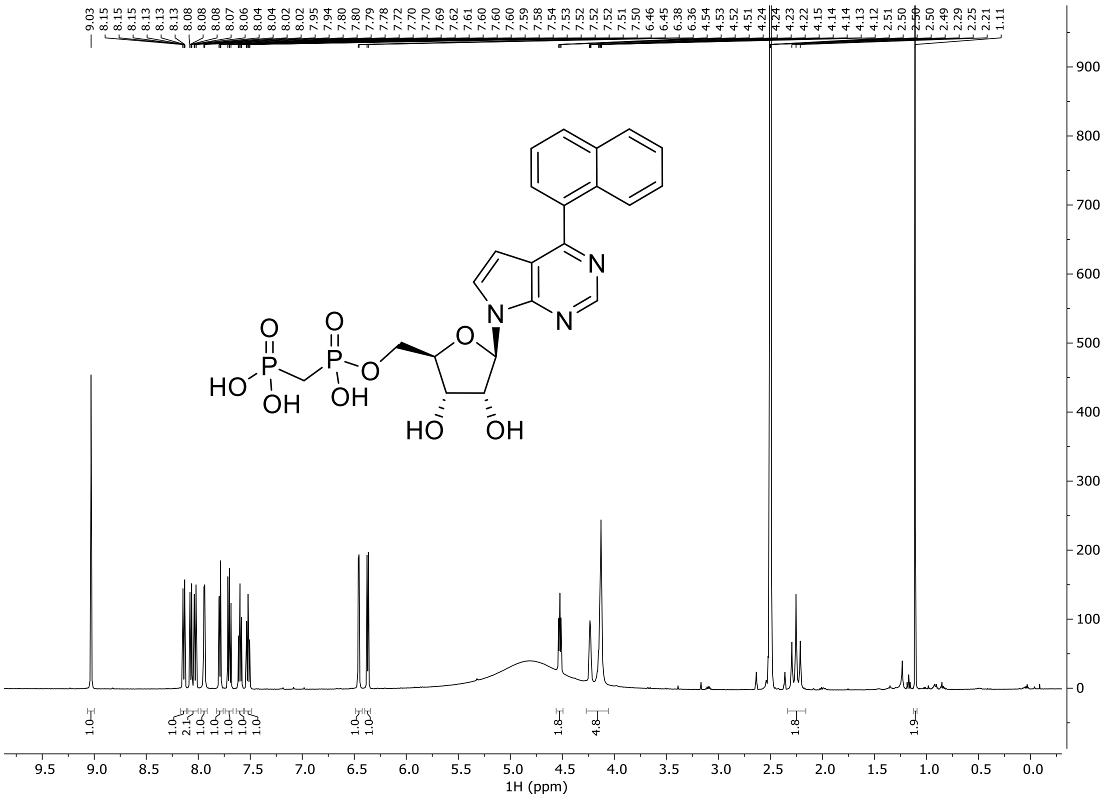

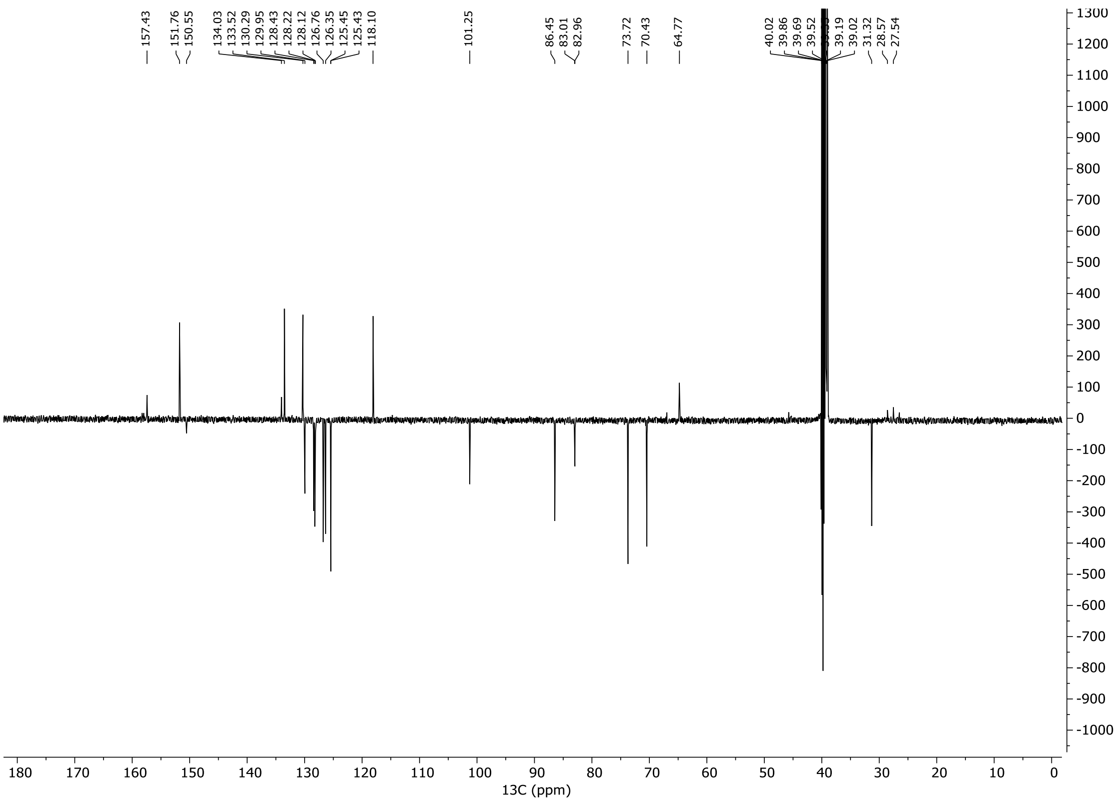

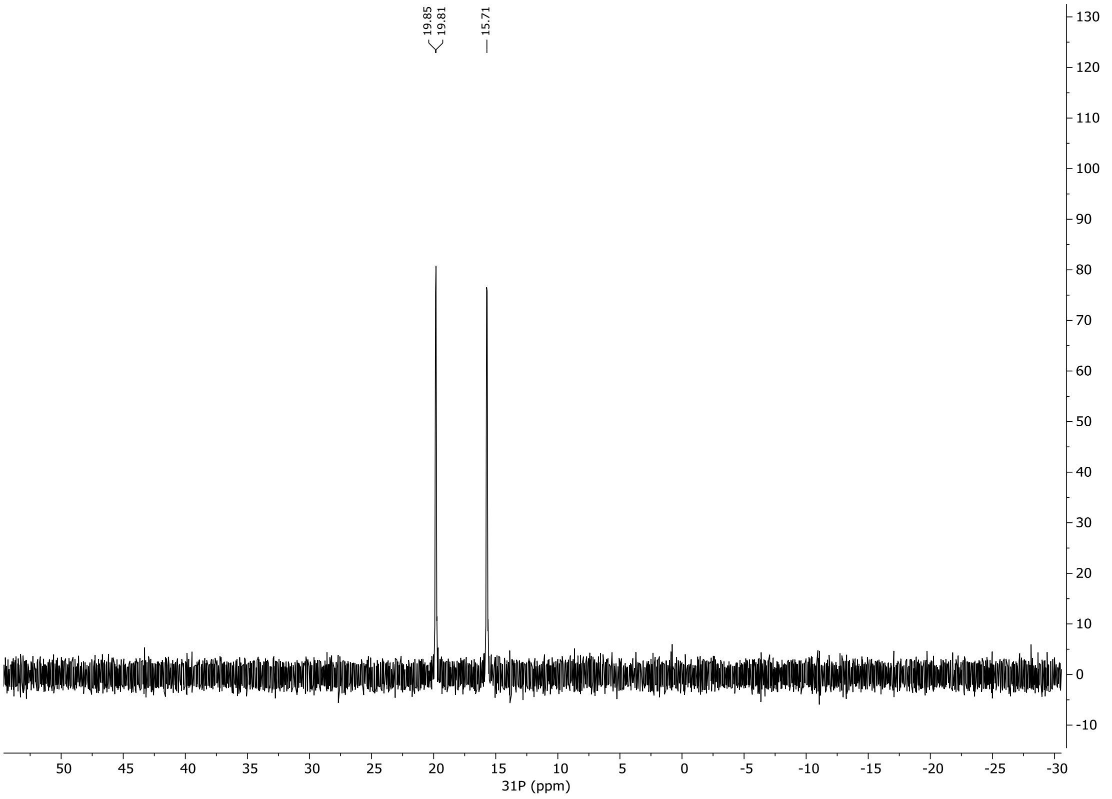

NMR spectra of compound **4A.7**

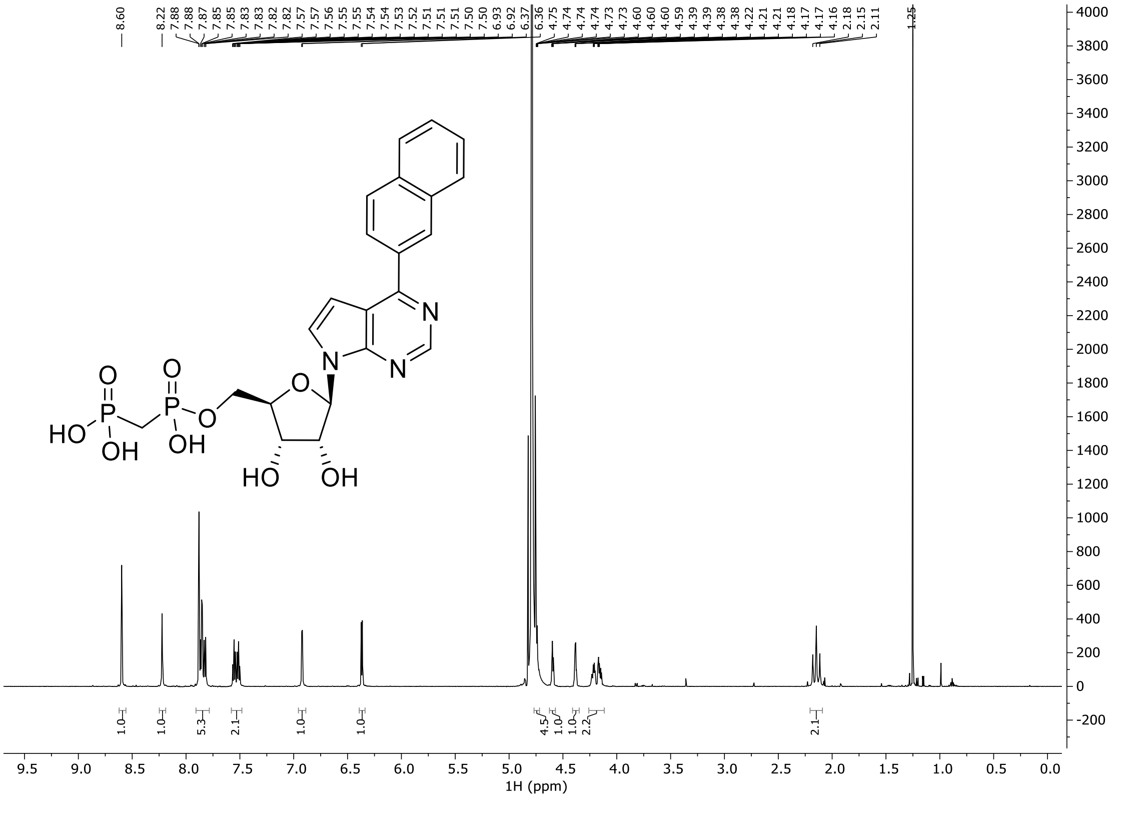

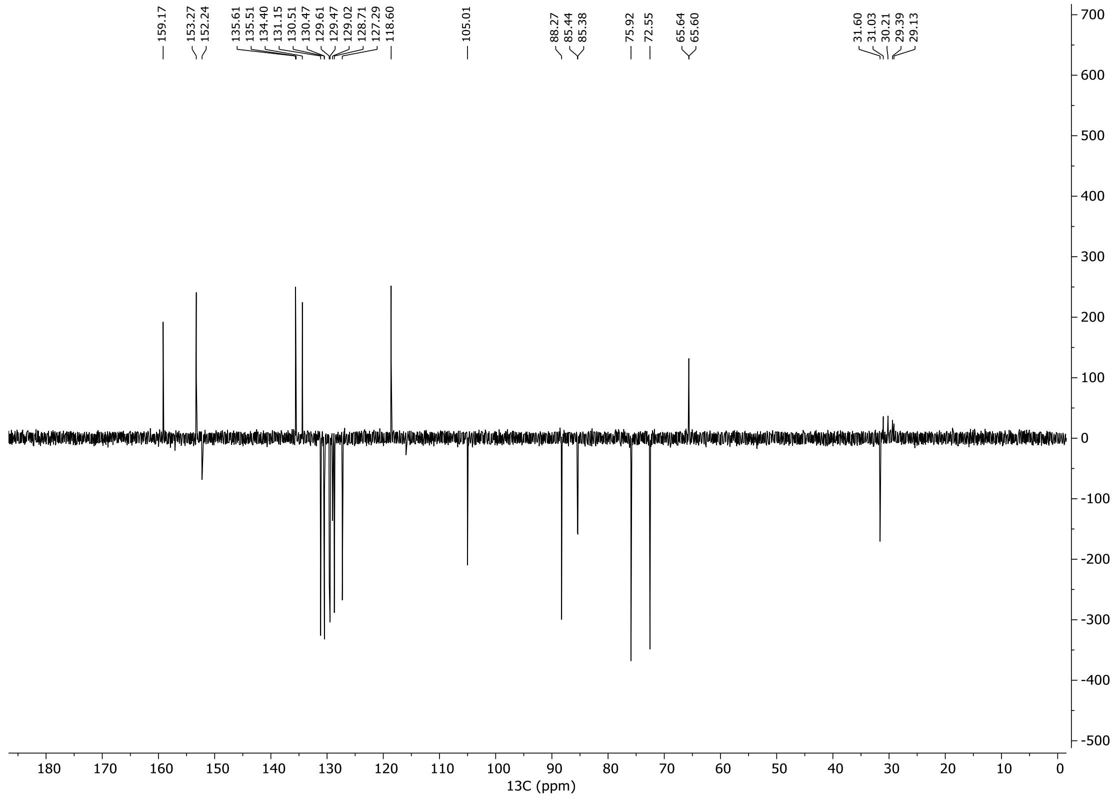
159.17
153.27
152.24
135.61
135.51
134.40
131.15
130.51
130.47
129.61
129.47
129.02
128.71
127.29
118.60
105.01
88.27
85.44
85.38
75.92
72.55
65.64
65.60
31.60
31.03
30.21
29.39
29.13
13C (ppm)

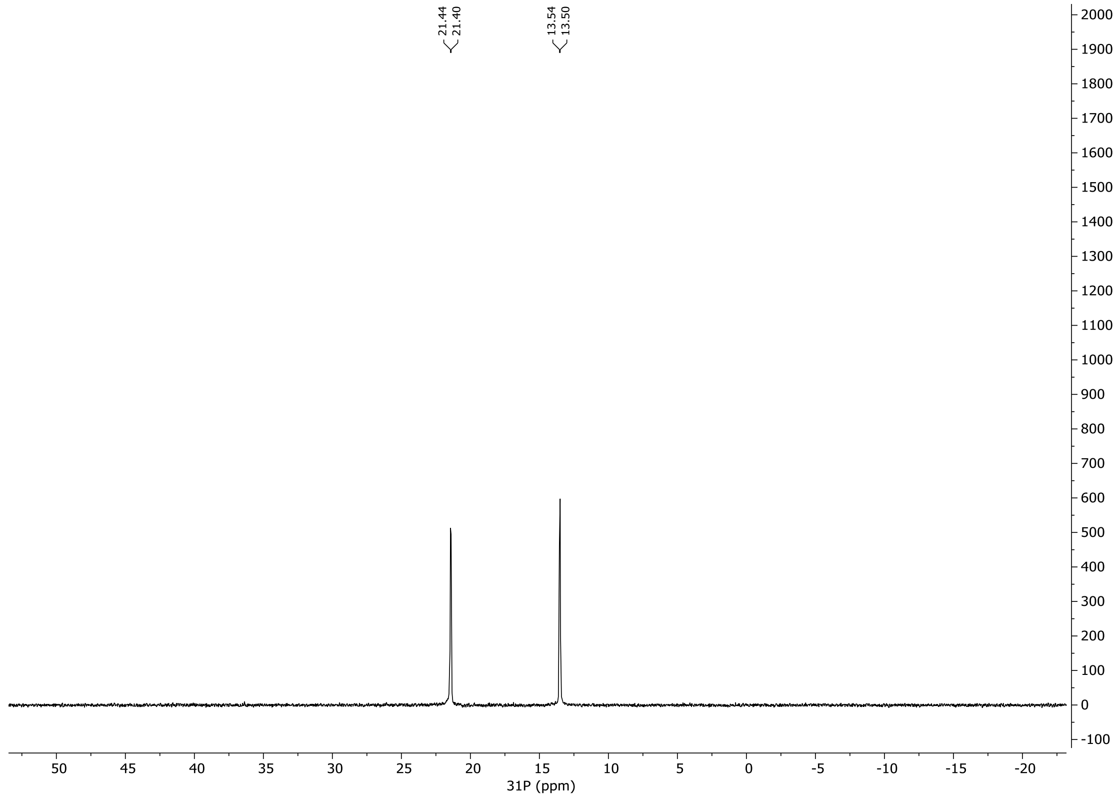
21.44
21.40
13.54
13.50
31P (ppm)

NMR spectra of compound **4A.8**

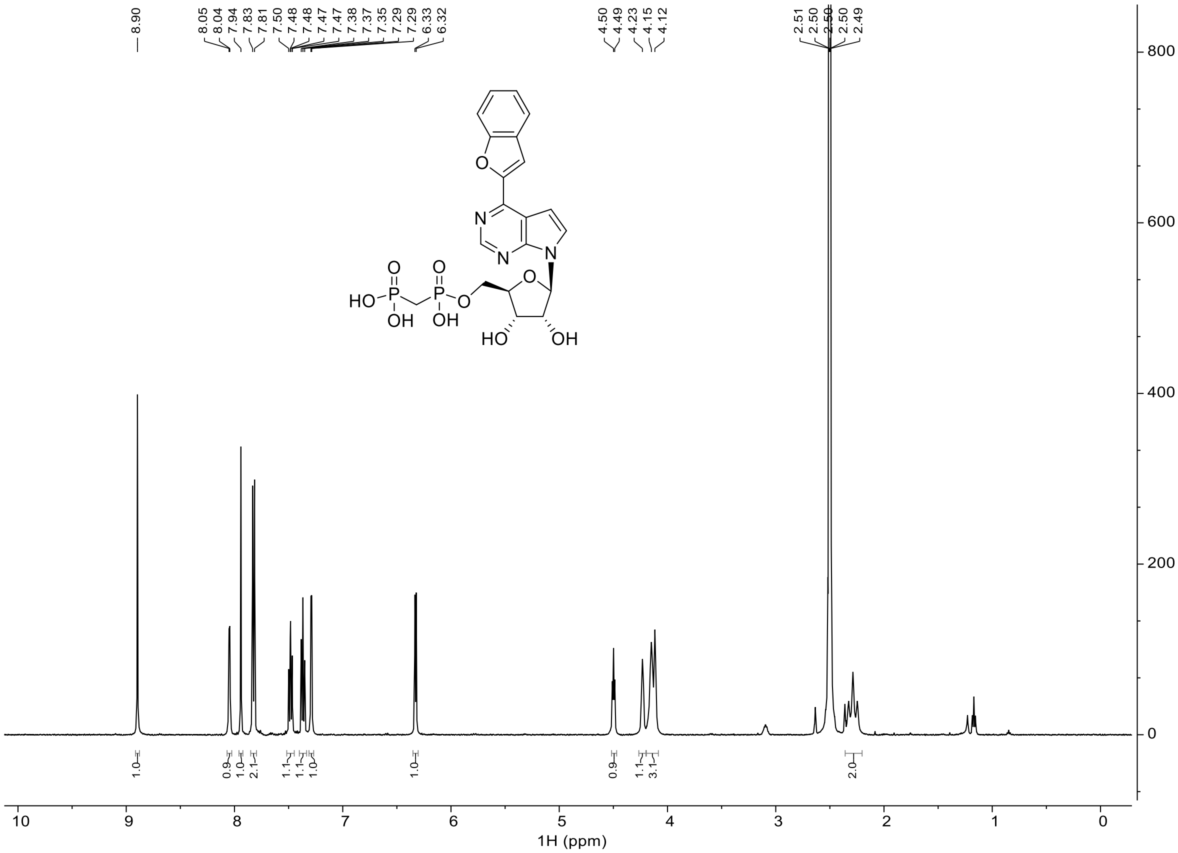

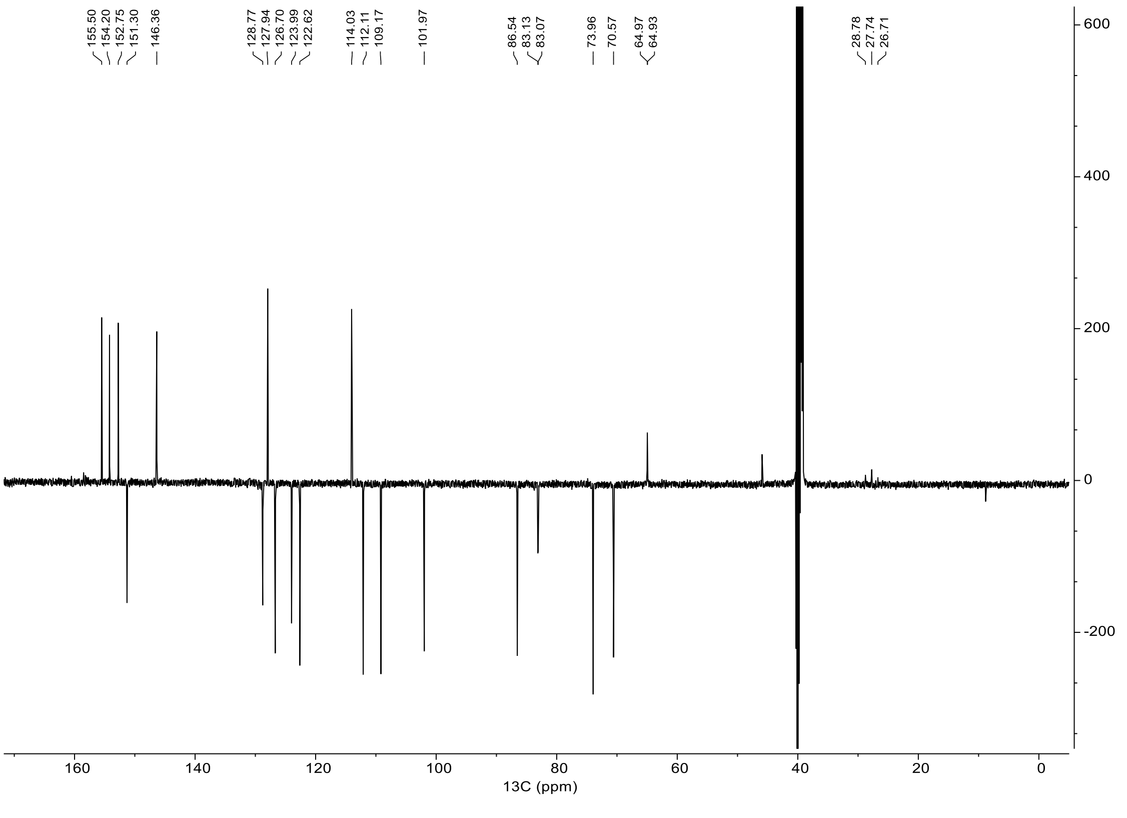

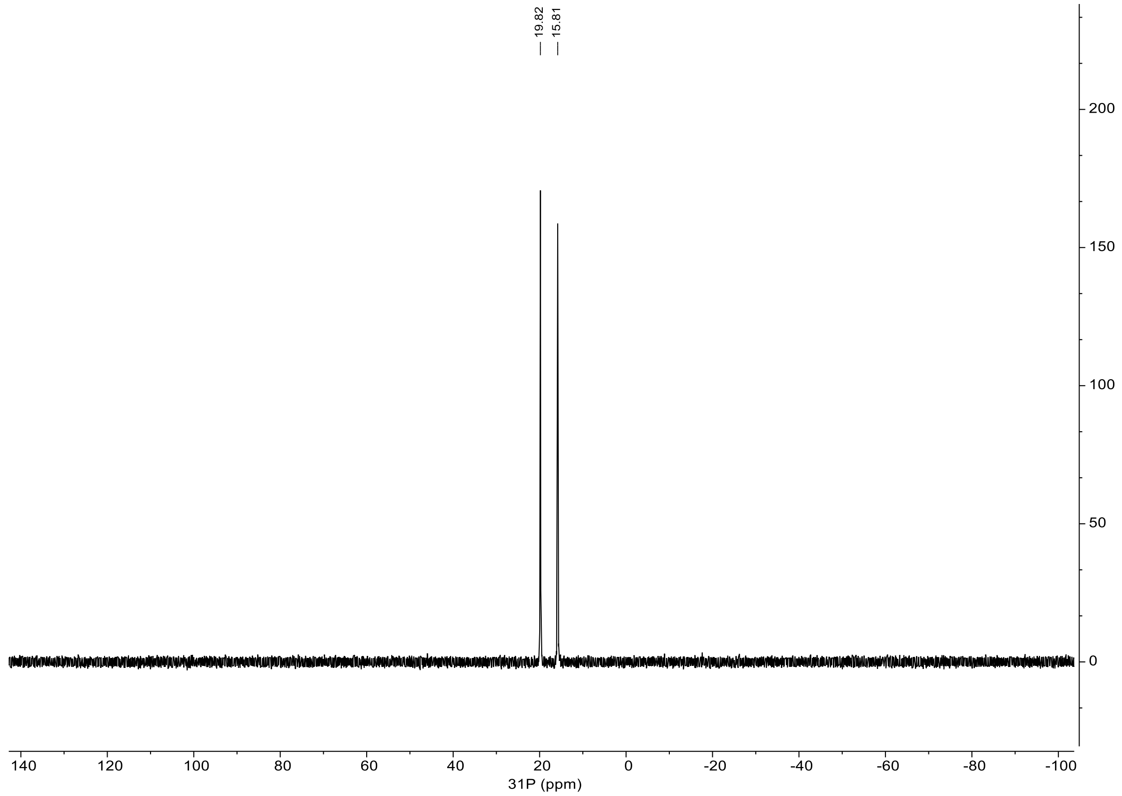

NMR spectra of compound **4A.9**

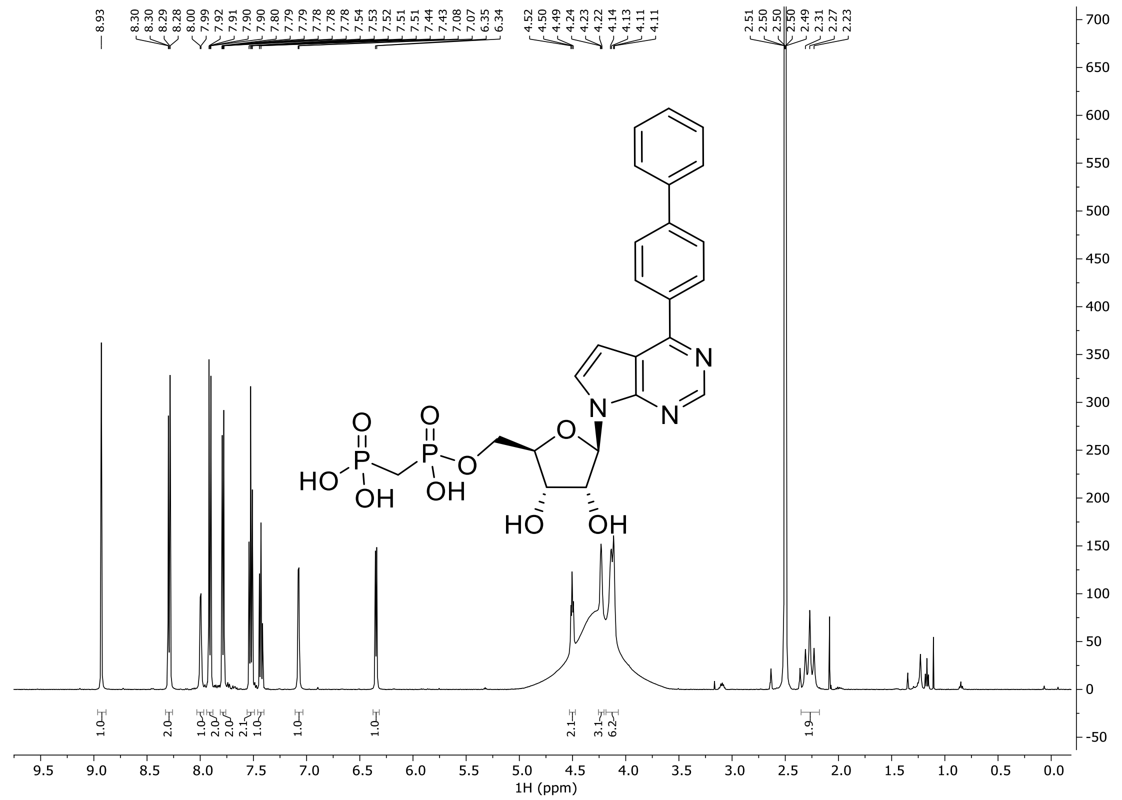

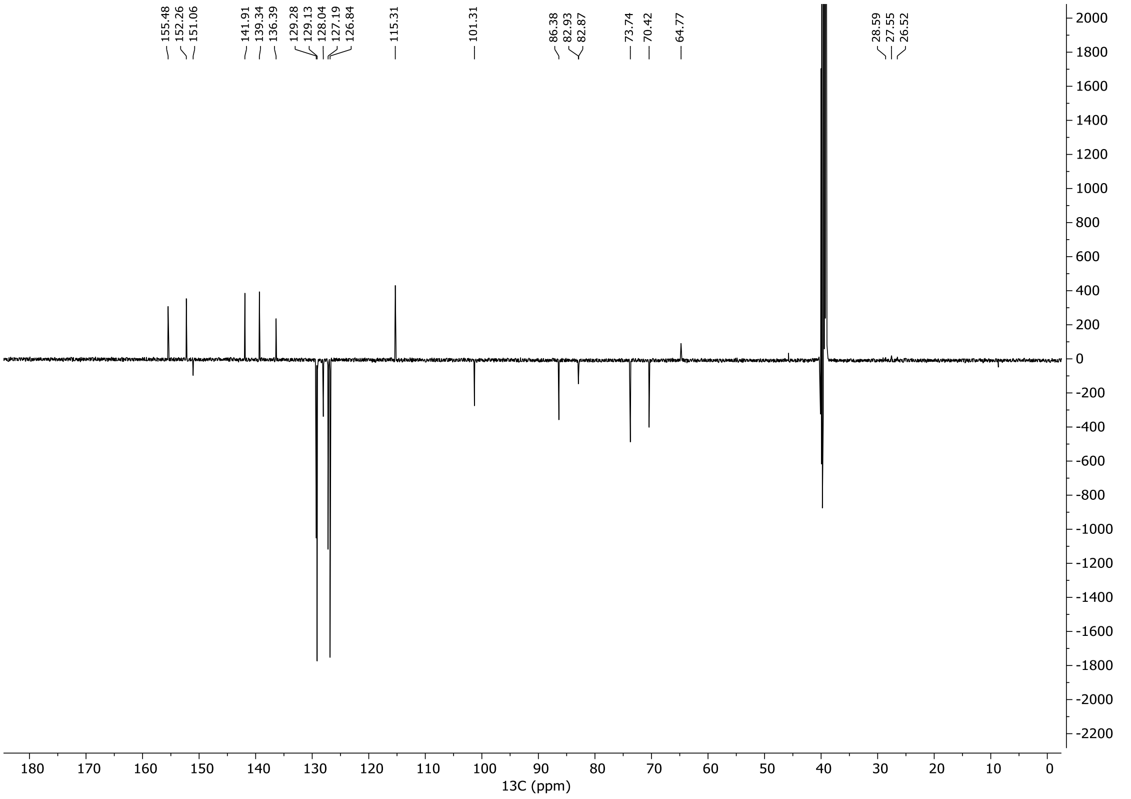
155.48
152.26
151.06
141.91
139.34
136.39
129.28
129.13
128.04
127.19
126.84
115.31
101.31
86.38
82.93
82.87
73.74
70.42
64.77
28.59
27.55
26.52
13C (ppm)

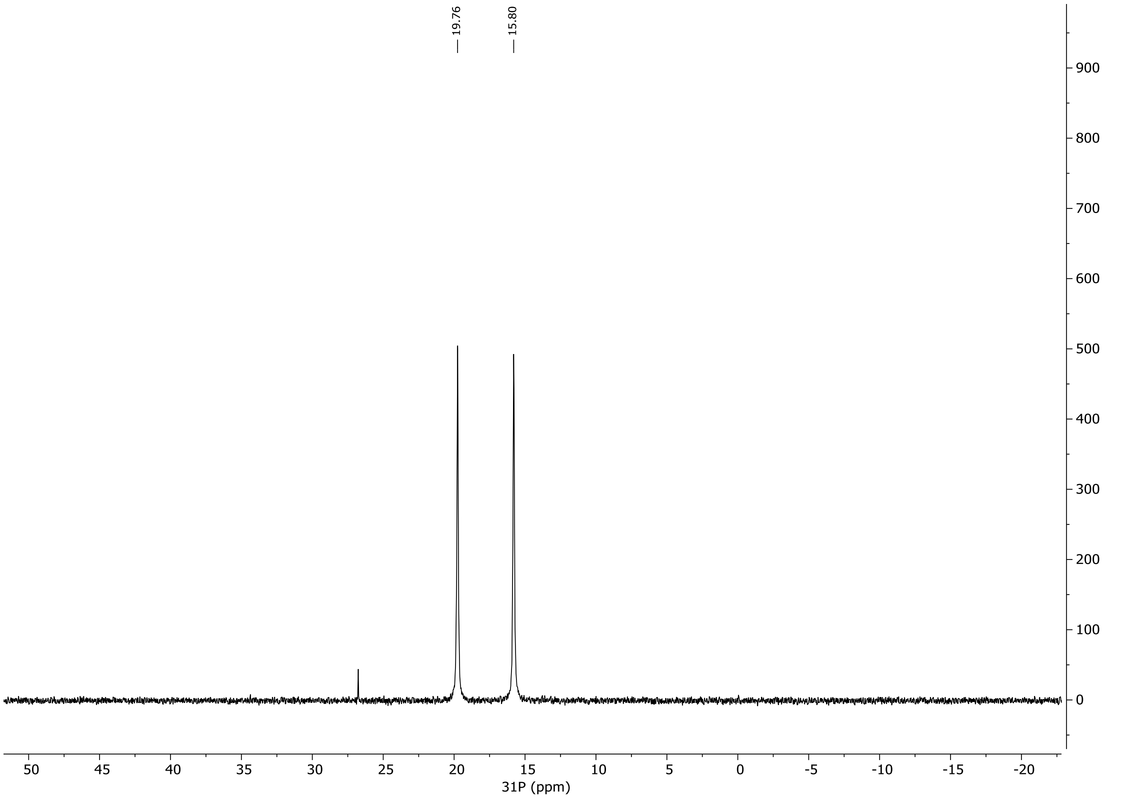
19.76
15.80
31P (ppm)

NMR spectra of compound **4A.10**

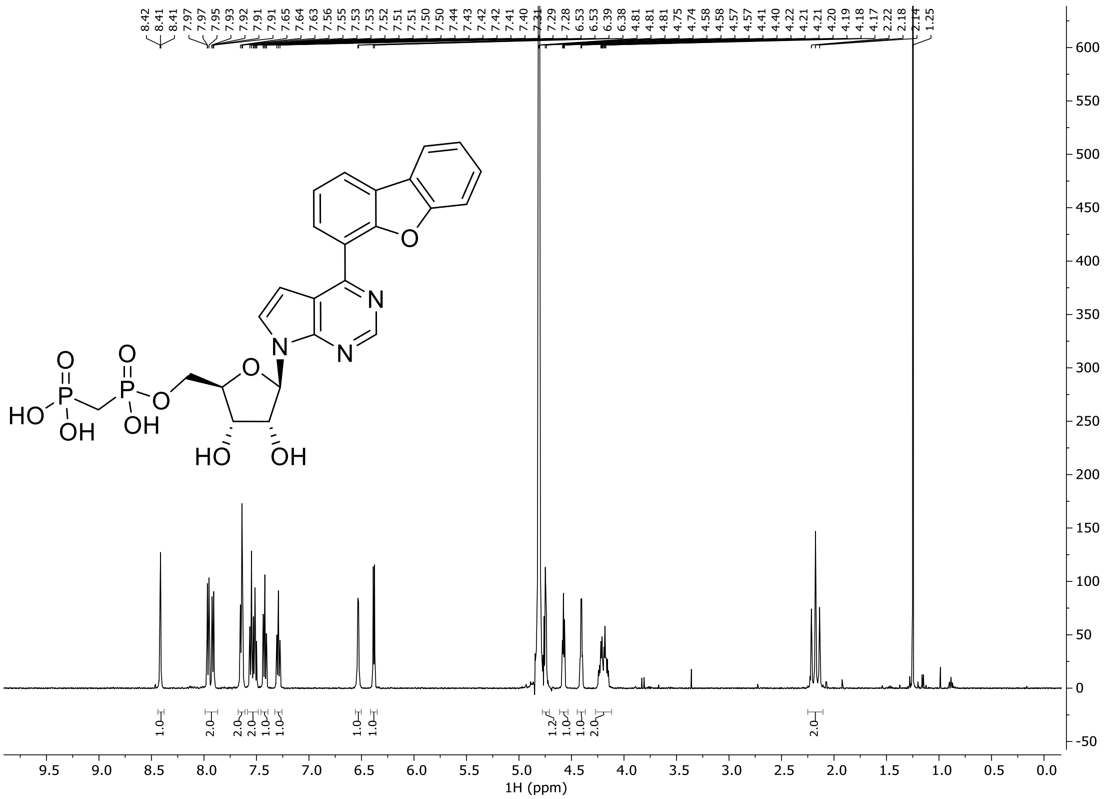

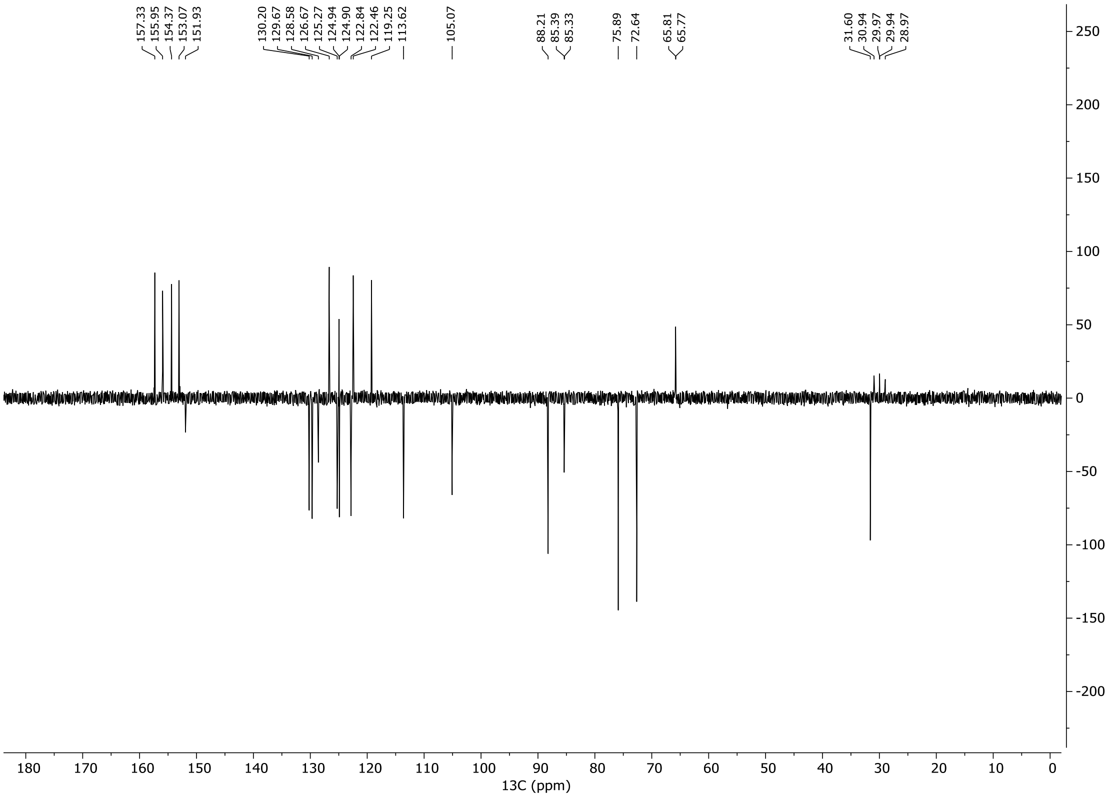

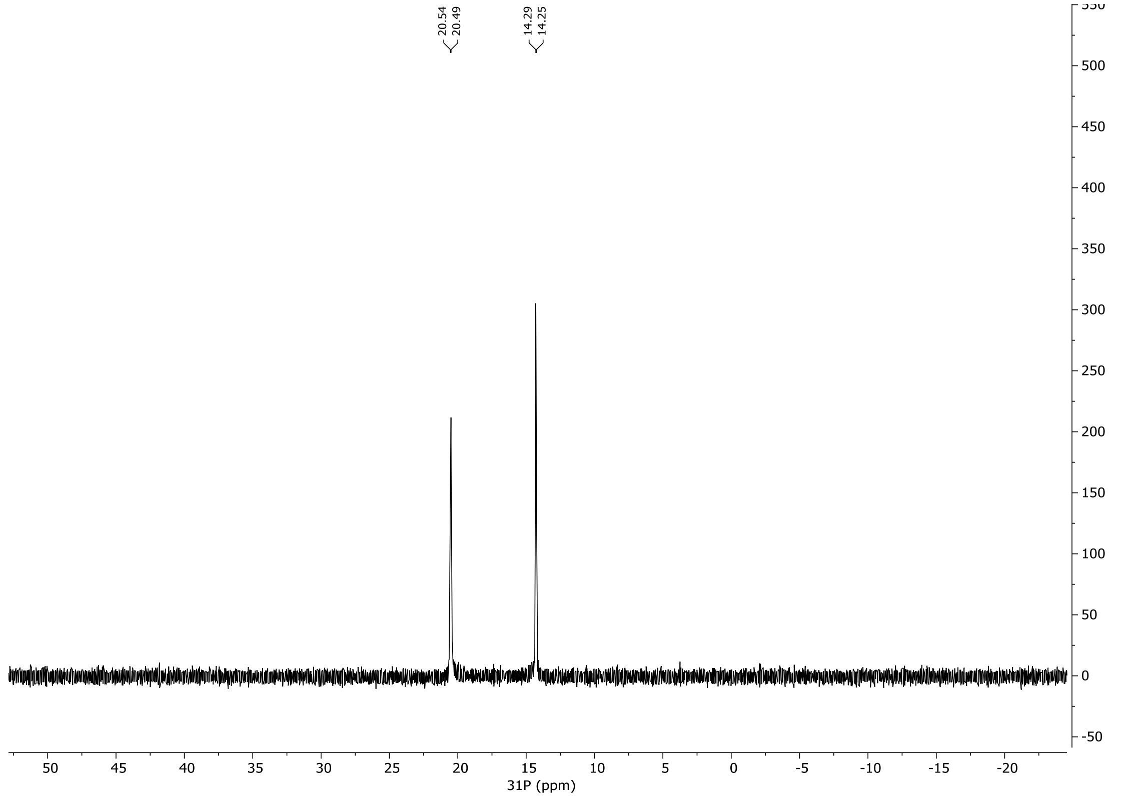

NMR spectra of compound **4A.11**

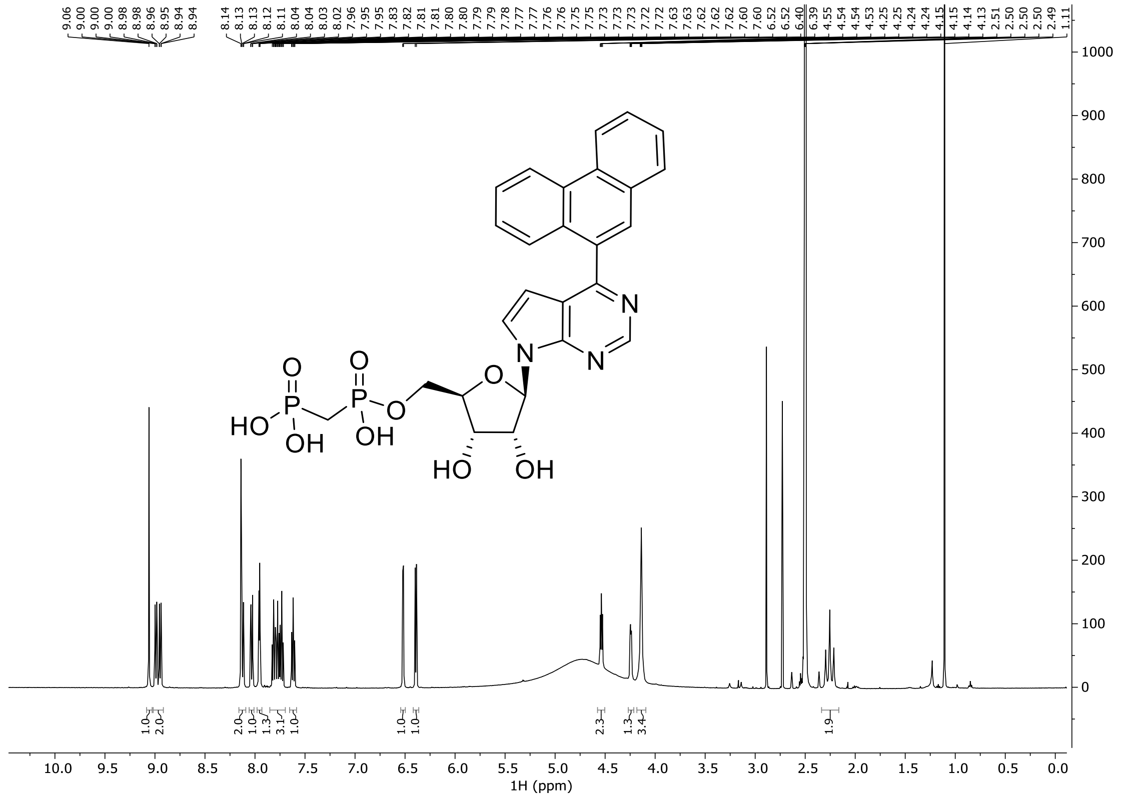

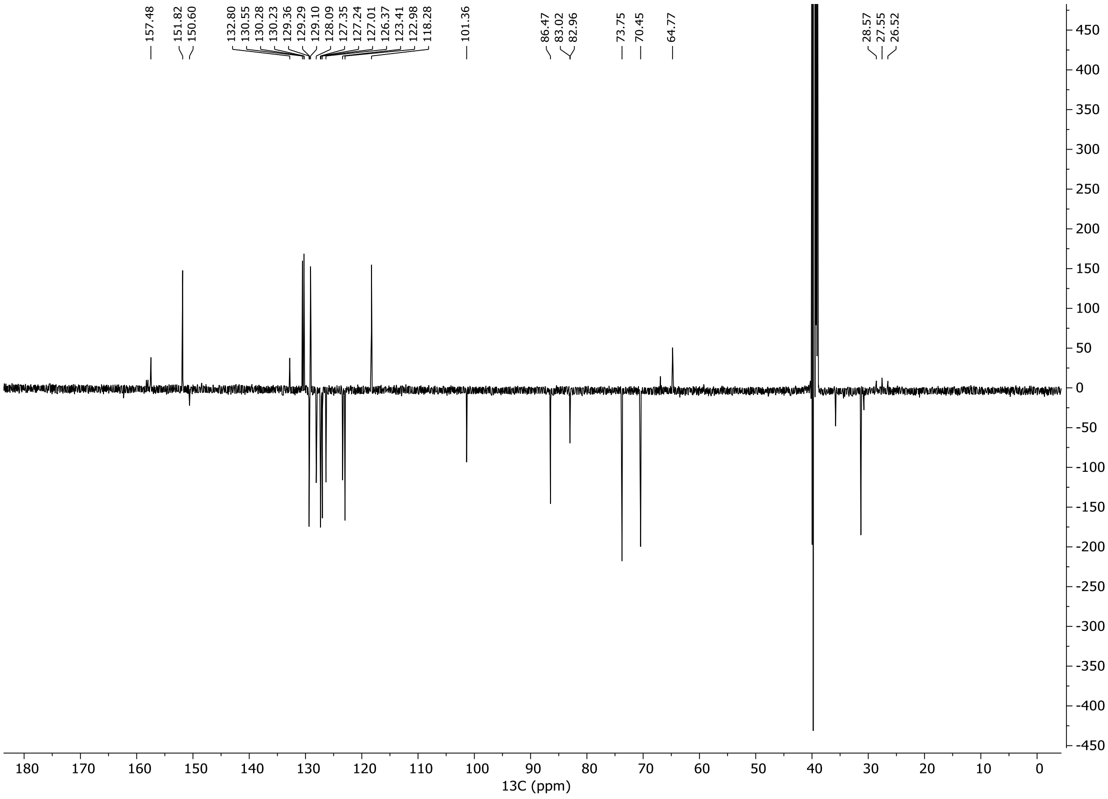
157.48
151.82
150.60
132.80
130.55
130.28
130.23
129.36
129.29
129.10
128.09
127.35
127.24
127.01
126.37
123.41
122.98
118.28
101.36
86.47
83.02
82.96
73.75
70.45
64.77
28.57
27.55
26.52
13C (ppm)

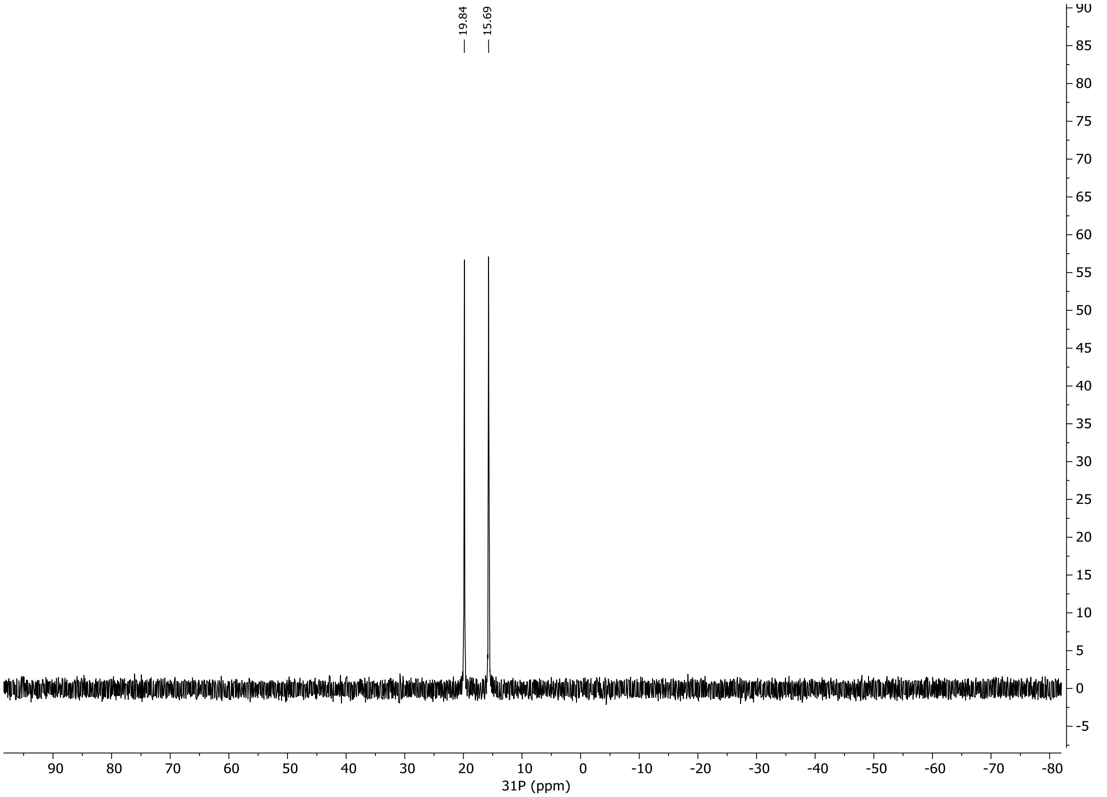
19.84
15.69
31P (ppm)

NMR spectra of compound **4A.12**

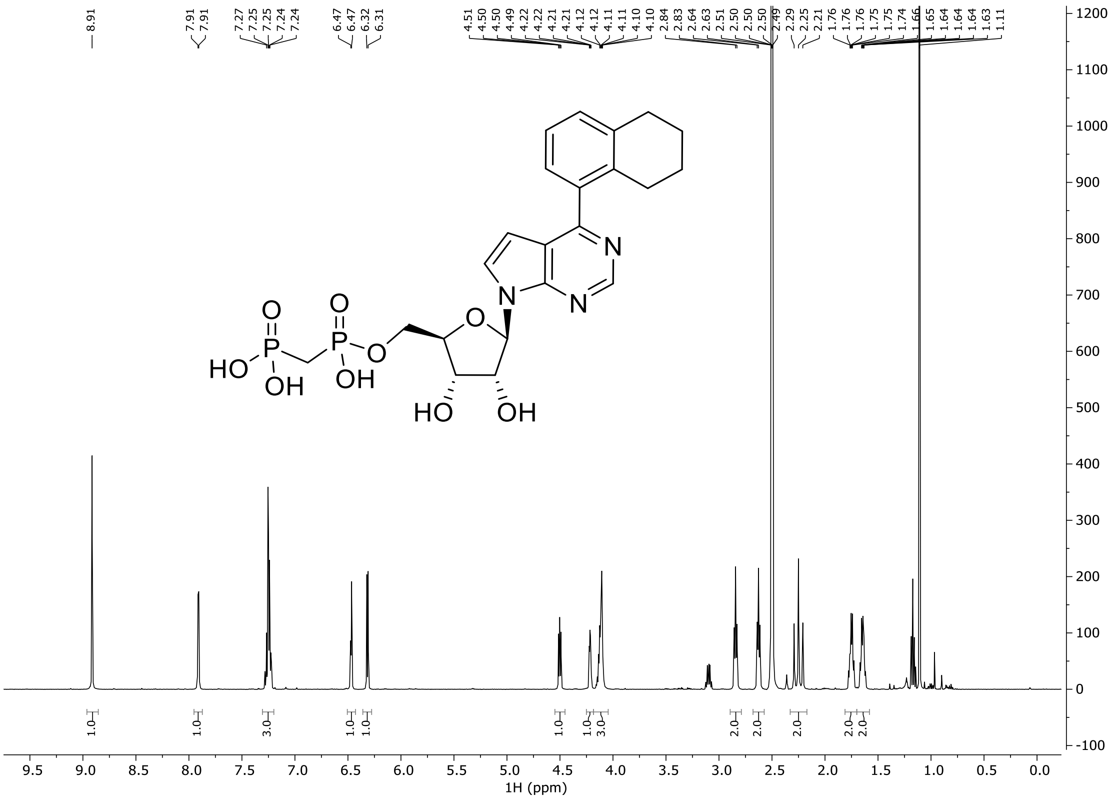

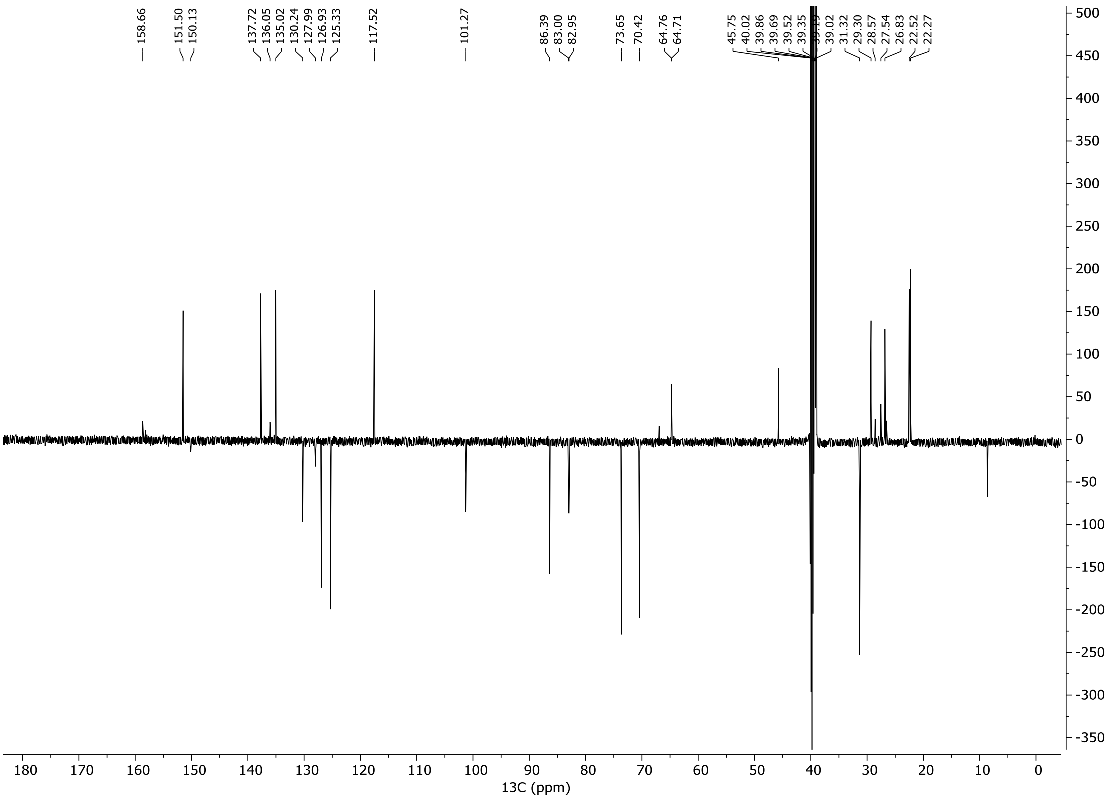

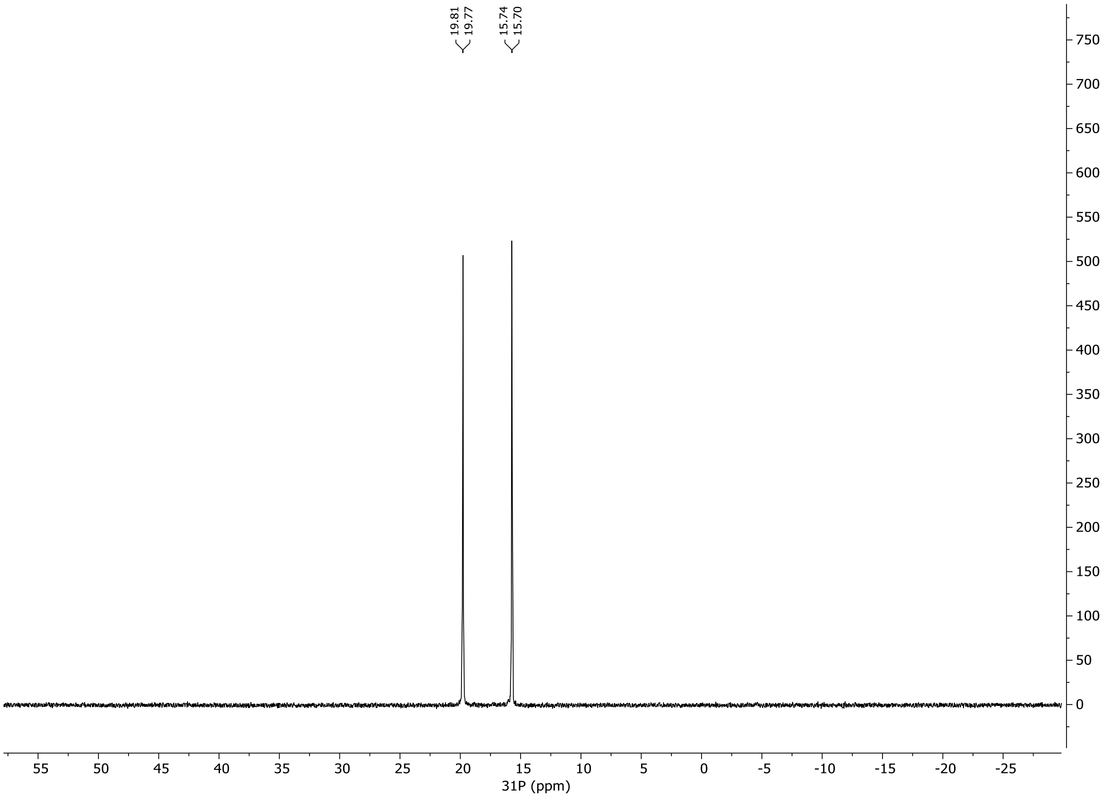

NMR spectra of compound **4A.13**

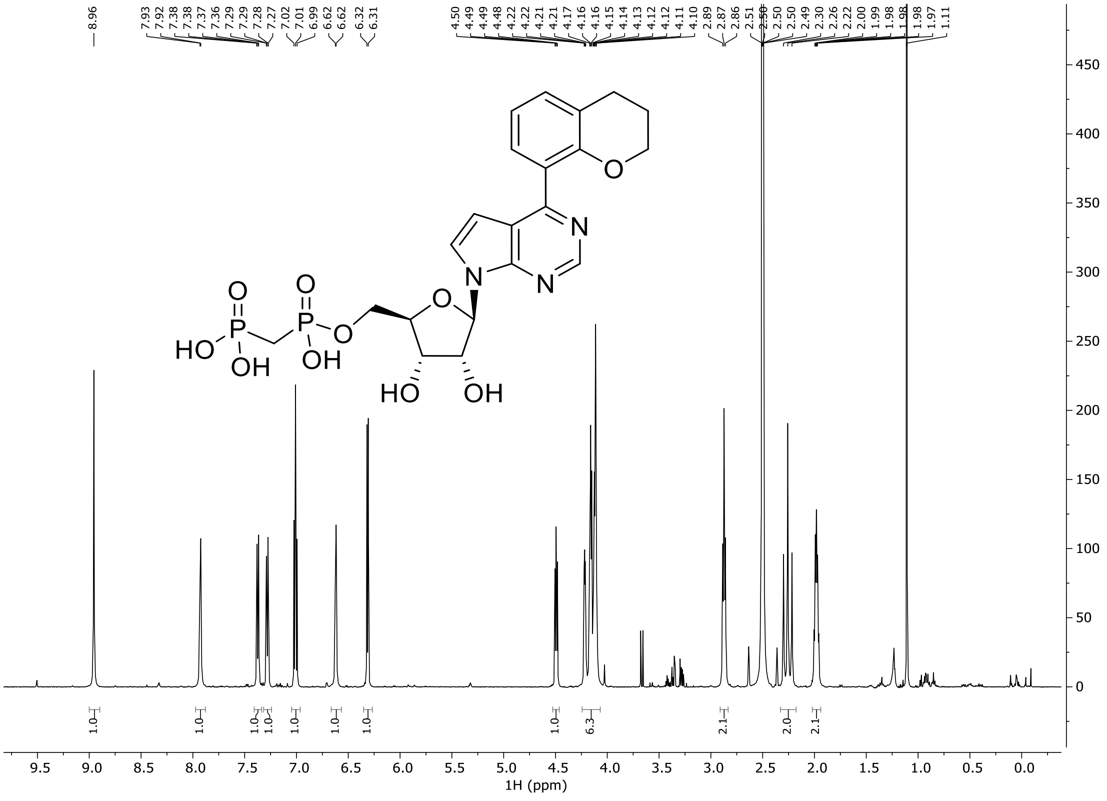

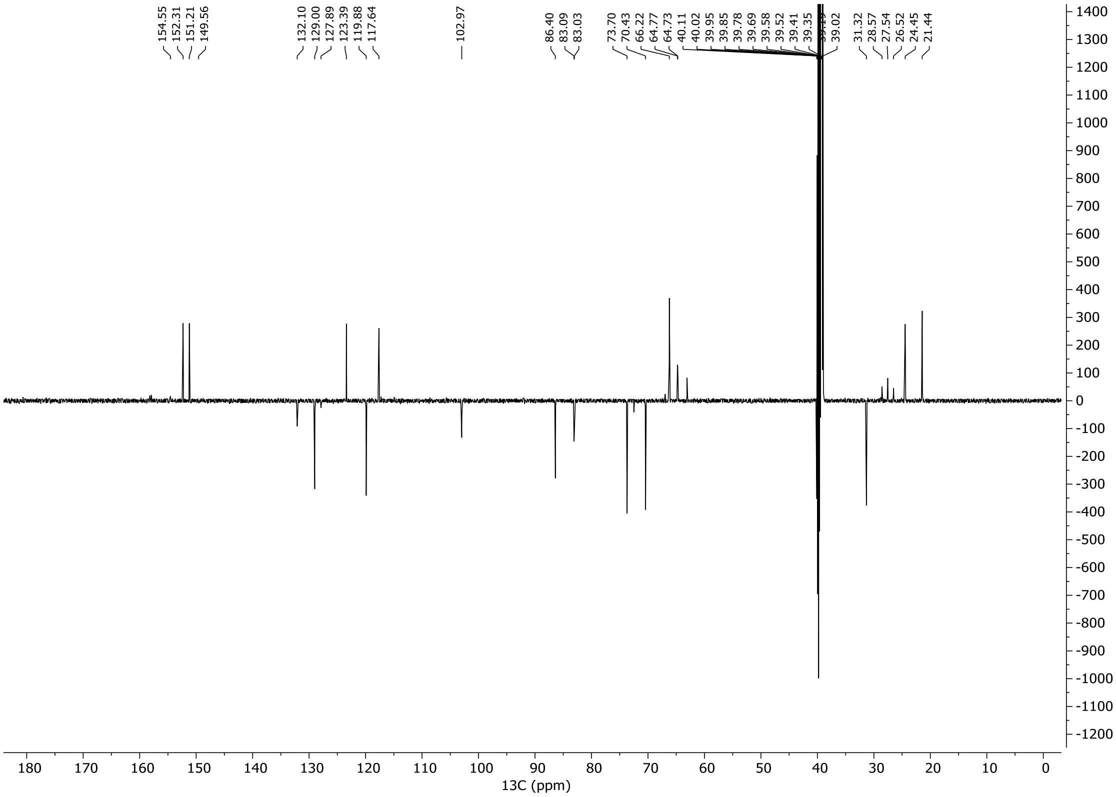
154.55
152.31
151.21
149.56
132.10
129.00
127.89
123.39
119.88
117.64
102.97
86.40
83.09
83.03
73.70
70.43
66.22
64.77
64.73
40.11
40.02
39.95
39.85
39.78
39.69
39.58
39.52
39.41
39.35
39.02
31.32
28.57
27.54
26.52
24.45
21.44
13C (ppm)

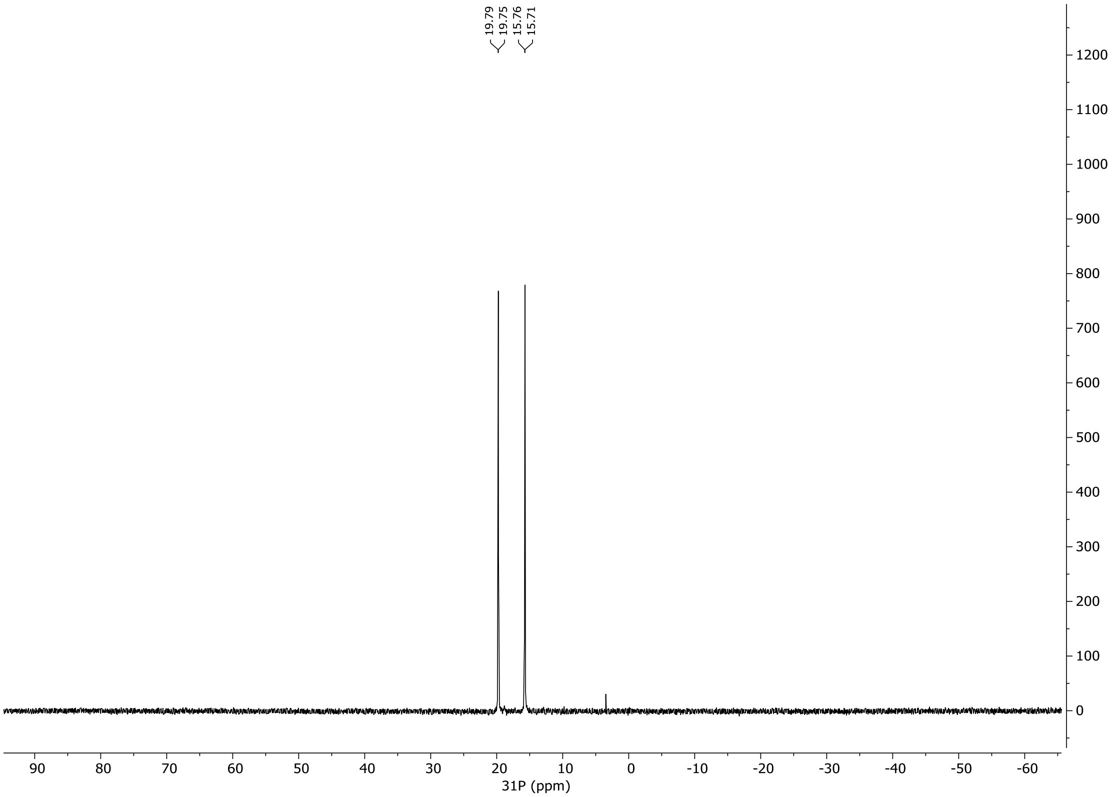
19.79
19.75
15.76
15.71
31P (ppm)

NMR spectra of compound **4A.14**

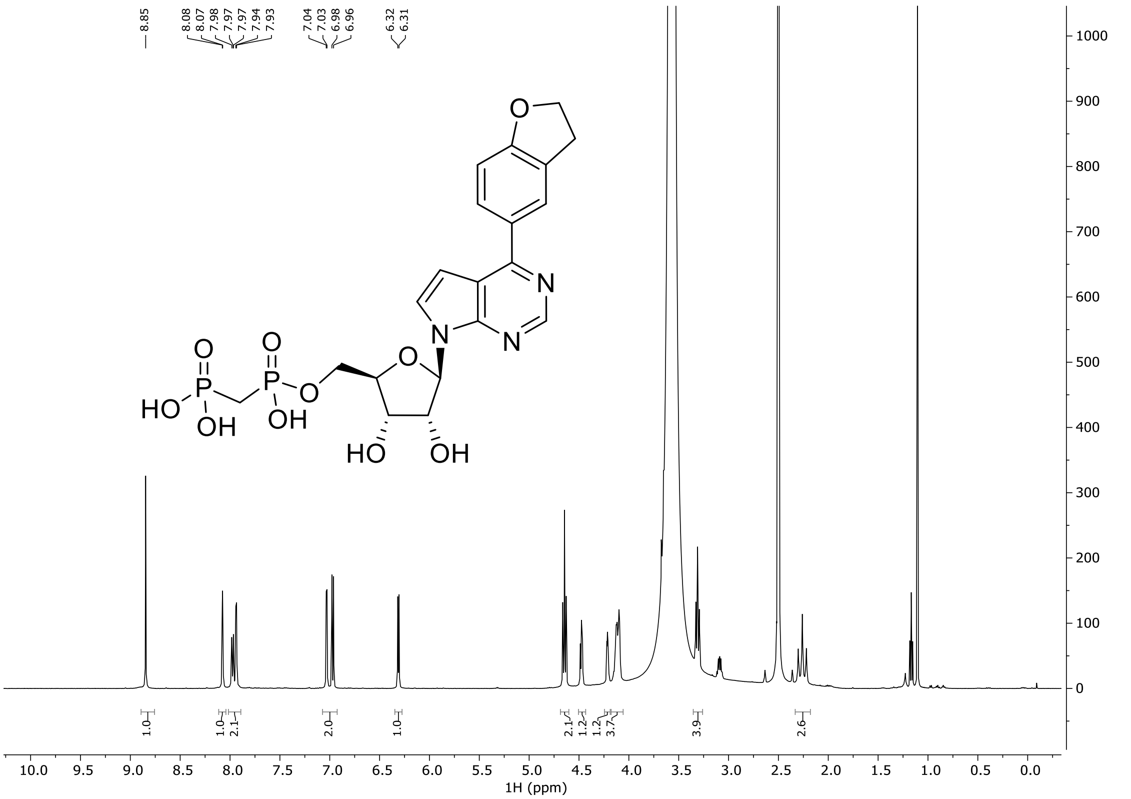

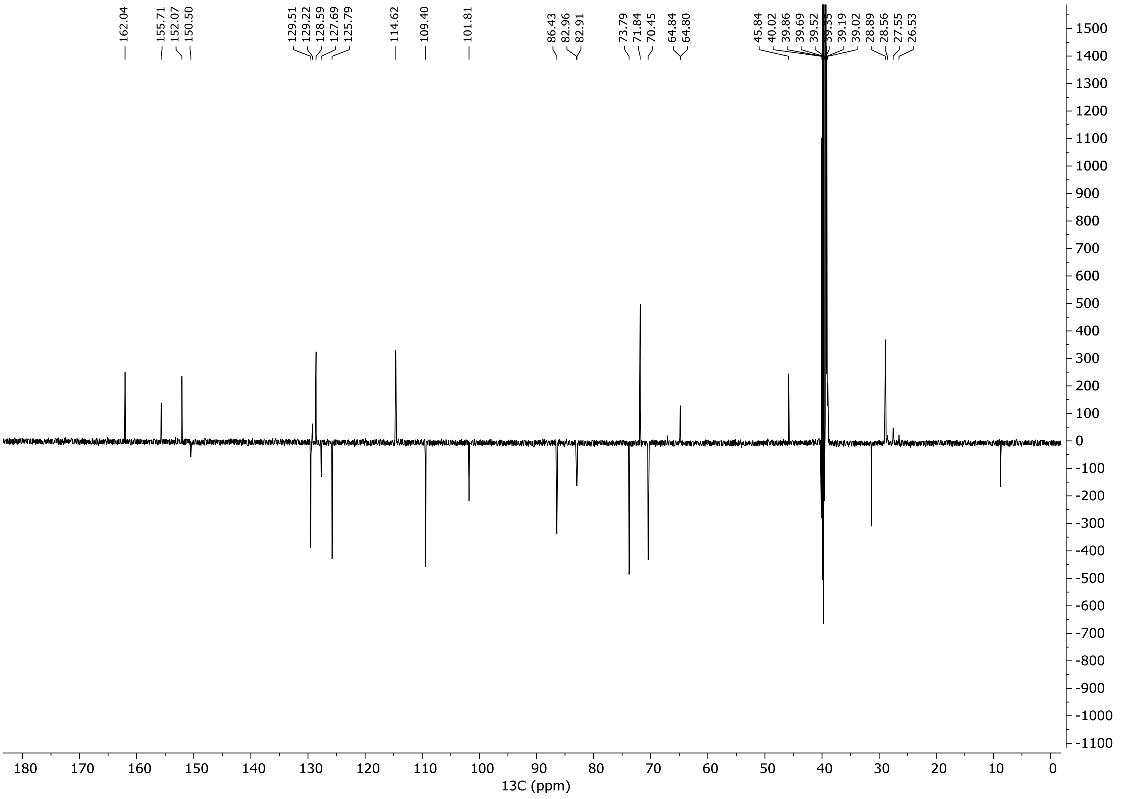

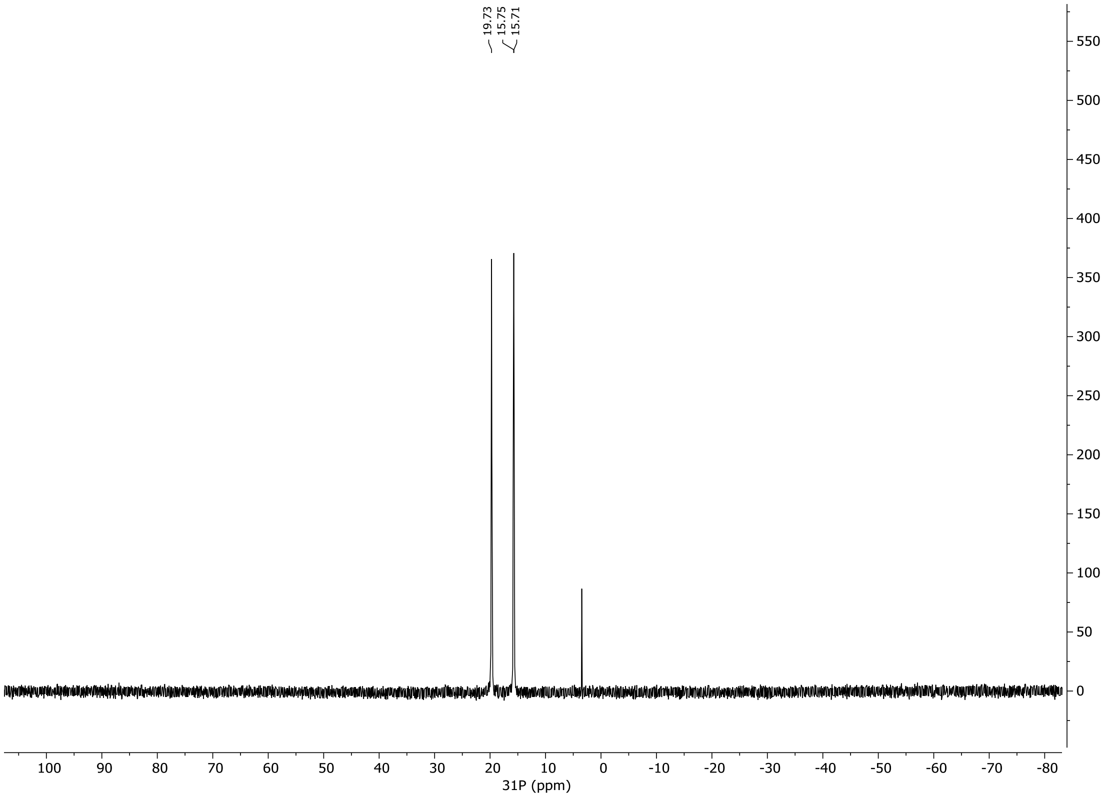

NMR spectra of compound **4A.15**

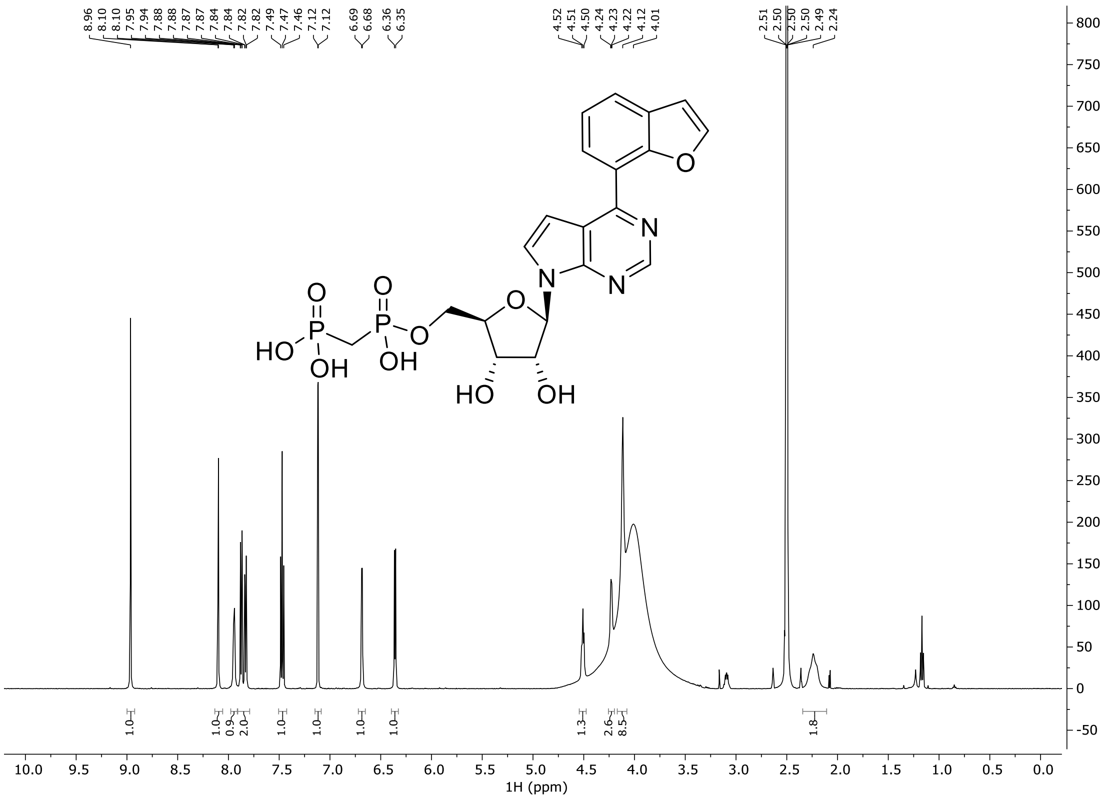

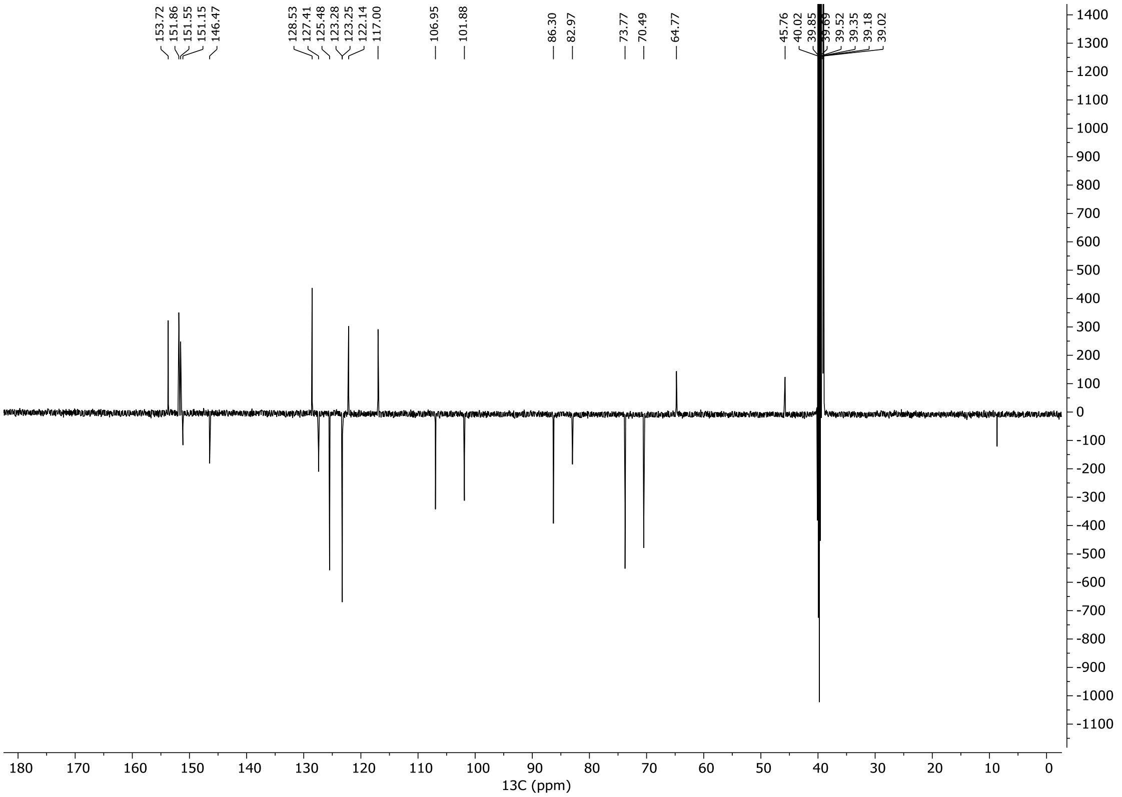

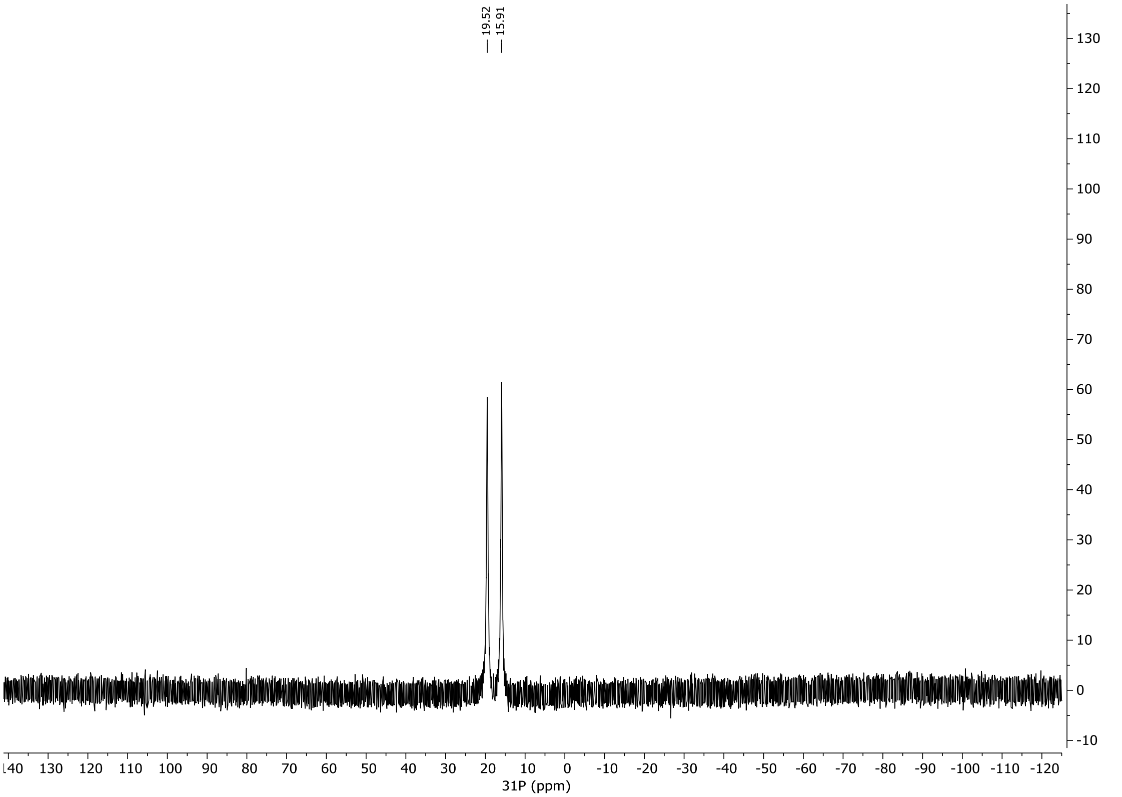

NMR spectra of compound **4A.16**

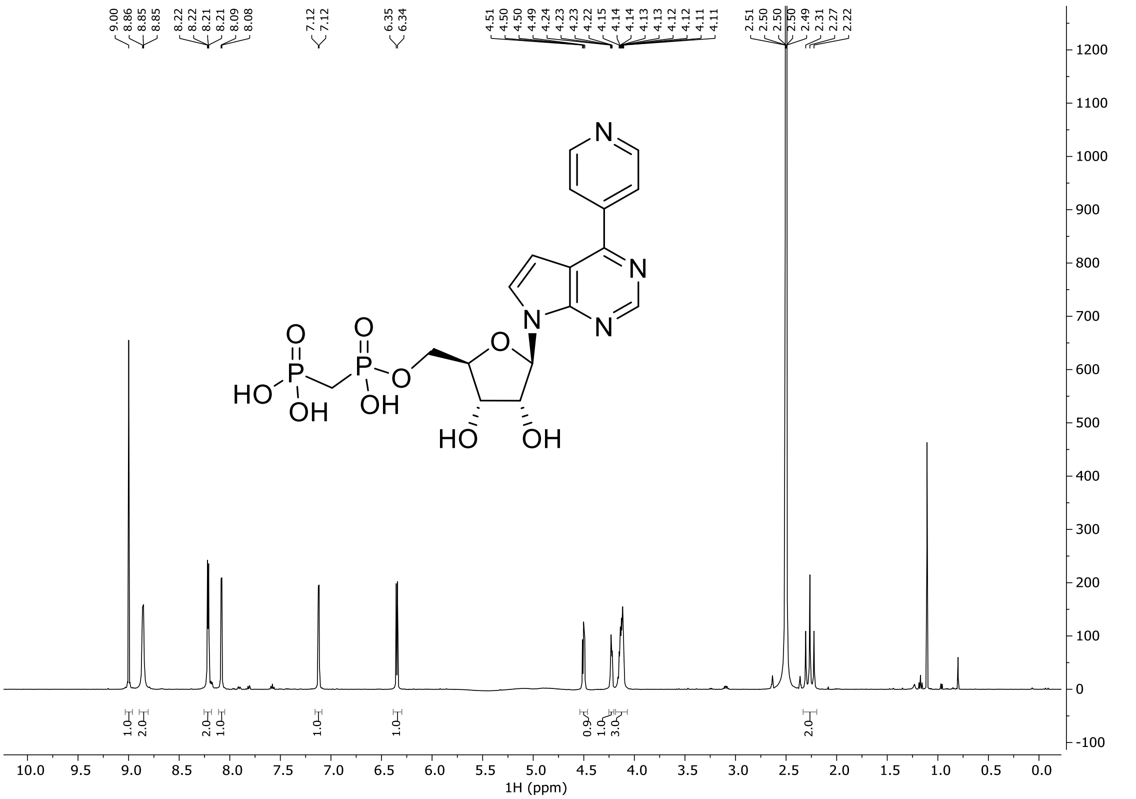

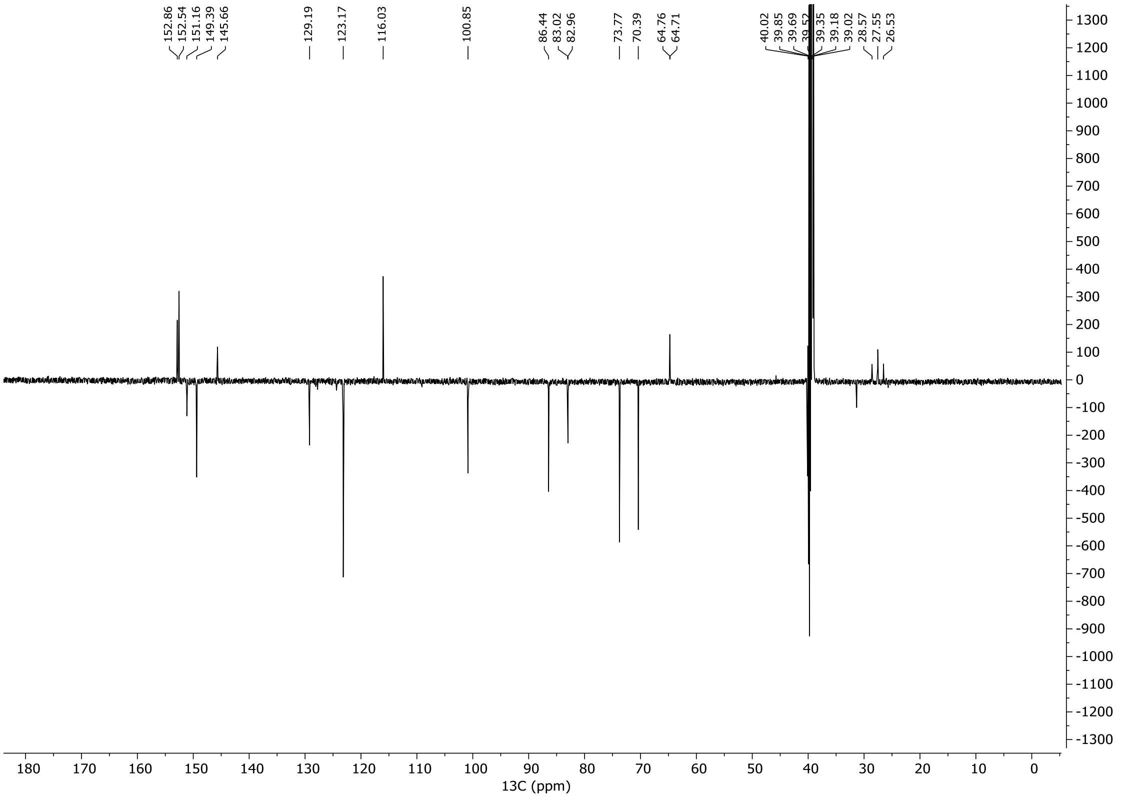

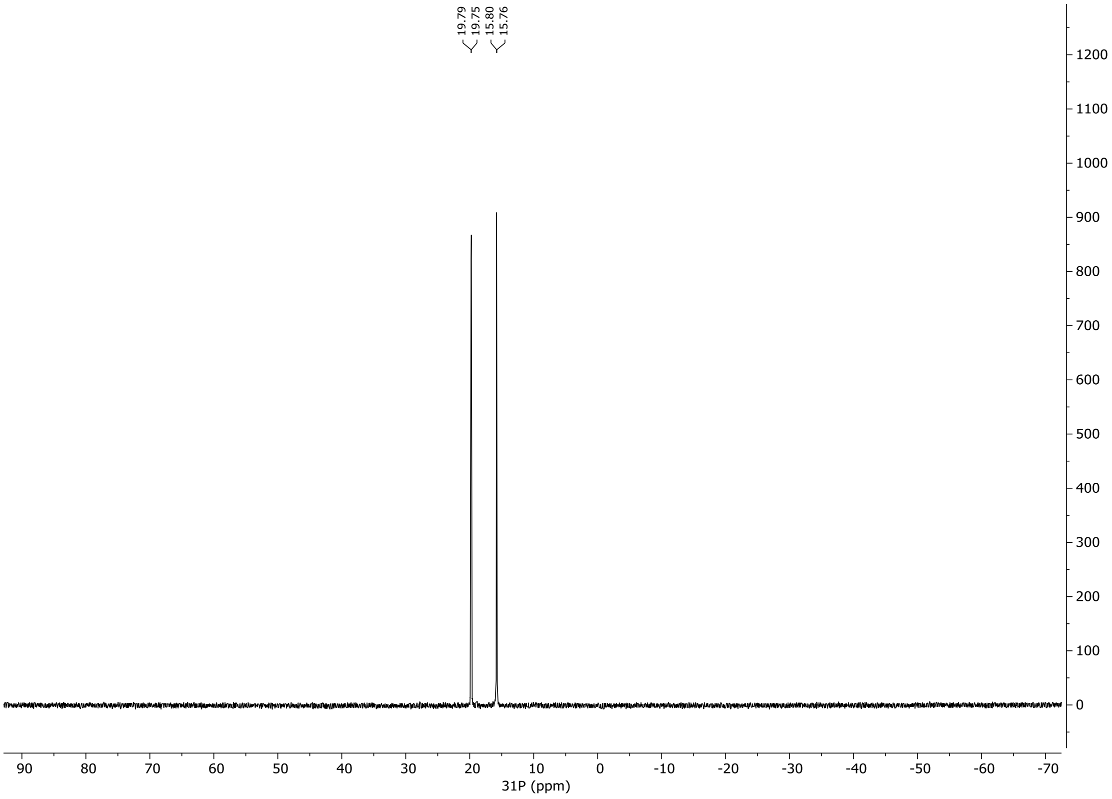

NMR spectra of compound **4A.17**

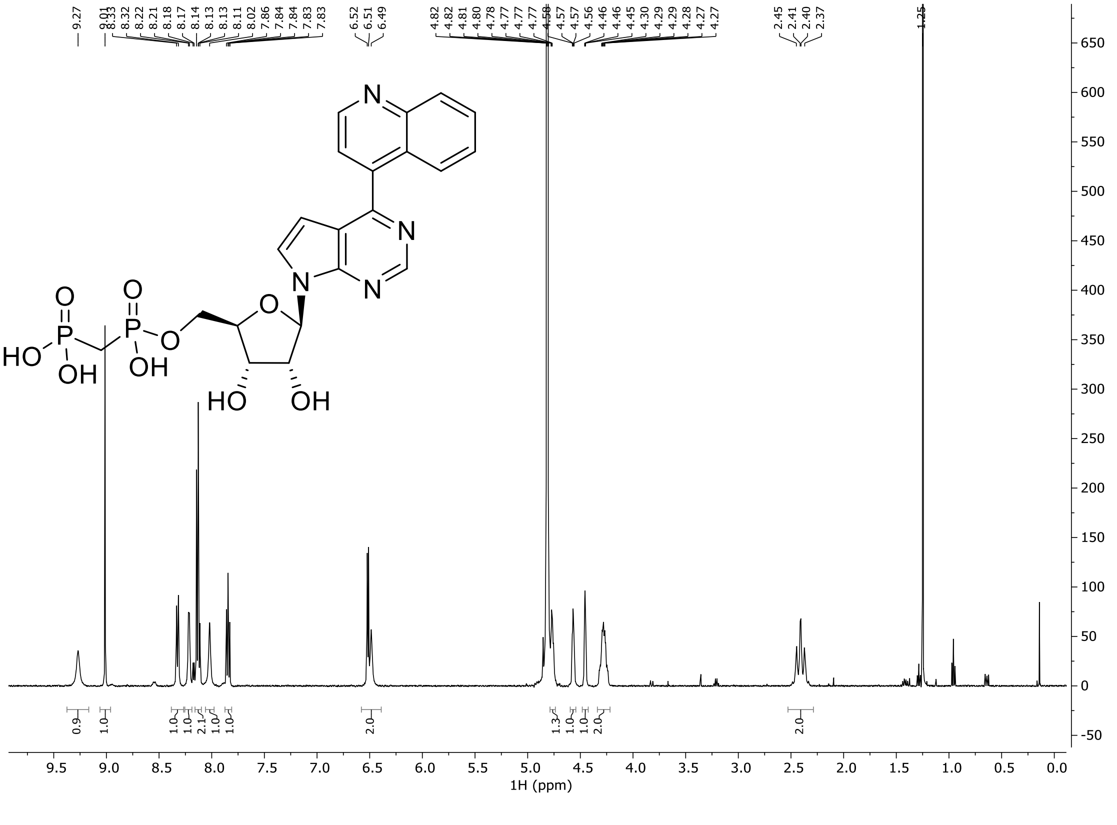

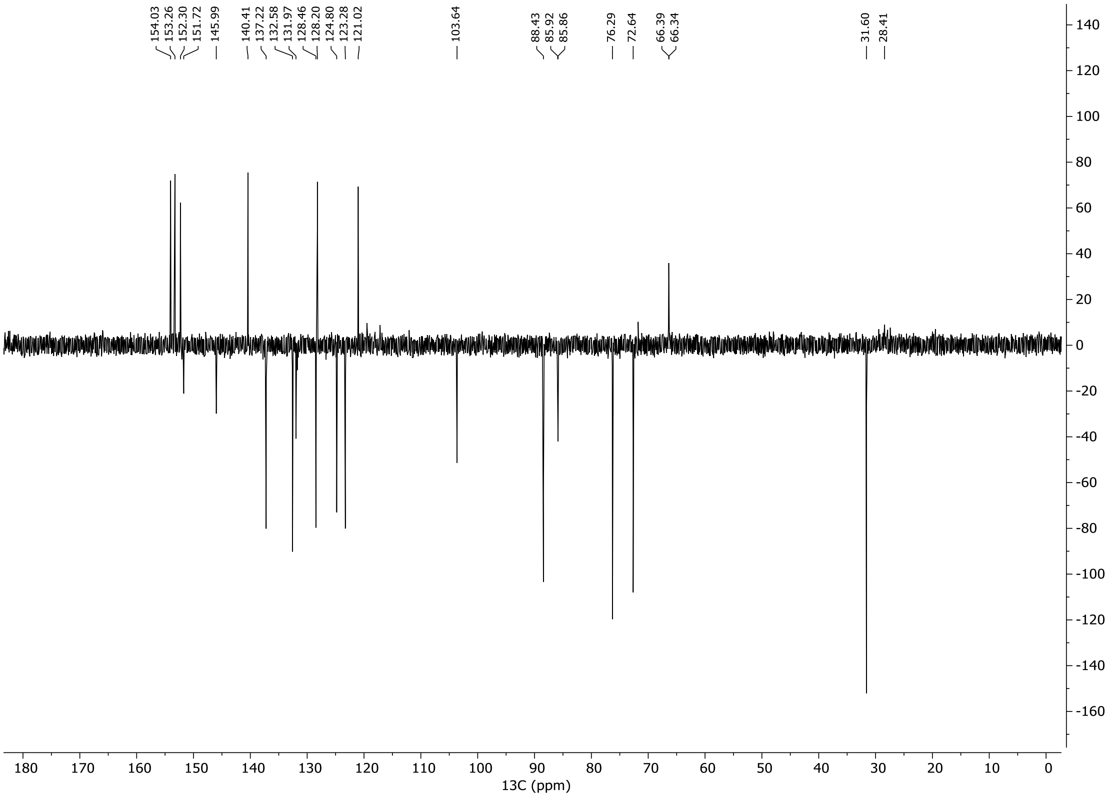
154.03
153.26
152.30
151.72
145.99
140.41
137.22
132.58
131.97
128.46
128.20
124.80
123.28
121.02
103.64
88.43
85.92
85.86
76.29
72.64
66.39
66.34
31.60
28.41
13C (ppm)

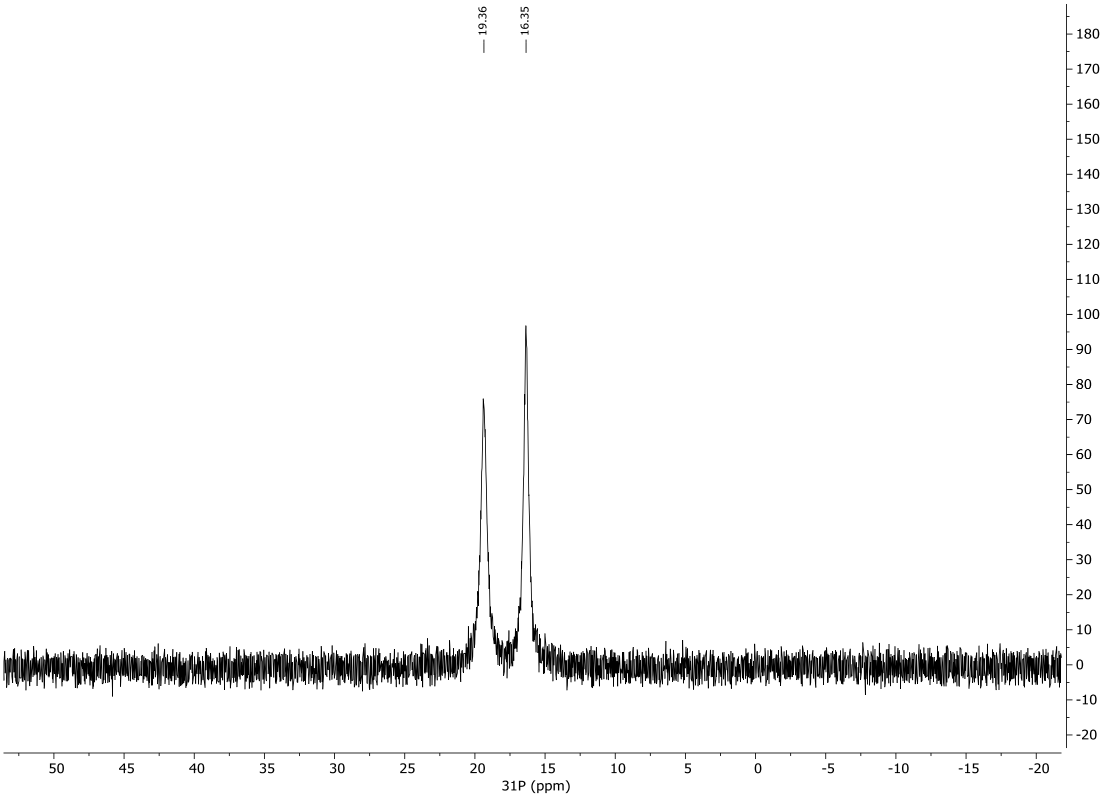
19.36
16.35
31P (ppm)

NMR spectra of compound **4A.18**

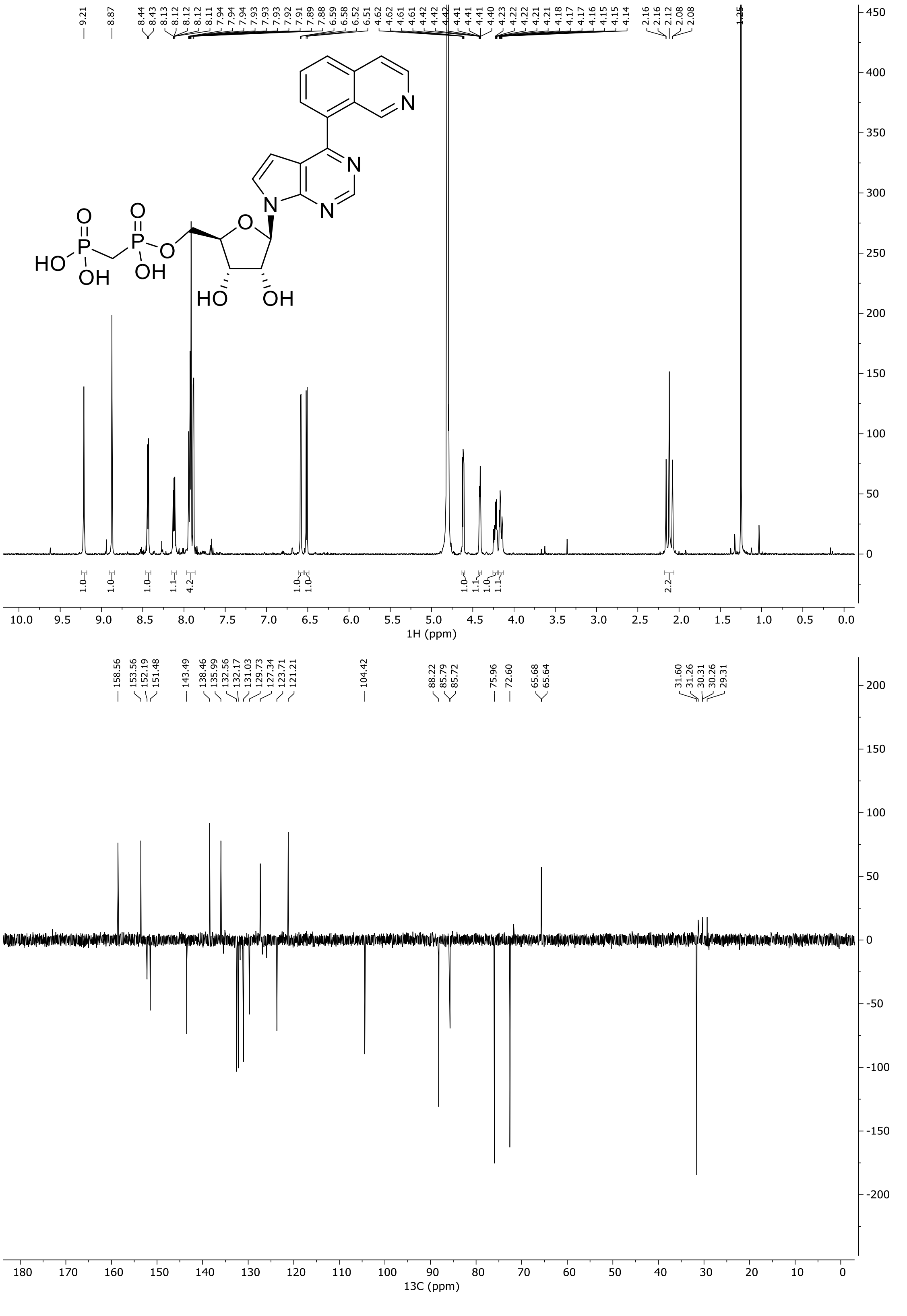

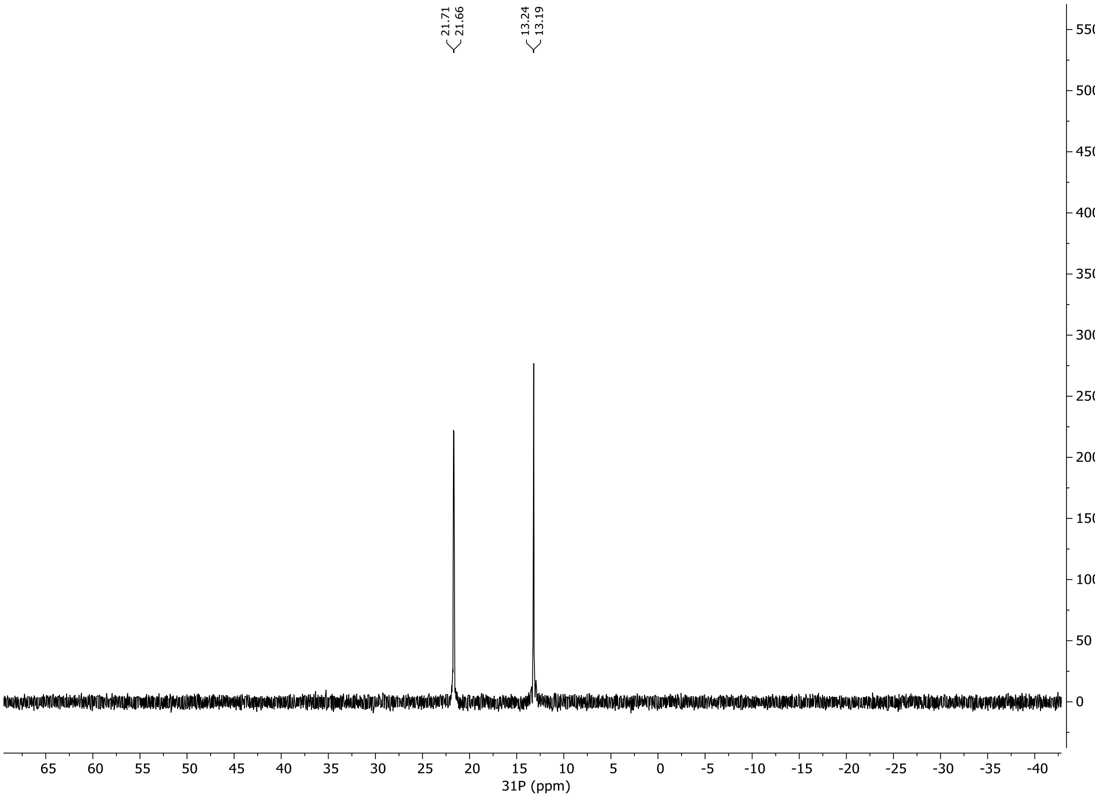

NMR spectra of compound **4A.19**

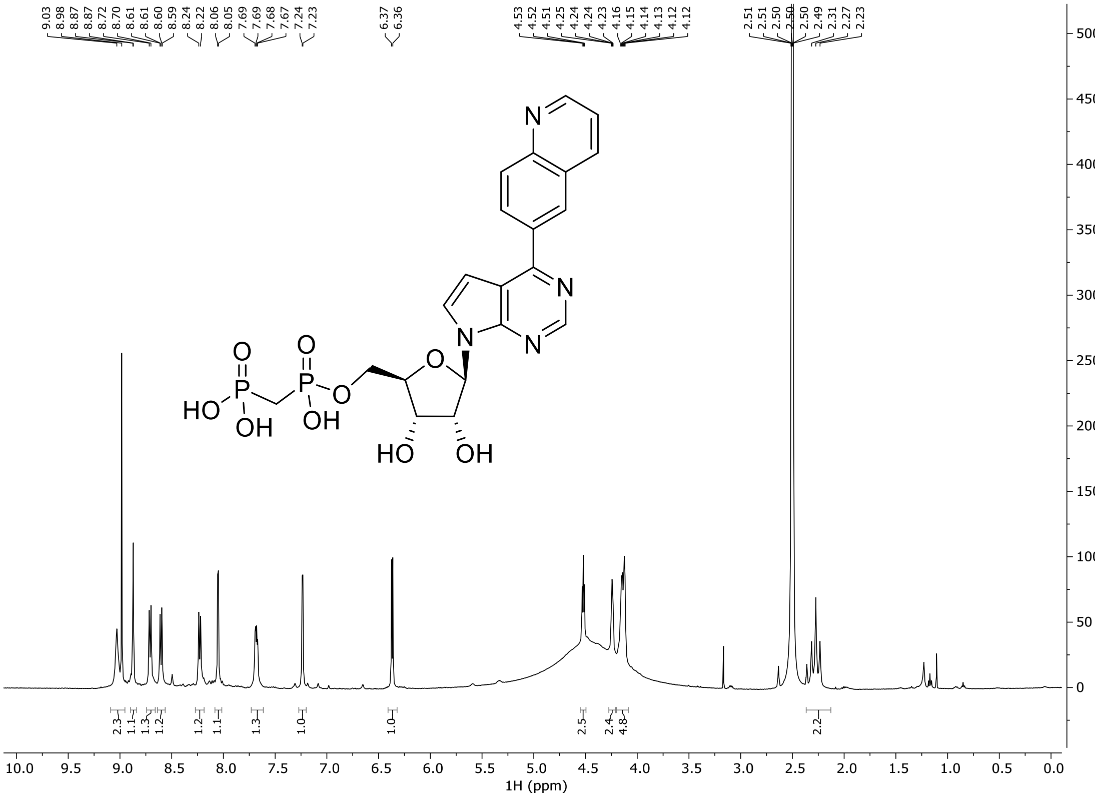

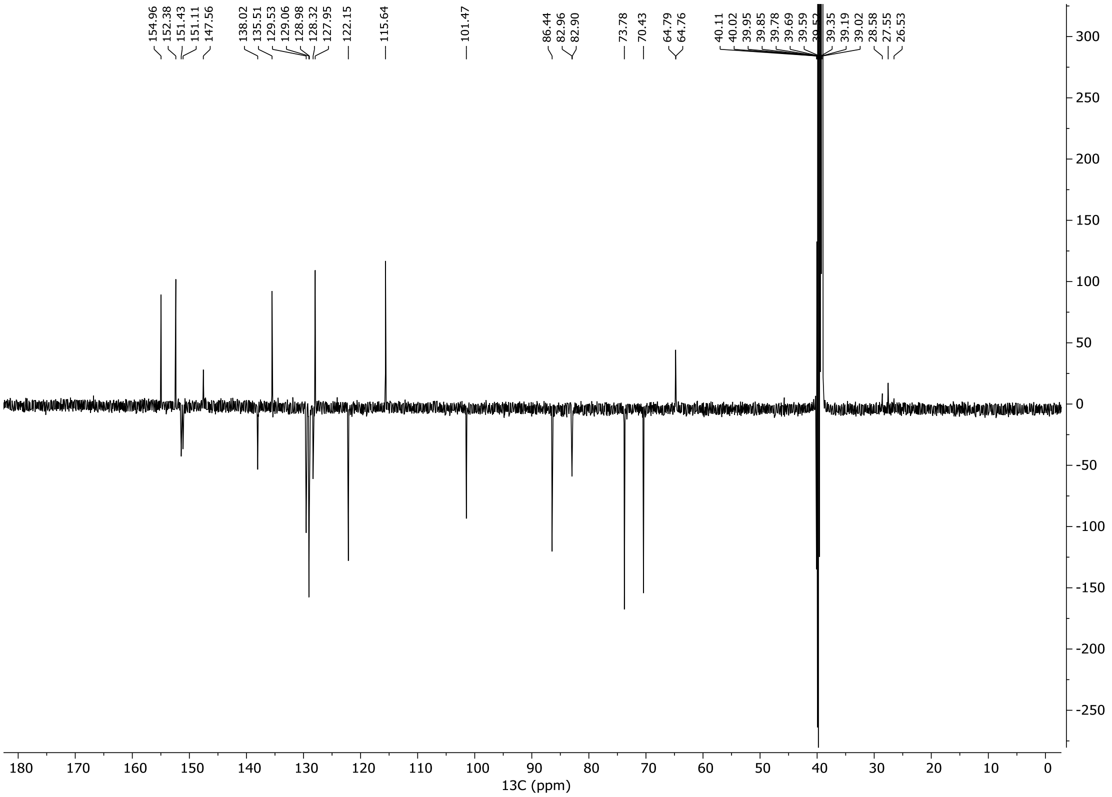
154.96
152.38
151.43
151.11
147.56
138.02
135.51
129.53
129.06
128.98
128.32
127.95
122.15
115.64
101.47
86.44
82.96
82.90
73.78
70.43
64.79
64.76
40.11
40.02
39.95
39.85
39.78
39.69
39.59
39.35
39.19
39.02
28.58
27.55
26.53
13C (ppm)

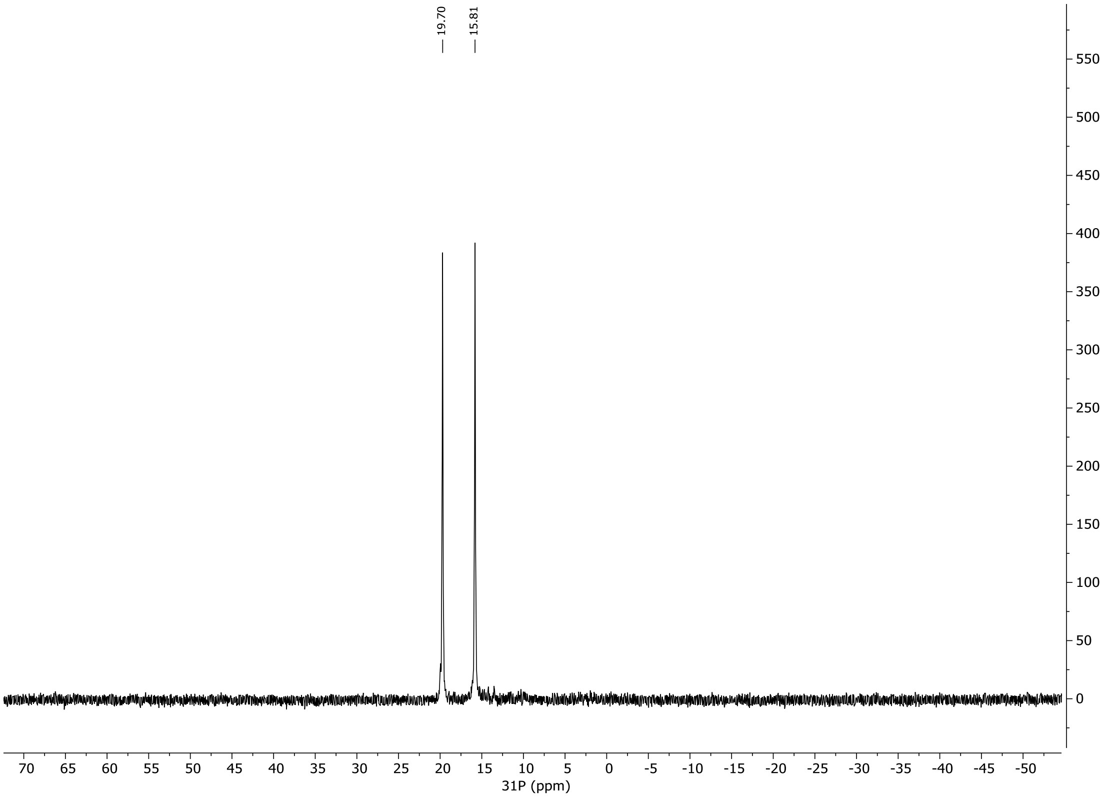
19.70
15.81
31P (ppm)

NMR spectra of compound **4A.20**

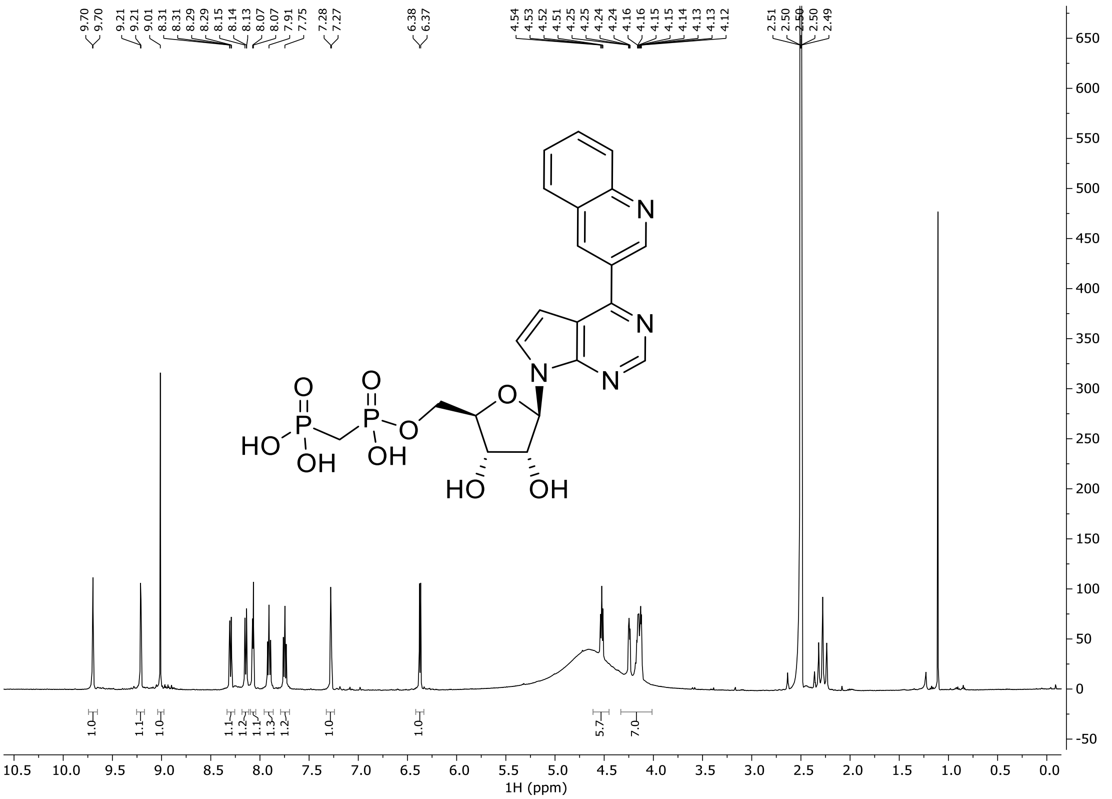

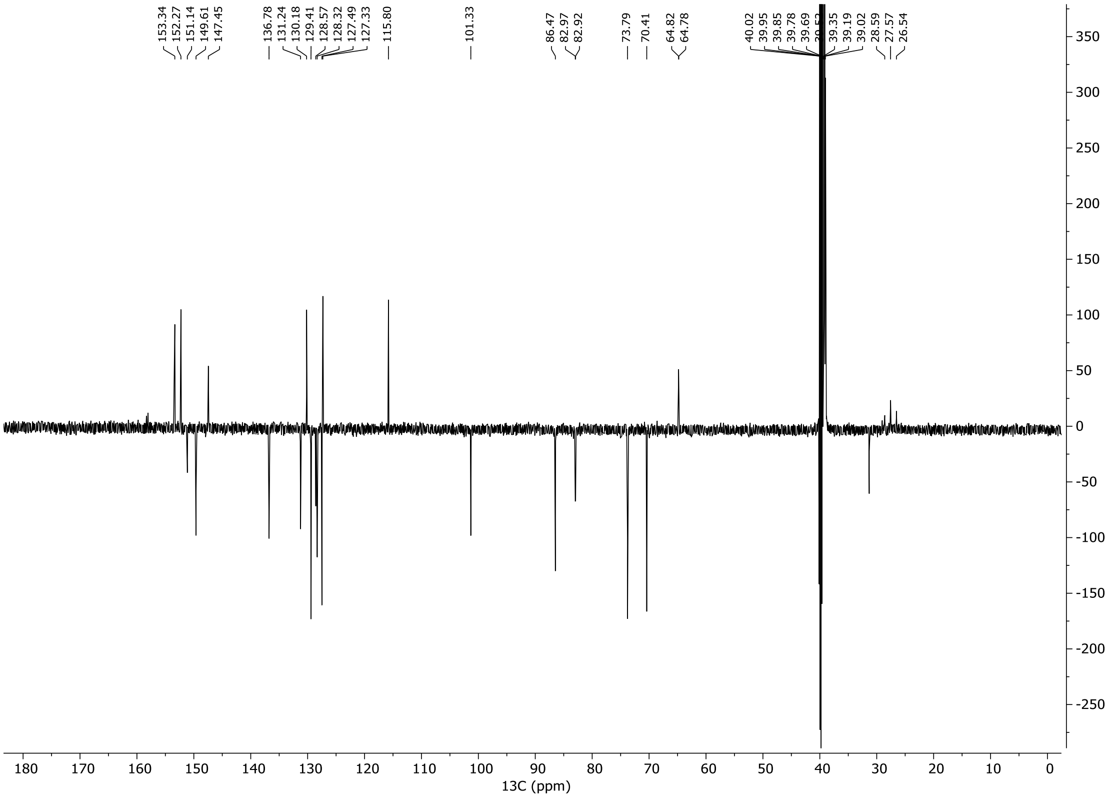

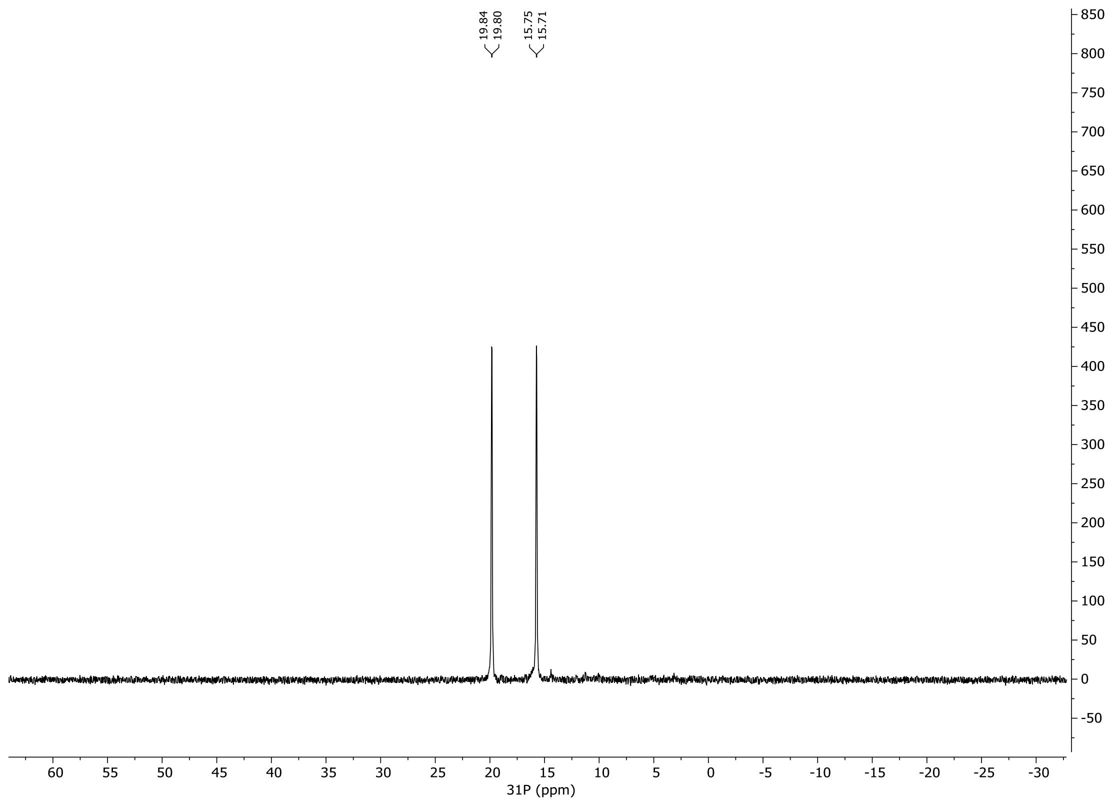

NMR spectra of compound **4A.21**

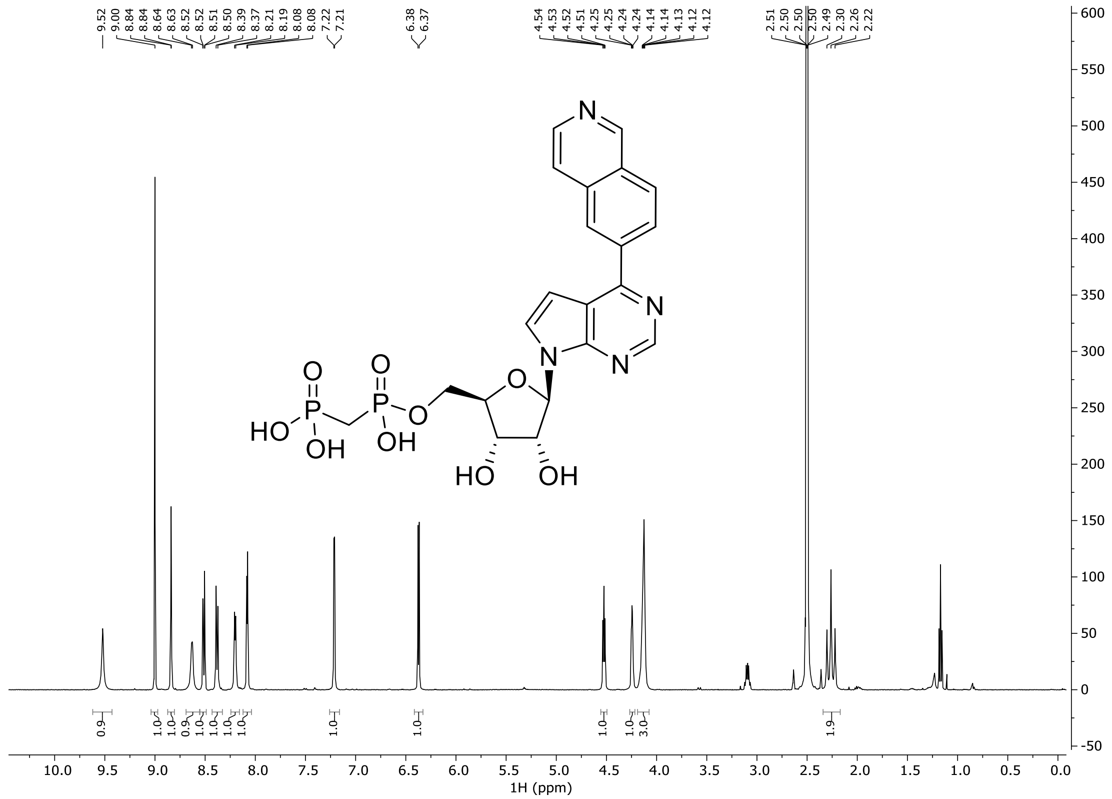

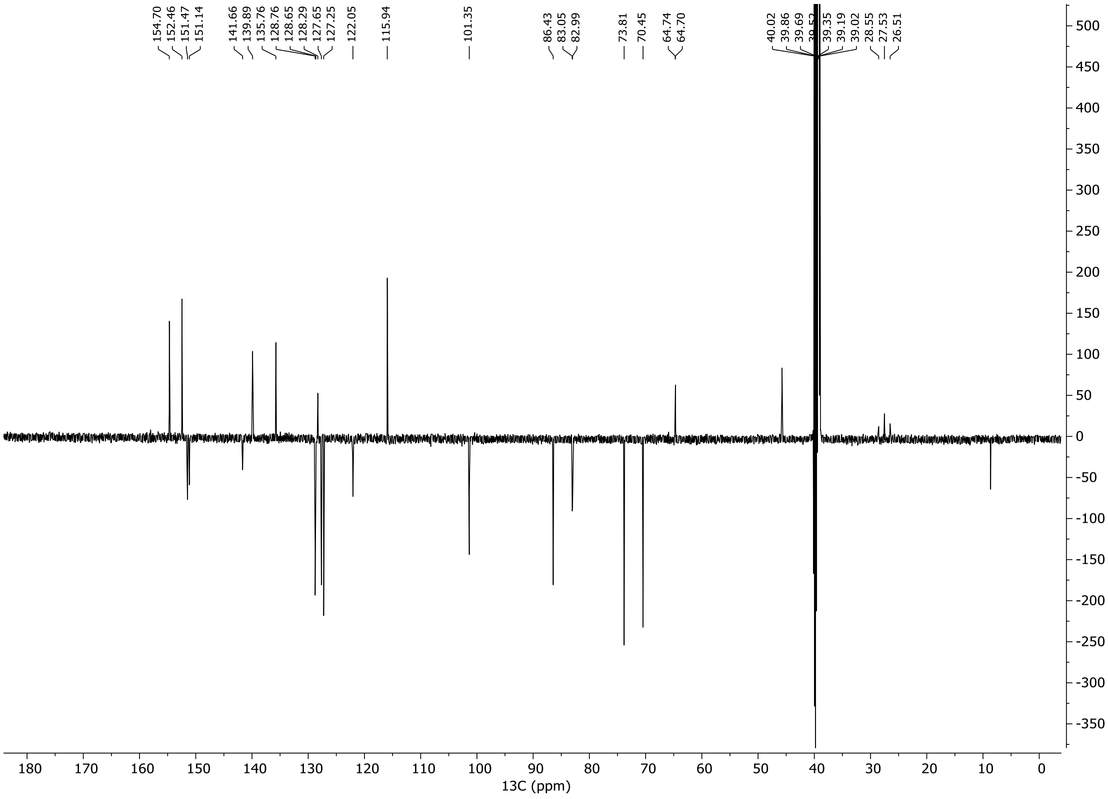
154.70
152.46
151.47
151.14
141.66
139.89
135.76
128.76
128.65
128.29
127.65
127.25
122.05
115.94
101.35
86.43
83.05
82.99
73.81
70.45
64.74
64.70
40.02
39.86
39.69
39.35
39.19
39.02
28.55
27.53
26.51
13C (ppm)

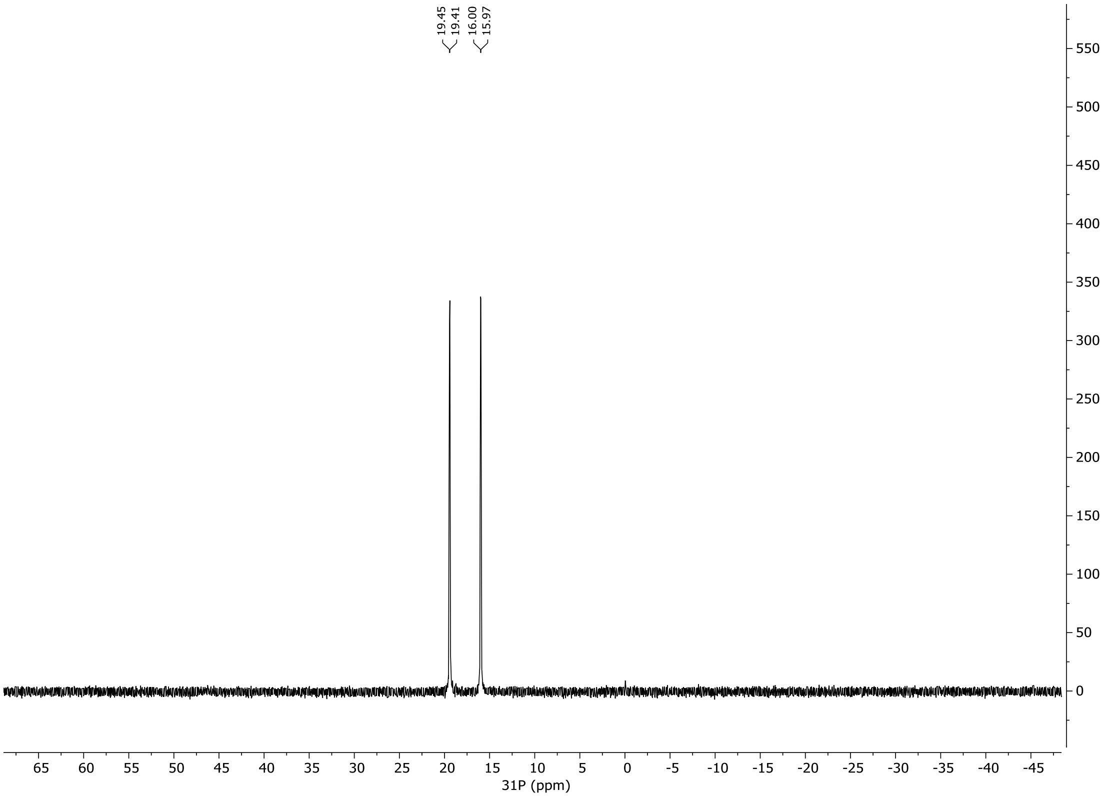
19.45
19.41
16.00
15.97
31P (ppm)

NMR spectra of compound **4A.22**

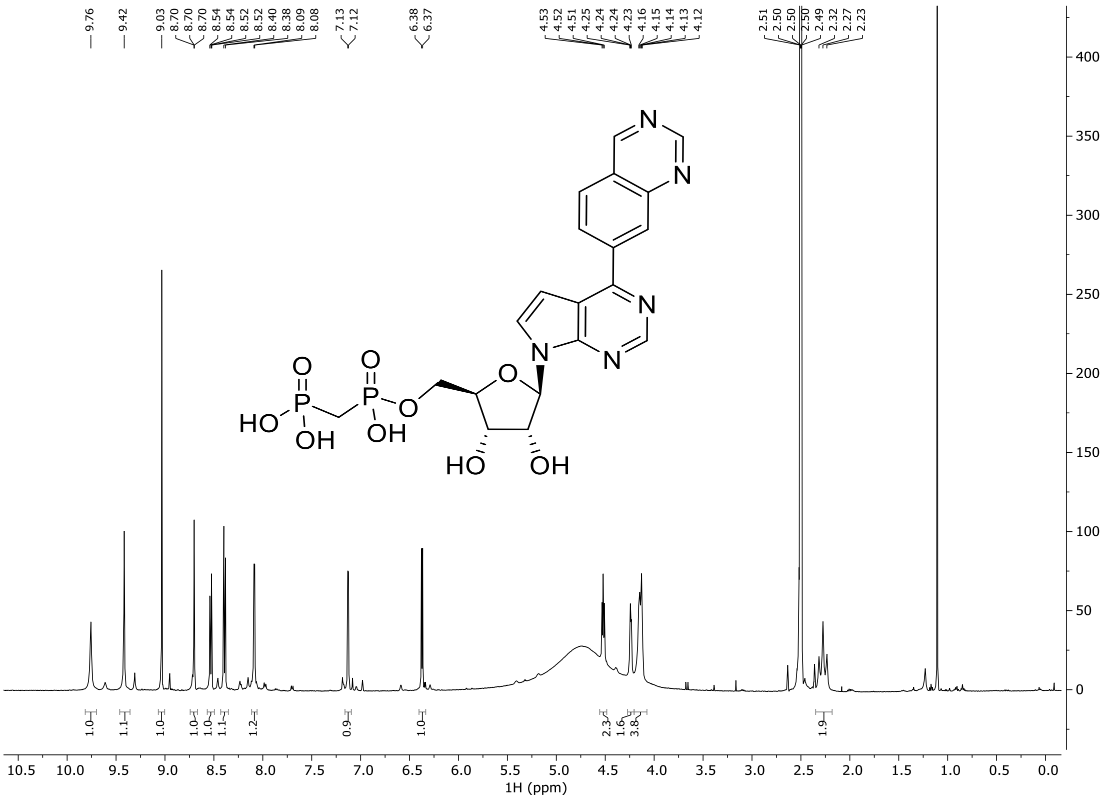

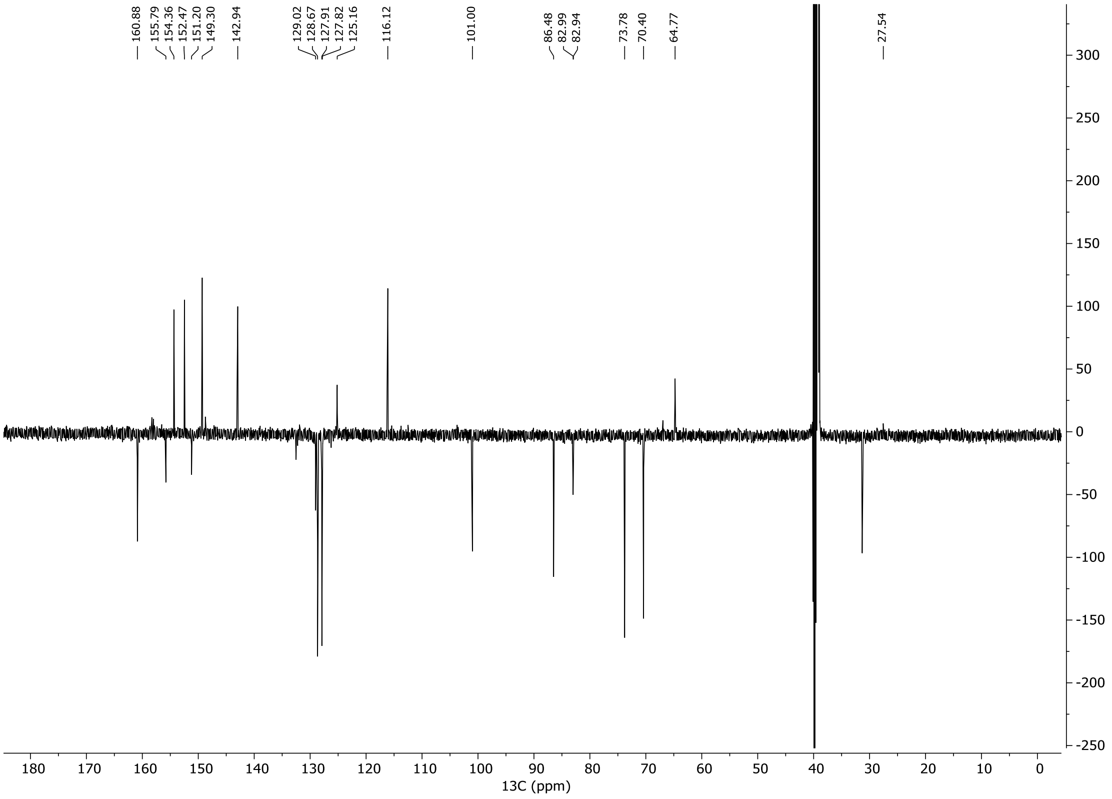

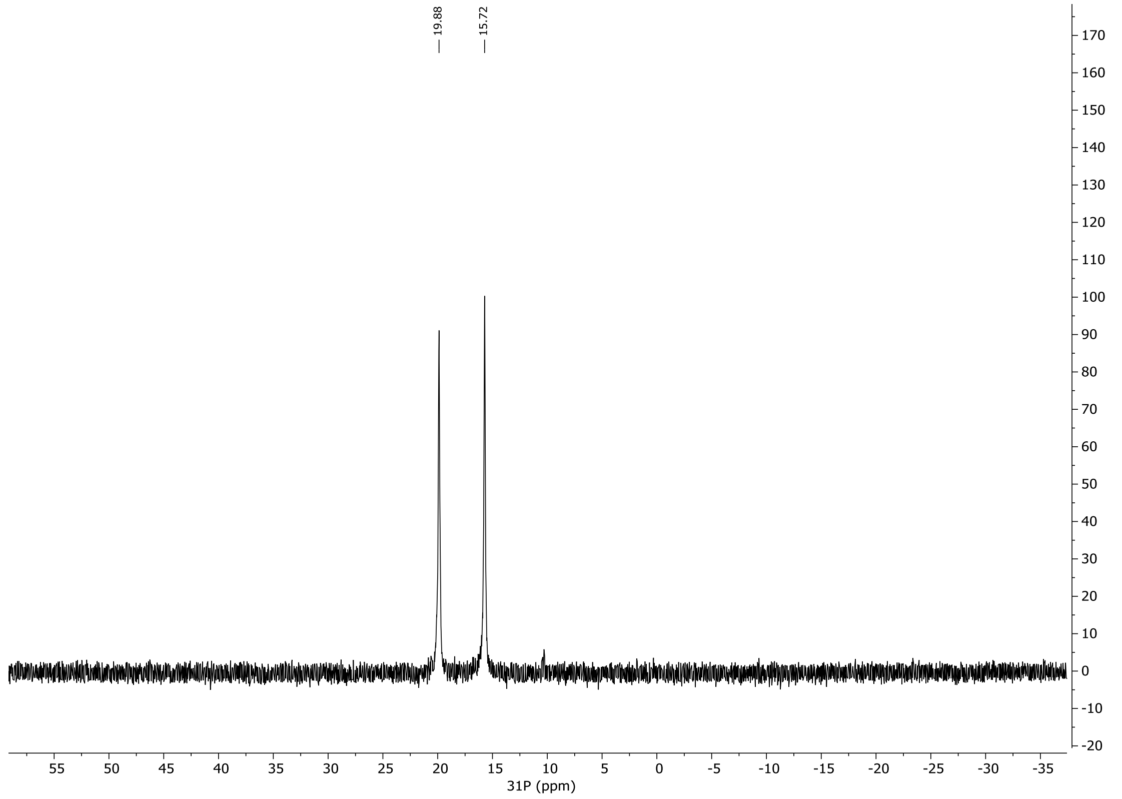

NMR spectra of compound **6B.6**

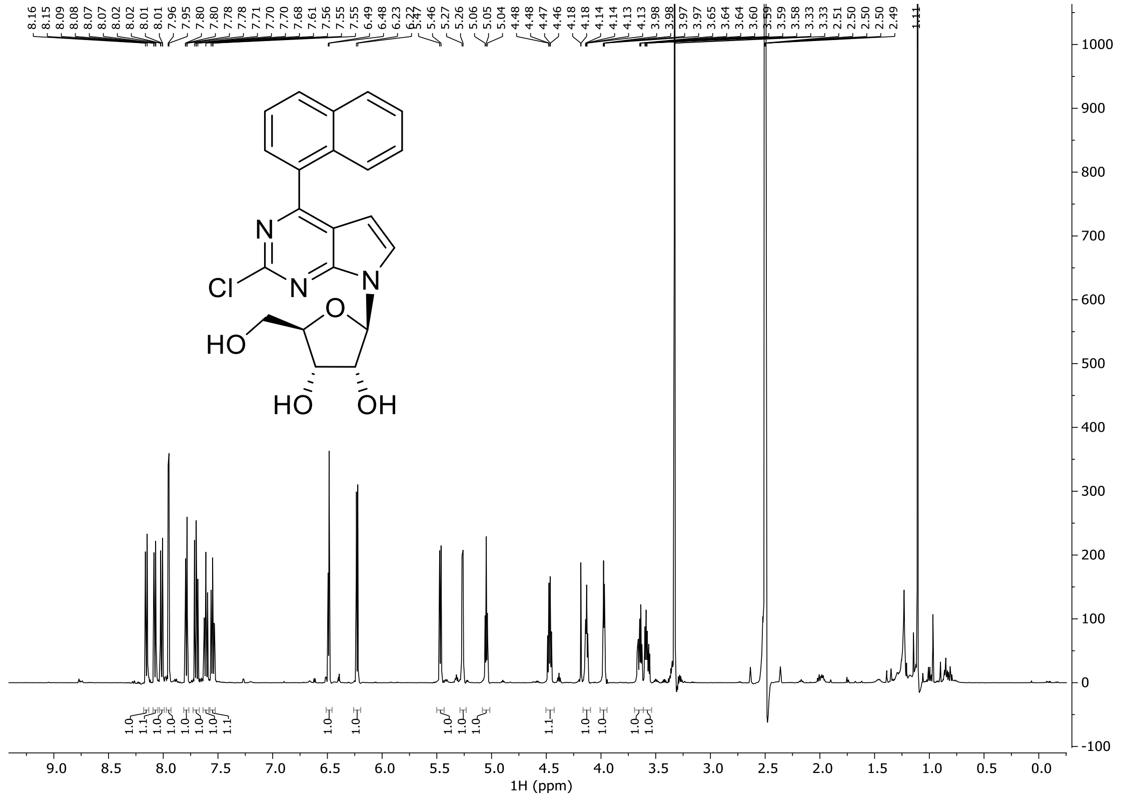

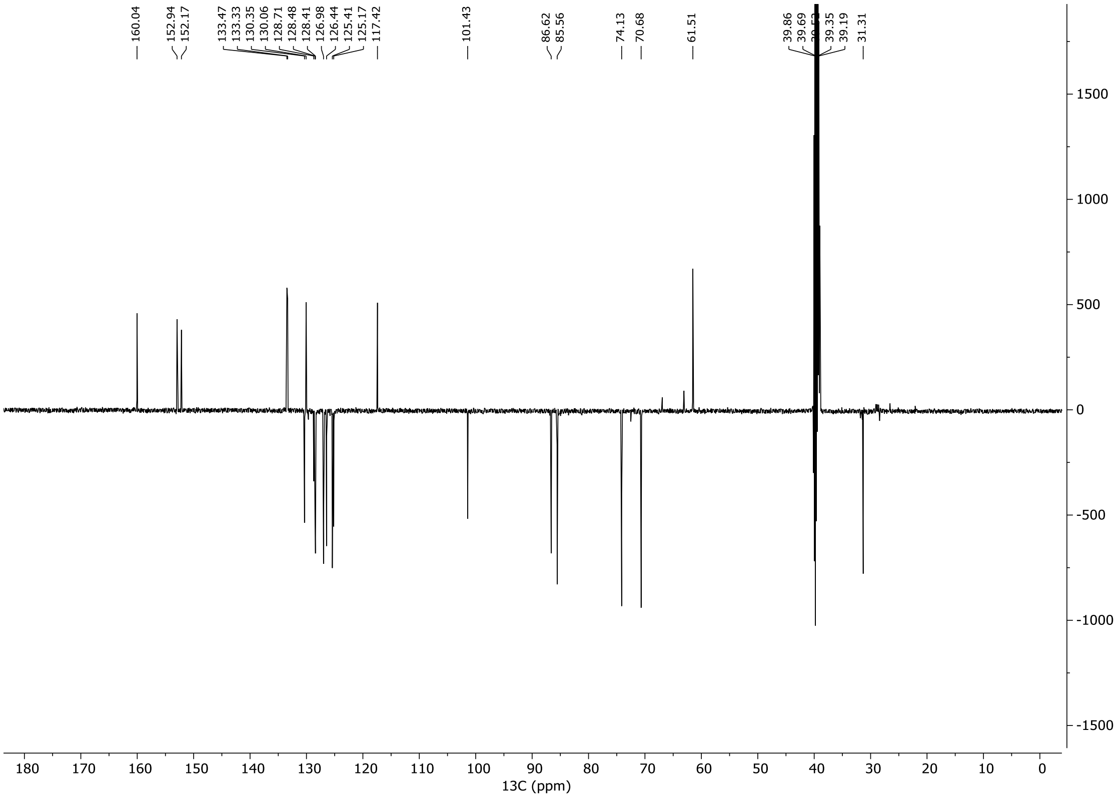

NMR spectra of compound **6B.7**

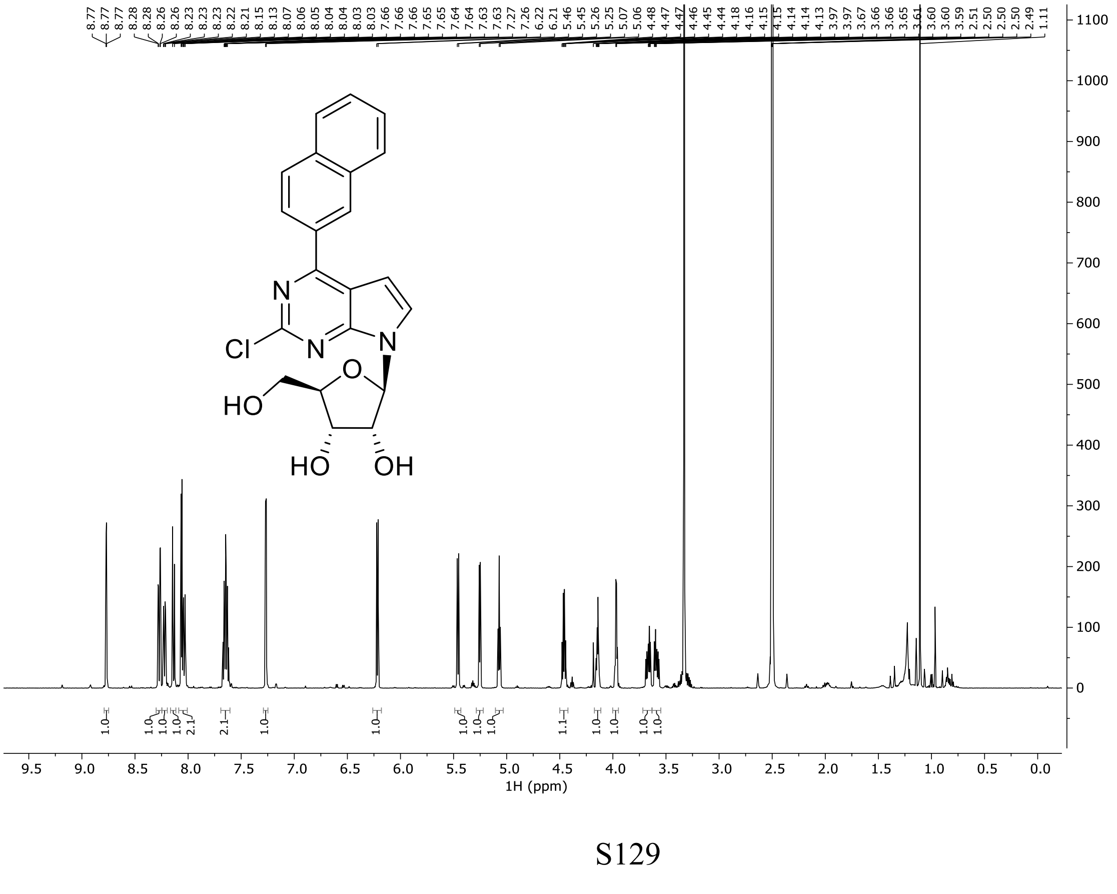

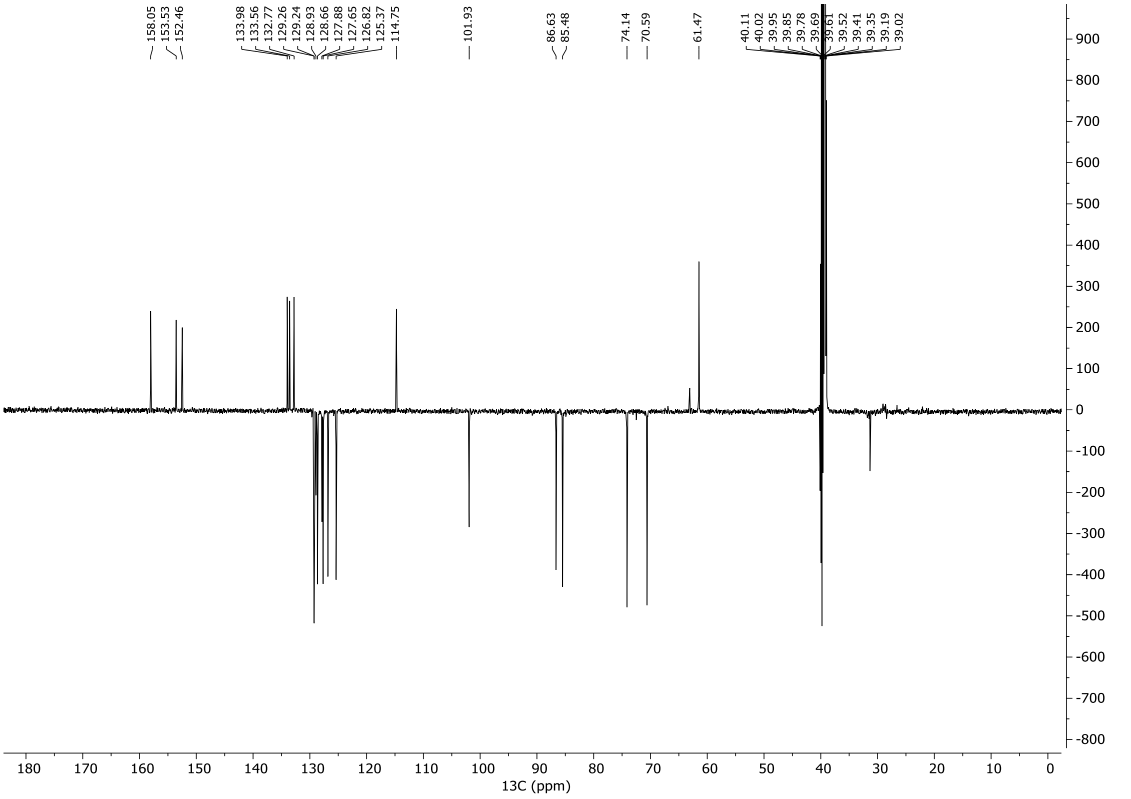

NMR spectra of compound **6B.12**

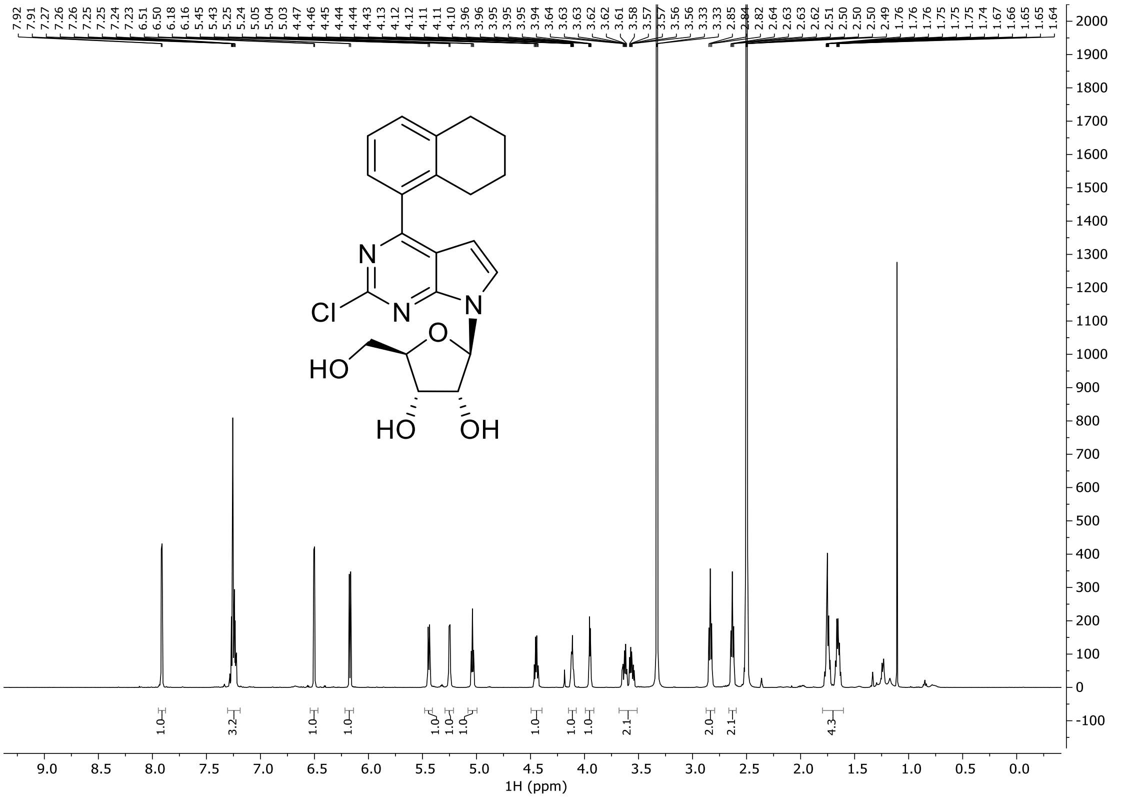

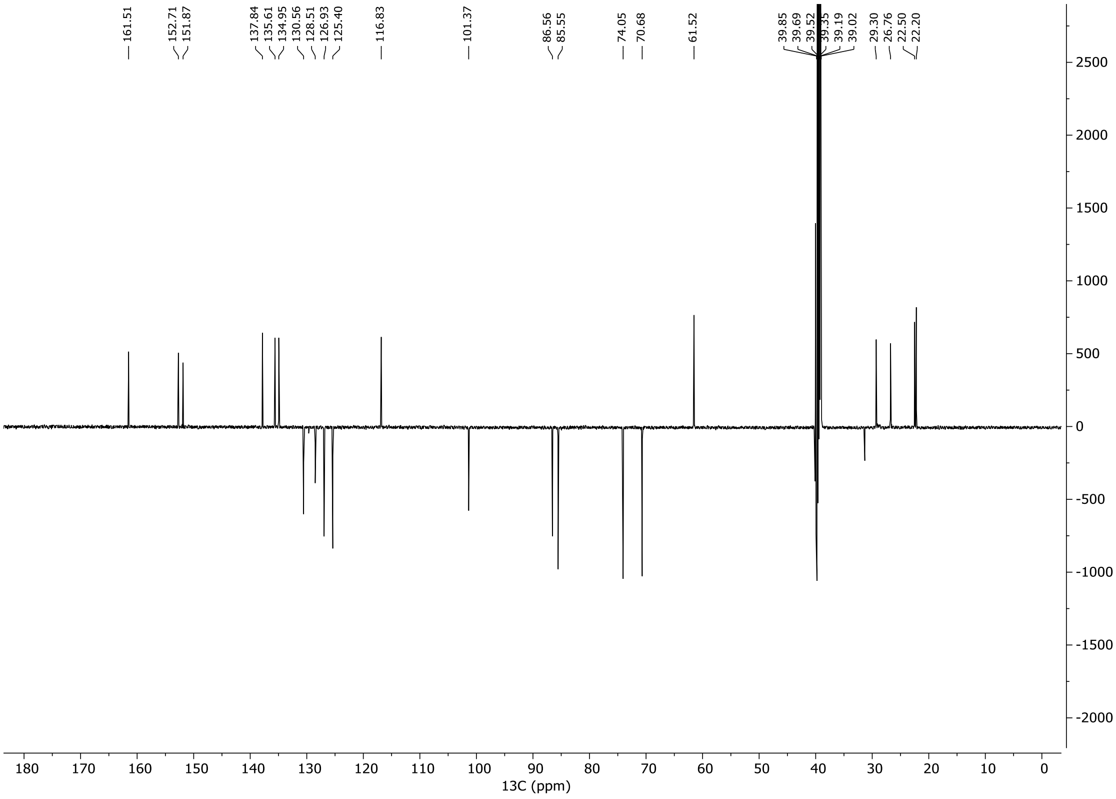

NMR spectra of compound **6B.23**

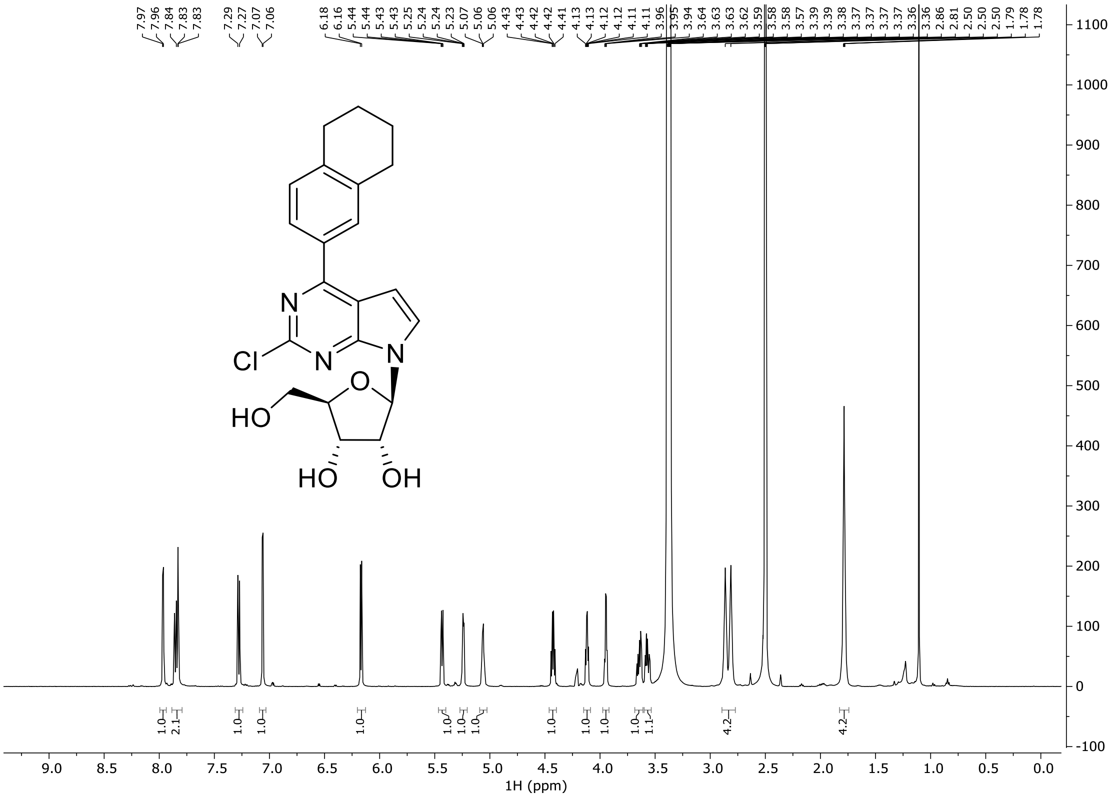

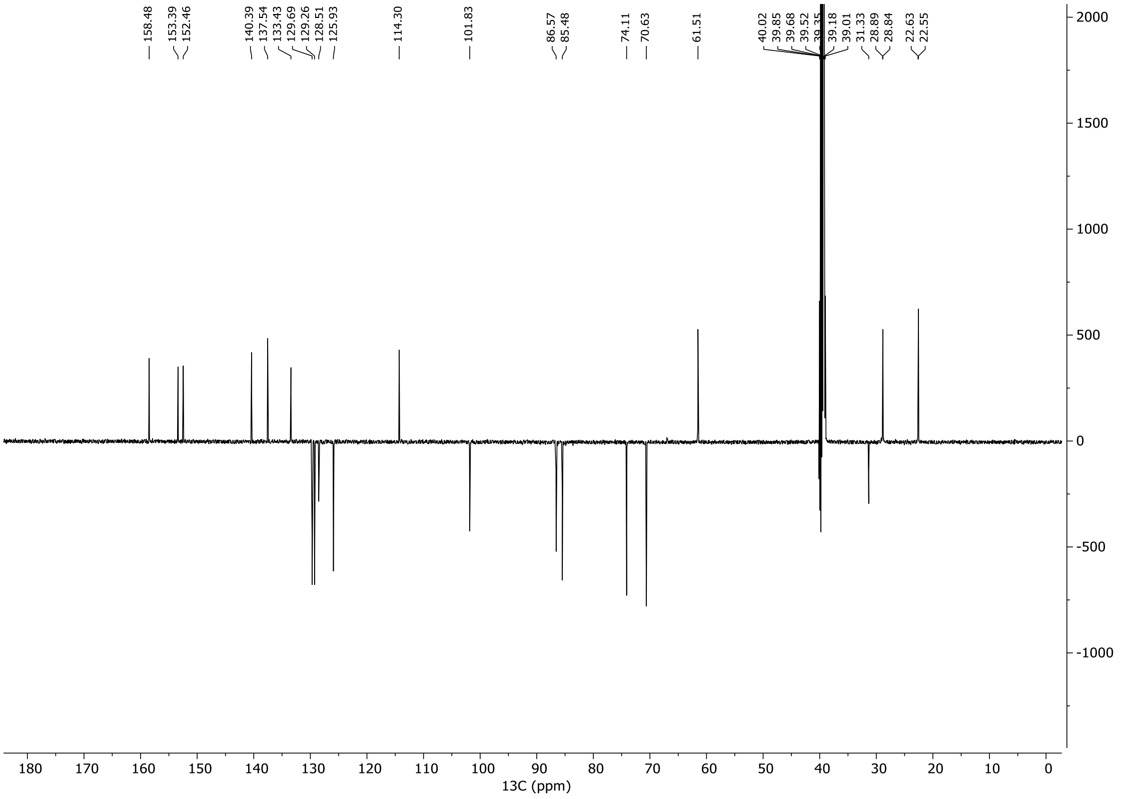

NMR spectra of compound **6B.24**

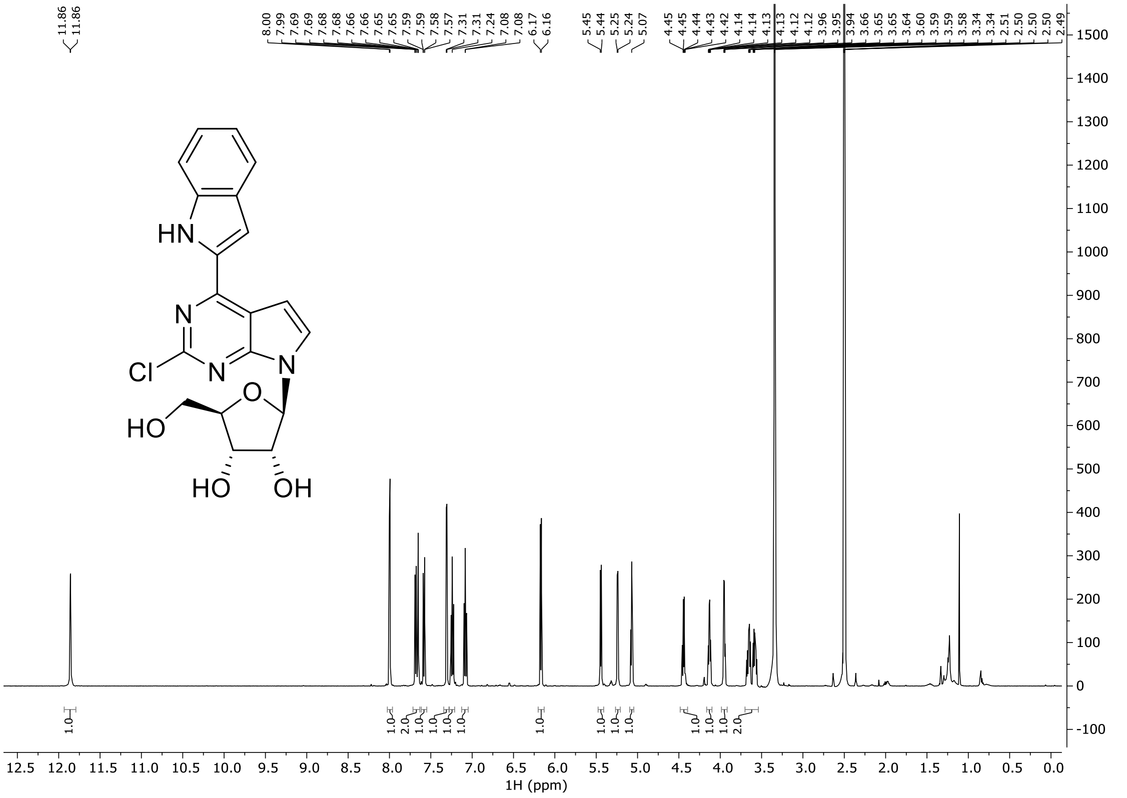

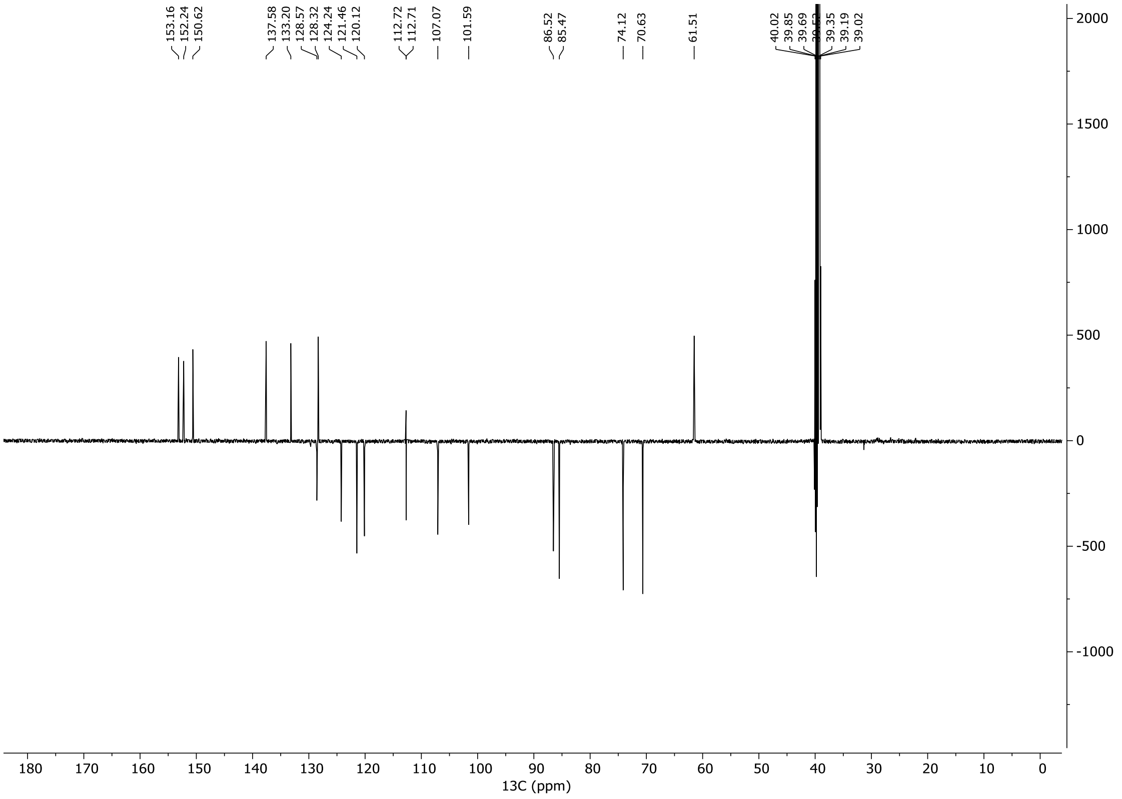

NMR spectra of compound **6B.25**

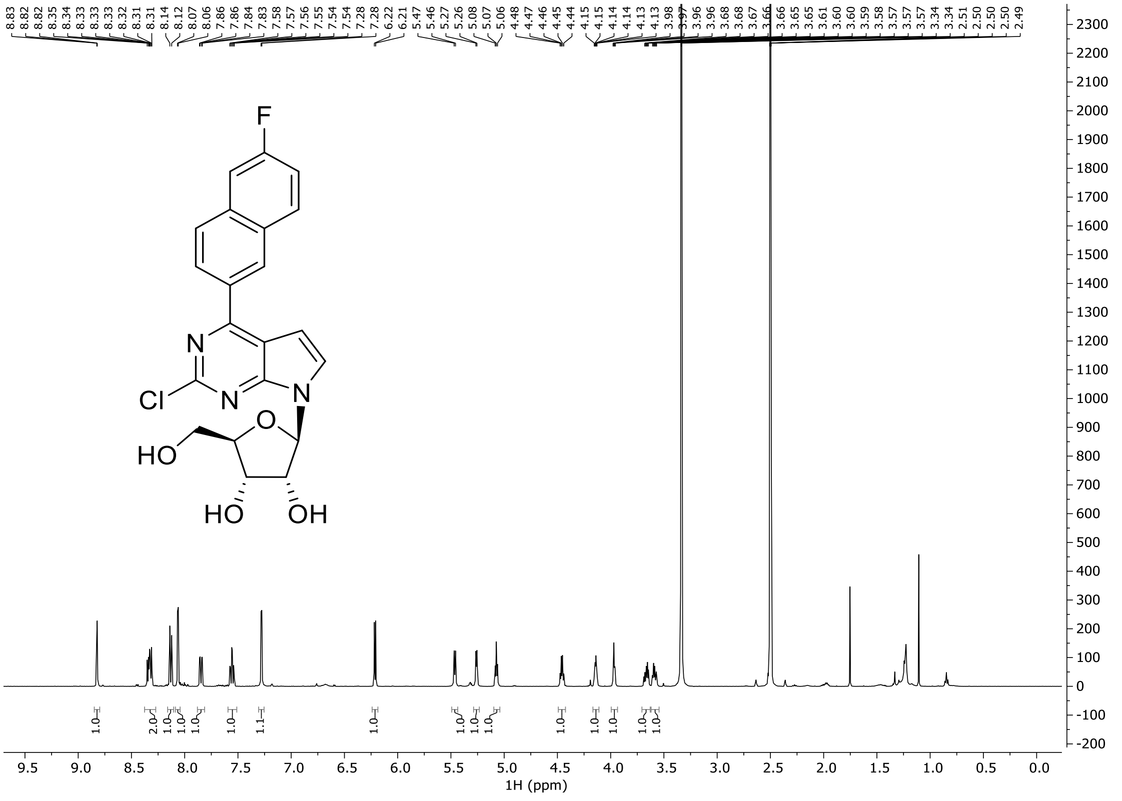

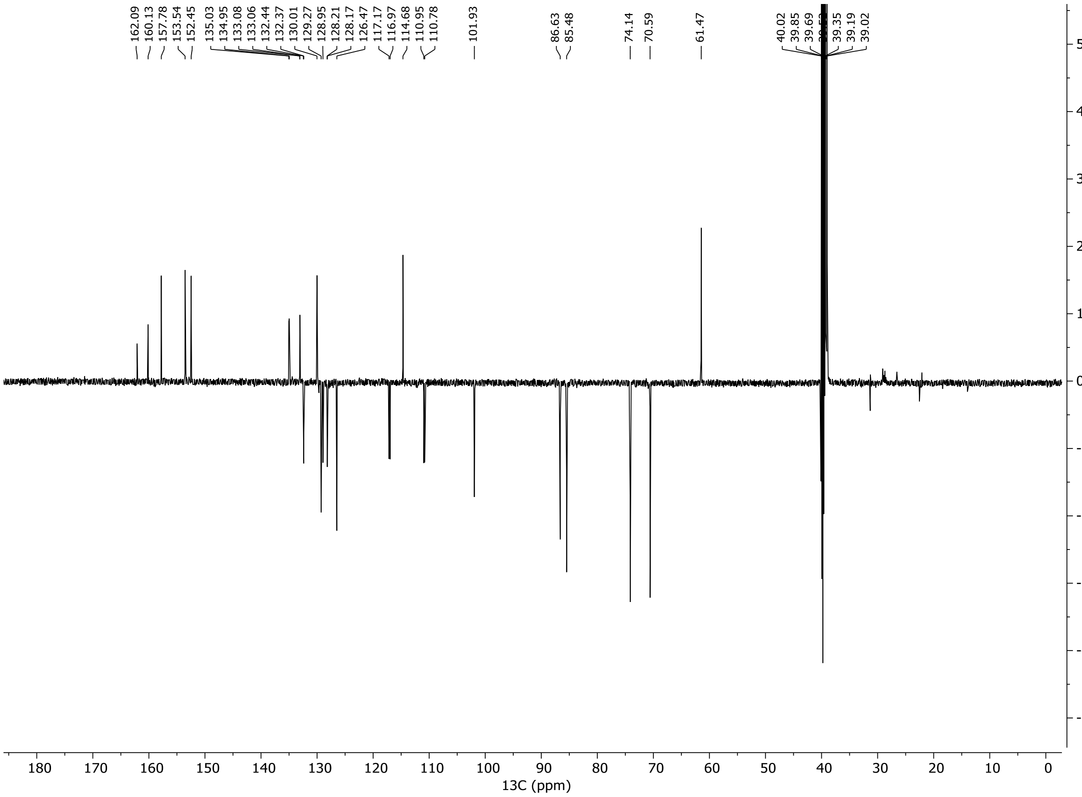

NMR spectra of compound **6B.26**

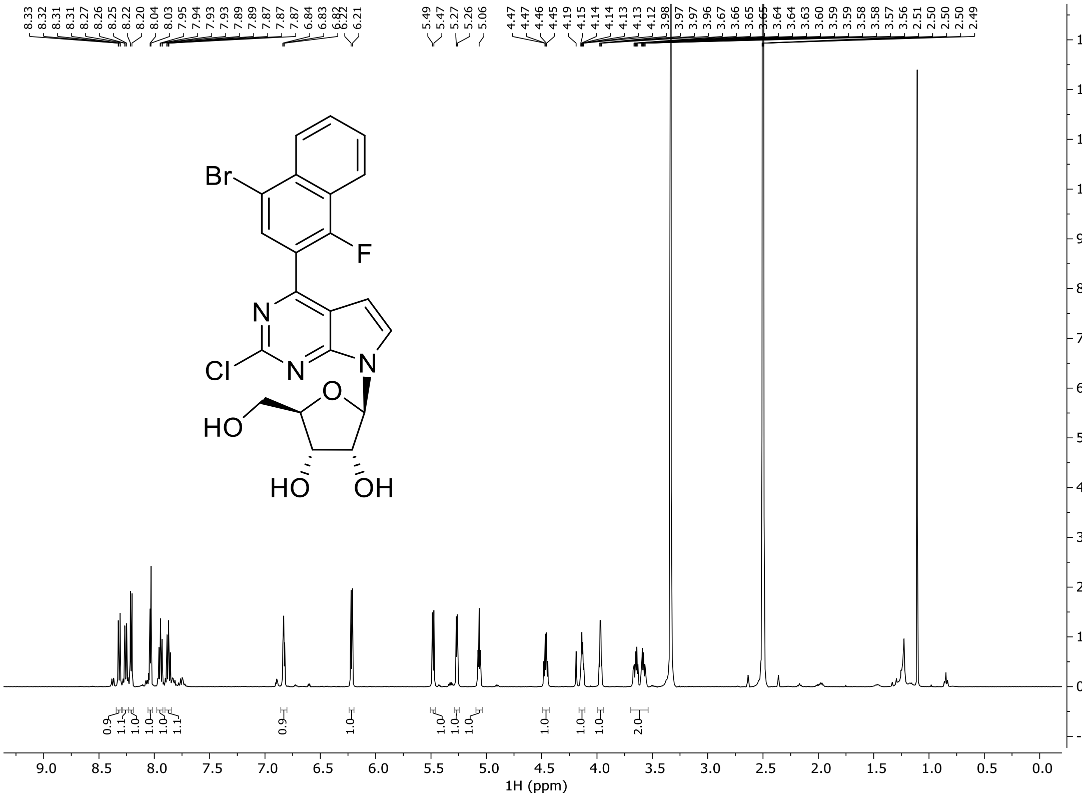

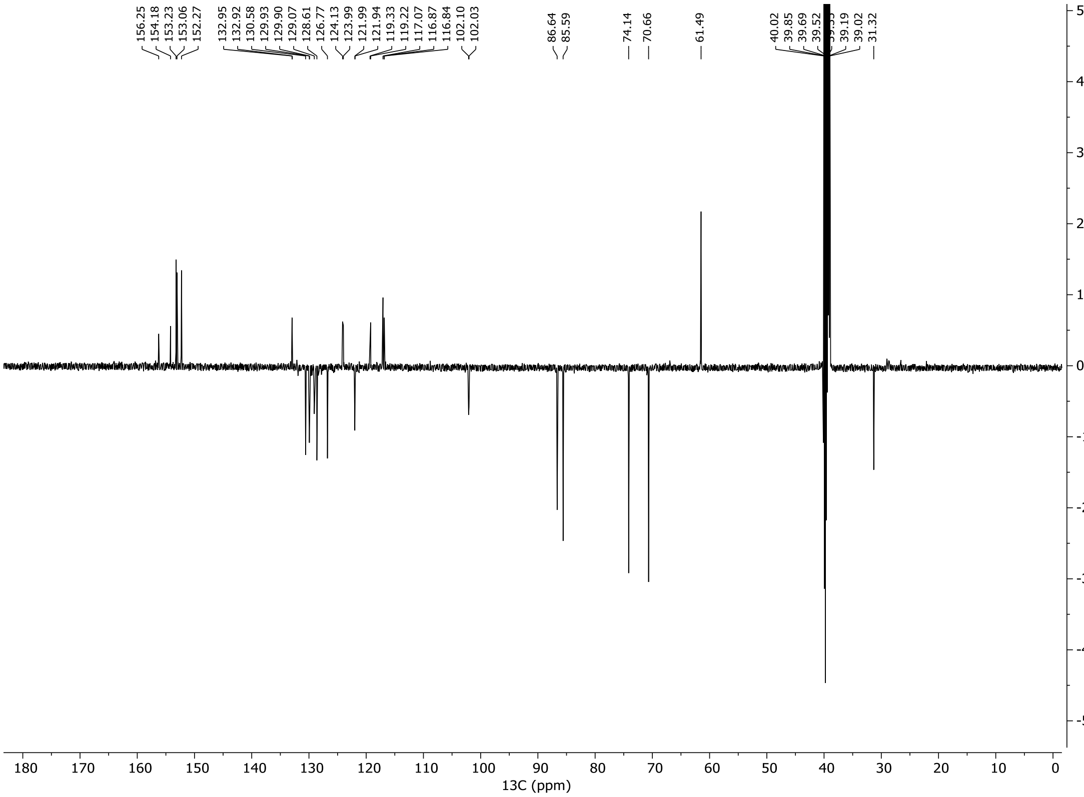

NMR spectra of compound **6B.27**

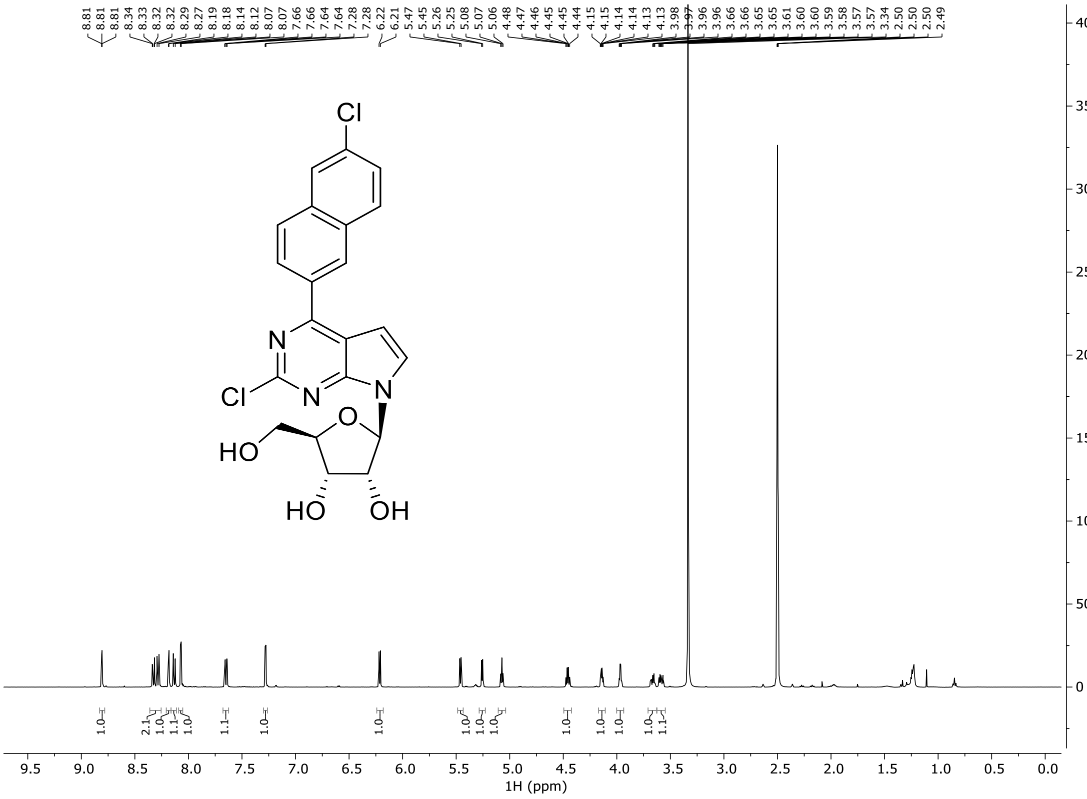

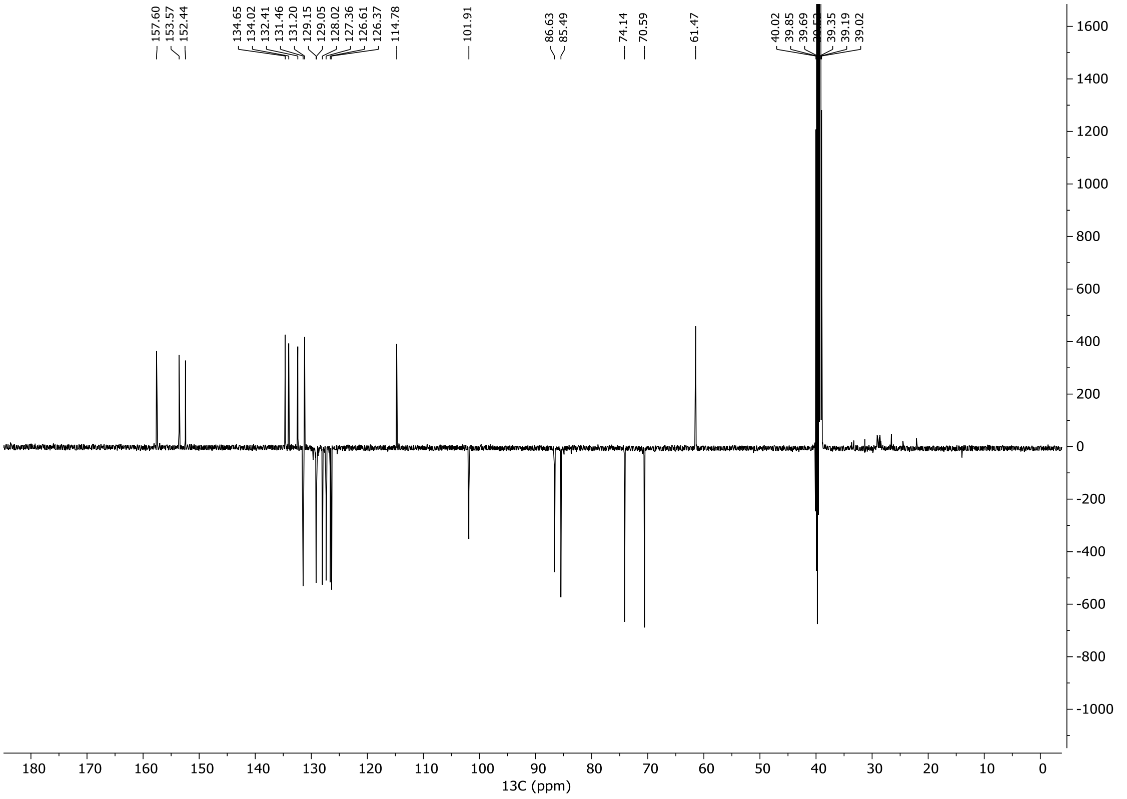

NMR spectra of compound **6B.28**

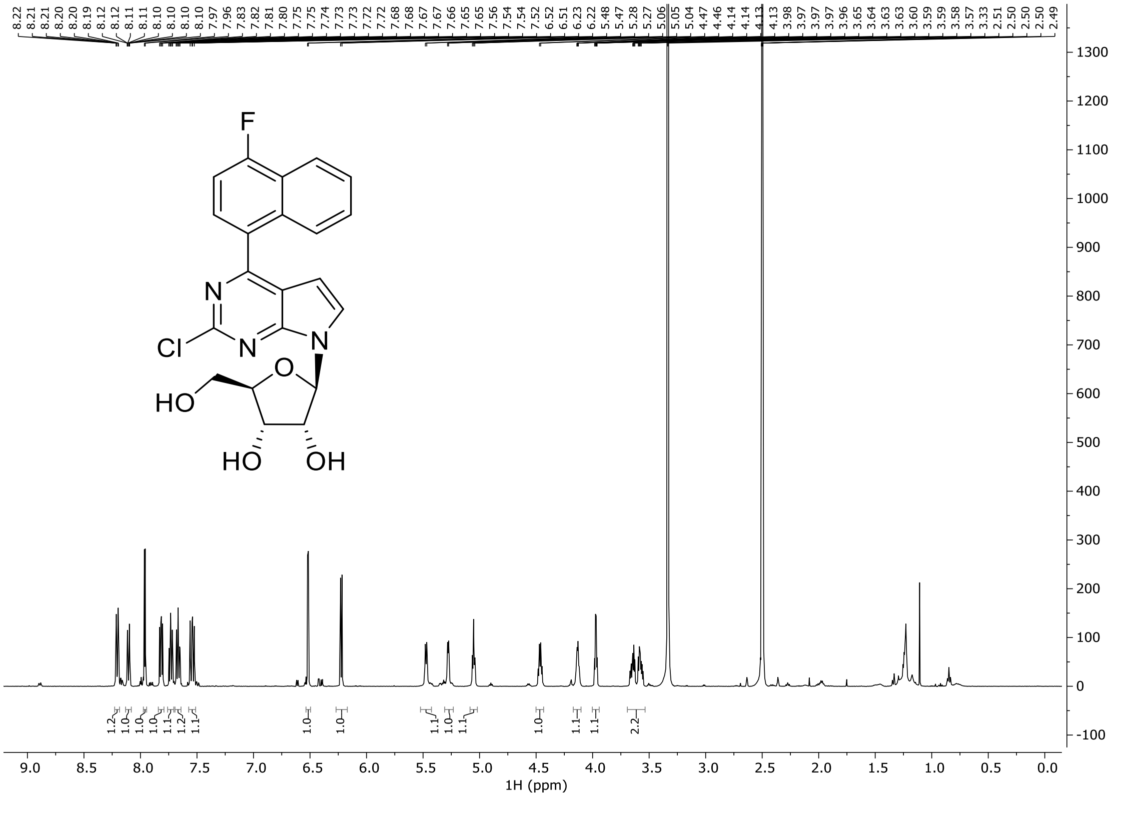

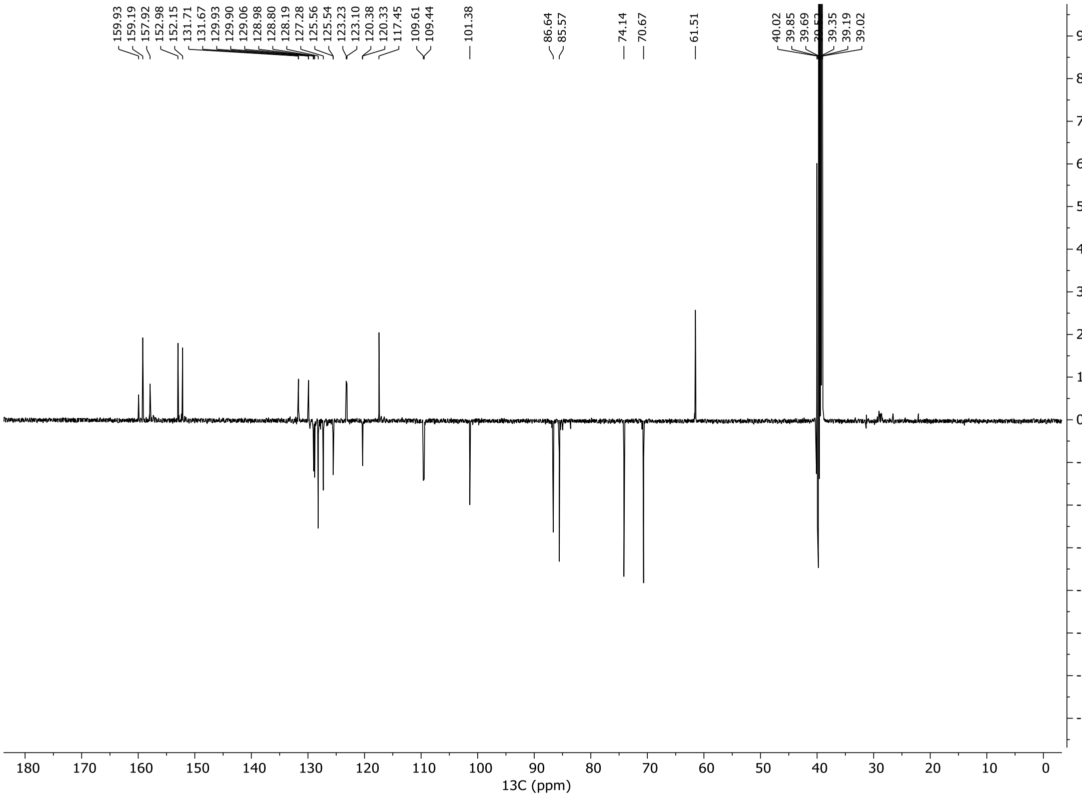

NMR spectra of compound **6B.29**

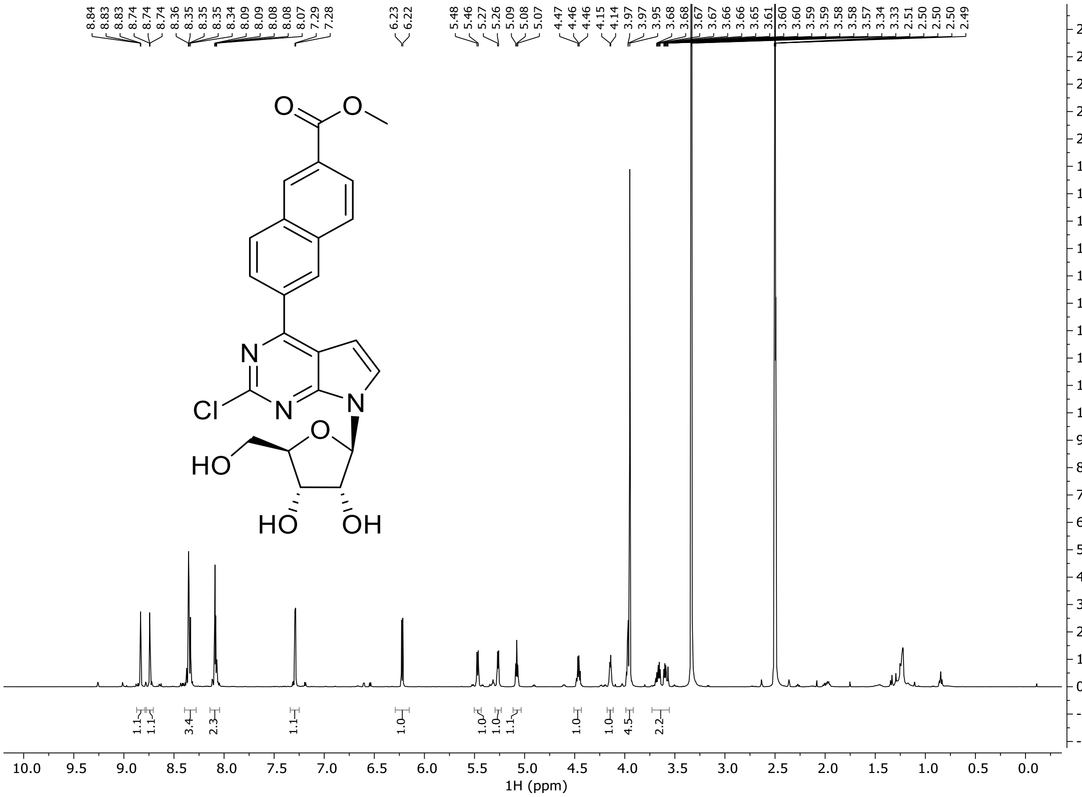

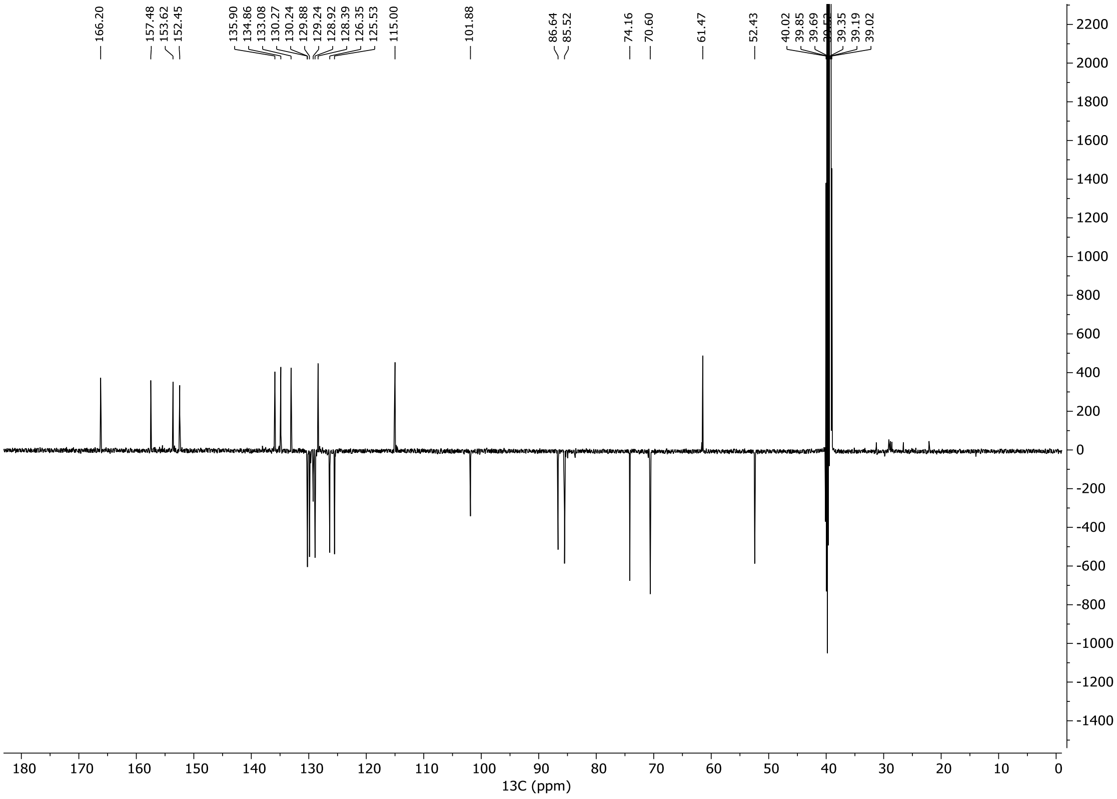

NMR spectra of compound **6B.30**

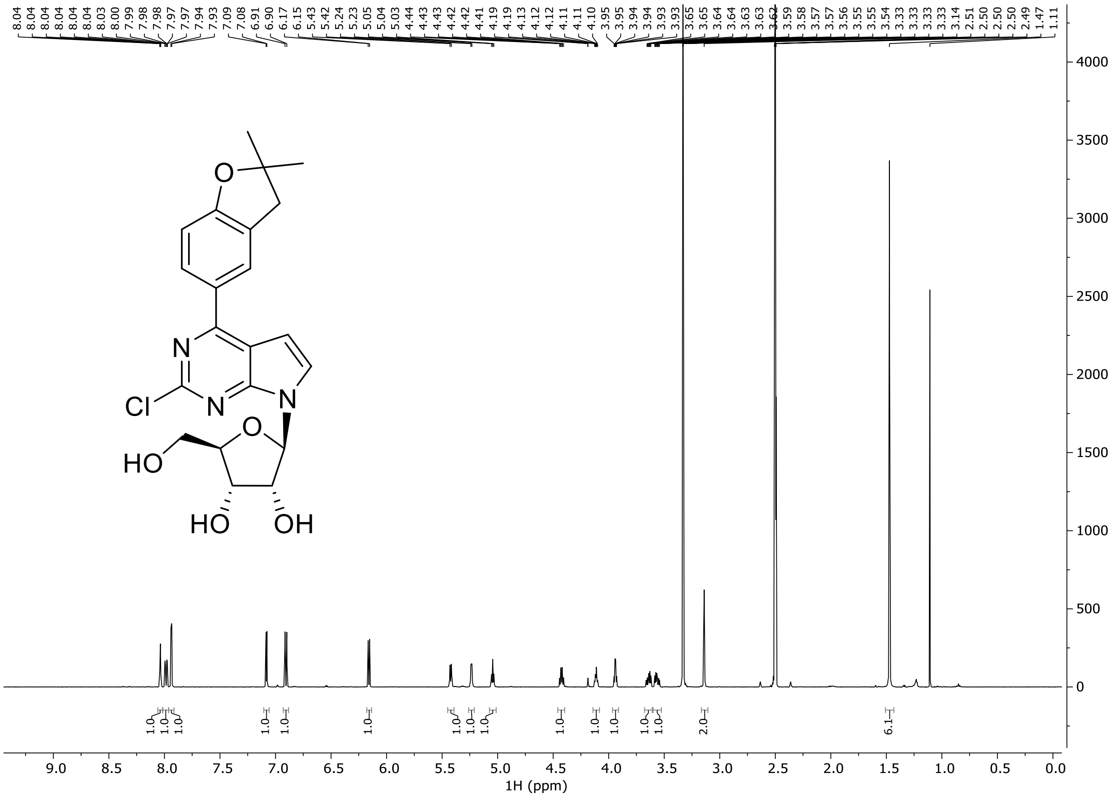

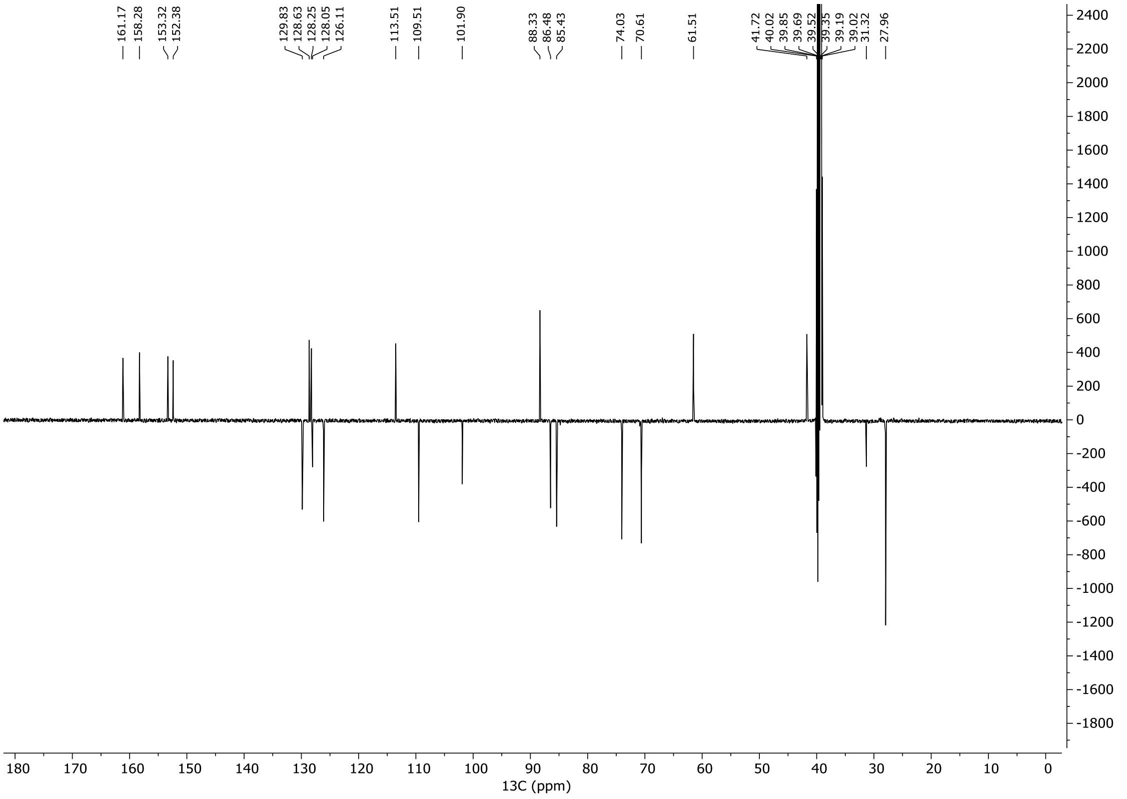

NMR spectra of compound **6B.31**

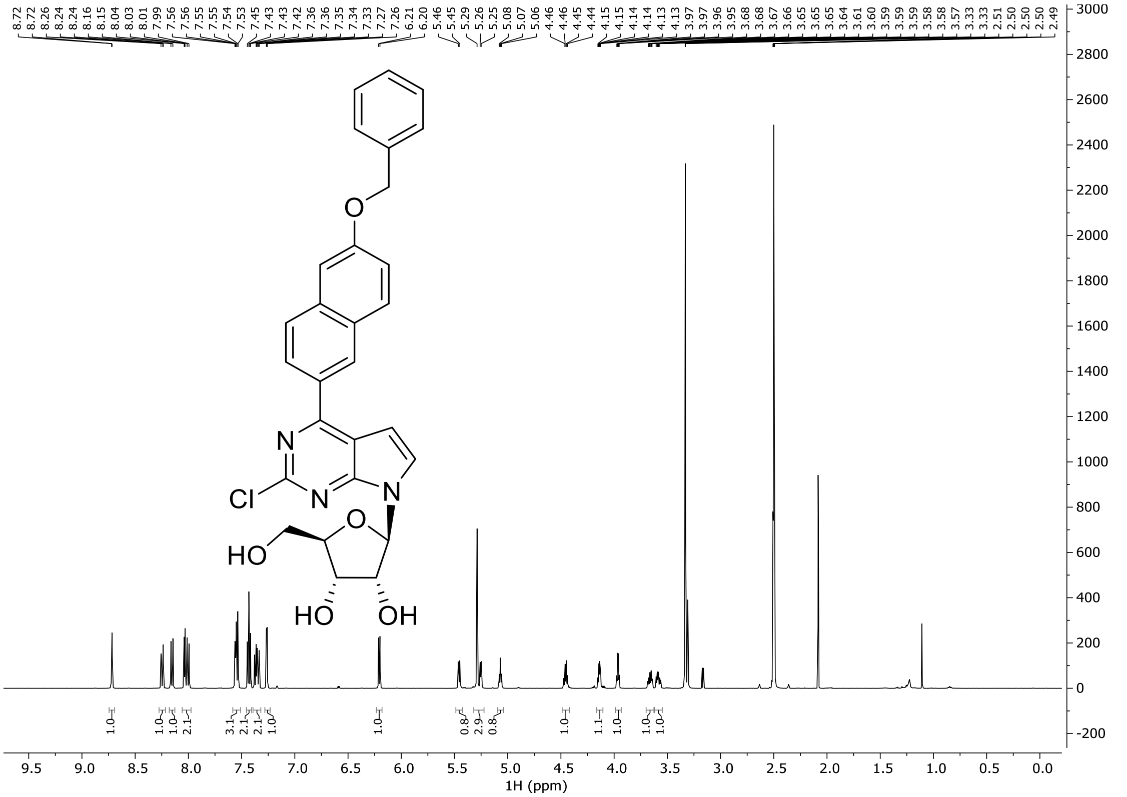

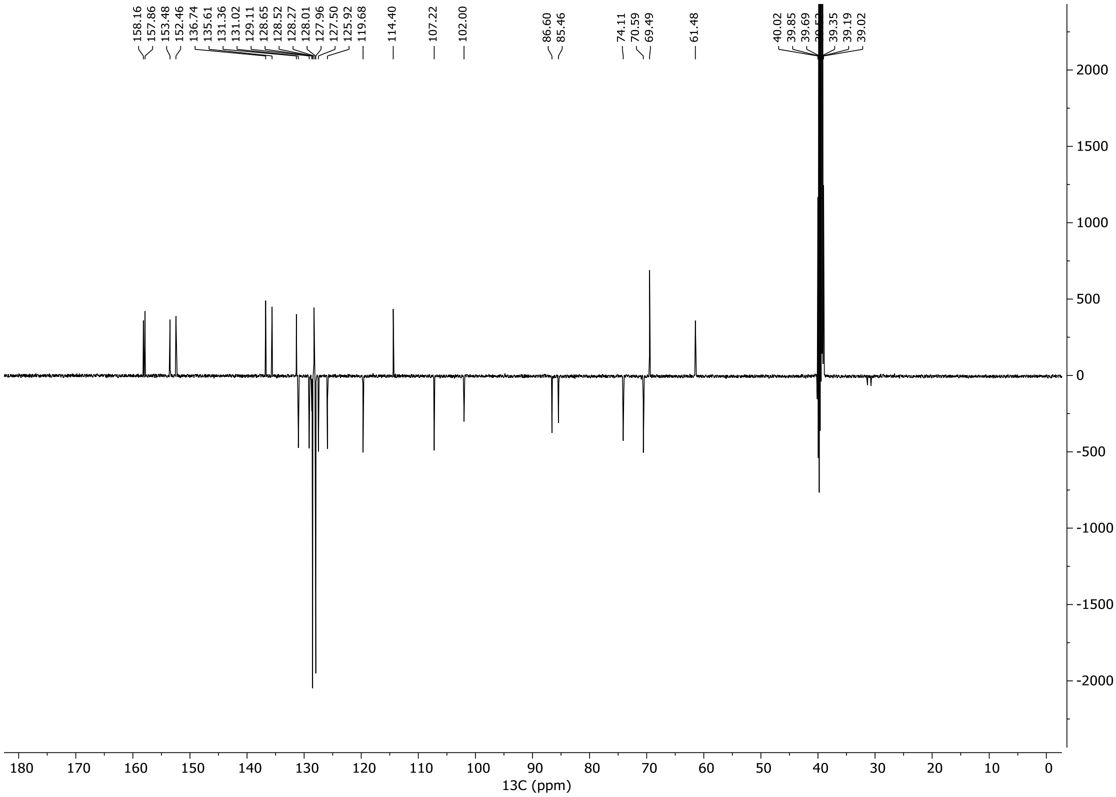

NMR spectra of compound **6B.32**

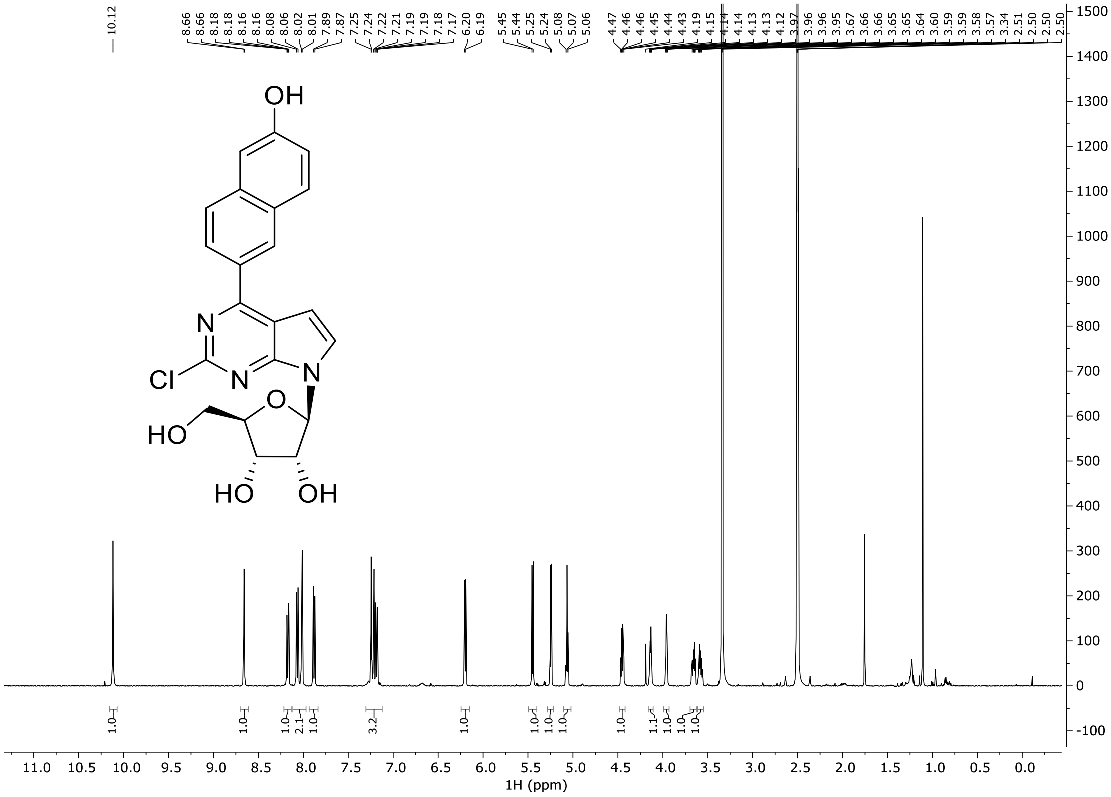

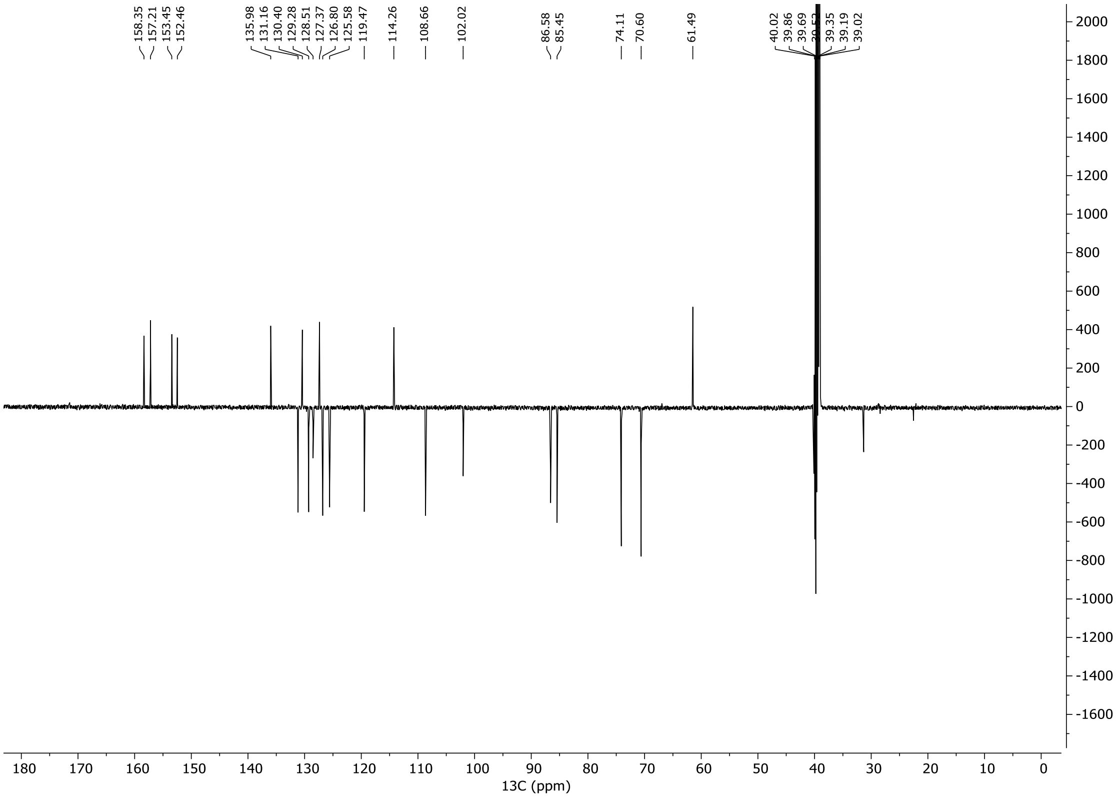

NMR spectra of compound **6B.33**

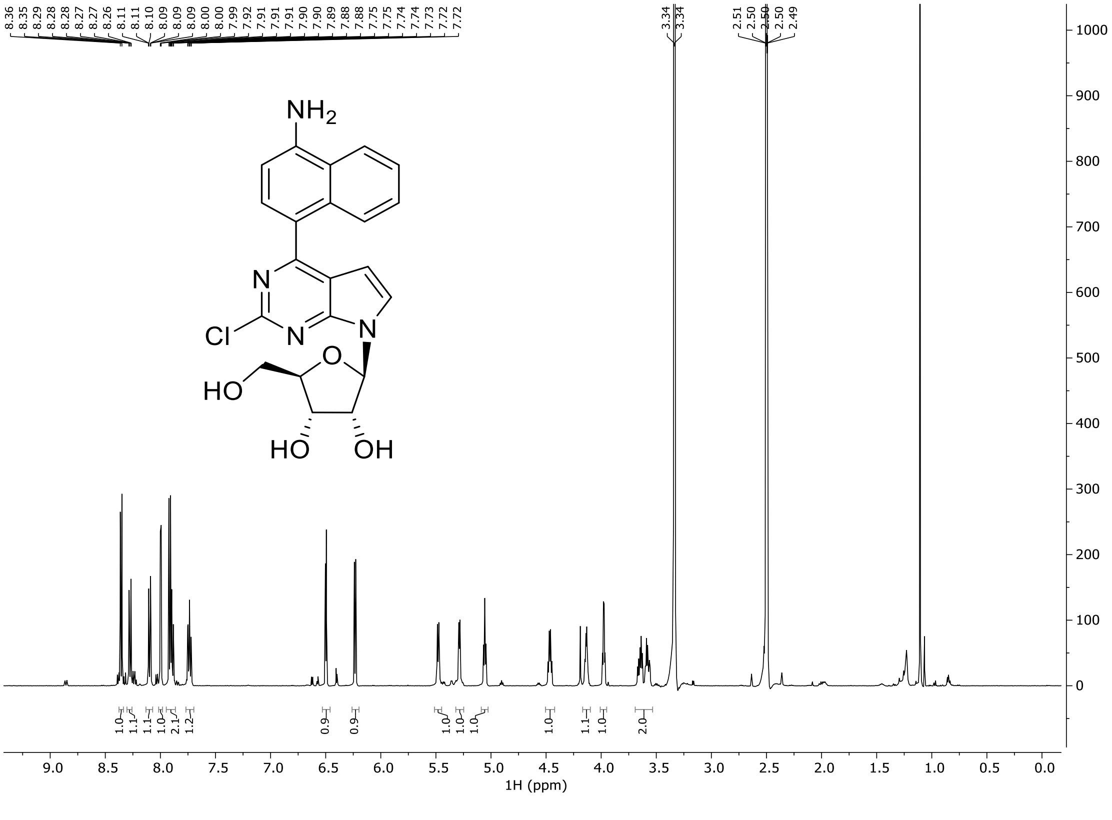

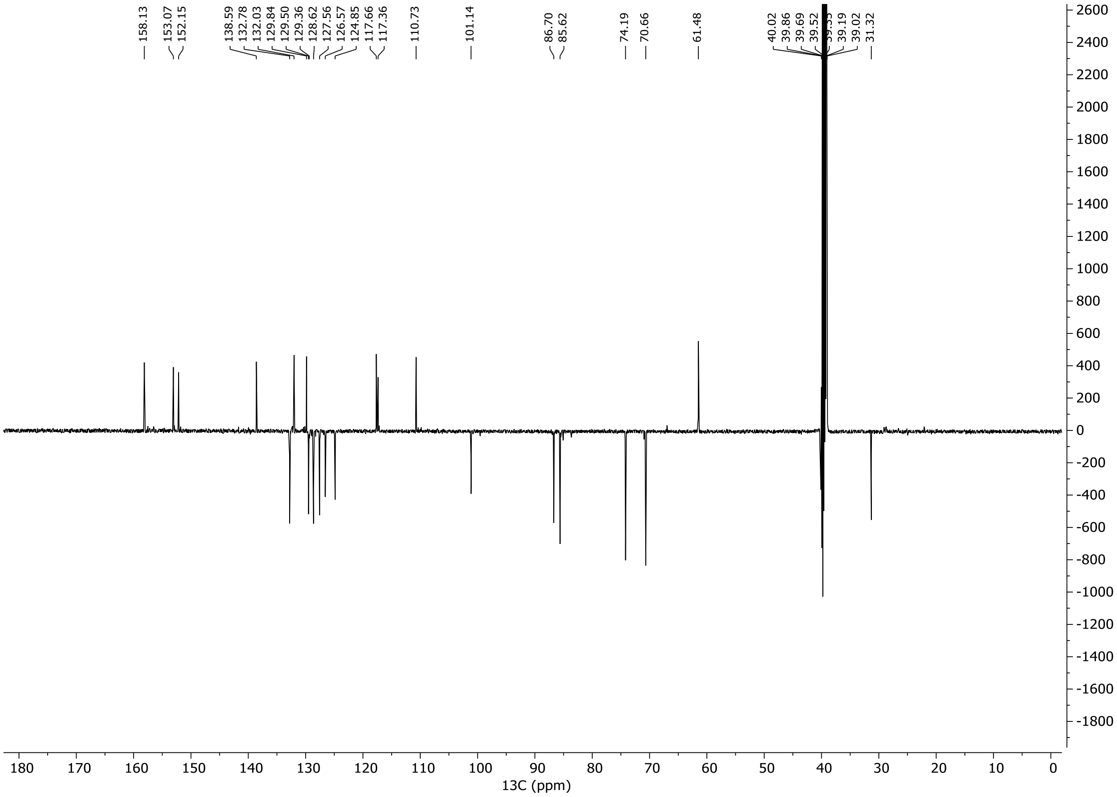

NMR spectra of compound **6B.34**

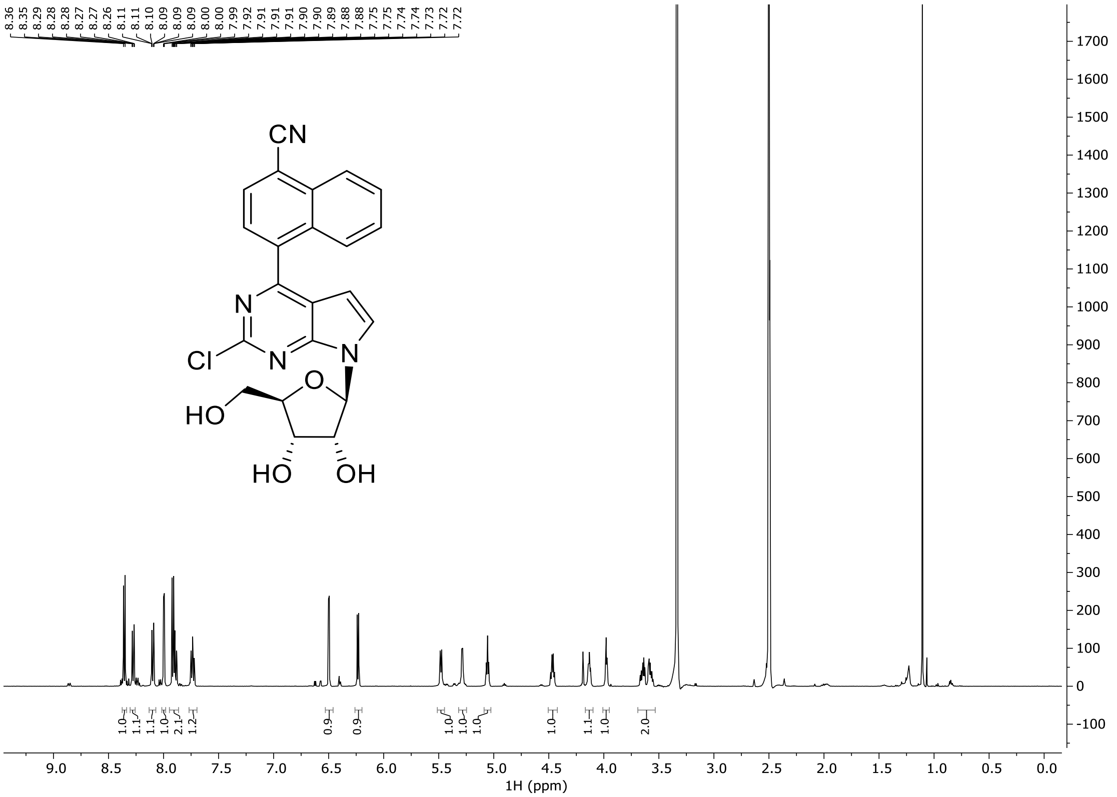

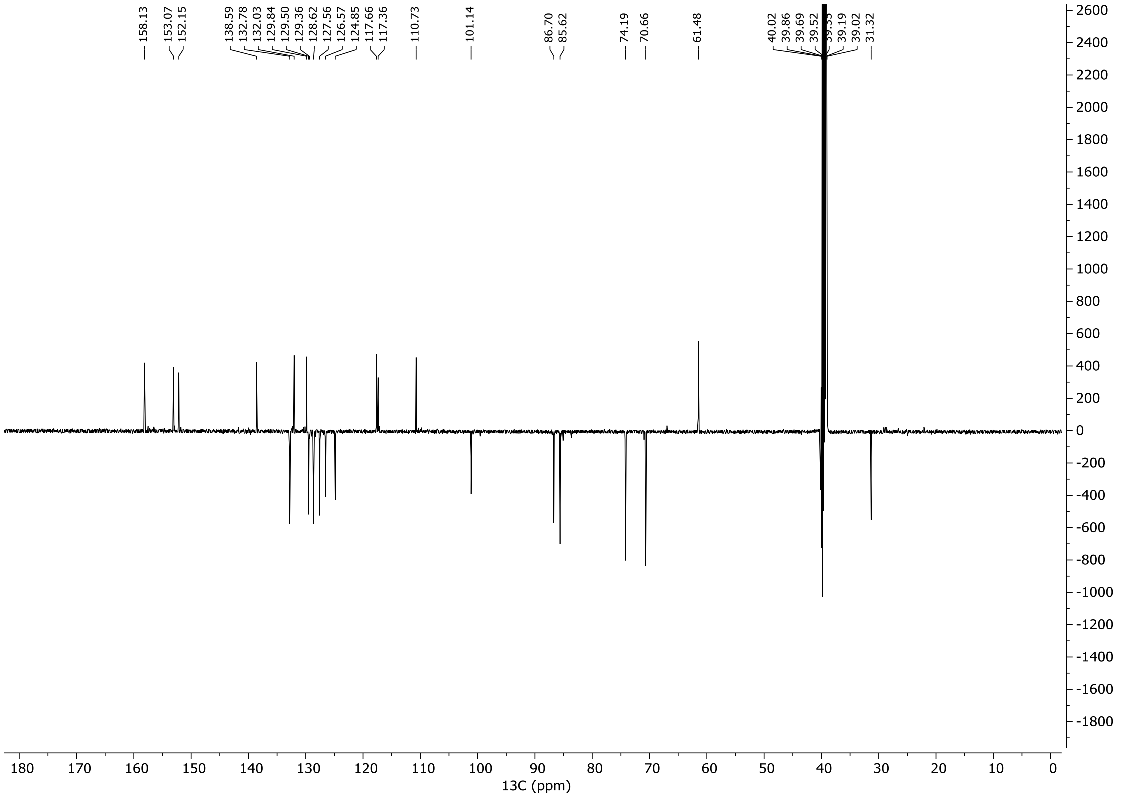

NMR spectra of compound **6B.35**

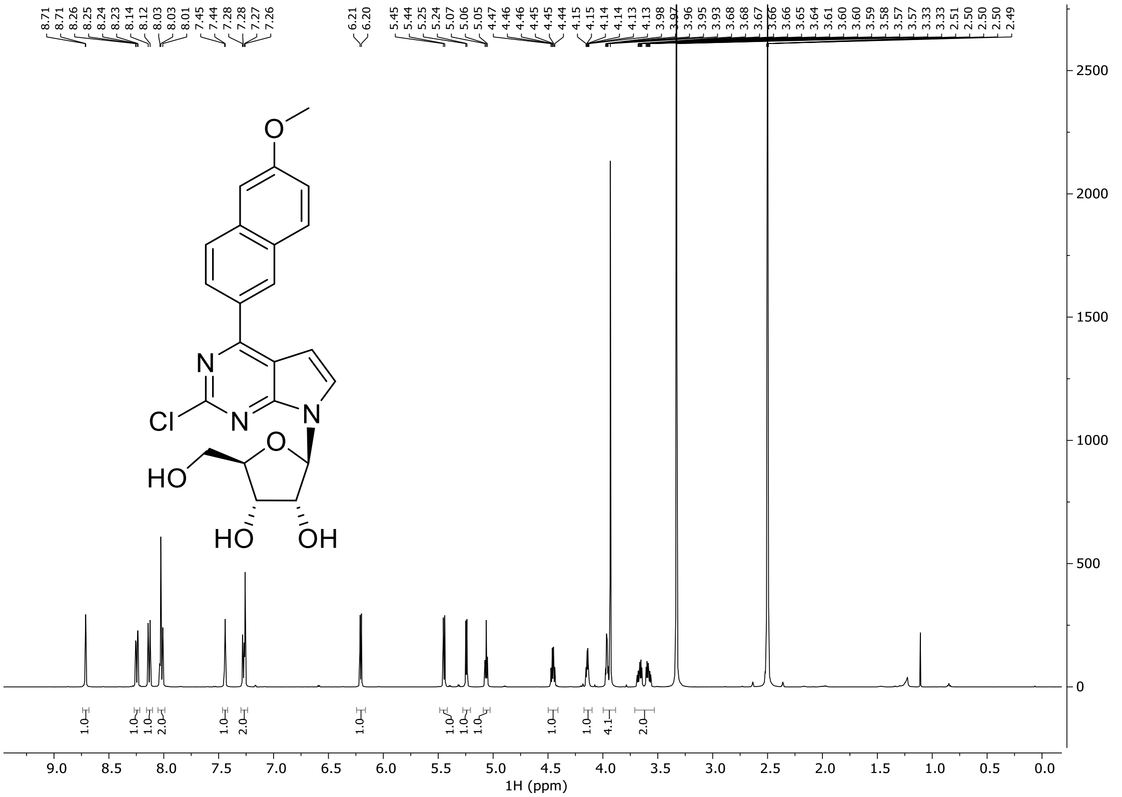

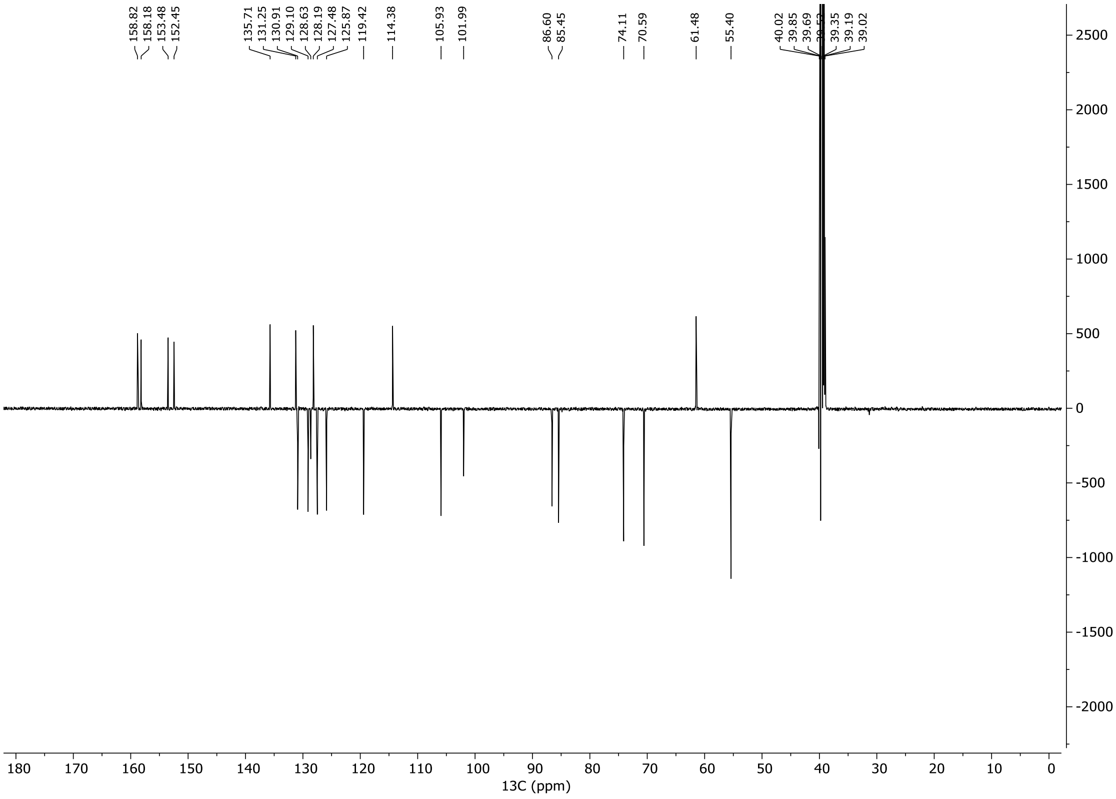

NMR spectra of compound **7B.1**

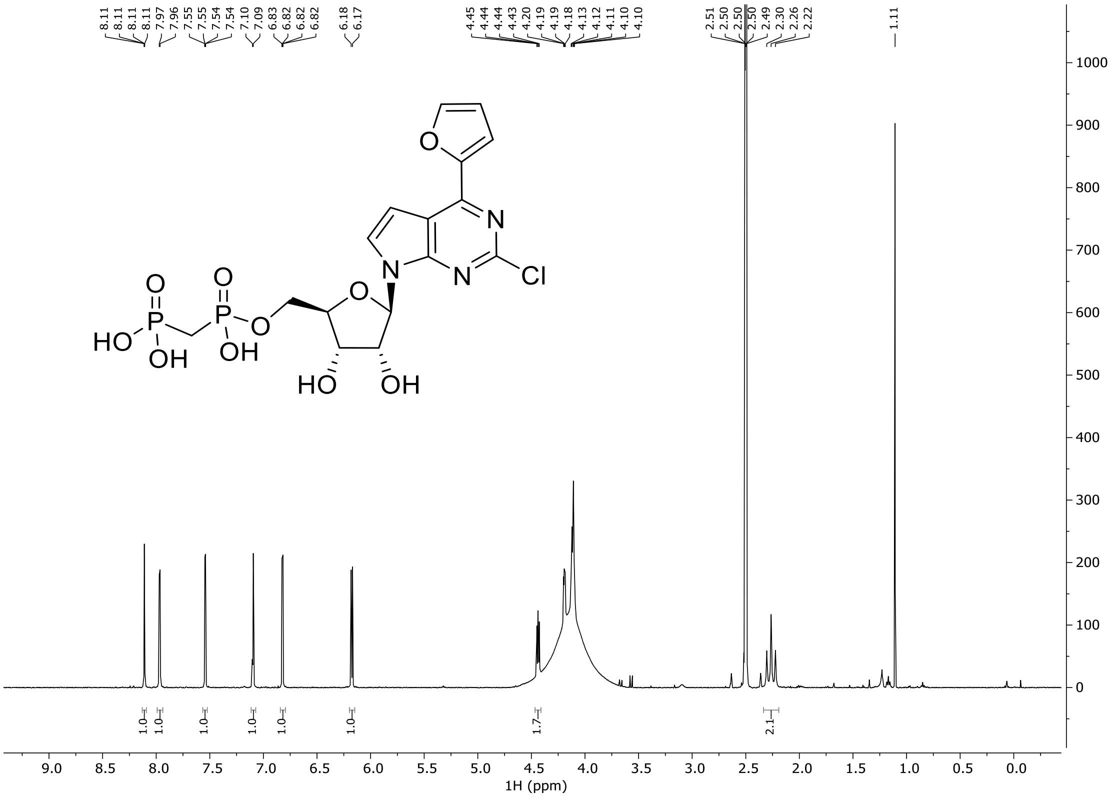

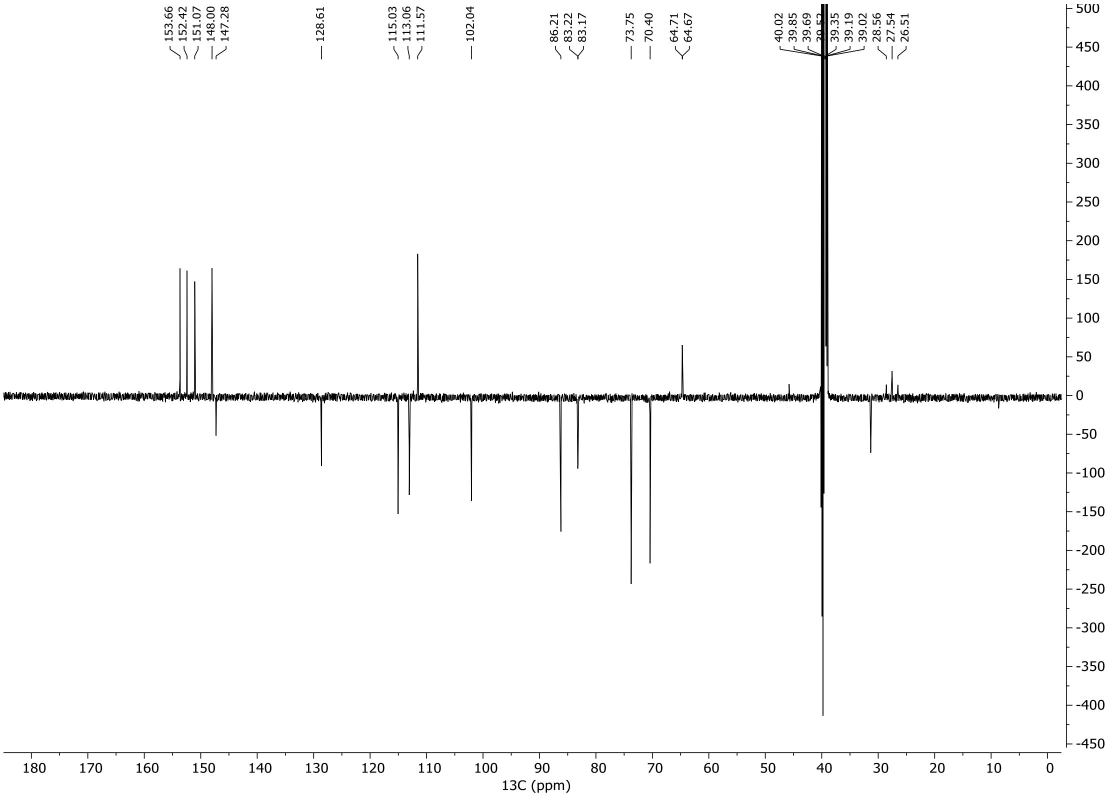

153.66
152.42
151.07
148.00
147.28
128.61
115.03
113.06
111.57
102.04
86.21
83.22
83.17
73.75
70.40
64.71
64.67
40.02
39.85
39.69
39.35
39.19
39.02
28.56
27.54
26.51
13C (ppm)

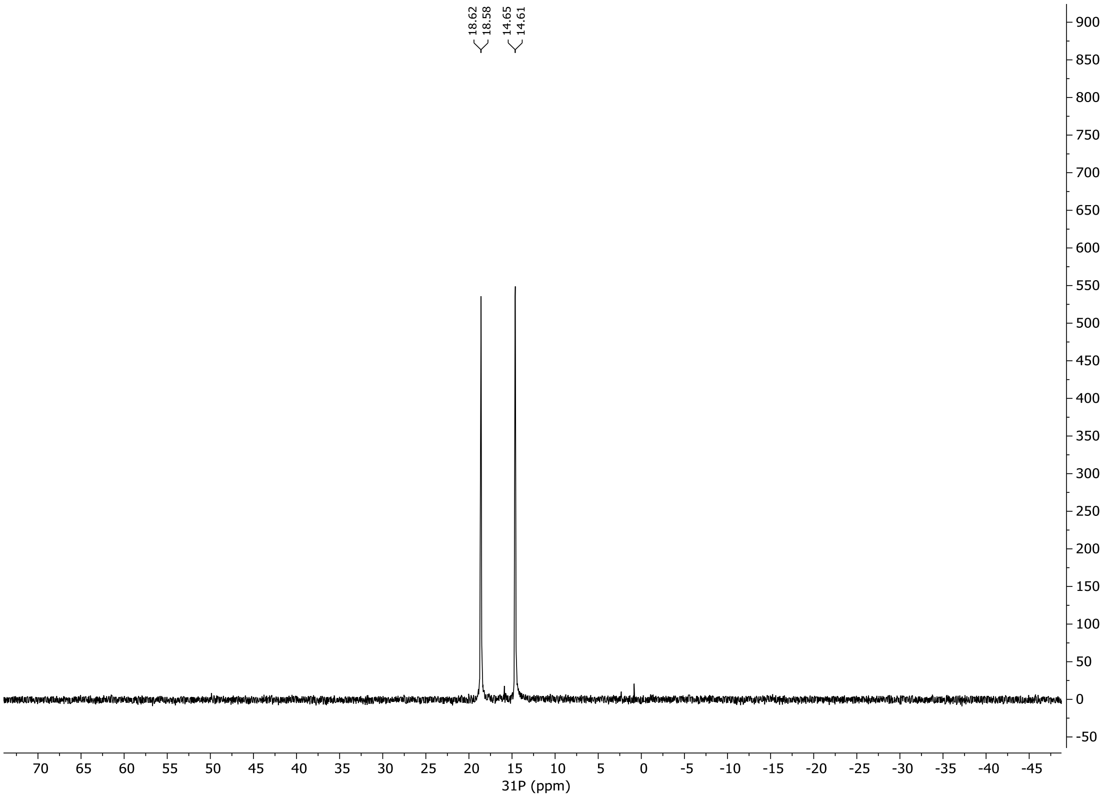

18.62
18.58
14.65
14.61
31P (ppm)

NMR spectra of compound **7B.6**

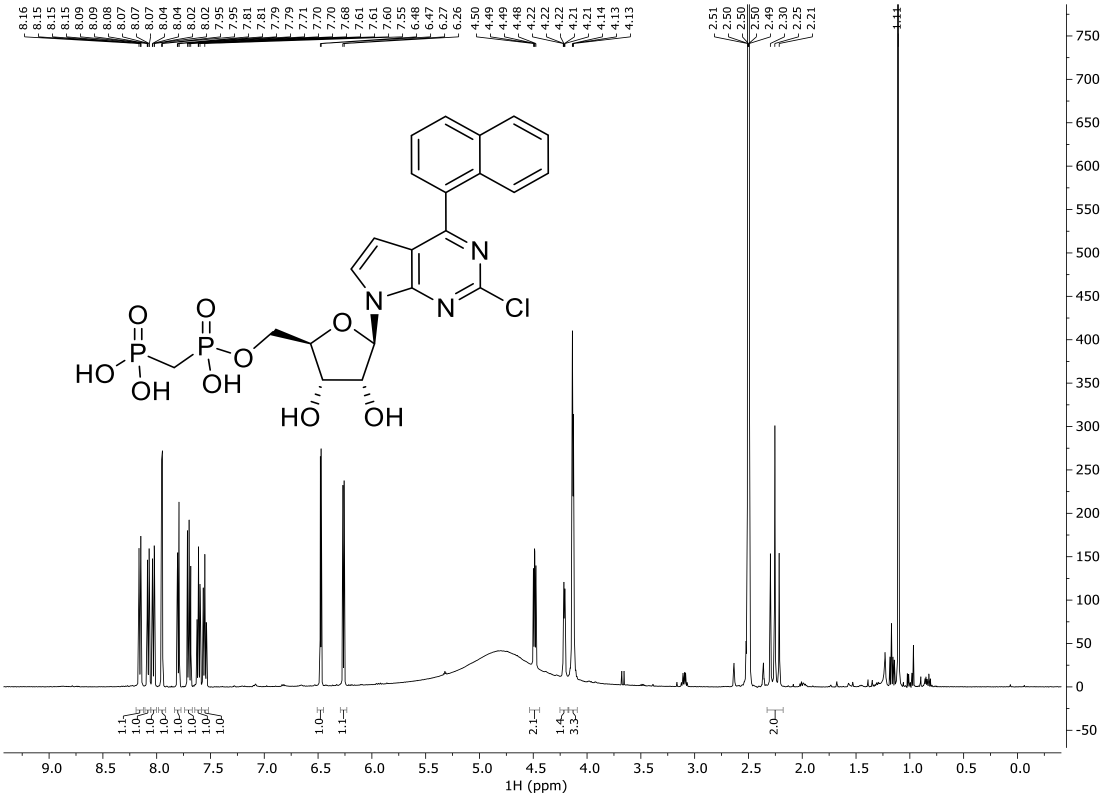

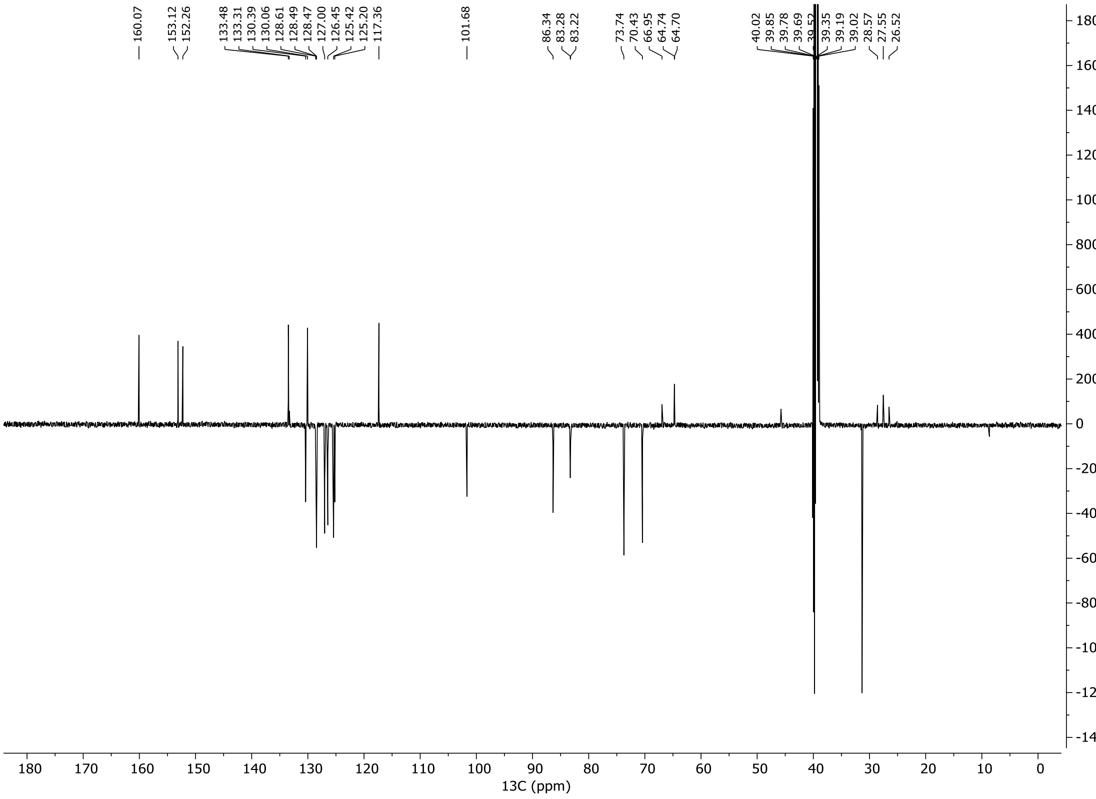

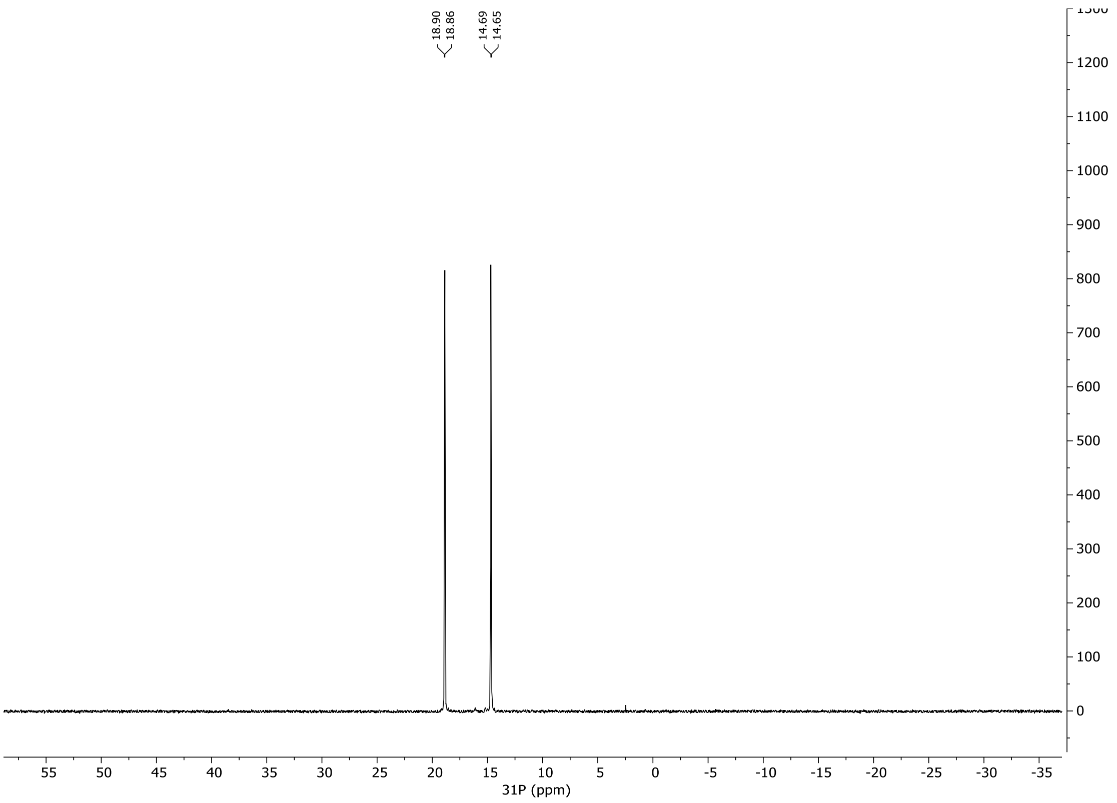

NMR spectra of compound **7B.7**

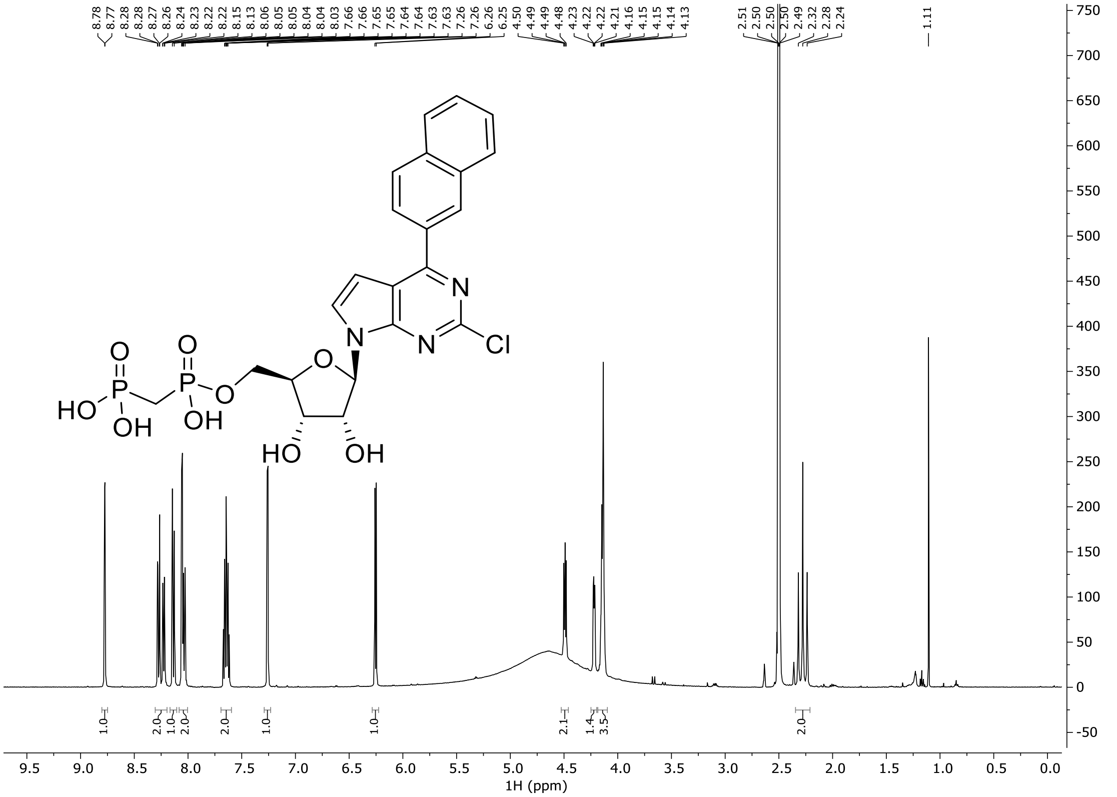

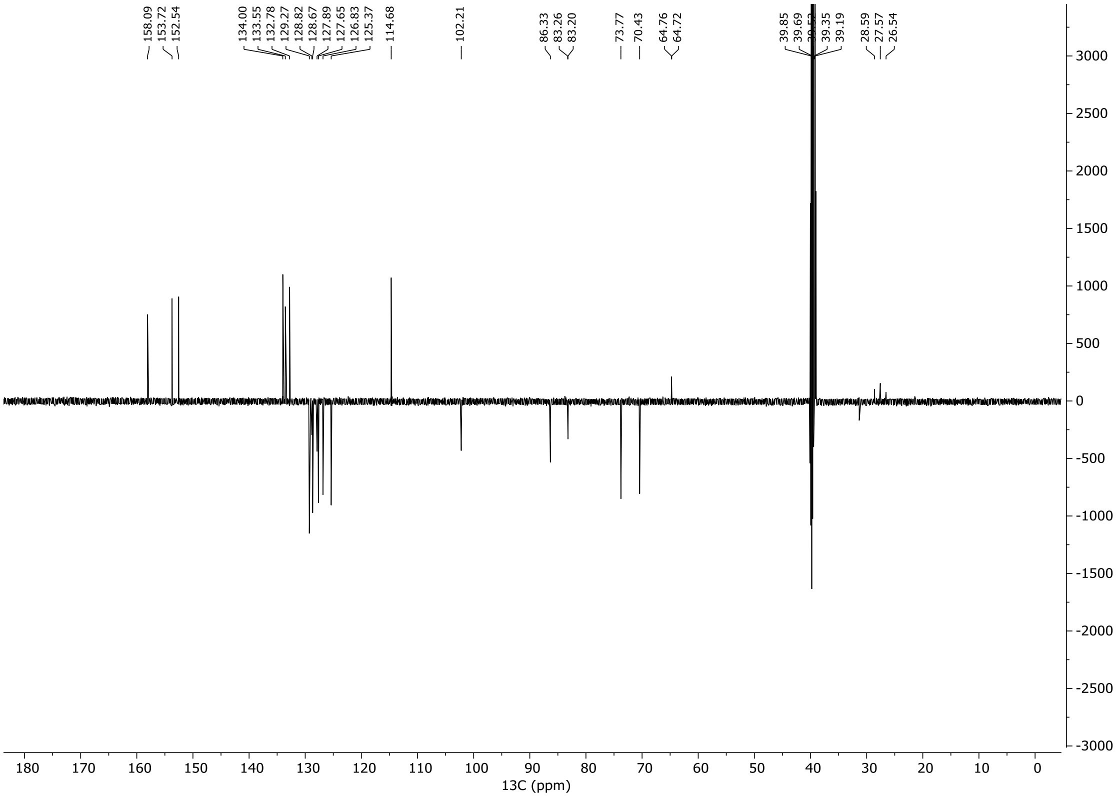
158.09
153.72
152.54
134.00
133.55
132.78
129.27
128.82
128.67
127.89
127.65
126.83
125.37
114.68
102.21
86.33
83.26
83.20
73.77
70.43
64.76
64.72
39.85
39.69
39.35
39.19
28.59
27.57
26.54
13C (ppm)

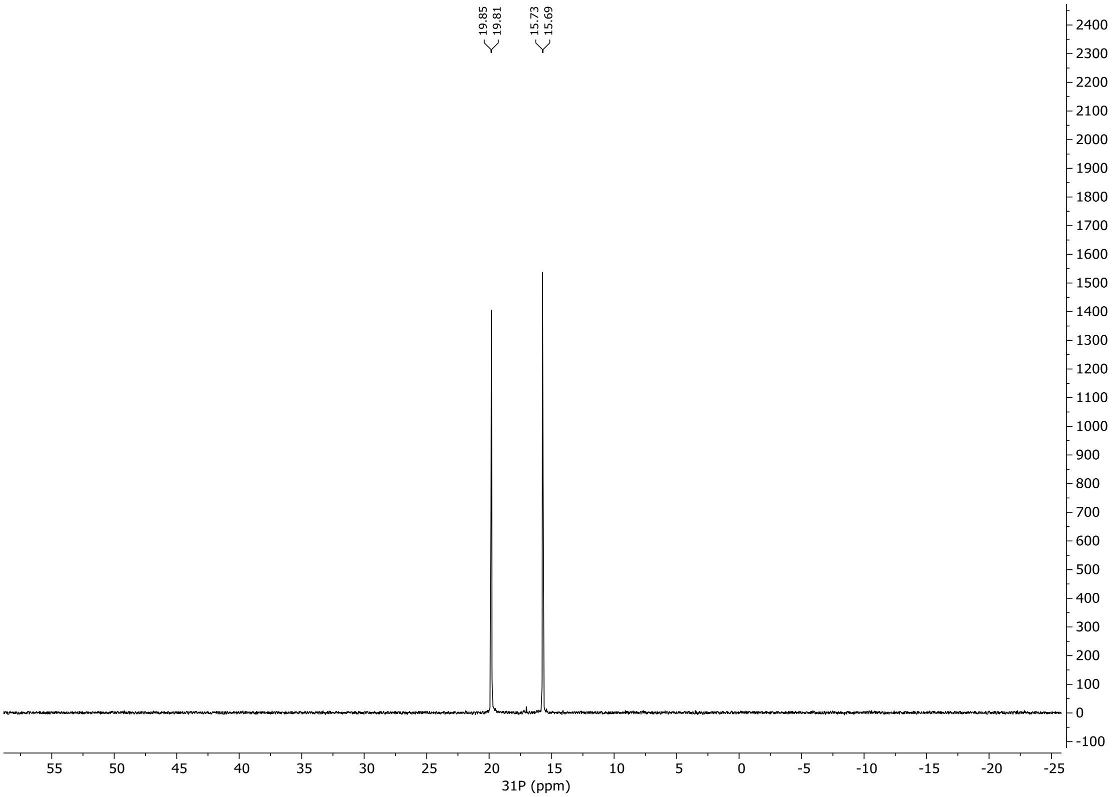
19.85
19.81
15.73
15.69
31P (ppm)

NMR spectra of compound **7B.8**

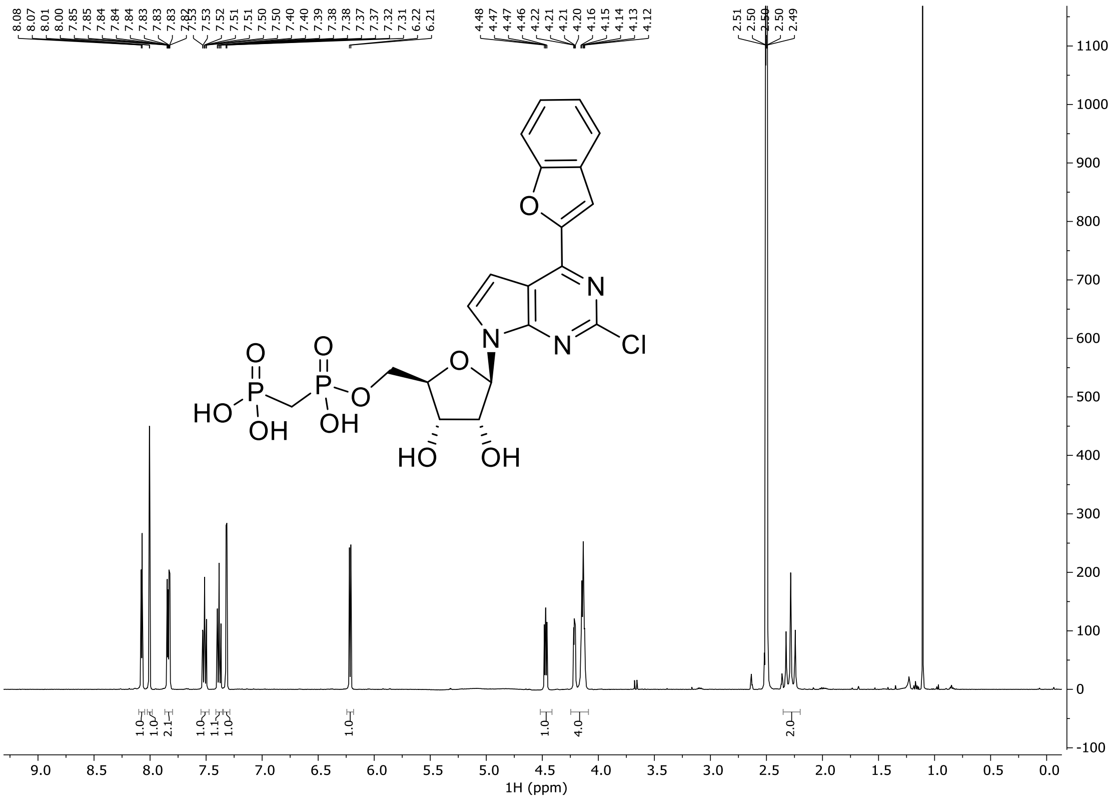

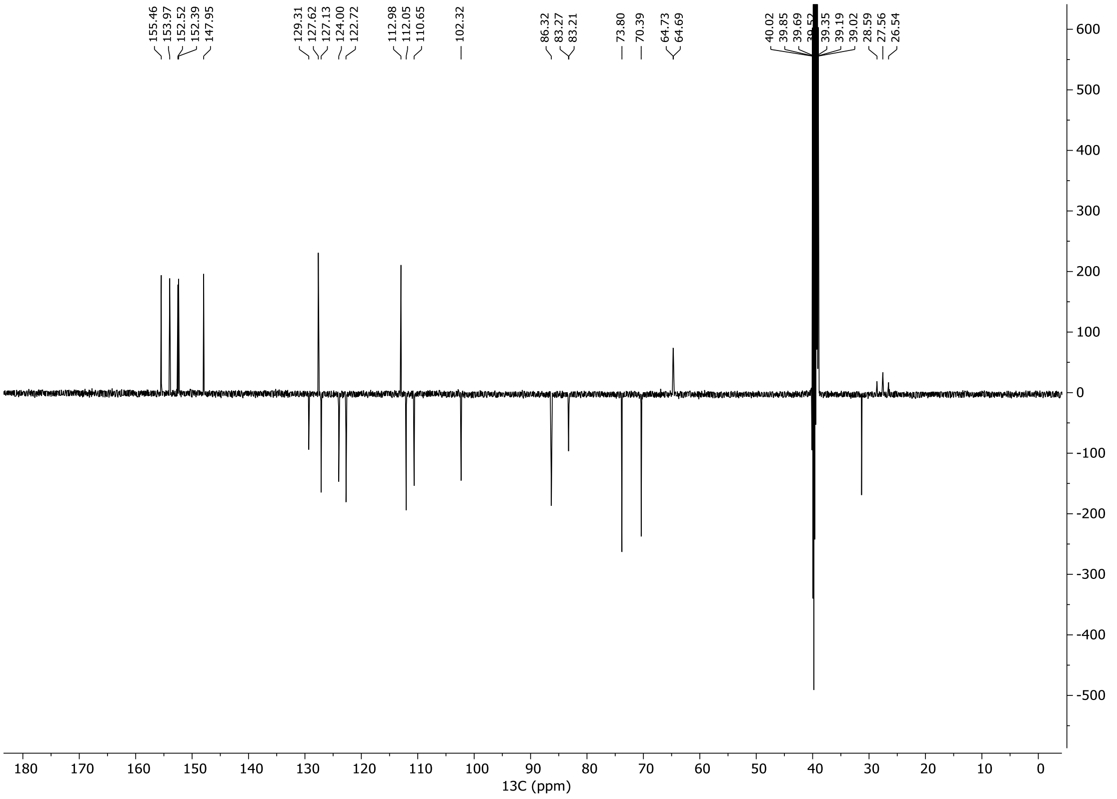

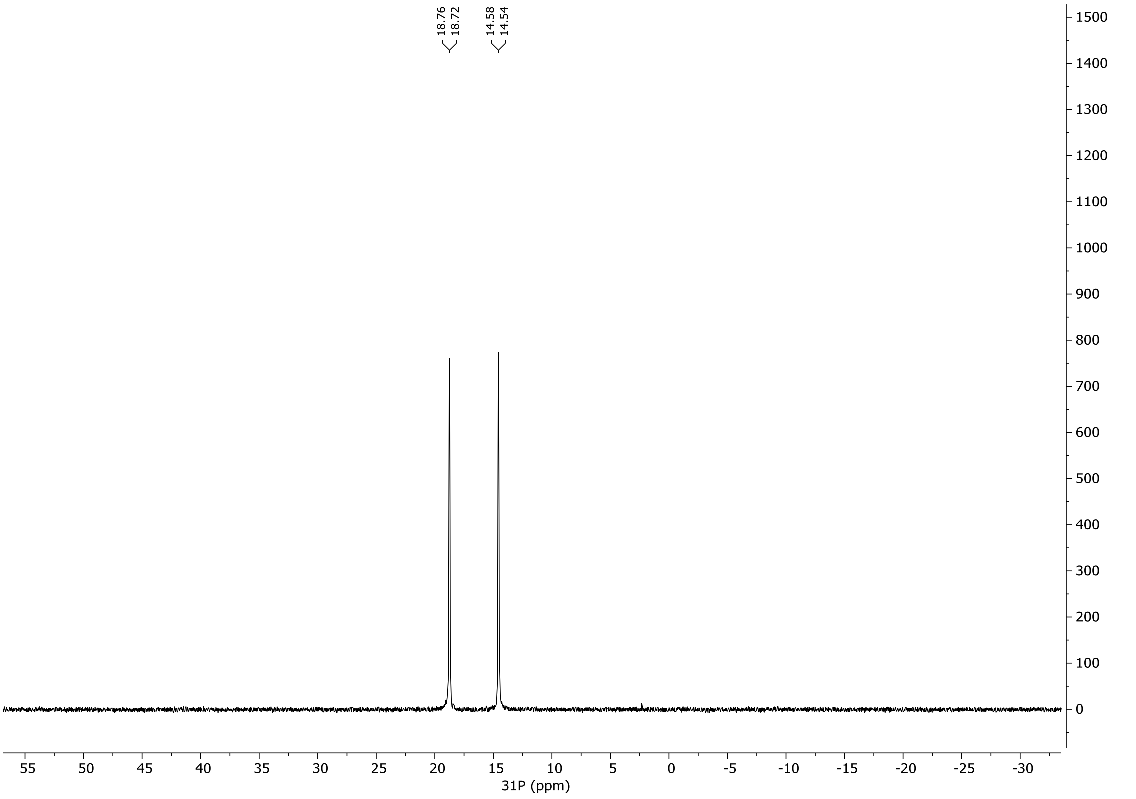

NMR spectra of compound **7B.12**

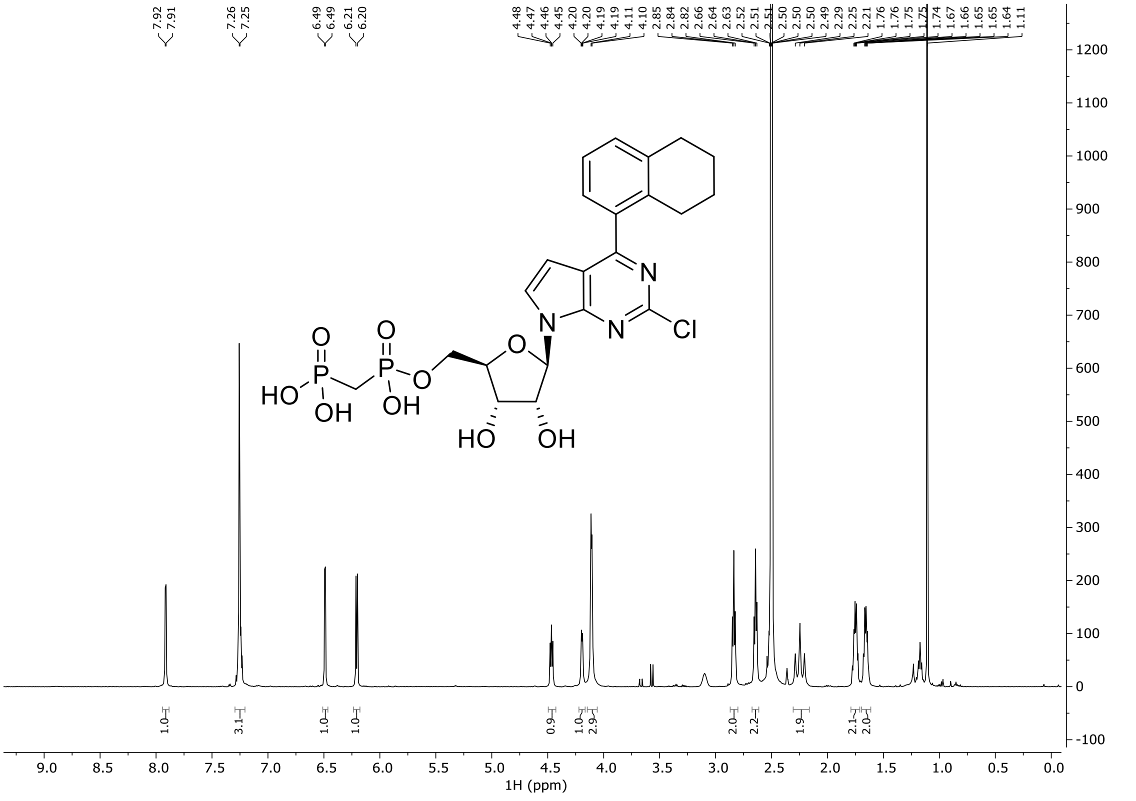

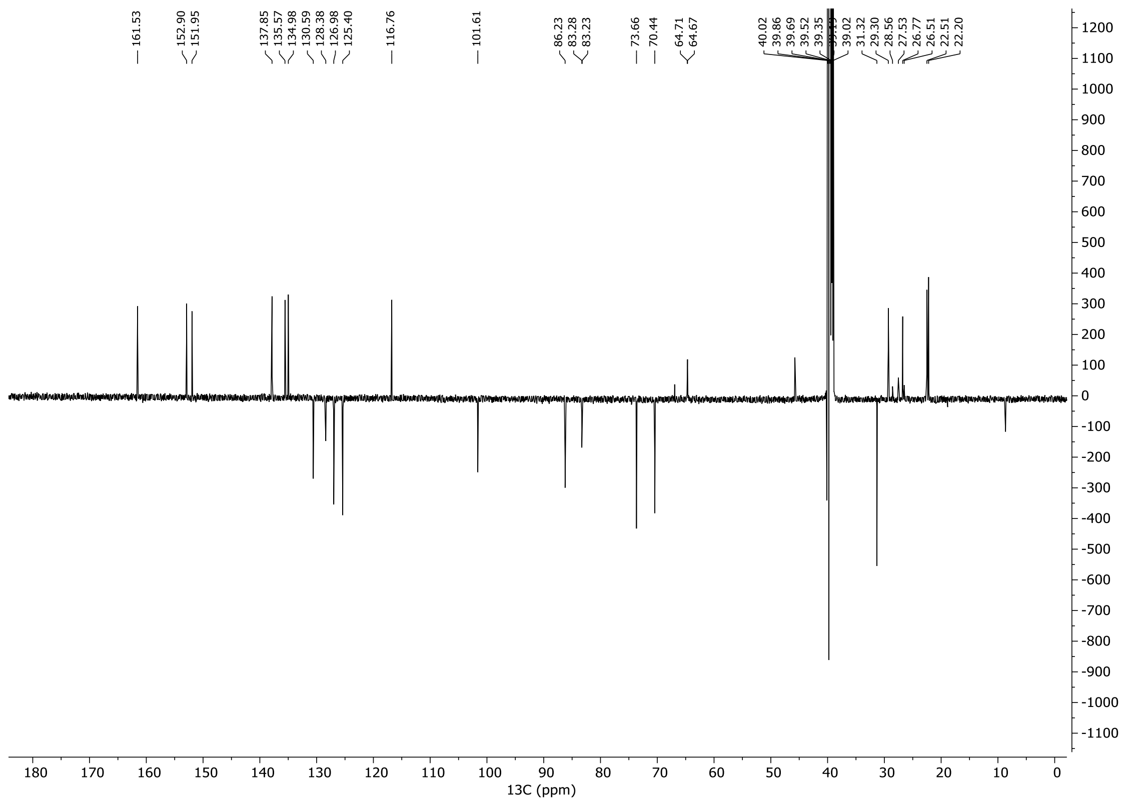
161.53
152.90
151.95
137.85
135.57
134.98
130.59
128.38
126.98
125.40
116.76
101.61
86.23
83.28
83.23
73.66
70.44
64.71
64.67
40.02
39.86
39.69
39.52
39.35
39.02
31.32
29.30
28.56
27.53
26.77
26.51
22.51
22.20
13C (ppm)

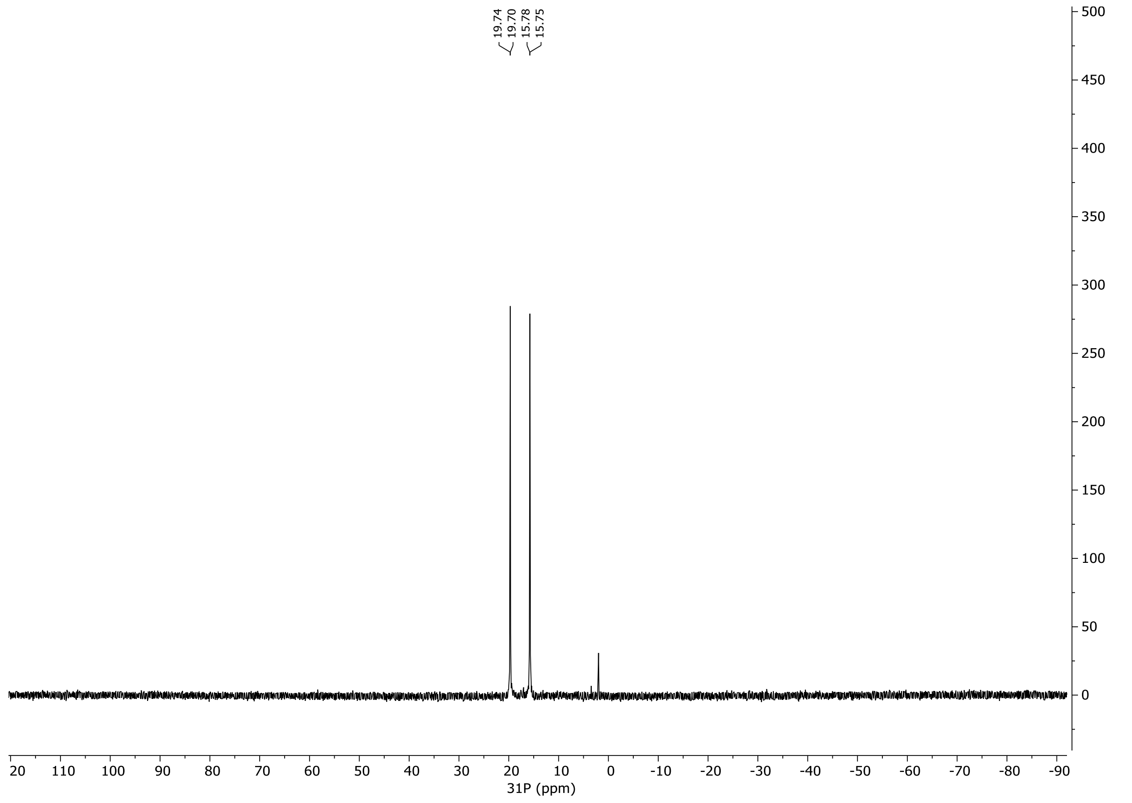
19.74
19.70
15.78
15.75
31P (ppm)

NMR spectra of compound **7B.23**

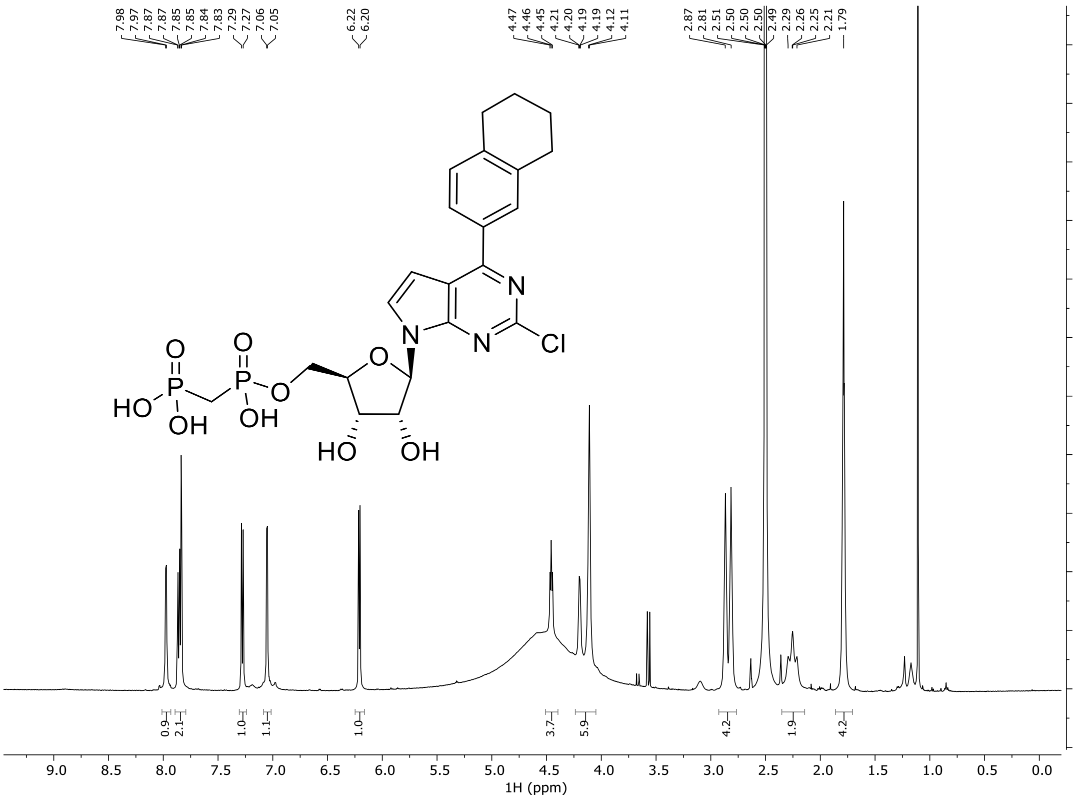

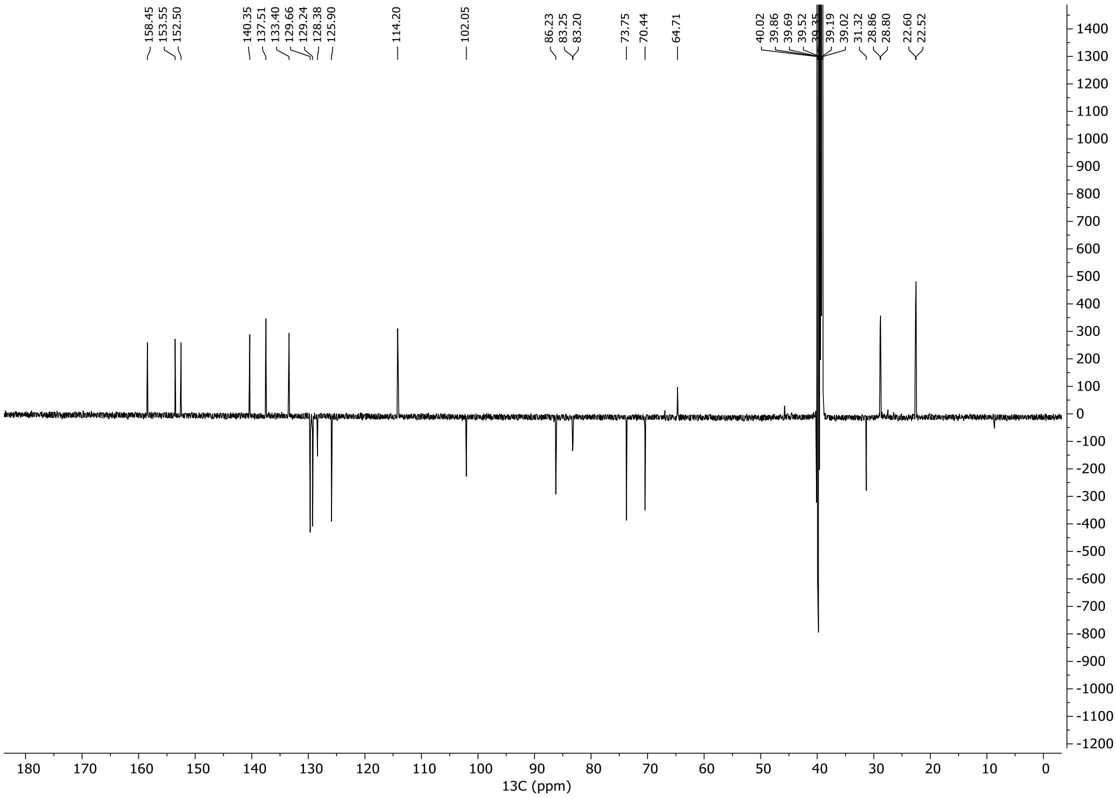

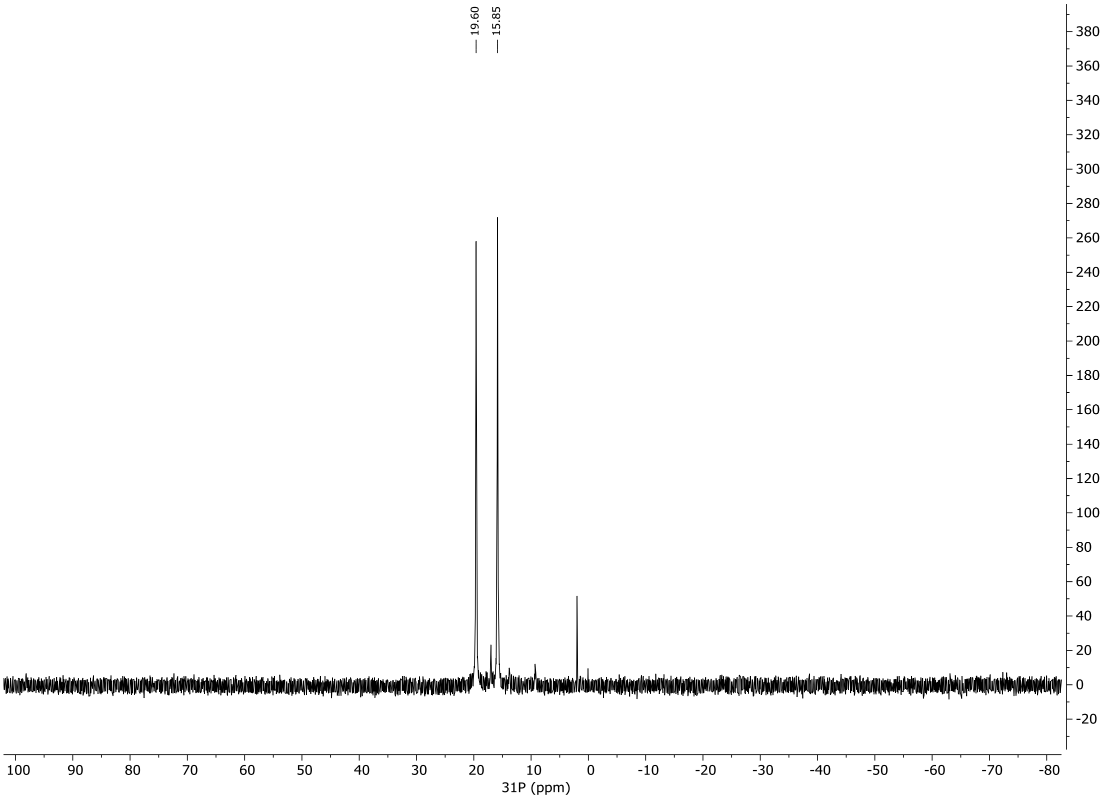

NMR spectra of compound **7B.24**

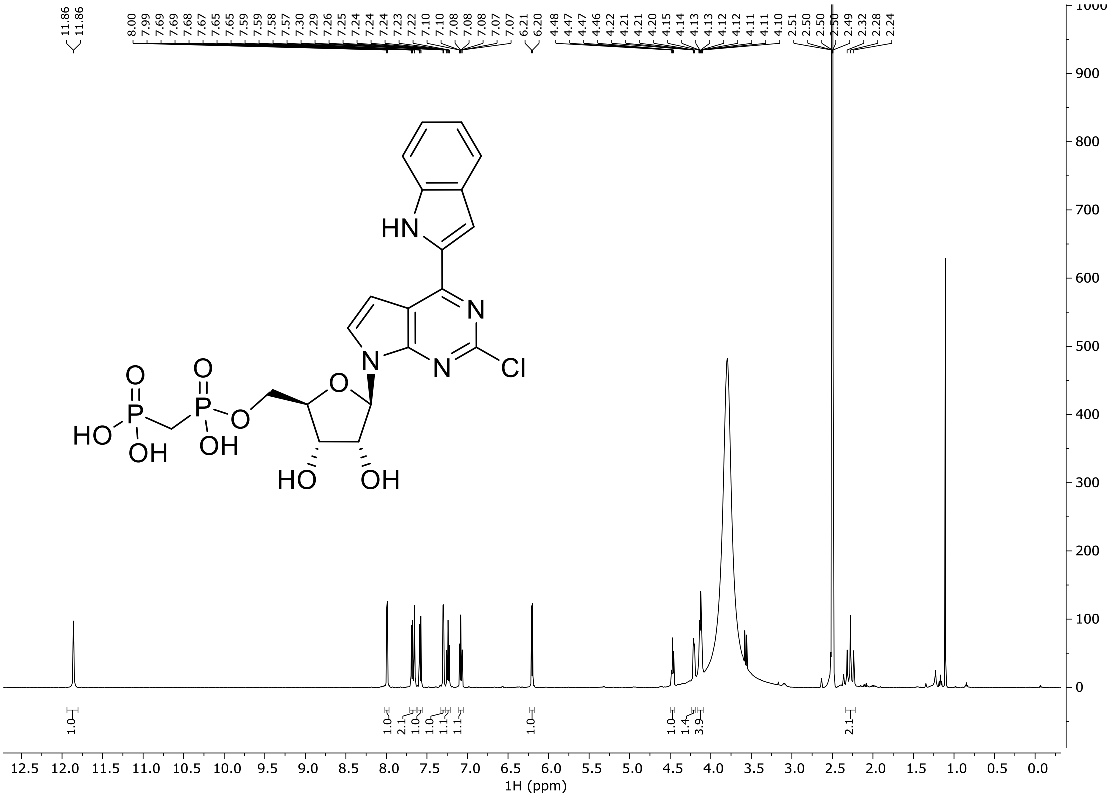

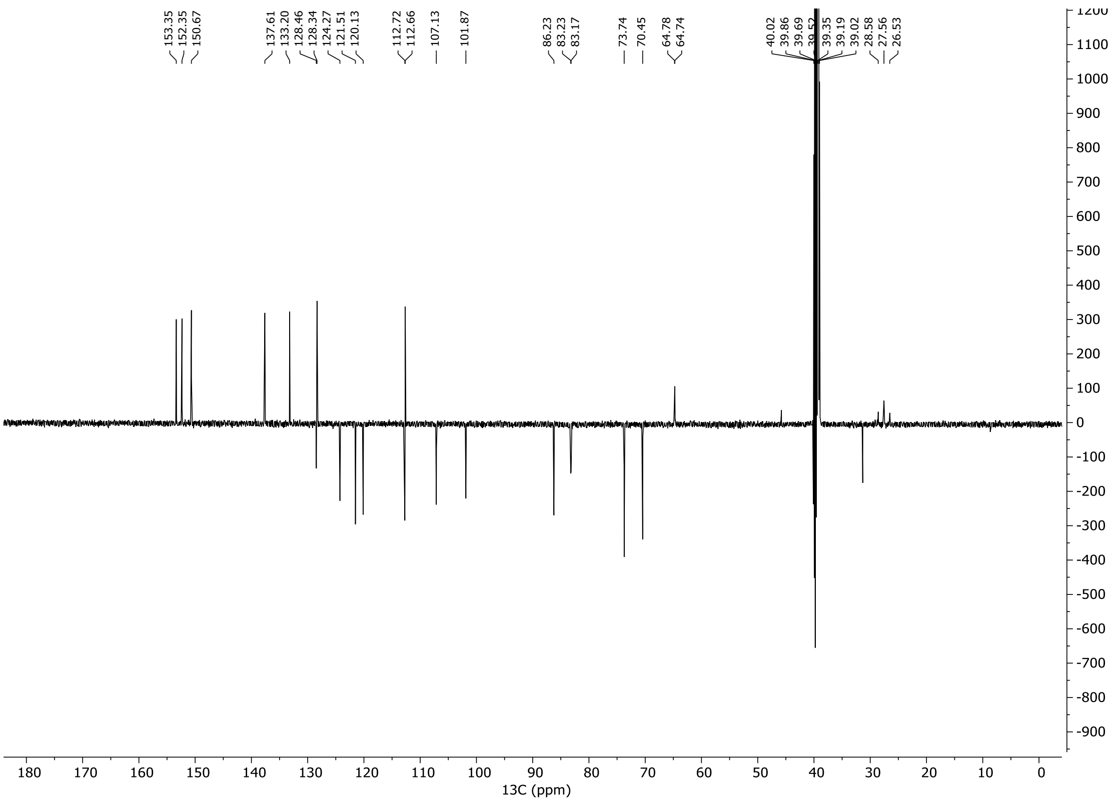
153.35
152.35
150.67
137.61
133.20
128.46
128.34
124.27
121.51
120.13
112.72
112.66
107.13
101.87
86.23
83.23
83.17
73.74
70.45
64.78
64.74
40.02
39.86
39.69
39.52
39.35
39.19
39.02
28.58
27.56
26.53
13C (ppm)

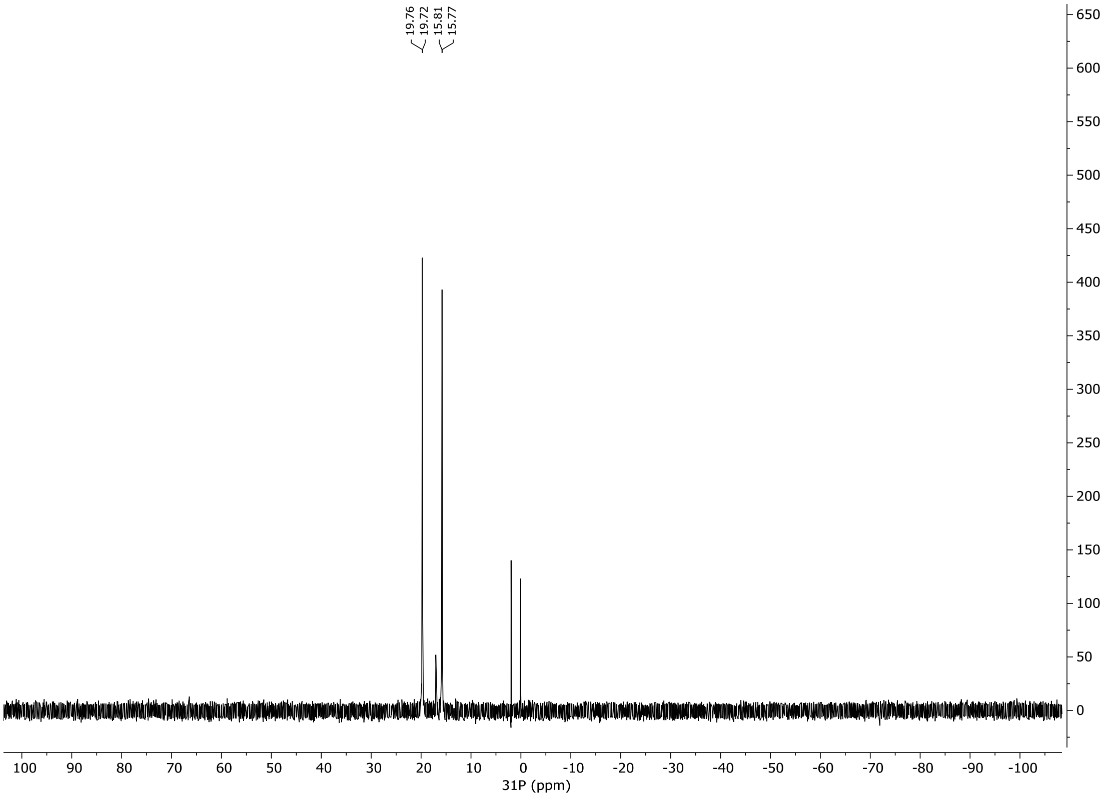
19.76
19.72
15.81
15.77
31P (ppm)

NMR spectra of compound **7B.25**

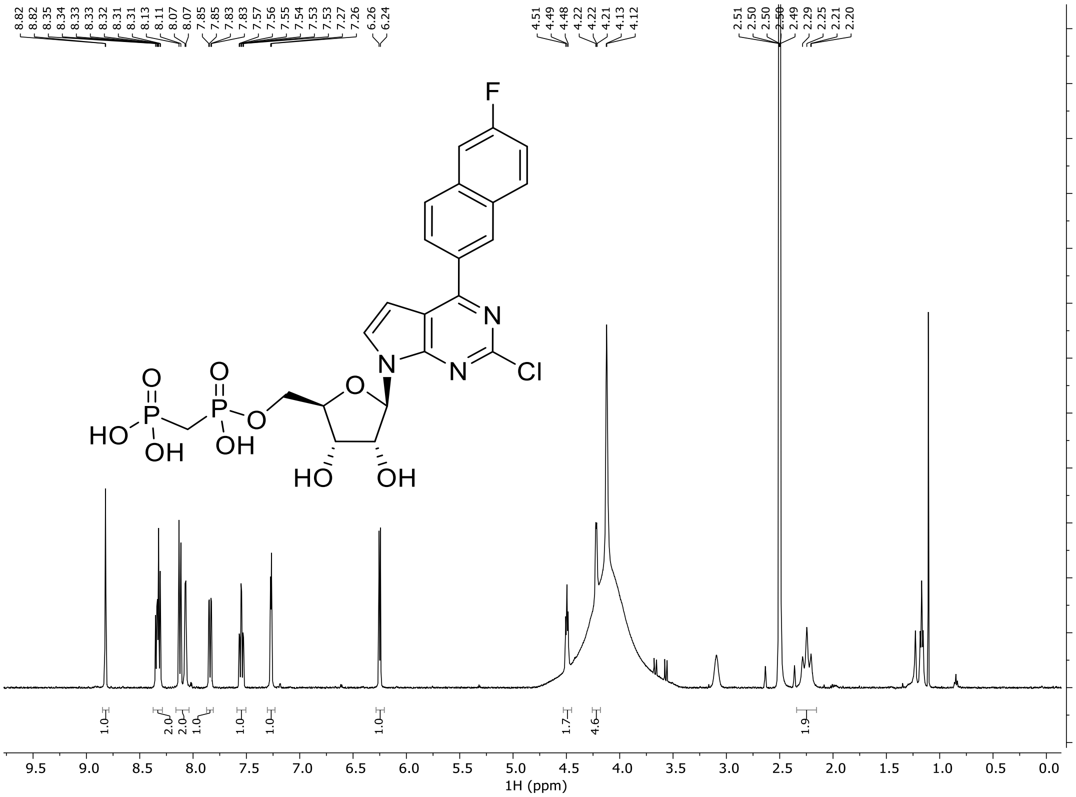

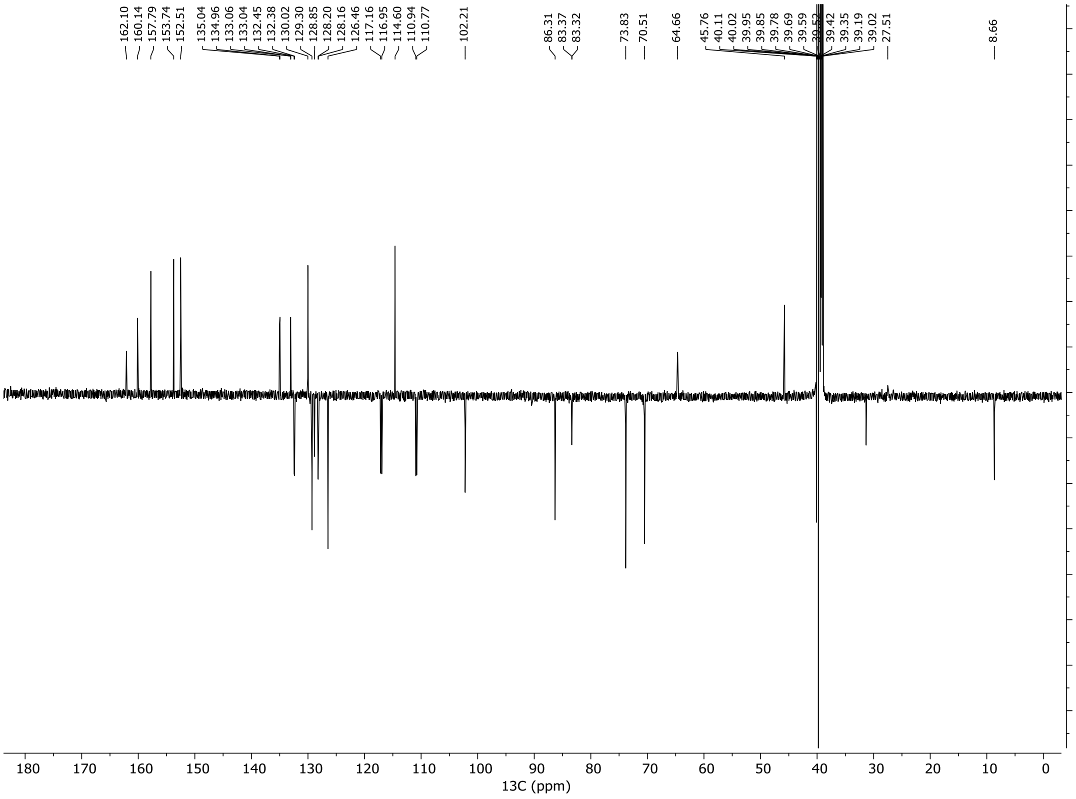

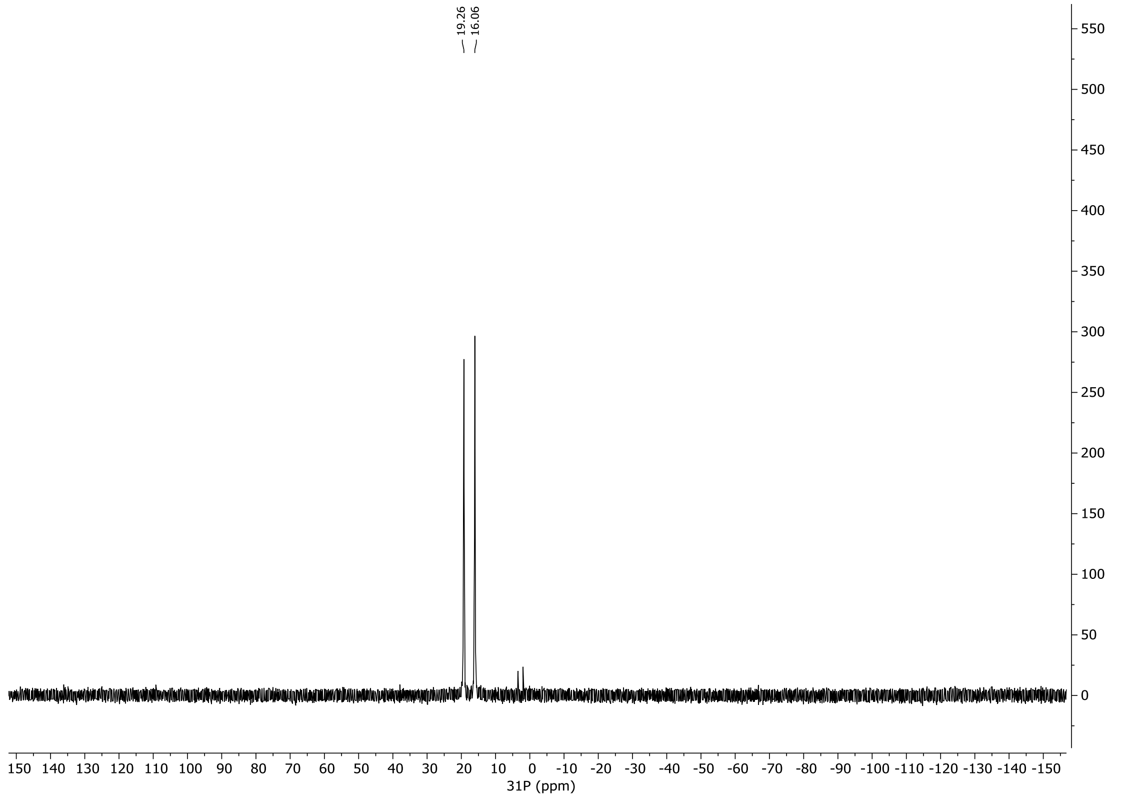
19.26
16.06
31P (ppm)

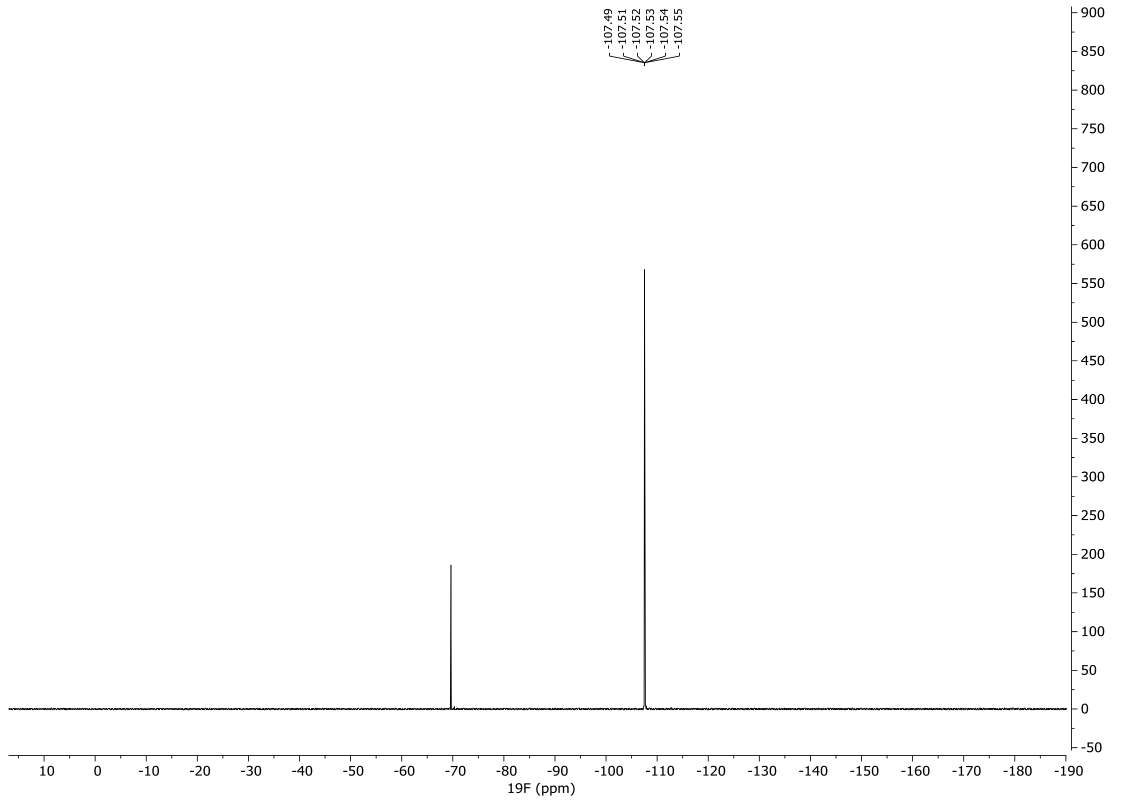
-107.49
-107.51
-107.52
-107.53
-107.54
-107.55
19F (ppm)

NMR spectra of compound **7B.26**

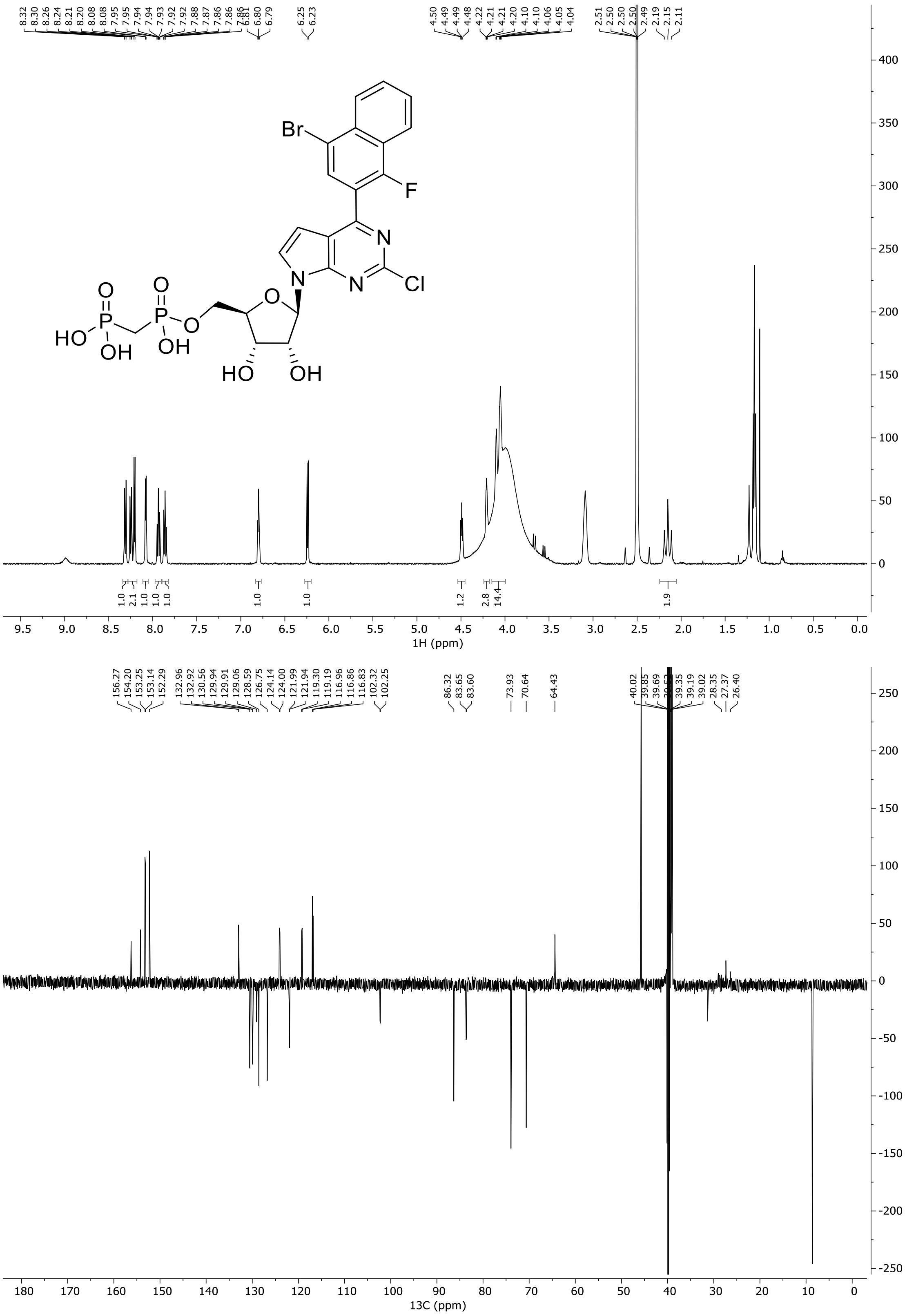

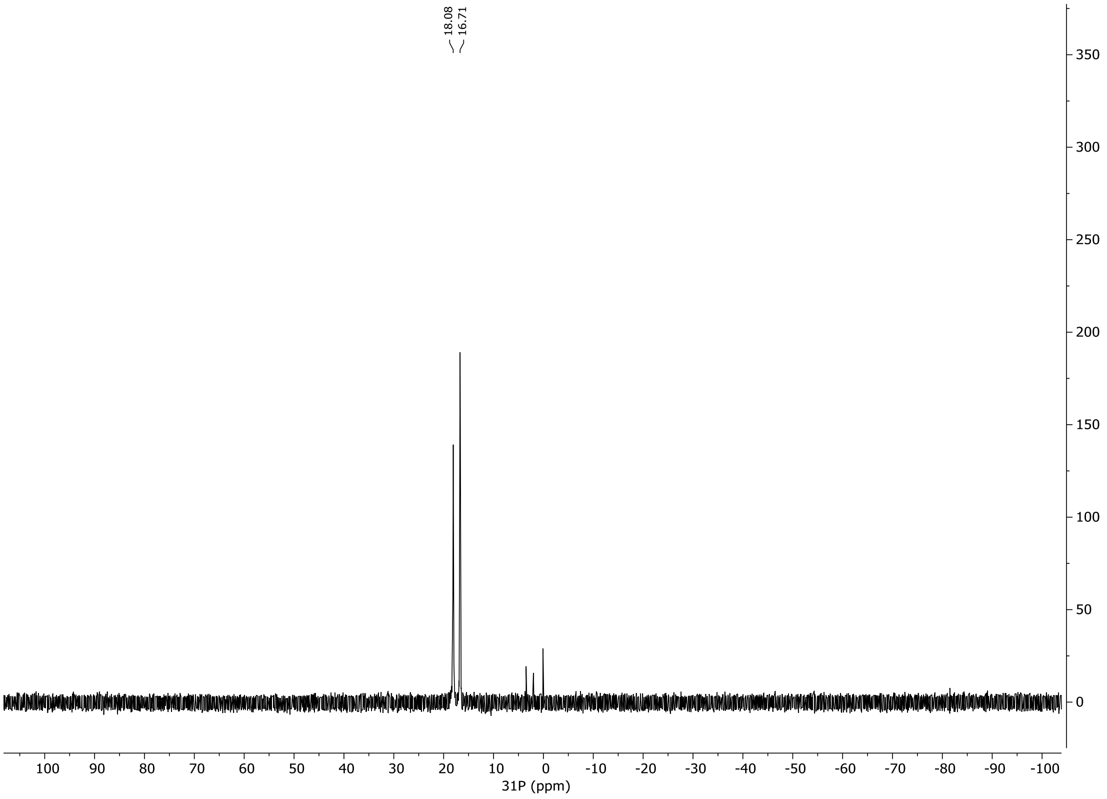
18.08
16.71
31P (ppm)

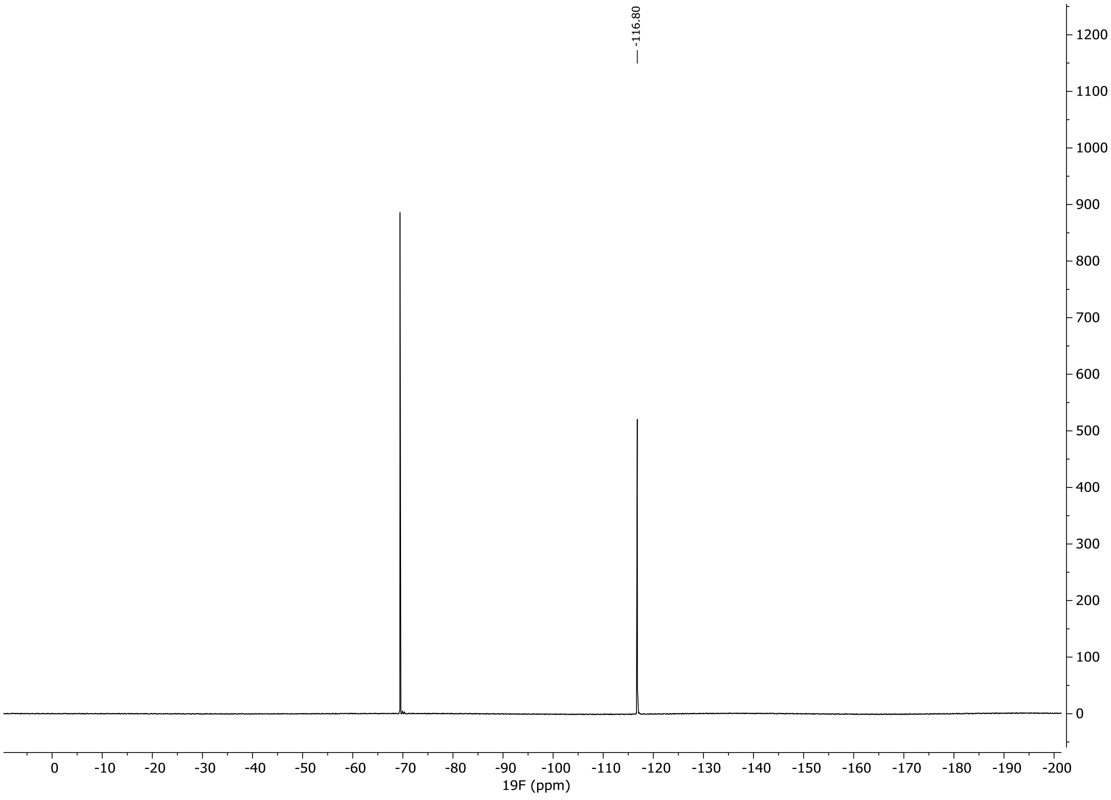
-116.80
19F (ppm)

NMR spectra of compound **7B.27**

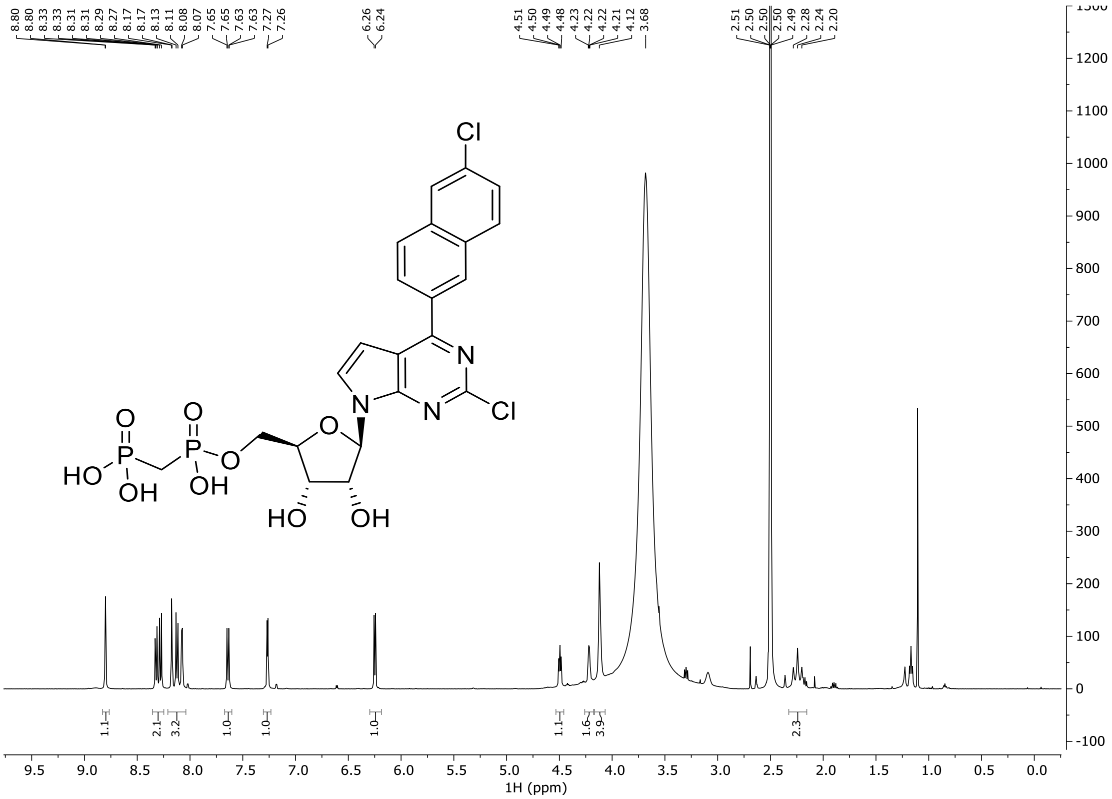

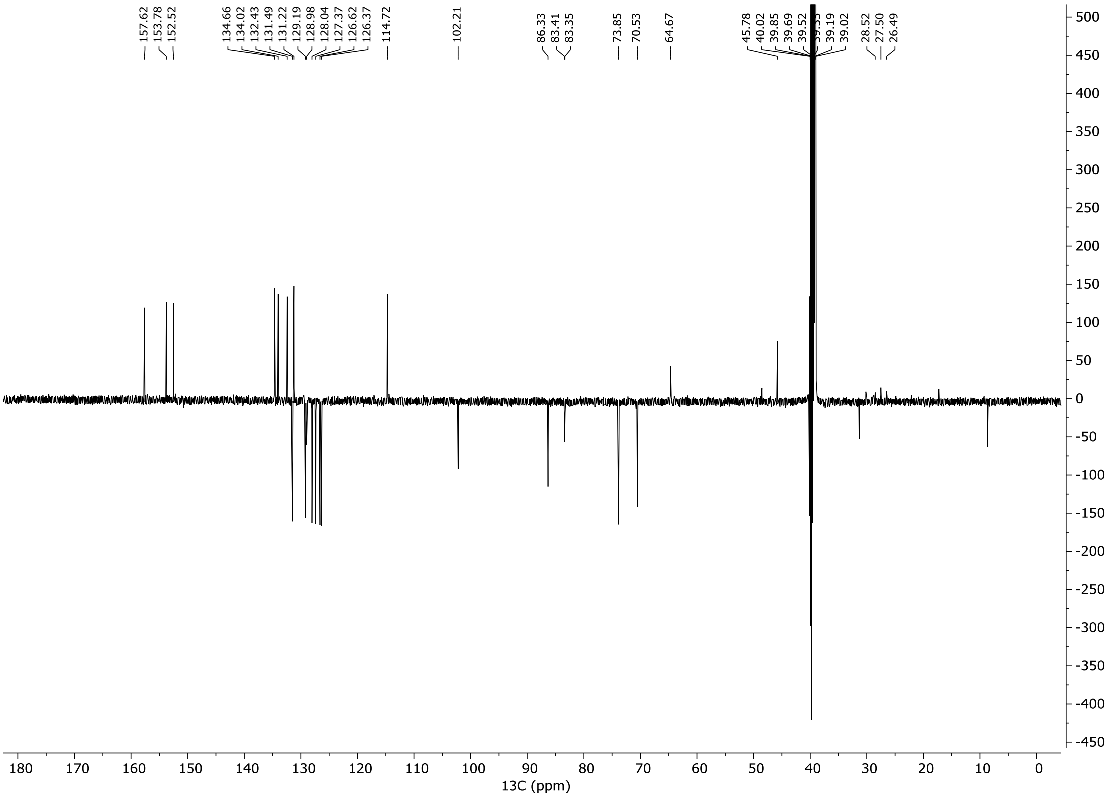

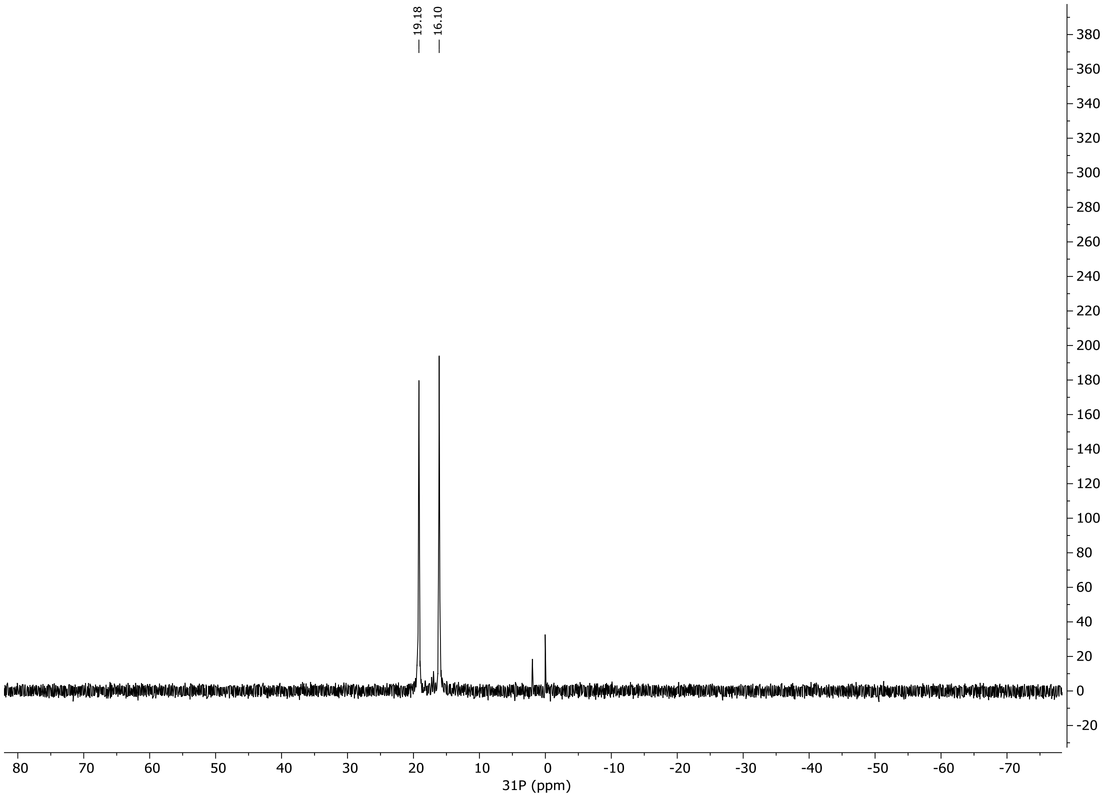

NMR spectra of compound **7B.28**

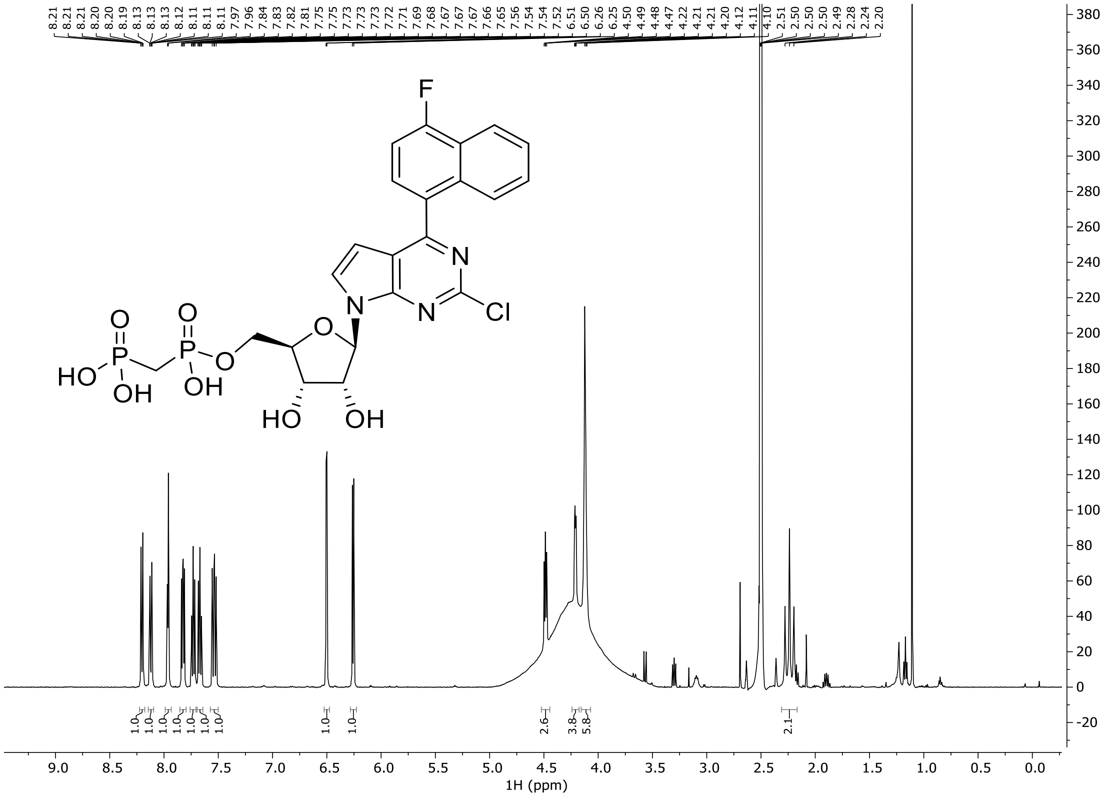

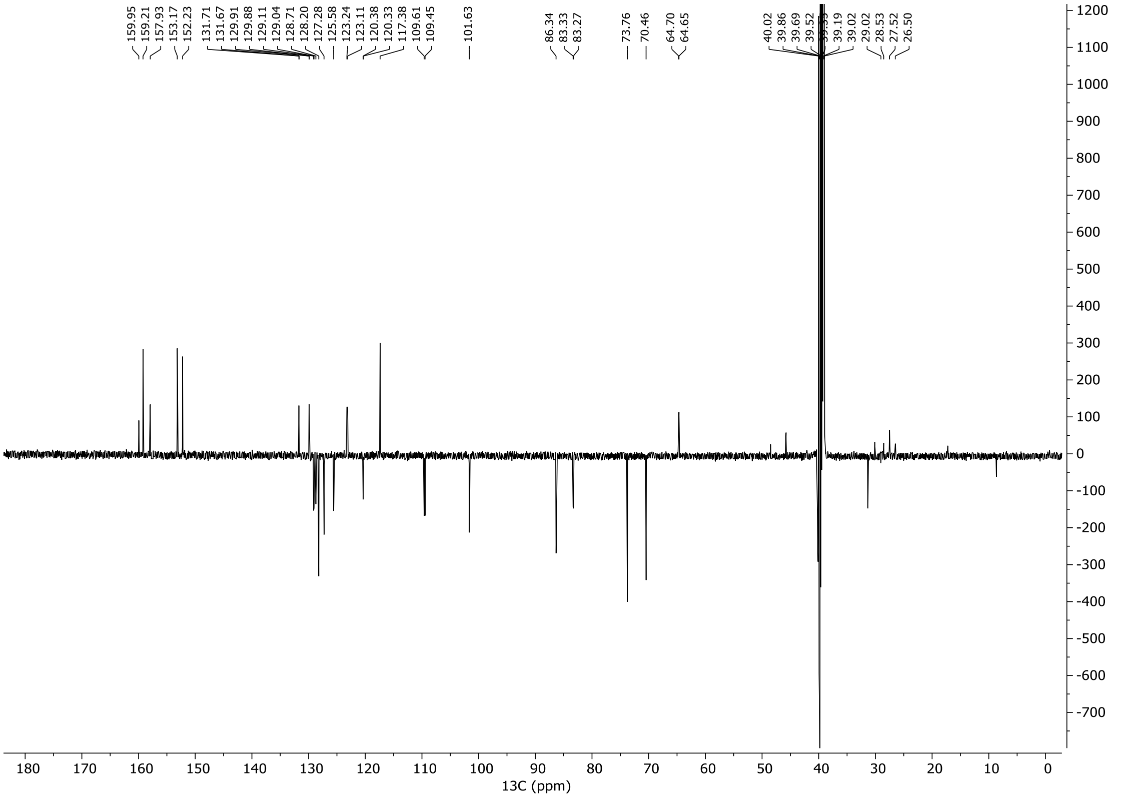
159.95
159.21
157.93
153.17
152.23
131.71
131.67
129.91
129.88
129.11
129.04
128.71
128.20
127.28
125.58
123.24
123.11
120.38
120.33
117.38
109.61
109.45
101.63
86.34
83.33
83.27
73.76
70.46
64.70
64.65
40.02
39.86
39.69
39.52
39.19
39.02
29.02
28.53
27.52
26.50
13C (ppm)

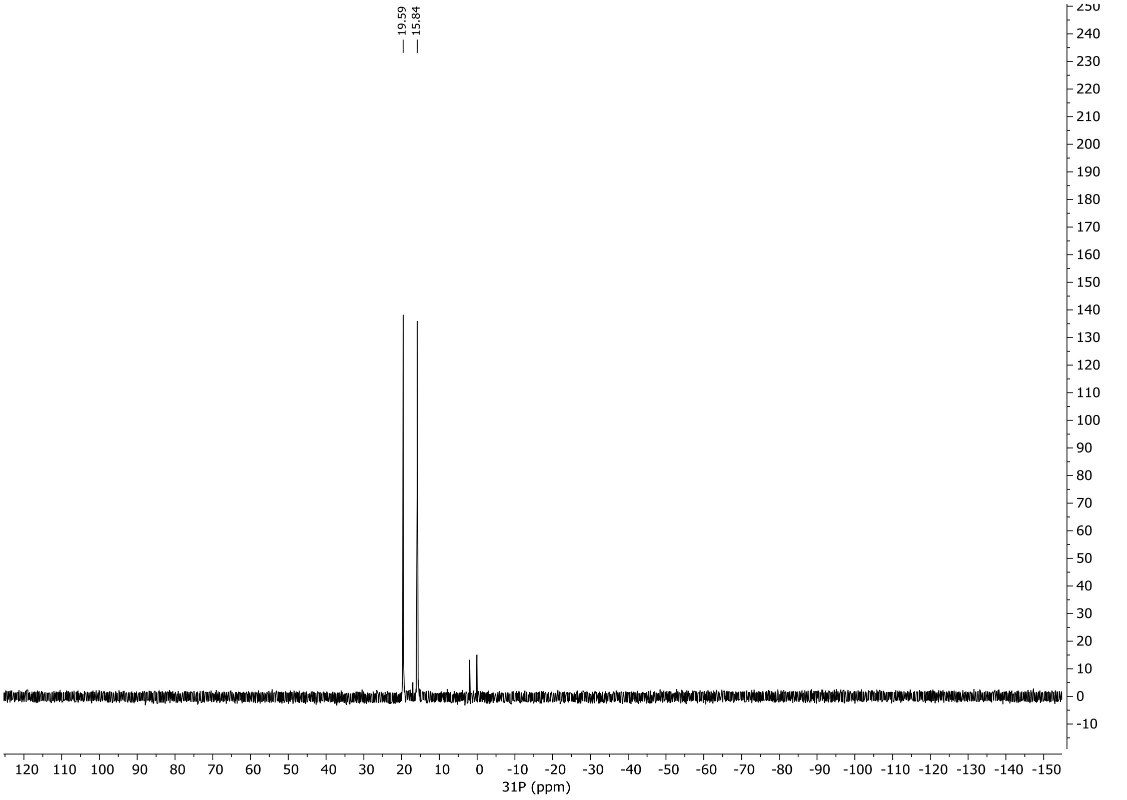
19.59
15.84
31P (ppm)

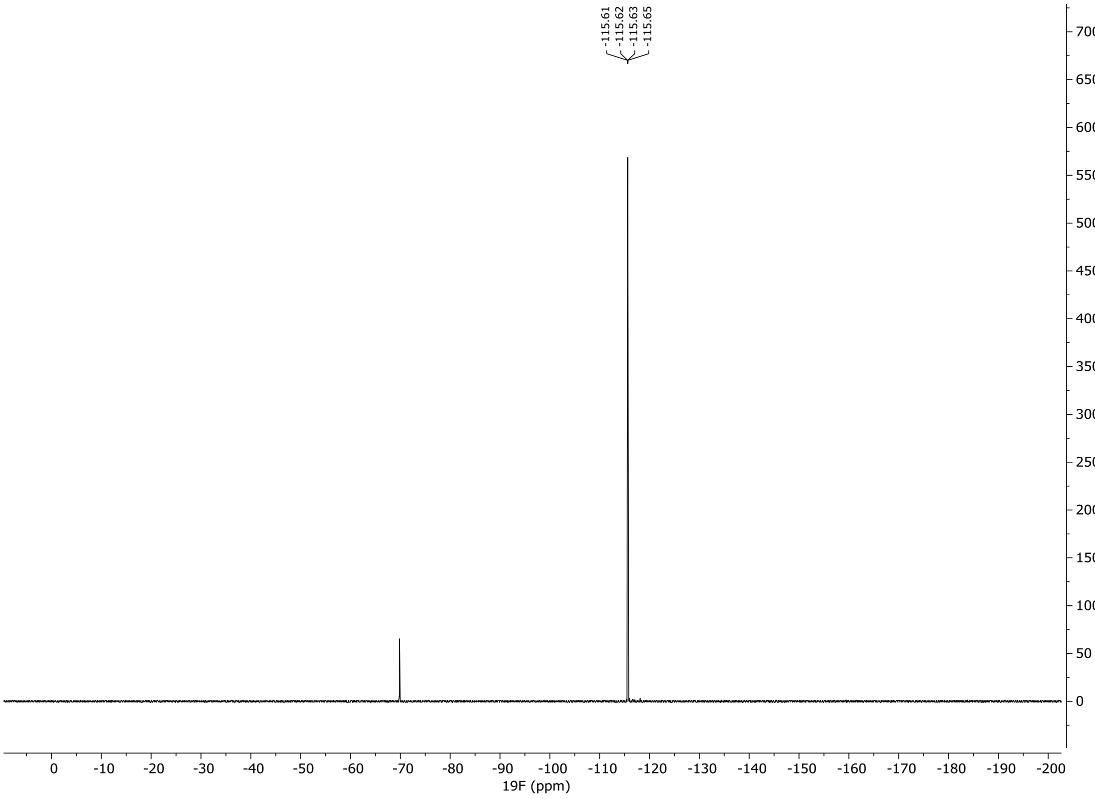

NMR spectra of compound **7B.29**

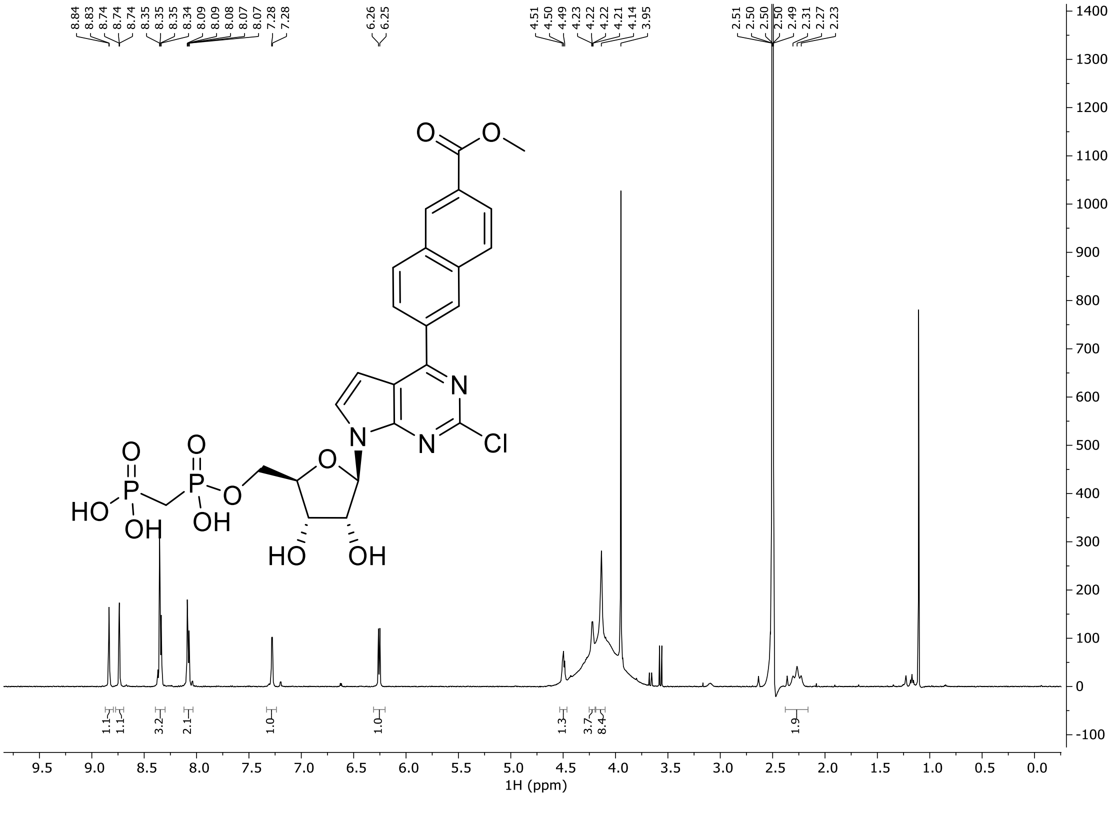

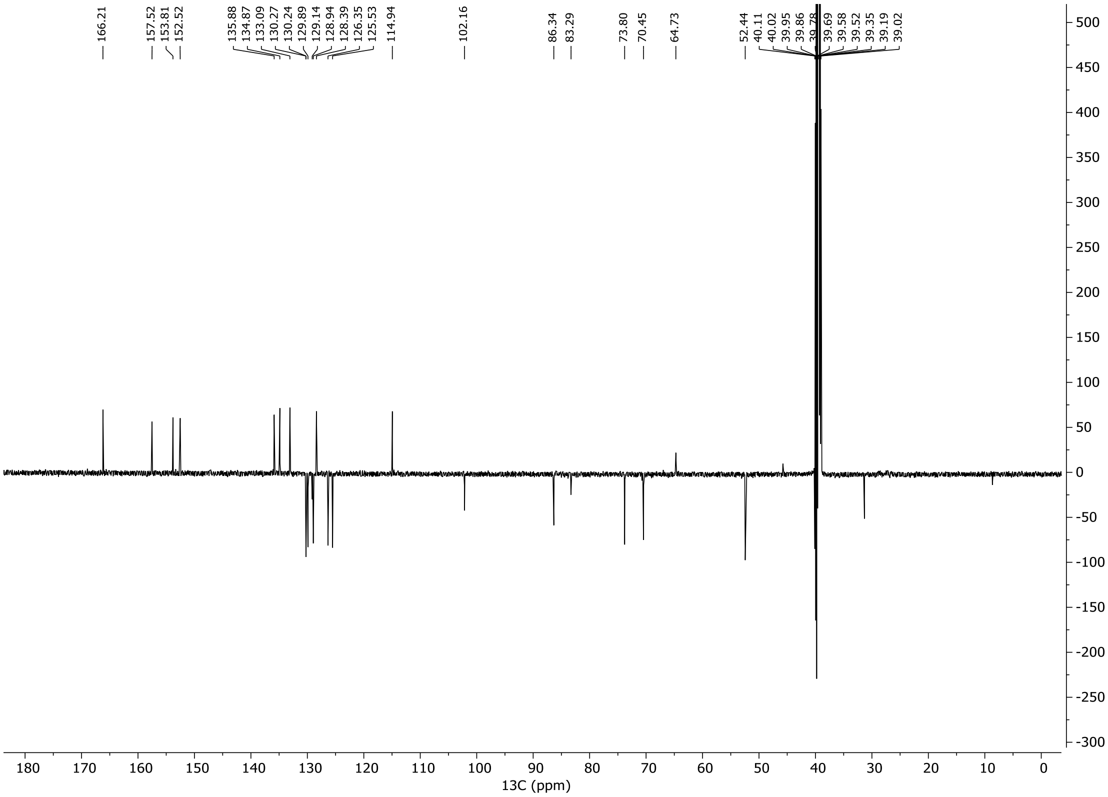
166.21
157.52
153.81
152.52
135.88
134.87
133.09
130.27
130.24
129.89
129.14
128.94
128.39
126.35
125.53
114.94
102.16
86.34
83.29
73.80
70.45
64.73
52.44
40.11
40.02
39.95
39.86
39.78
39.69
39.58
39.52
39.35
39.19
39.02
13C (ppm)

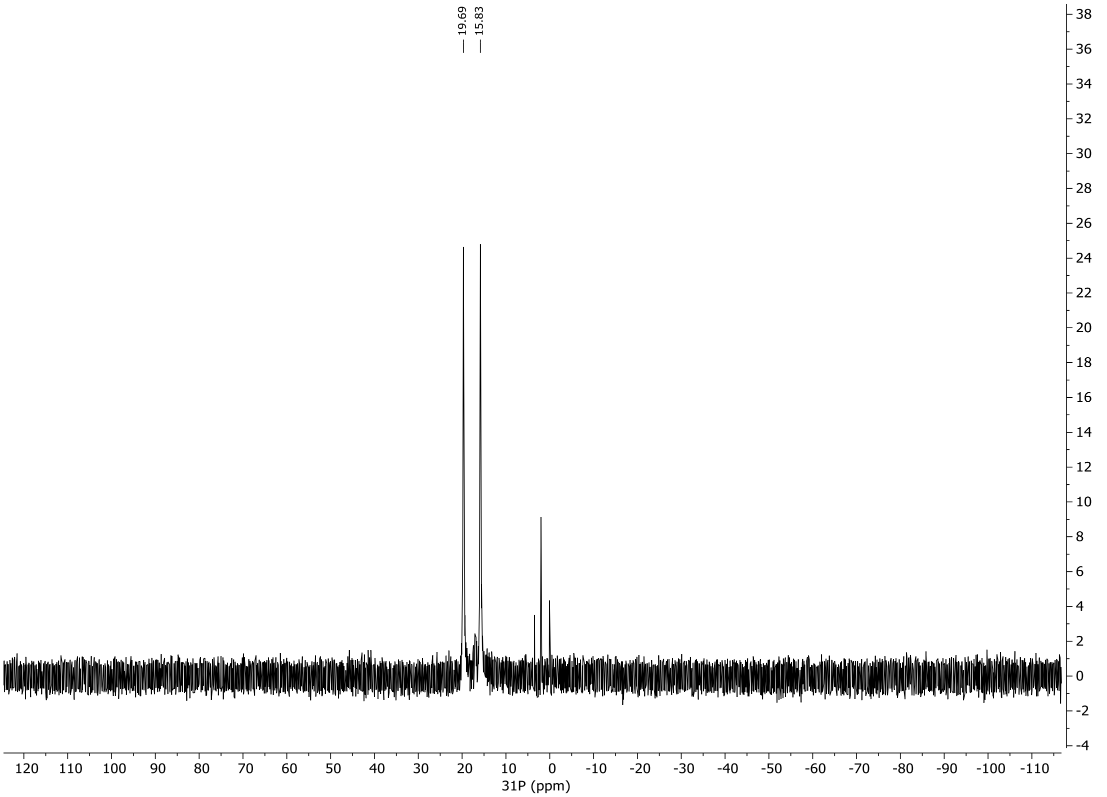
19.69
15.83
31P (ppm)

NMR spectra of compound **7B.30**

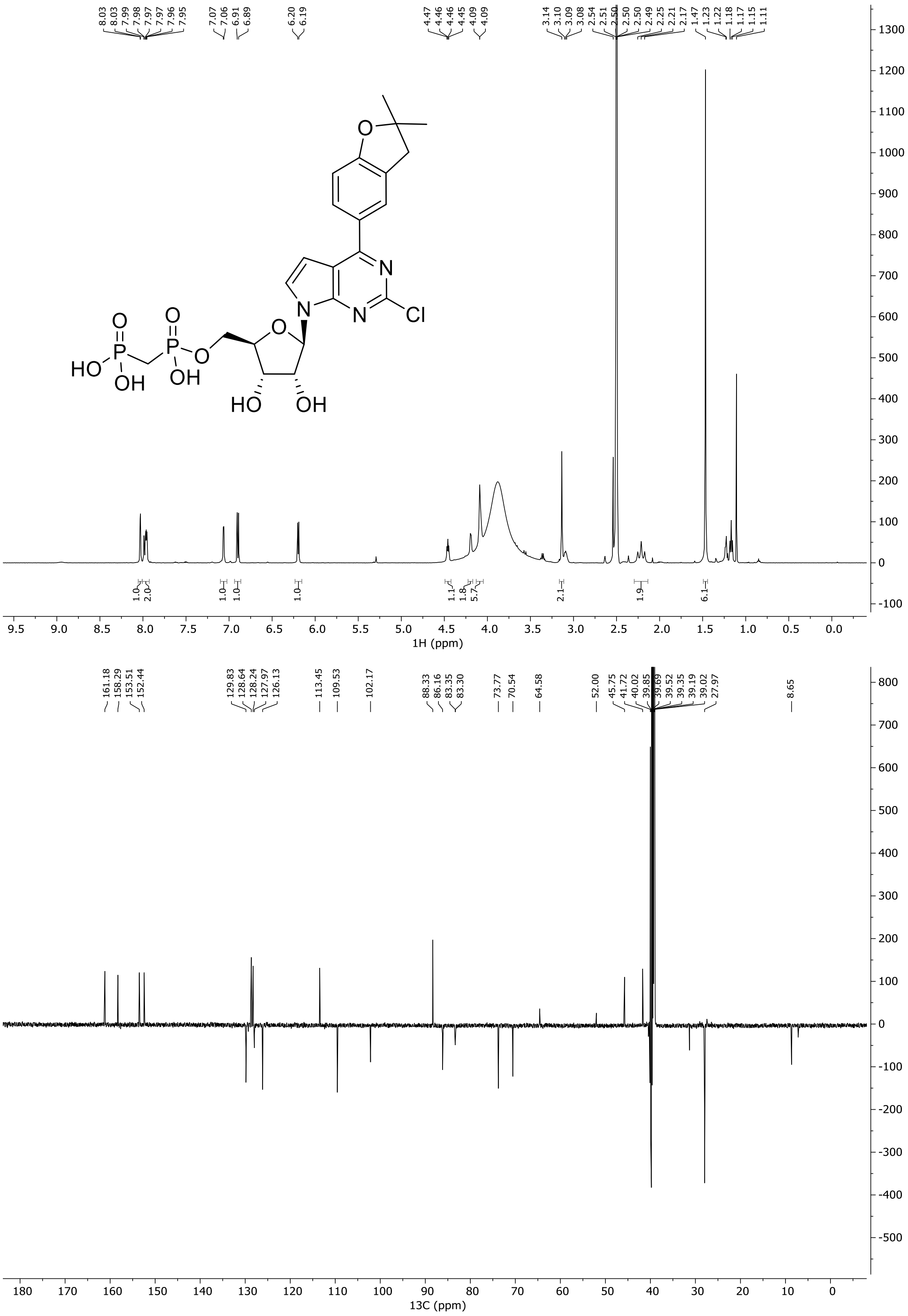

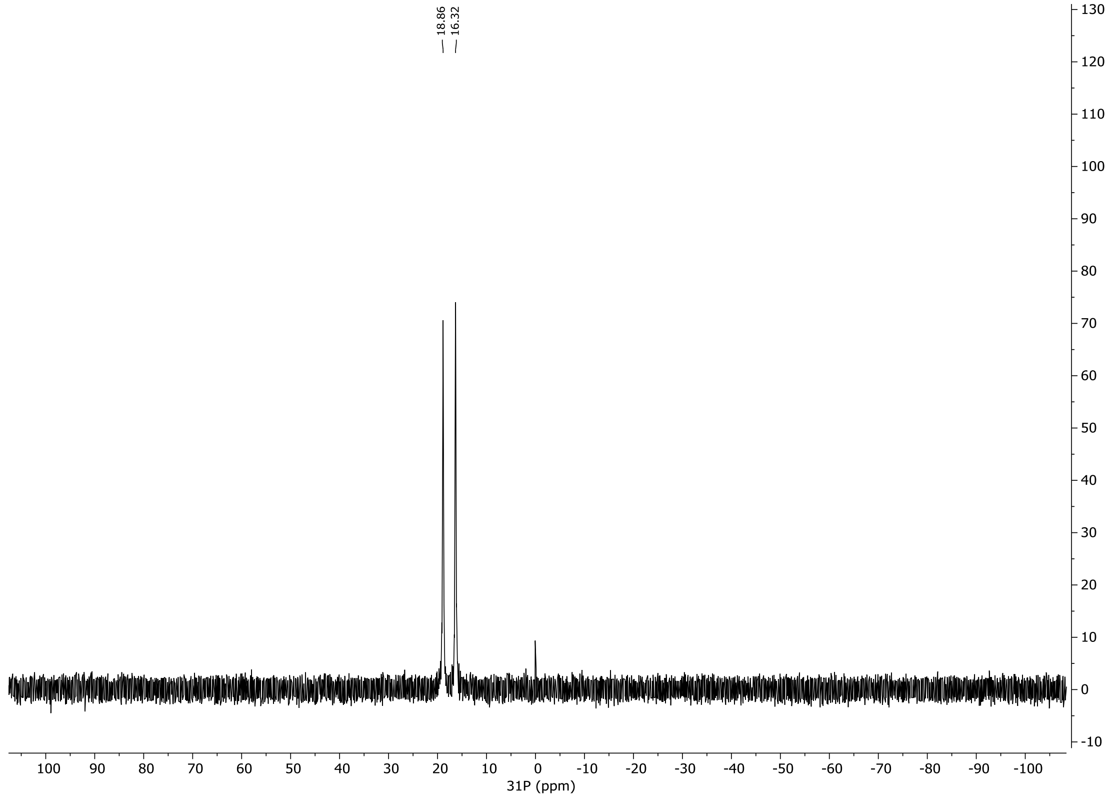

NMR spectra of compound **7B.31**

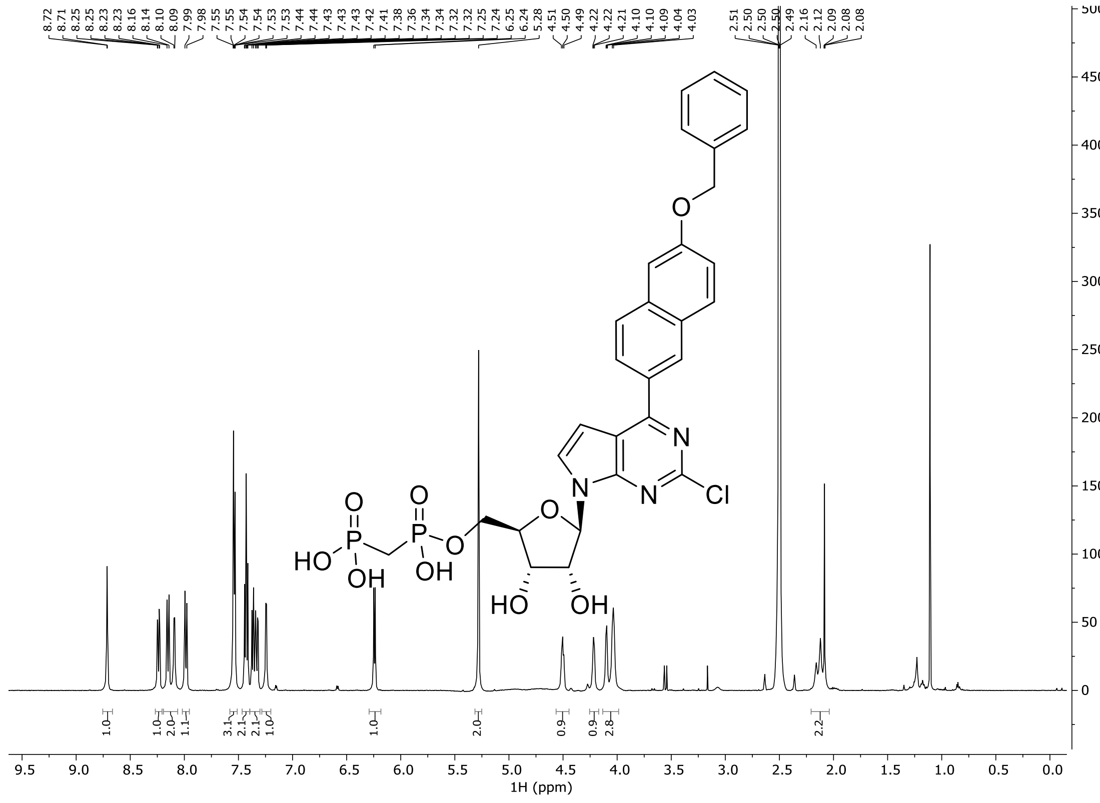

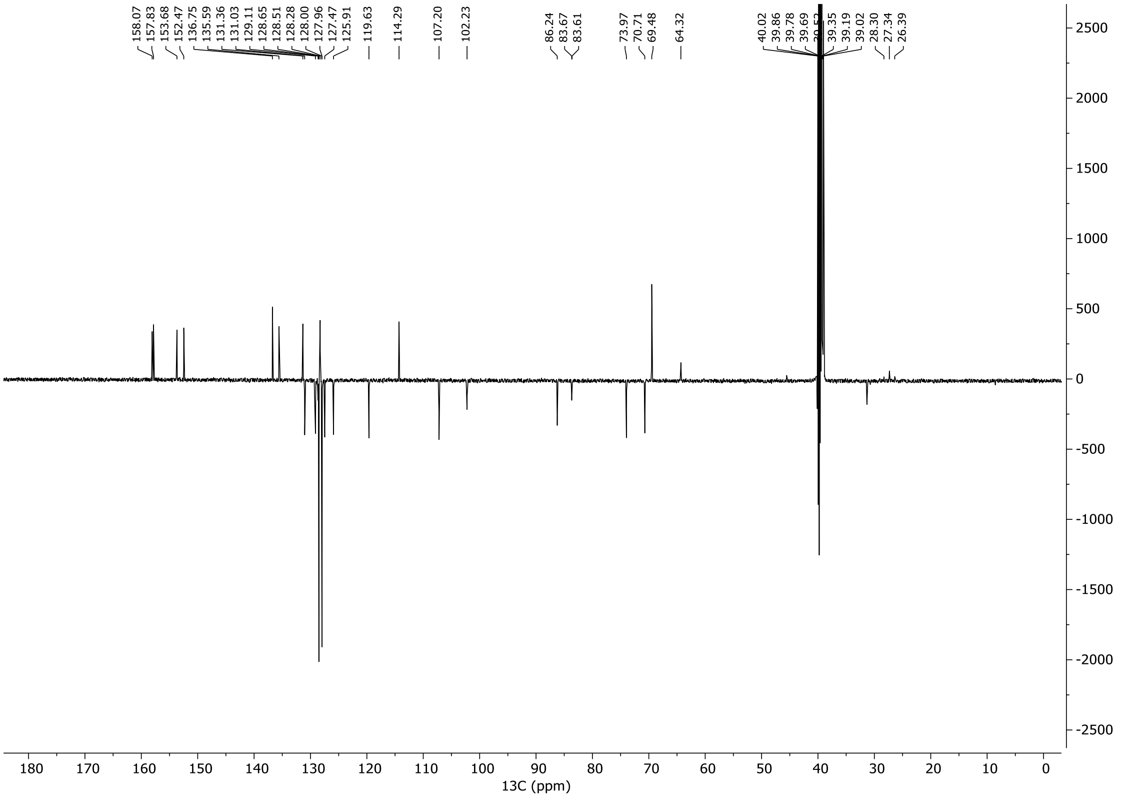
158.07
157.83
153.68
152.47
136.75
135.59
131.36
131.03
129.11
128.65
128.51
128.28
128.00
127.96
127.47
125.91
119.63
114.29
107.20
102.23
86.24
83.67
83.61
73.97
70.71
69.48
64.32
40.02
39.86
39.78
39.69
39.35
39.19
39.02
28.30
27.34
26.39
13C (ppm)

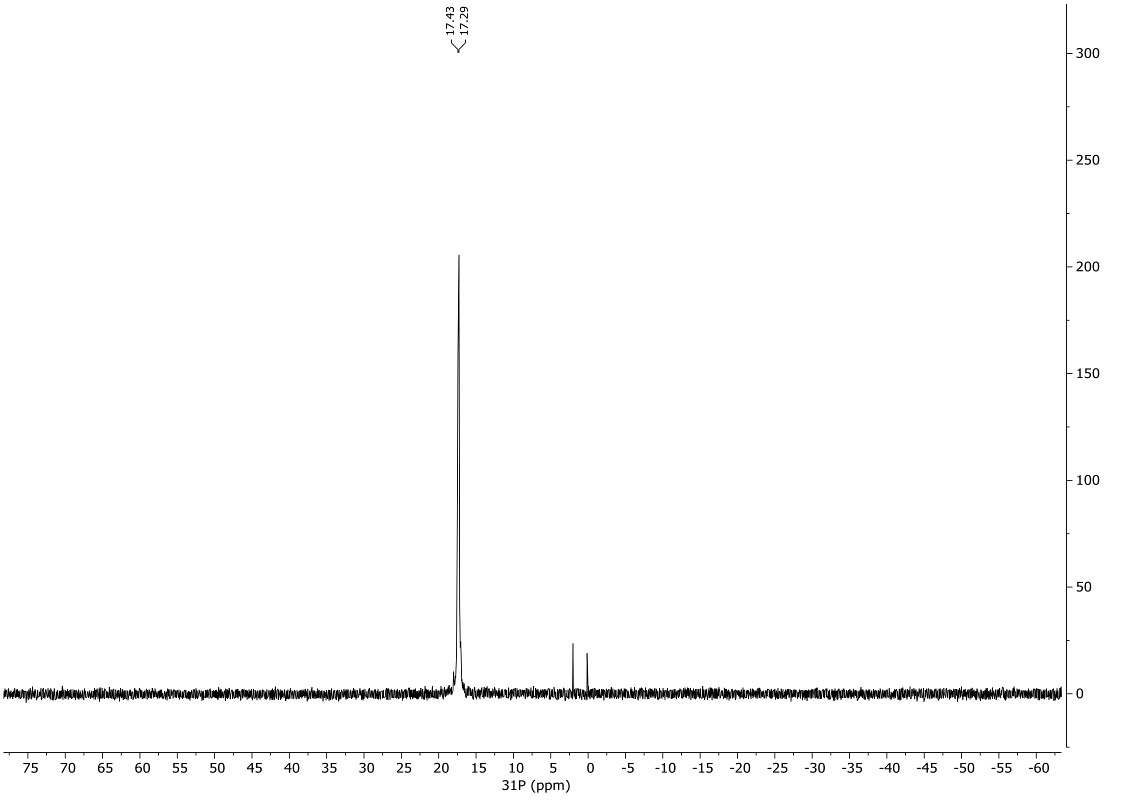
17.43
17.29
31P (ppm)

NMR spectra of compound **7B.32**

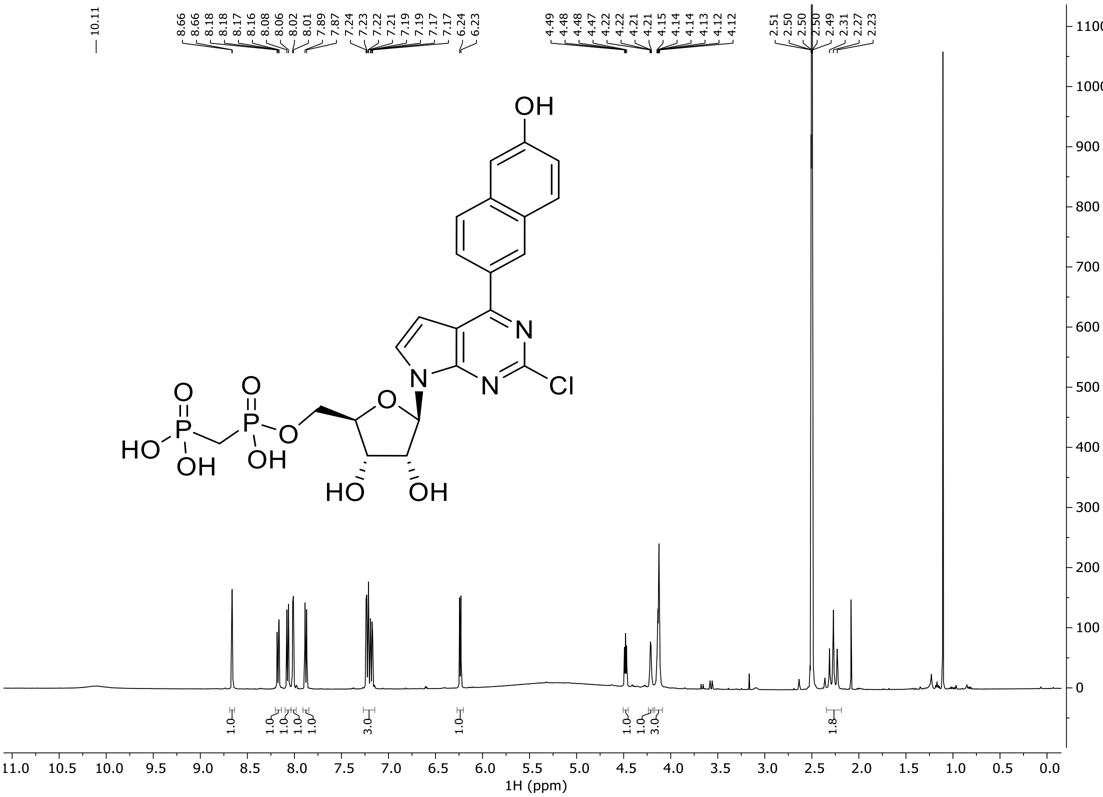

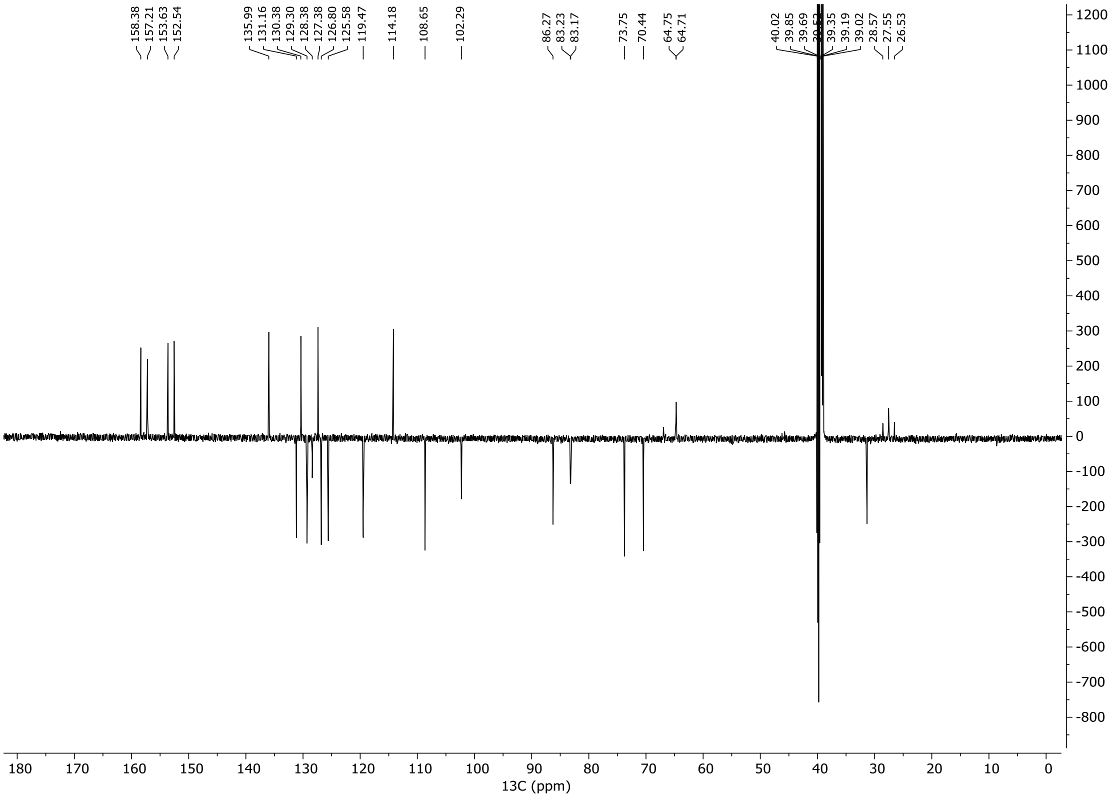

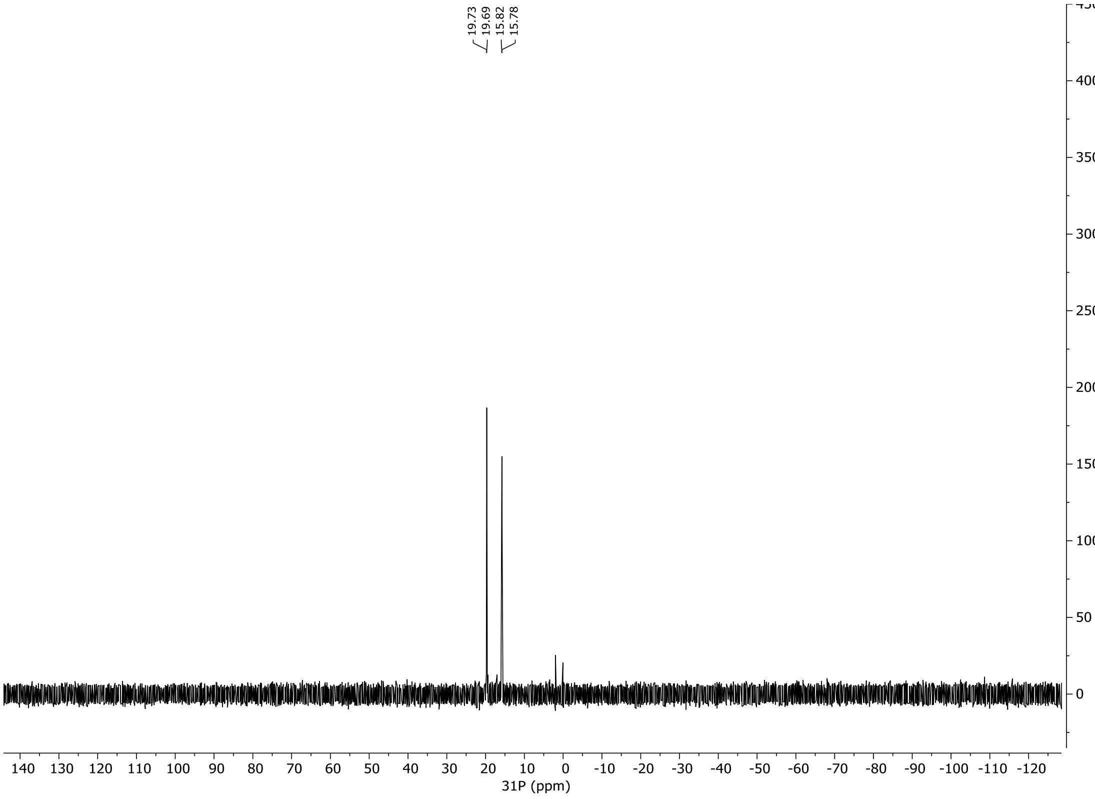

NMR spectra of compound **7B.33**

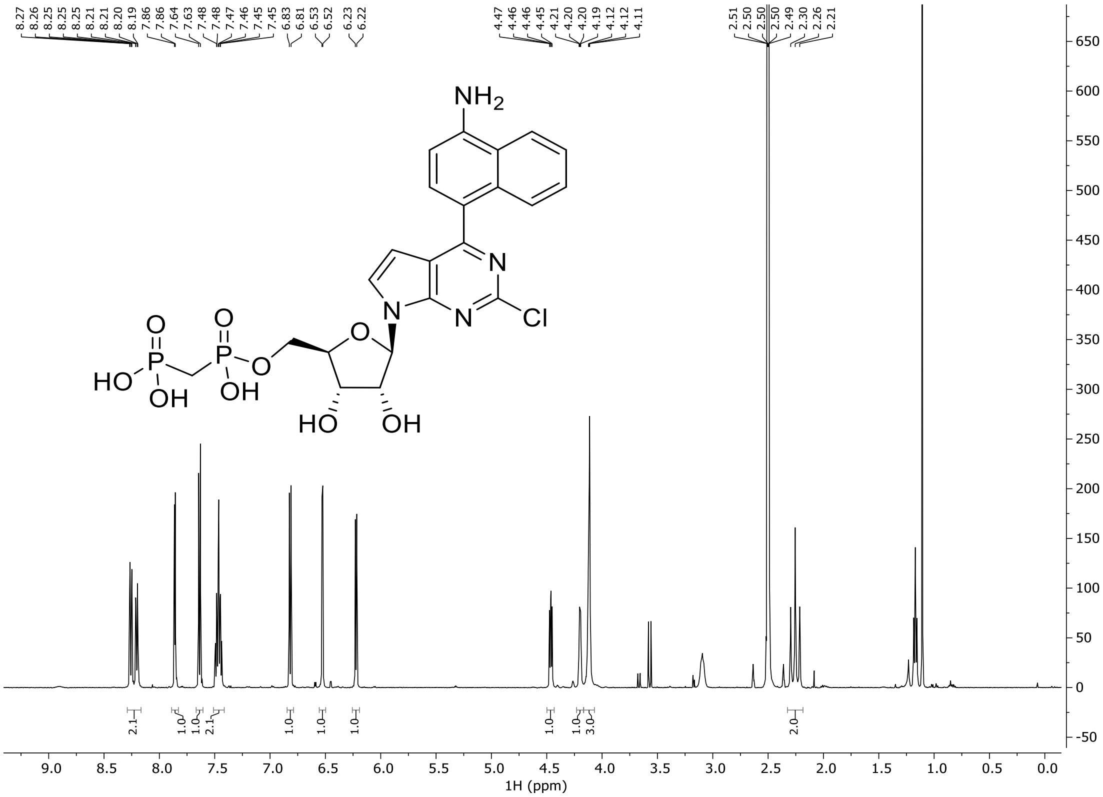

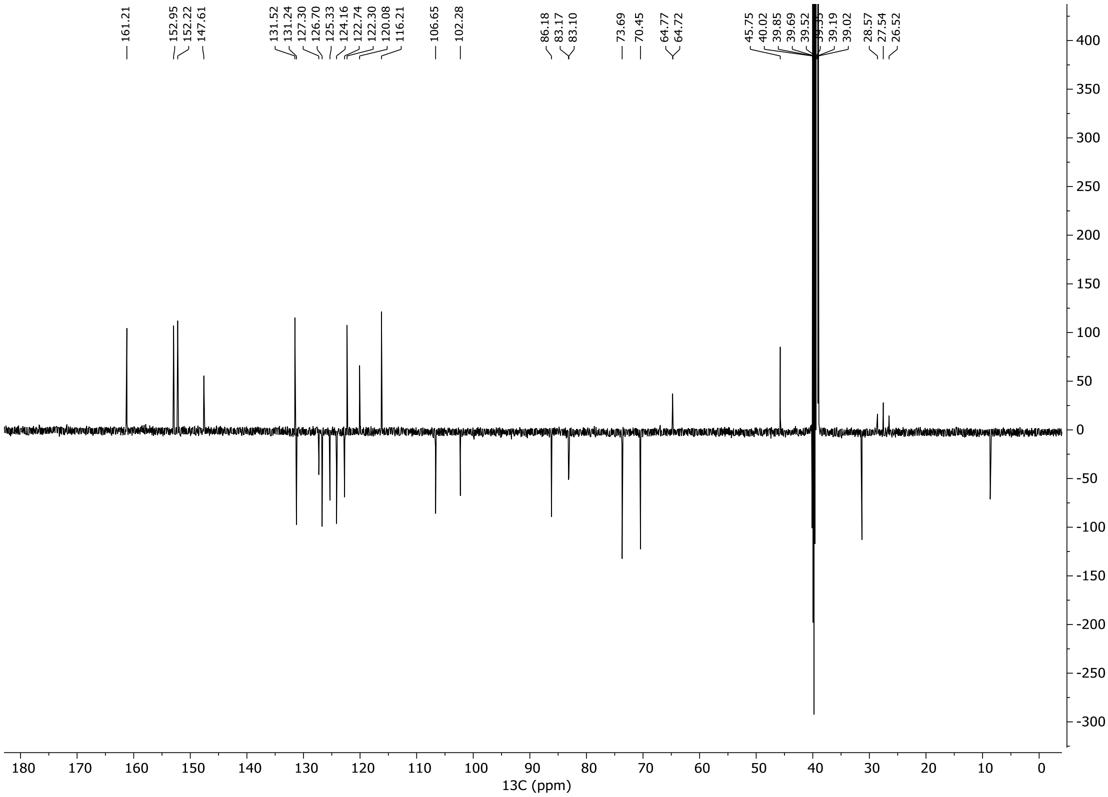
161.21
152.95
152.22
147.61
131.52
131.24
127.30
126.70
125.33
124.16
122.74
122.30
120.08
116.21
106.65
102.28
86.18
83.17
83.10
73.69
70.45
64.77
64.72
45.75
40.02
39.85
39.69
39.52
39.19
39.02
28.57
27.54
26.52
13C (ppm)

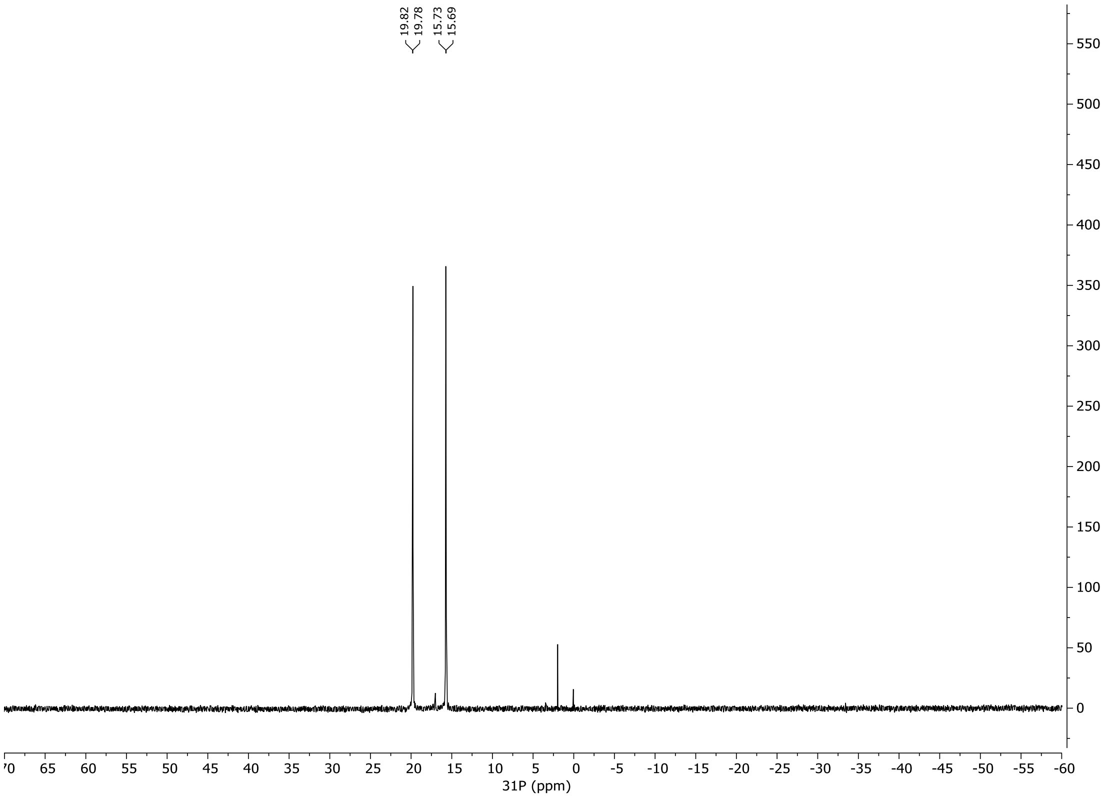
19.82
19.78
15.73
15.69
31P (ppm)

NMR spectra of compound **7B.34**

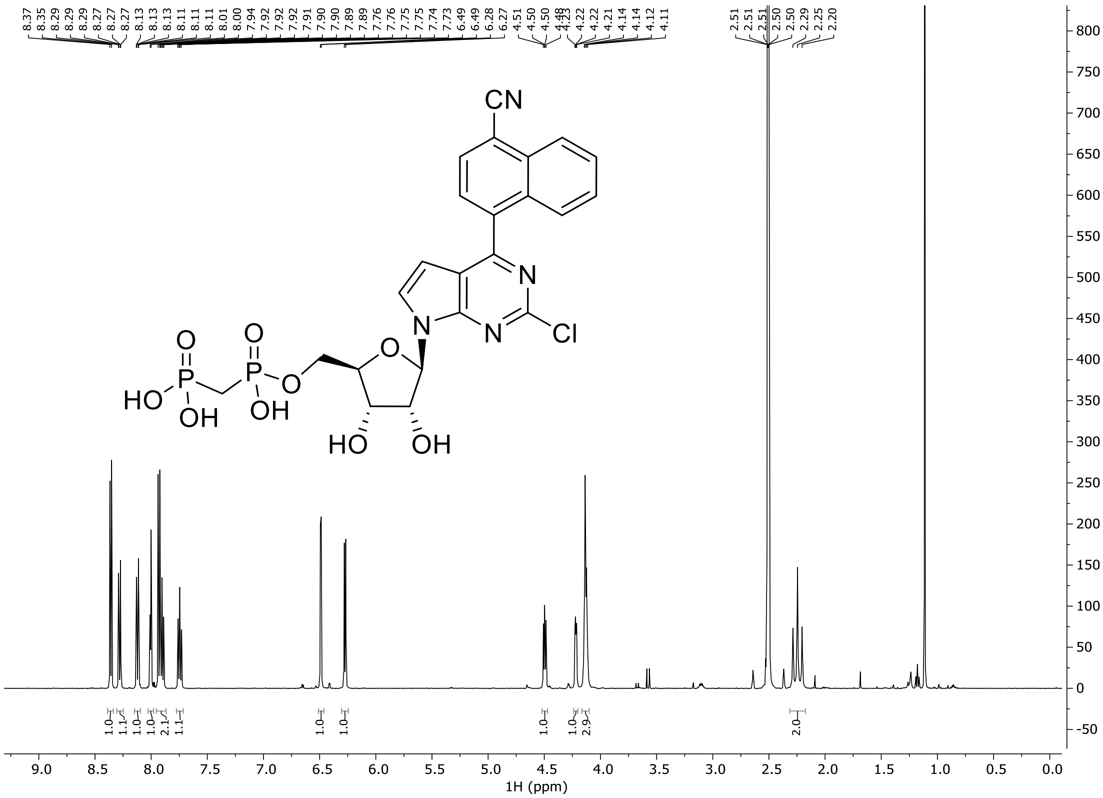

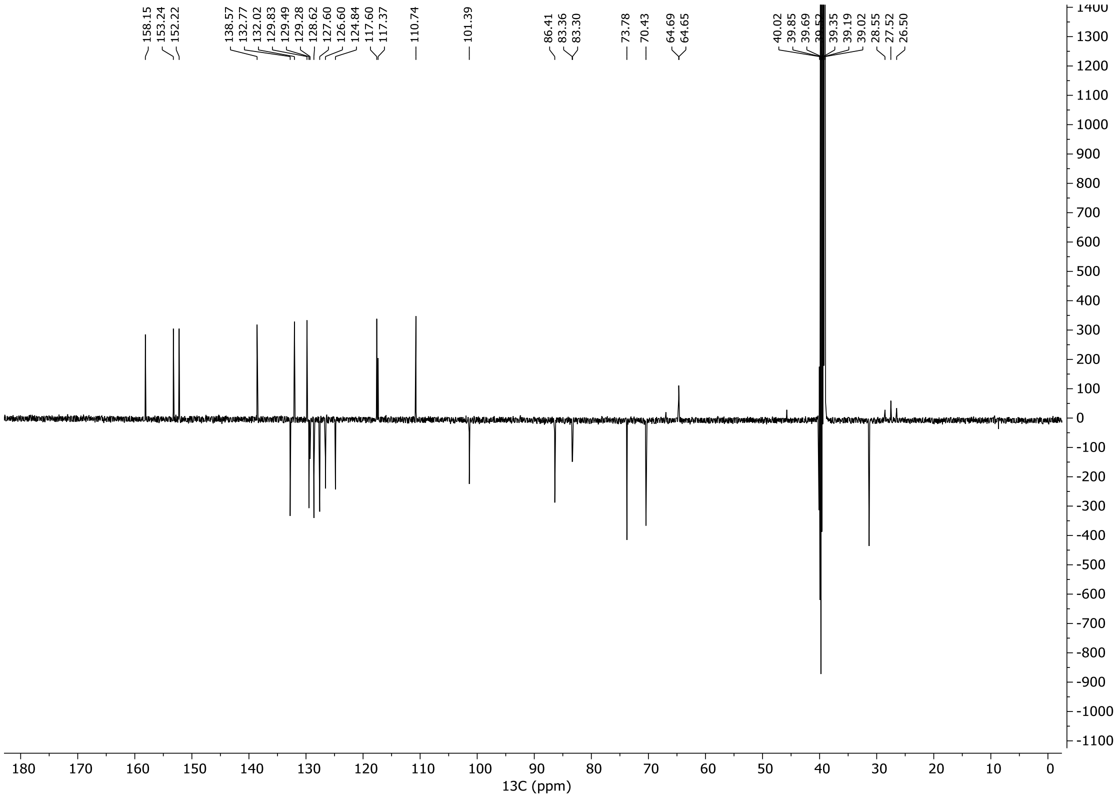

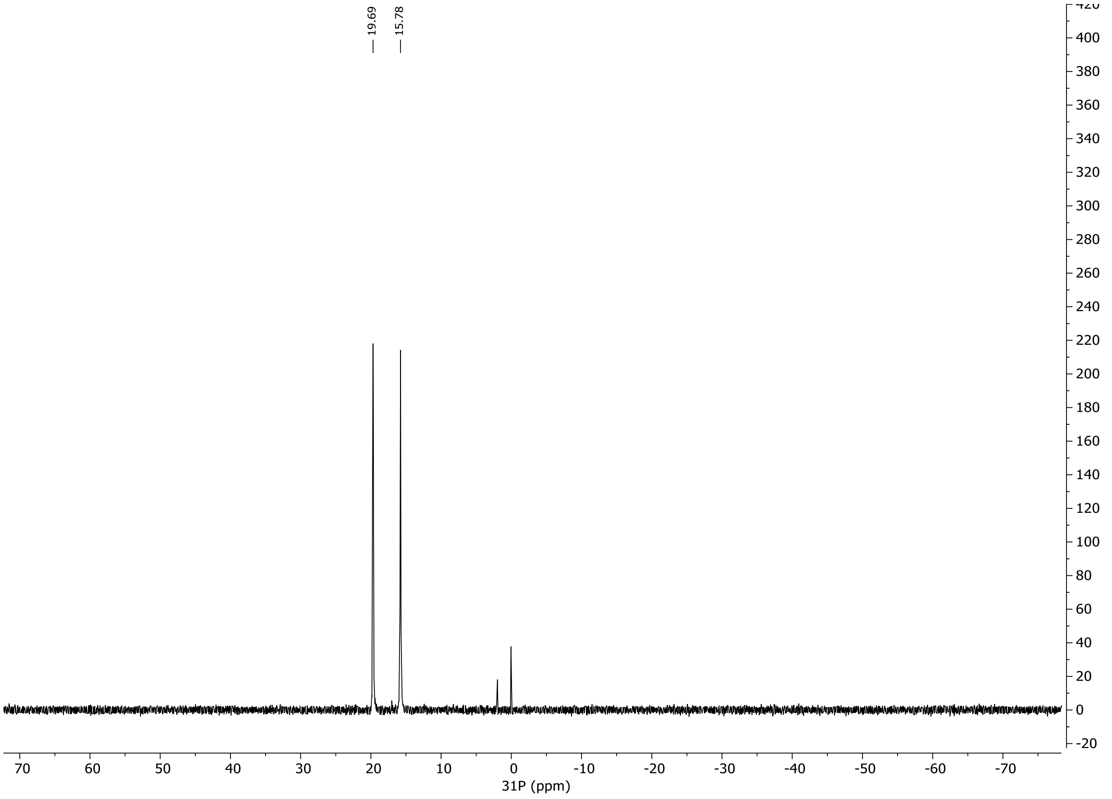

NMR spectra of compound **7B.35**

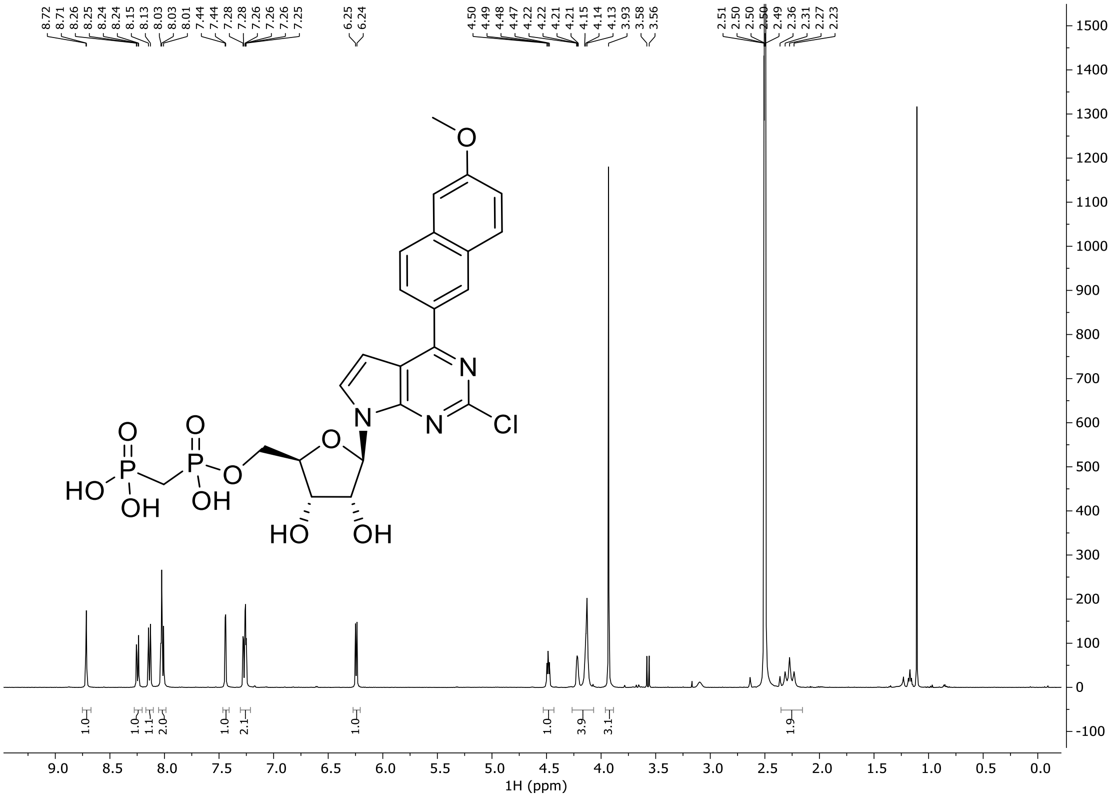

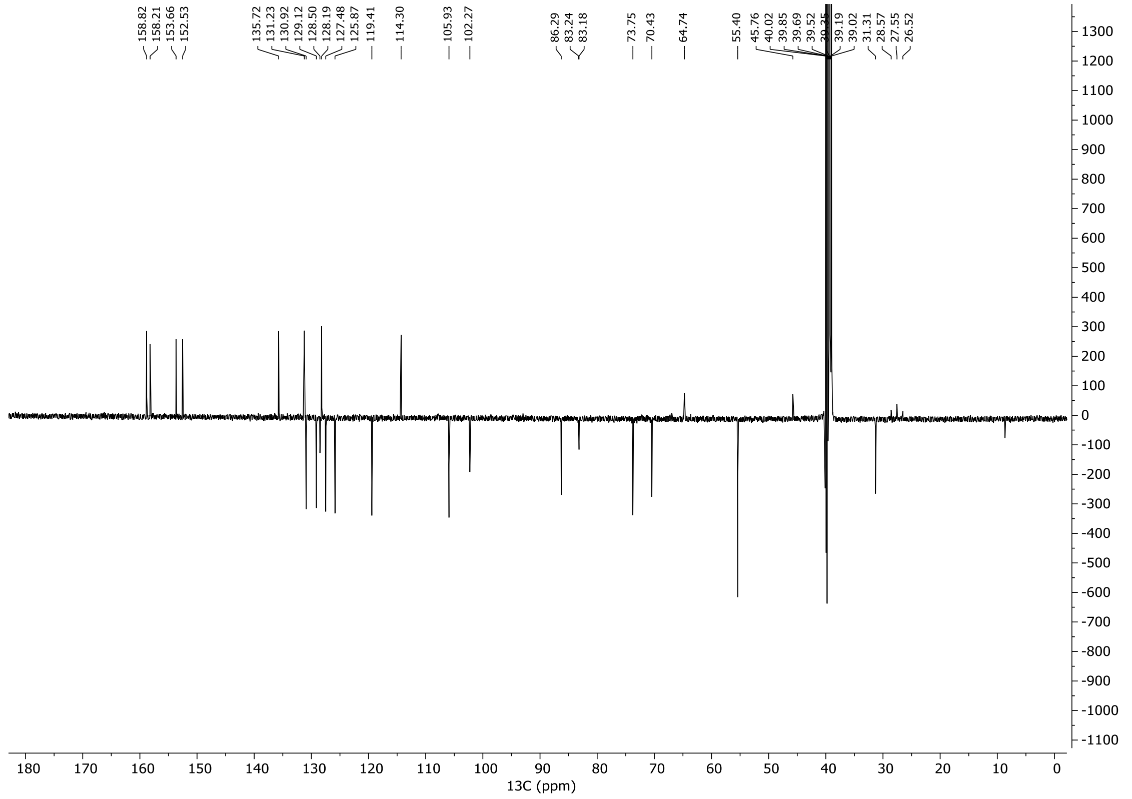
158.82
158.21
153.66
152.53
135.72
131.23
130.92
129.12
128.50
128.19
127.48
125.87
119.41
114.30
105.93
102.27
86.29
83.24
83.18
73.75
70.43
64.74
55.40
45.76
40.02
39.85
39.69
39.52
39.19
39.02
31.31
28.57
27.55
26.52
13C (ppm)

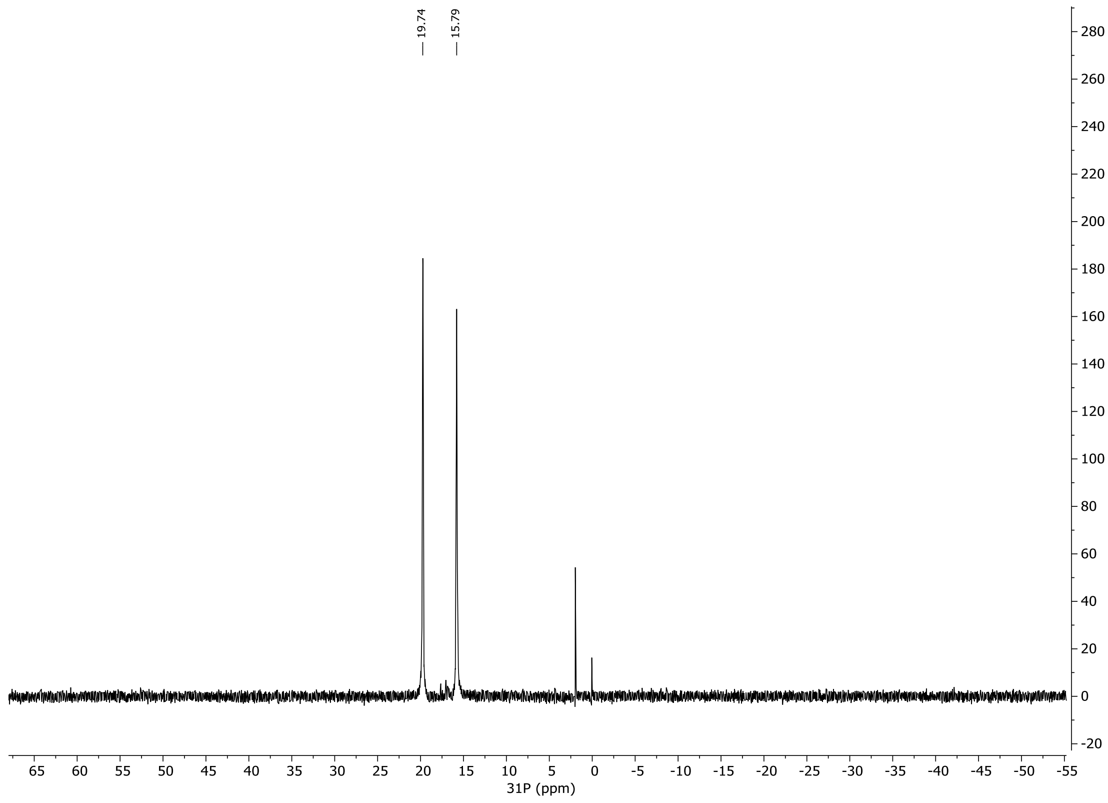
19.74
15.79
31P (ppm)

NMR spectra of compound **9**

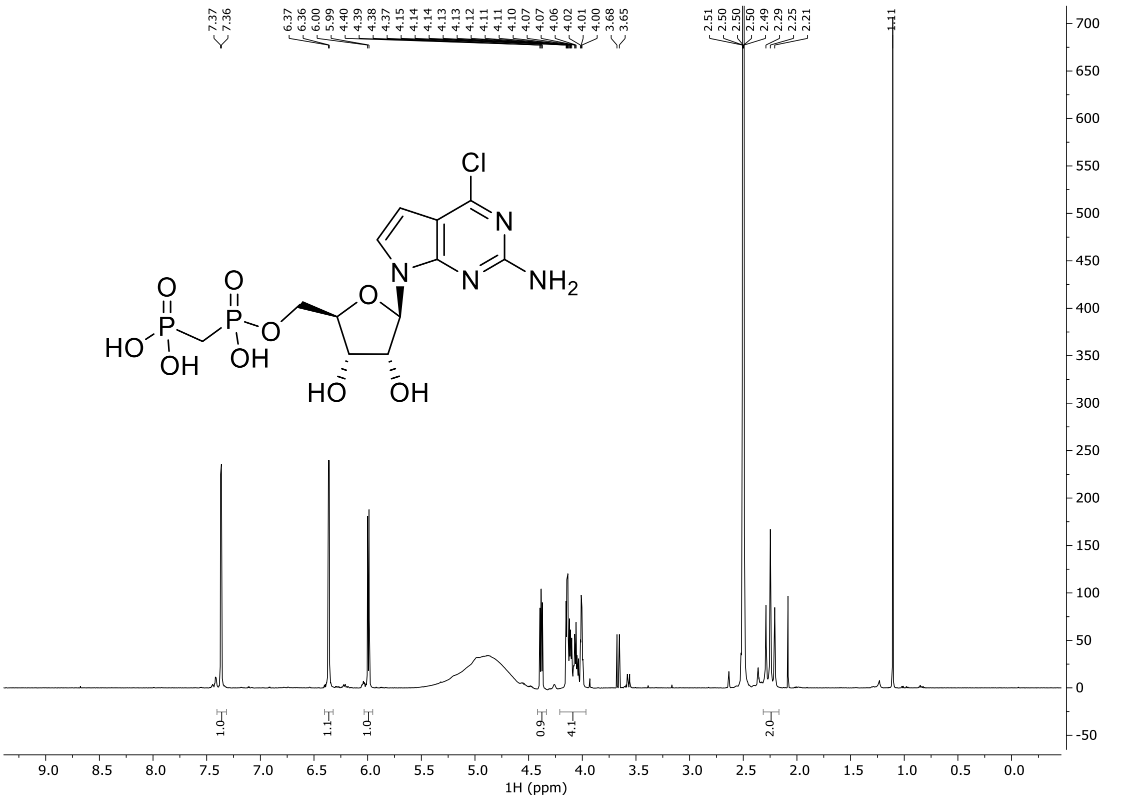

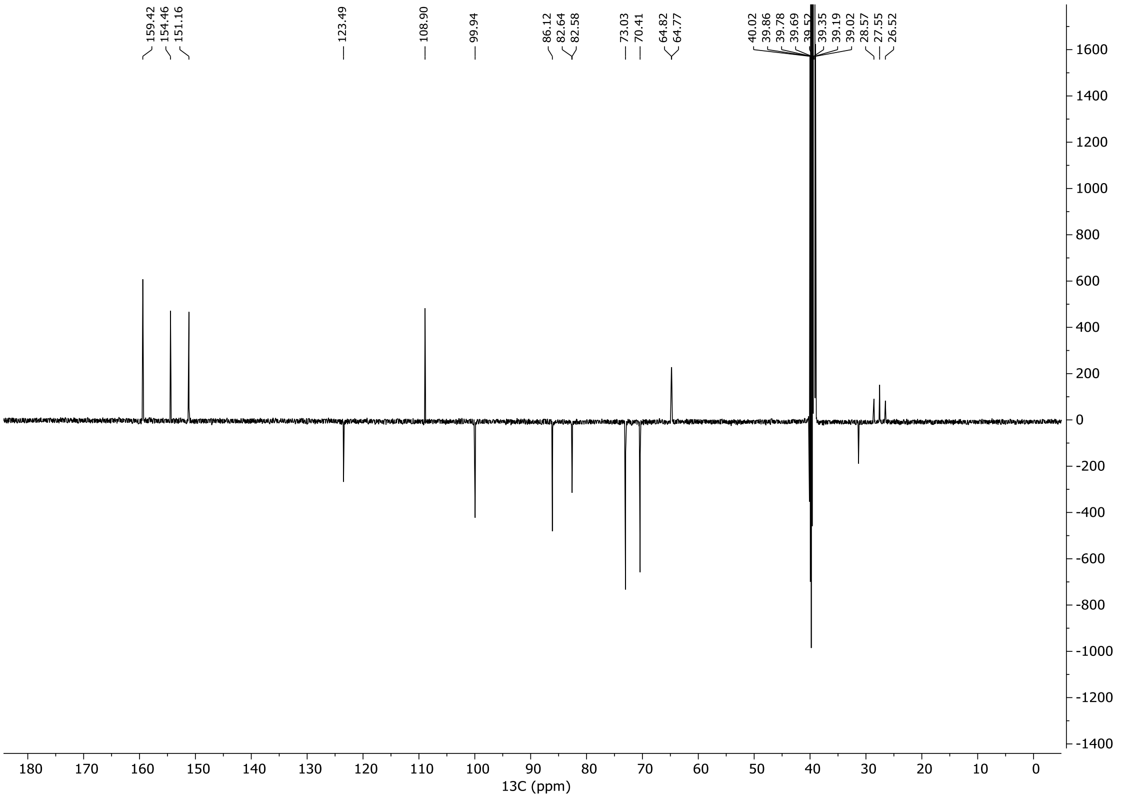

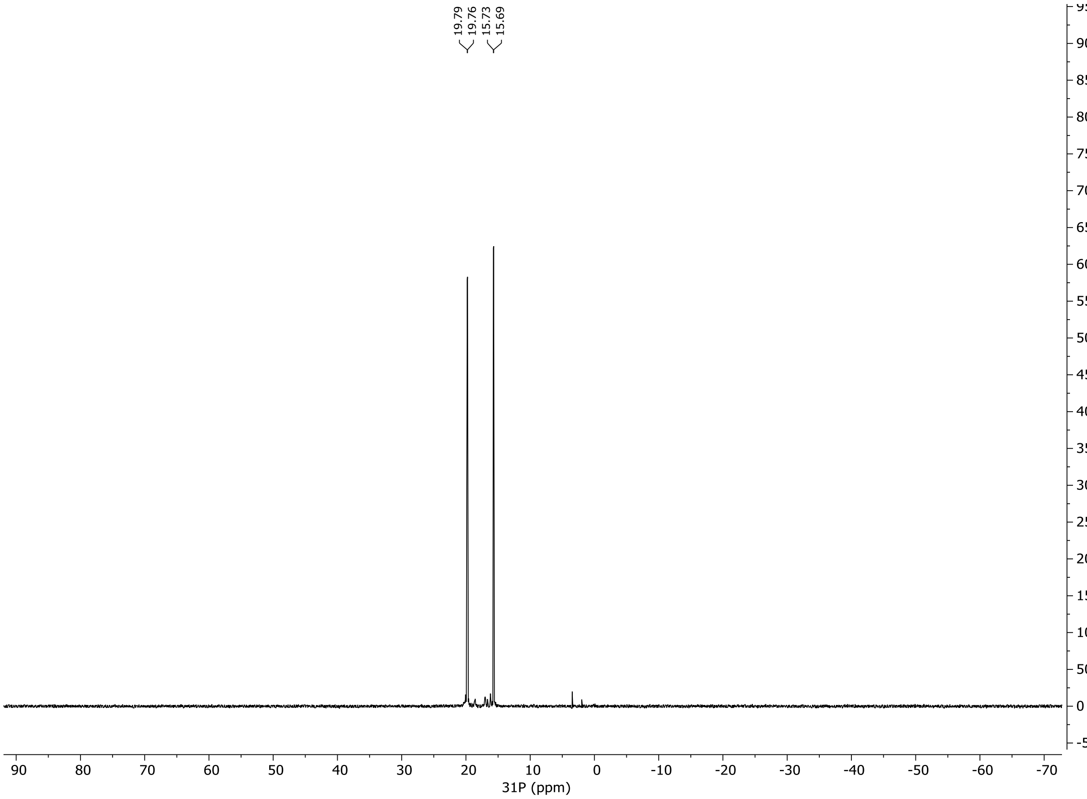

NMR spectra of compound **10C.6**

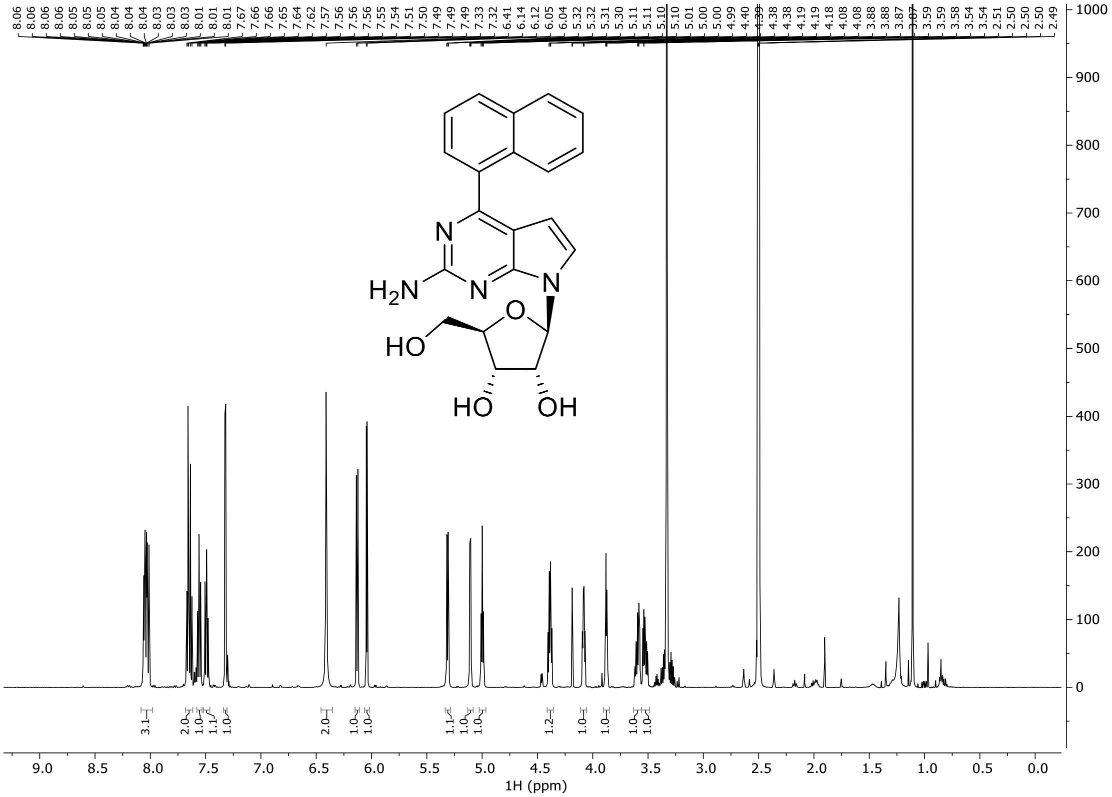

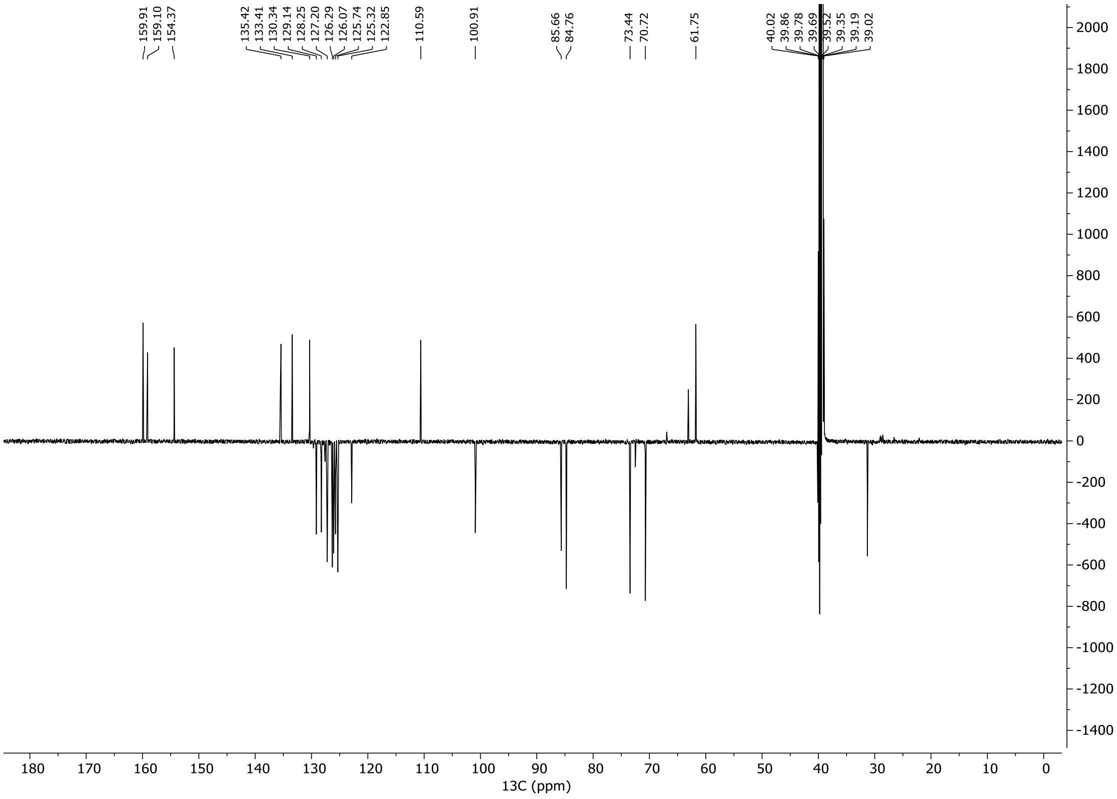

NMR spectra of compound **10C.7**

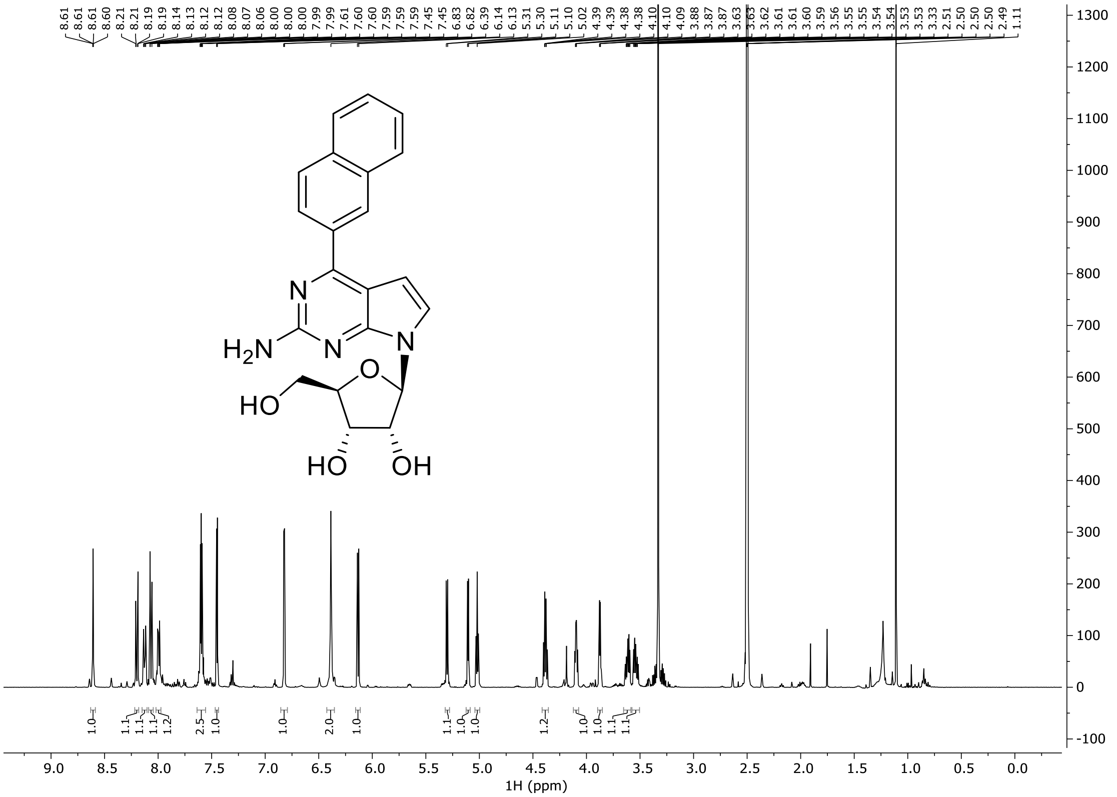

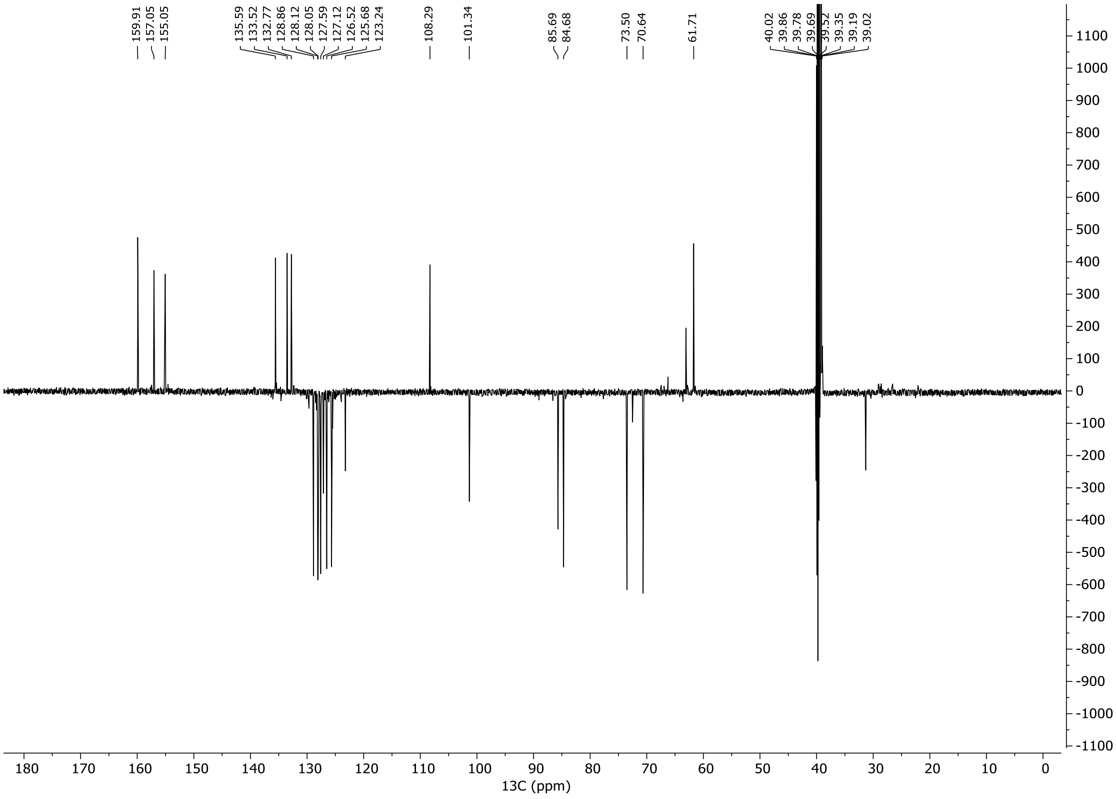

NMR spectra of compound **10C.8**

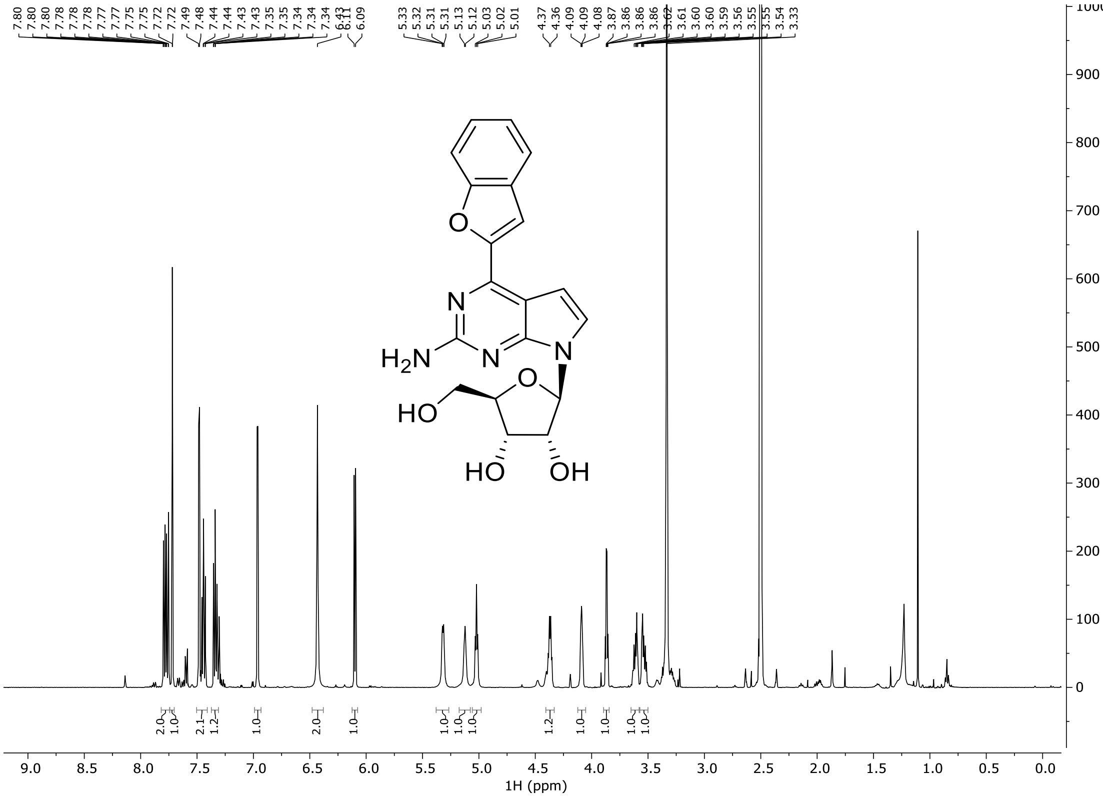

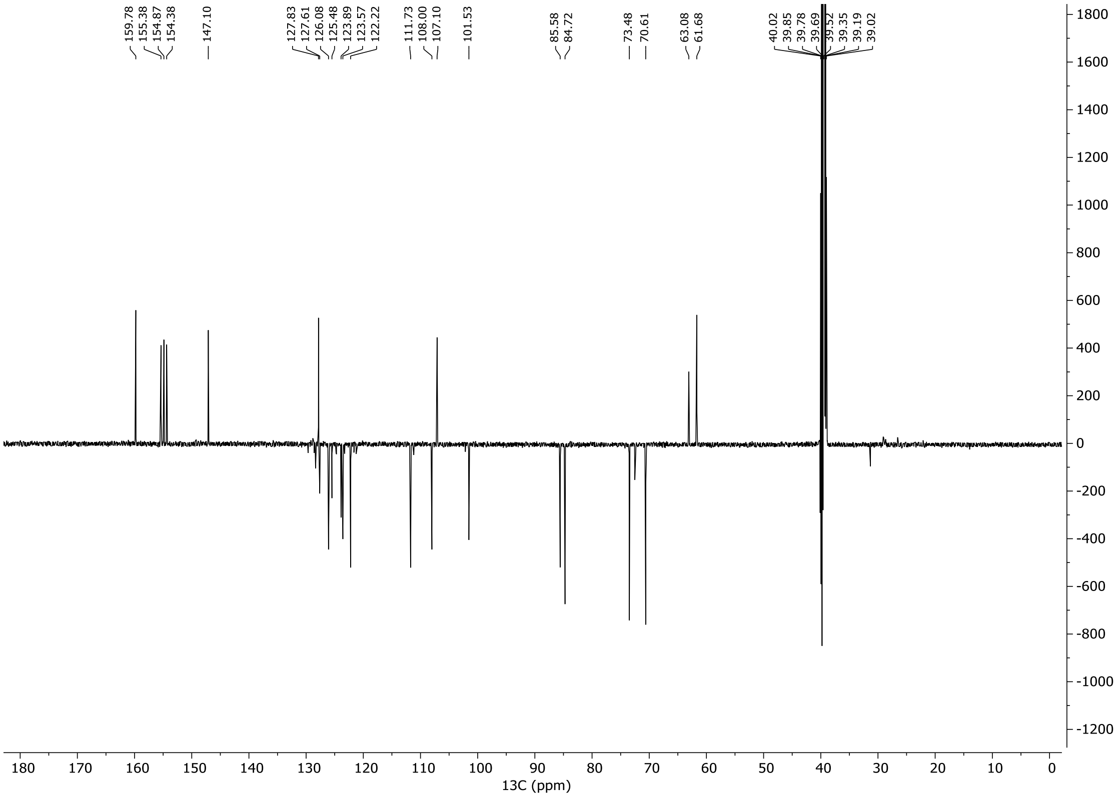

NMR spectra of compound **10C.12**

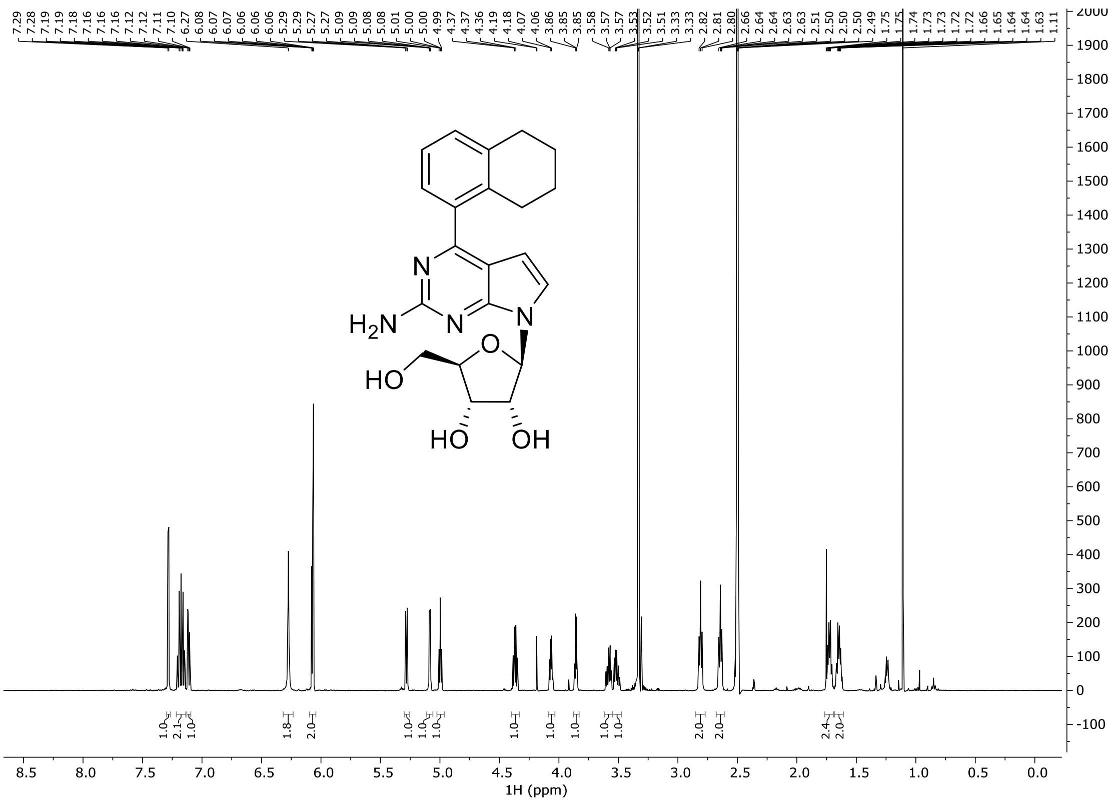

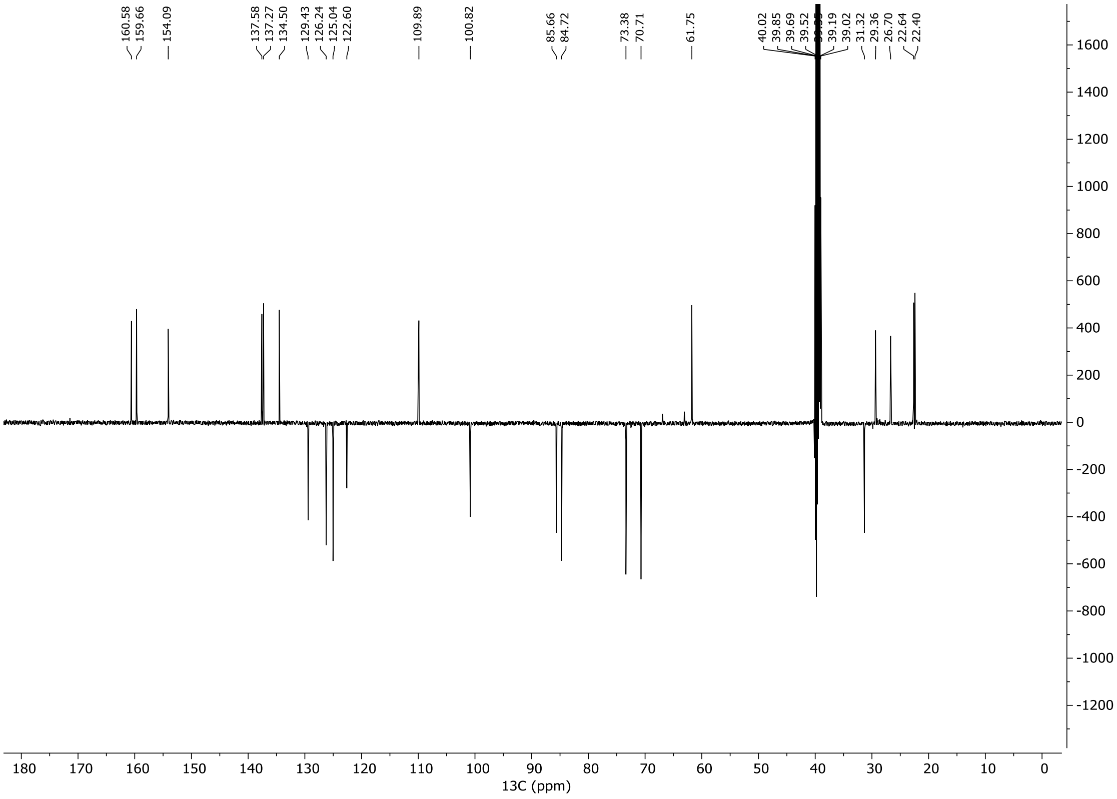

NMR spectra of compound **11C.1**

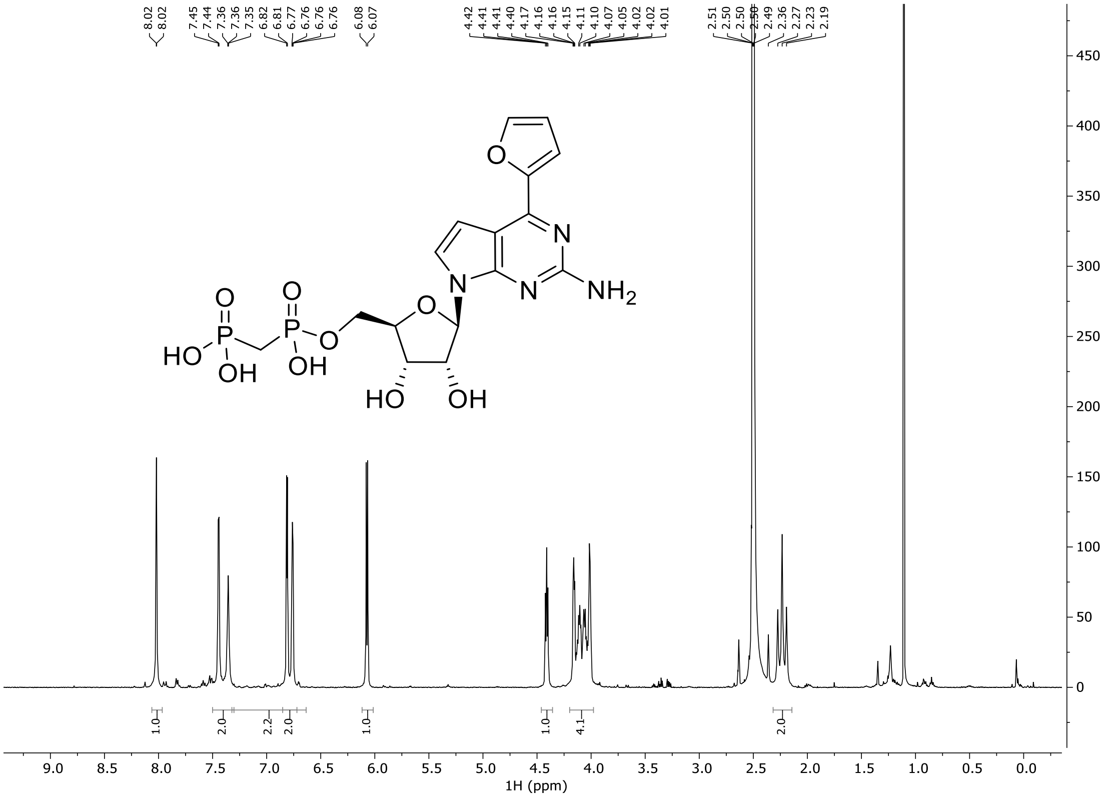

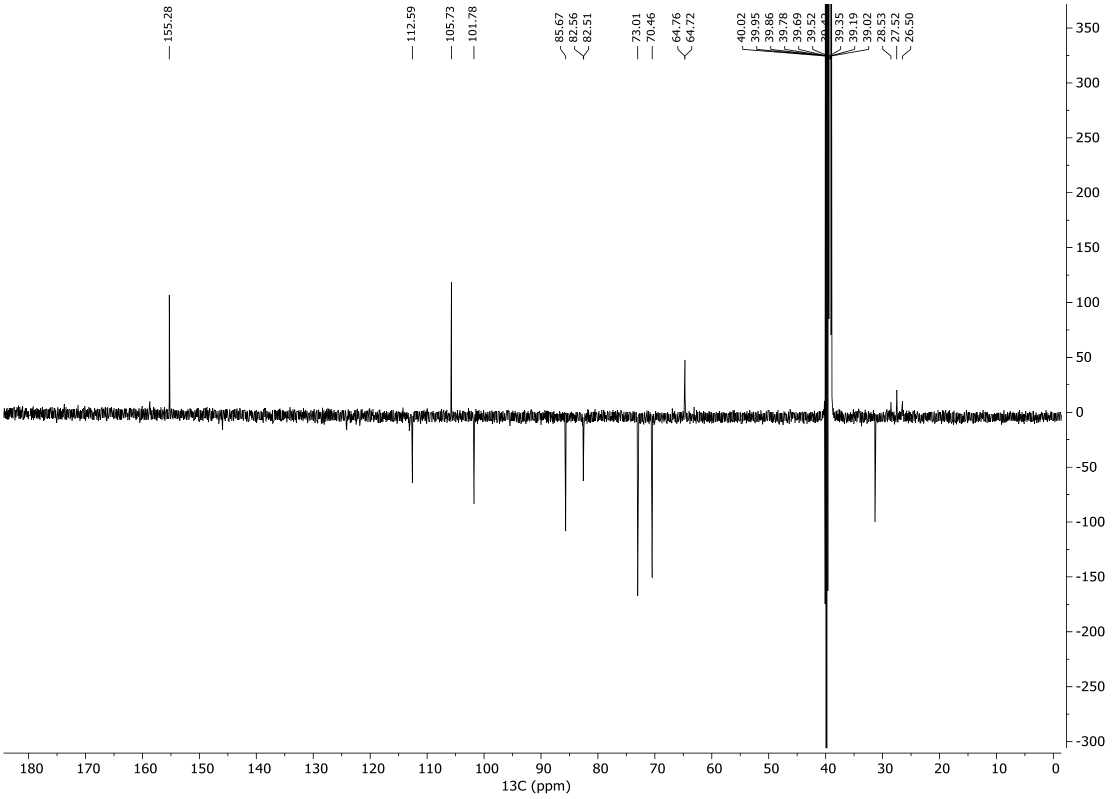
155.28
112.59
105.73
101.78
85.67
82.56
82.51
73.01
70.46
64.76
64.72
40.02
39.95
39.86
39.78
39.69
39.52
39.35
39.19
39.02
28.53
27.52
26.50
13C (ppm)

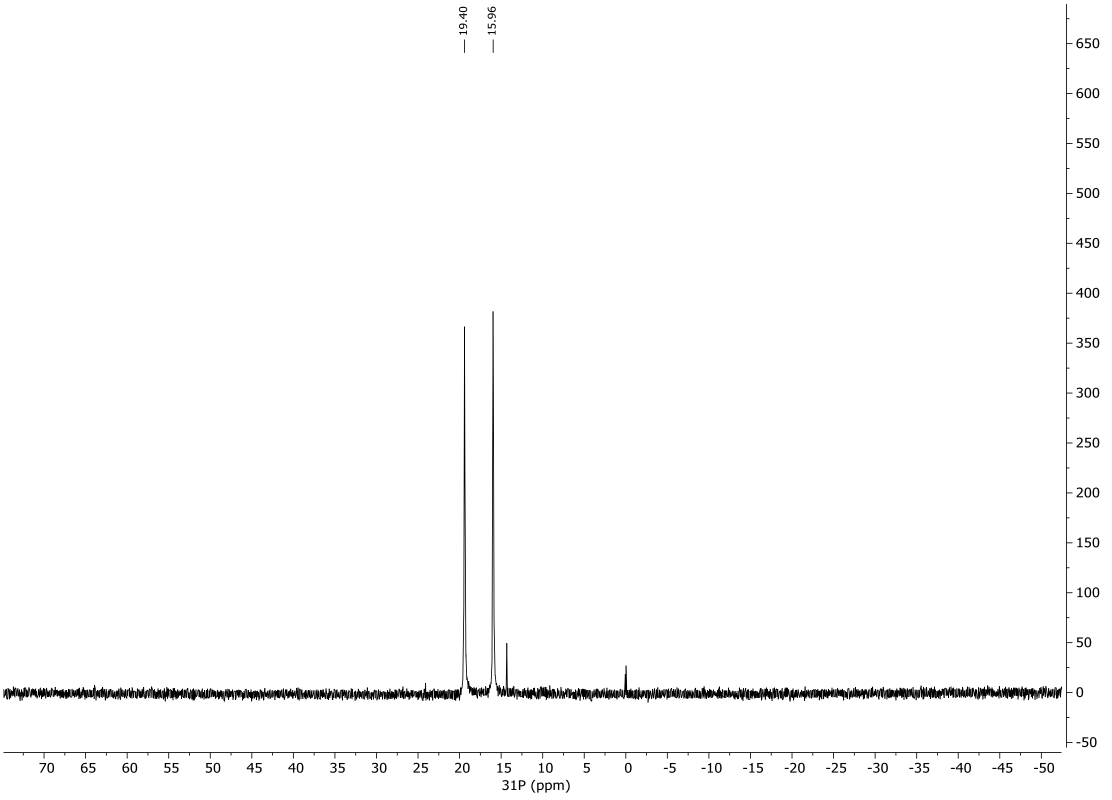
19.40
15.96
31P (ppm)

NMR spectra of compound **11C.6**

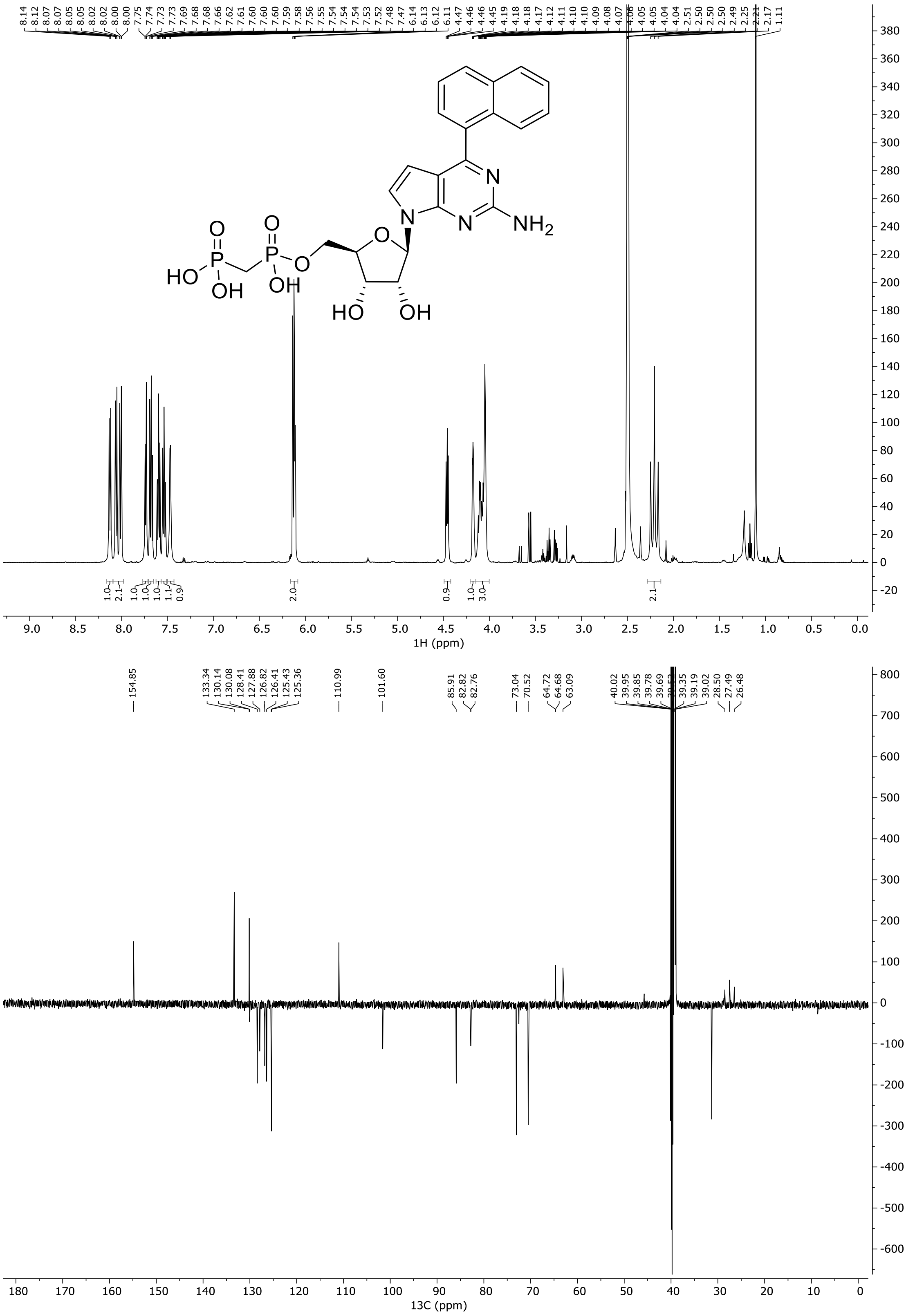

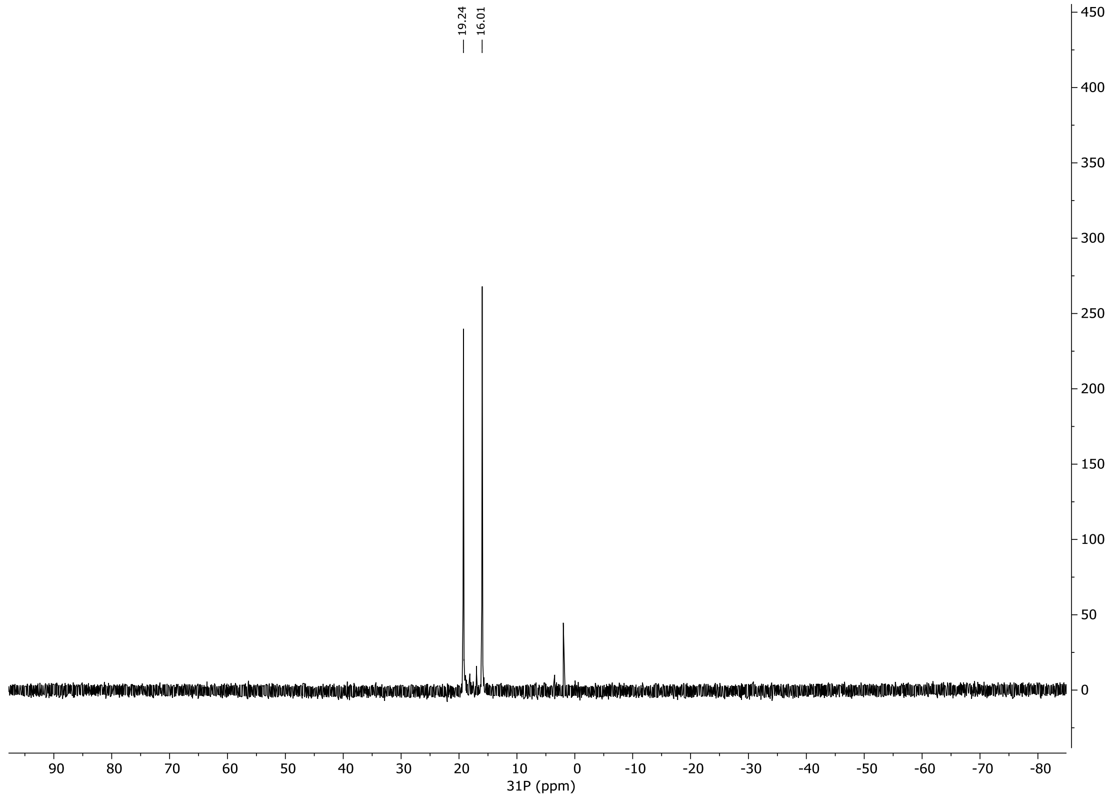

NMR spectra of compound **11C.7**

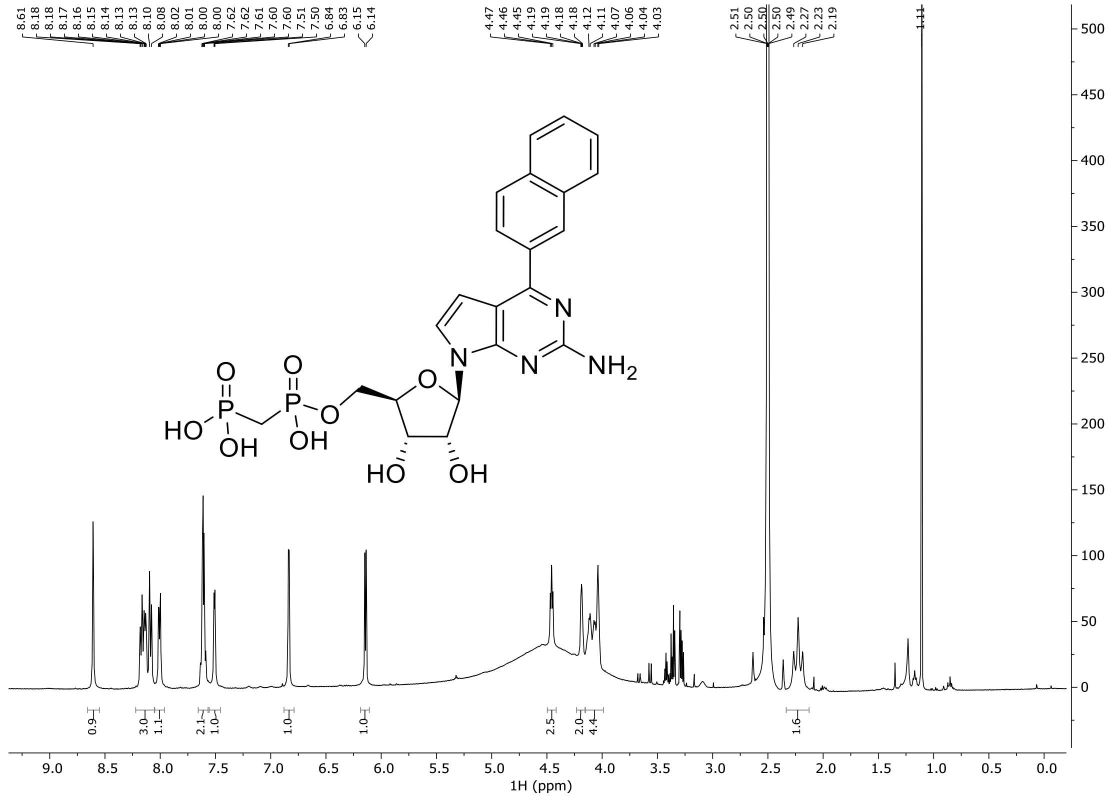

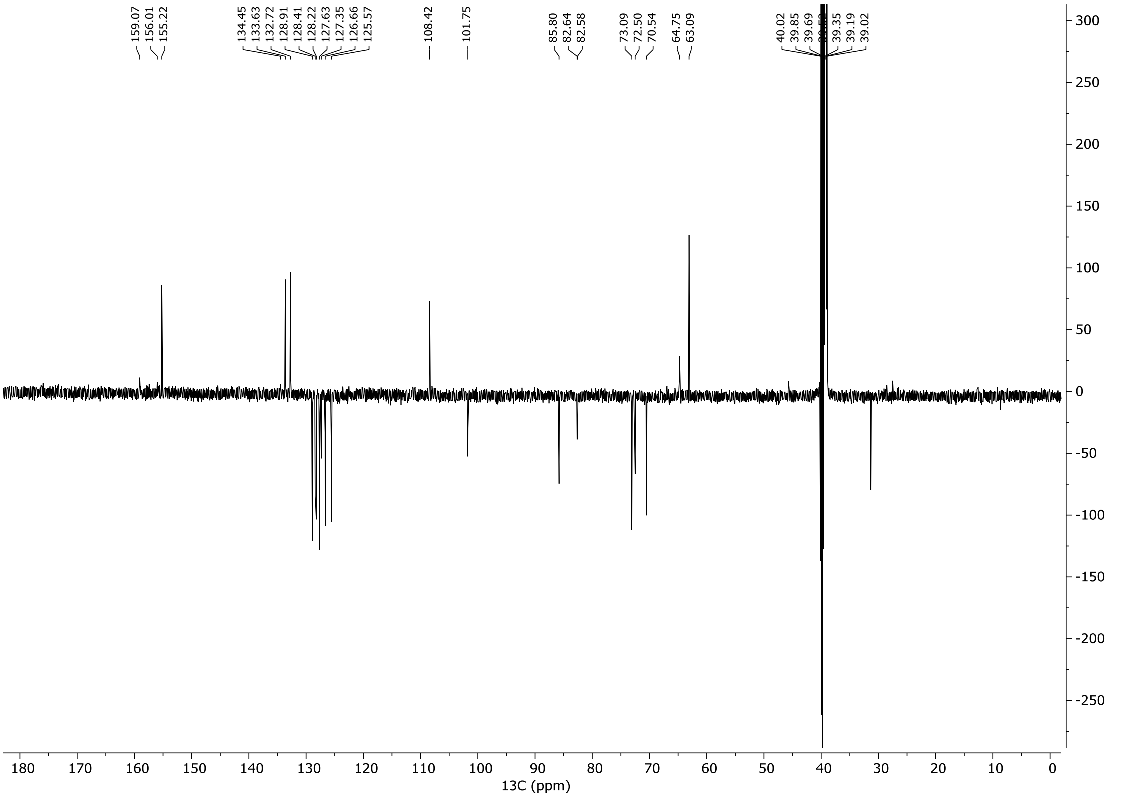
159.07
156.01
155.22
134.45
133.63
132.72
128.91
128.41
128.22
127.63
127.35
126.66
125.57
108.42
101.75
85.80
82.64
82.58
73.09
72.50
70.54
64.75
63.09
40.02
39.85
39.69
39.35
39.19
39.02
13C (ppm)

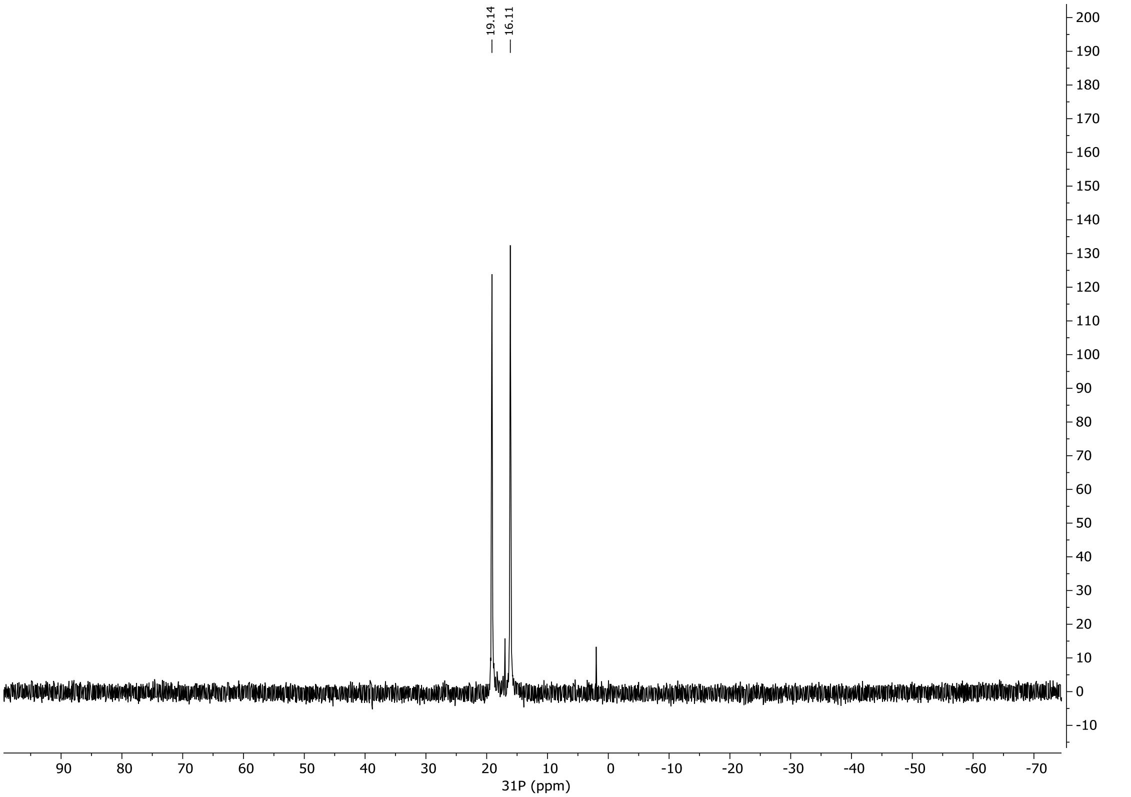
19.14
16.11
31P (ppm)

NMR spectra of compound **11C.8**

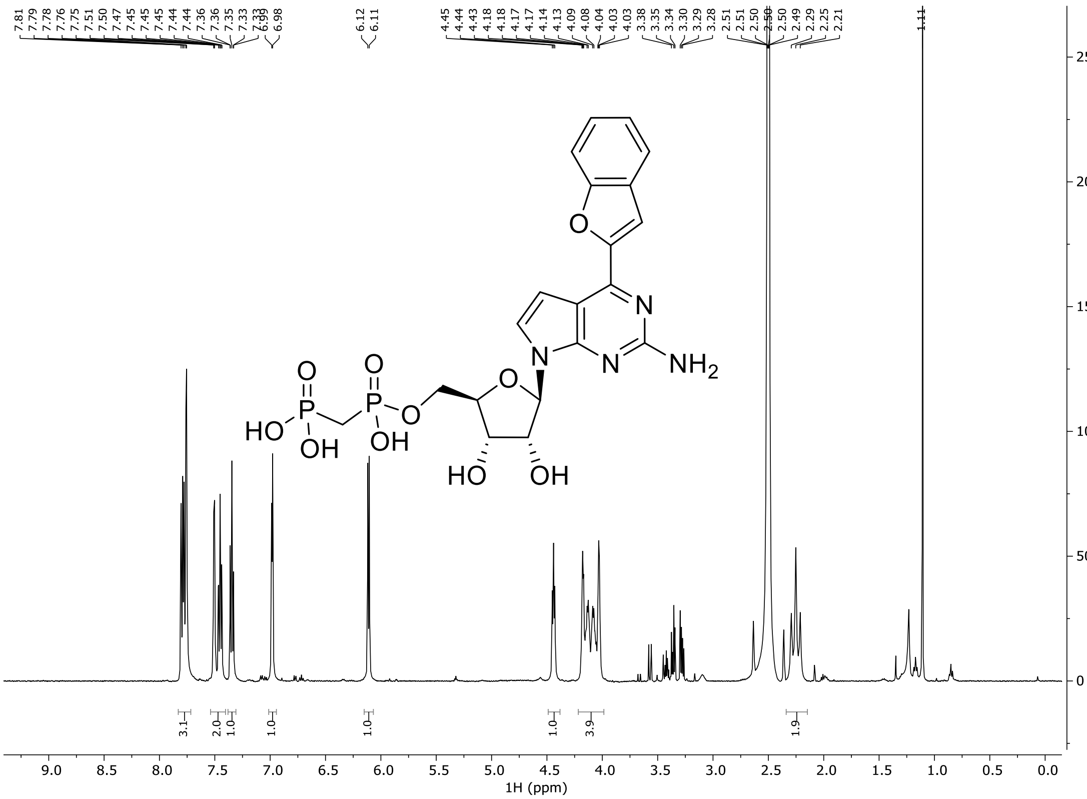

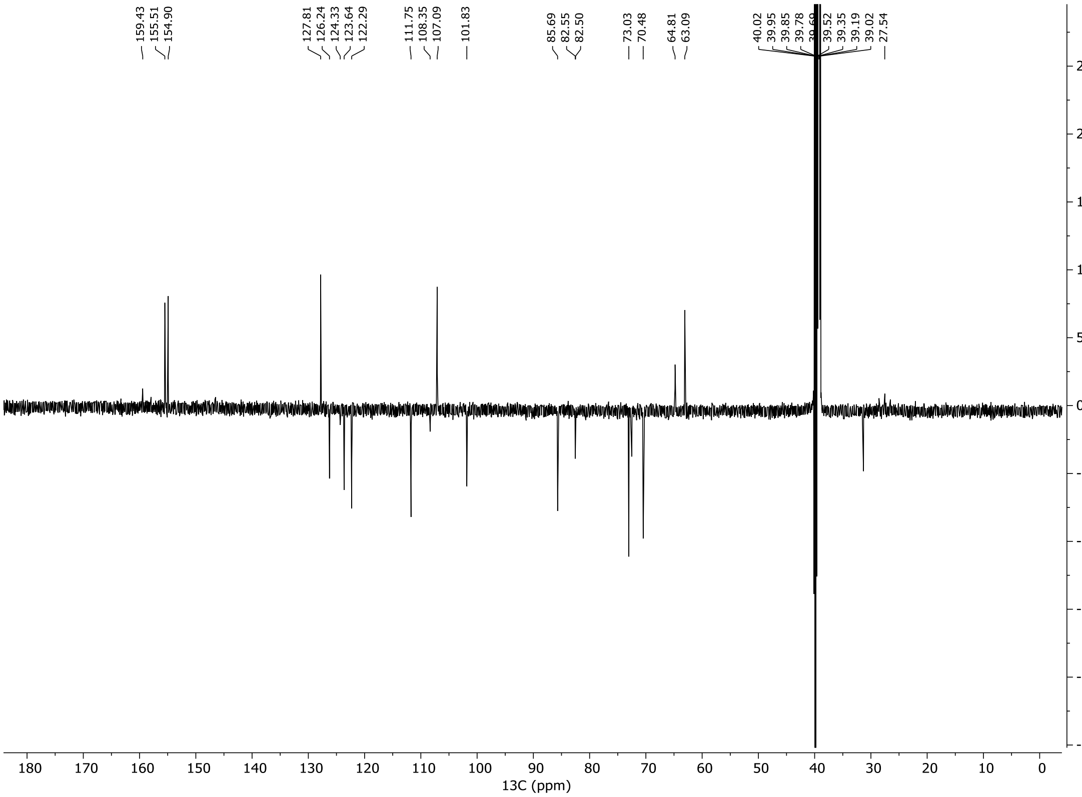

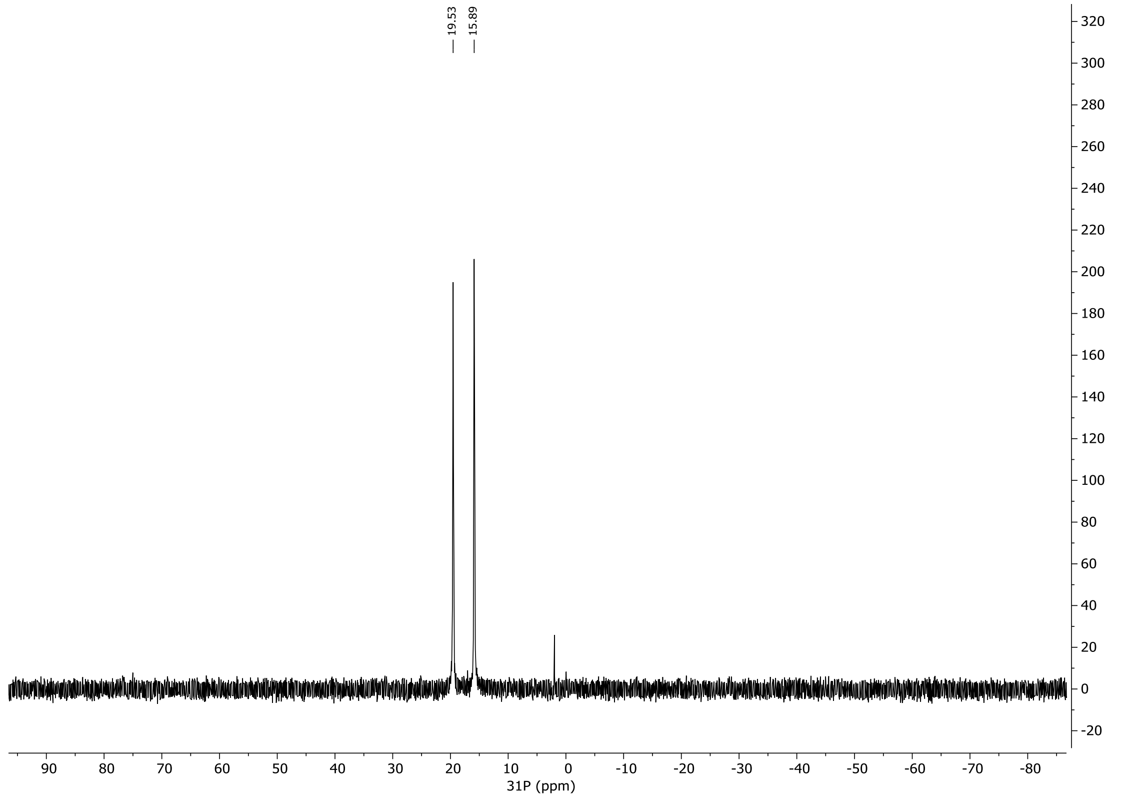

NMR spectra of compound **11C.12**

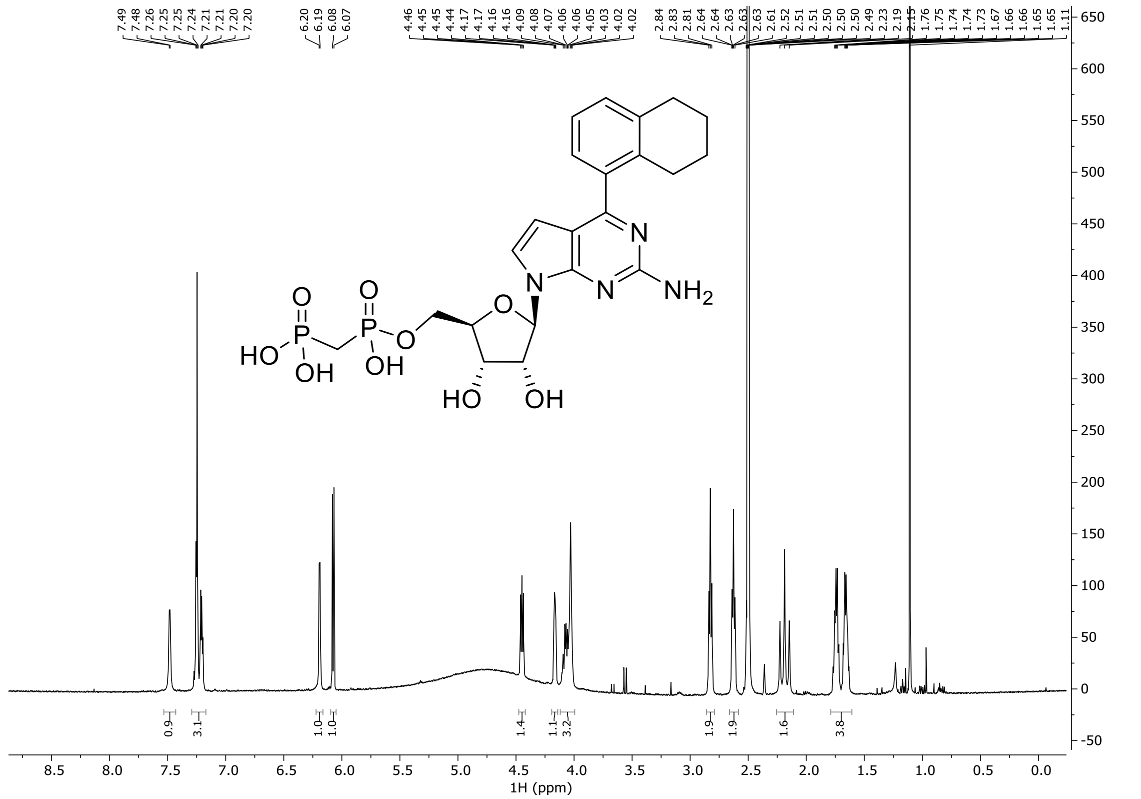

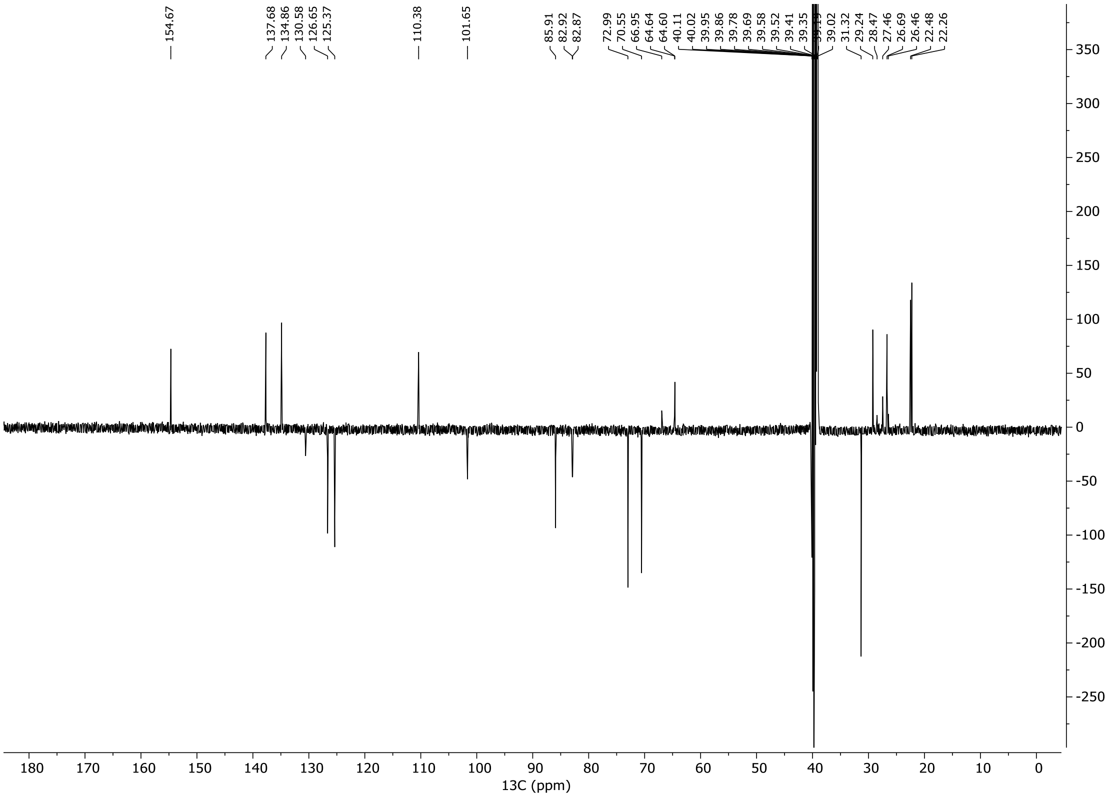
154.67
137.68
134.86
130.58
126.65
125.37
110.38
101.65
85.91
82.92
82.87
72.99
70.55
66.95
64.64
64.60
40.11
40.02
39.95
39.86
39.78
39.69
39.58
39.52
39.41
39.35
39.02
31.32
29.24
28.47
27.46
26.69
26.46
22.48
22.26
13C (ppm)

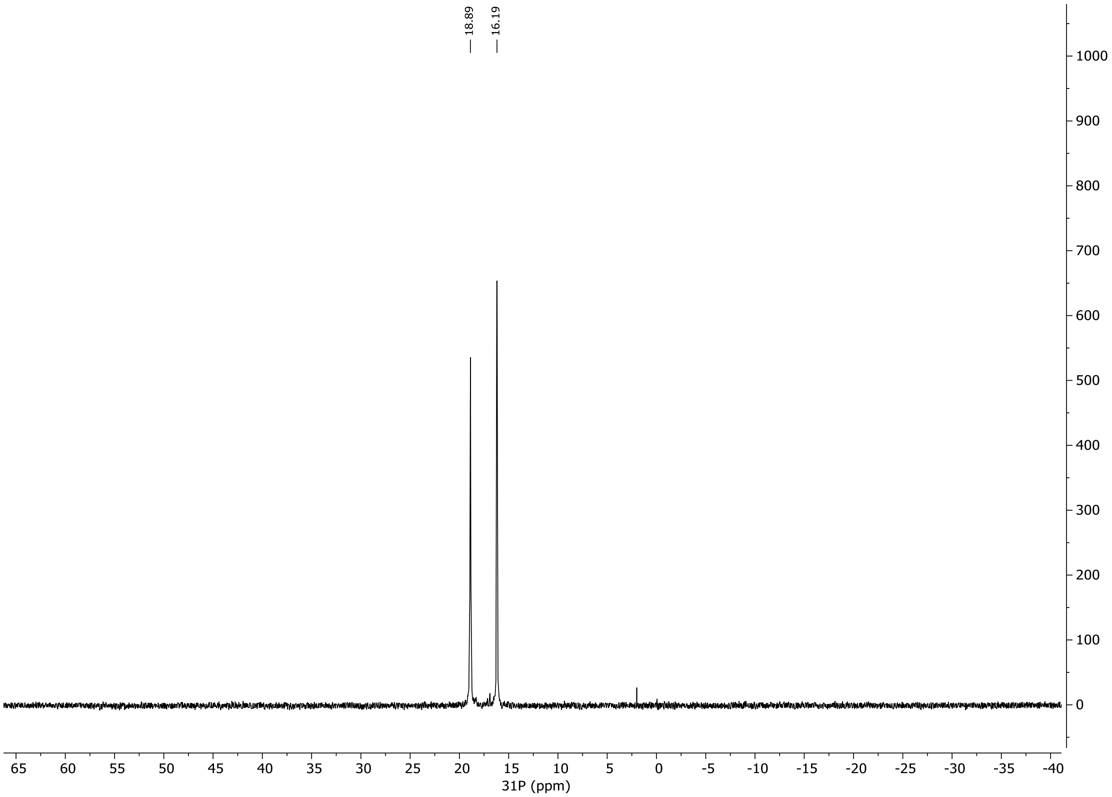
18.89
16.19
31P (ppm)

NMR spectra of compound **13D.6**

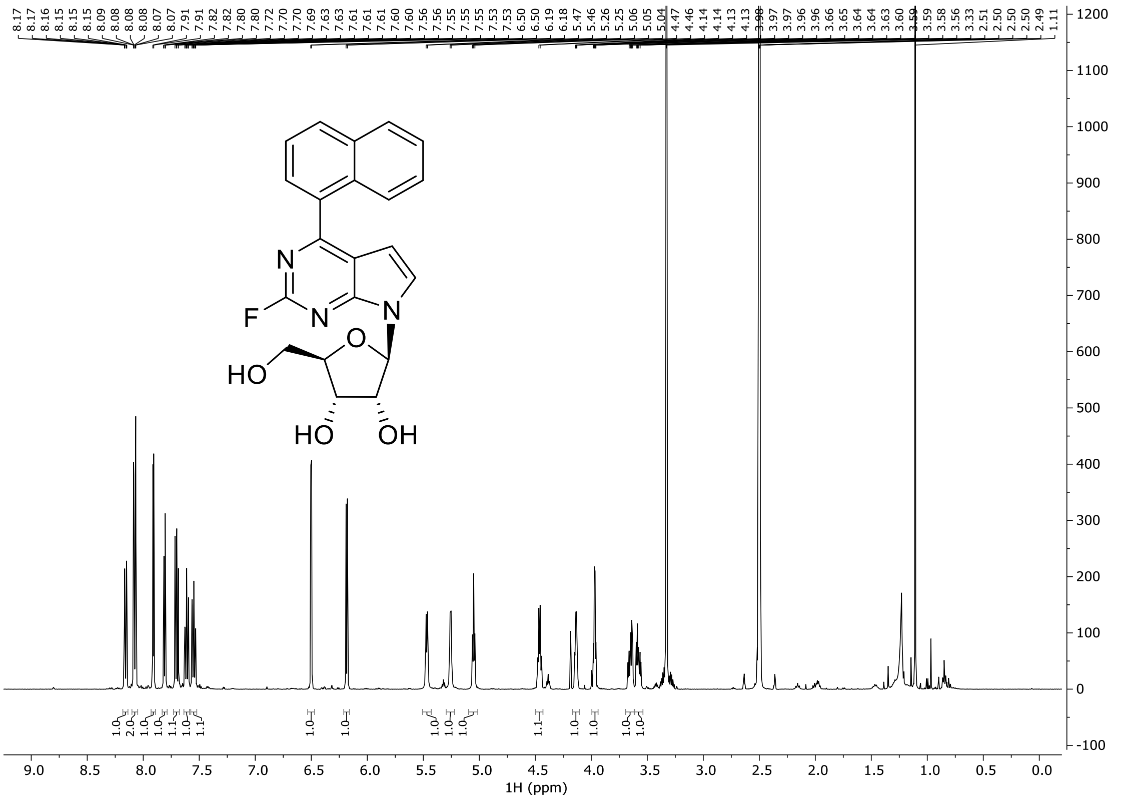

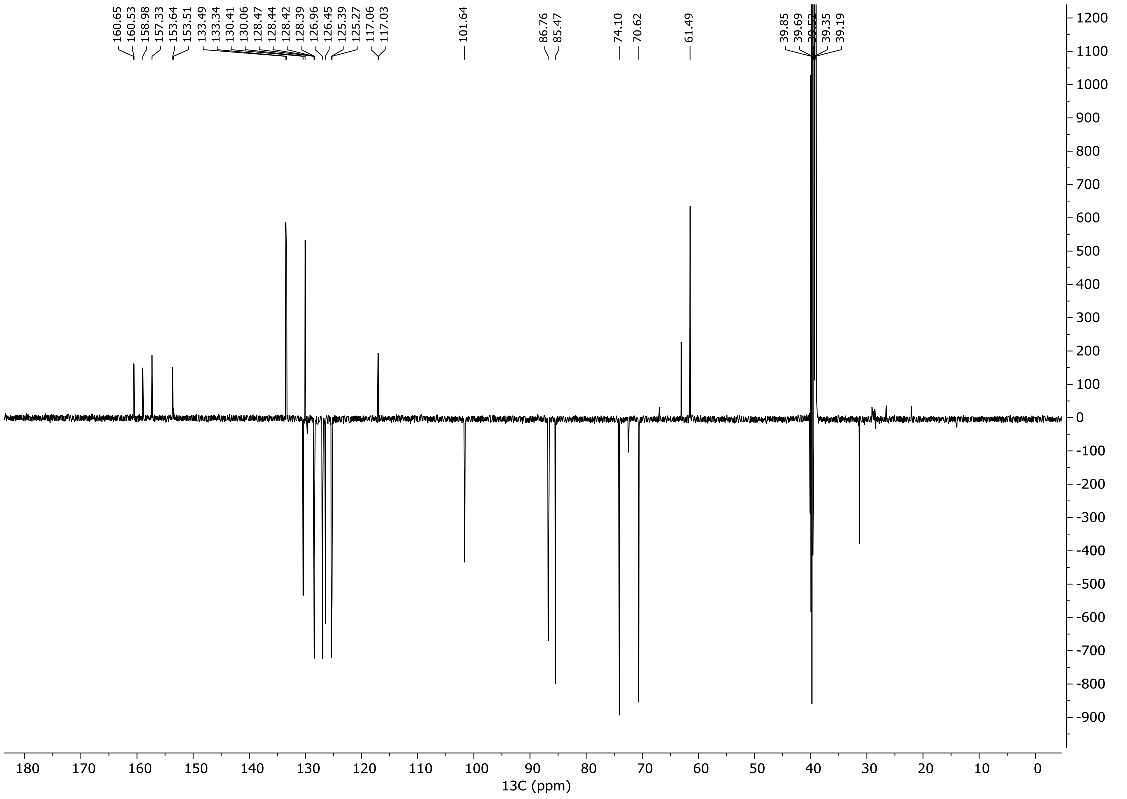

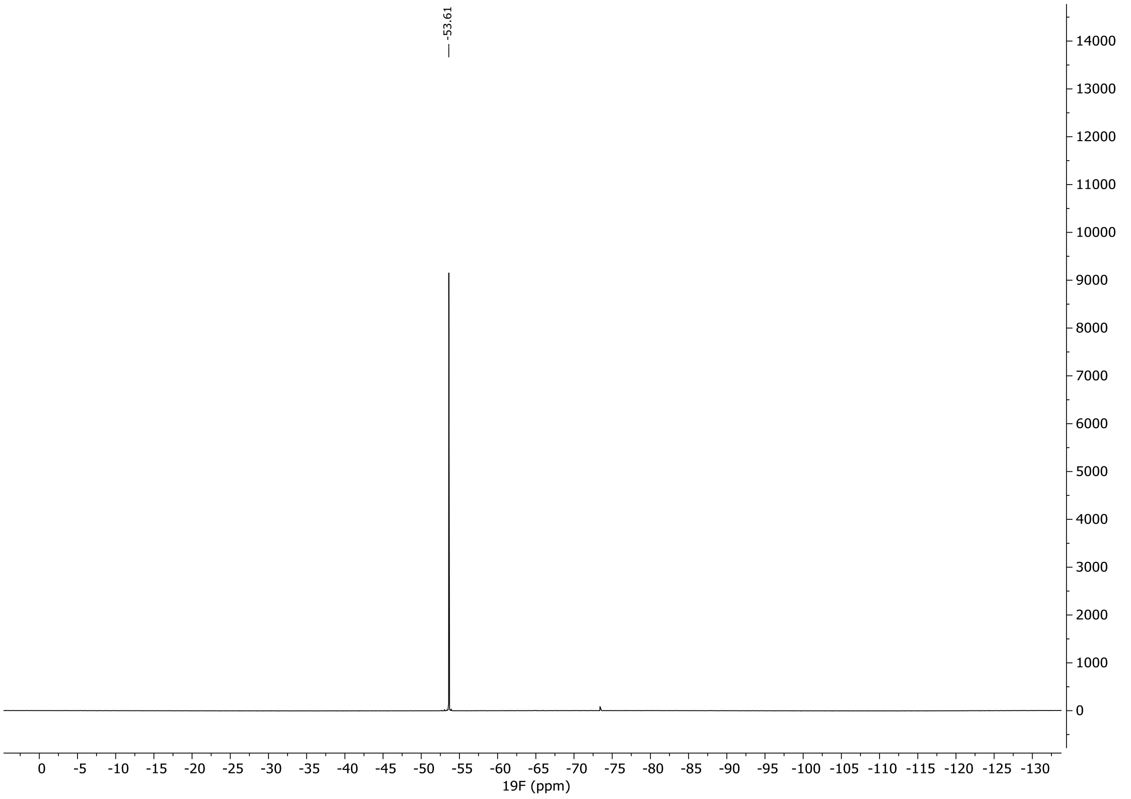

NMR spectra of compound **13D.7**

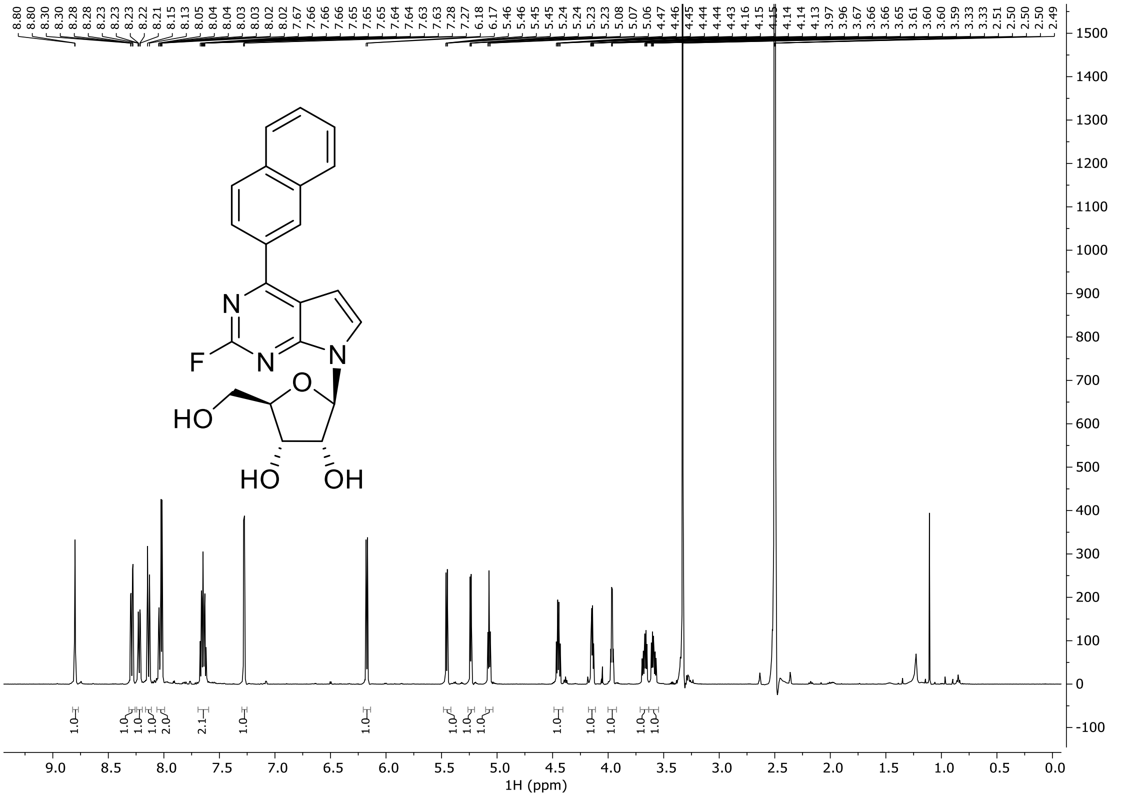

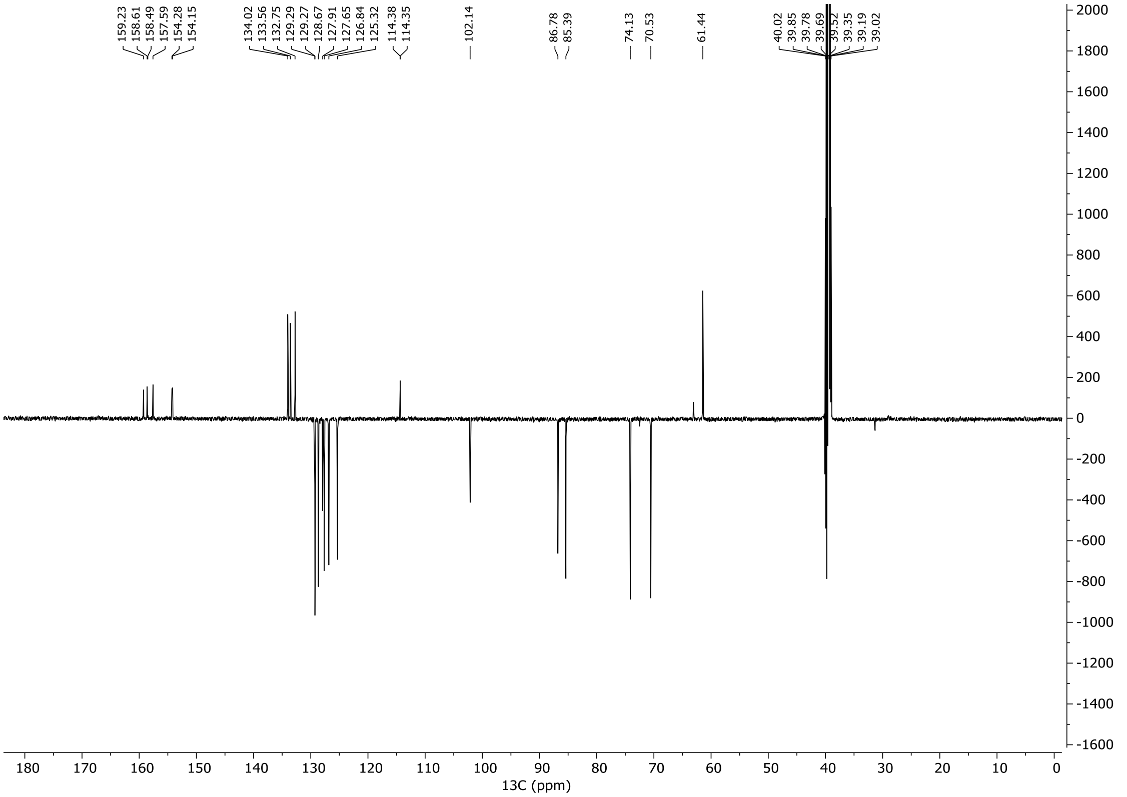
159.23
158.61
158.49
157.59
154.28
154.15
134.02
133.56
132.75
129.29
129.27
128.67
127.91
127.65
126.84
125.32
114.38
114.35
102.14
86.78
85.39
74.13
70.53
61.44
40.02
39.85
39.78
39.69
39.52
39.35
39.19
39.02
13C (ppm)

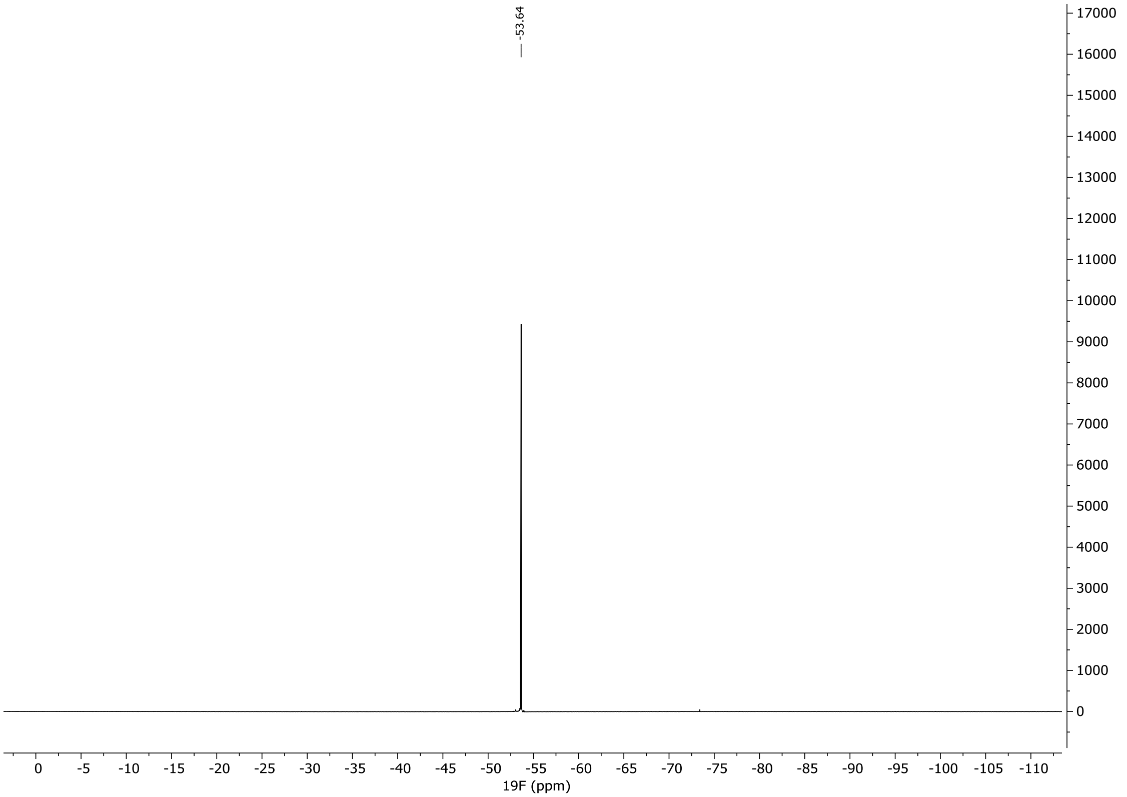
-53.64
19F (ppm)

NMR spectra of compound **13D.8**

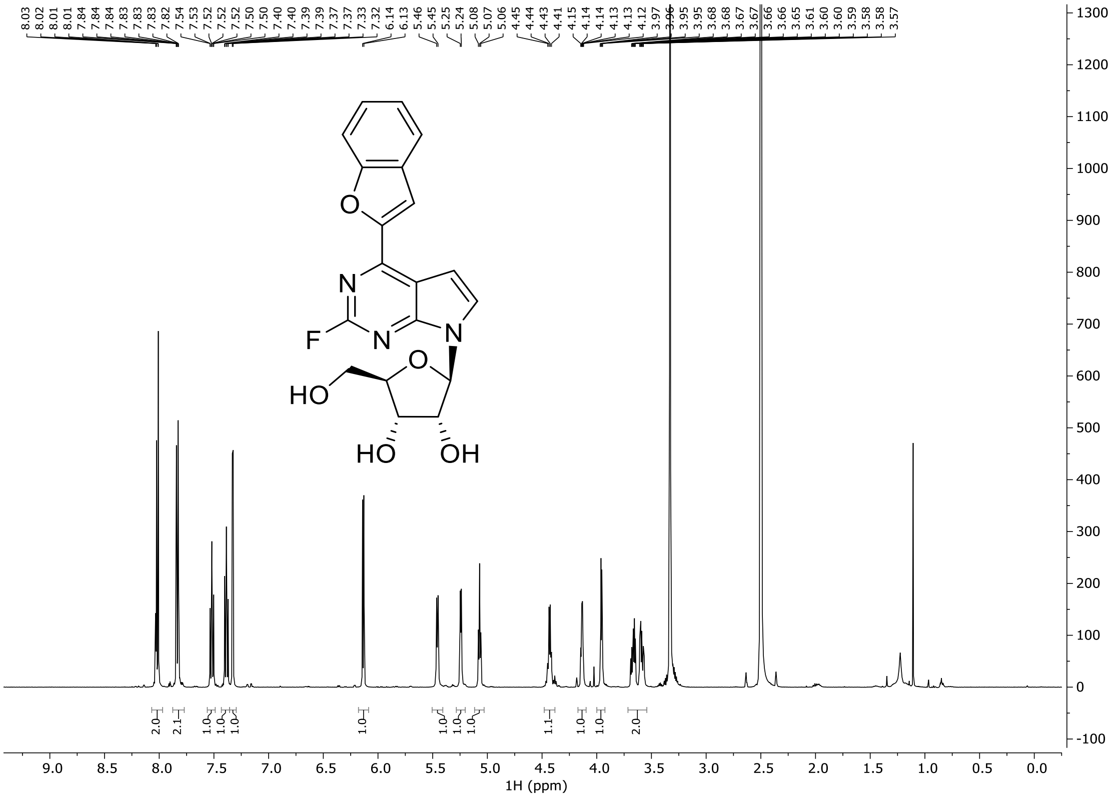

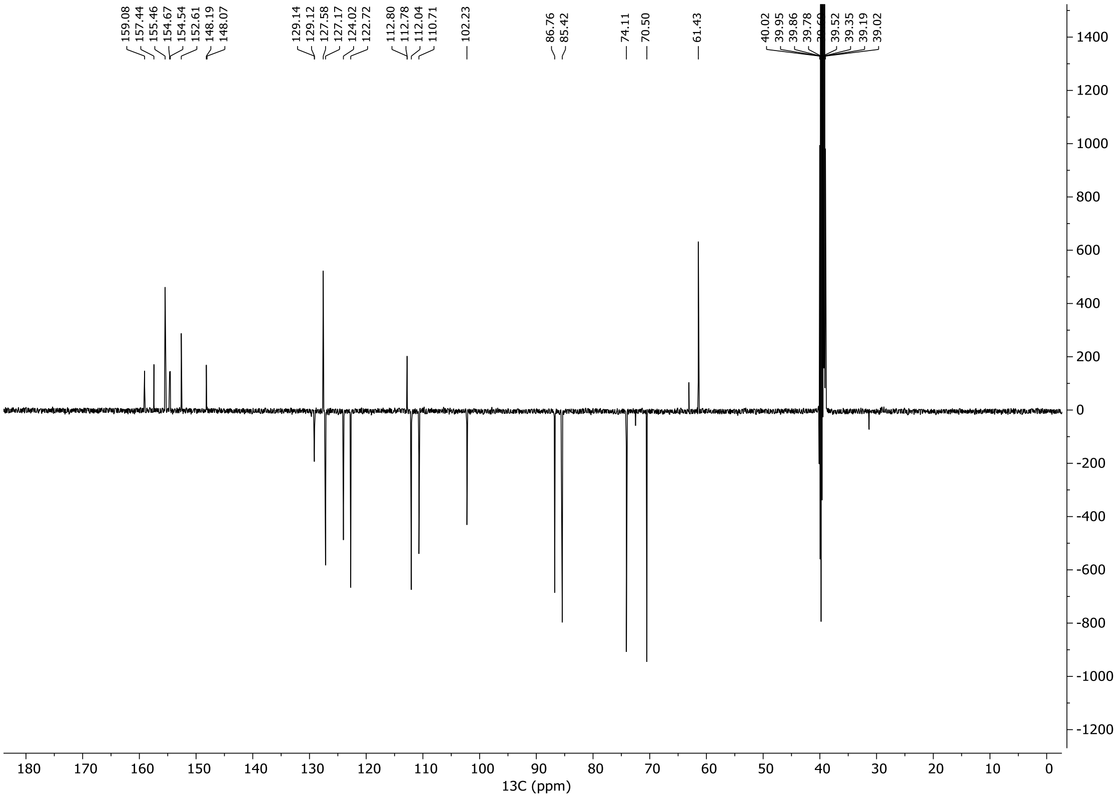

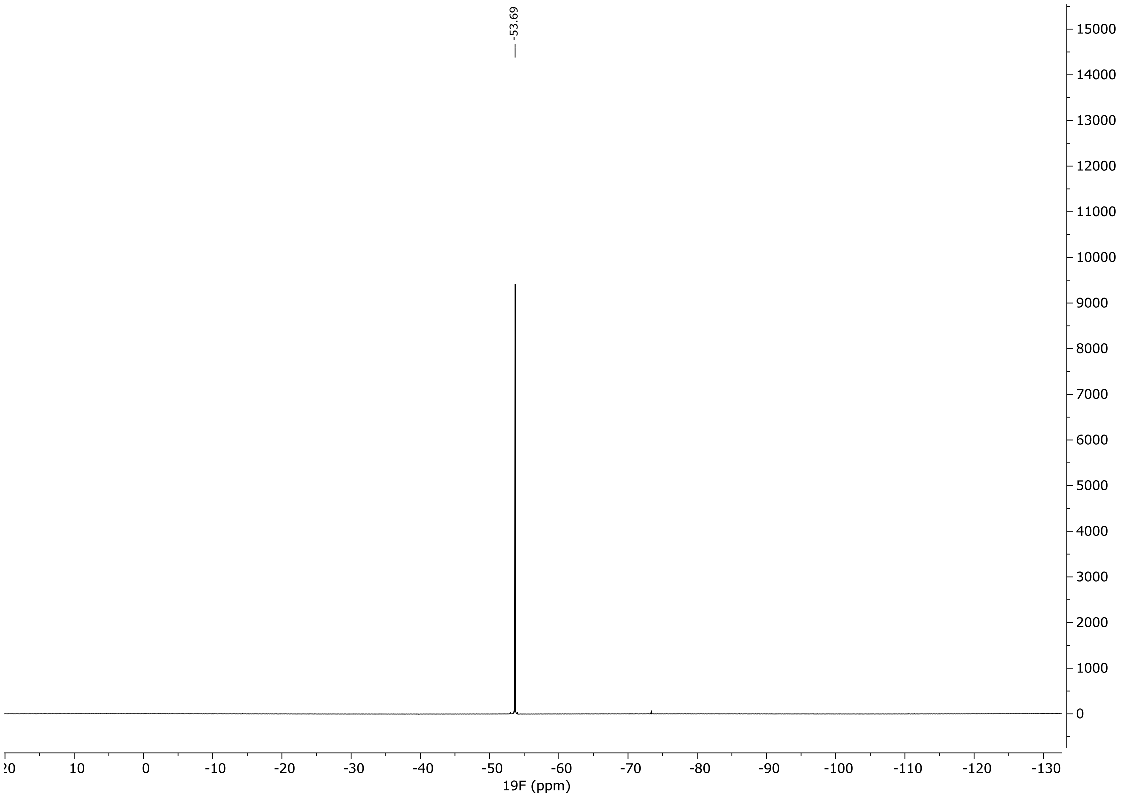

NMR spectra of compound **13D.12**

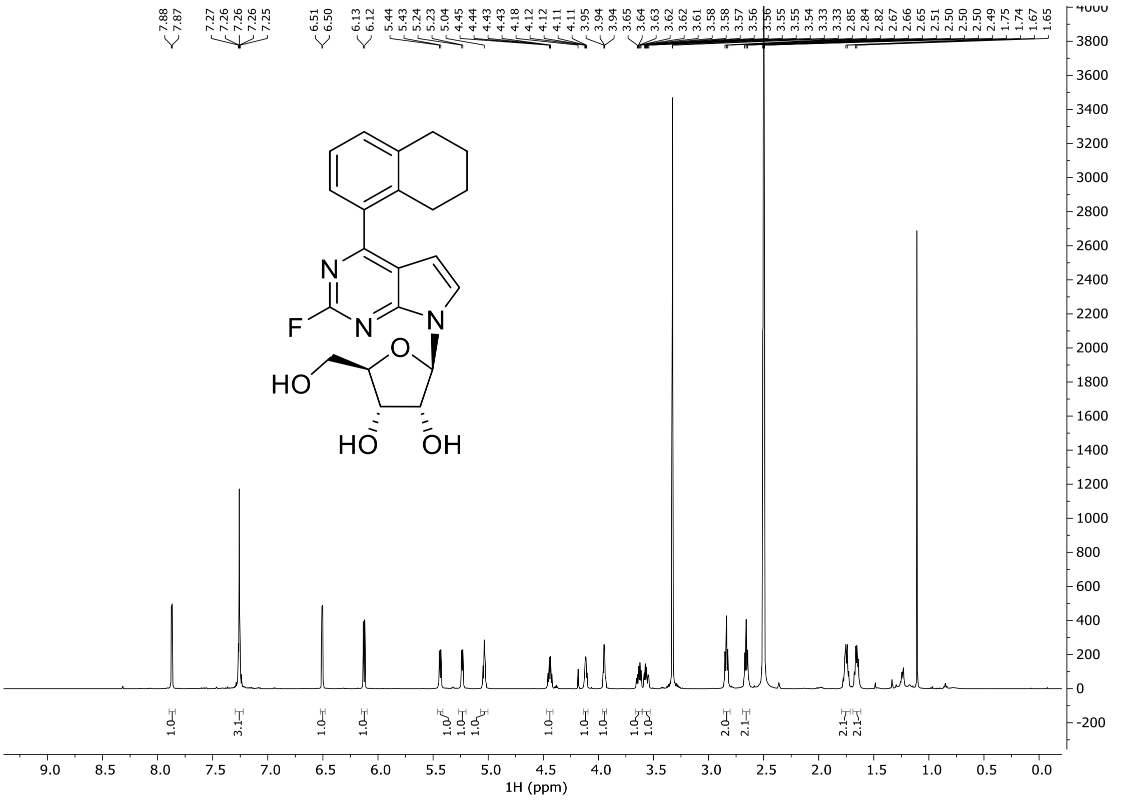

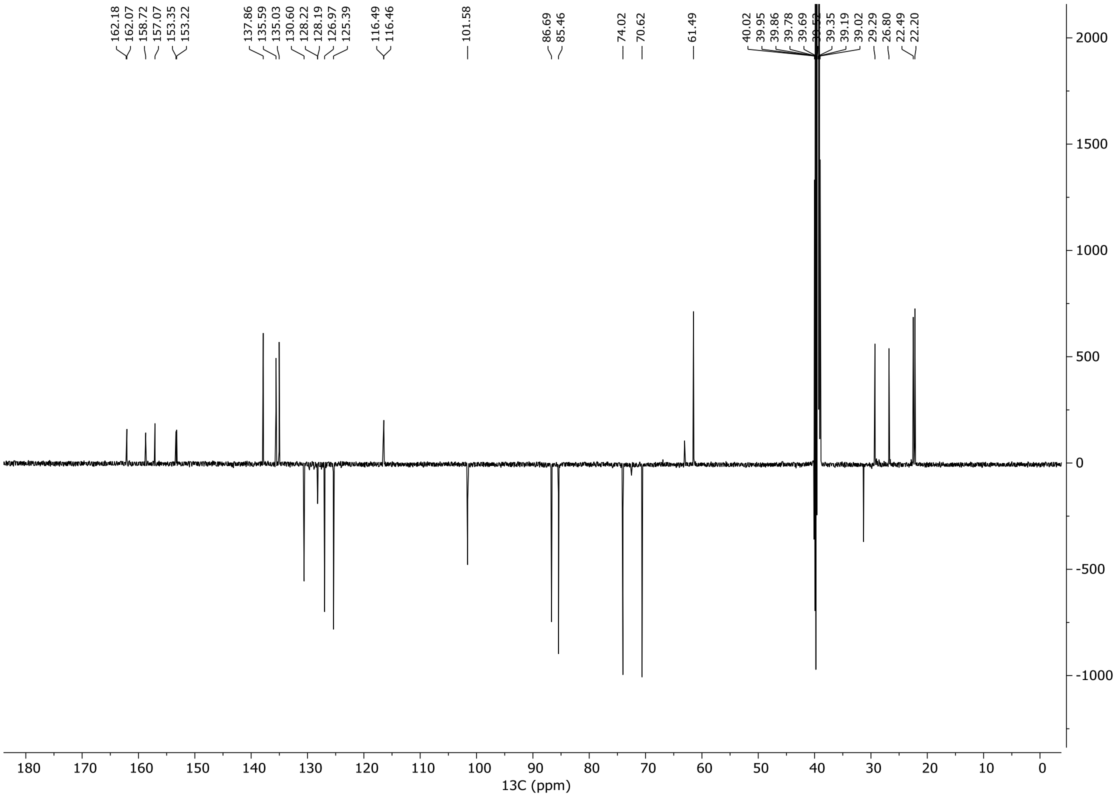
162.18
162.07
158.72
157.07
153.35
153.22
137.86
135.59
135.03
130.60
128.22
128.19
126.97
125.39
116.49
116.46
101.58
86.69
85.46
74.02
70.62
61.49
40.02
39.95
39.86
39.78
39.69
39.52
39.35
39.19
39.02
29.29
26.80
22.49
22.20
13C (ppm)

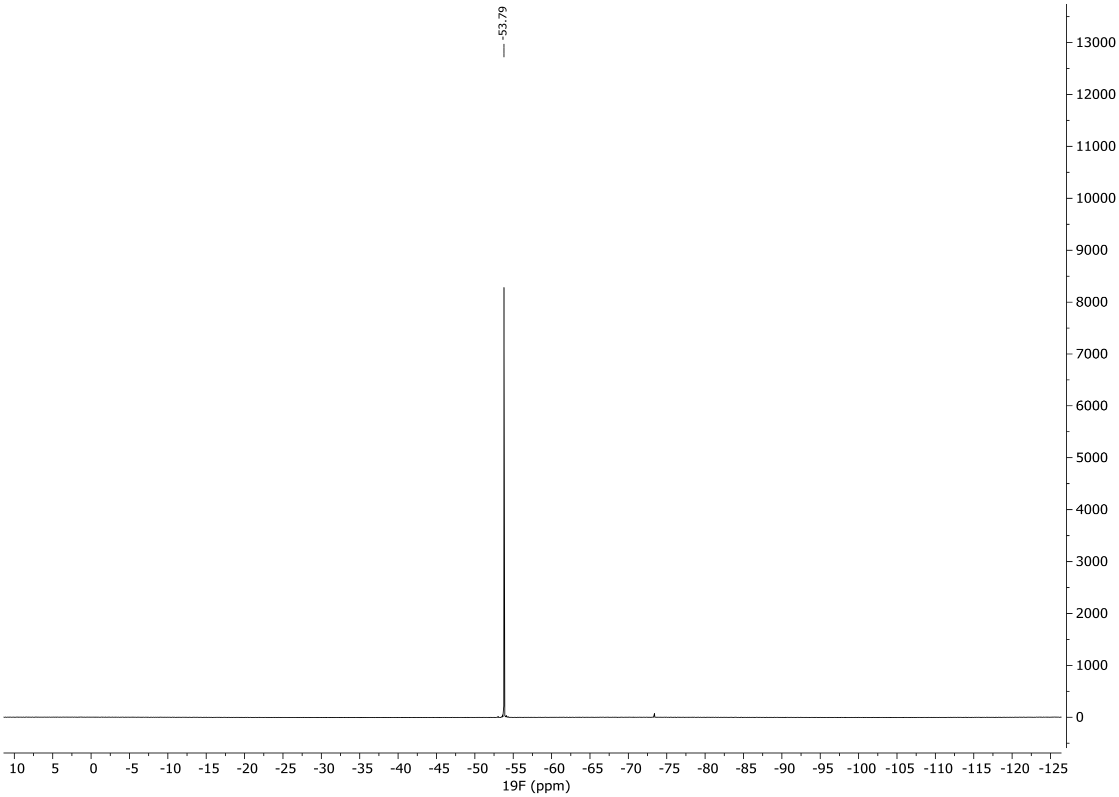
-53.79
19F (ppm)

NMR spectra of compound **14D.1**

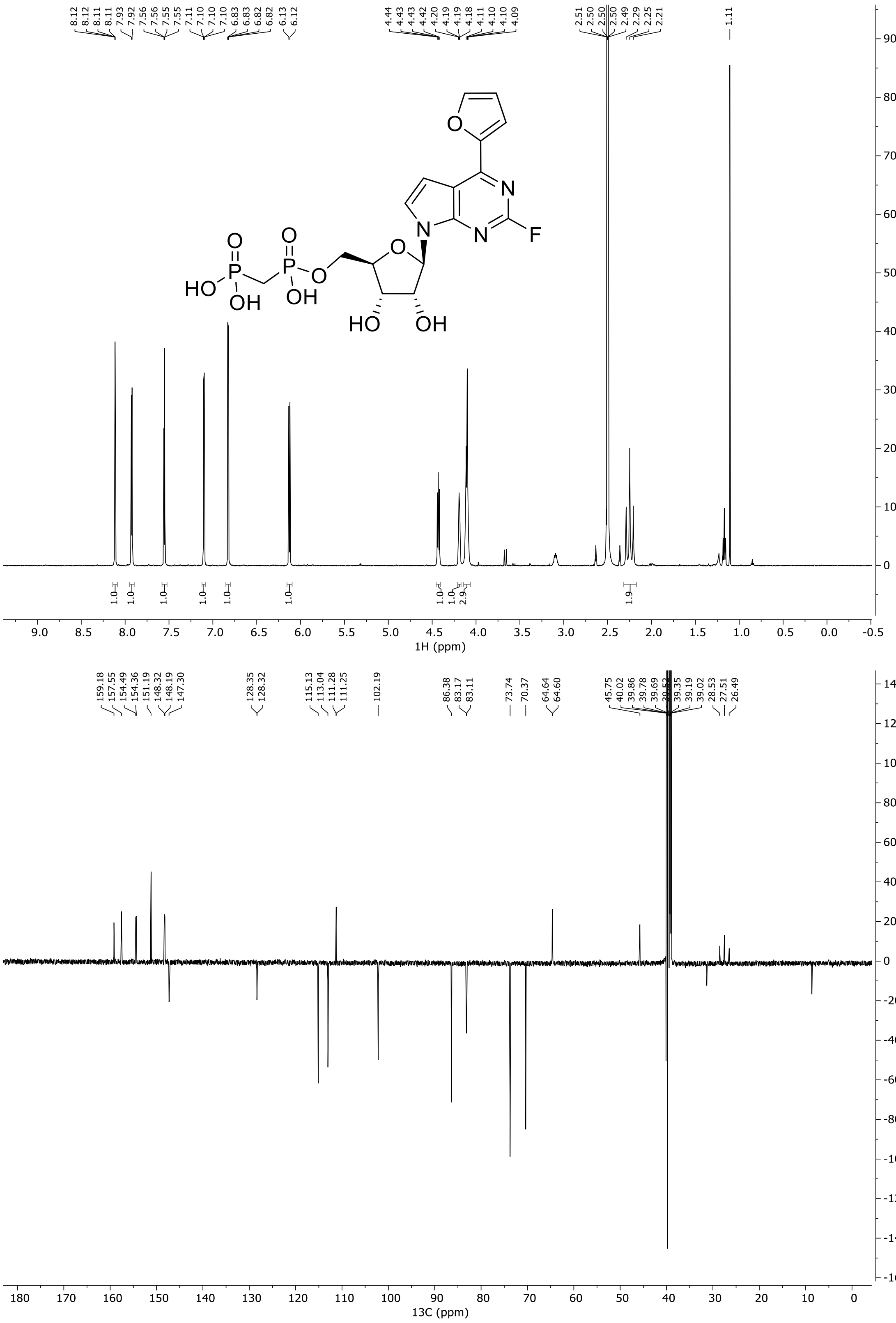

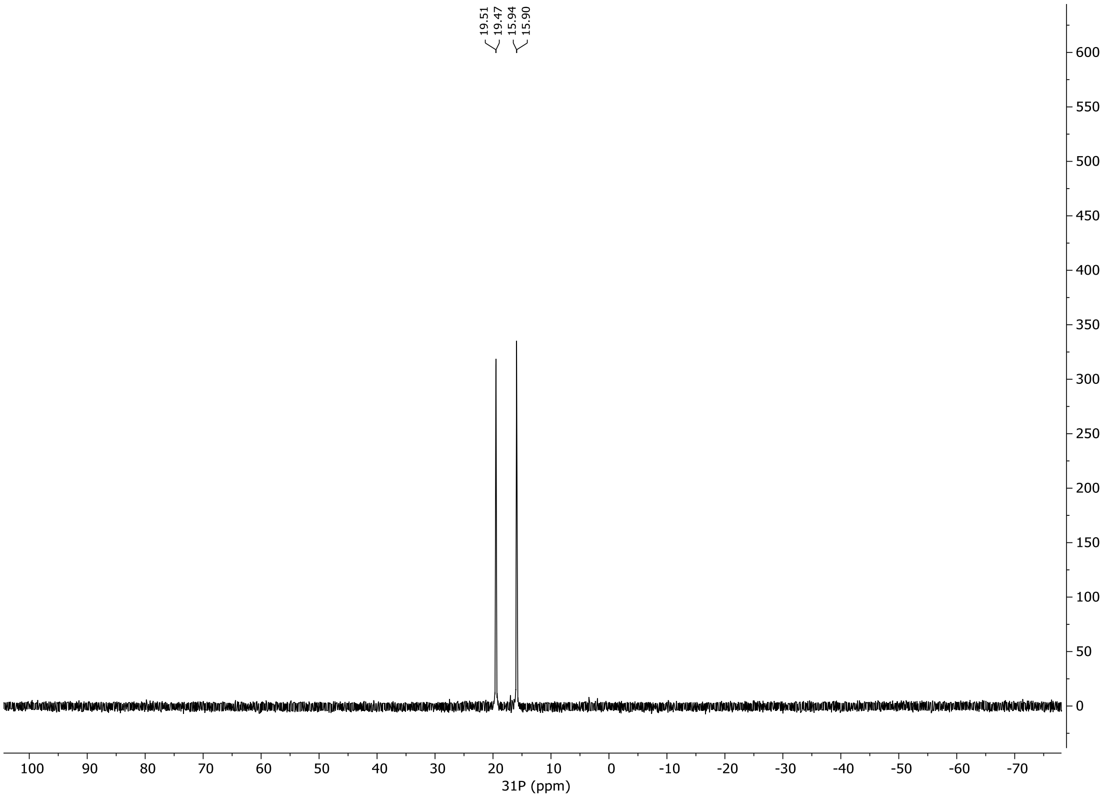
19.51
19.47
15.94
15.90
31P (ppm)

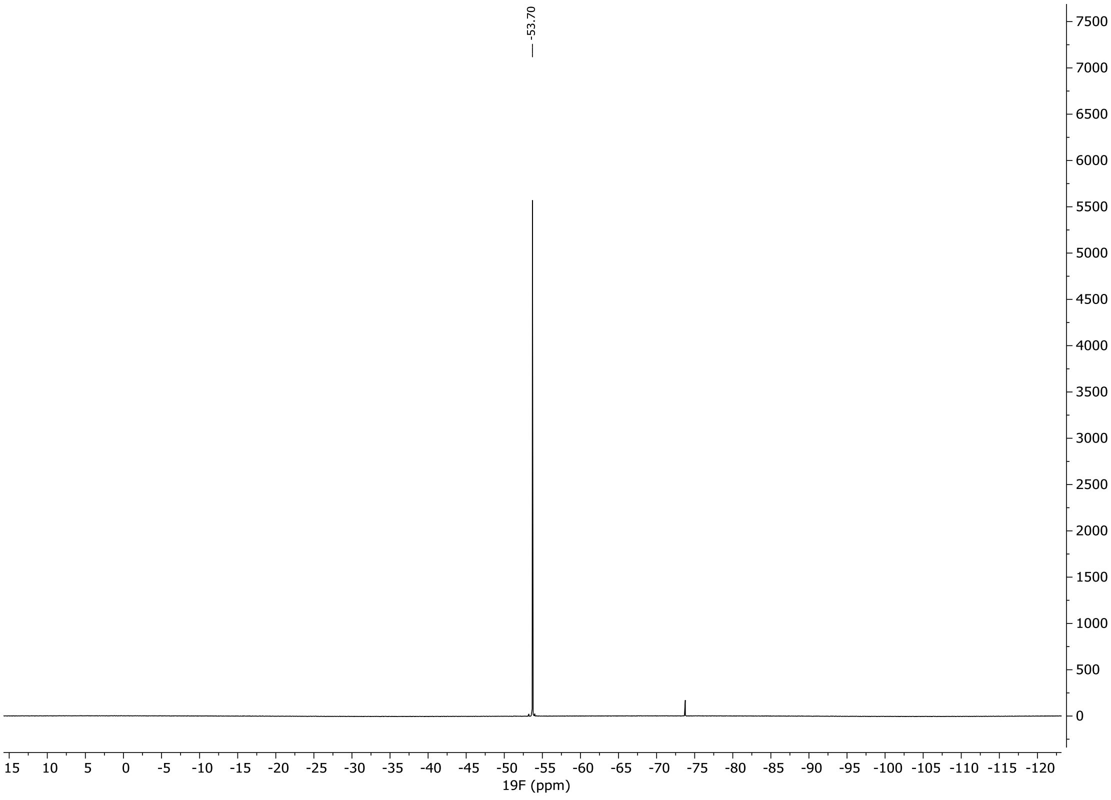
-53.70
19F (ppm)

NMR spectra of compound **14D.6**

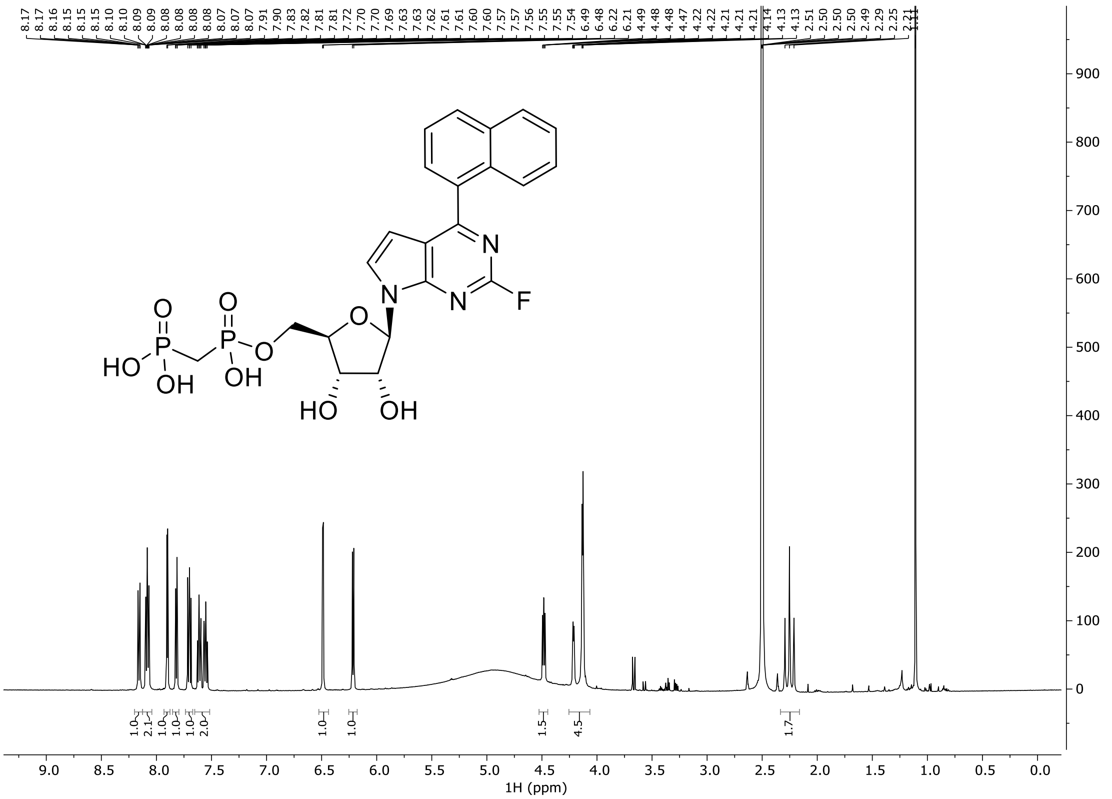

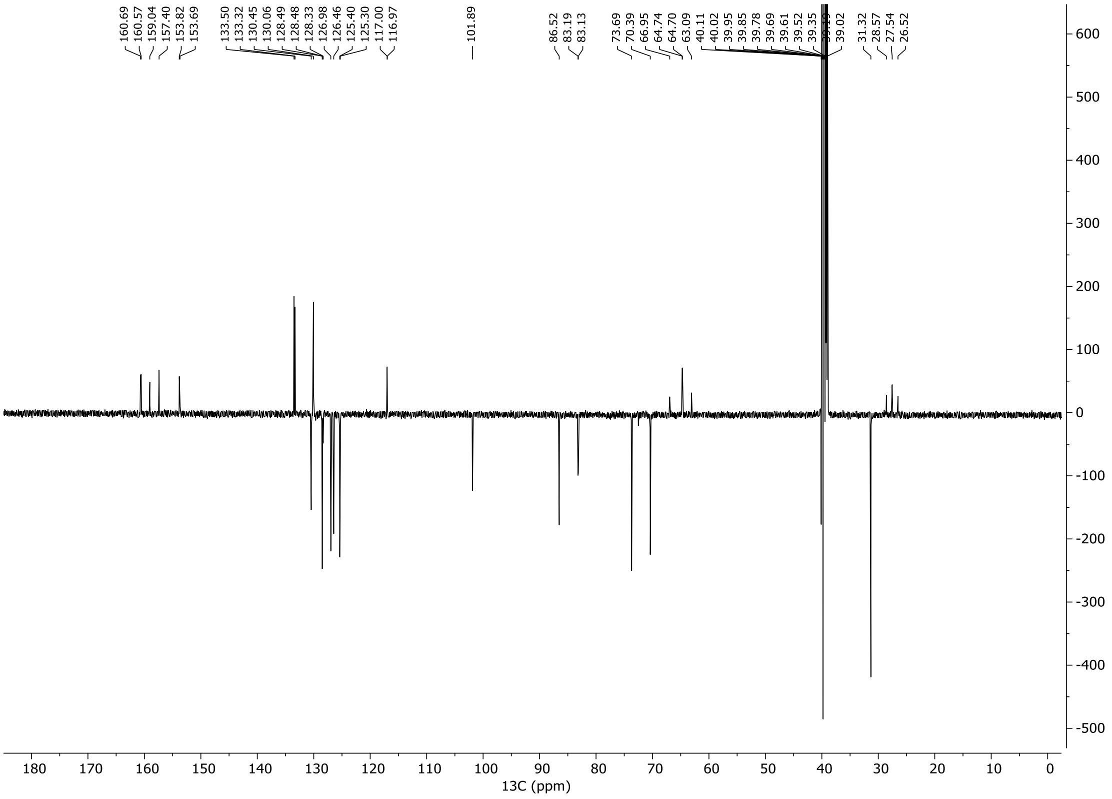

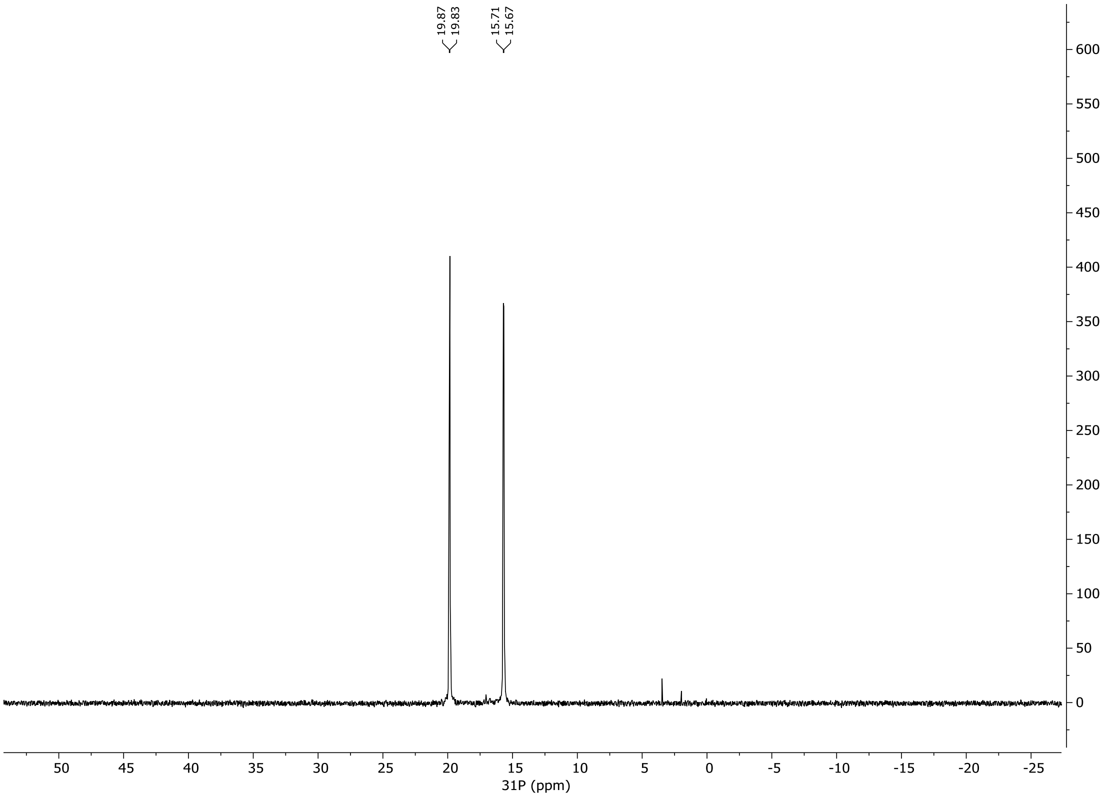
19.87
19.83
15.71
15.67
31P (ppm)

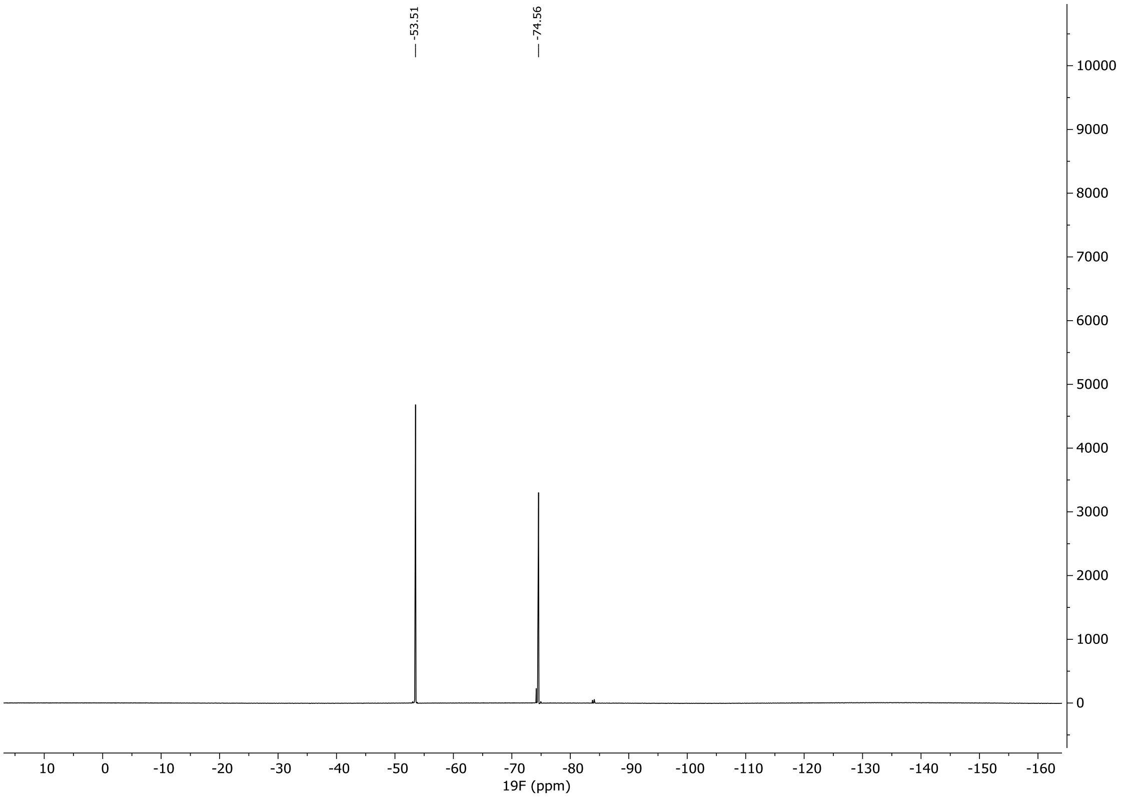
-53.51
-74.56
19F (ppm)

NMR spectra of compound **14D.7**

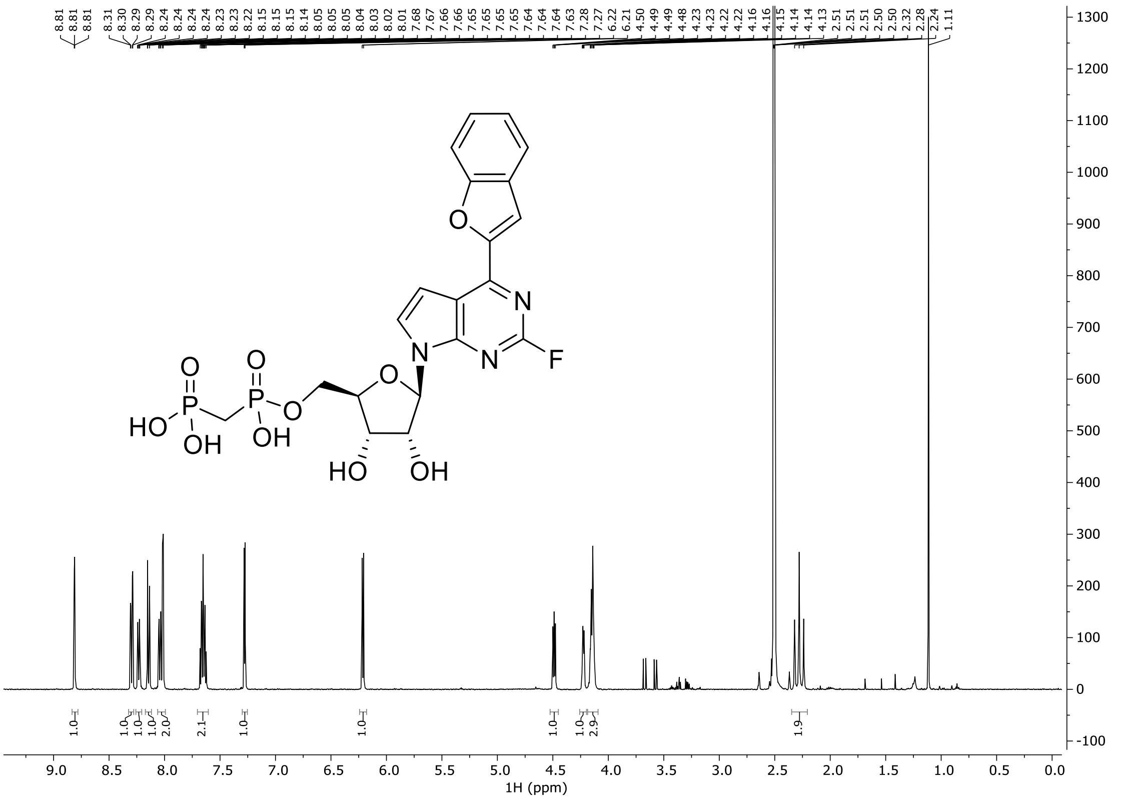

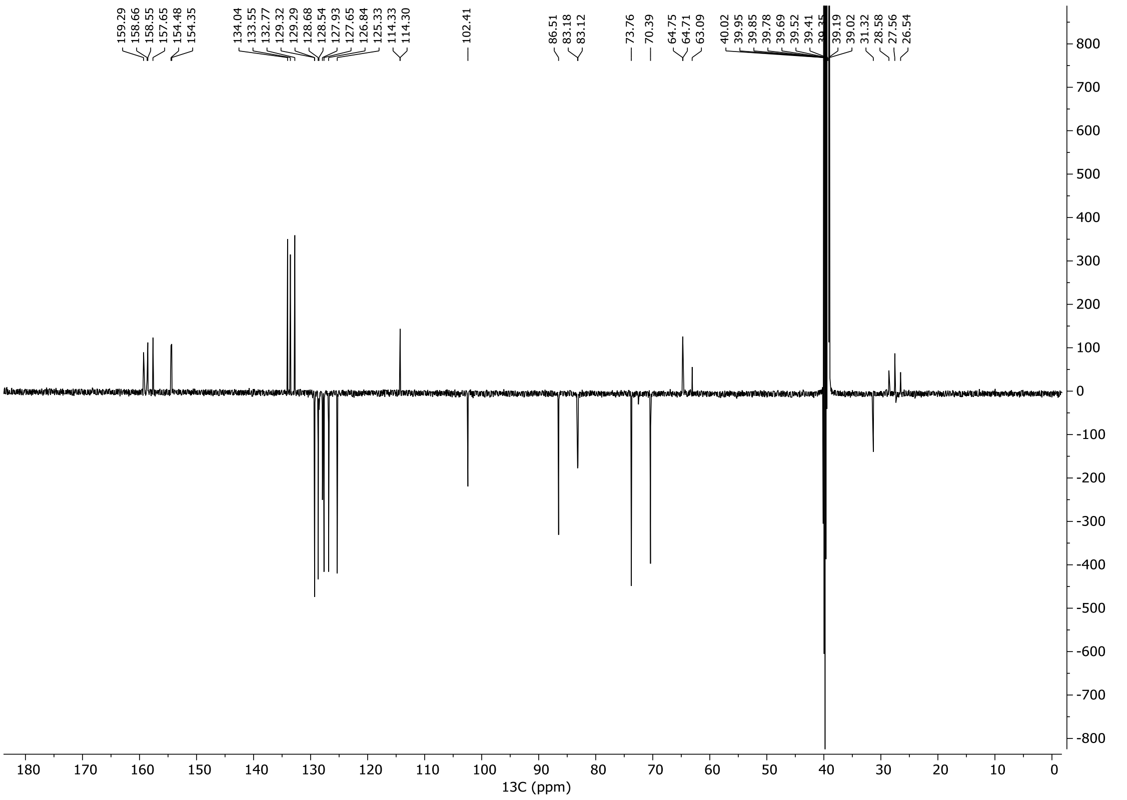

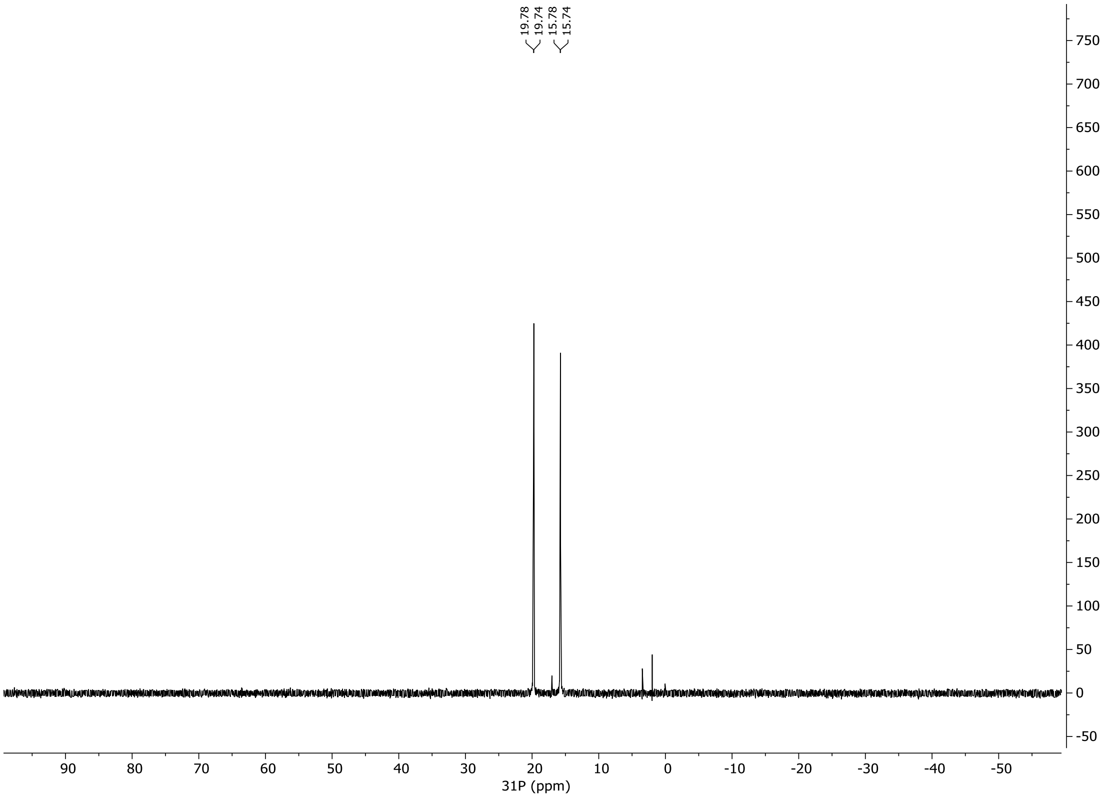
19.78
19.74
15.78
15.74
31P (ppm)

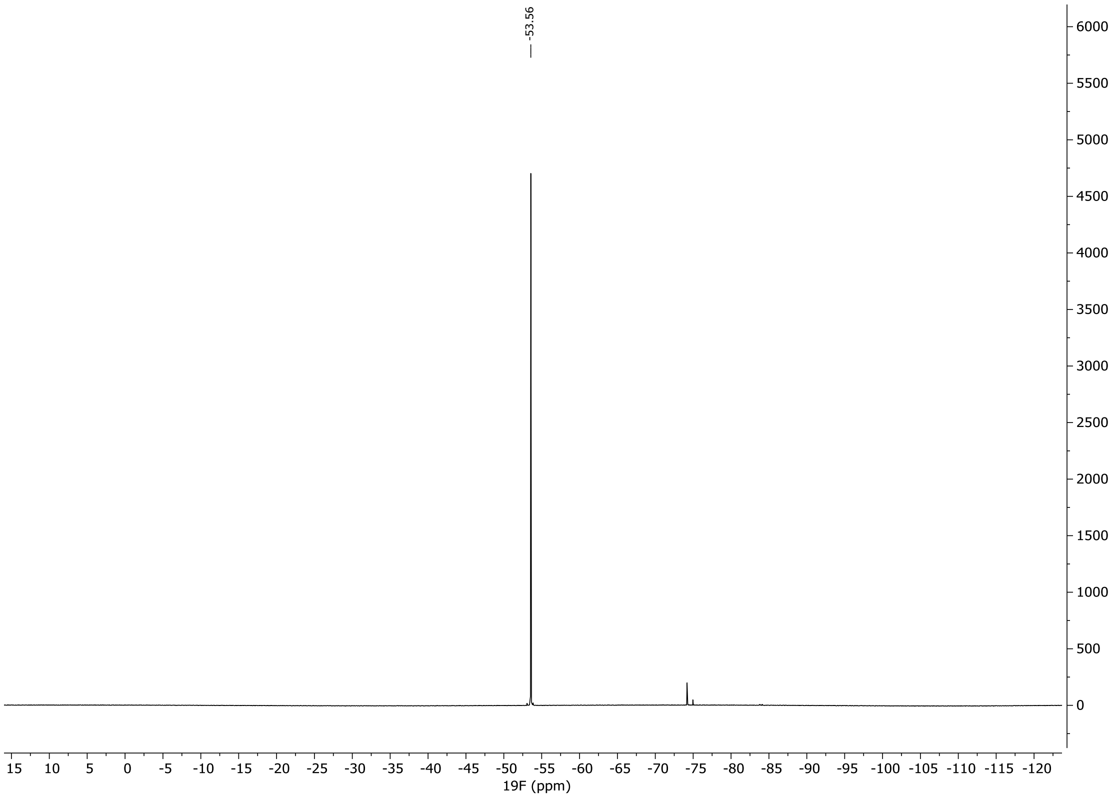
-53.56
19F (ppm)

NMR spectra of compound **14D.8**

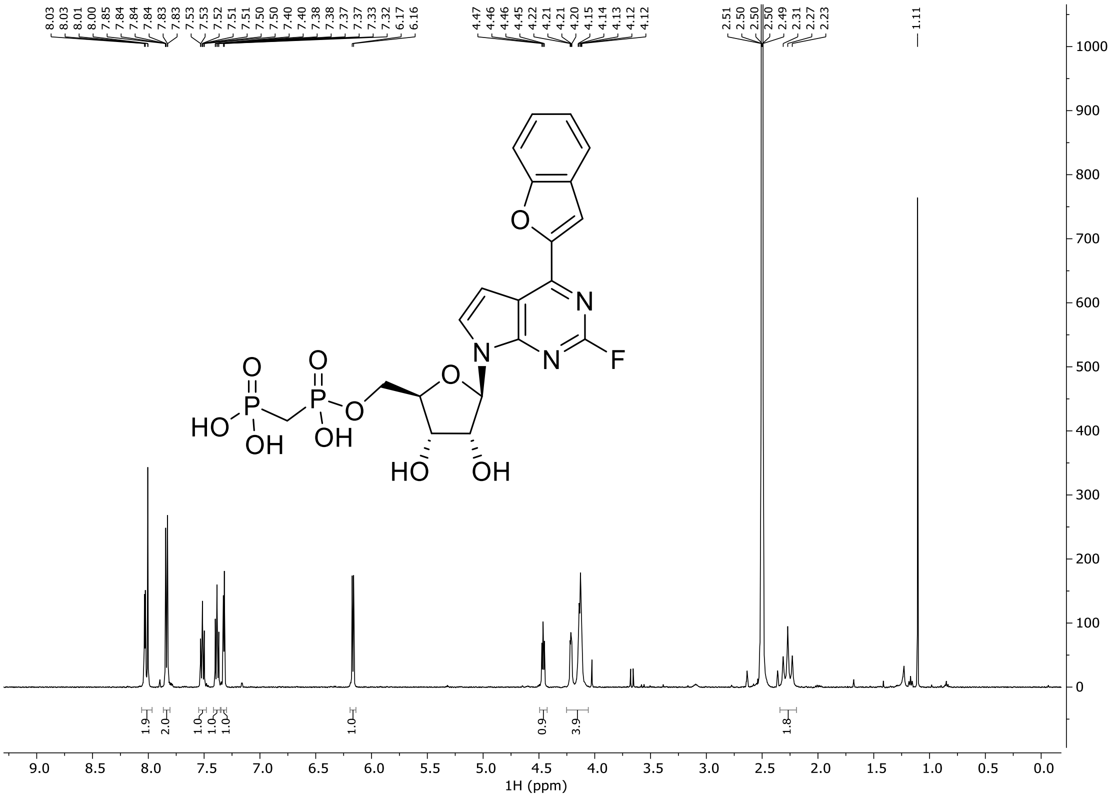

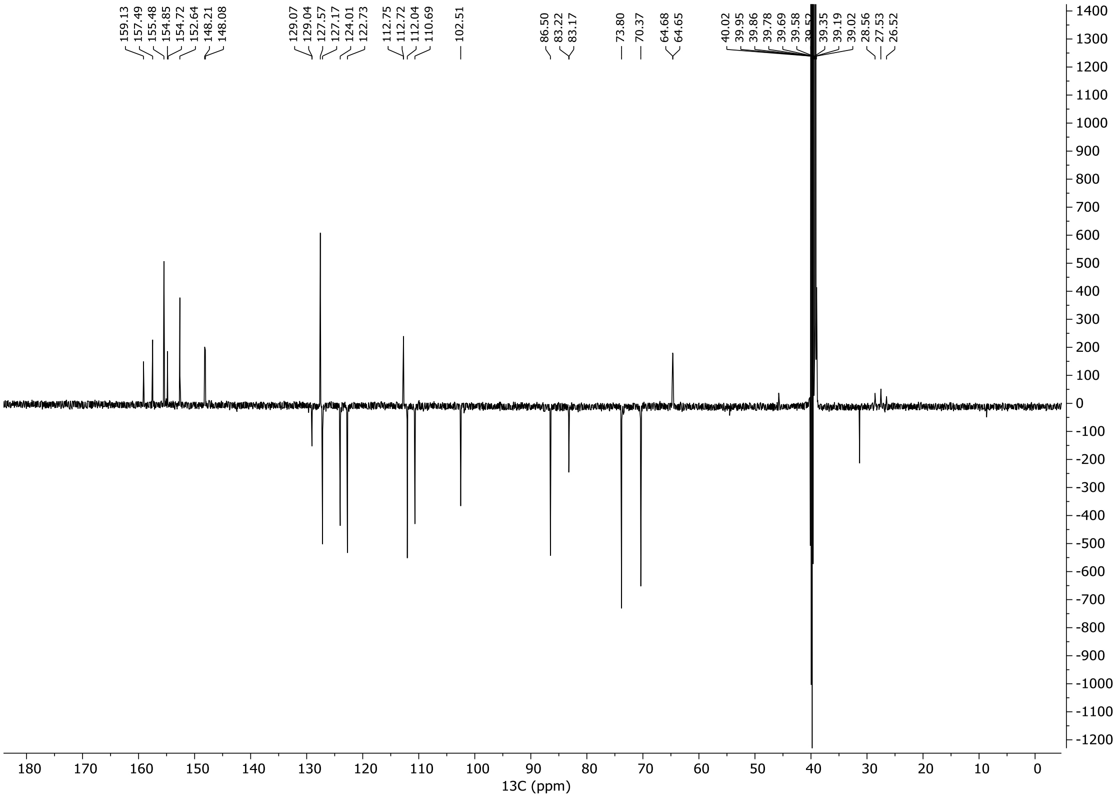

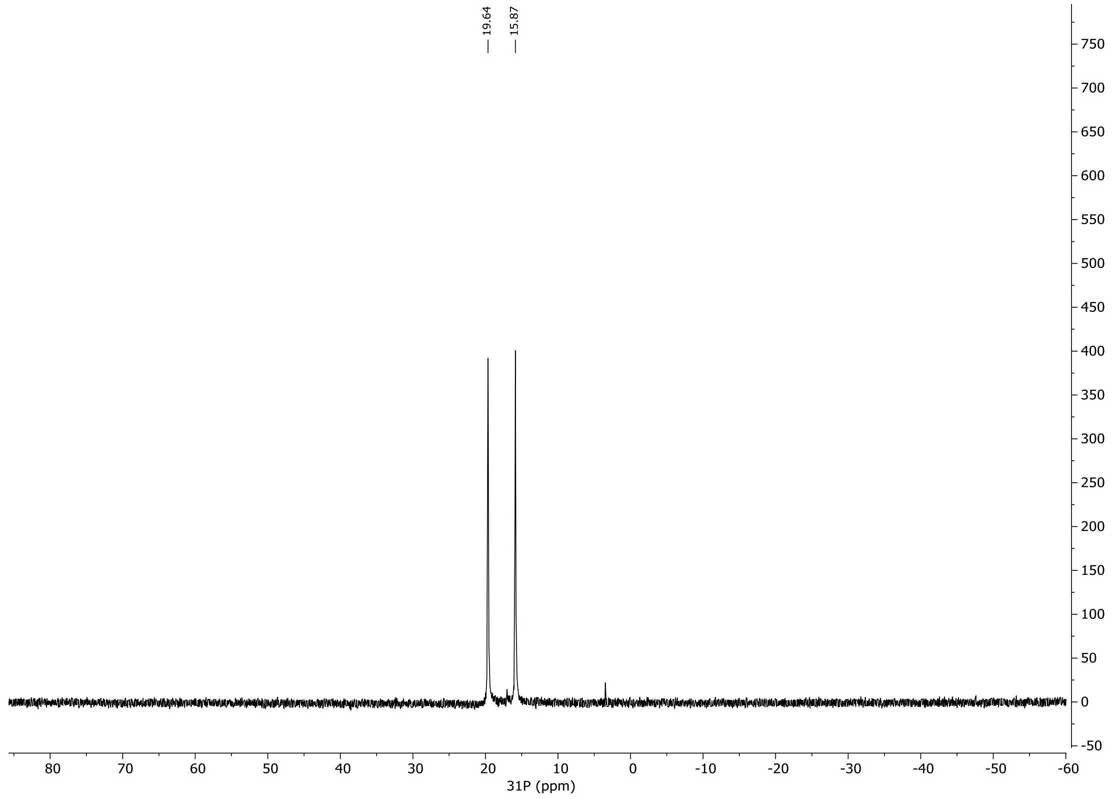
19.64
15.87
31P (ppm)

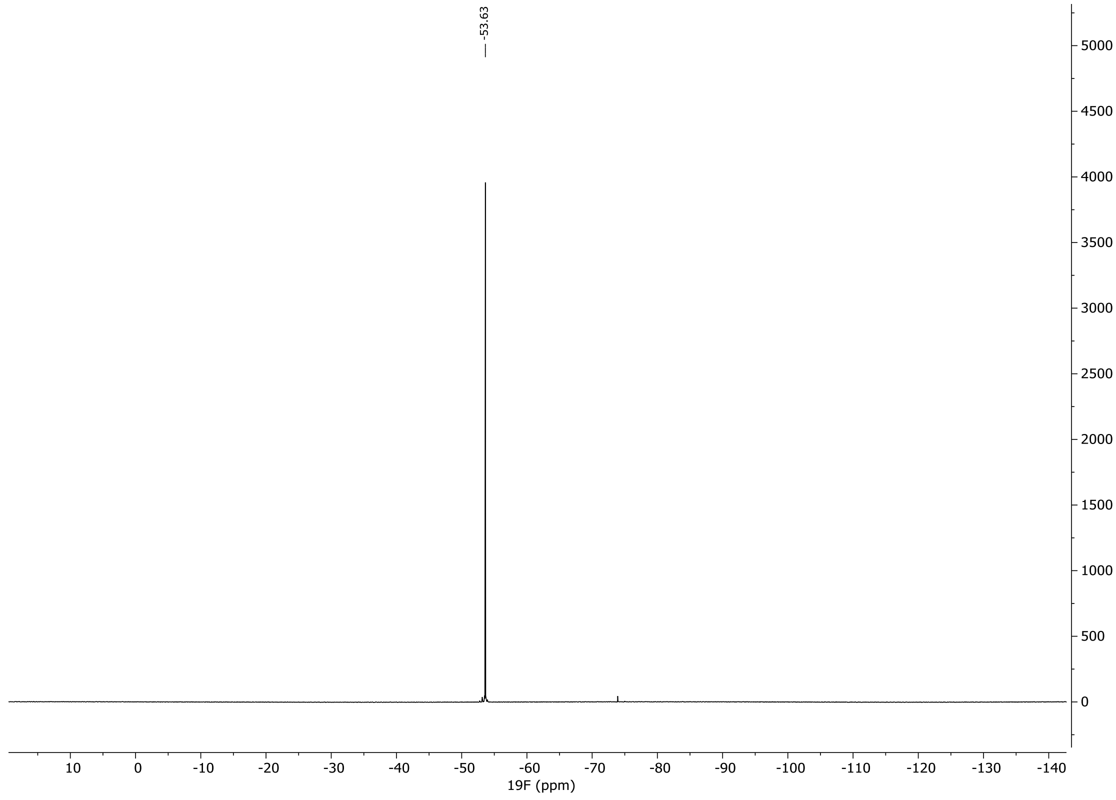
-53.63
19F (ppm)

NMR spectra of compound **14D.12**

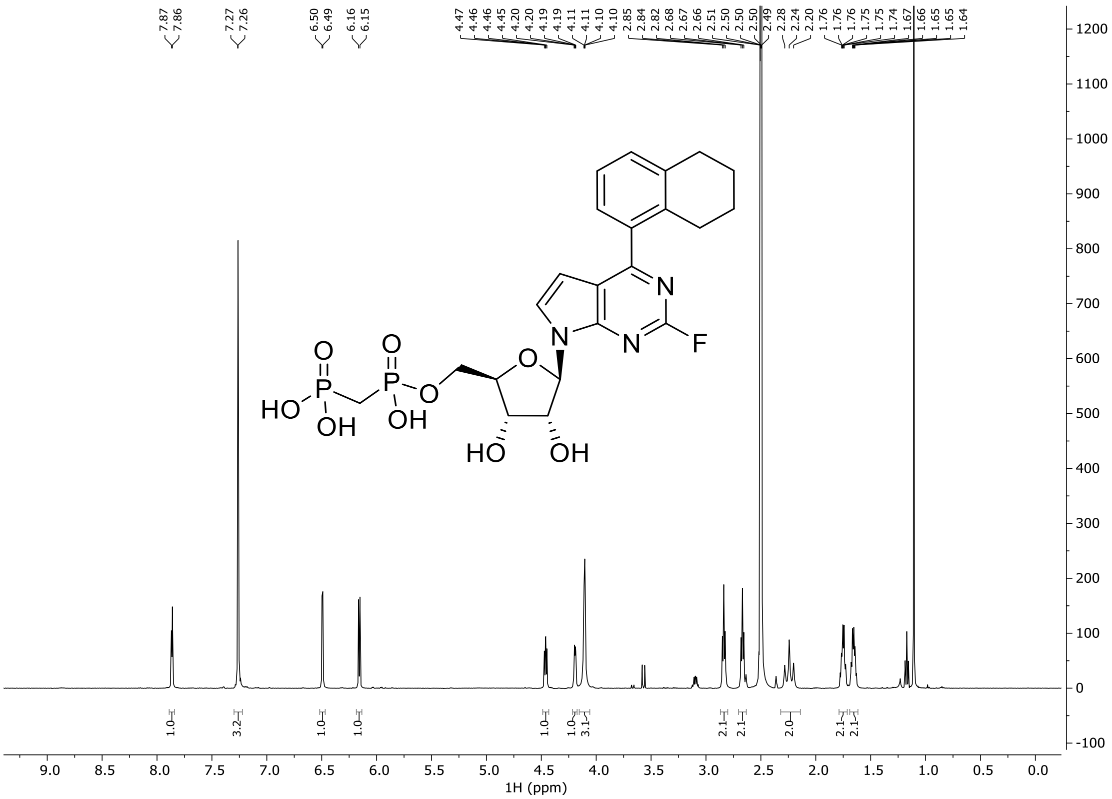

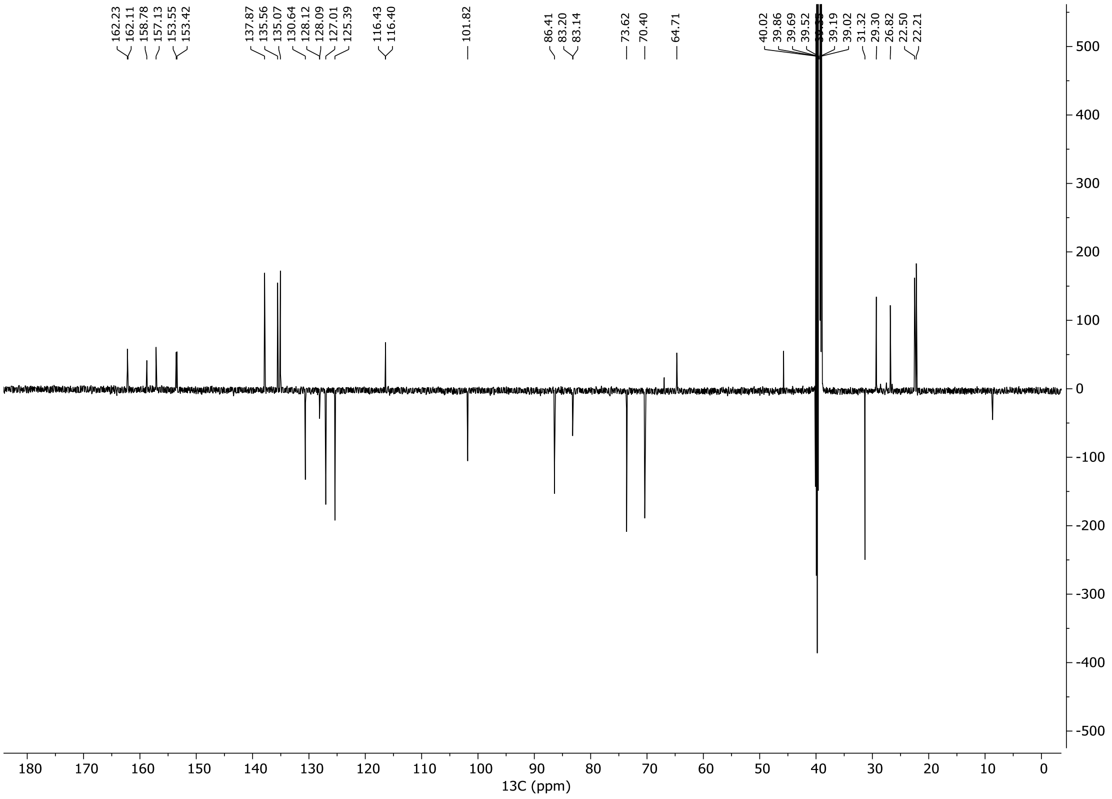

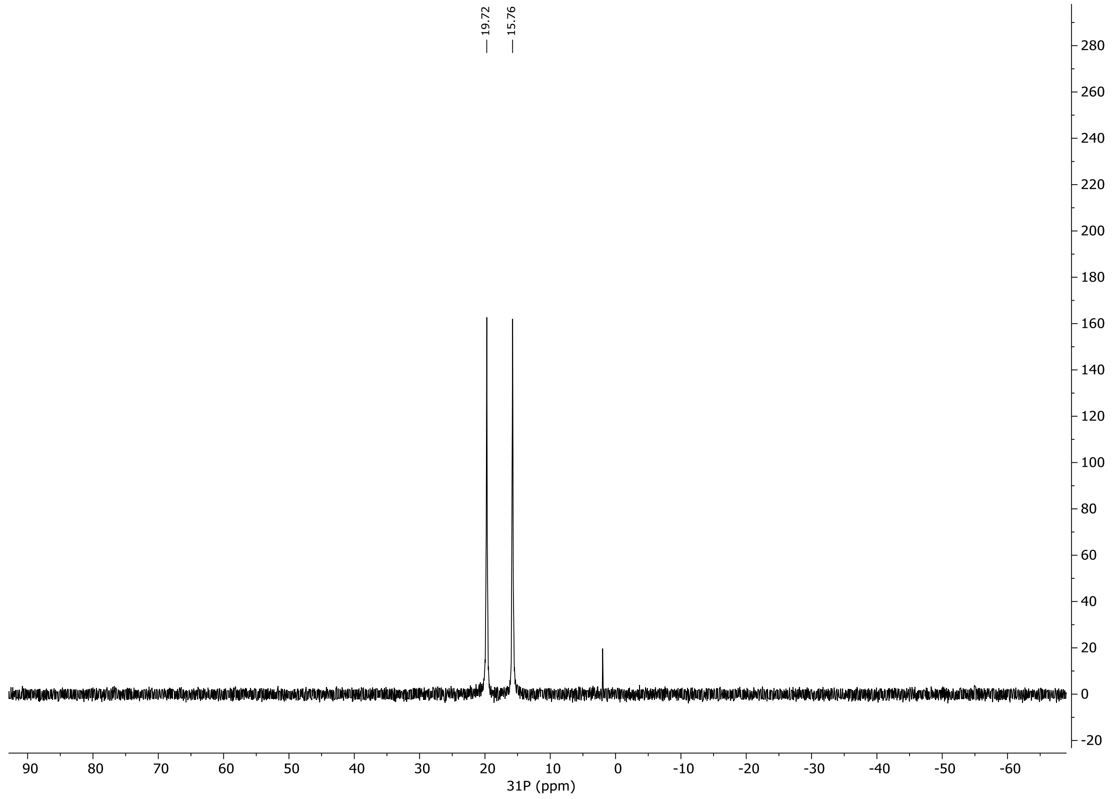
19.72
15.76
31P (ppm)

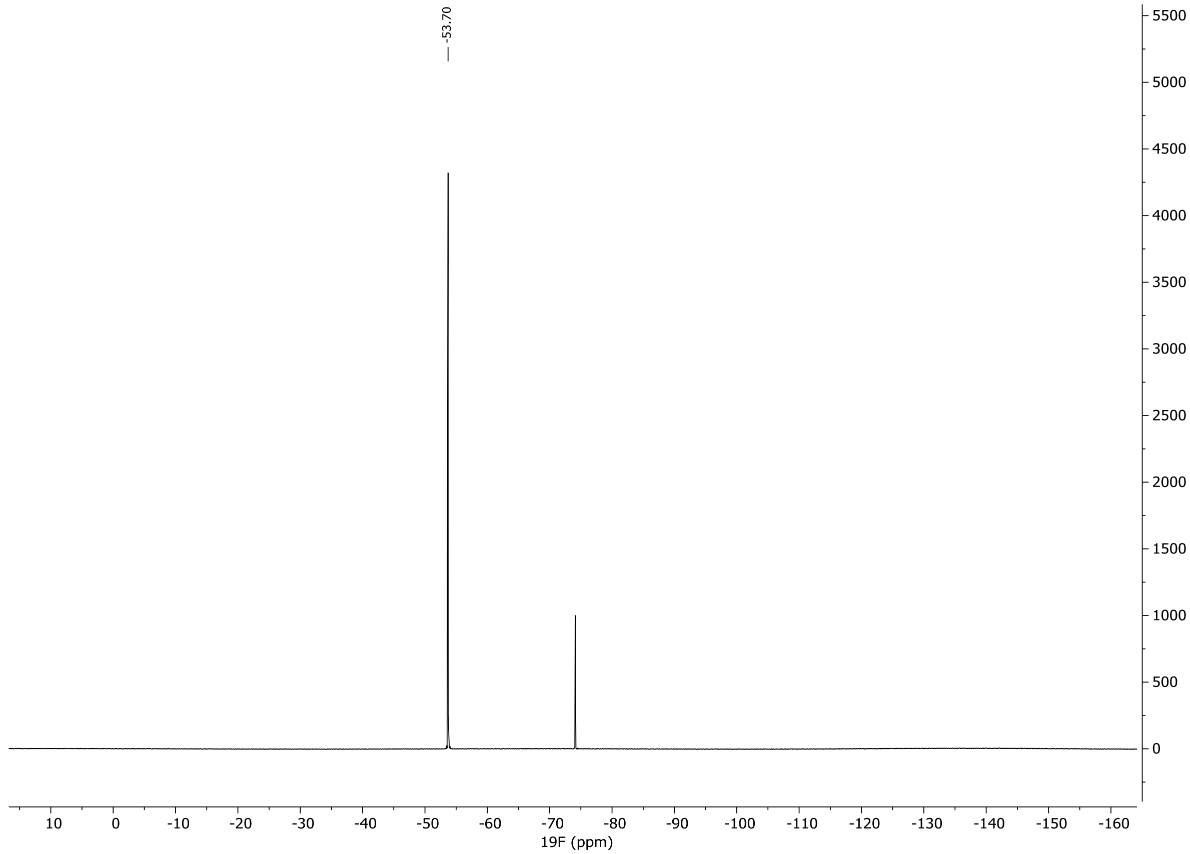
-53.70
19F (ppm)

NMR spectra of compound **16**

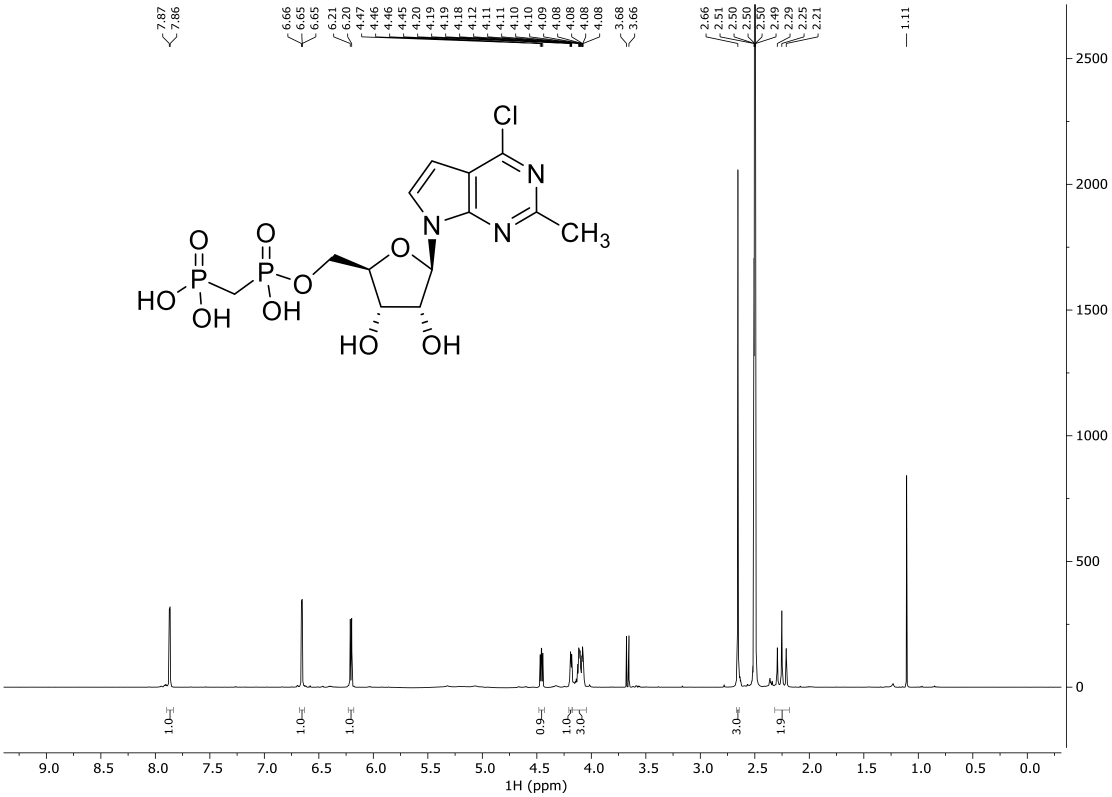

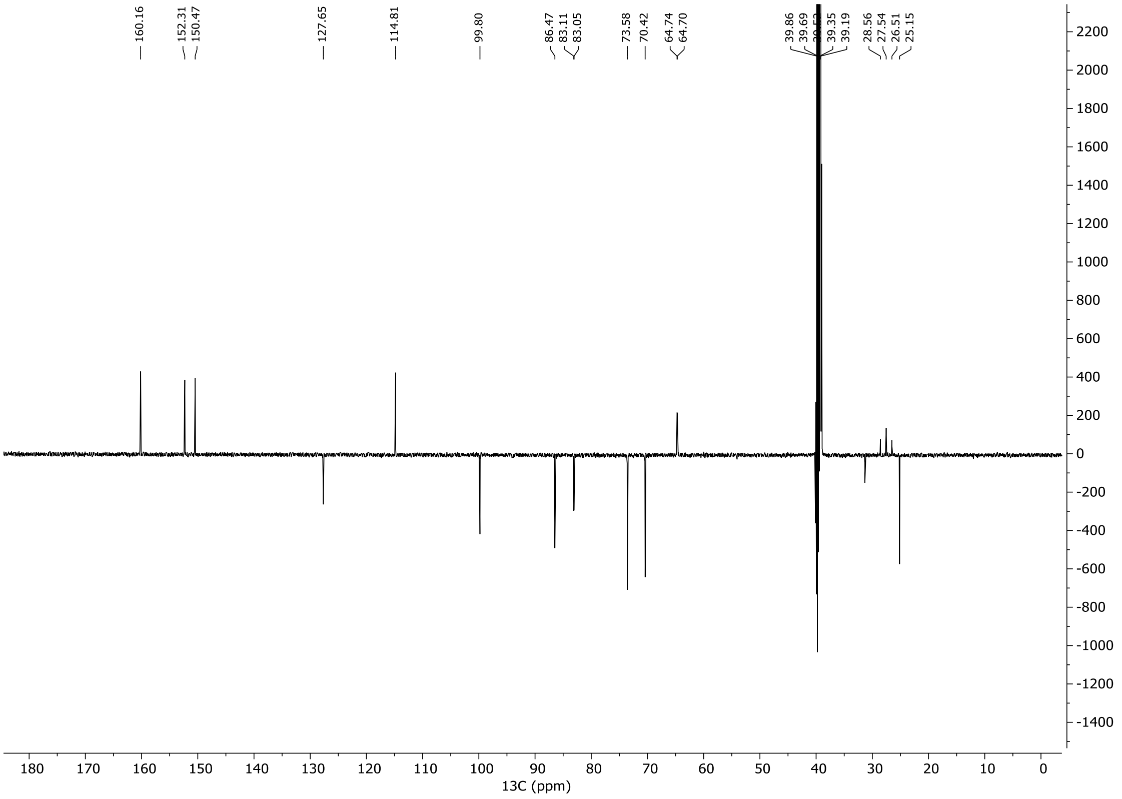

NMR spectra of compound **17E.6**

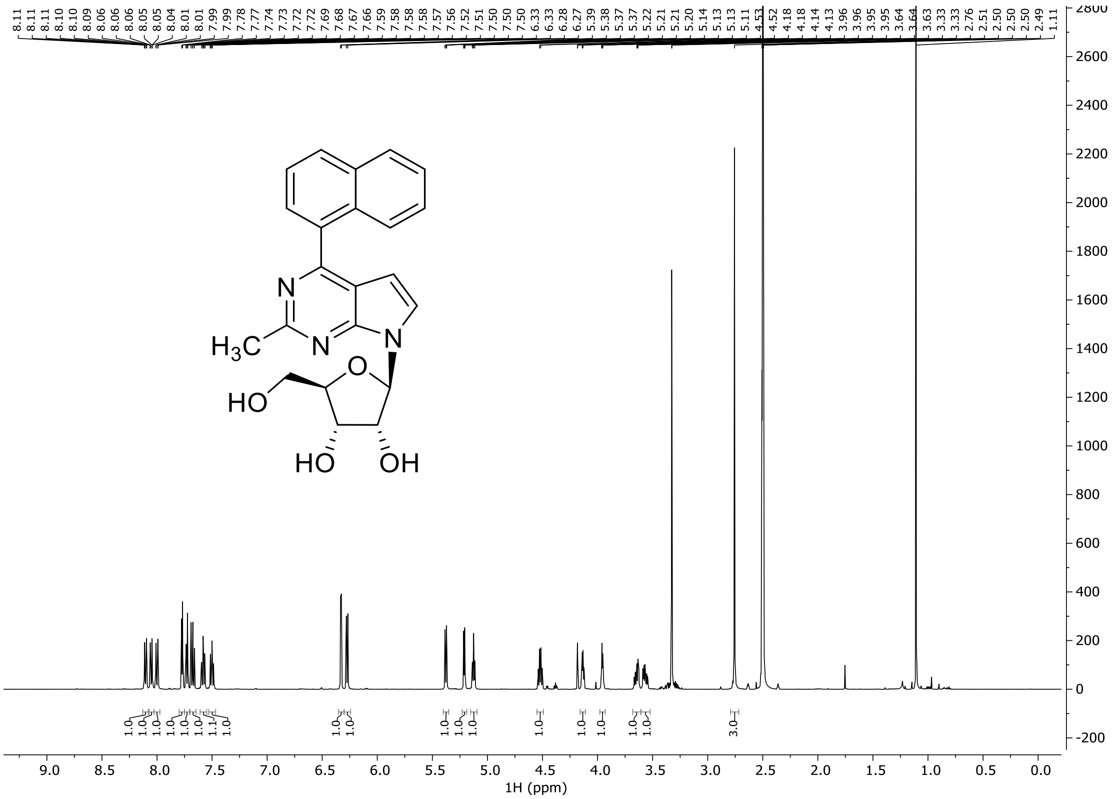

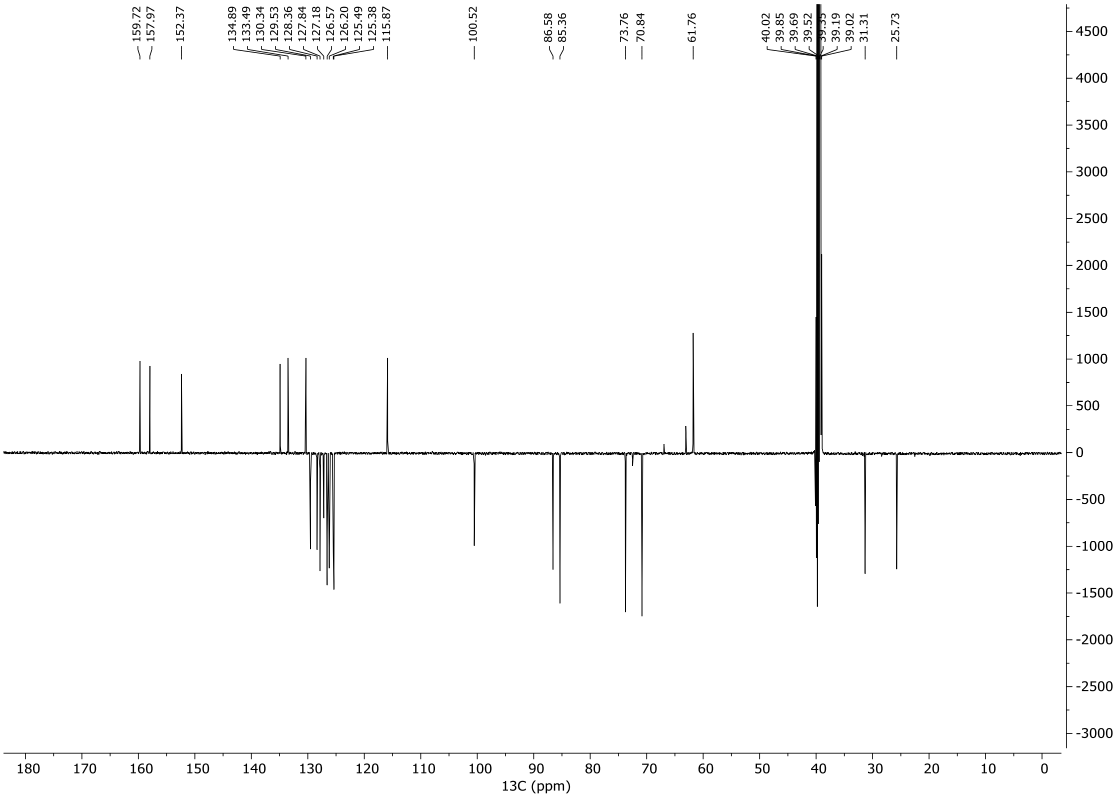

NMR spectra of compound **18E.1**

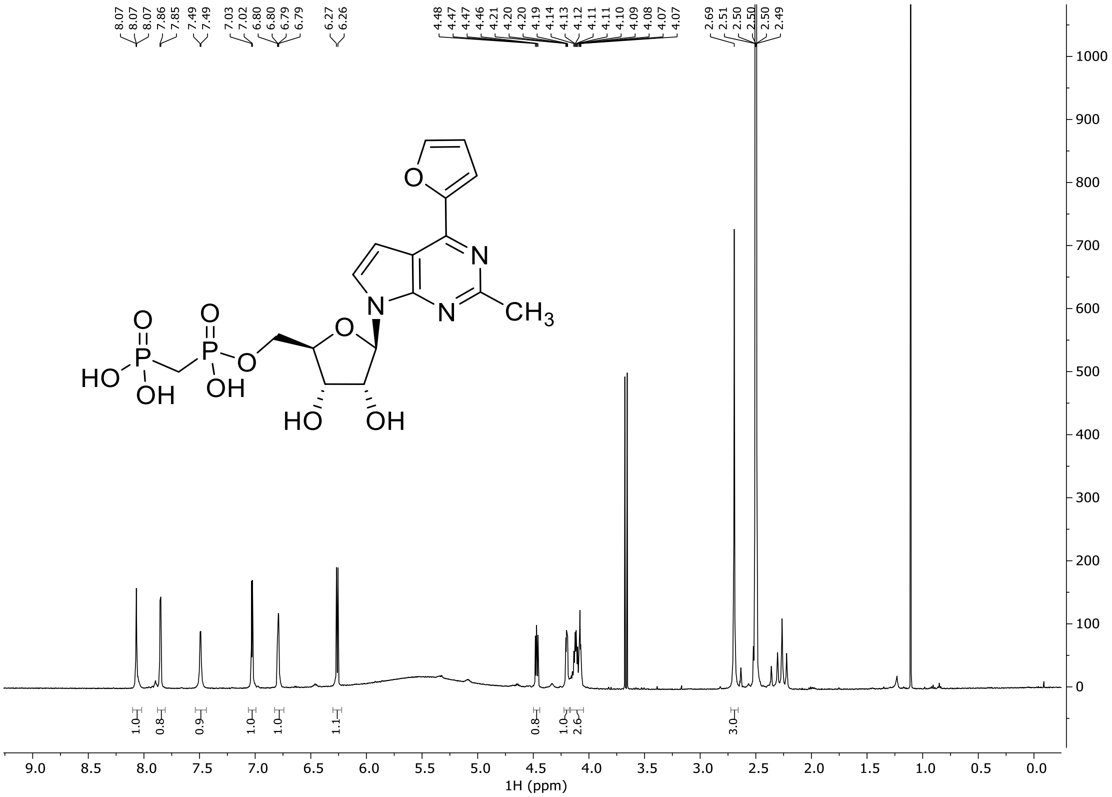

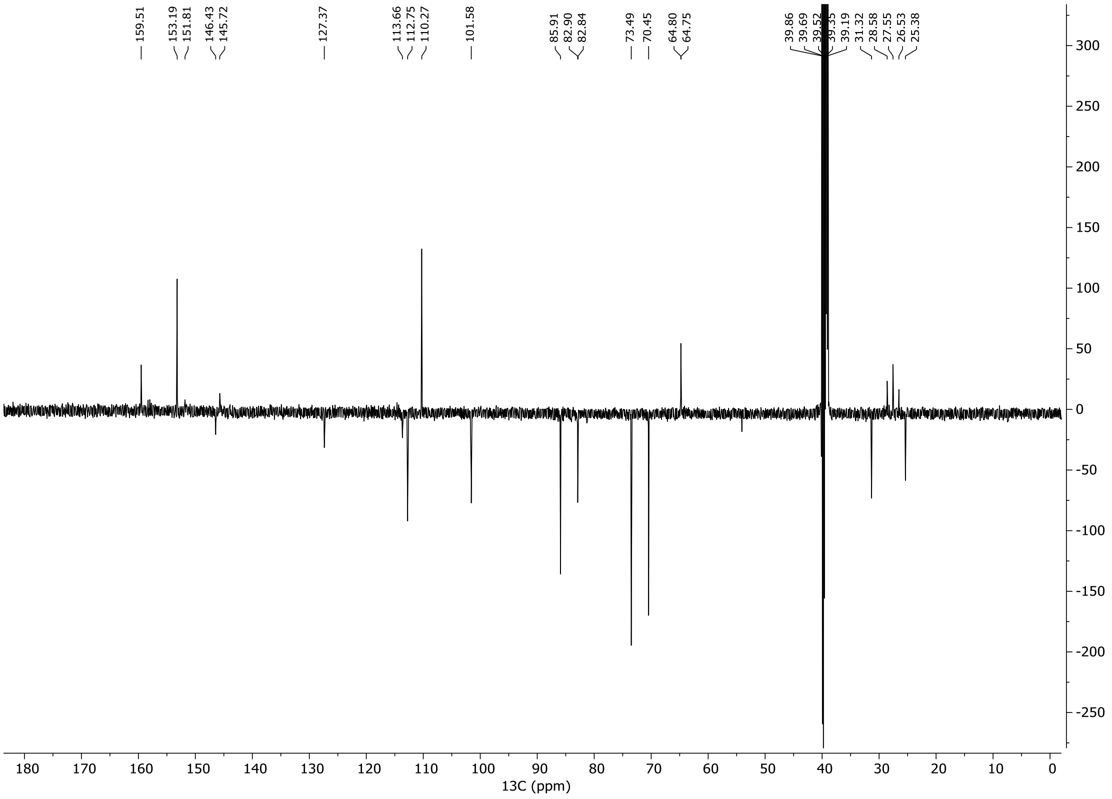

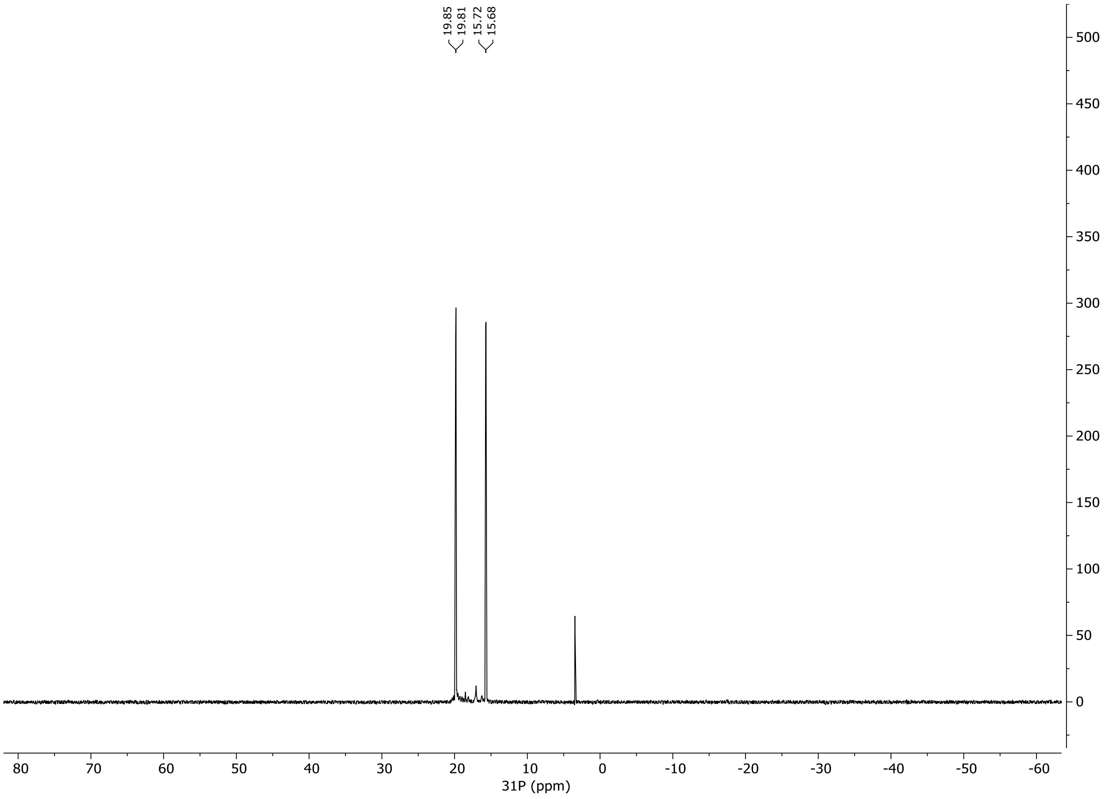

NMR spectra of compound **18E.6**

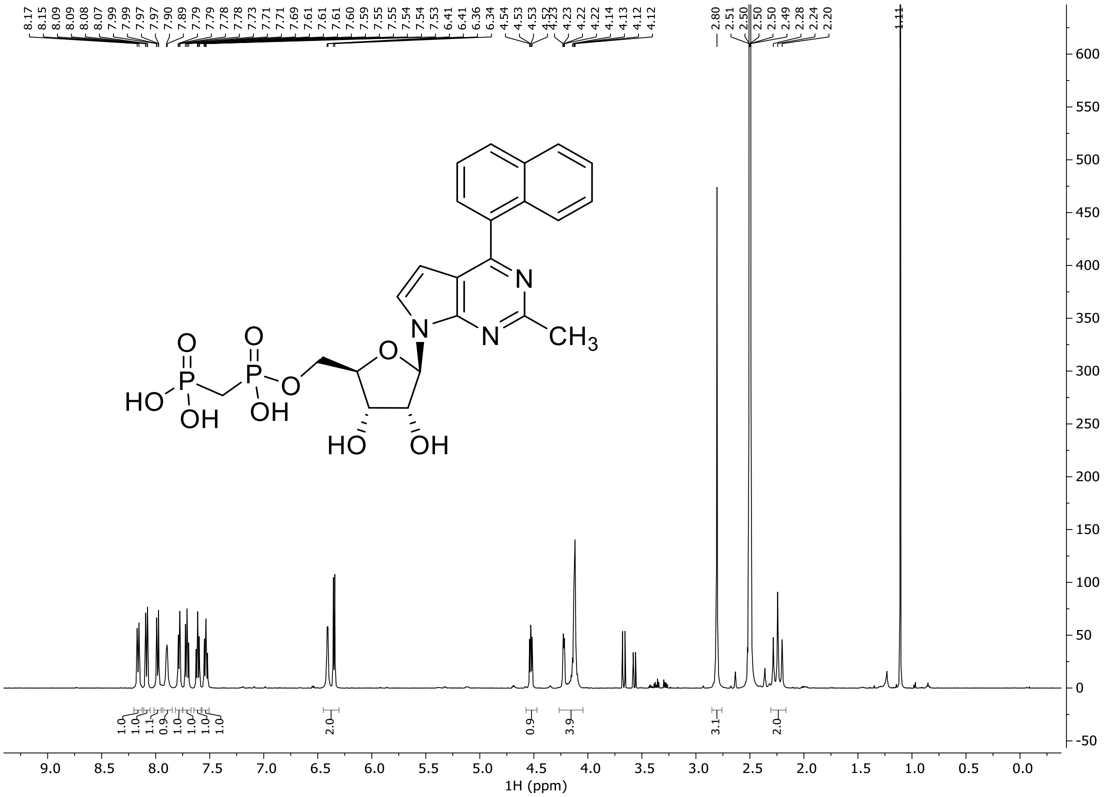

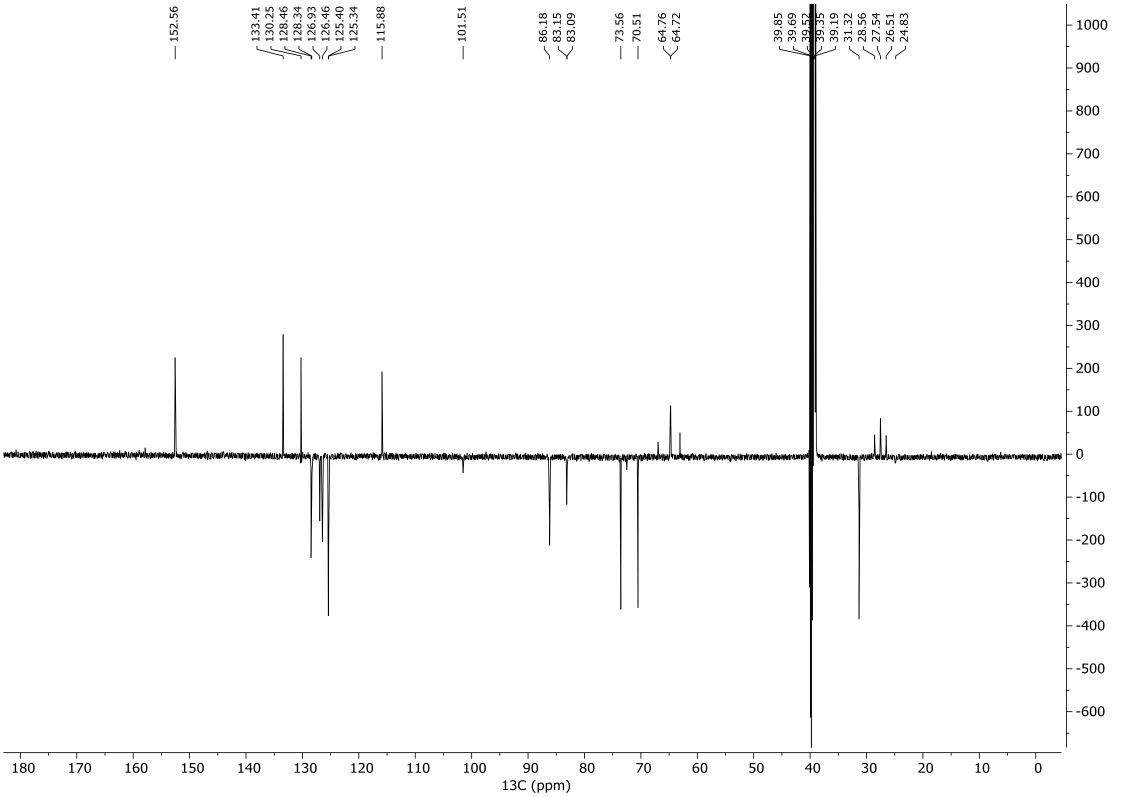
152.56
133.41
130.25
128.46
128.34
126.93
126.46
125.40
125.34
115.88
101.51
86.18
83.15
83.09
73.56
70.51
64.76
64.72
39.85
39.69
39.52
39.35
39.19
31.32
28.56
27.54
26.51
24.83
13C (ppm)

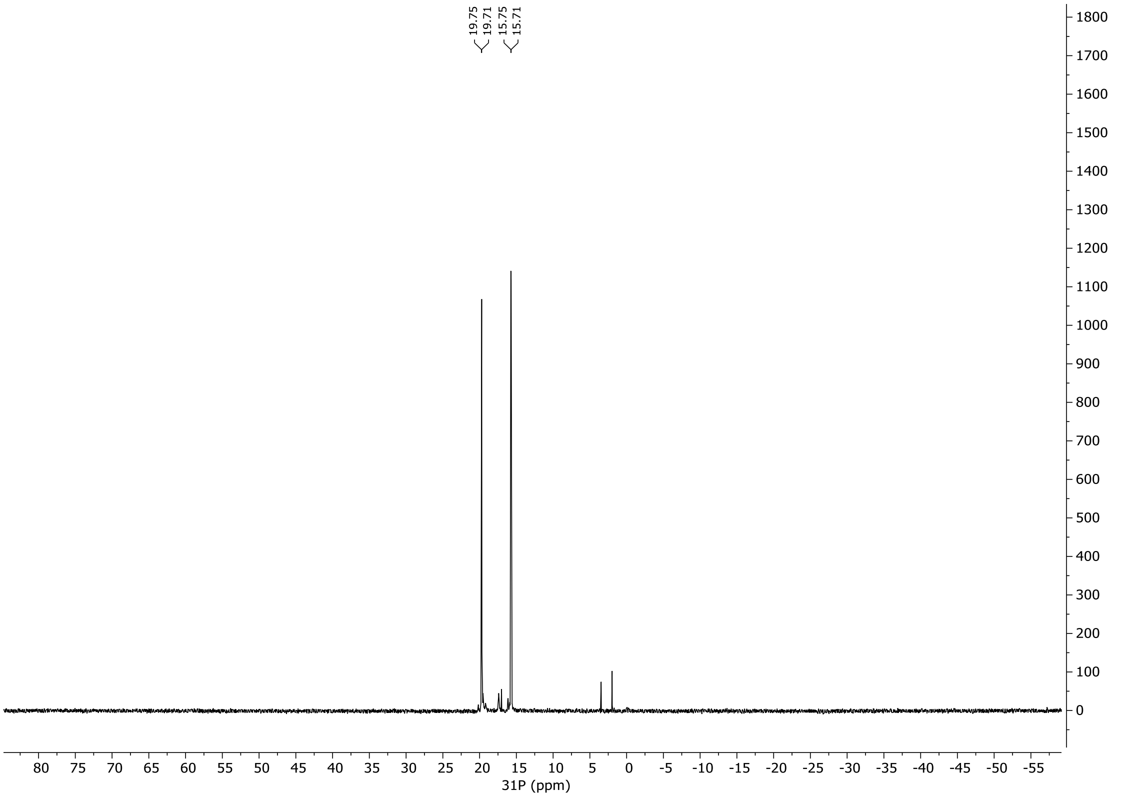
19.75
19.71
15.75
15.71
31P (ppm)

NMR spectra of compound **18E.7**

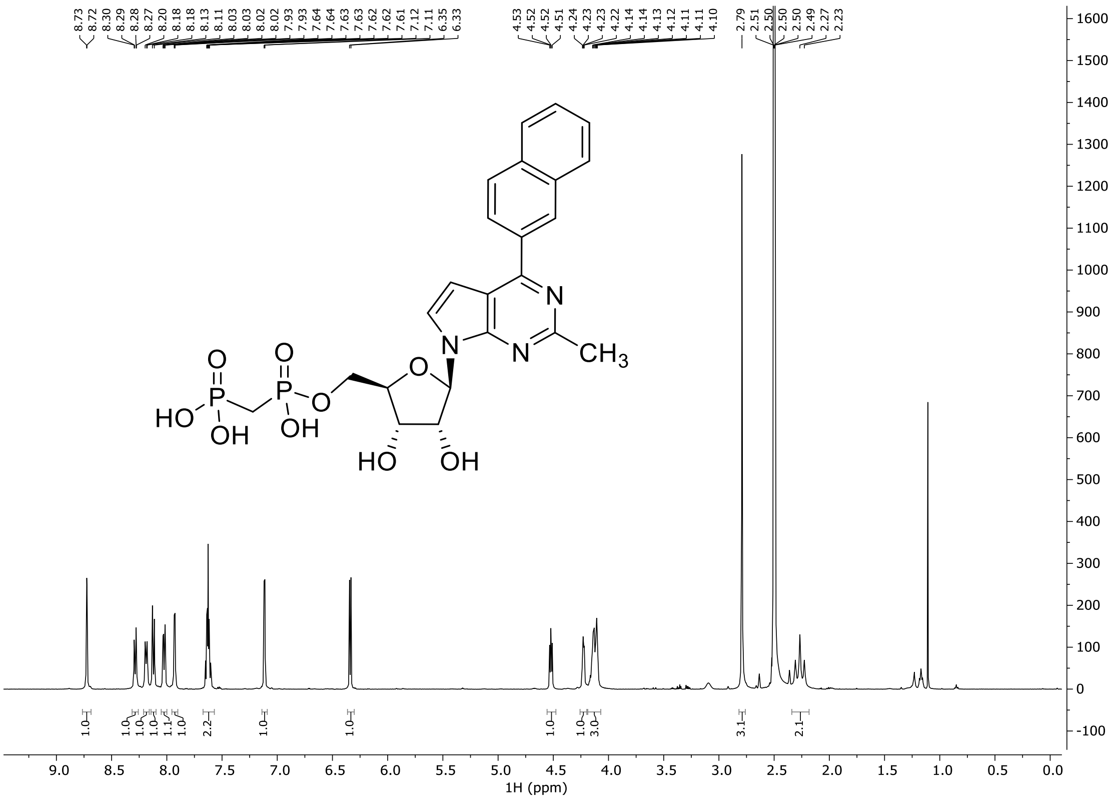

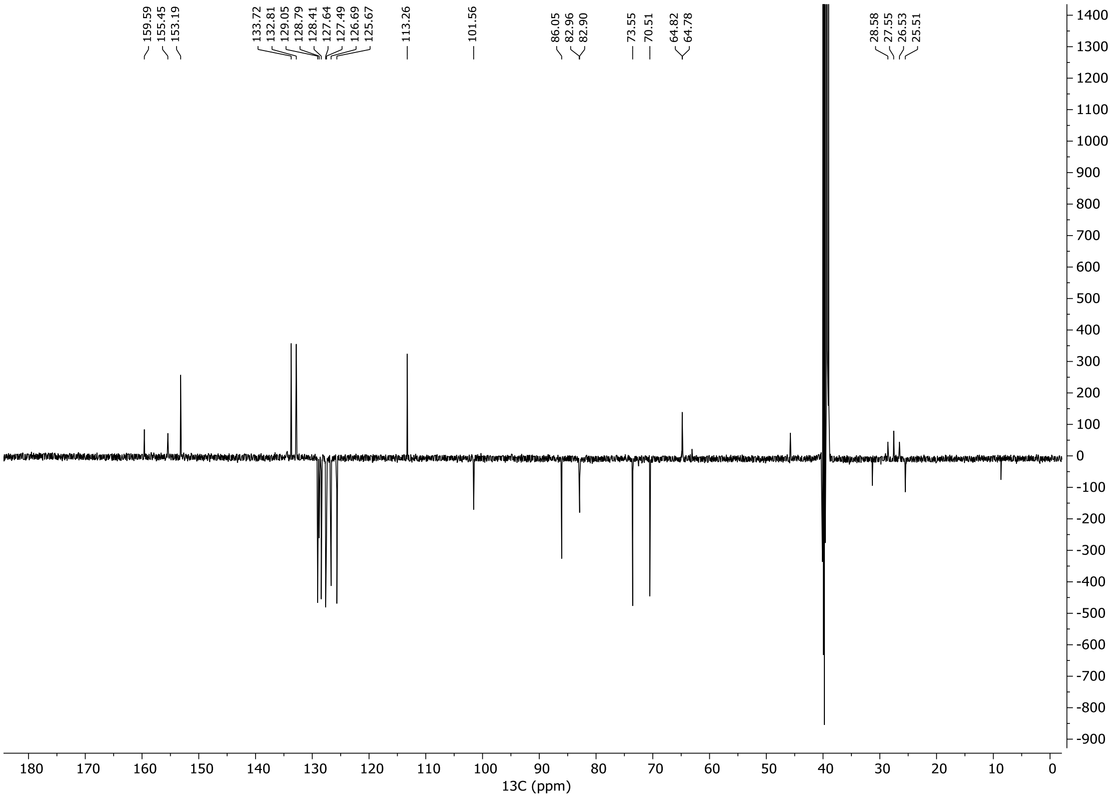

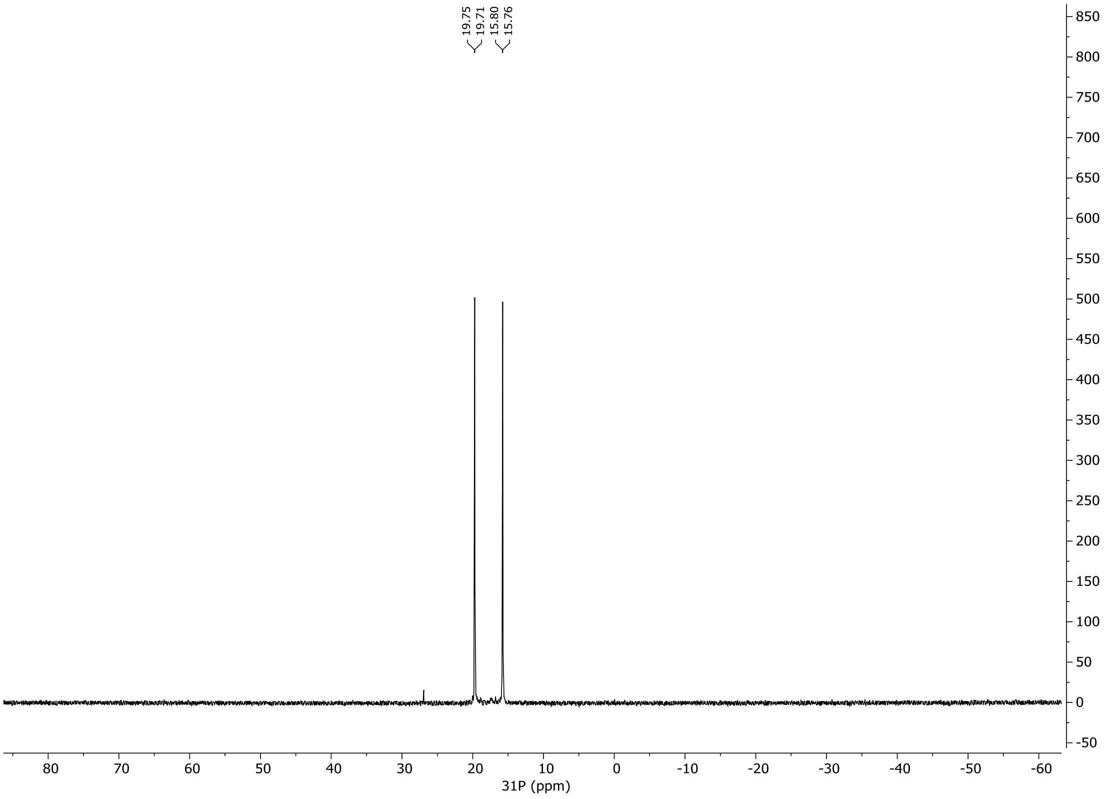

NMR spectra of compound **18E.8**

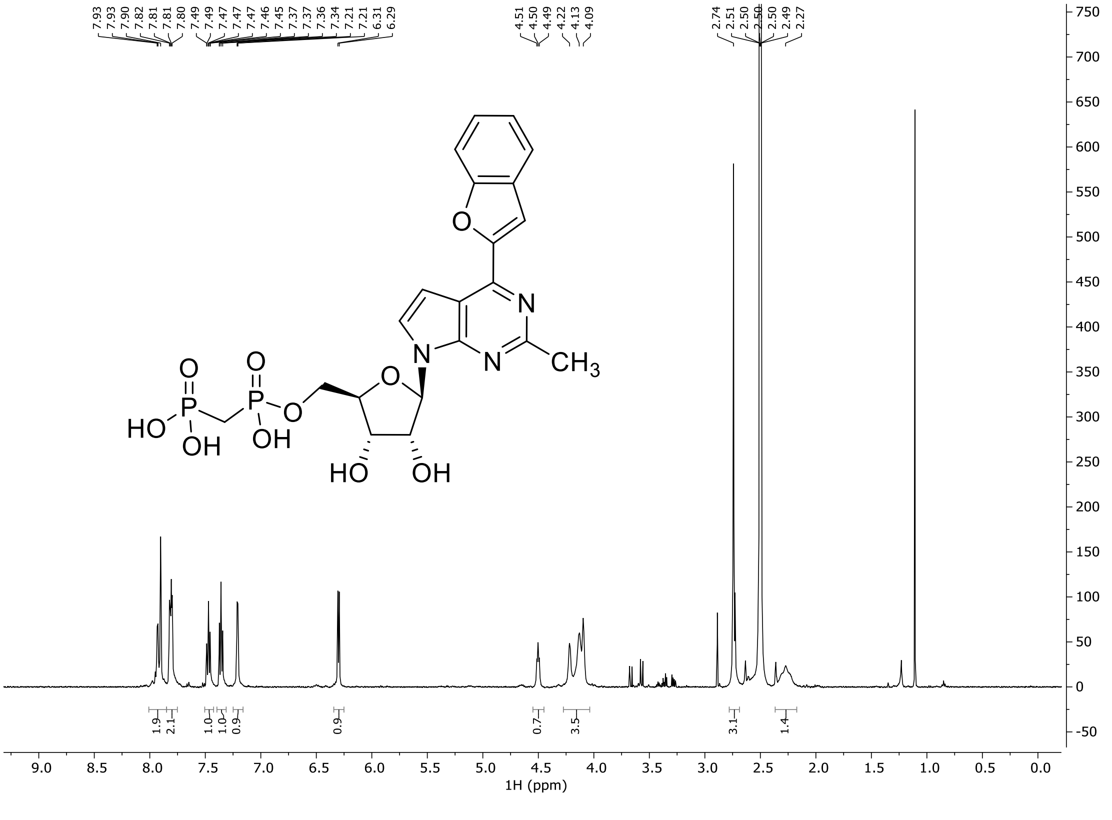

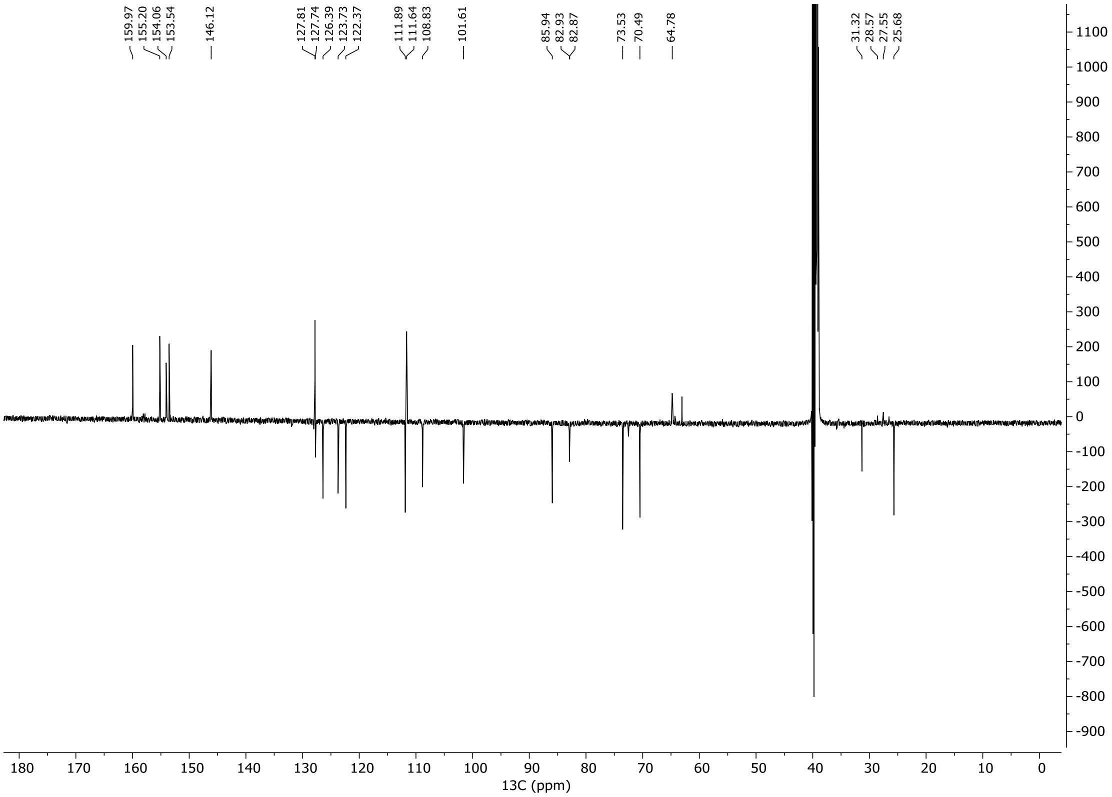
159.97
155.20
154.06
153.54
146.12
127.81
127.74
126.39
123.73
122.37
111.89
111.64
108.83
101.61
85.94
82.93
82.87
73.53
70.49
64.78
31.32
28.57
27.55
25.68
13C (ppm)

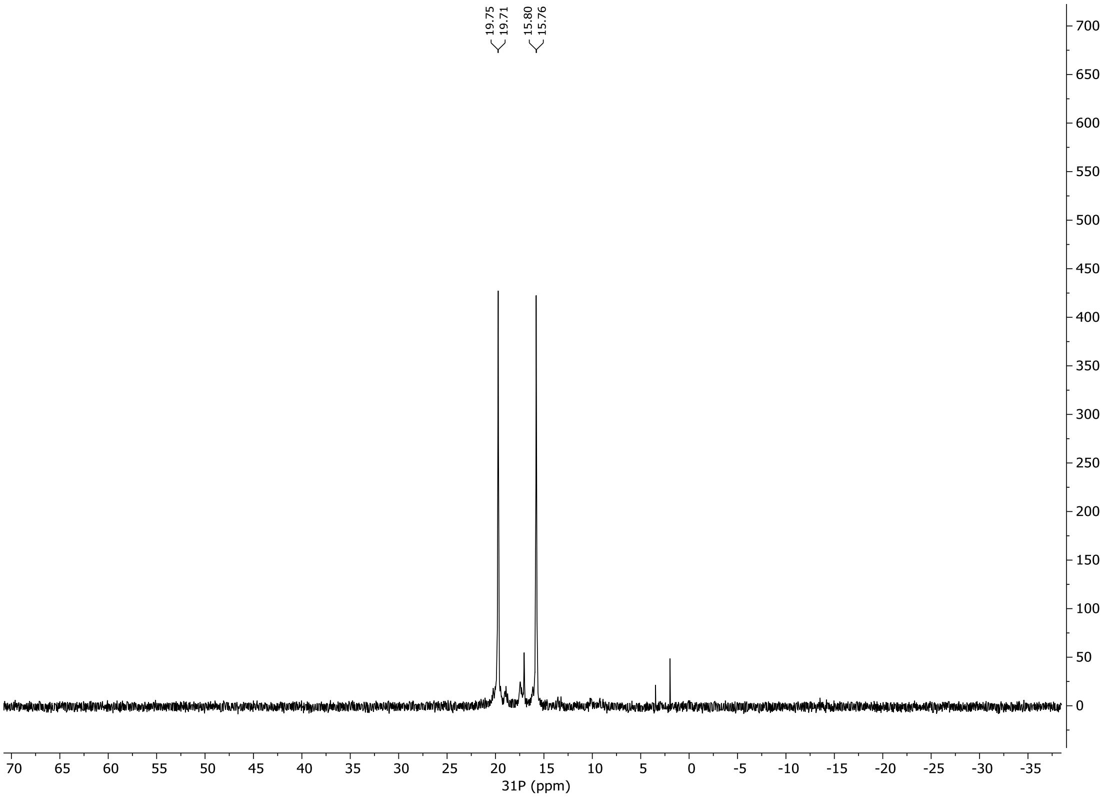
19.75
19.71
15.80
15.76
31P (ppm)

NMR spectra of compound **18E.12**

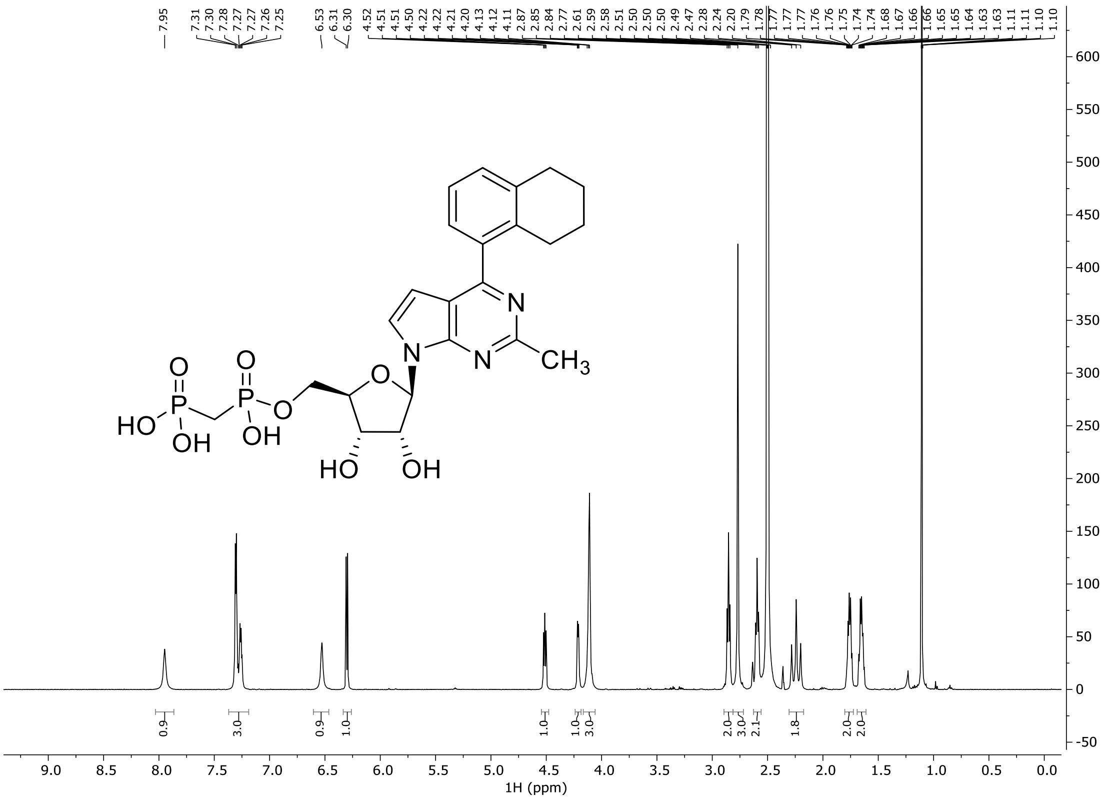

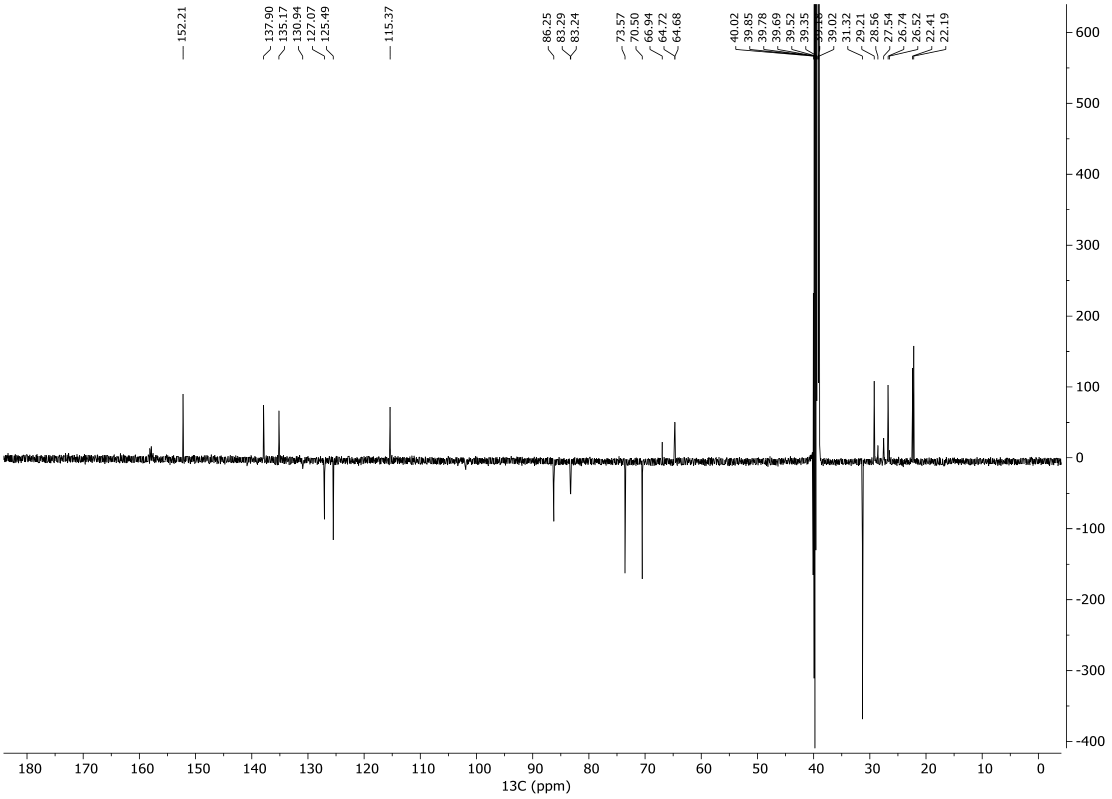

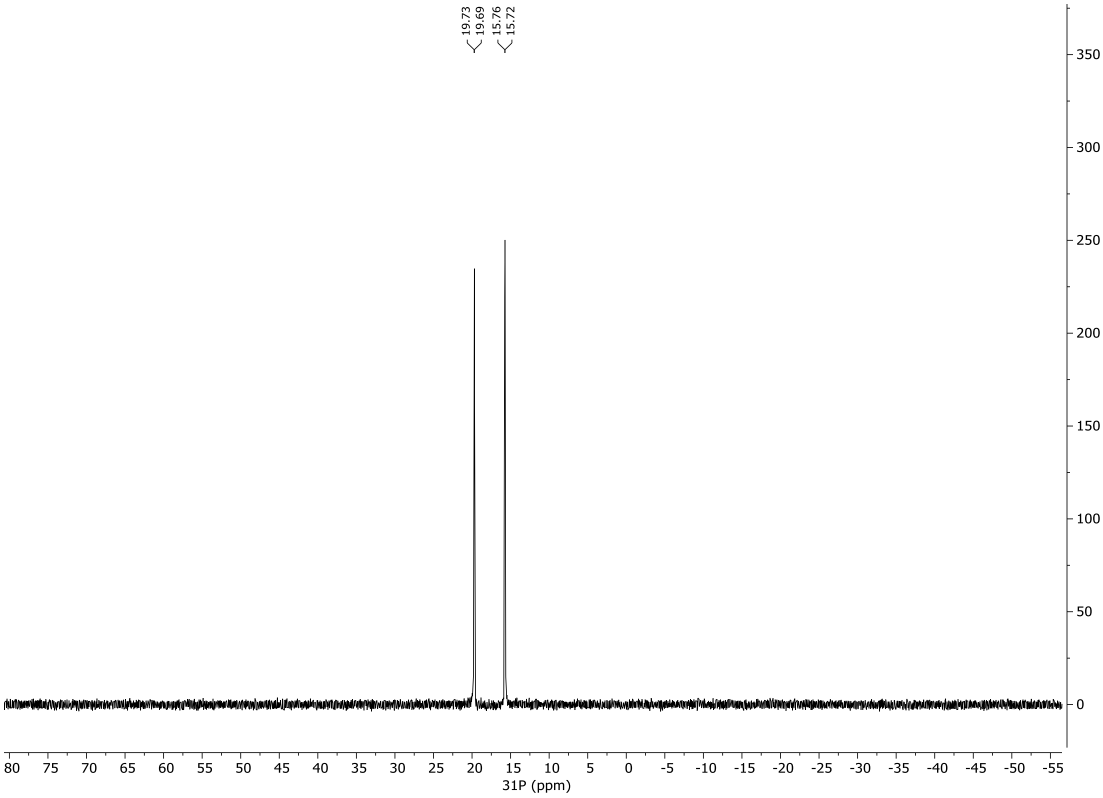

NMR spectra of compound **19F.7**

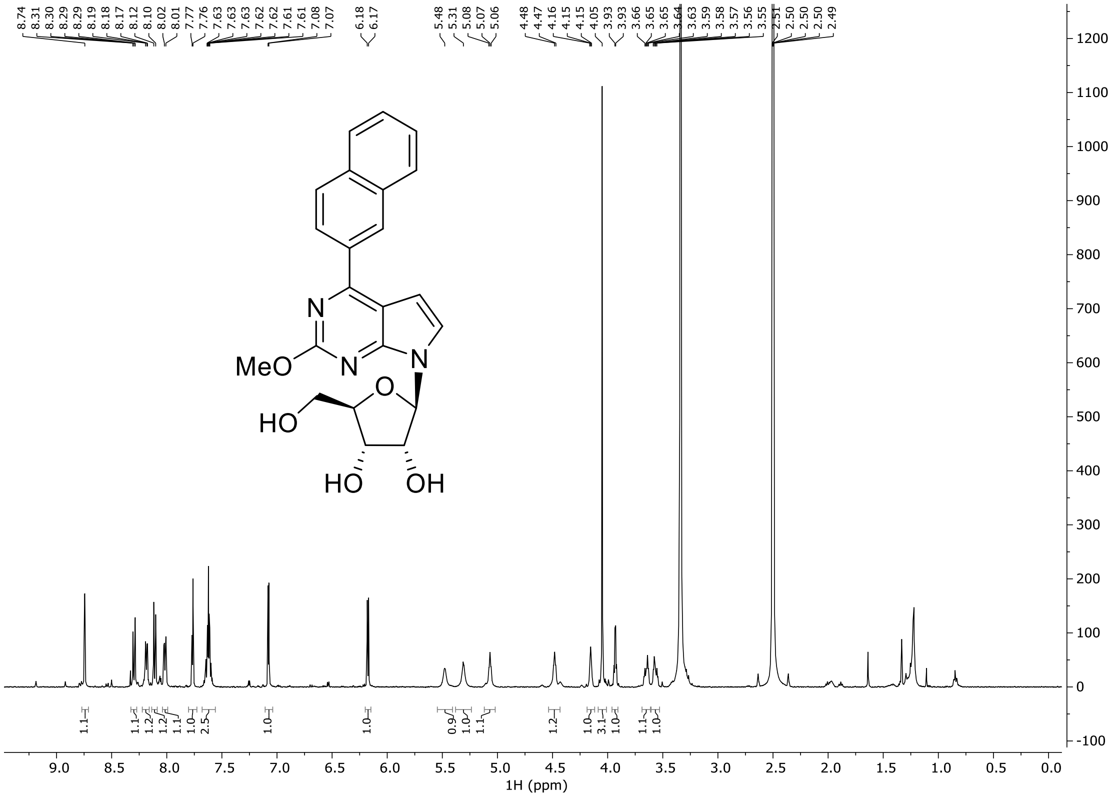

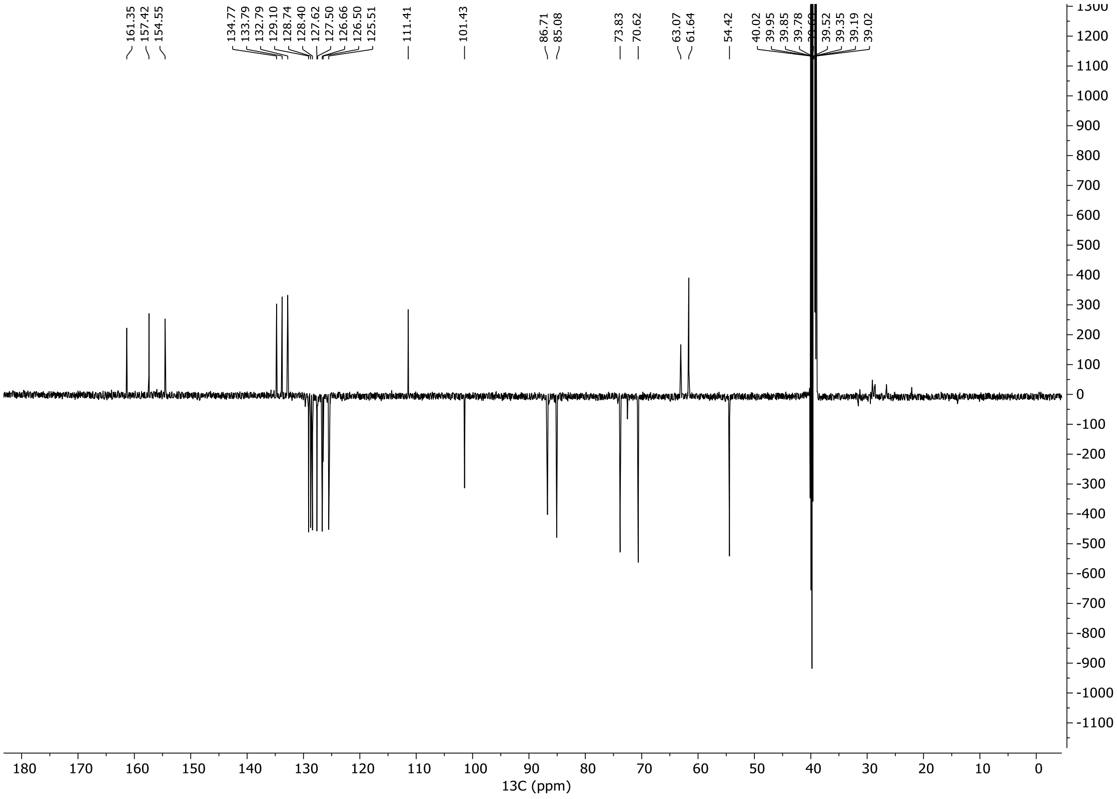

NMR spectra of compound **20G.7**

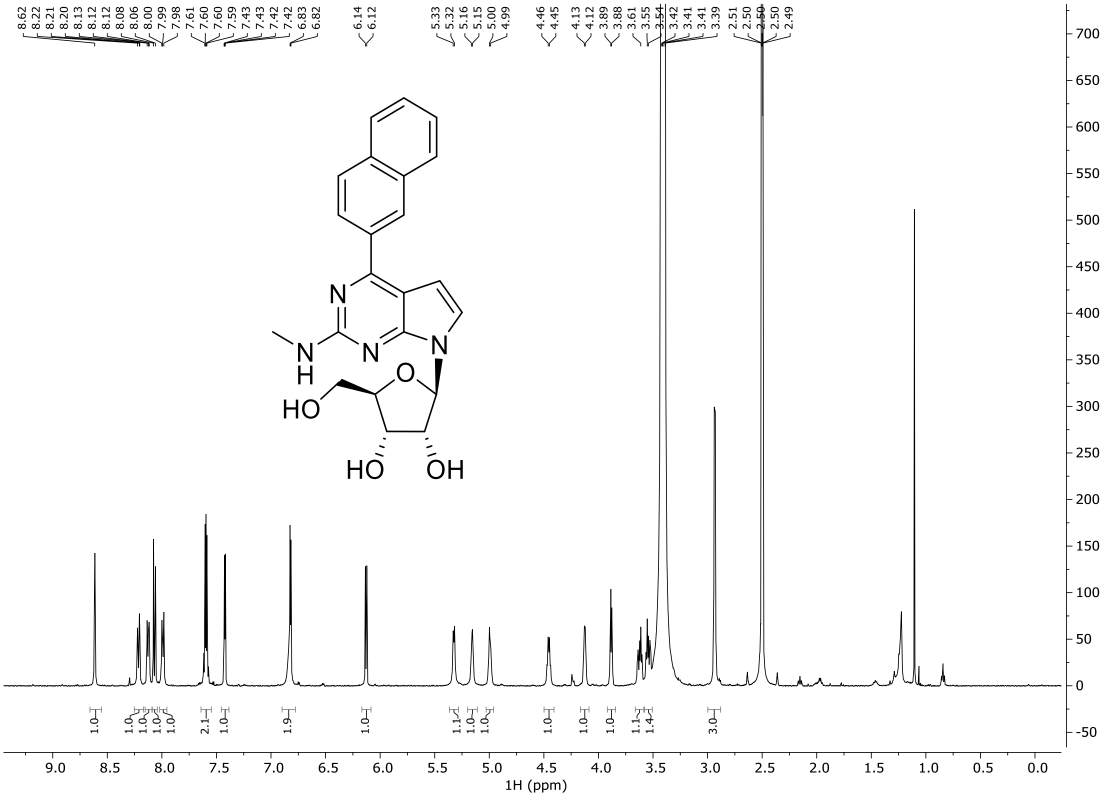

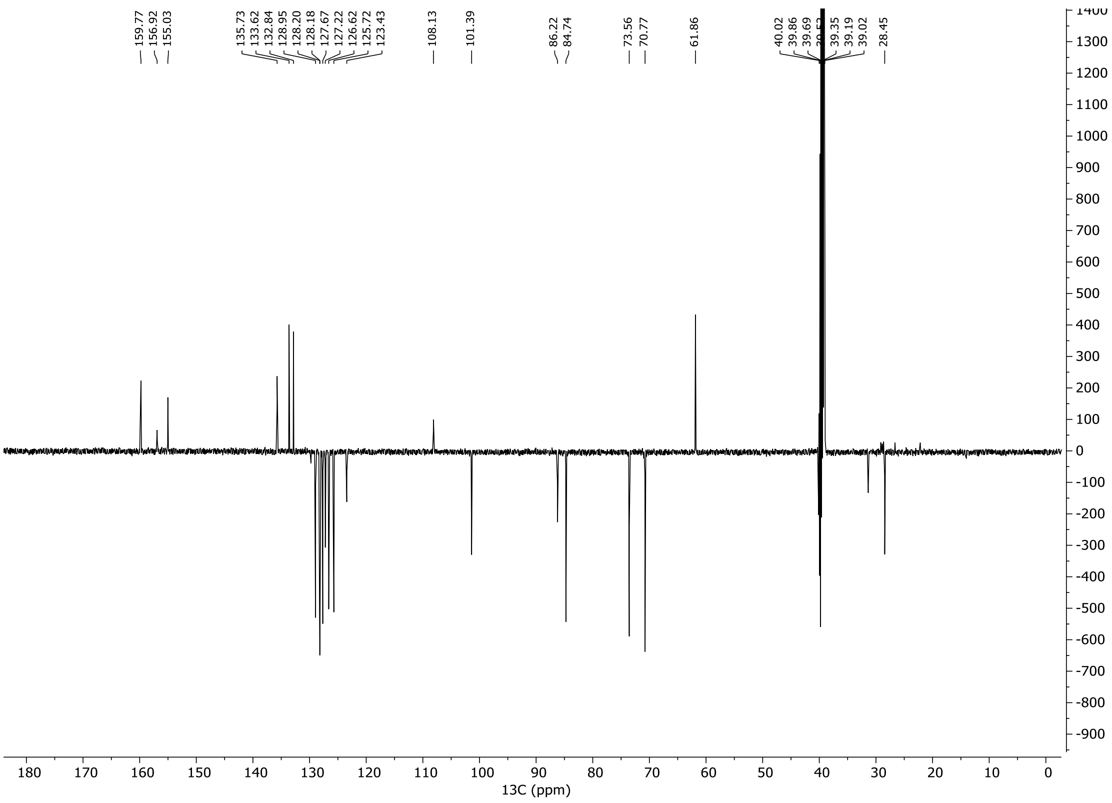

NMR spectra of compound **21F.7**

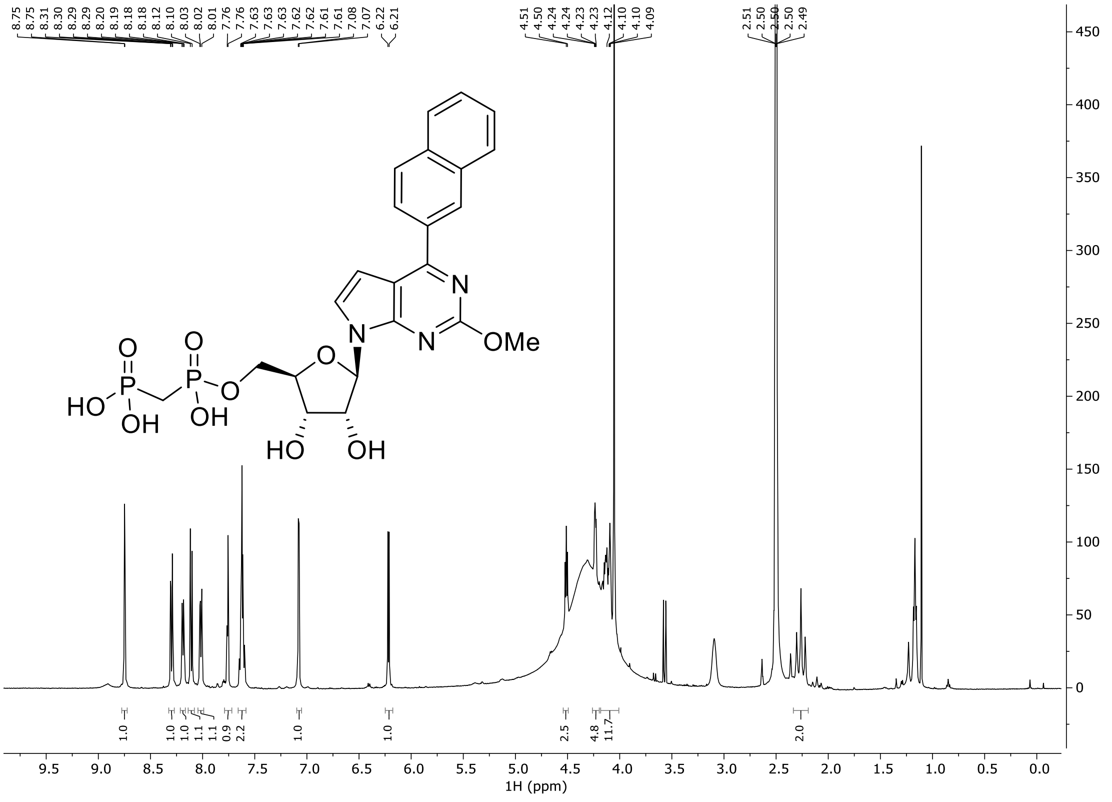

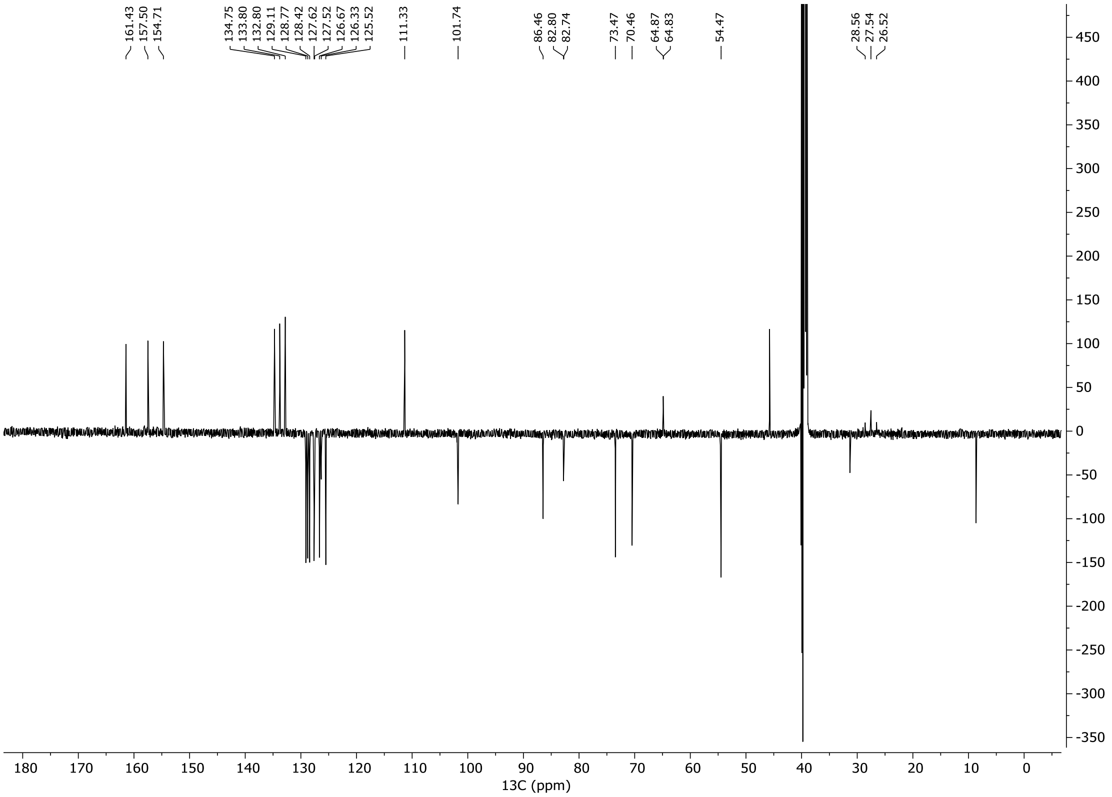
161.43
157.50
154.71
134.75
133.80
132.80
129.11
128.77
128.42
127.62
127.52
126.67
126.33
125.52
111.33
101.74
86.46
82.80
82.74
73.47
70.46
64.87
64.83
54.47
28.56
27.54
26.52
13C (ppm)

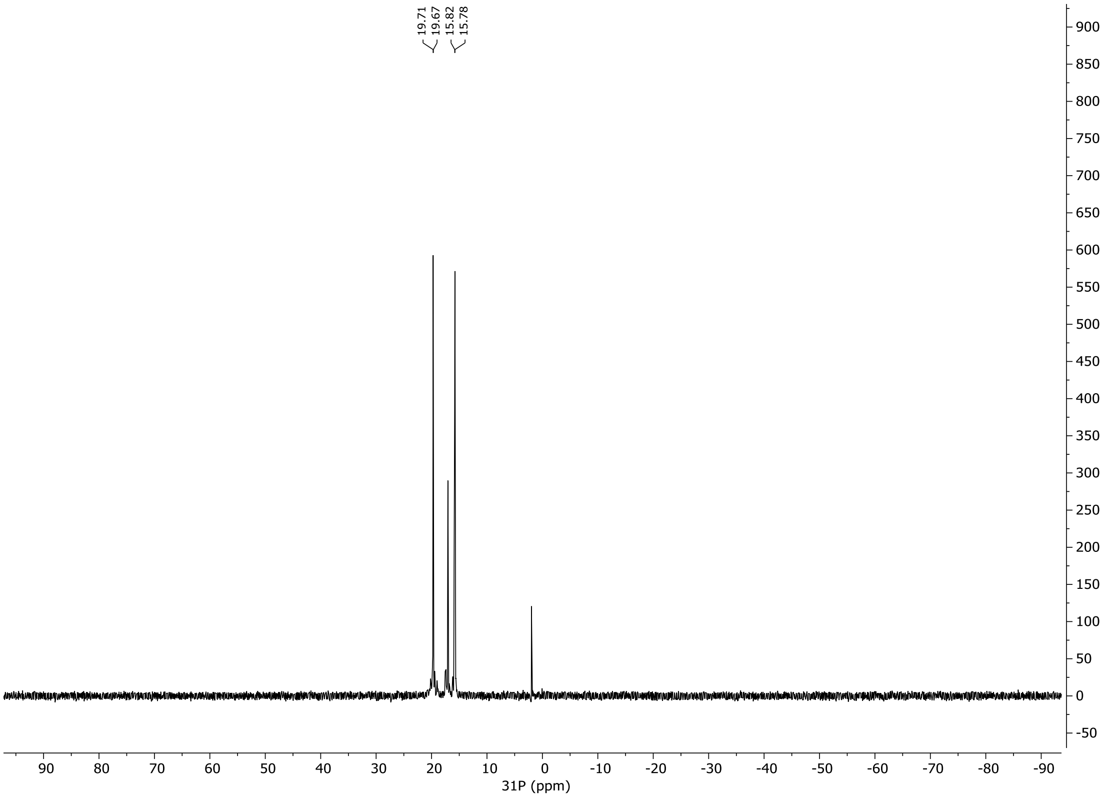
19.71
19.67
15.82
15.78
31P (ppm)

NMR spectra of compound **22G.7**

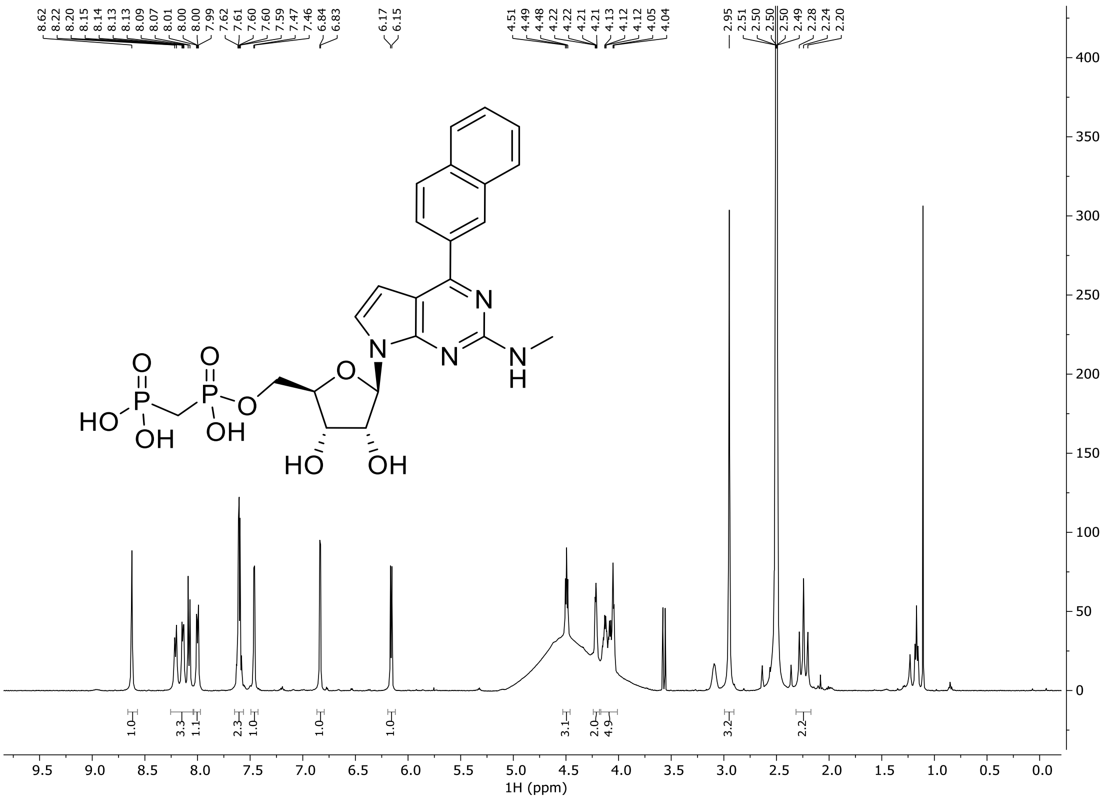

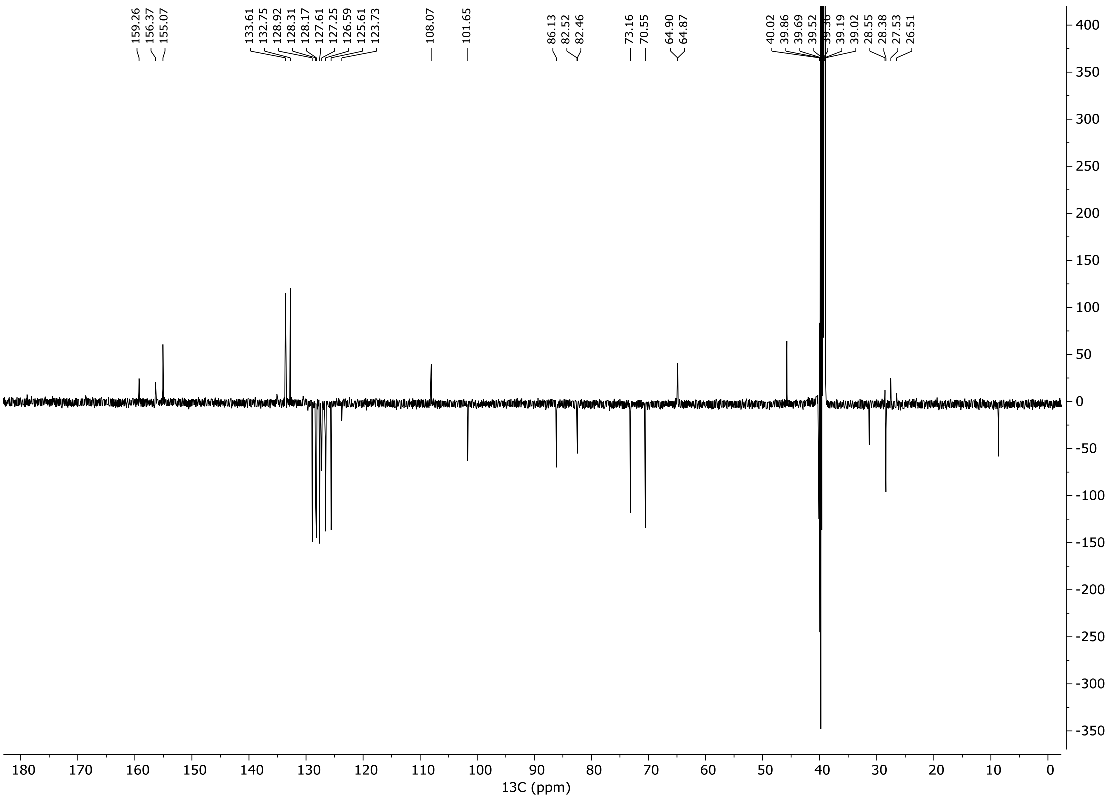

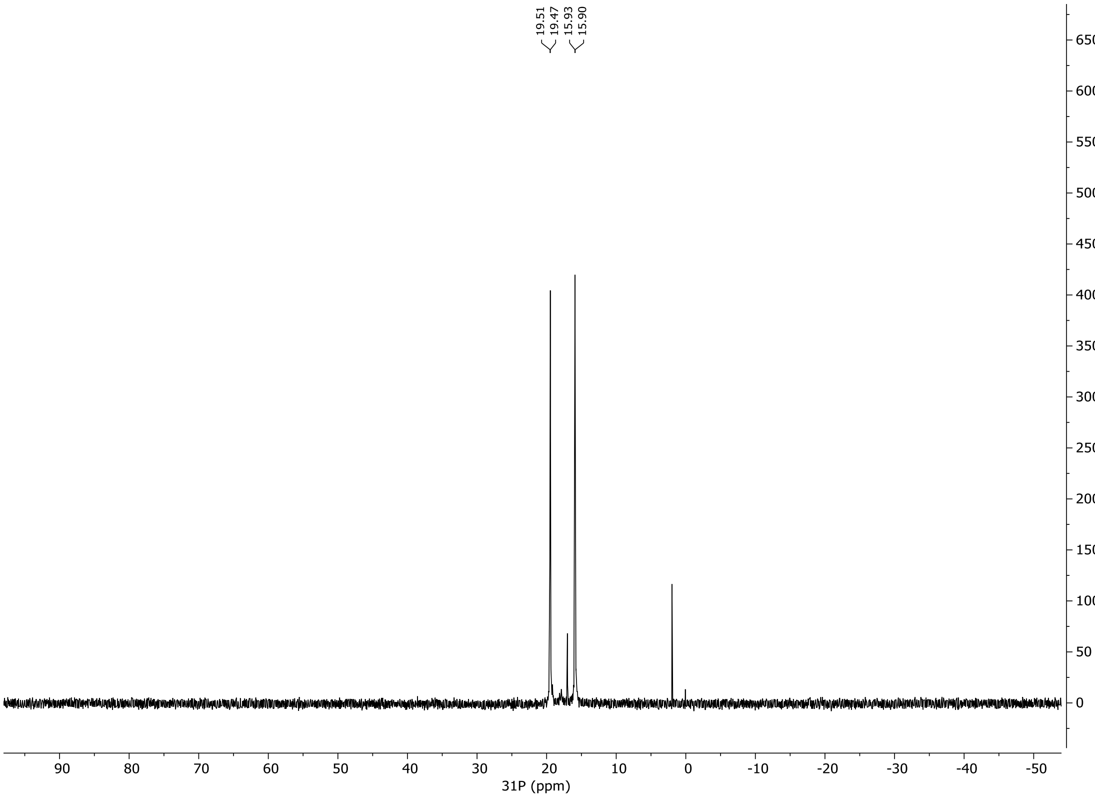

NMR spectra of compound **24C.23**

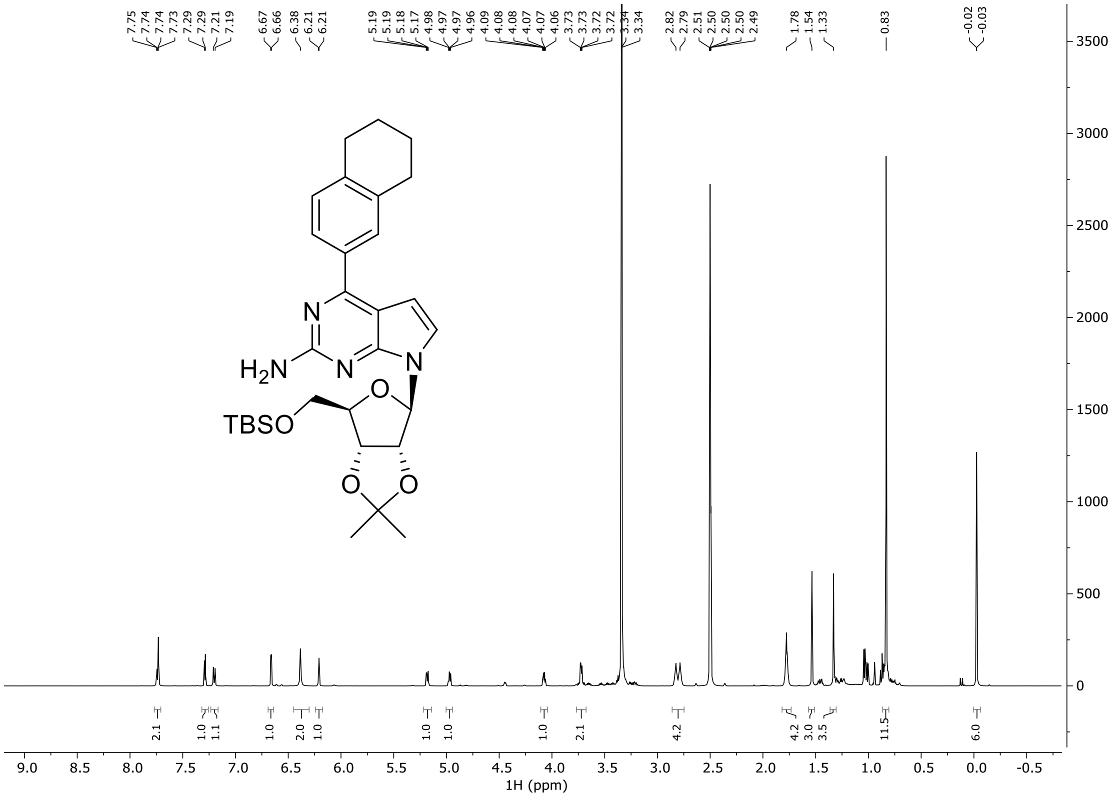

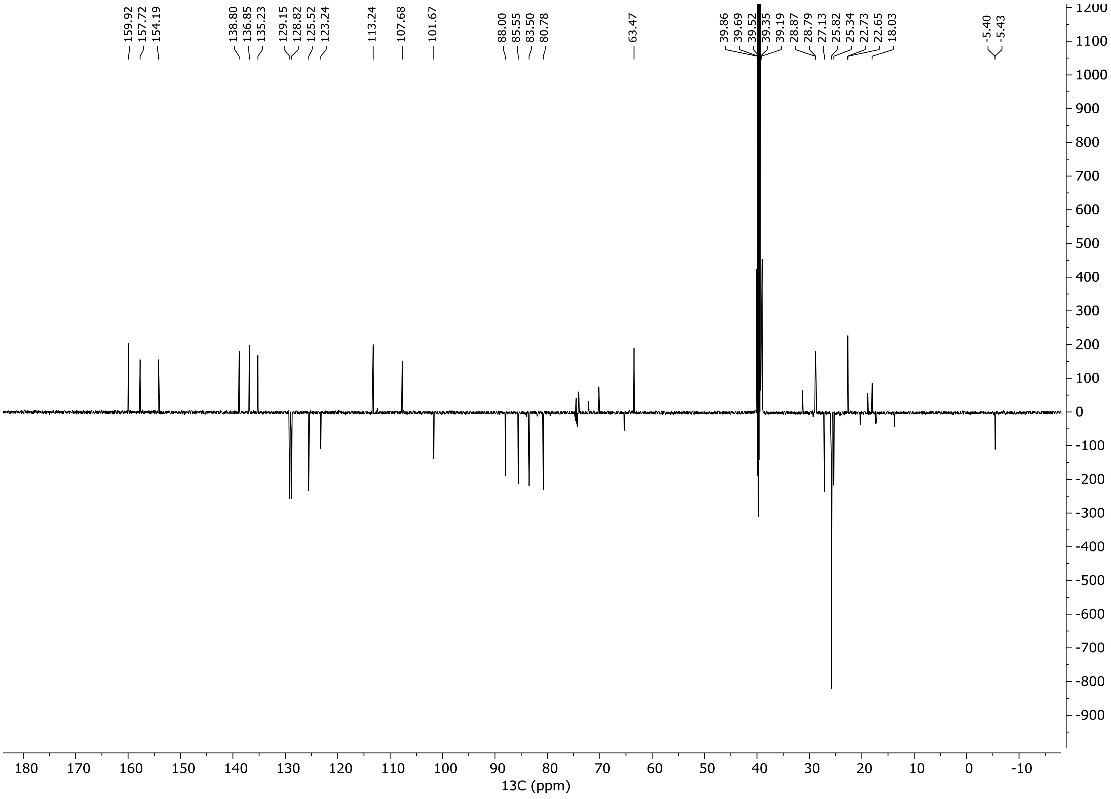

NMR spectra of compound **25H.23**

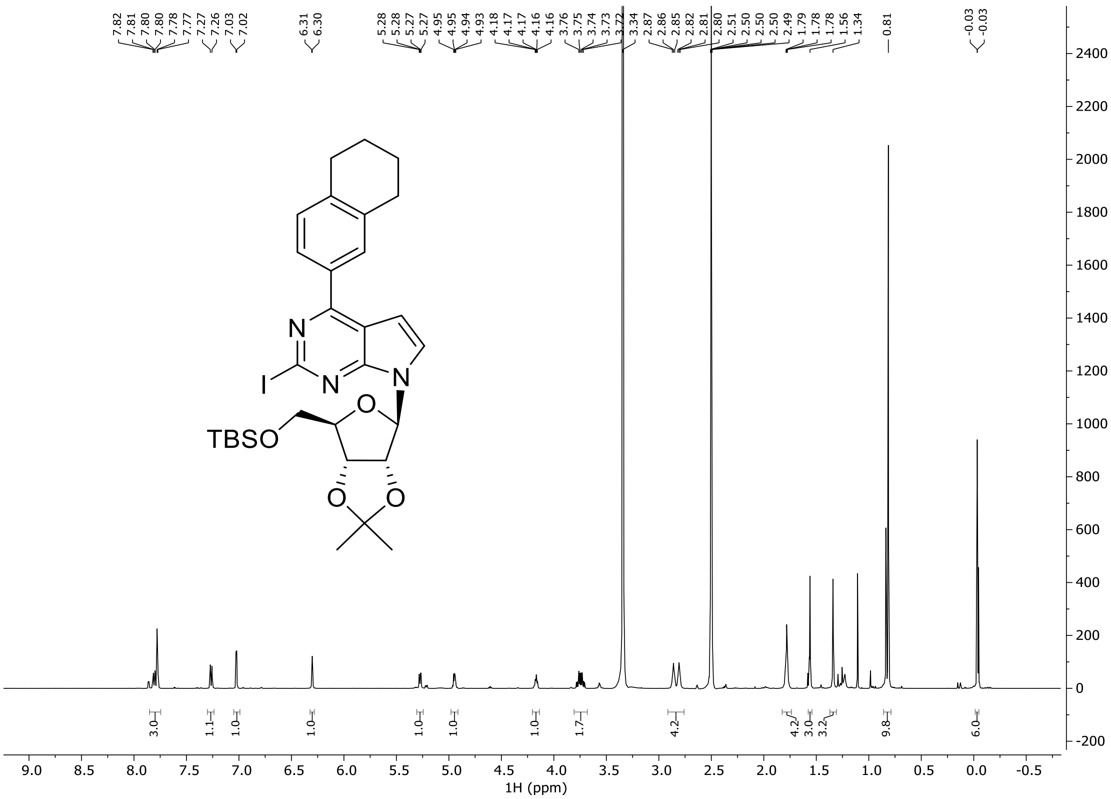

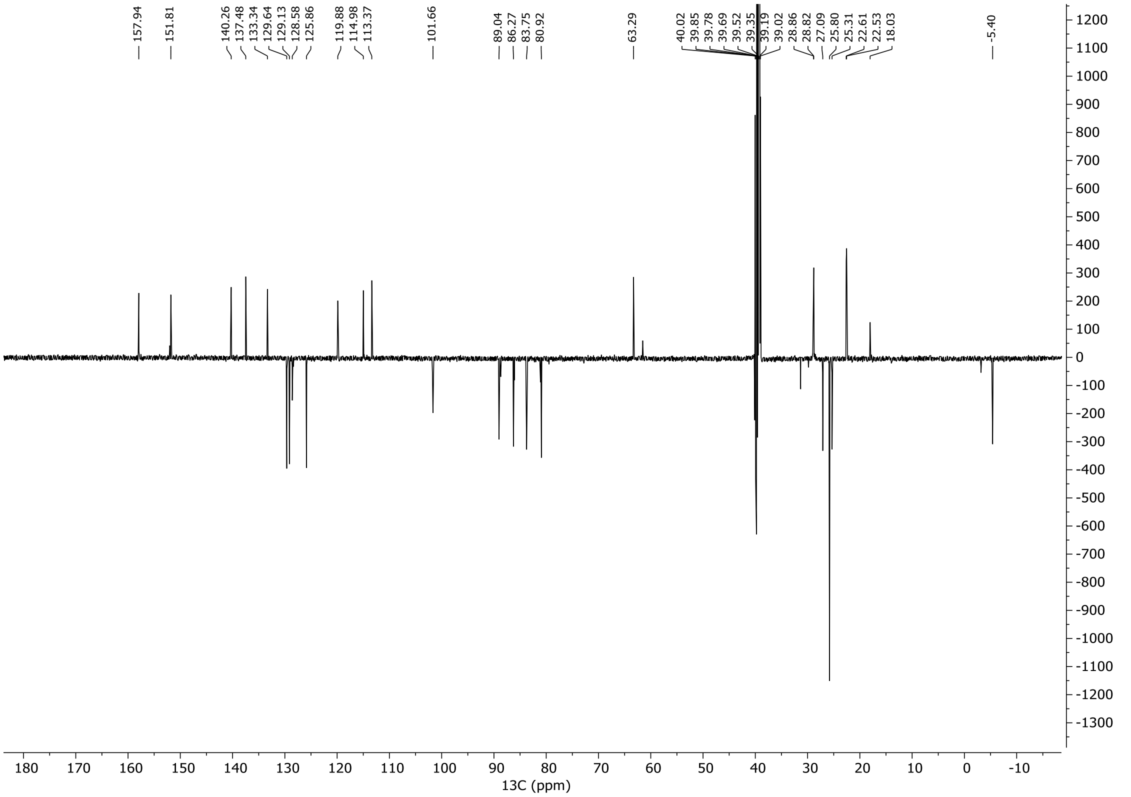

NMR spectra of compound **26H.23**

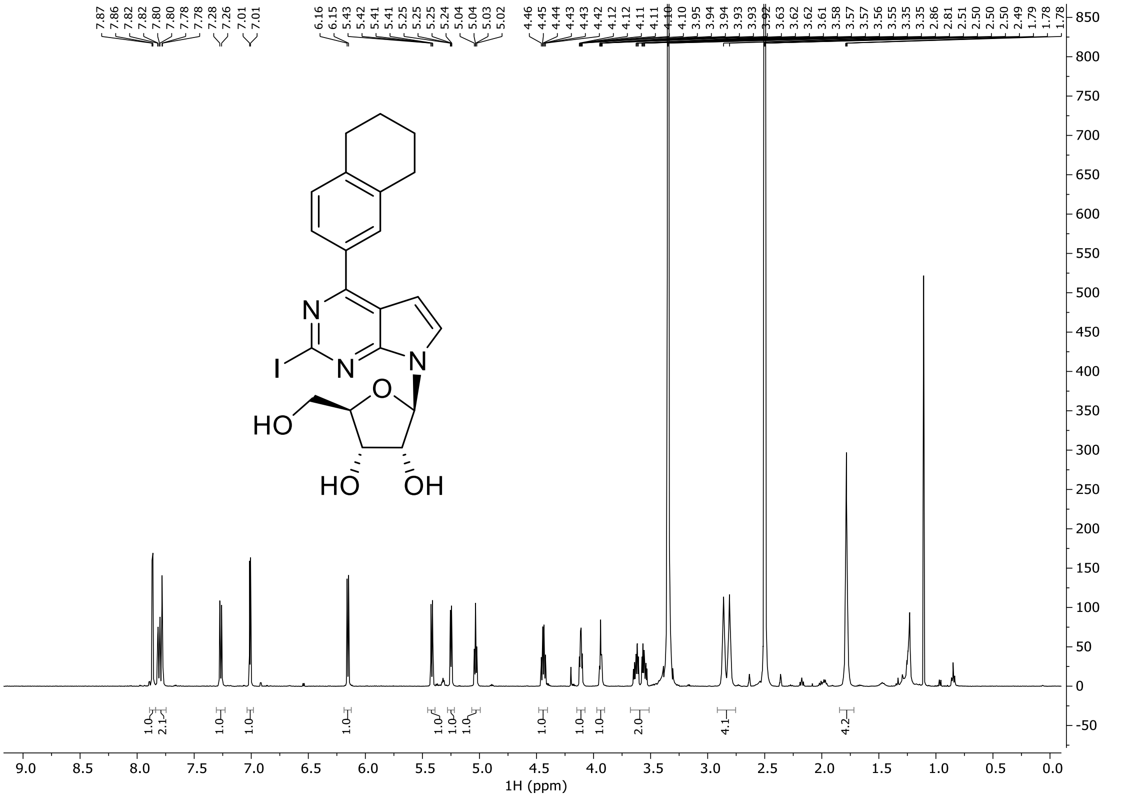

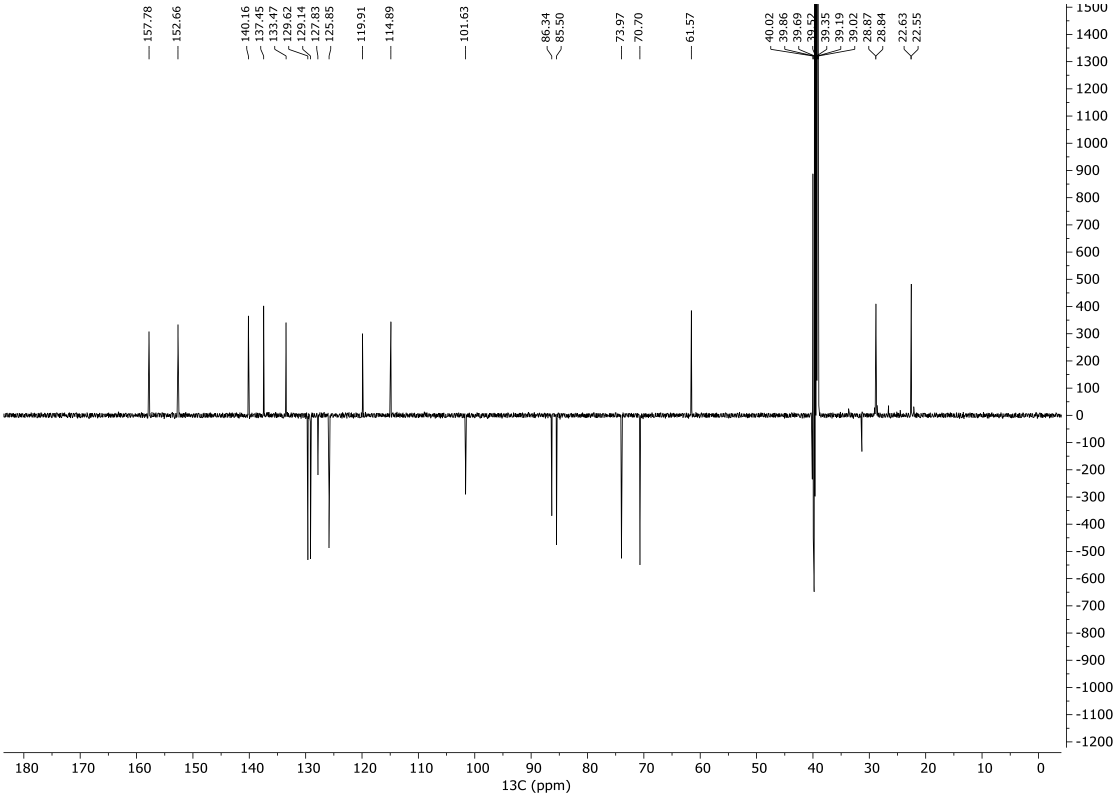

NMR spectra of compound **27H.23**

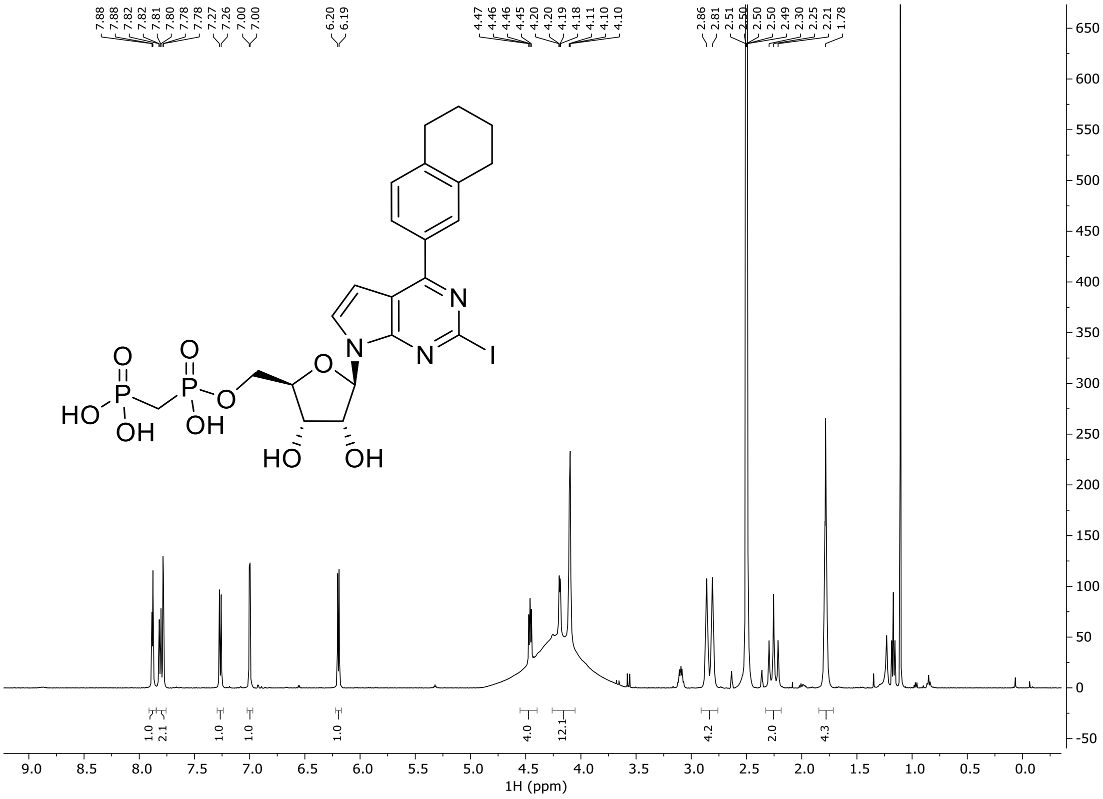

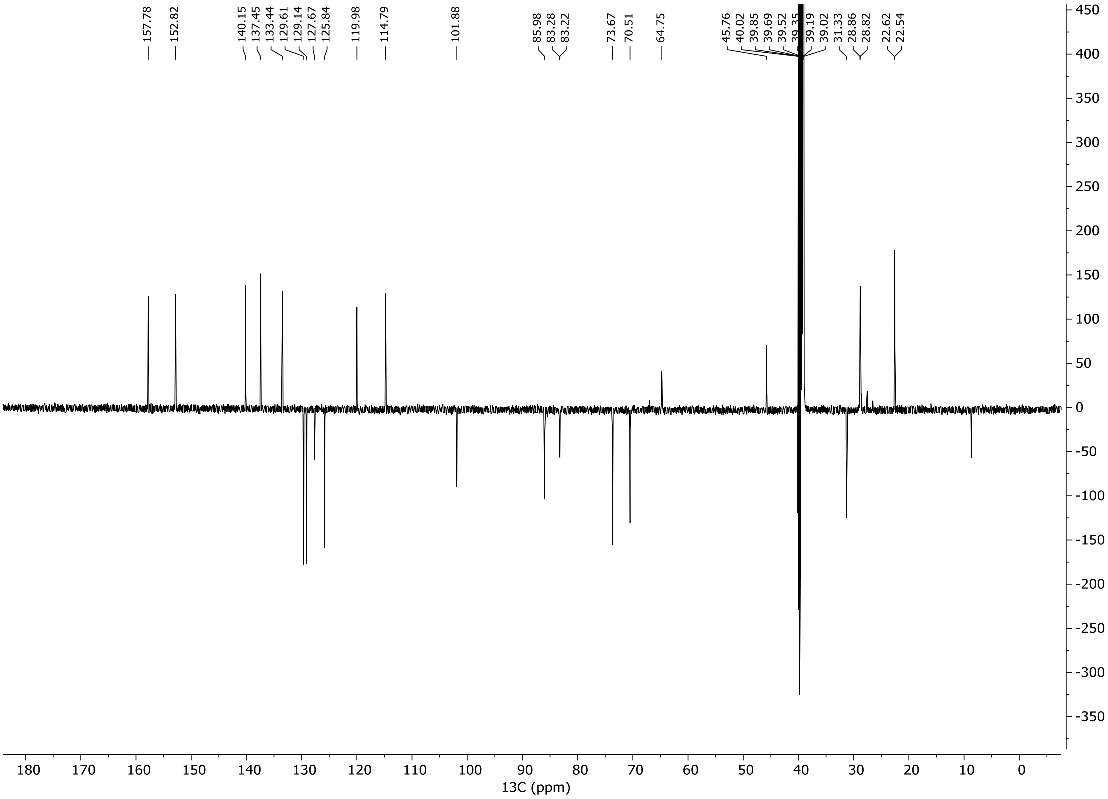

157.78
152.82
140.15
137.45
133.44
129.61
129.14
127.67
125.84
119.98
114.79
101.88
85.98
83.28
83.22
73.67
70.51
64.75
45.76
40.02
39.85
39.69
39.52
39.35
39.19
39.02
31.33
28.86
28.82
22.62
22.54
13C (ppm)

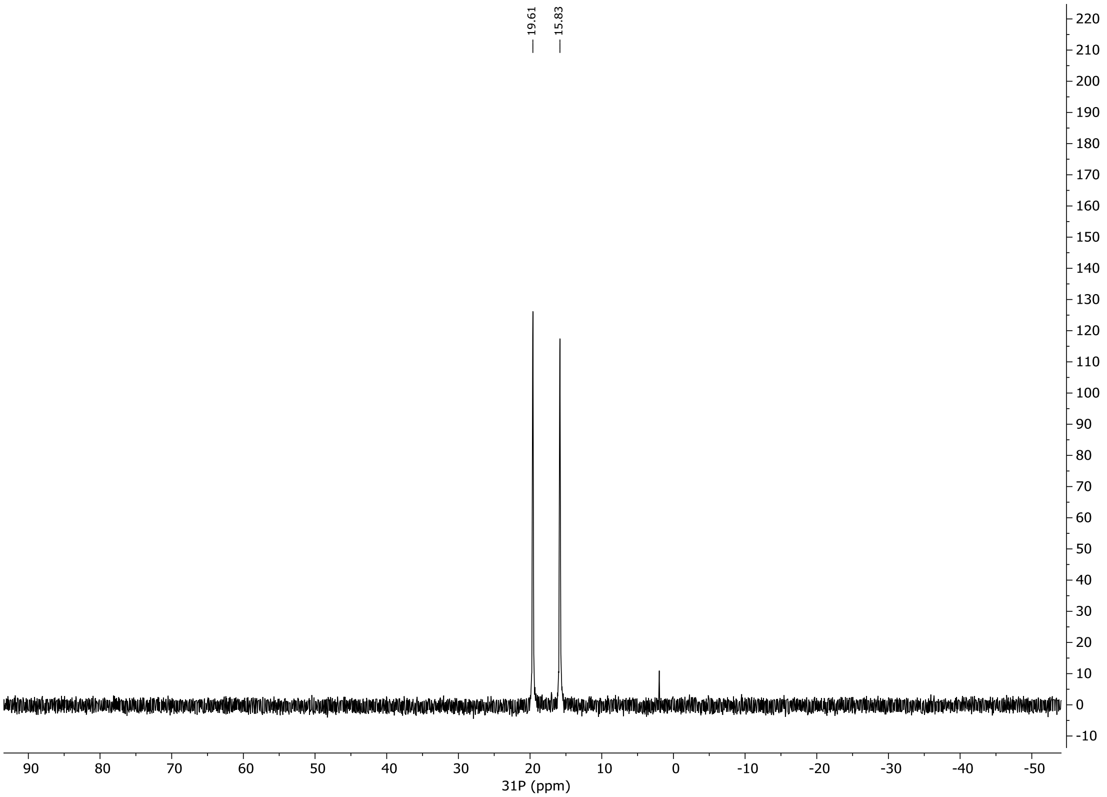

19.61
15.83
31P (ppm)

NMR spectra of compound **29**

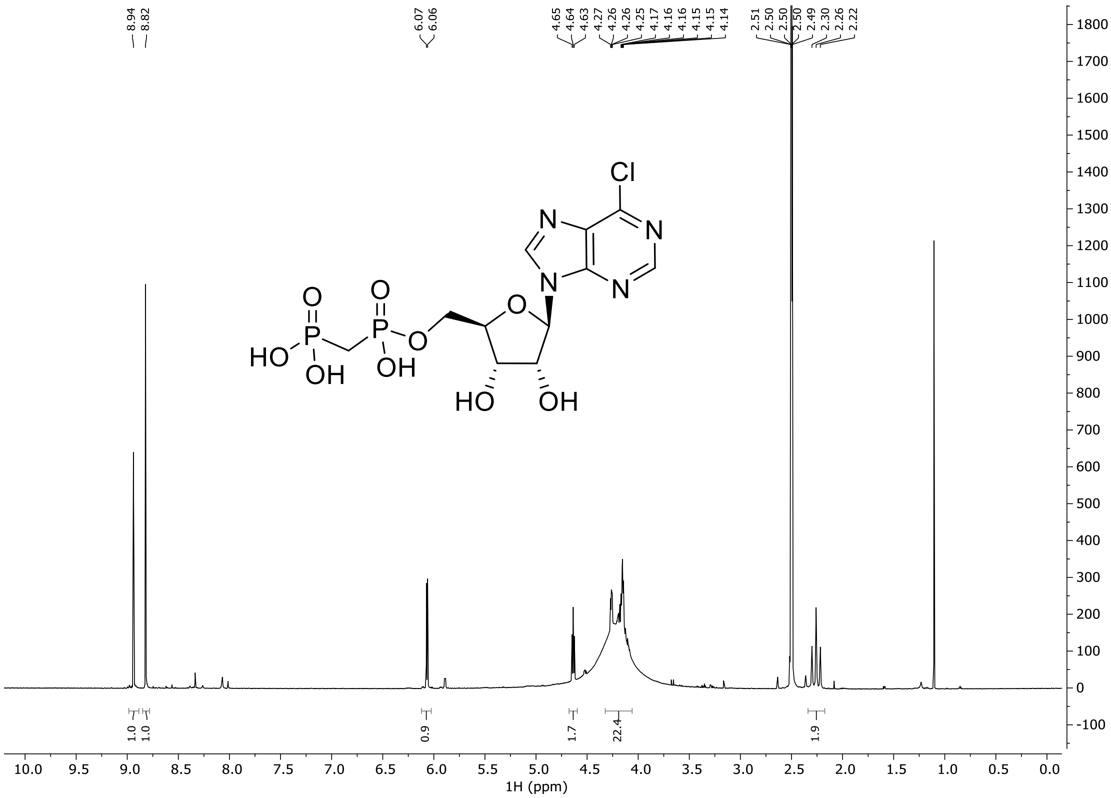

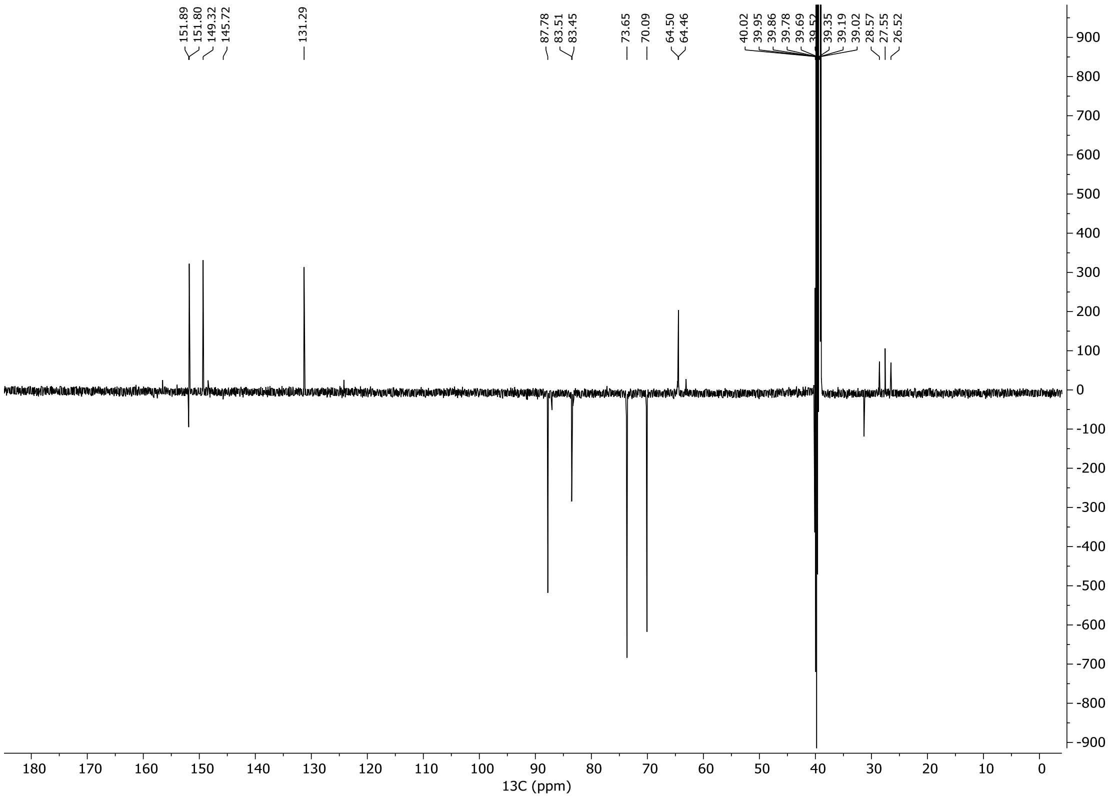

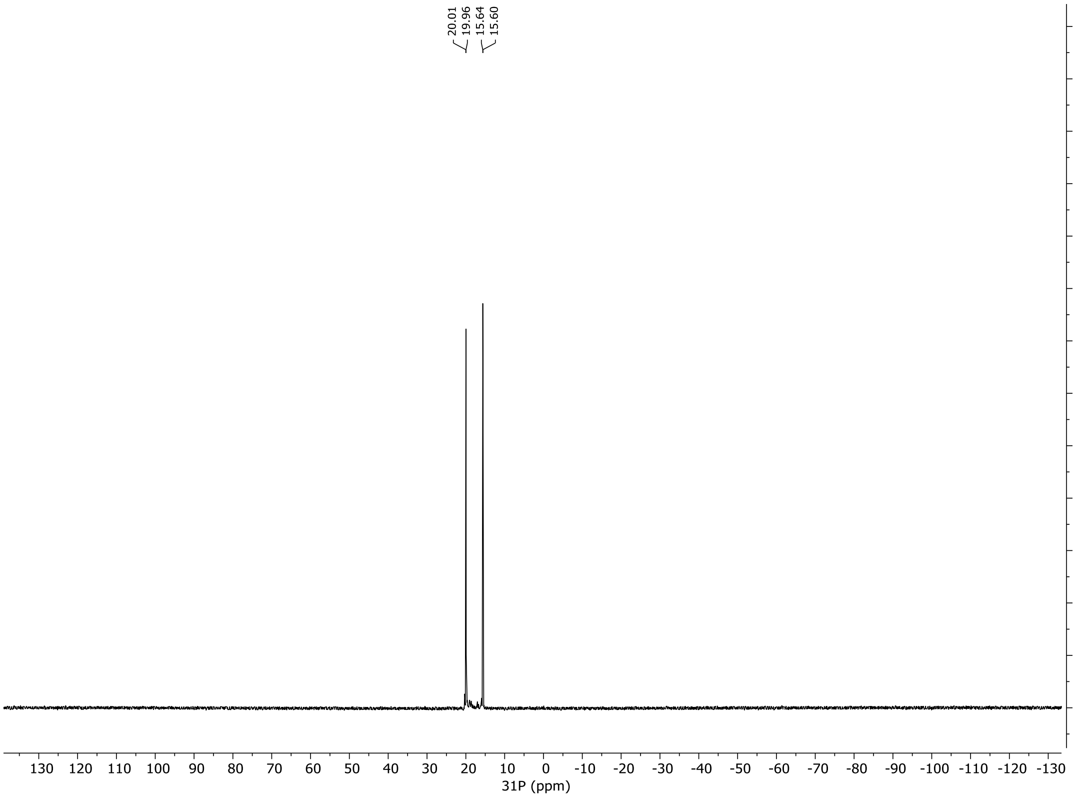

NMR spectra of compound **30A.6**

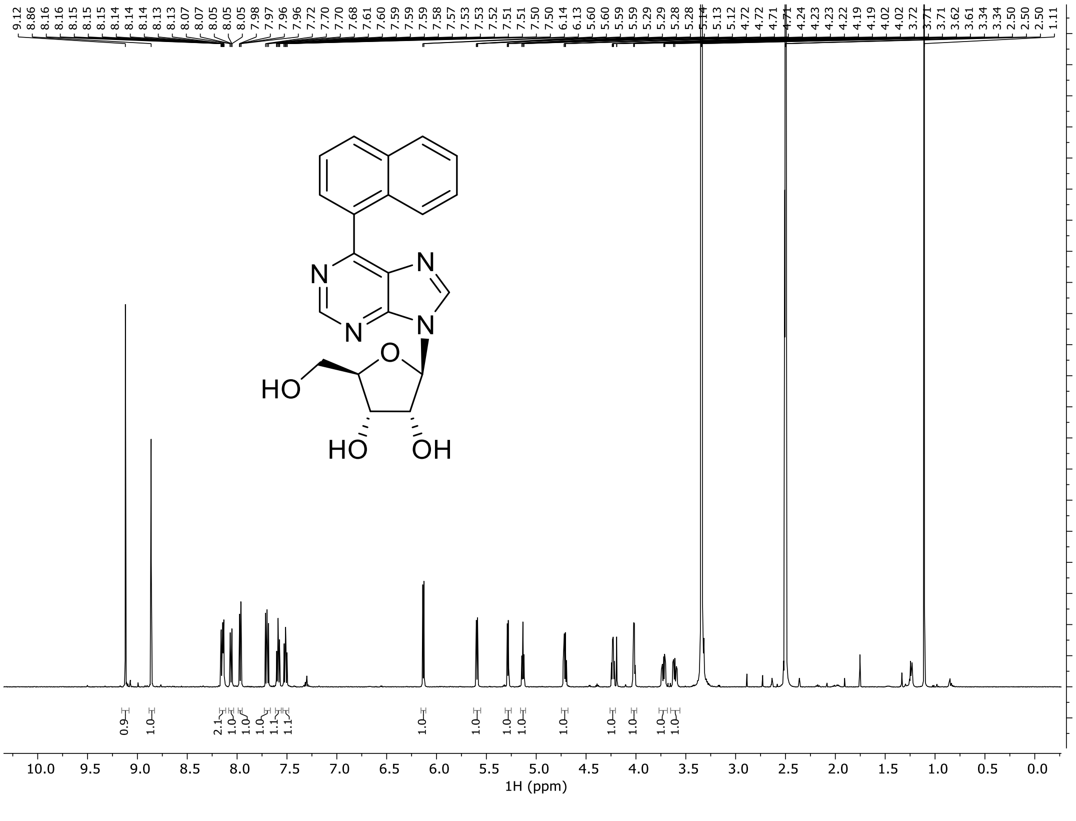

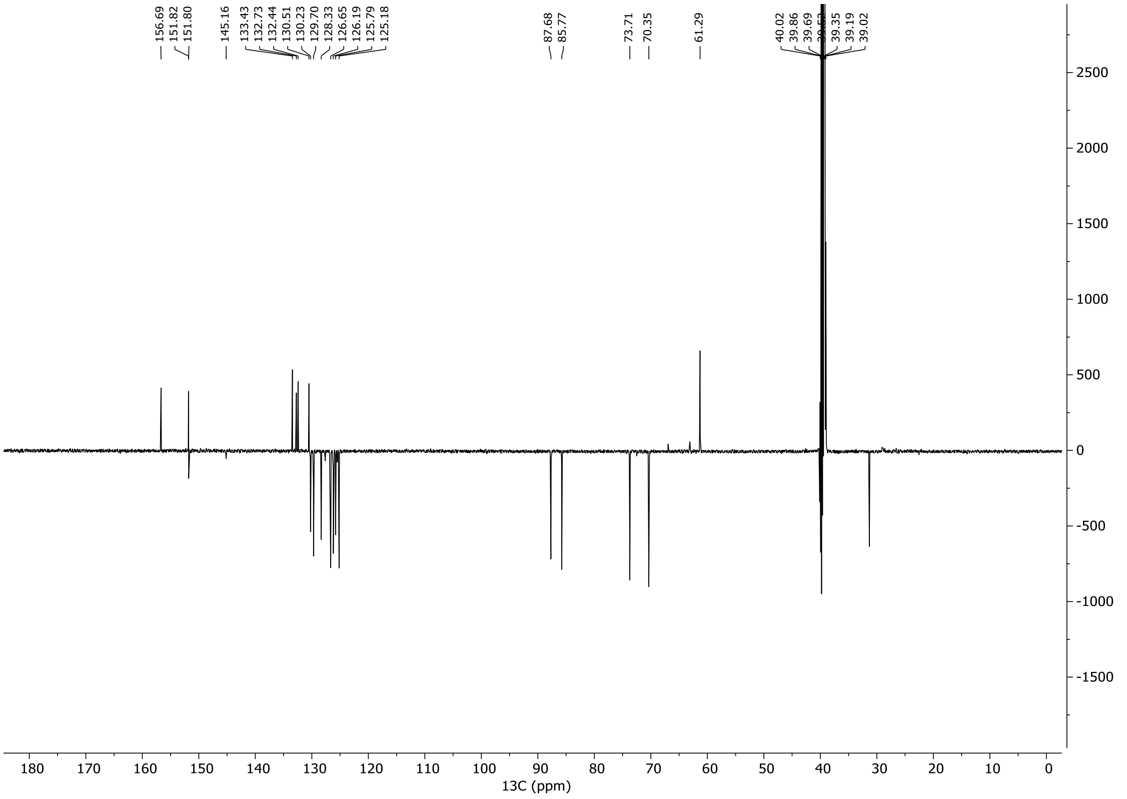

NMR spectra of compound **30A.12**

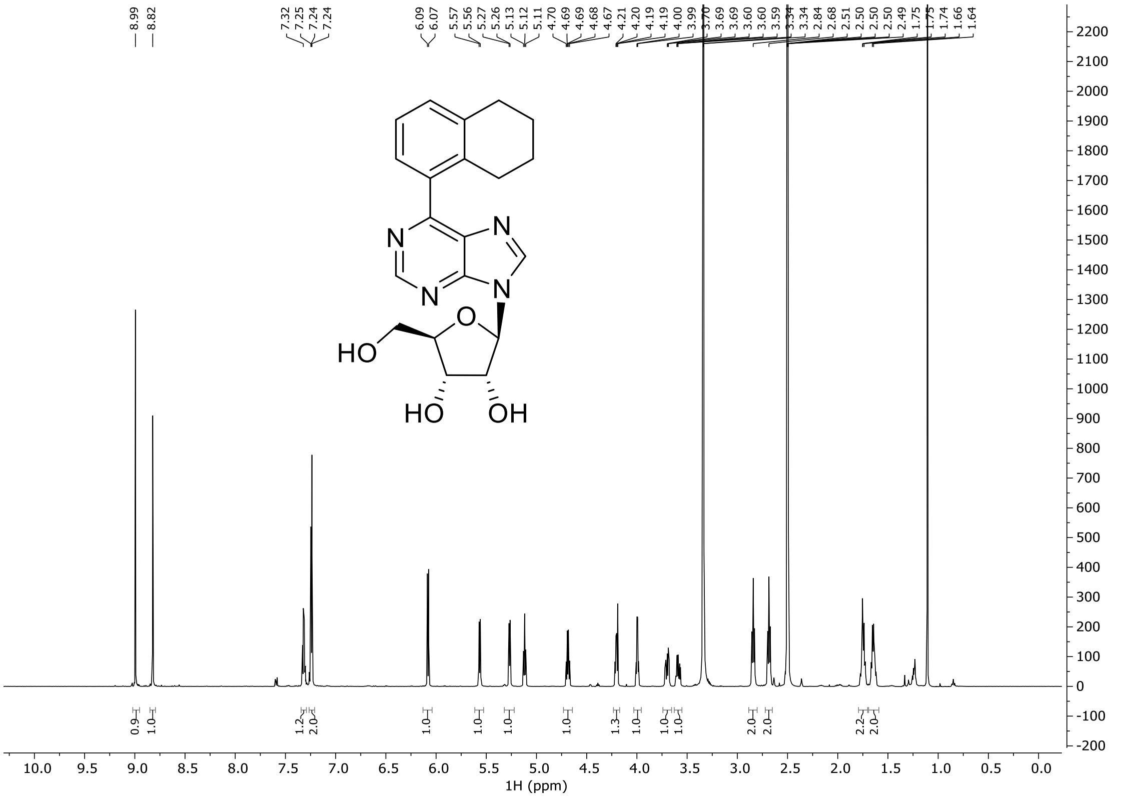

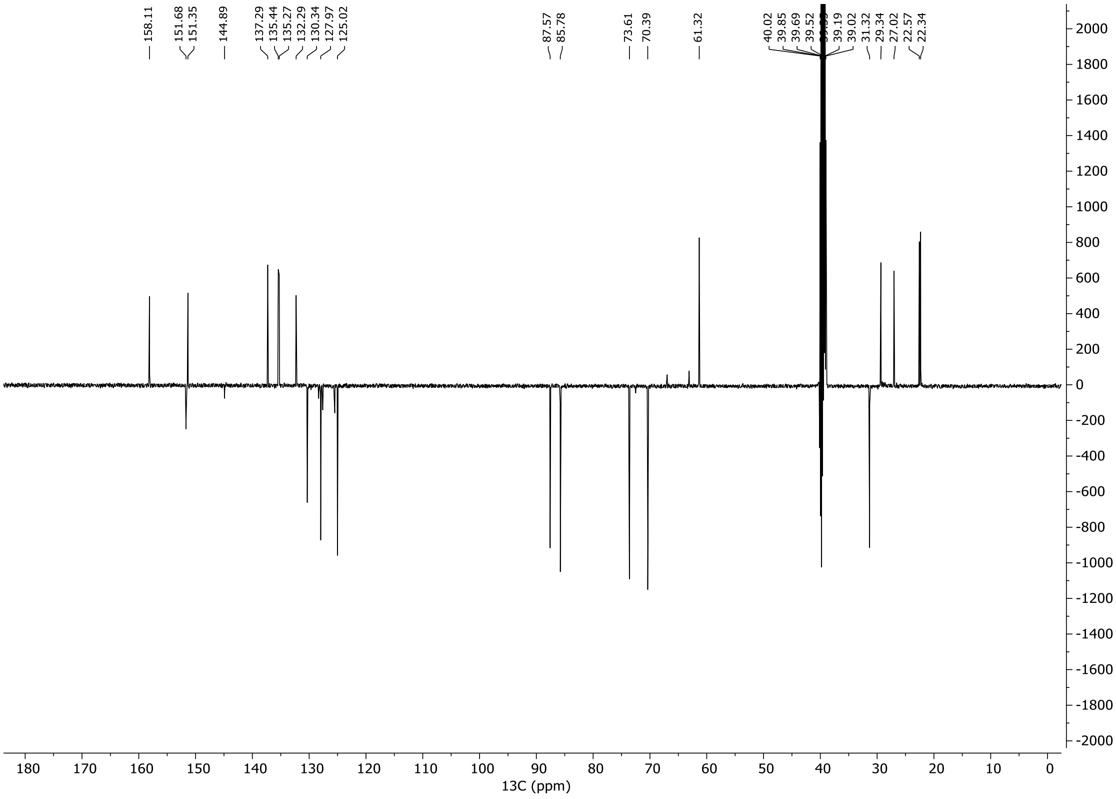

NMR spectra of compound **31A.6**

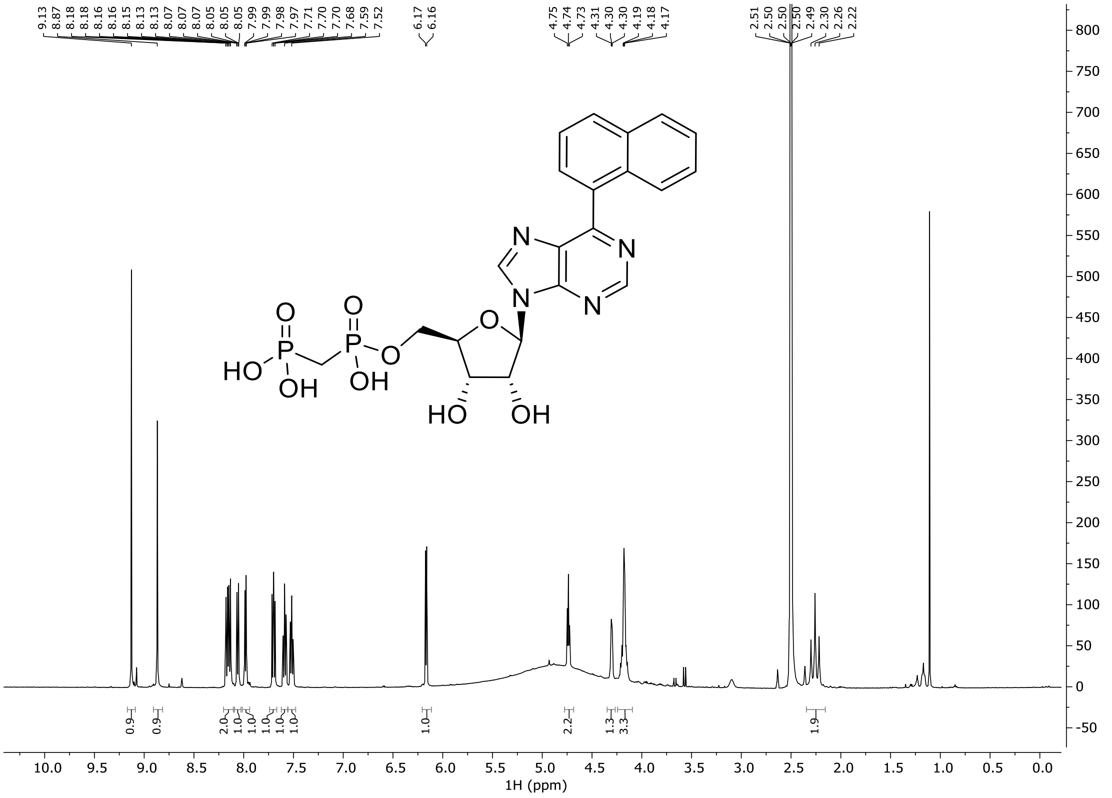

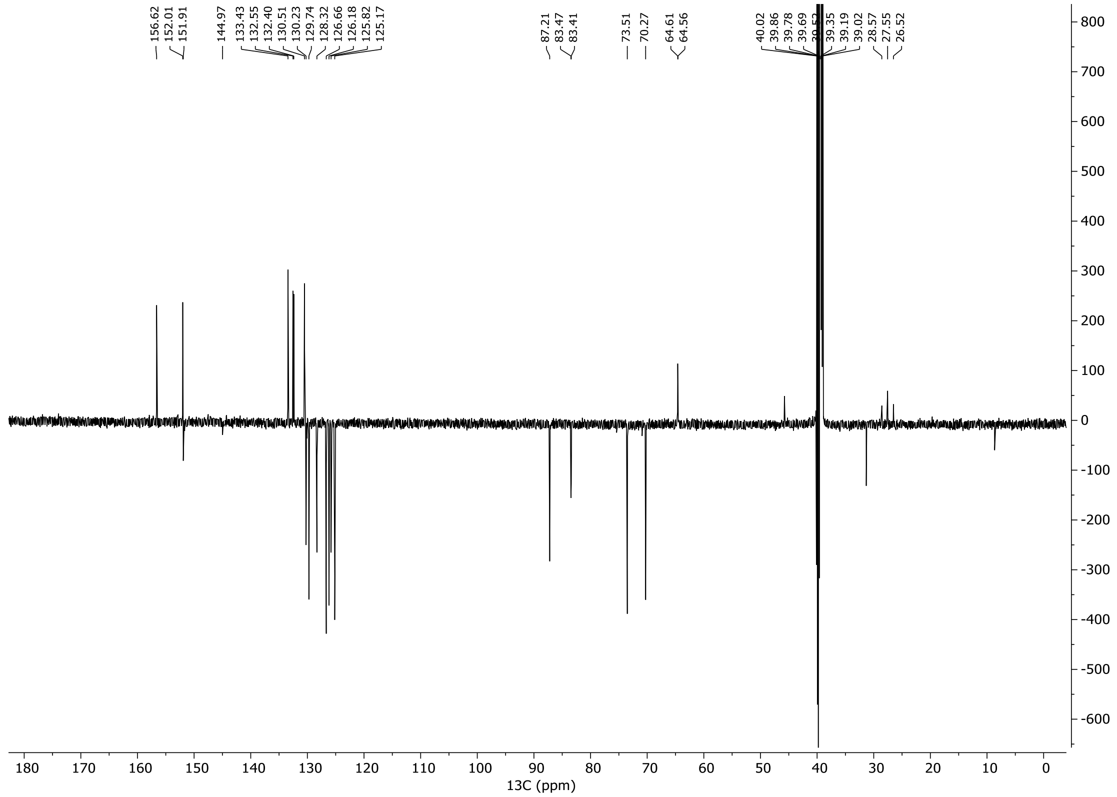
156.62
152.01
151.91
144.97
133.43
132.55
132.40
130.51
130.23
129.74
128.32
126.66
126.18
125.82
125.17
87.21
83.47
83.41
73.51
70.27
64.61
64.56
40.02
39.86
39.78
39.69
39.35
39.19
39.02
28.57
27.55
26.52
13C (ppm)

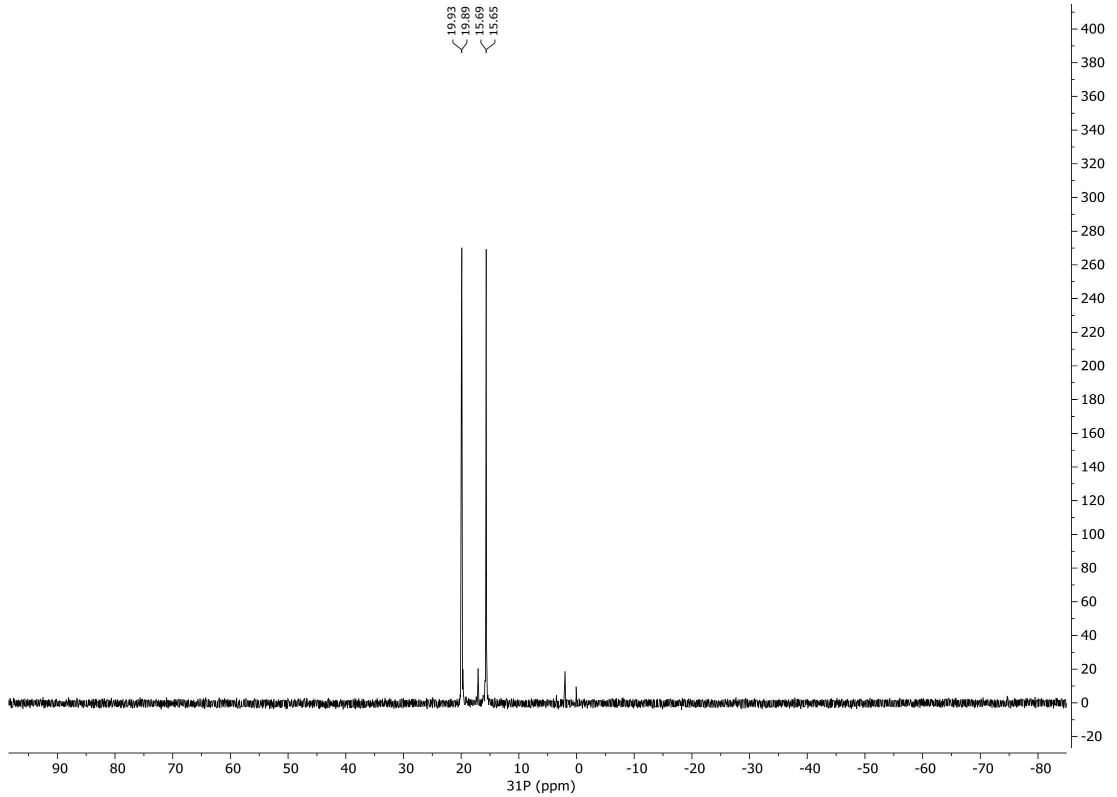
19.93
19.89
15.69
15.65
31P (ppm)

NMR spectra of compound **31A.7**

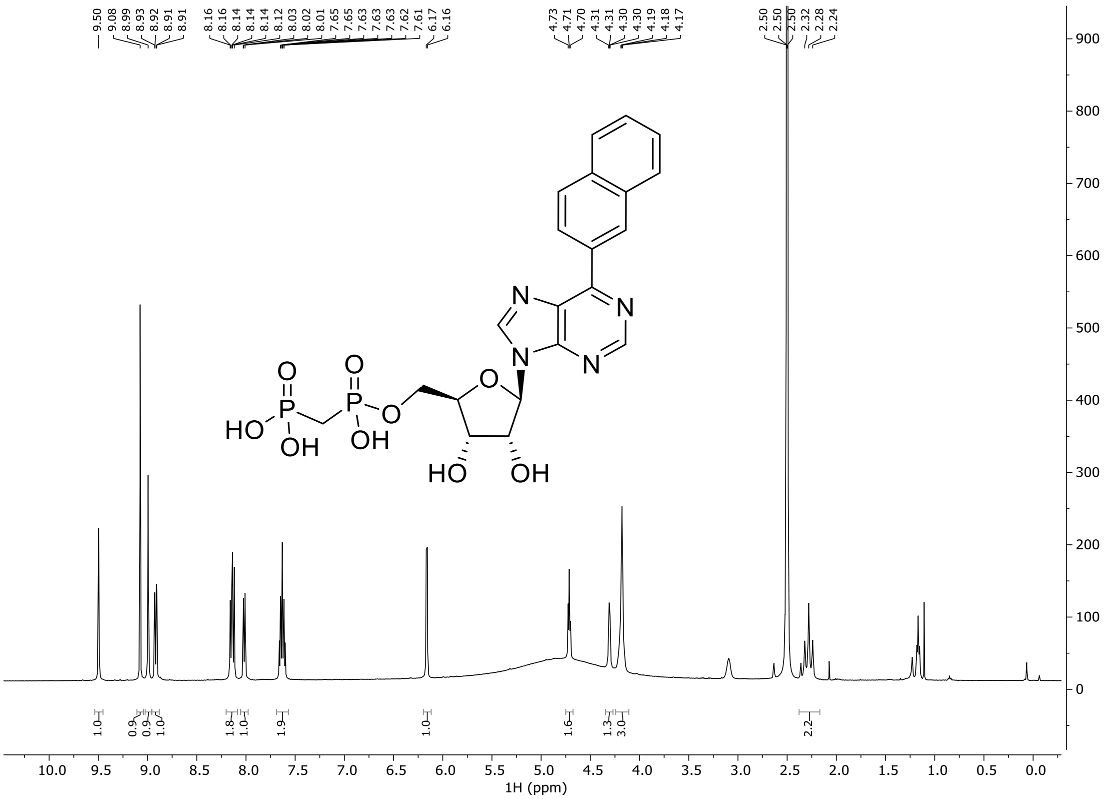

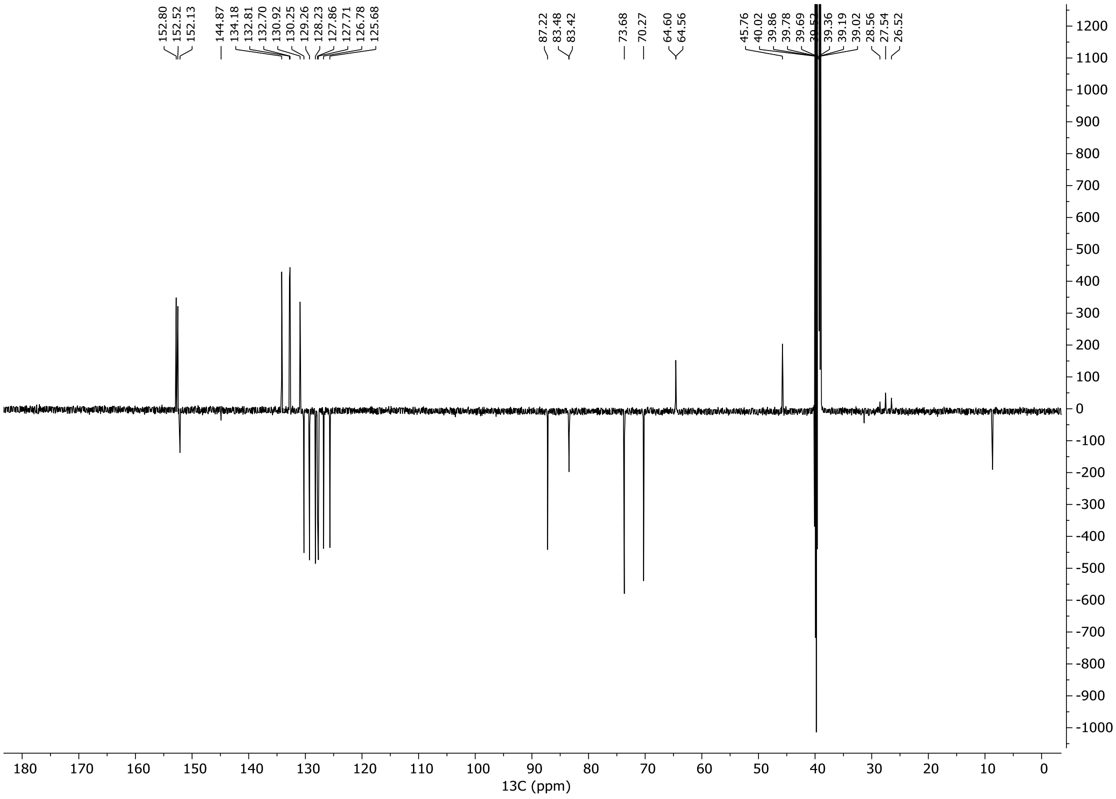

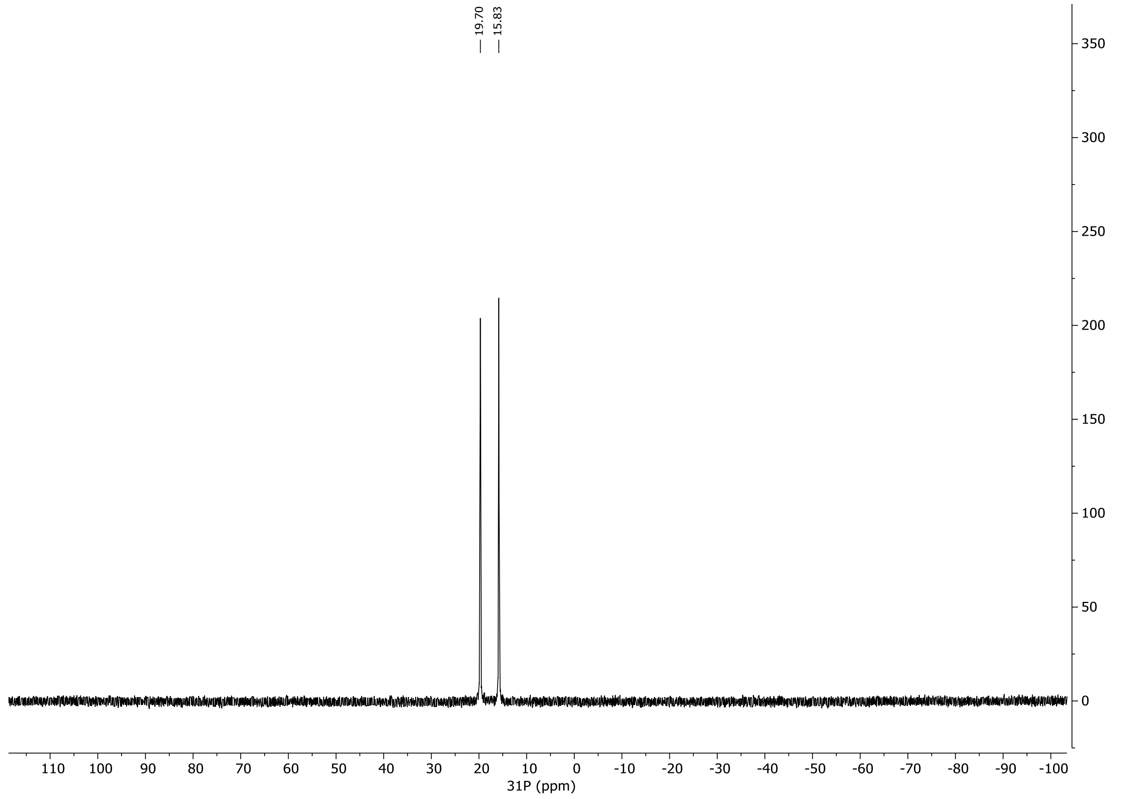

NMR spectra of compound **31A.12**

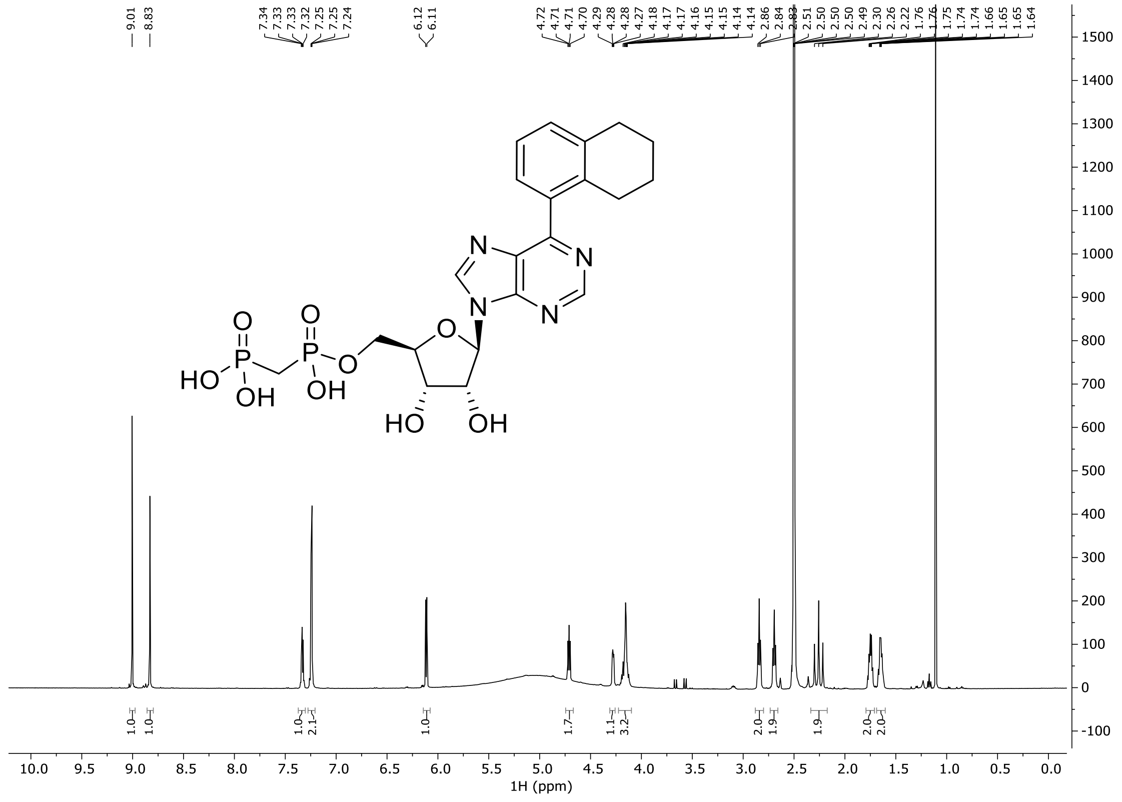

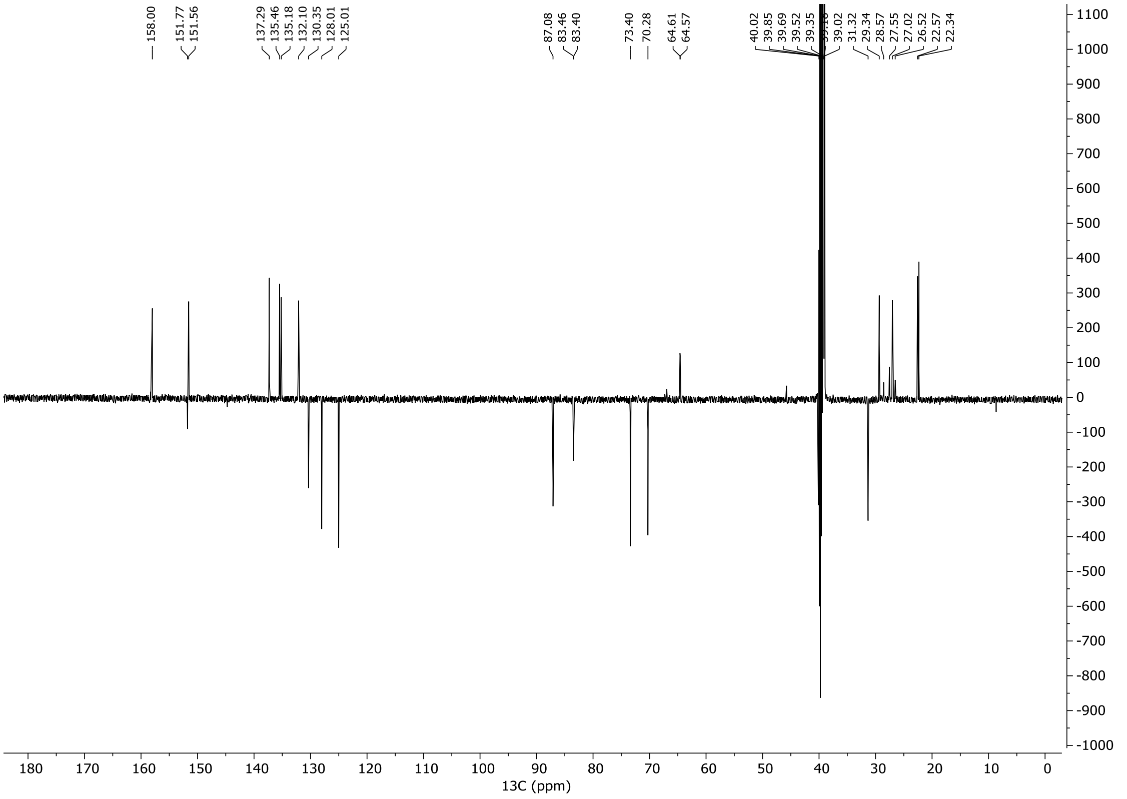
158.00
151.77
151.56
137.29
135.46
135.18
132.10
130.35
128.01
125.01
87.08
83.46
83.40
73.40
70.28
64.61
64.57
40.02
39.85
39.69
39.52
39.35
39.02
31.32
29.34
28.57
27.55
27.02
26.52
22.57
22.34
13C (ppm)

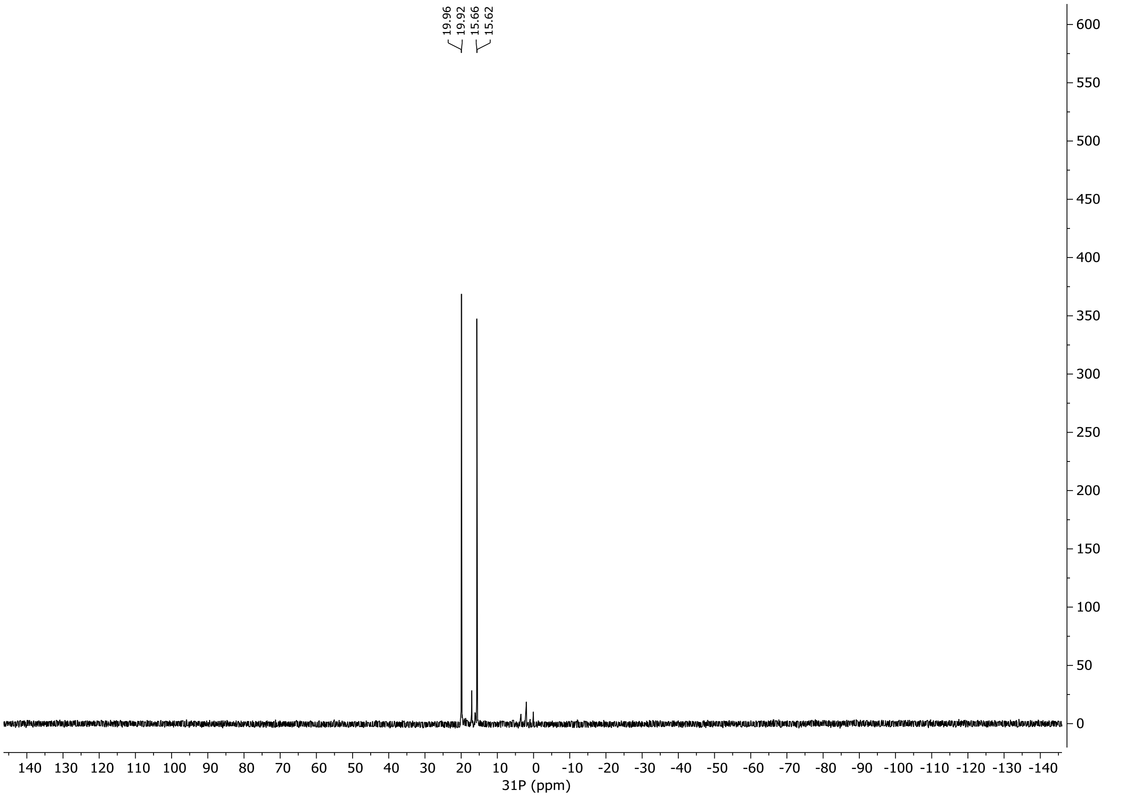
19.96
19.92
15.66
15.62
31P (ppm)

NMR spectra of compound **33A.5**

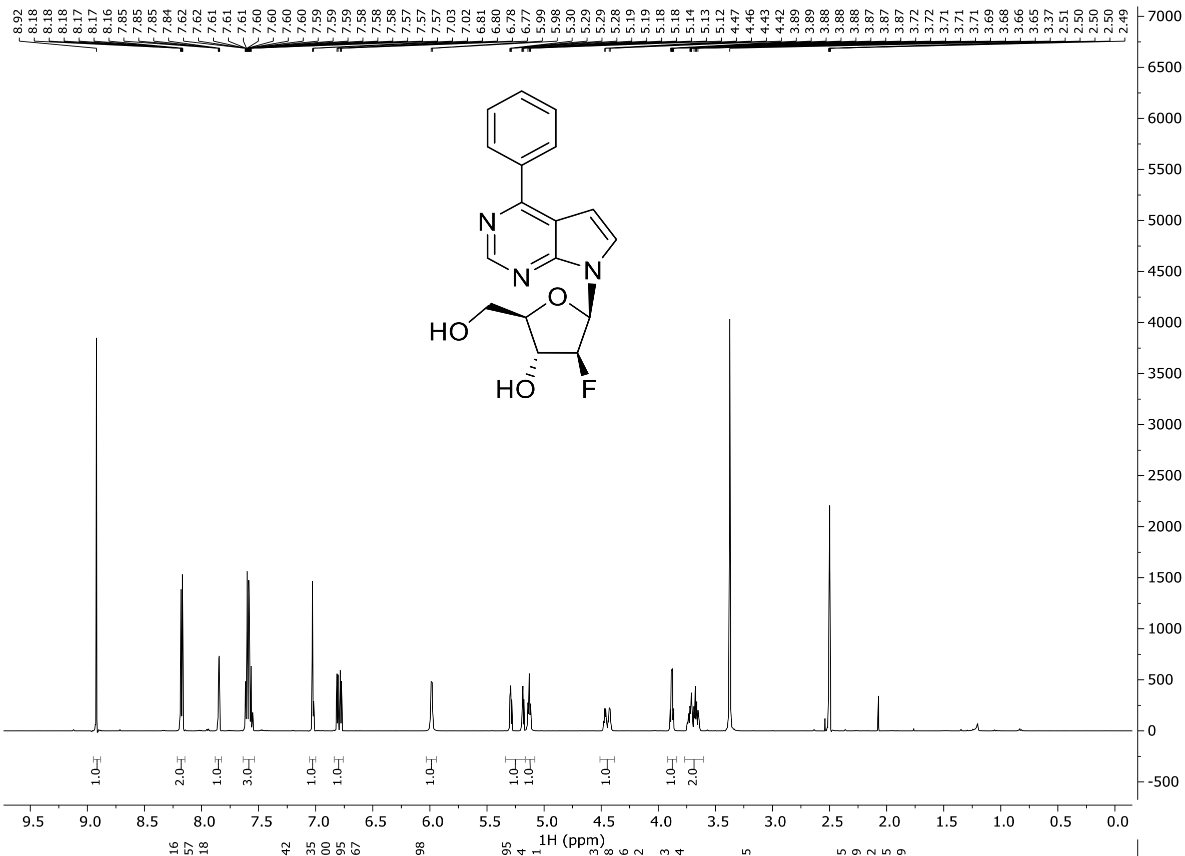

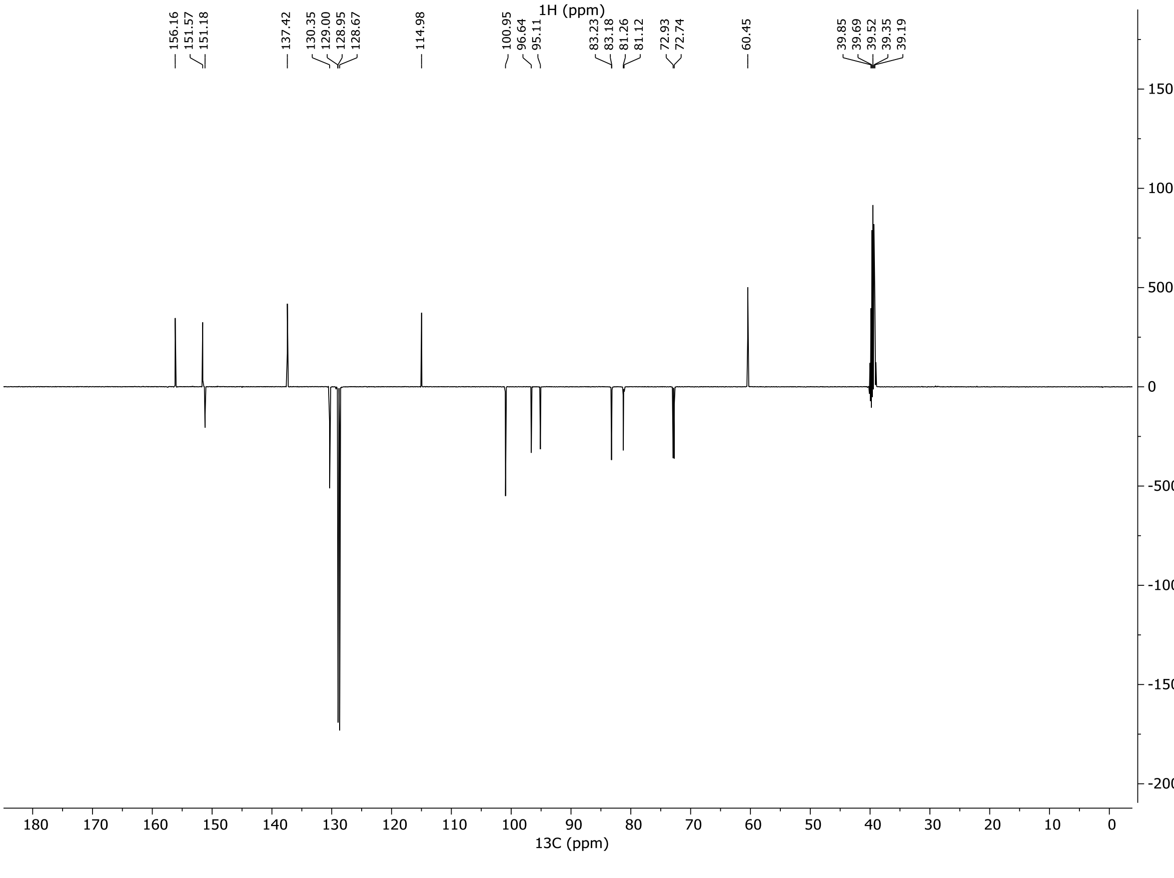

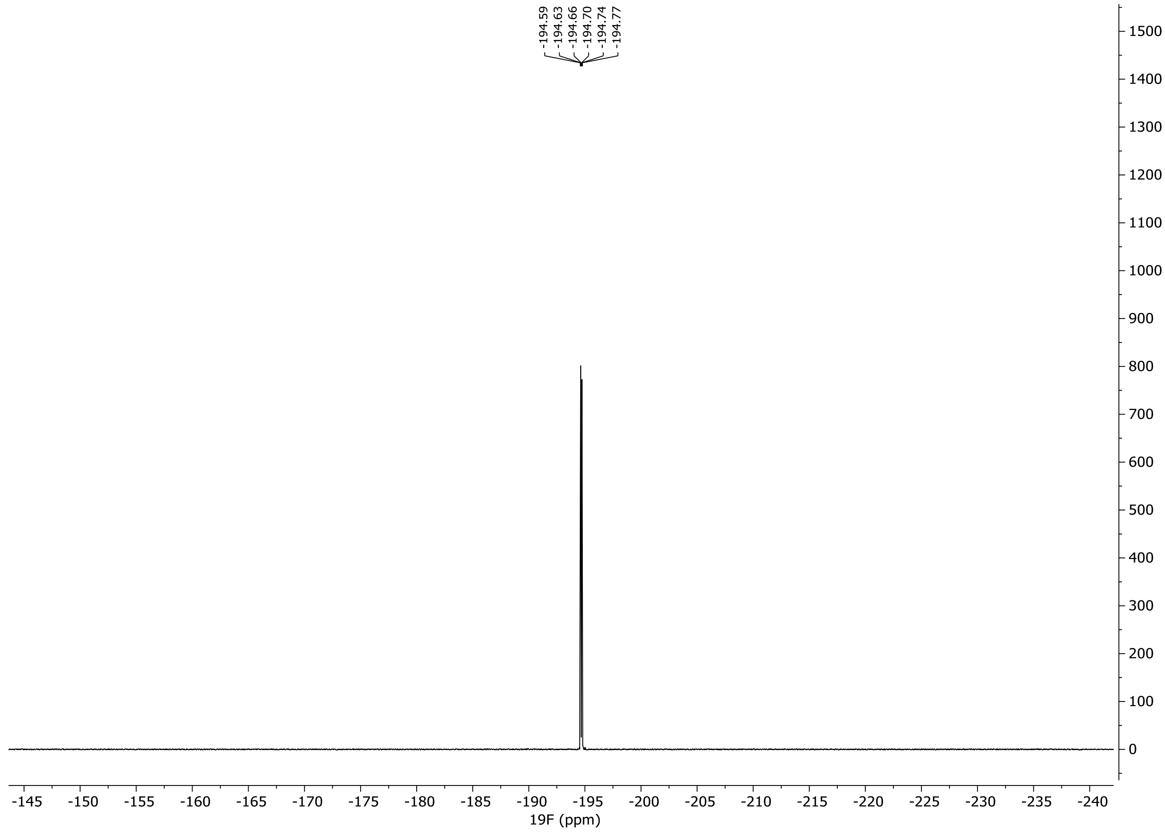

NMR spectra of compound **33A.6**

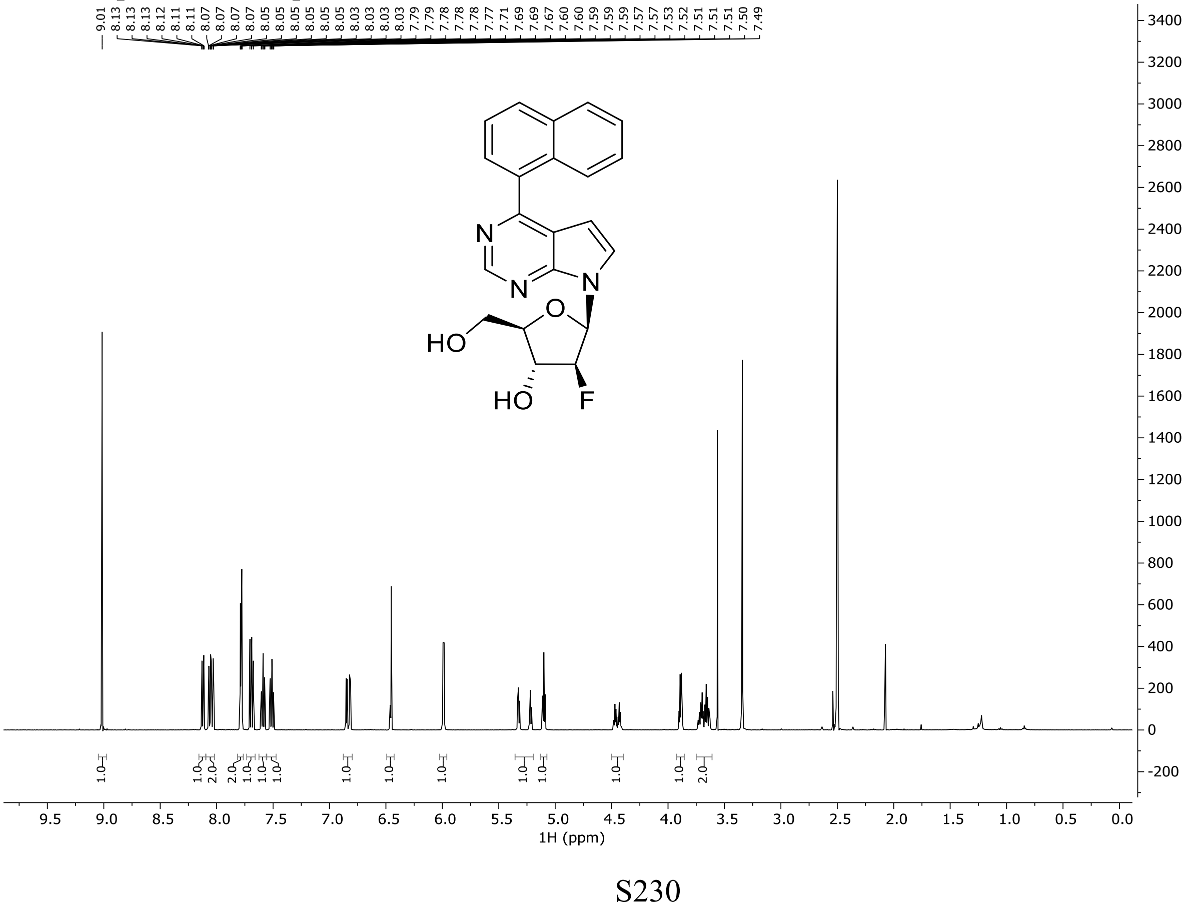

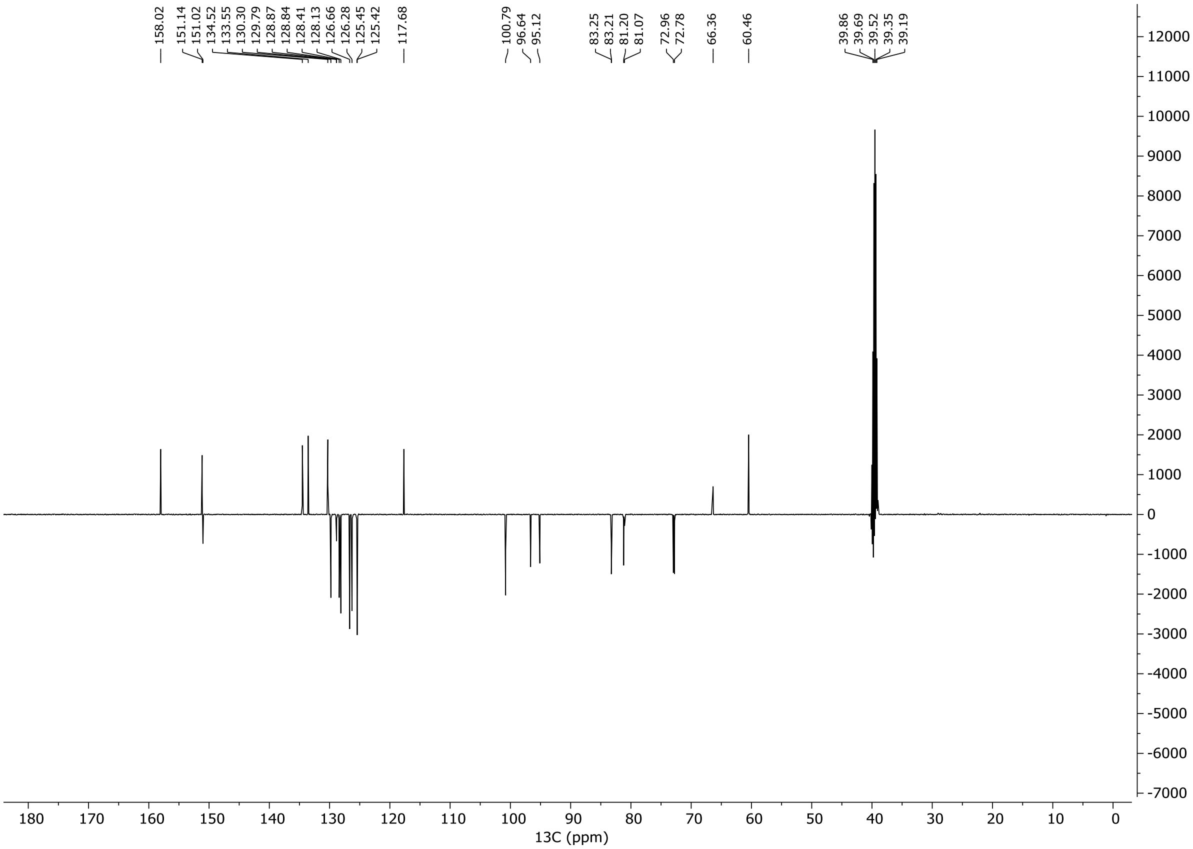

158.02
151.14
151.02
134.52
133.55
130.30
129.79
128.87
128.84
128.41
128.13
126.66
126.28
125.45
125.42
117.68
100.79
96.64
95.12
83.25
83.21
81.20
81.07
72.96
72.78
66.36
60.46
39.86
39.69
39.52
39.35
39.19
13C (ppm)

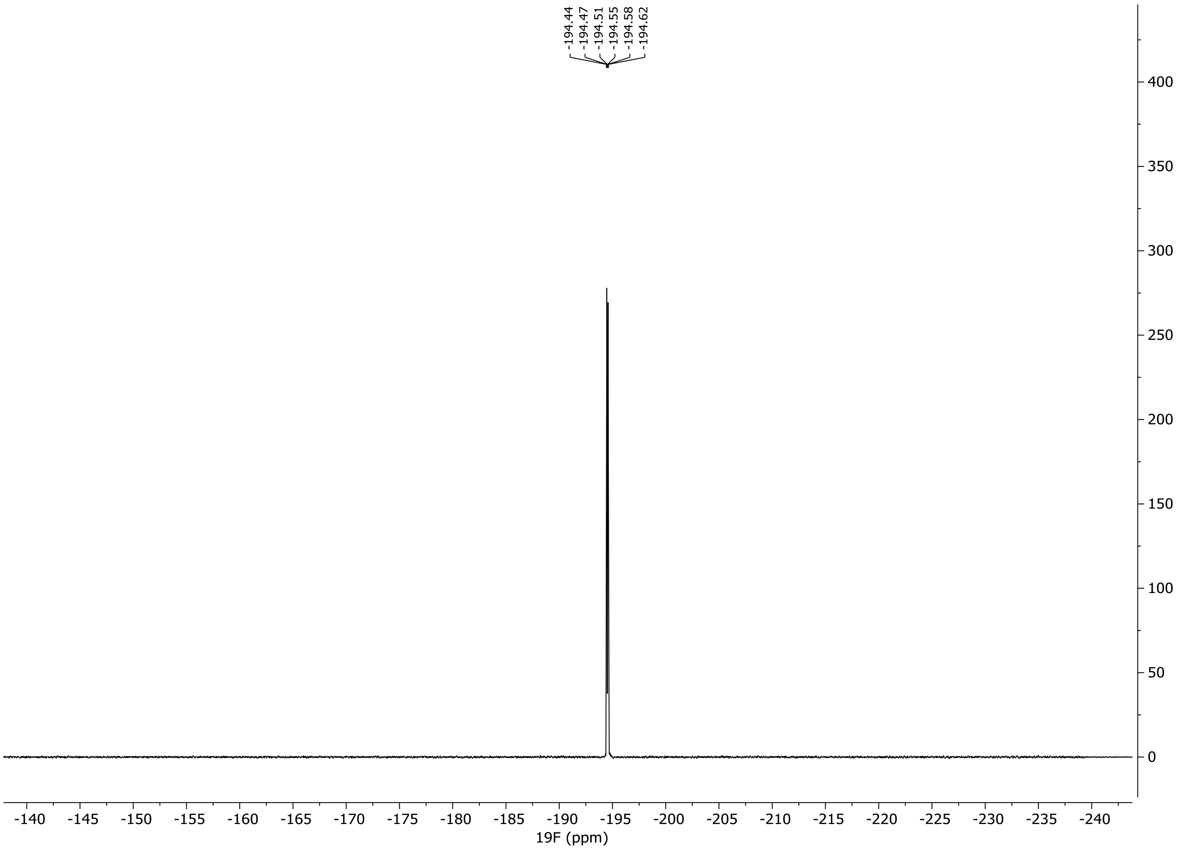

-194.44
-194.47
-194.51
-194.55
-194.58
-194.62
19F (ppm)

NMR spectra of compound **33A.7**

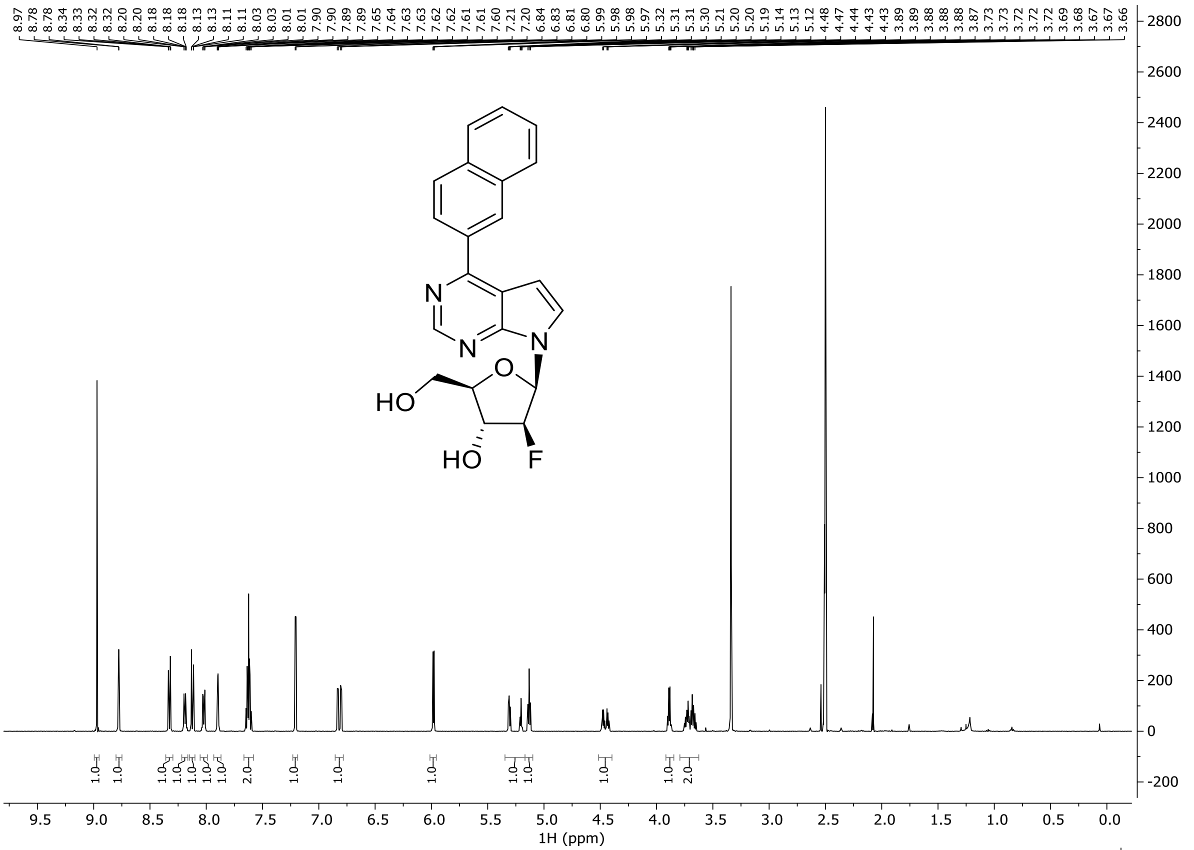

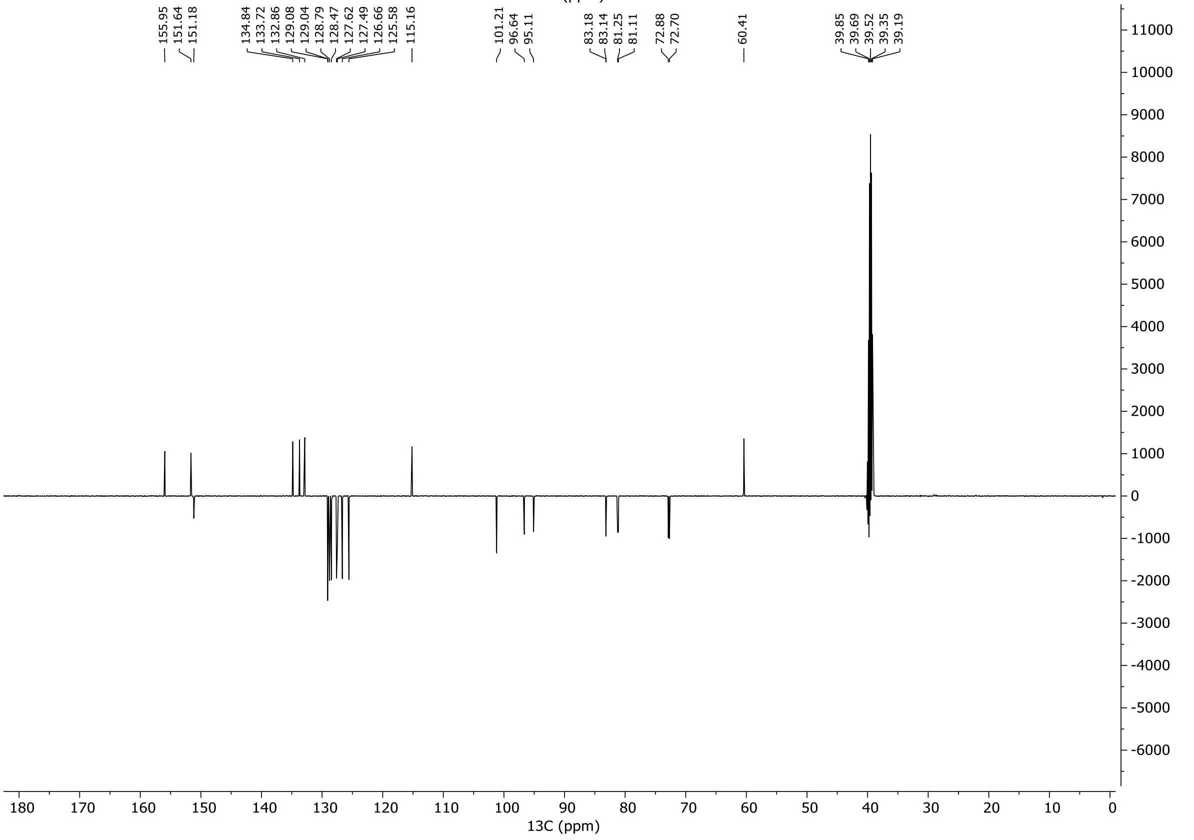

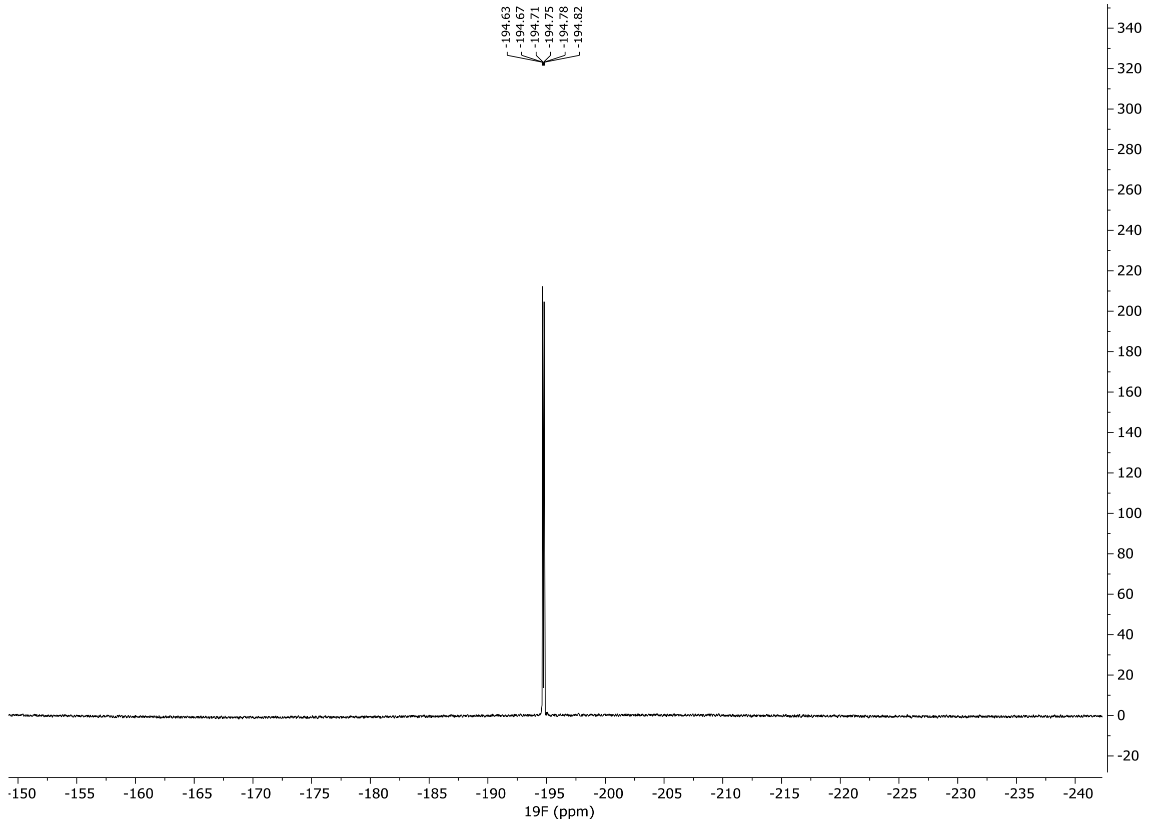

NMR spectra of compound **33A.12**

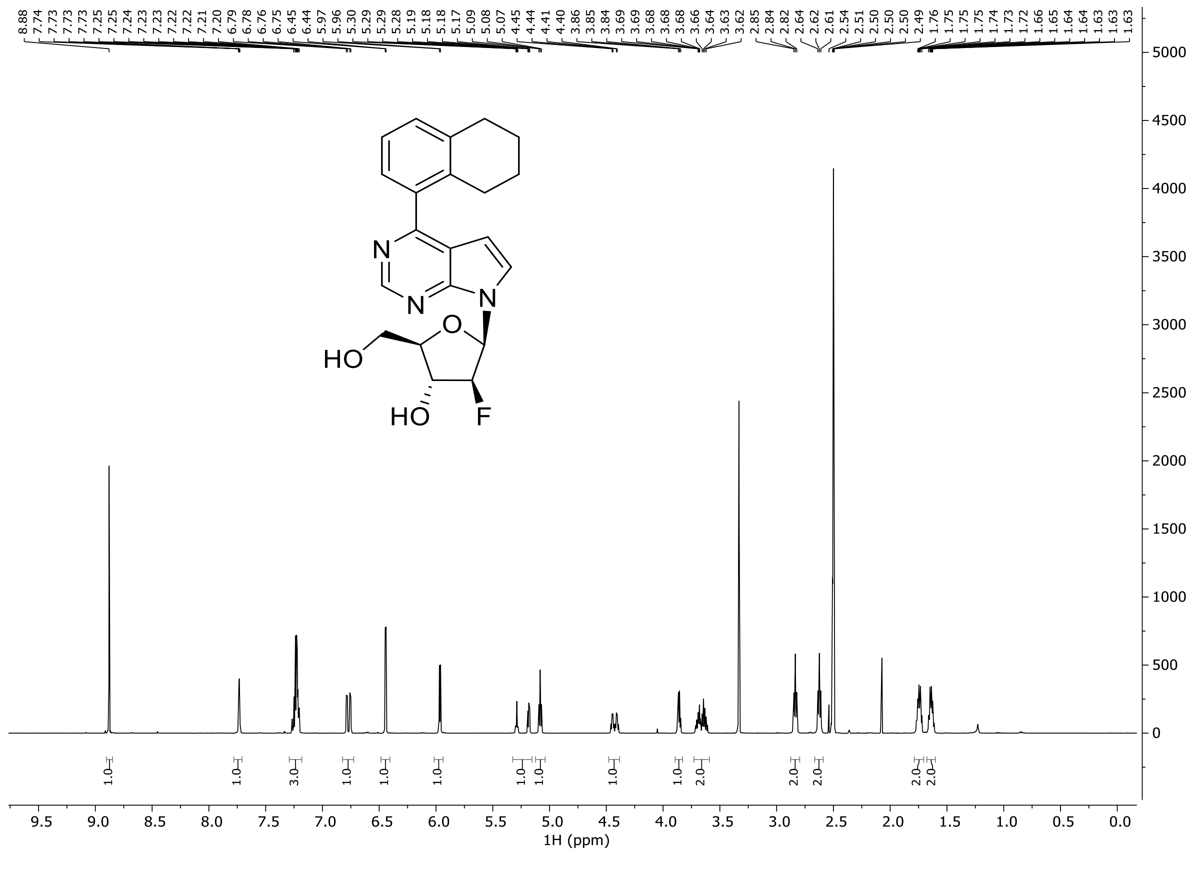

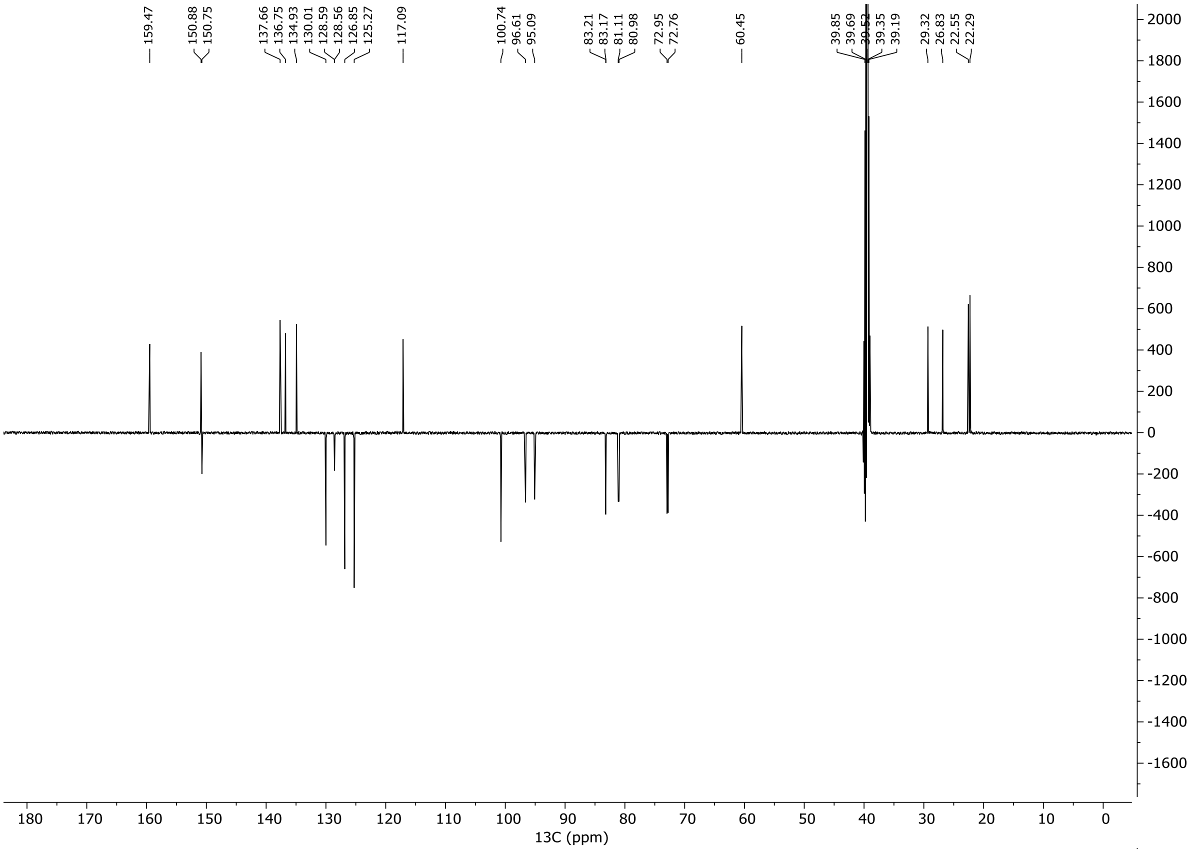
159.47
150.88
150.75
137.66
136.75
134.93
130.01
128.59
128.56
126.85
125.27
117.09
100.74
96.61
95.09
83.21
83.17
81.11
80.98
72.95
72.76
60.45
39.85
39.69
39.52
39.35
39.19
29.32
26.83
22.55
22.29
13C (ppm)

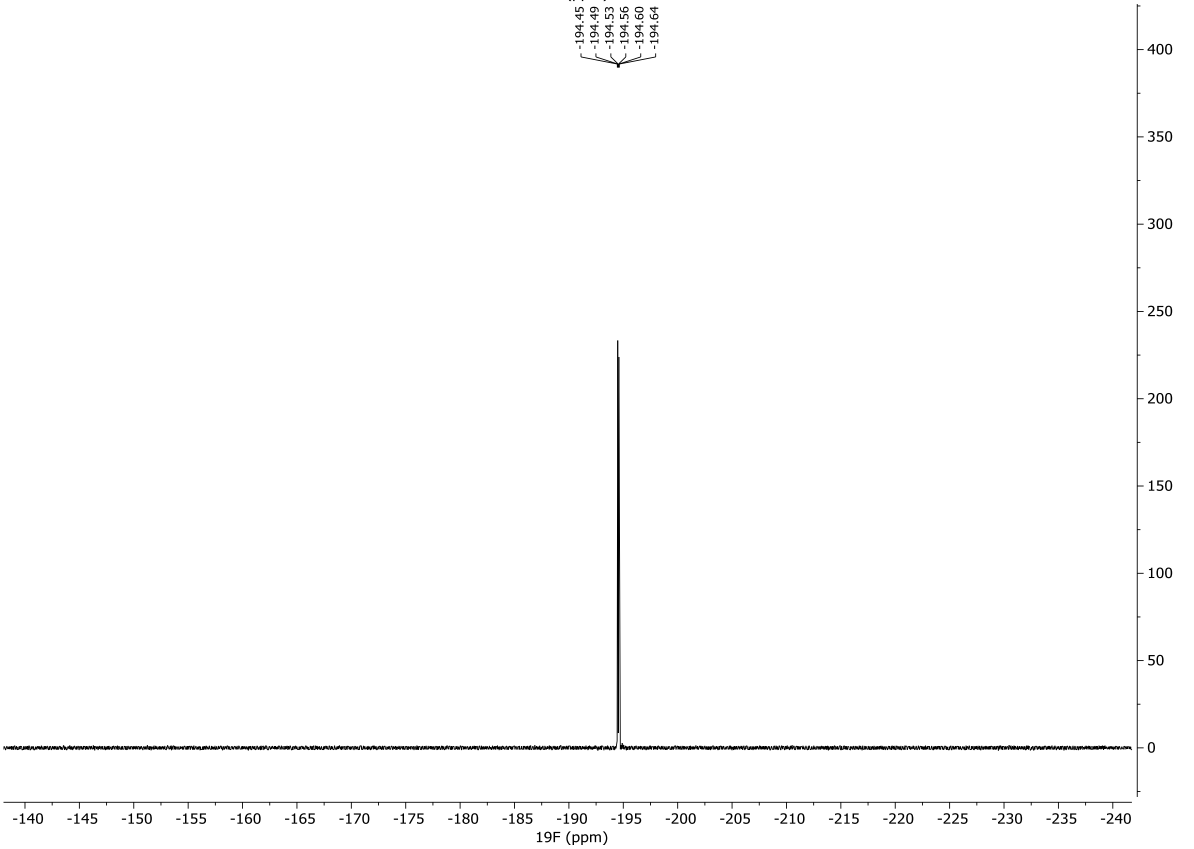
-194.45
-194.49
-194.53
-194.56
-194.60
-194.64
19F (ppm)

NMR spectra of compound **33A.17**

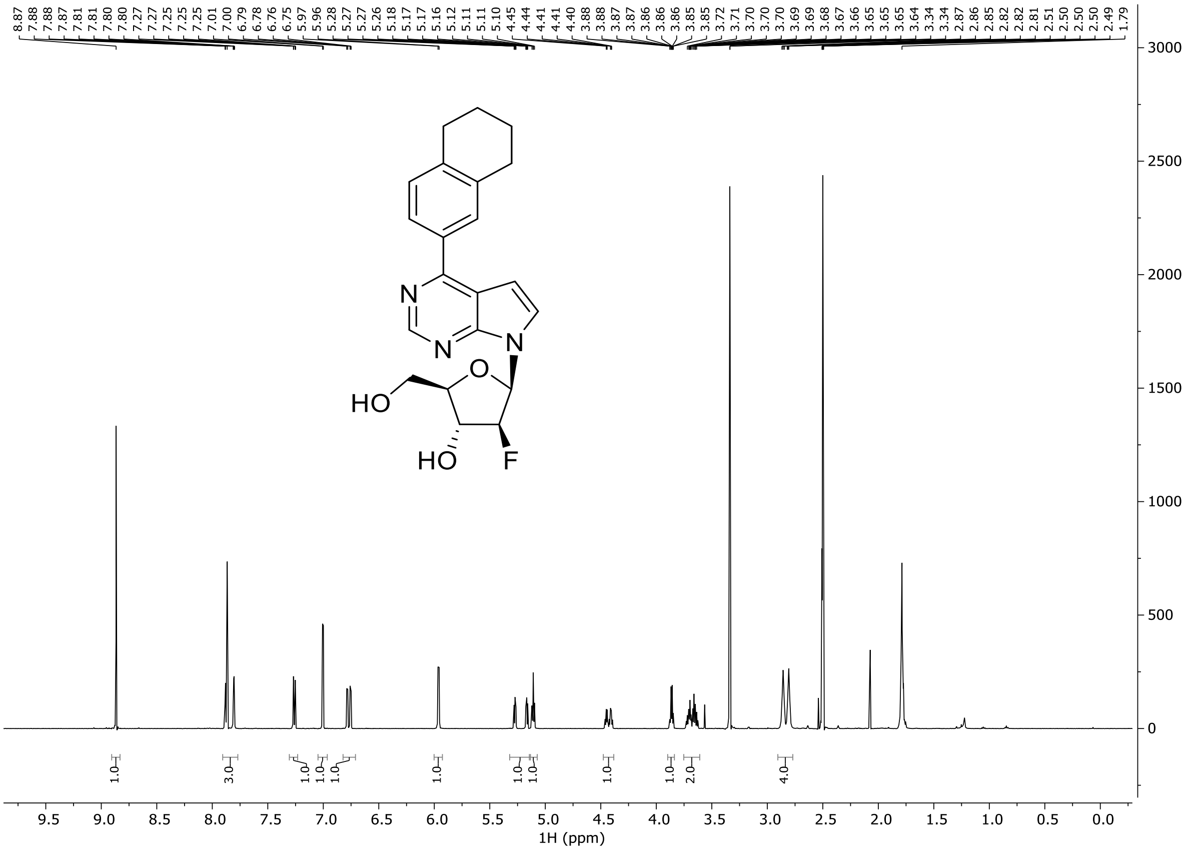

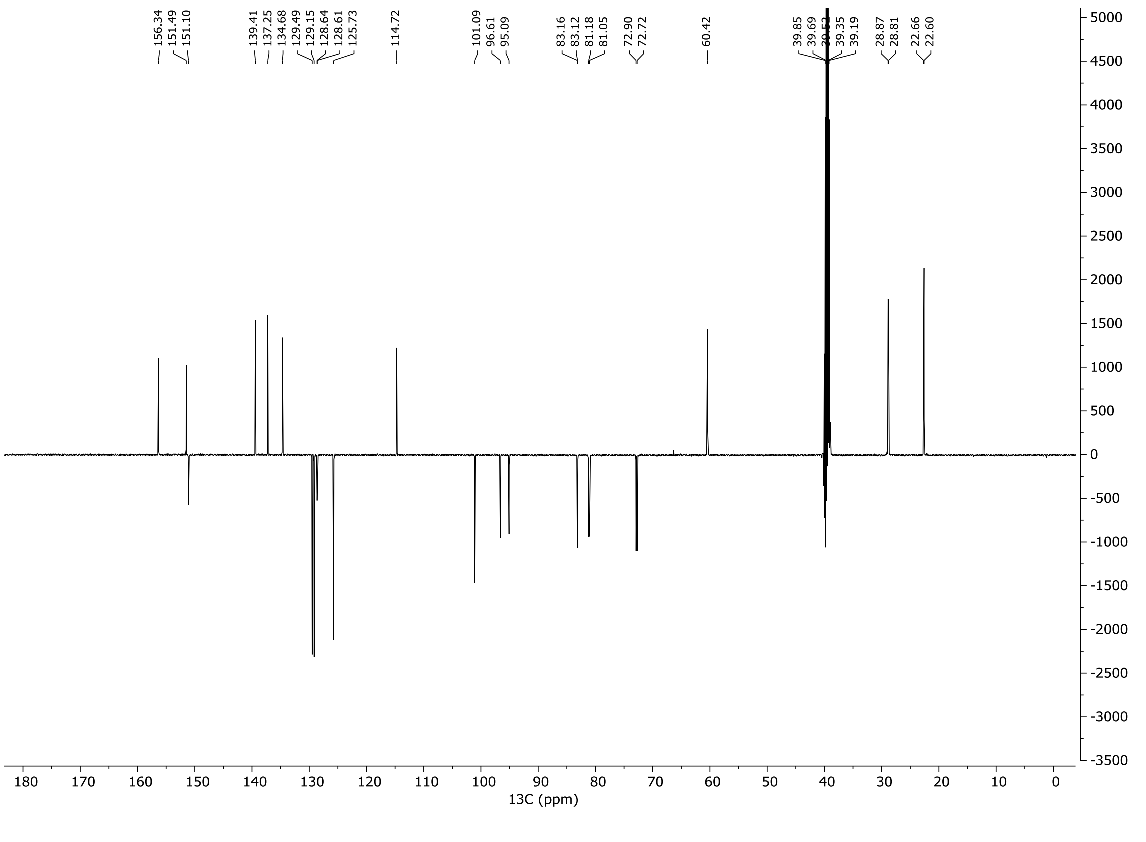

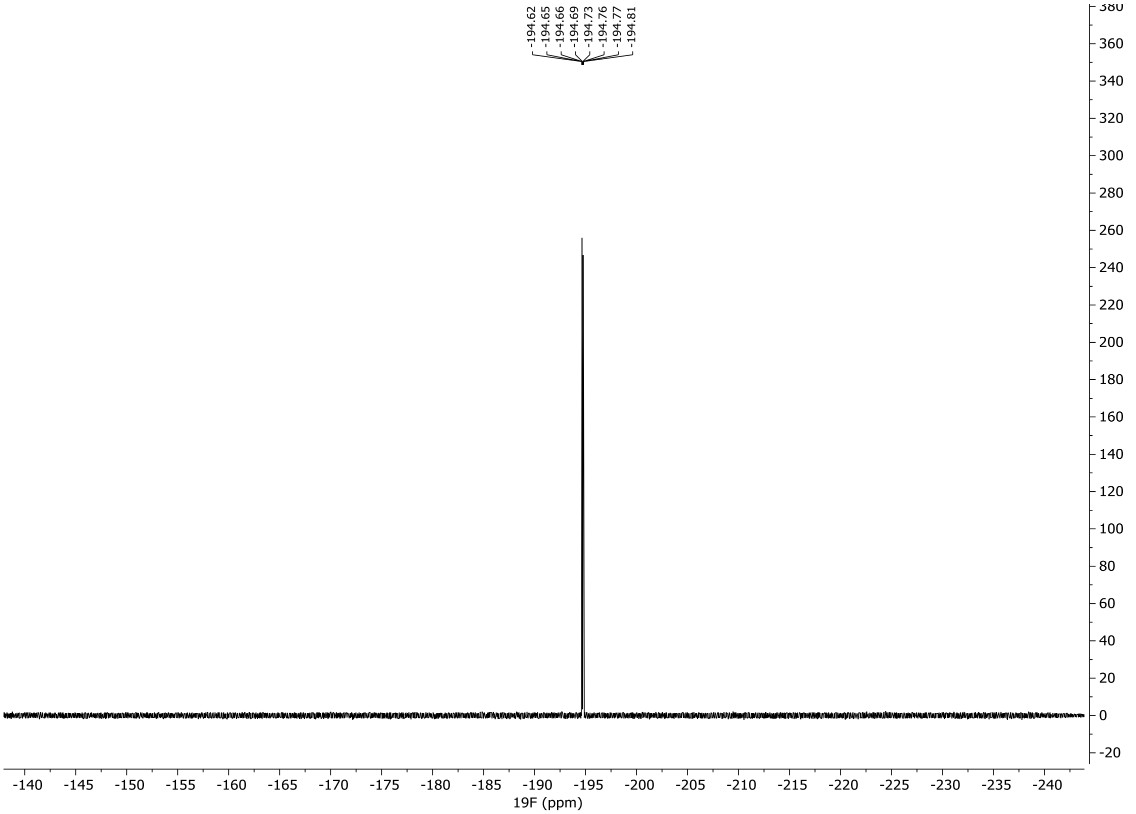

NMR spectra of compound **33A.23**

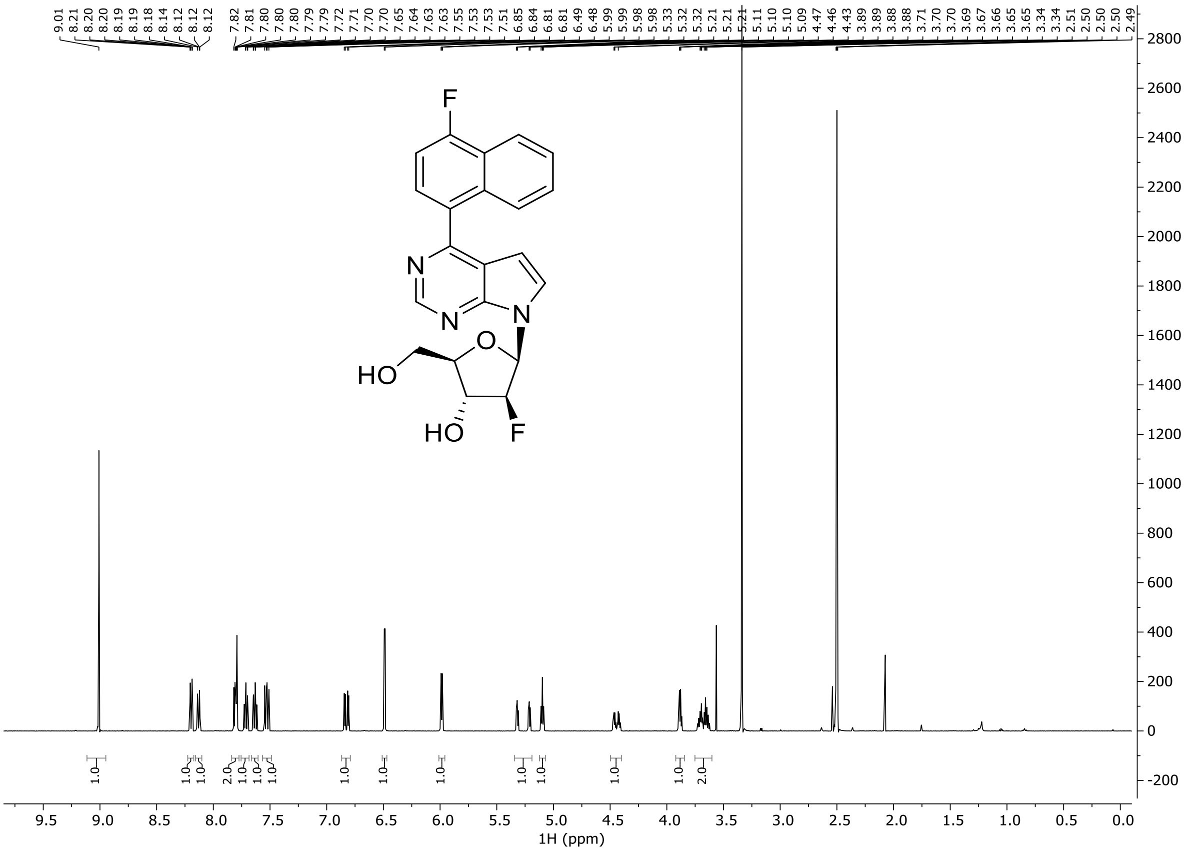

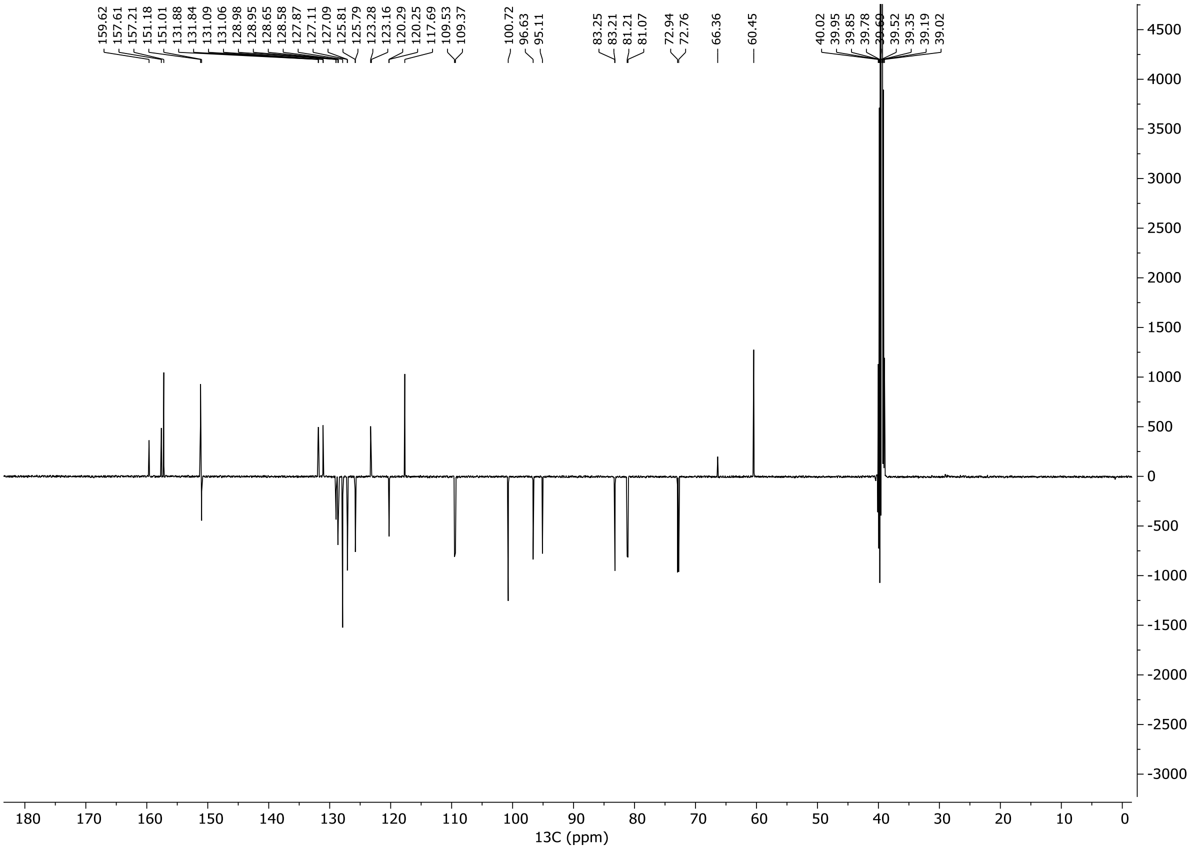

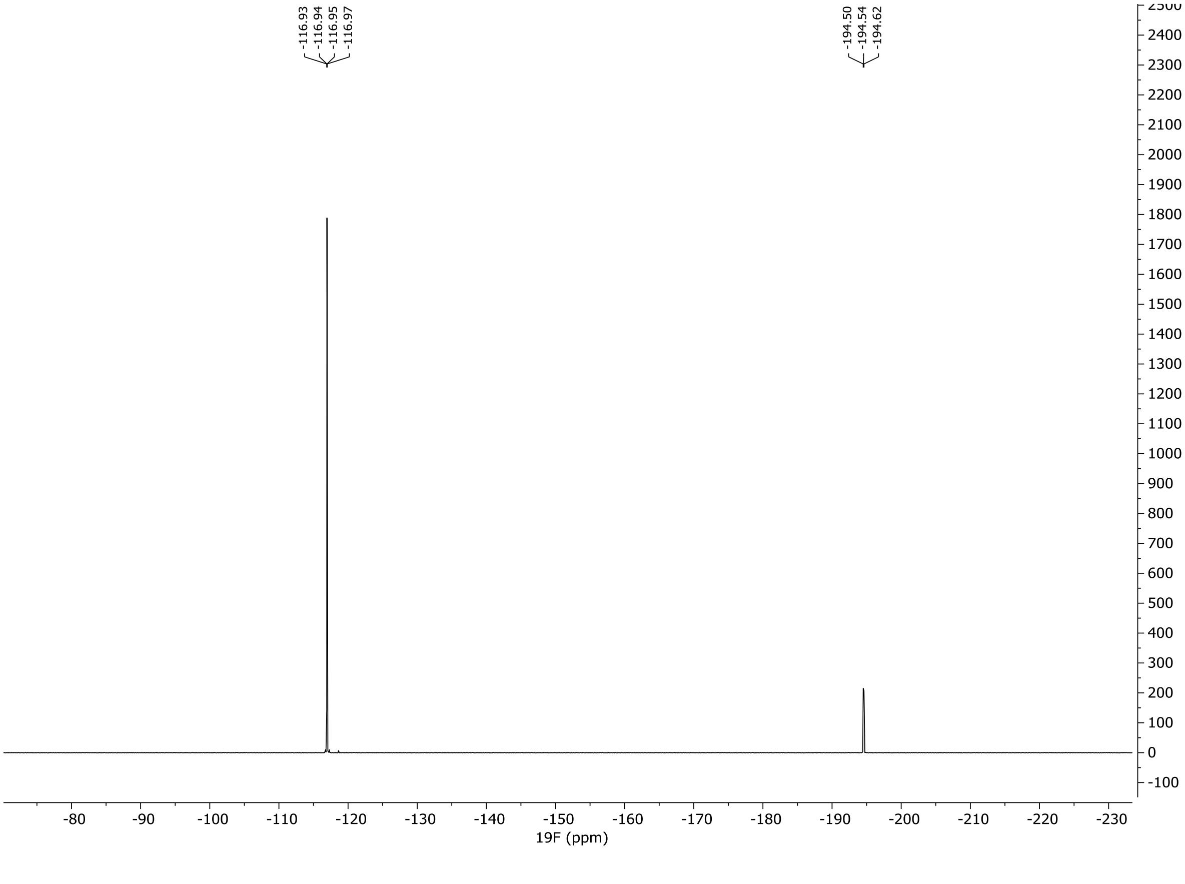

NMR spectra of compound **33A.28**

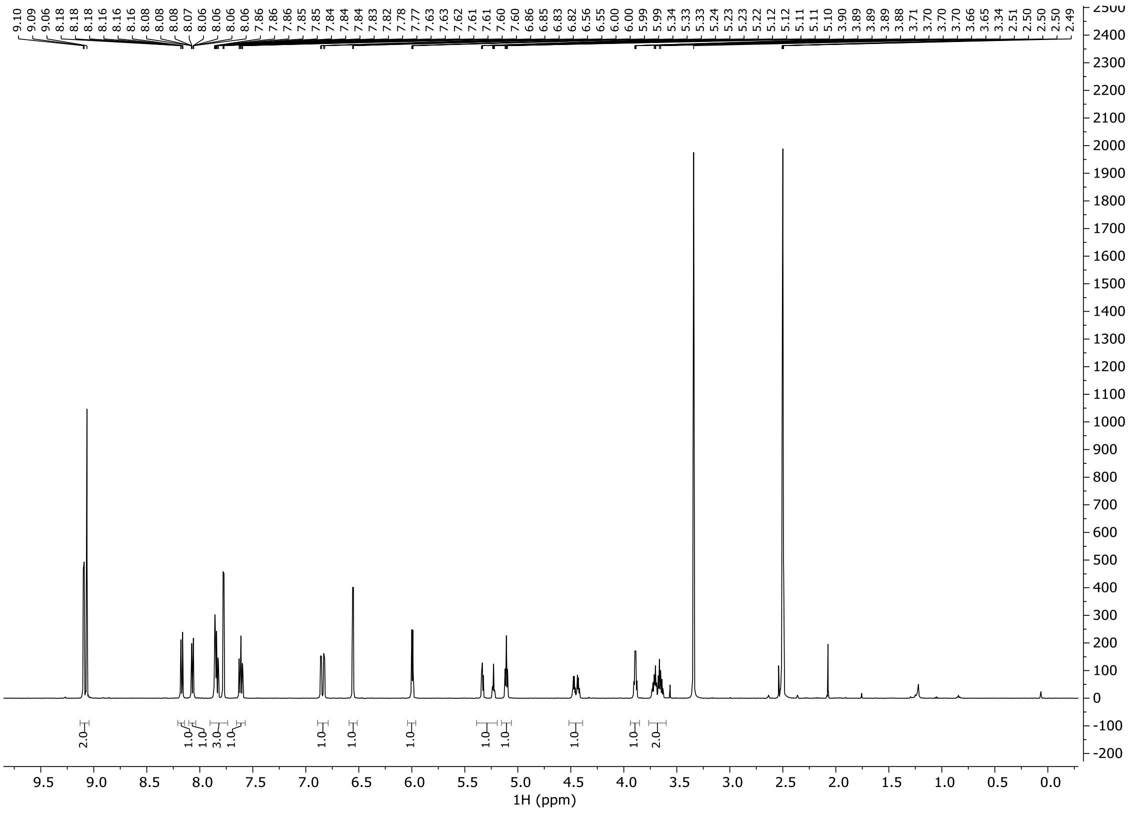

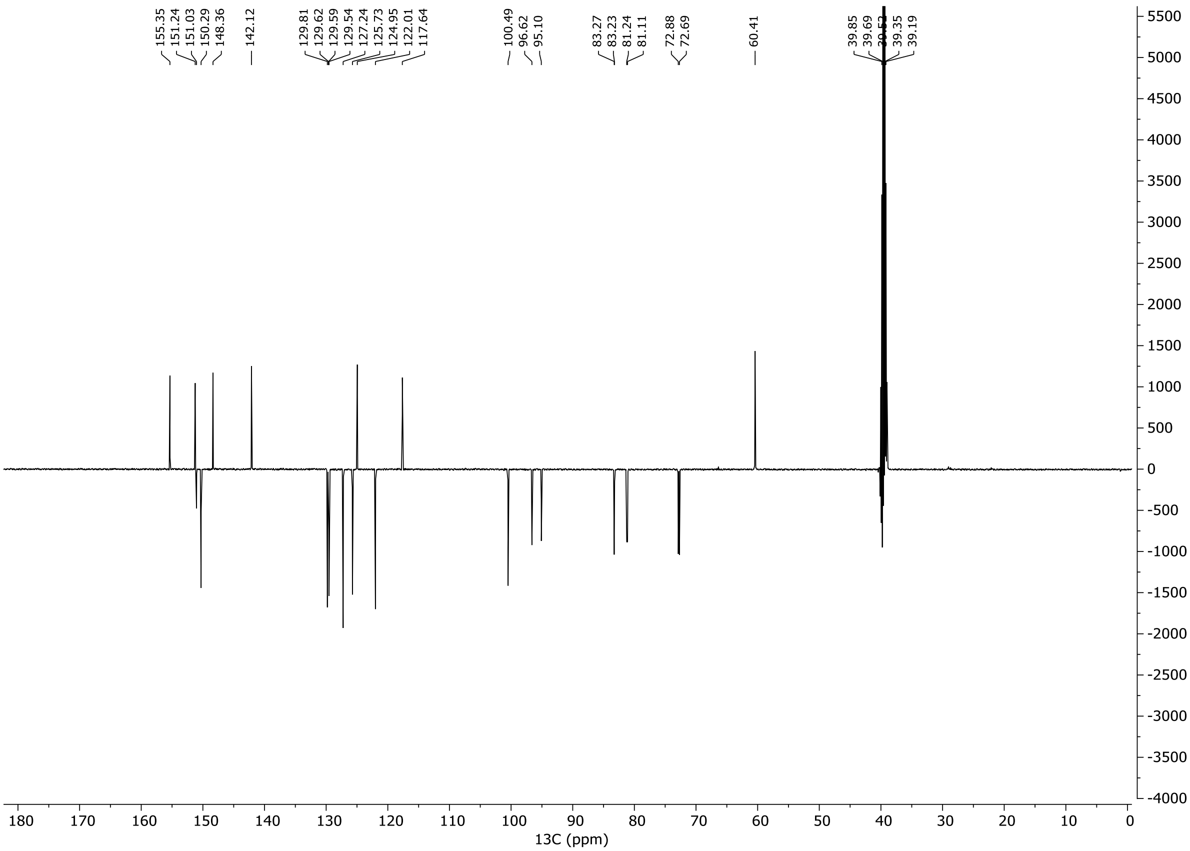

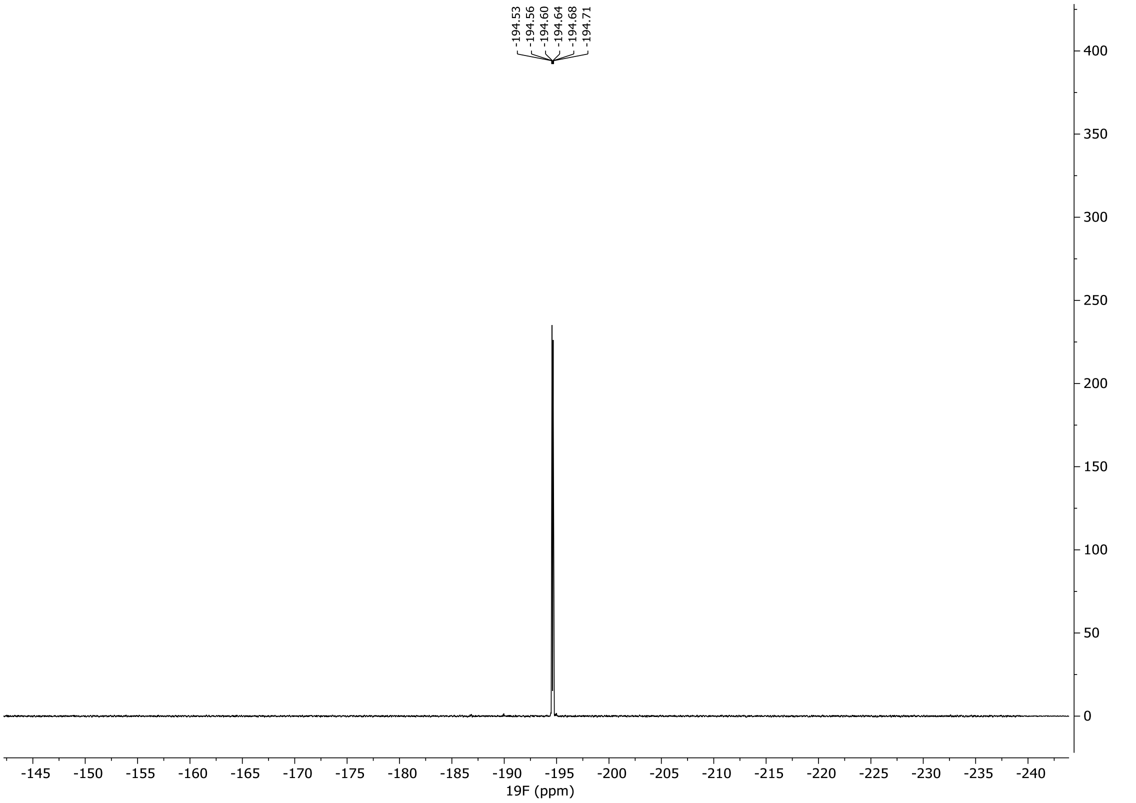

NMR spectra of compound **34A.5**

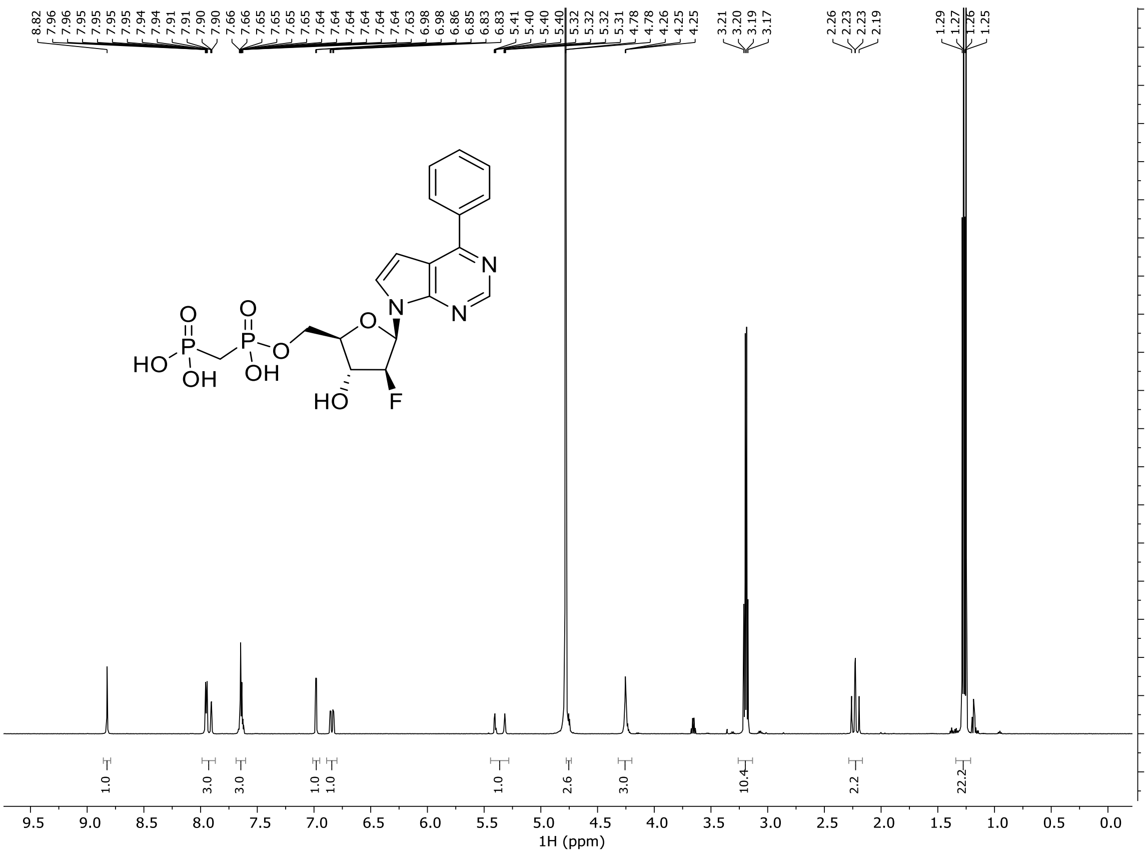

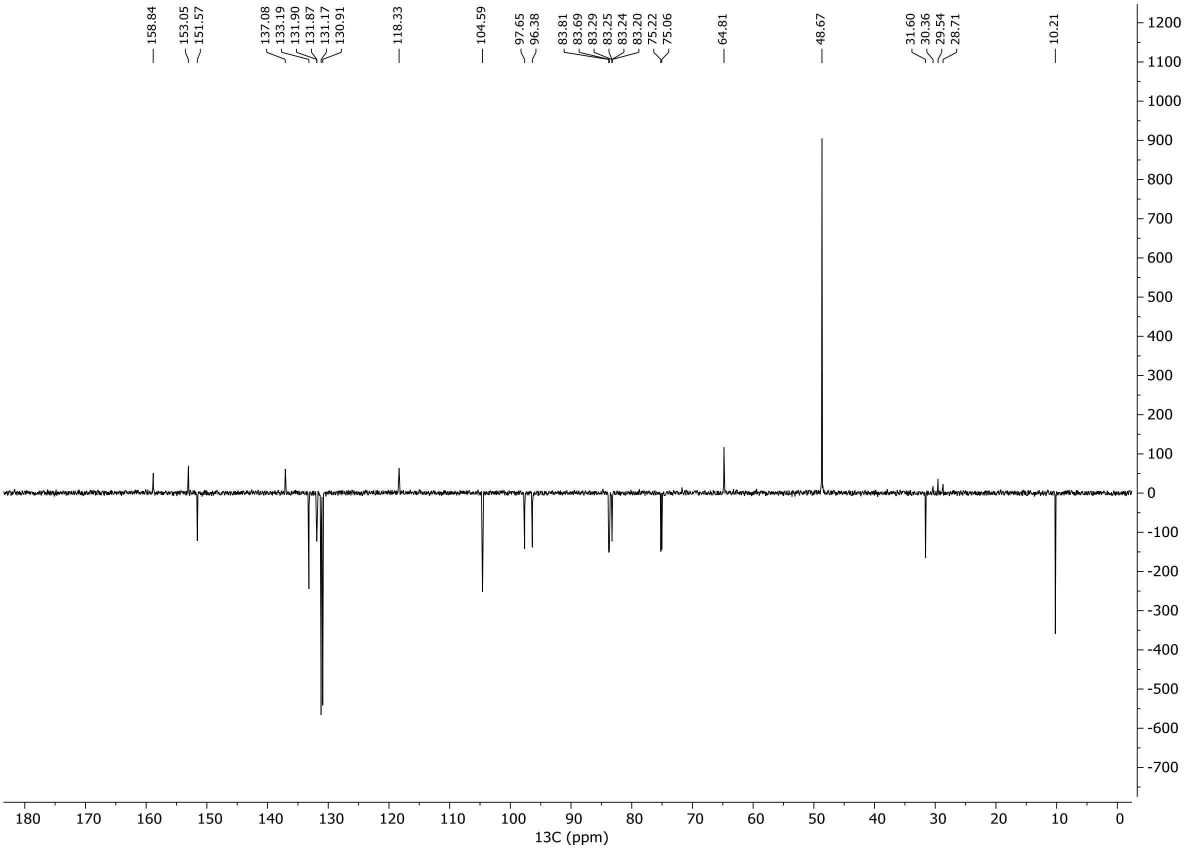
158.84
153.05
151.57
137.08
133.19
131.90
131.87
131.17
130.91
118.33
104.59
97.65
96.38
83.81
83.69
83.29
83.25
83.24
83.20
75.22
75.06
64.81
48.67
31.60
30.36
29.54
28.71
10.21
13C (ppm)

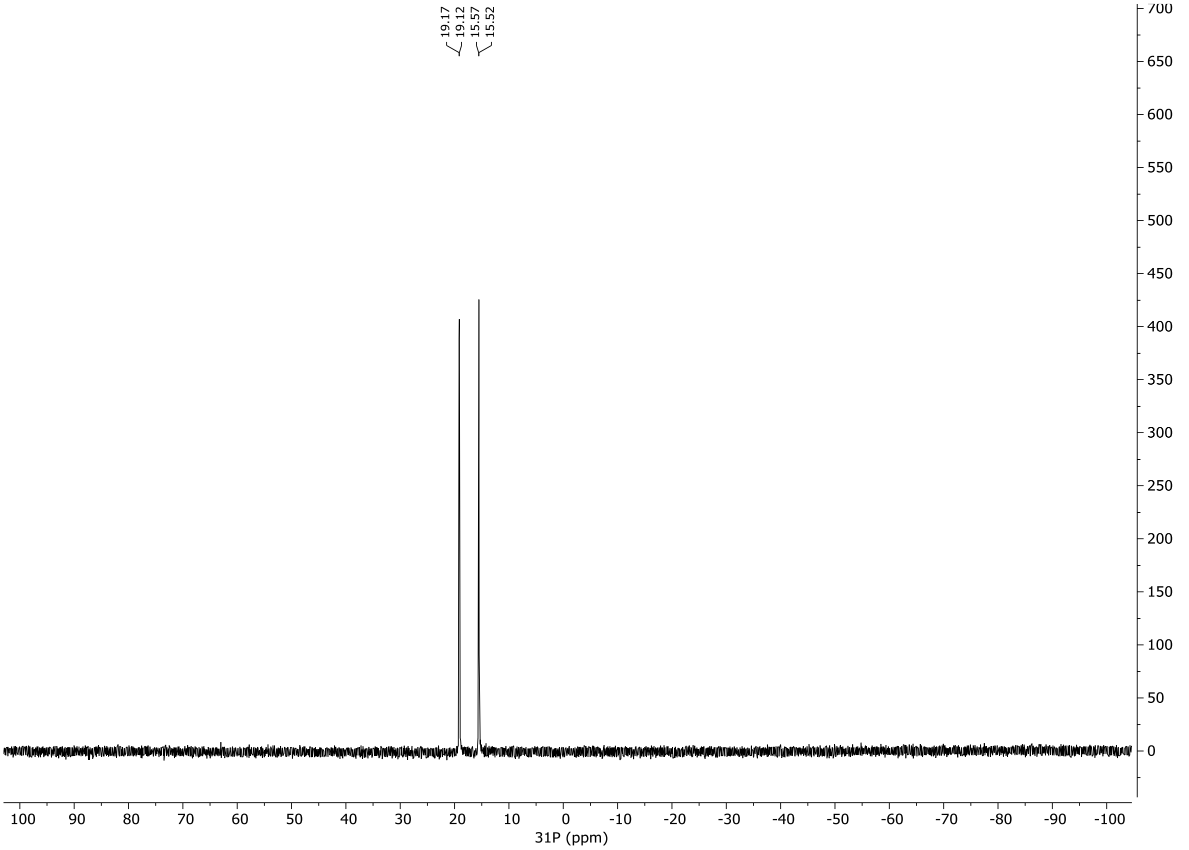
19.17
19.12
15.57
15.52
31P (ppm)

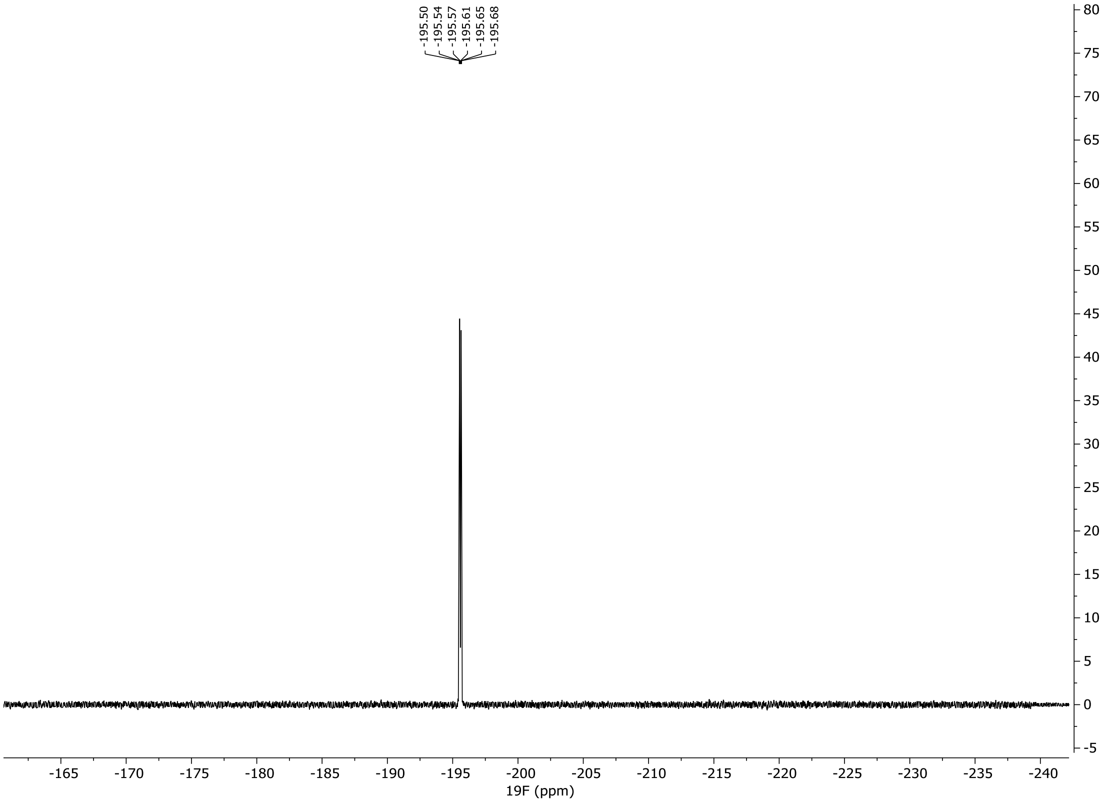

NMR spectra of compound **34A.6**

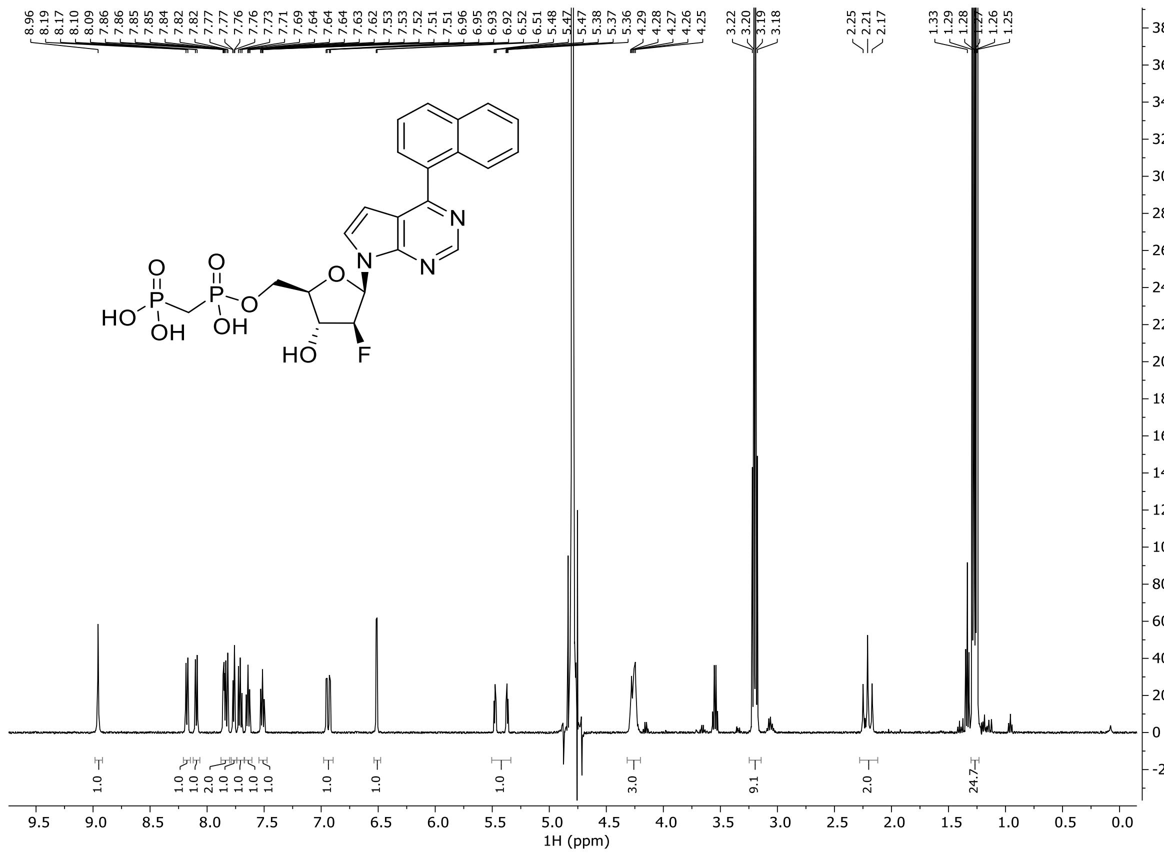

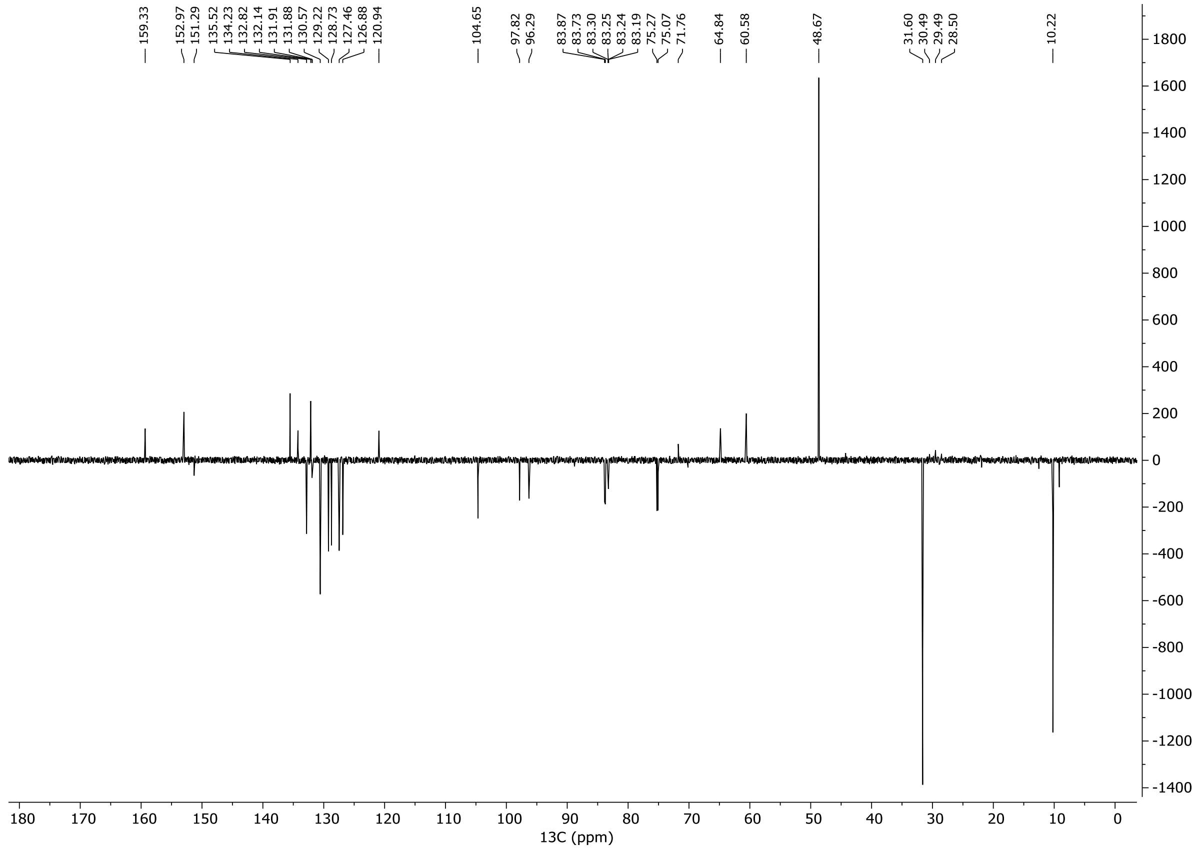
159.33
152.97
151.29
135.52
134.23
132.82
132.14
131.91
131.88
130.57
129.22
128.73
127.46
126.88
120.94
104.65
97.82
96.29
83.87
83.73
83.30
83.25
83.24
83.19
75.27
75.07
71.76
64.84
60.58
48.67
31.60
30.49
29.49
28.50
10.22
13C (ppm)

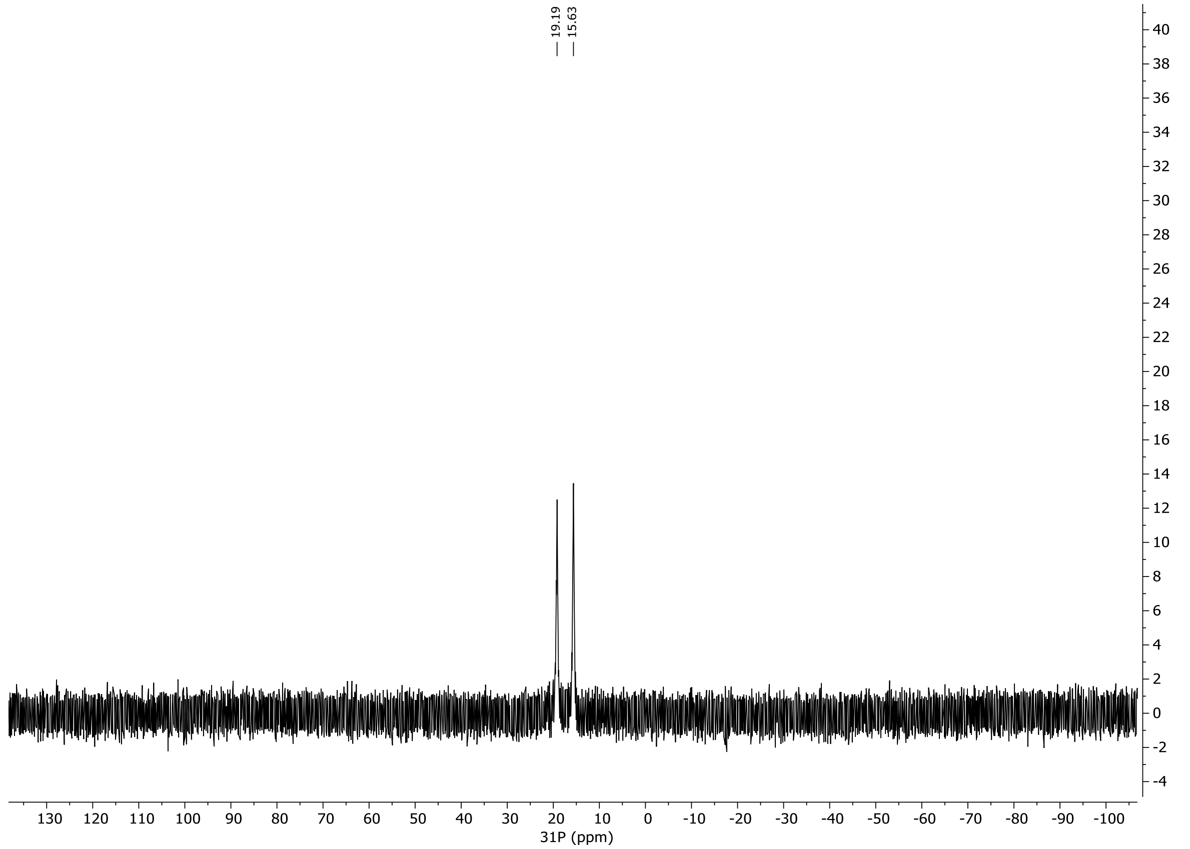
19.19
15.63
31P (ppm)

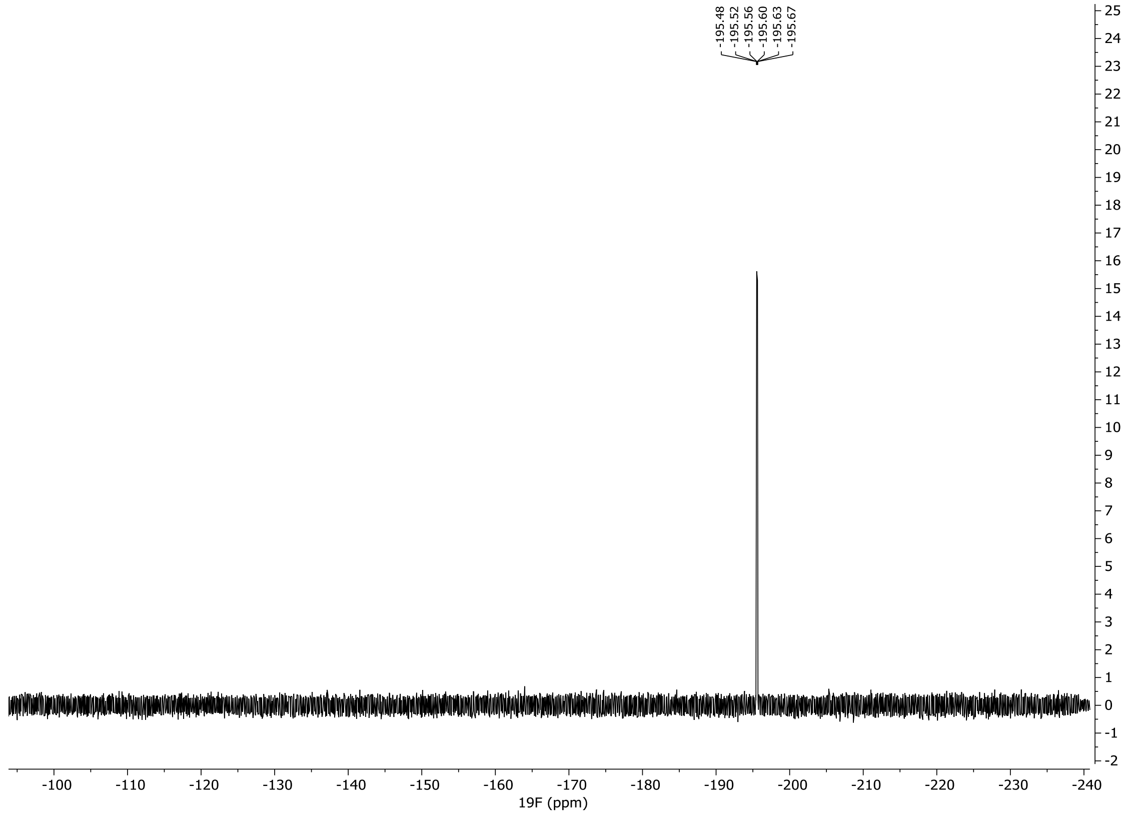

NMR spectra of compound **34A.7**

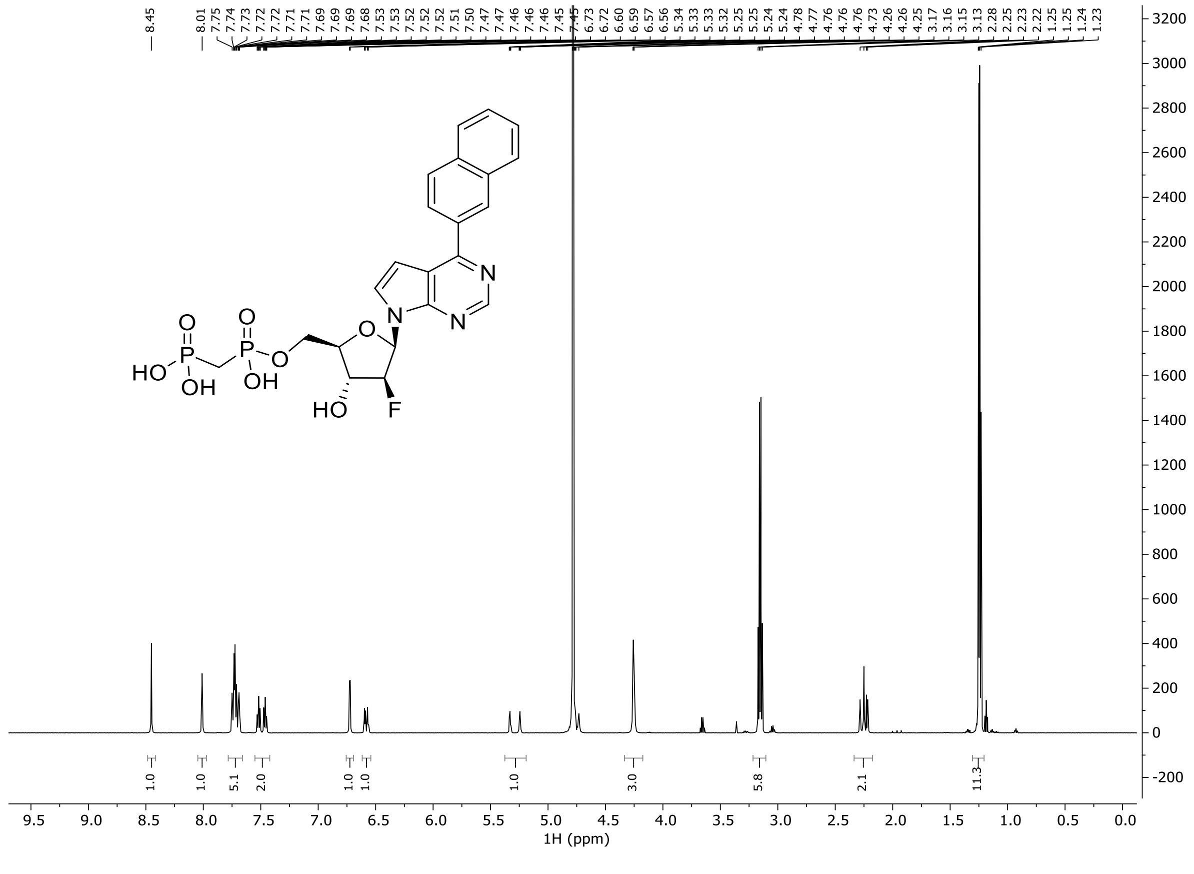

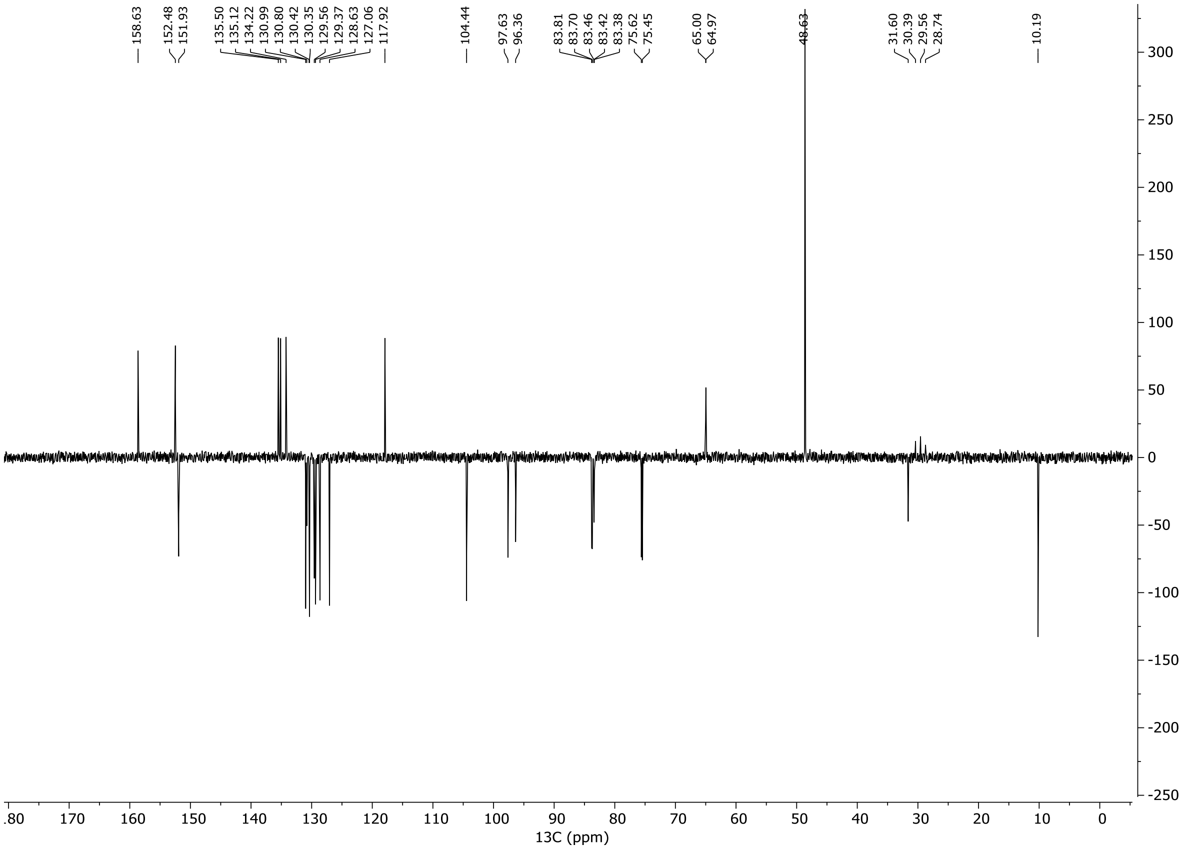
158.63
152.48
151.93
135.50
135.12
134.22
130.99
130.80
130.42
130.35
129.56
129.37
128.63
127.06
117.92
104.44
97.63
96.36
83.81
83.70
83.46
83.42
83.38
75.62
75.45
65.00
64.97
48.63
31.60
30.39
29.56
28.74
10.19
13C (ppm)

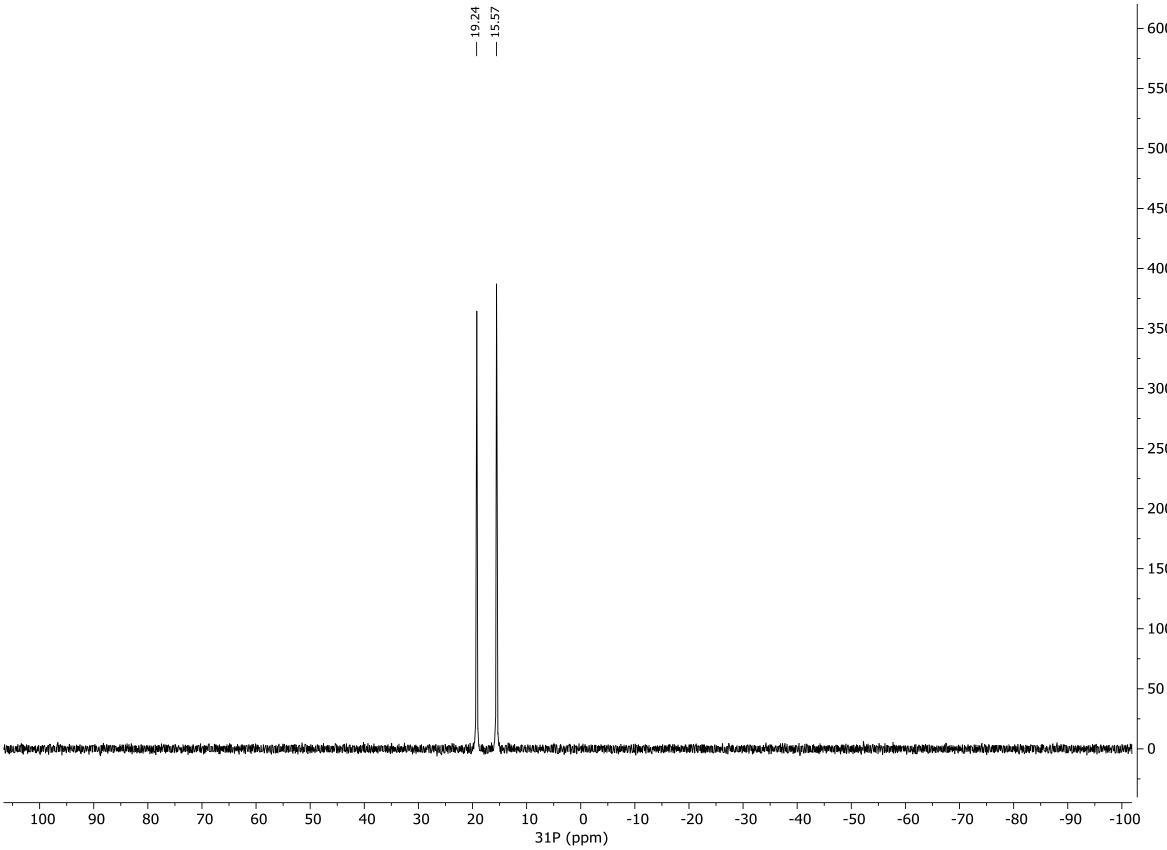
19.24
15.57
31P (ppm)

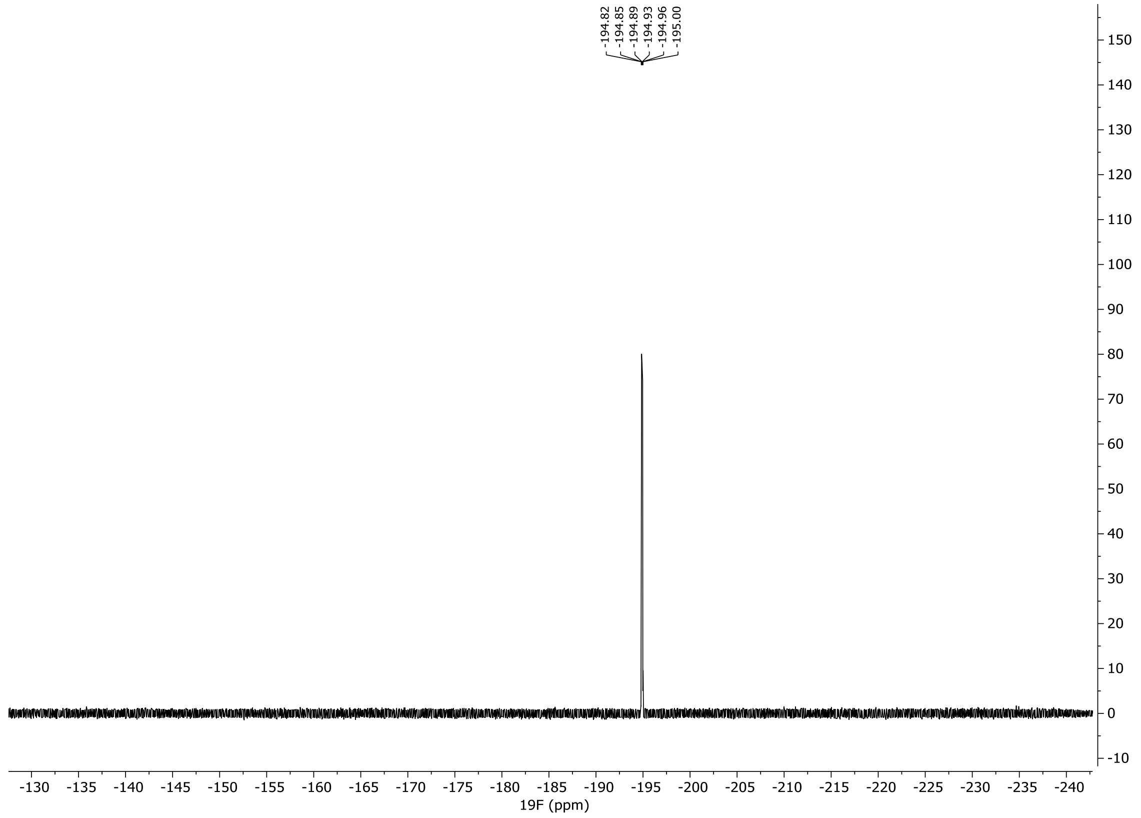

NMR spectra of compound **34A.12**

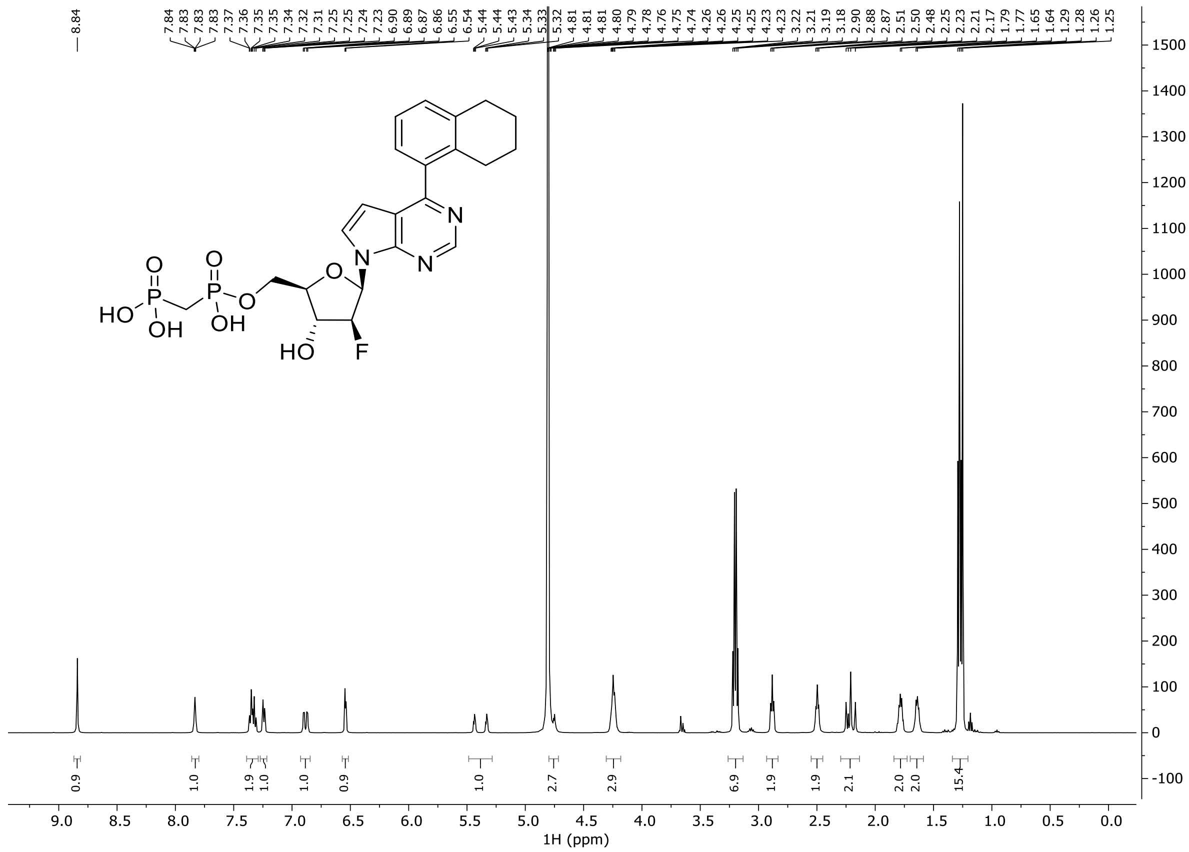

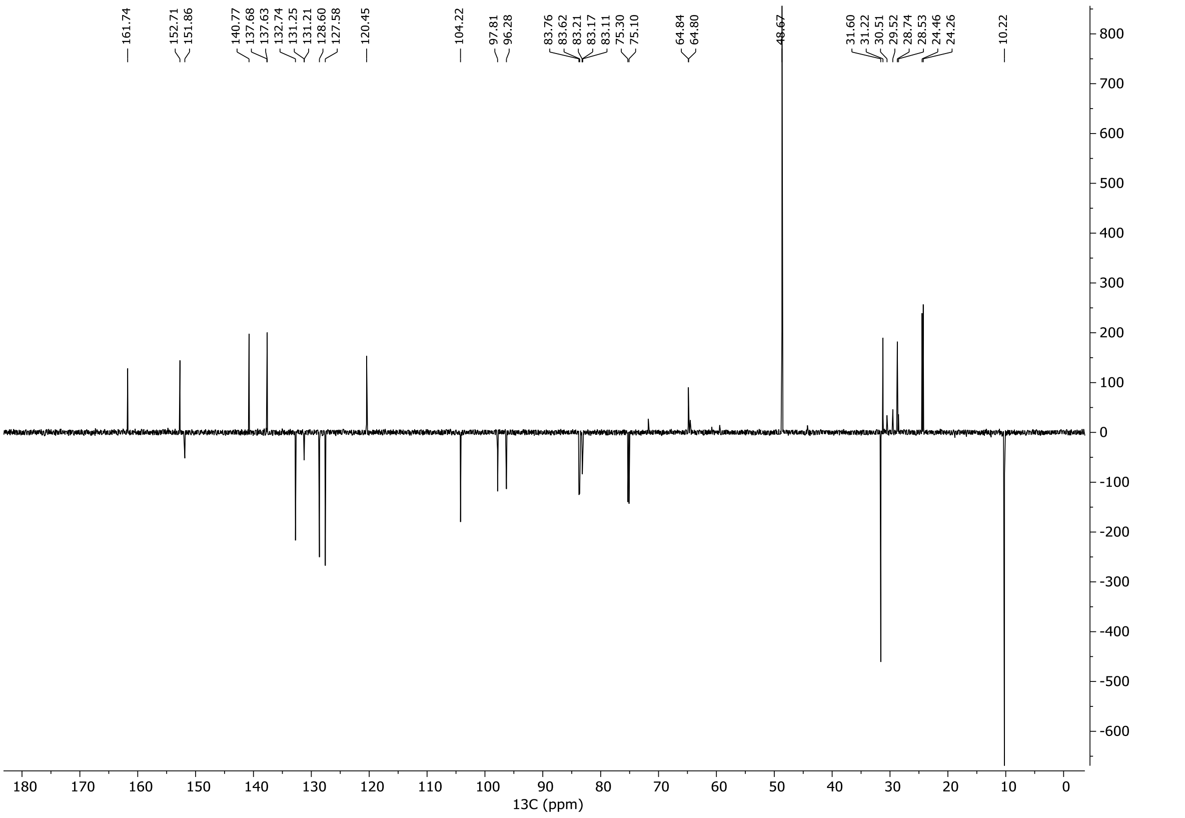
161.74
152.71
151.86
140.77
137.68
137.63
132.74
131.25
131.21
128.60
127.58
120.45
104.22
97.81
96.28
83.76
83.62
83.21
83.17
83.11
75.30
75.10
64.84
64.80
48.67
31.60
31.22
30.51
29.52
28.74
28.53
24.46
24.26
10.22
13C (ppm)

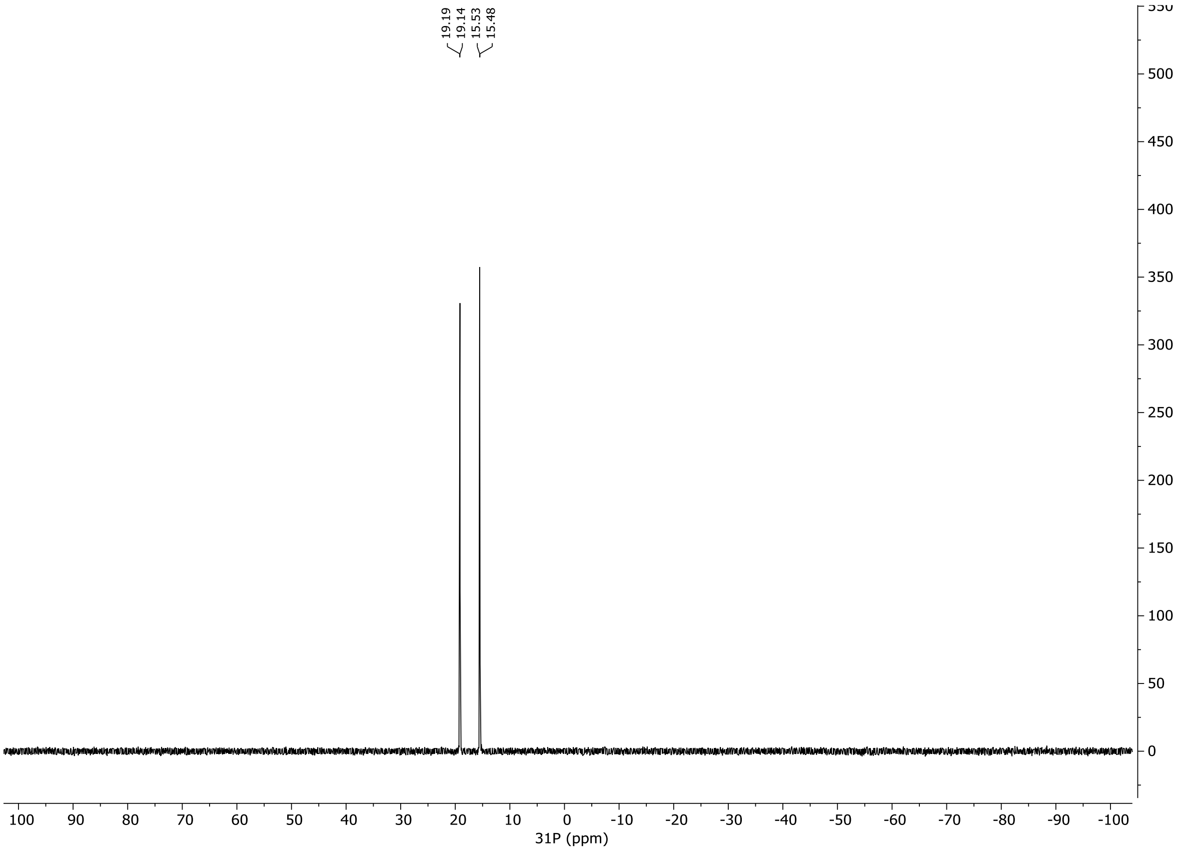
19.19
19.14
15.53
15.48
31P (ppm)

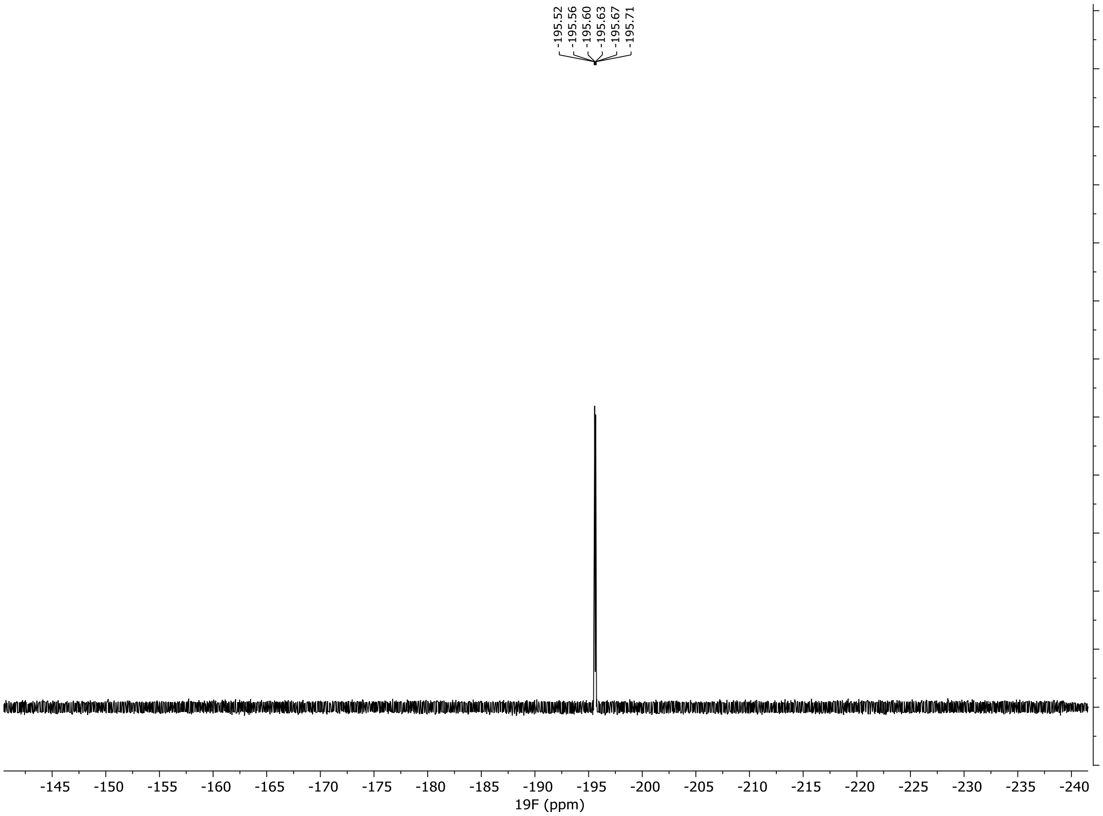

NMR spectra of compound **34A.17**

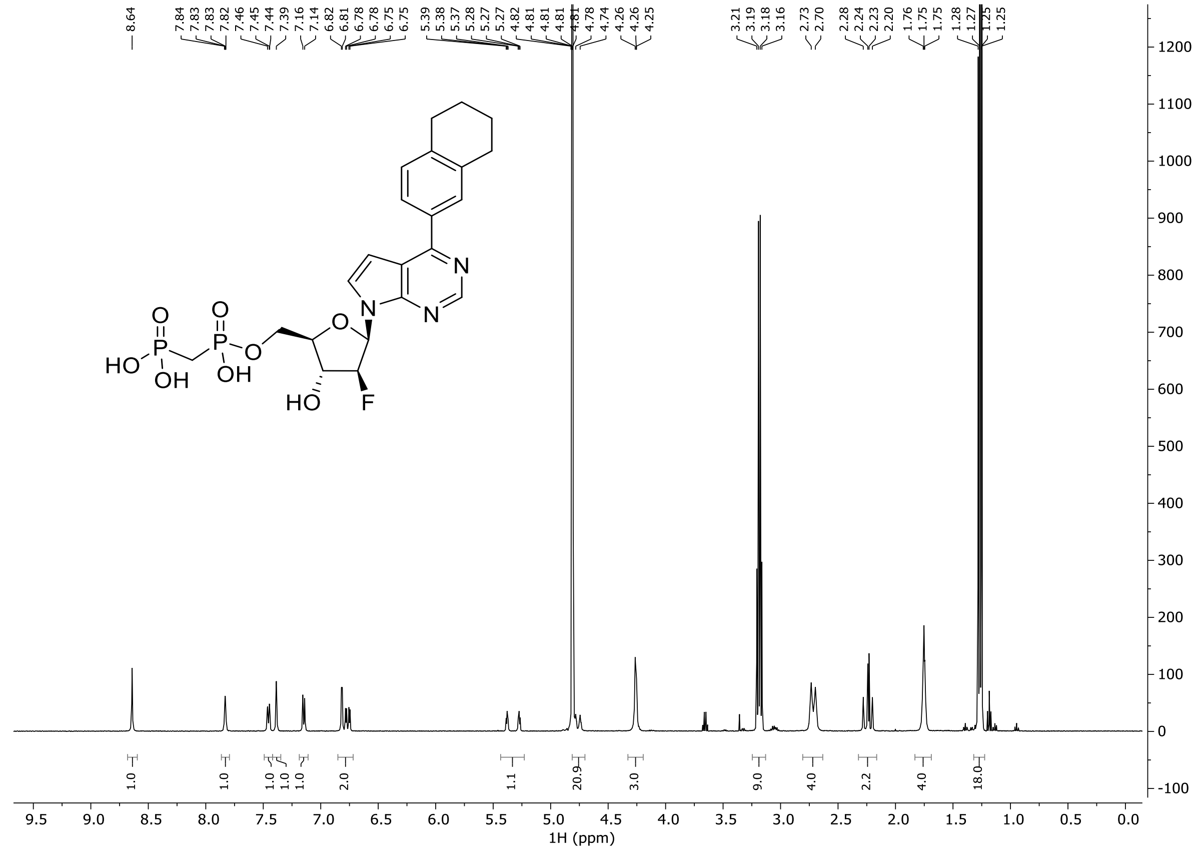

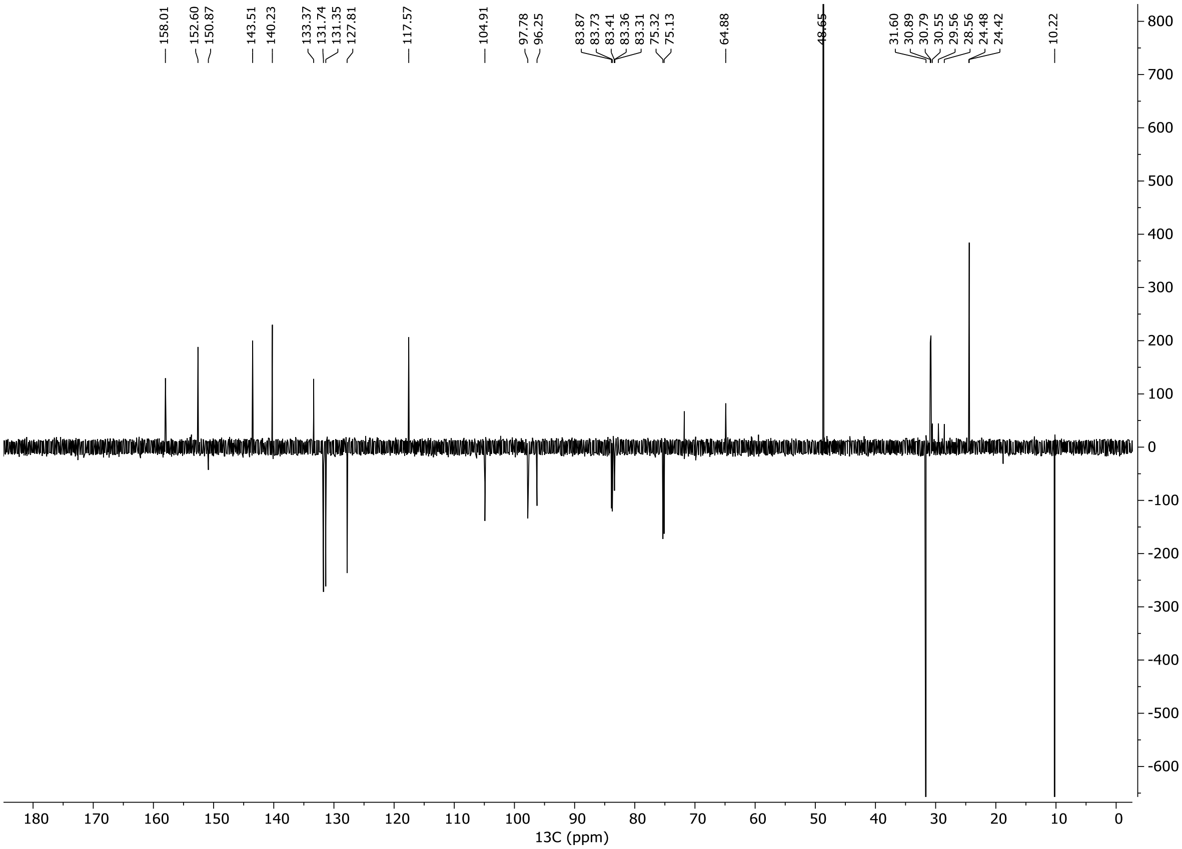
158.01
152.60
150.87
143.51
140.23
133.37
131.74
131.35
127.81
117.57
104.91
97.78
96.25
83.87
83.73
83.41
83.36
83.31
75.32
75.13
64.88
48.65
31.60
30.89
30.79
30.55
29.56
28.56
24.48
24.42
10.22
13C (ppm)

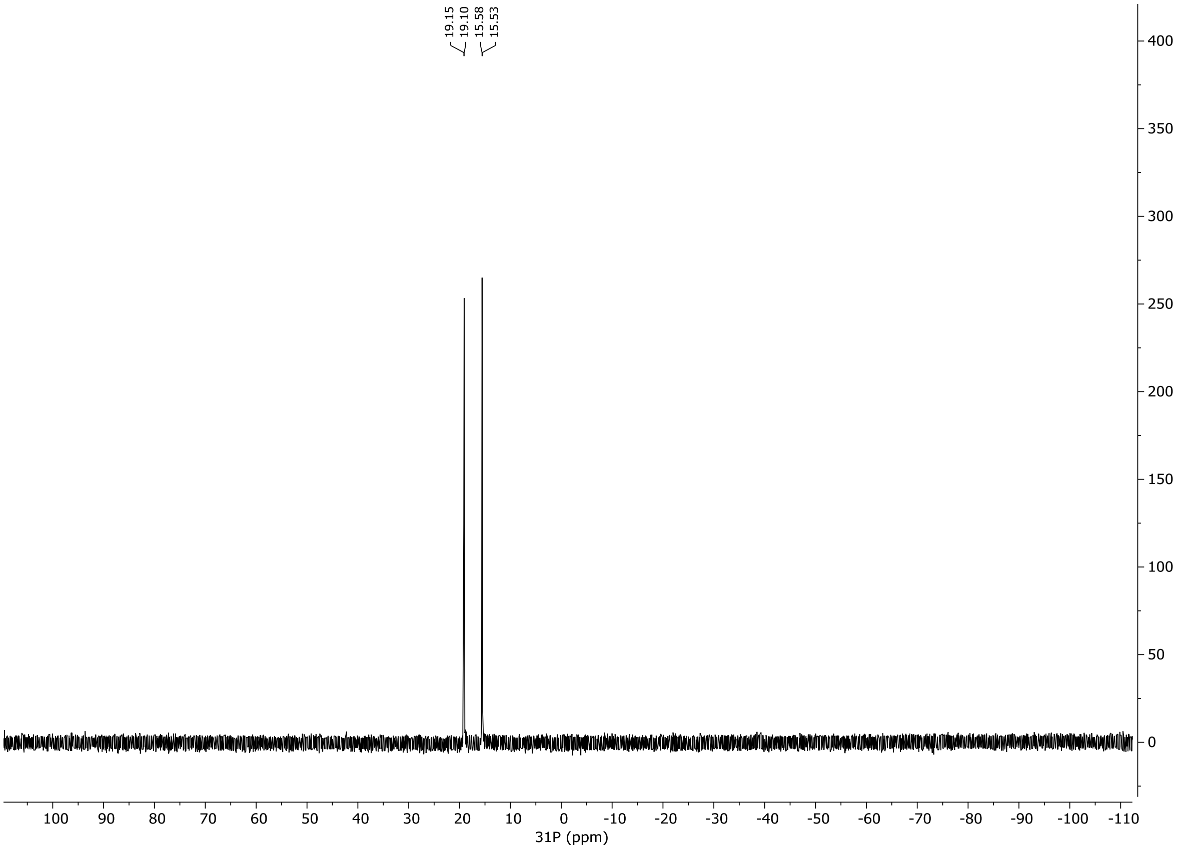
19.15
19.10
15.58
15.53
31P (ppm)

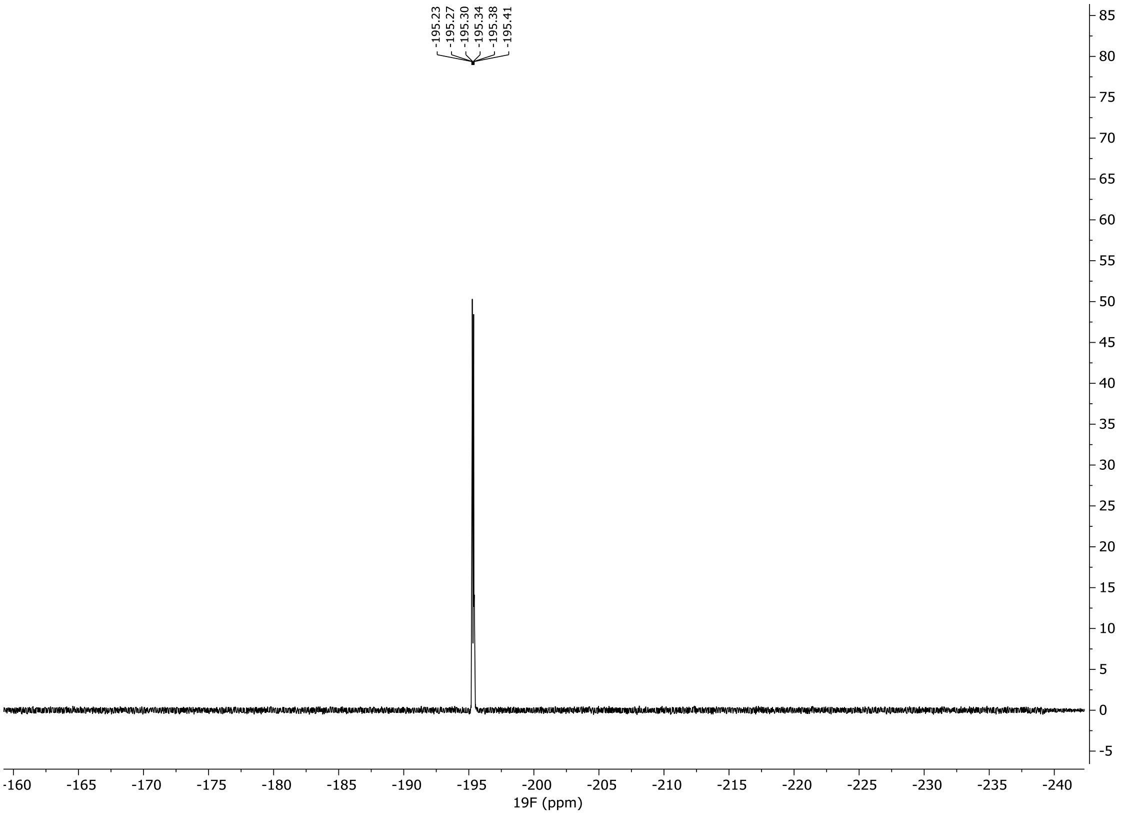

NMR spectra of compound **34A.23**

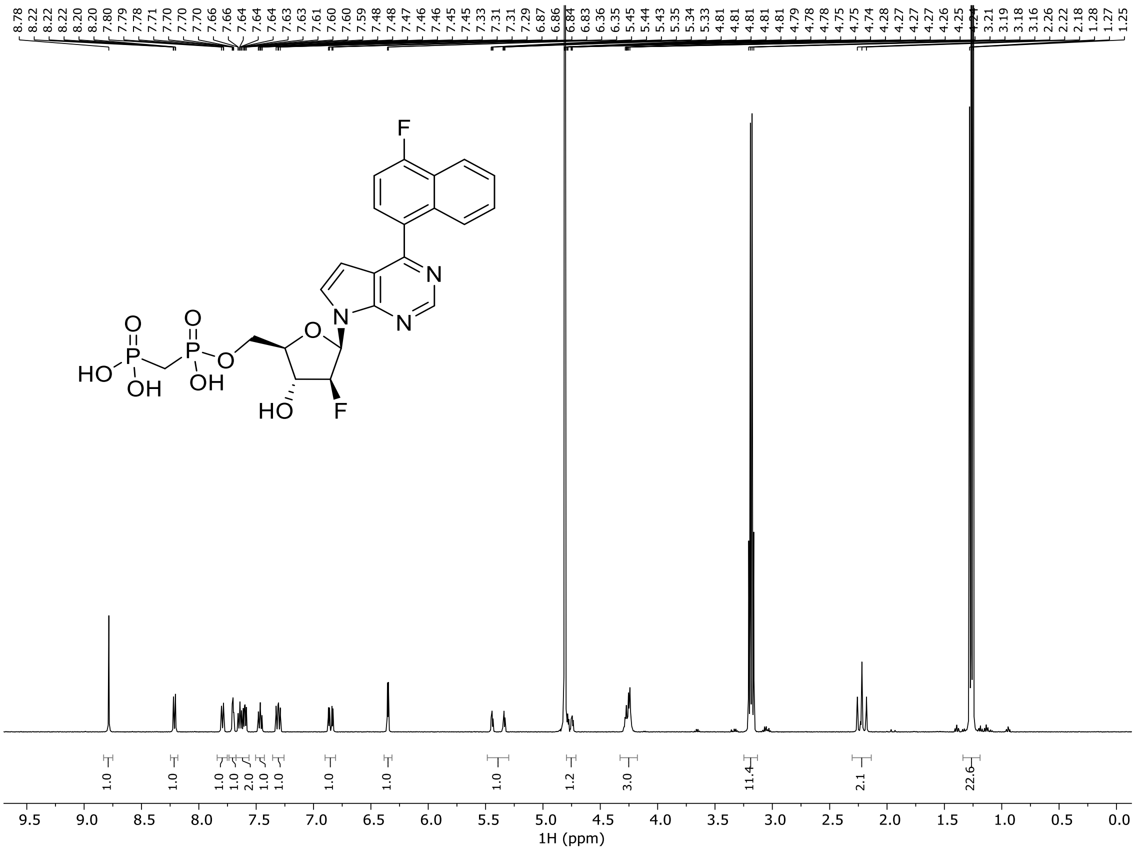

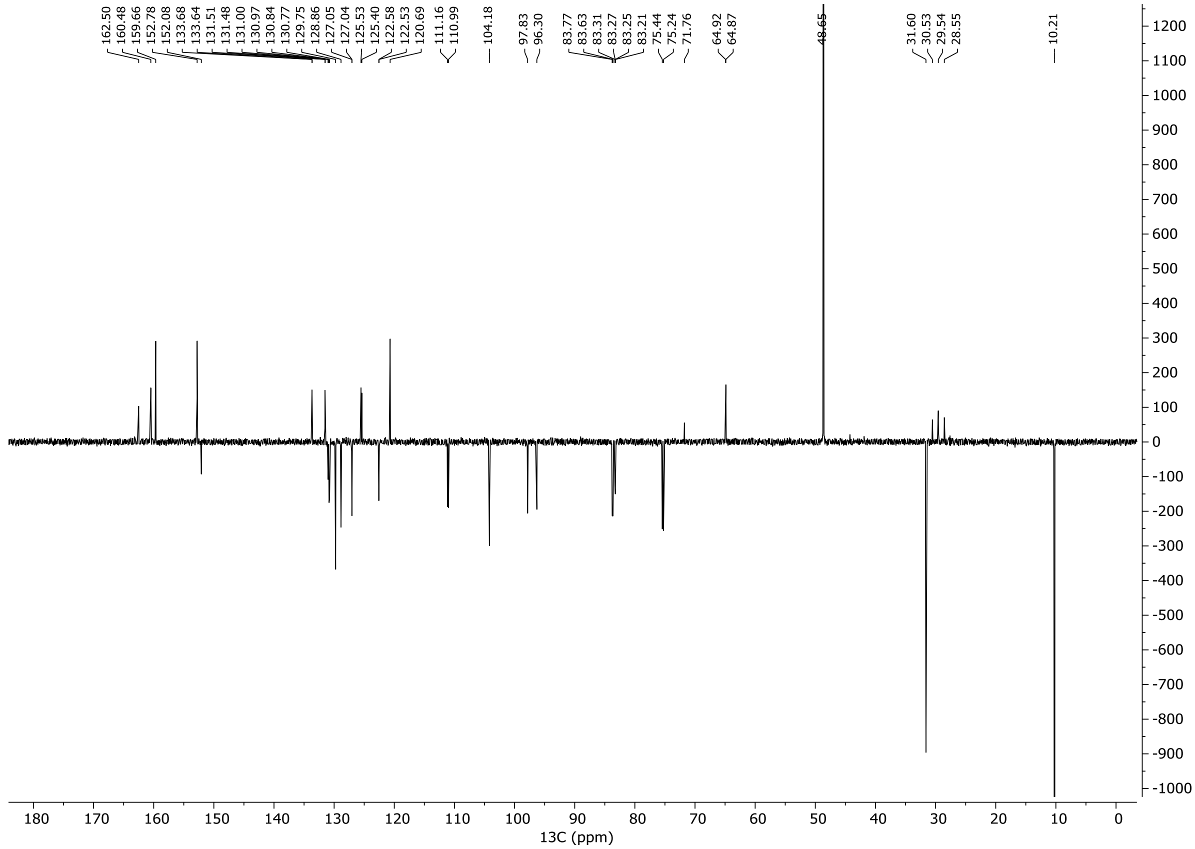
162.50
160.48
159.66
152.78
152.08
133.68
133.64
131.51
131.48
131.00
130.97
130.84
130.77
129.75
128.86
127.05
127.04
125.53
125.40
122.58
122.53
120.69
111.16
110.99
104.18
97.83
96.30
83.77
83.63
83.31
83.27
83.25
83.21
75.44
75.24
71.76
64.92
64.87
48.65
31.60
30.53
29.54
28.55
10.21
13C (ppm)

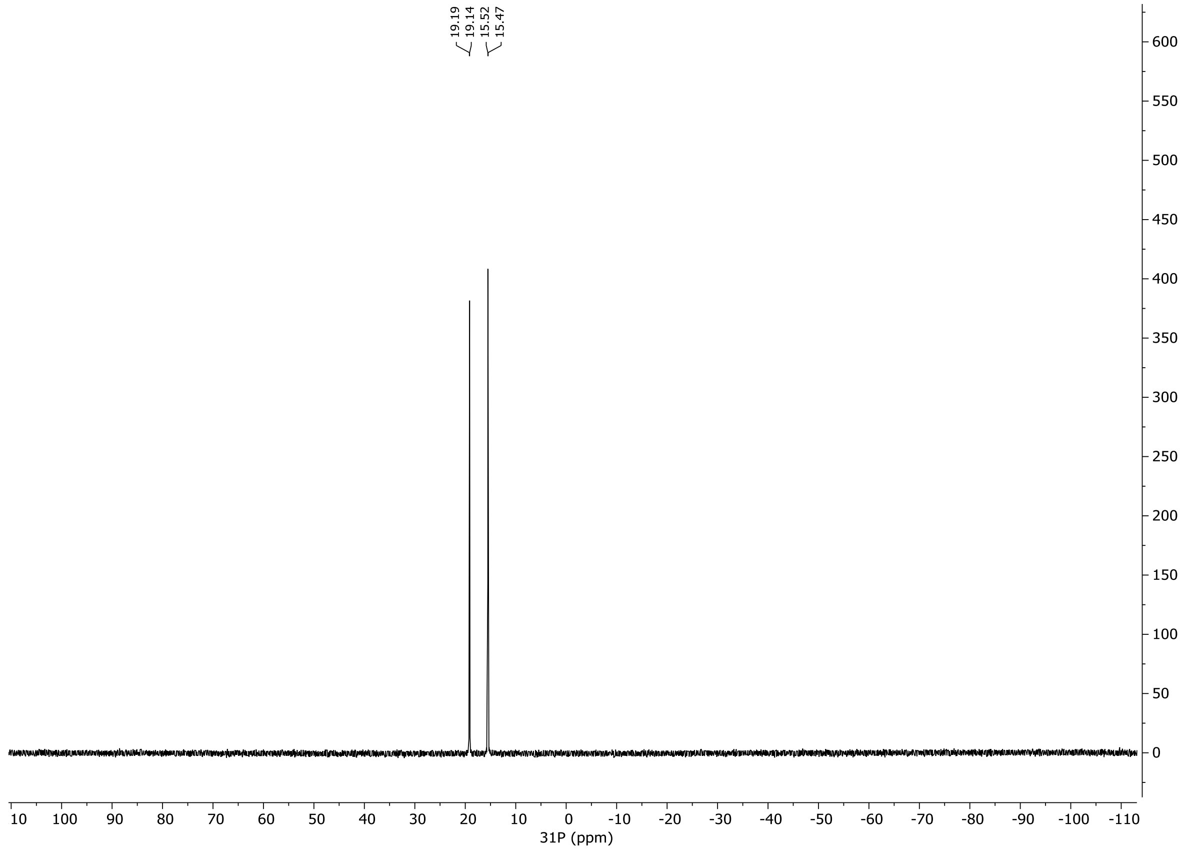
19.19
19.14
15.52
15.47
31P (ppm)

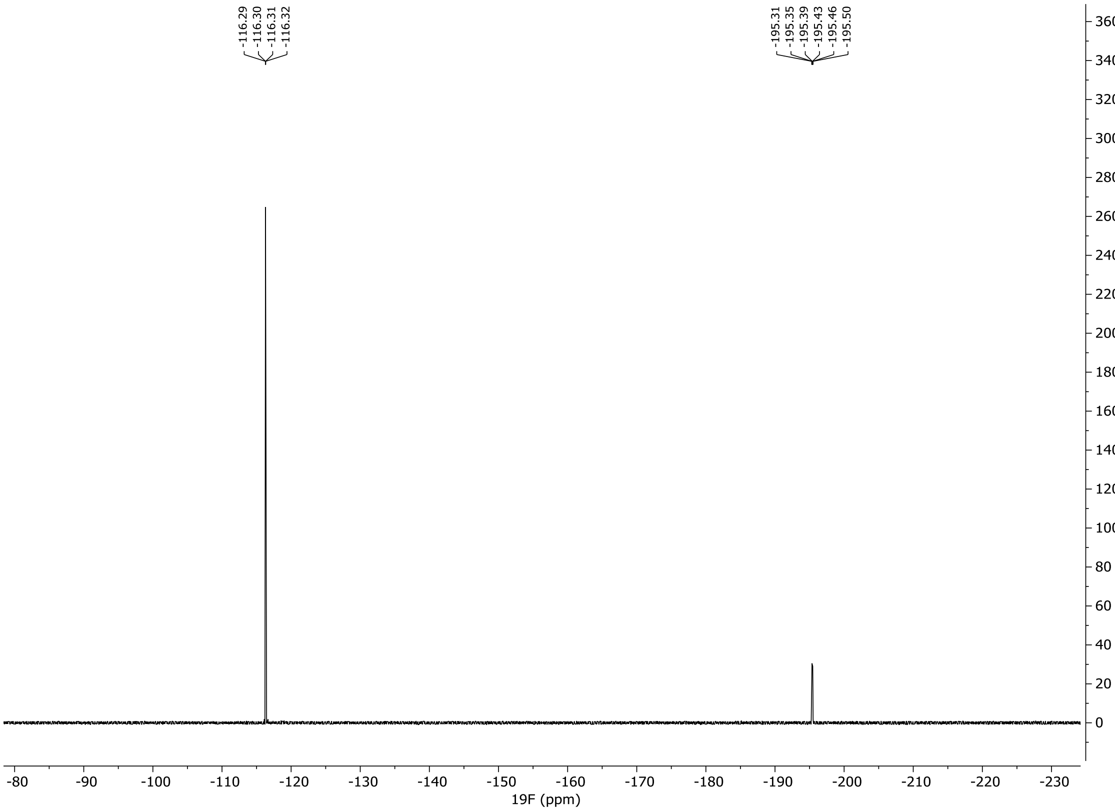

NMR spectra of compound **34A.28**

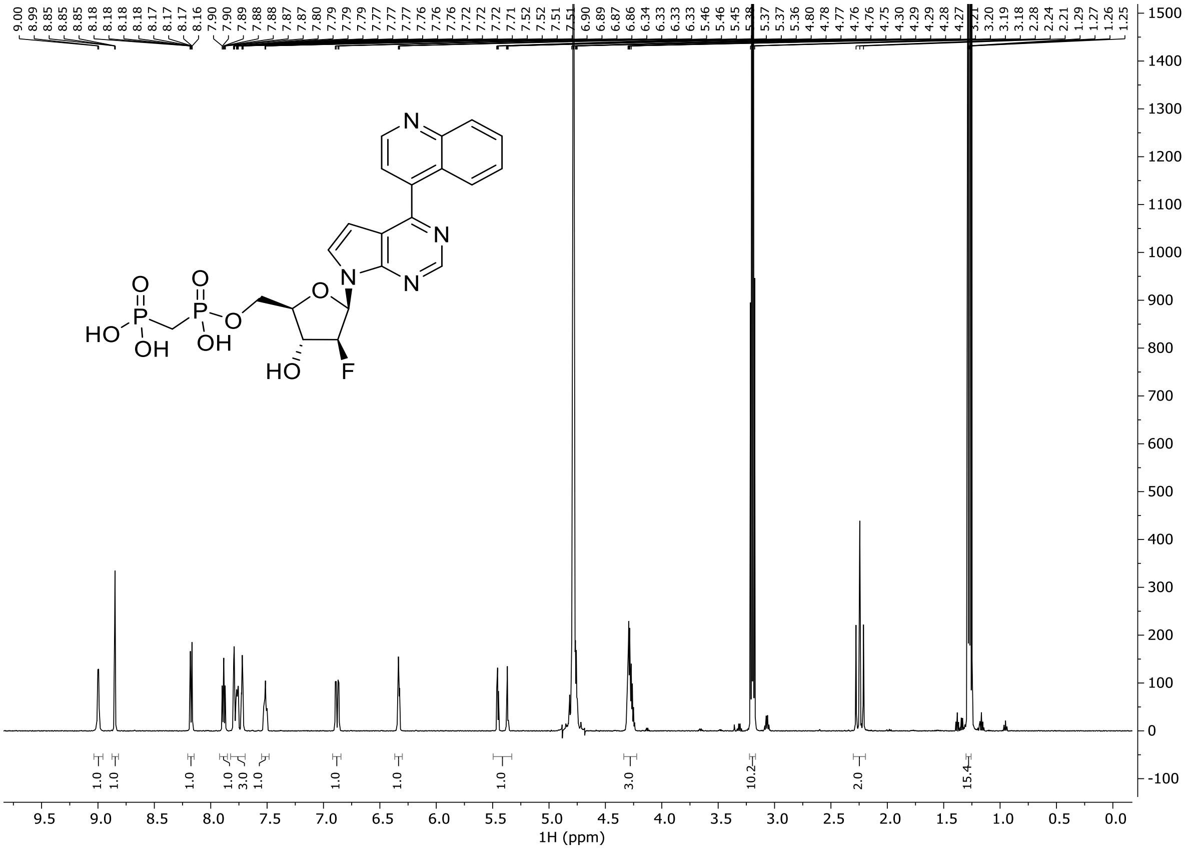

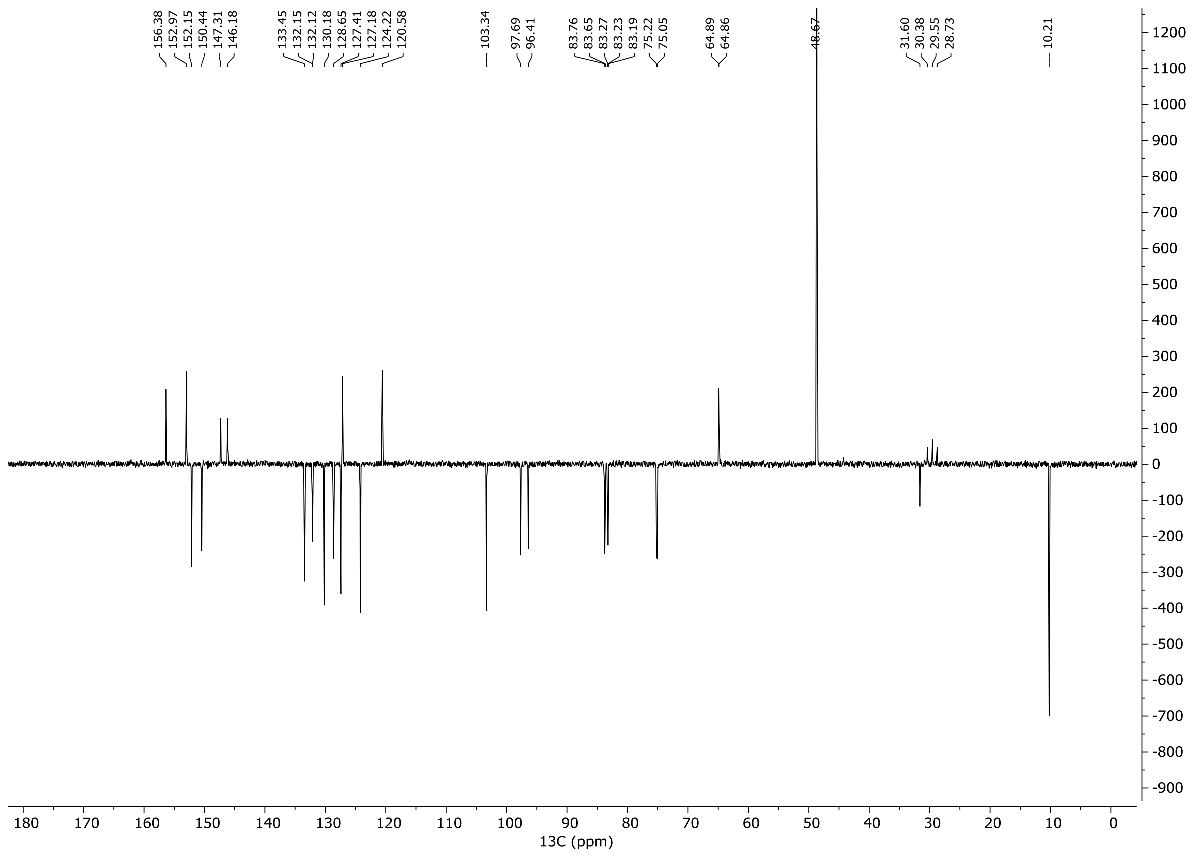
156.38
152.97
152.15
150.44
147.31
146.18
133.45
132.15
132.12
130.18
128.65
127.41
127.18
124.22
120.58
103.34
97.69
96.41
83.76
83.65
83.27
83.23
83.19
75.22
75.05
64.89
64.86
48.67
31.60
30.38
29.55
28.73
10.21
13C (ppm)

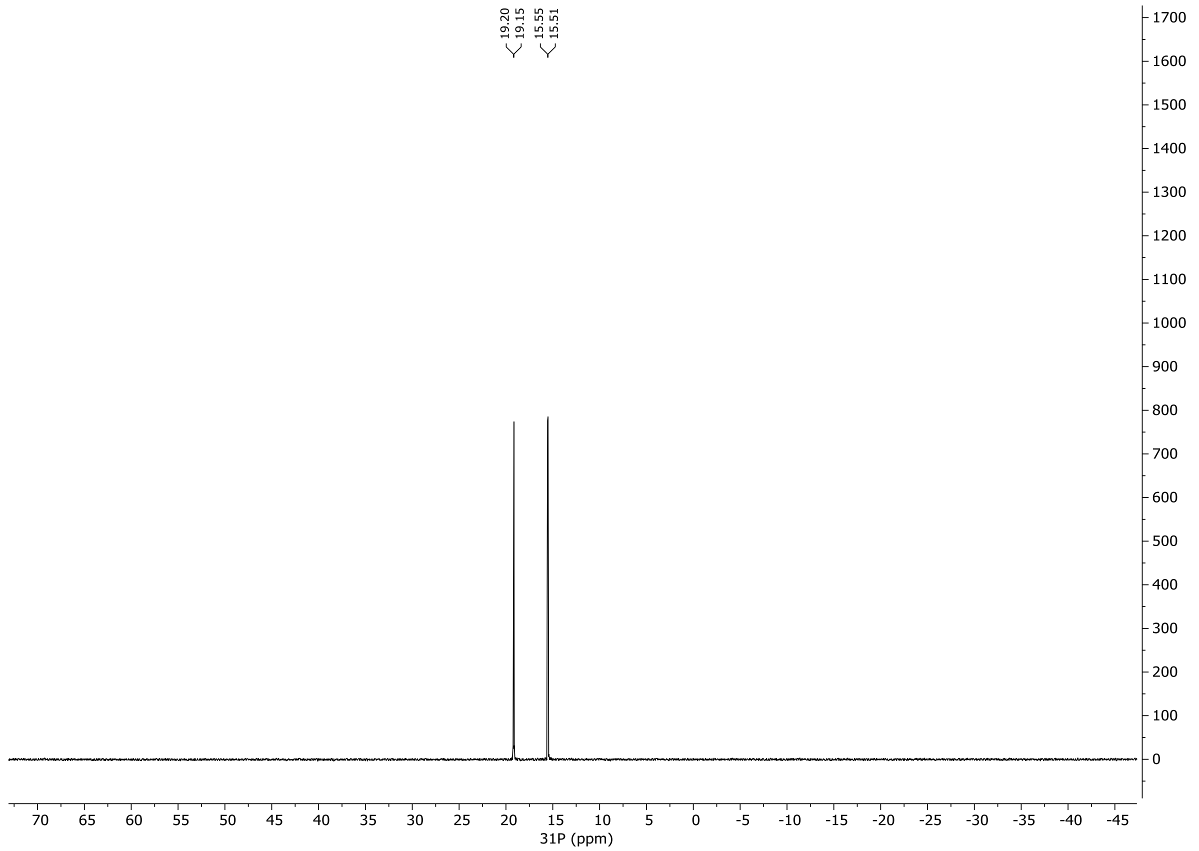
19.20
19.15
15.55
15.51
31P (ppm)

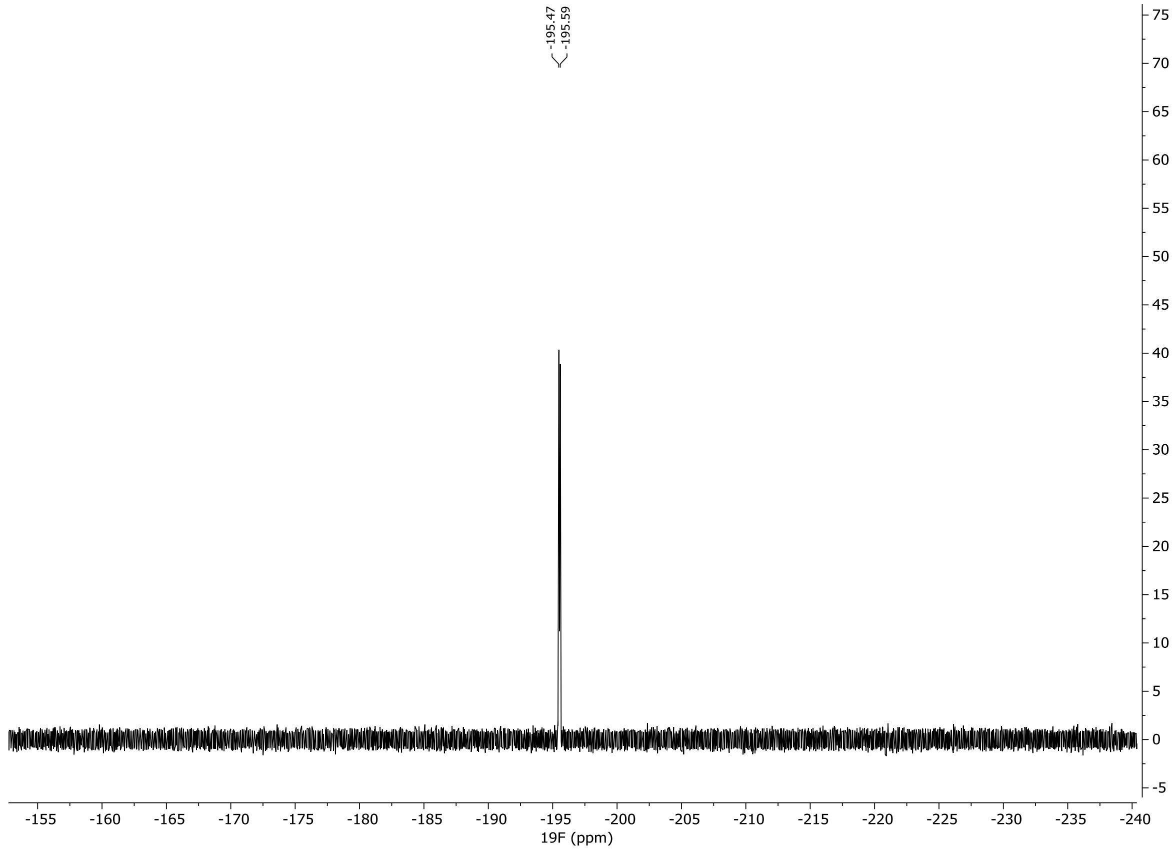
-195.47
-195.59
75
70
65
60
55
50
45
40
35
30
25
20
15
10
5
0
-5
-155
-160
-165
-170
-175
-180
-185
-190
-195
-200
-205
-210
-215
-220
-225
-230
-235
-240
19F (ppm)

References

1 Lawson, K. V.; Kalisiak, J.; Lindsey, E. A.; Newcomb, E. T.; Leleti, M. R.; Debien, L.; Rosen, B. R.; Miles, D. H.; Sharif, E. U.; Jeffrey, J. L.; Tan, J. B. L.; Chen, A.; Zhao, S.; Xu, G.; Fu, L.; Jin, L.; Park, T. W.; Berry, W.; Moschütz, S.; Scaletti, E.; Sträter, N.; Walker, N. P.; Young, S. W.; Walters, M. J.; Schindler, U.; Powers, J. P. Discovery of AB680: A Potent and Selective Inhibitor of CD73. *J. Med. Chem.* **2020**, *63*, 11448–11468. https://doi.org/10.1021/acs.jmedchem.0c00525.
2 Malnuit, V.; Slavětínská, L. P.; Nauš, P.; Džubák, P.; Hajdúch, M.; Stolaříková, J.; Snášel, J.; Pichová, I.; Hocek, M. 2-Substituted 6-(Het)Aryl-7-deazapurine Ribonucleosides: Synthesis, Inhibition of Adenosine Kinases, and Antimycobacterial Activity. *ChemMedChem* **2015**, *10*, 1079–1093. https://doi.org/10.1002/cmdc.201500081.
3 Nauš, P.; Perlíková, P.; Pohl, R.; Hocek, M. Sugar-Modified Derivatives of Cytostatic 6-(Het)Aryl-7-Deazapurine Nucleosides: 2′-*C*-Methylribonucleosides, Arabinonucleosides and 2′-Deoxy-2′-Fluoroarabinonucleosides. *Collect. Czech. Chem. Commun.* **2011**, *76*, 957–988. https://doi.org/10.1135/cccc2011082.
